# Preparation of 1,4-Naphthoquinones and 9,10-Anthraquinones through Brønsted Acid-Catalyzed [4+2] Cycloaddition

**DOI:** 10.1021/acs.orglett.6c02602

**Published:** 2026-07-19

**Authors:** Kai Feuer, Peter R. Schreiner

**Affiliations:** Institute of Organic Chemistry, Justus Liebig University, 35392 Giessen, Germany

## Abstract

We report the synthesis of substituted 1,4-naphthoquinones (1,4-NQs) and 9,10-anthraquinones (9,10-AQs) via Brønsted acid-catalyzed [4+2] cycloaddition of simple butadienes and *p*-benzoquinones to give 5,8-dihydro-1,4-NQs under ambient conditions in up to 90% yield. Subsequent dehydrogenation gives the corresponding 1,4-NQs (62–98%), while a second Brønsted acid-catalyzed [4+2] cycloaddition (that can be performed in one pot) generates 1,4,5,8-tetrahydro-9,10-AQs (71–88%). This enables the synthesis of a variety of highly substituted quinones and acenes.

## Graphical abstract

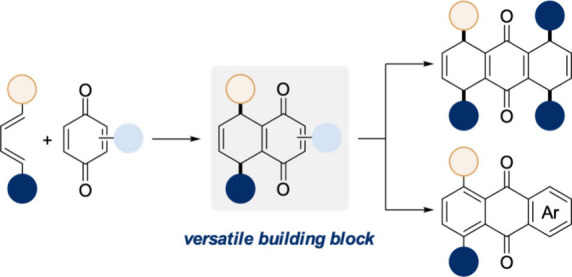

1,4-Naphthoquinones (1,4-NQs) and 9,10-anthraquinones (9,10-AQs) are common motifs in natural products, and their redox properties are key in biological processes.^1–3^ Consequently, 1,4-NQs and 9,10-AQs are also used as drug candidates,^4^ in redox flow batteries,^5–7^ for fluorescence detection,^8,9^ and in H_2_O_2_ production,^10,11^ semiconductors,^12,13^ and photocatalysts.^14–17^

This broad applicability has led to multiple methods for the preparation of substituted quinones. While modifications on the electrophilic quinone ring in 1,4-NQs are well-studied,^18,19^ modifications on the benzenoid ring in 1,4-NQs are challenging. Various *de novo* approaches have been established, including oxidations,^20–22^ Dötz benzannulation,^23,24^ Hauser–Kraus annulation,^25,26^ Friedel–Crafts reactions of phthalic anhydrides and substituted benzenes,^27^ and [2+2+2] cycloaddition (Figure 1).^28,29^ More recently, transition metal-catalyzed *o*-C–H bond hydroxylations and halogenations were used to prepare 1,4-NQs substituted in the benzenoid core.^30–33^ Kakiuchi et al. demonstrated that di- or tetrasubstituted 9,10-AQs can be synthesized by ruthenium-catalyzed *o*-C–H,^34^ *o*-C–O,^35^ or *o*-C–F bond activation.^36^ Bower et al. also demonstrated the functionalization of C6 and C7 with the synthesis of naphthoquinones that undergo cycloaddition or nucleophilic addition.^37^

**Figure 1:**
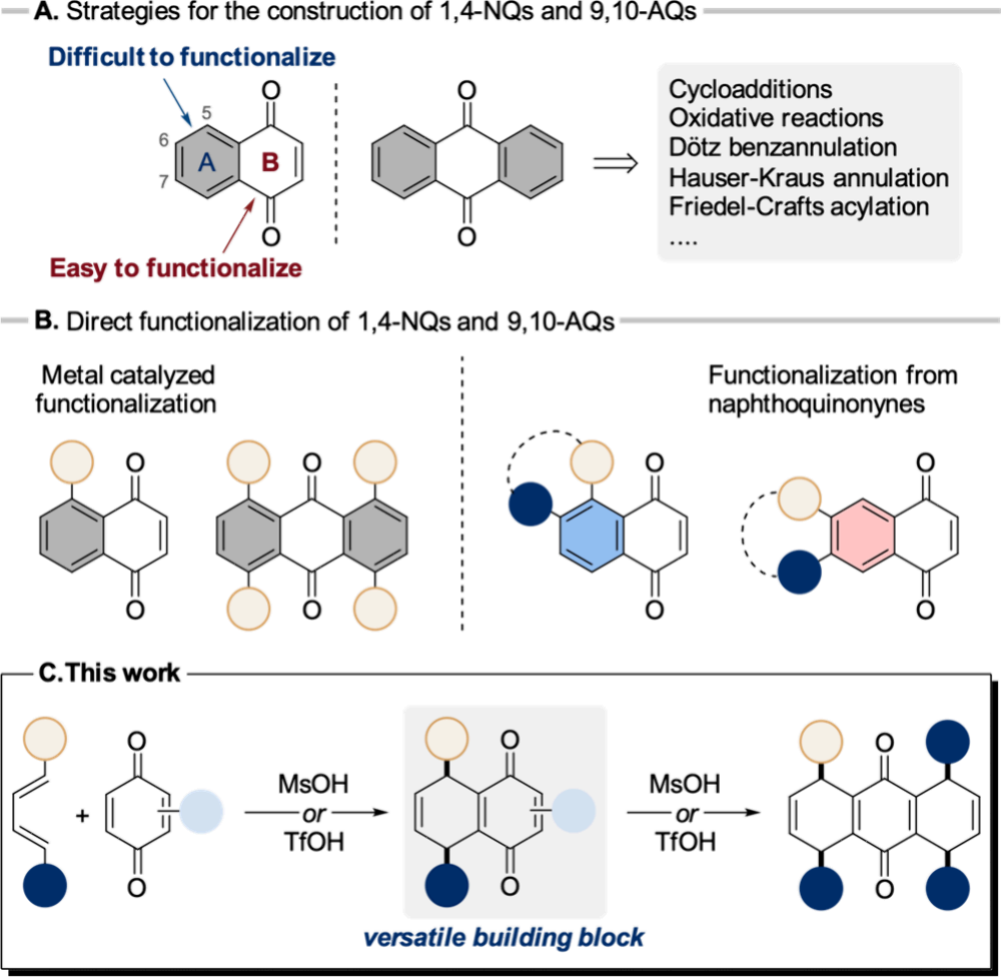
Common strategies for the synthesis of 1,4-naphthoquinones as well as 9,10-anthraquinones and our approach.

The Diels–Alder (DA) reaction has a long history for the preparation of NQ and AQ cores, starting with the 1928 report on the reaction of *p*-benzoquinone (*p*BQ) with cyclopentadiene.^38,39^ Since then, the control of reactivity, regioselectivity, and stereoselectivity has been intensively studied through the introduction of substituents on the dienophile double bond,^40–42^ the synthesis of reactive dienes,^43–45^ or the use of Lewis acids or organocatalysts.^46–60^ The earliest examples of Brønsted acid-catalyzed DA reactions date back to Wassermann’s work in 1942;^50^ Braude et al. used trichloroacetic acid to synthesize substituted 9,10-AQs,^61^ and Fieser employed glacial acetic acid as a solvent for the synthesis of 1,4-NQ from *p*BQ.^62^ Sulfonic acids, such as Nafion-H or *p*TsOH, are also effective catalysts.^51,52^ More recently, chiral *N*-triflyl phosphoramide and chiral phosphoric acids improved reactivities and yields, along with stereoinduction in the DA reaction of 1,4-NQ and *p*BQs.^53,54^ A common difficulty associated with the DA reaction of quinones and Brønsted acids is the stability of the DA adduct. In particular, cycloadditions in AcOH often isomerize to hydroquinones. However, *in situ* oxidation to the corresponding 5,8-dihydro-1,4-NQs is possible with an excess of quinone.

Only a few examples for the synthesis of 5,8-disubstituted 1,4-NQs and 1,4,5,8-tetrasubstituted 9,10-AQs are available.^34–36,48,49,54,55,63,64^ The DA reaction is ideal for the synthesis of such compounds as it tolerates various substituents. Here, we demonstrate the use of strong Brønsted acids that catalyze the reaction of various 1,3-butadiene and *p*BQ starting materials to form dihydro-1,4-NQs under ambient conditions with short reaction times. A second DA reaction under similar conditions enables access to substituted tetrahydro-9,10-AQs. To the best of our knowledge, the reaction of similar dienes and *p*BQ has only been reported under thermal conditions or being mediated by Lewis acids.^48,49,63,64^ The modularity of our approach enables the synthesis of a variety of substituted 1,4-NQs and 9,10-AQs (Figure 1). These *peri*-substituted quinones are ideal building blocks for the synthesis of polyaromatic hydrocarbons, in particular those that have a twisted backbone, like 1,4,5,8,9,10-hexaphenylanthracene.^34^ Additionally, we demonstrate through various straightforward functionalizations that polycyclic compounds, like tetrasubstituted azaanthraquinones or 1,4-disubstituted 5,14-pentacenediones, can readily be synthesized in a few steps from *p*BQ.

In our synthesis of 1,4,5,8-tetraphenyl-9,10-anthraquinone from 1,4-diphenyl-1,3-butadiene **1a** and *p*BQ, we first used thermal conditions, as this reaction yields the corresponding 1,4-NQ in 80%.^64^ This, however, gave an only 10% yield of the expected product. Inspired by the Lewis acid approach of similar substrates of the Wang group,^48,49^ various Lewis acids and strong Brønsted acids were tested in the reaction of **1a** with *p*BQ (Table S1). As noted above, DA product **3a** readily reacts further in the presence of acid and *p*BQ.^54,62^ We optimized the reaction to maximize the yield of **4a**, as the quinone moiety and its reactivity would be useful for a second DA reaction. Therefore, at least 2 equiv of *p*BQ is necessary, as *p*BQ serves to oxidize **3a** to **4a**. Among the tested acids, AlCl_3_ provided a combined 80% yield of **3a** and **4a**, in an 82.5:17.5 ratio, within 24 h. In comparison, the reactions of MsOH and TfOH were completed after 4 h and 15 min, respectively, providing **4a** as the sole product. Further optimization gave yields of the DA reaction comparable to AlCl_3_ (Tables S2 and S3), but maintaining the short reaction times. In the case of MsOH, the reaction was performed with 2 equiv of *p*BQ at room temperature and 20 mol % catalyst (Scheme 1). Note that lower catalyst loading gave similar yields, but at longer reaction times. We ran the TfOH reaction as a fed-batch process, with the dropwise addition of a solution of **1a** to a solution of *p*BQ and 2.5 mol % catalyst at 0 °C within 15 min (Scheme 1). To ensure complete conversion of **3a** to **4a** the reaction mixture was stirred for another 45 min at rt.

**Scheme 1:**
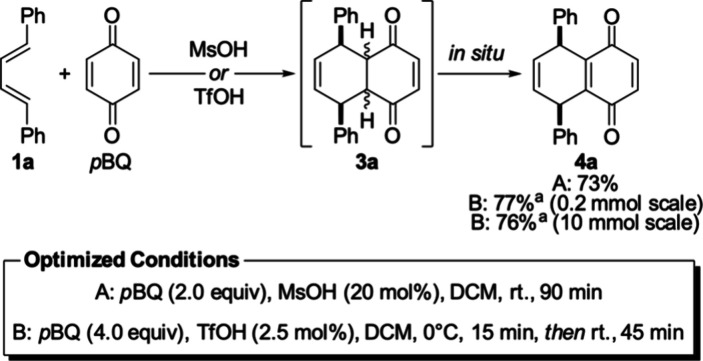
Optimized Reaction Conditions

a Fed-batch processes.

Next, we investigated the applicability of the reaction conditions using different substituents on diene **1** (Scheme 2). In general, electron-donating (EDG) and electron-withdrawing (EWG) functional groups are tolerated, either on one of the aryl groups or on the diene. In the case of monosubstitution on an aryl group, EDGs and EWGs significantly differ. While TfOH could be used for EWGs, significant degradation of the diene occurred for EDGs. Satisfactory yields for dienes with EDGs were achieved with MsOH as the catalyst. For most EDGs in the *ortho* or *para* position, the yield slightly decreases (**4b**, **4c**, **4e**–**4g**, and **4j**), while for EWGs, it increases (**4n**–**4p** and **4r**–**4w**). EWG (bromo) or EDG (methyl) *meta* substitution gave yields similar to that of **1a**. Significantly lower yields were obtained for methoxy-substituted dienes **1h**–**1j**. Optimal conditions require the controlled addition of the dienes. However, methoxy-substituted dienes are sensitive to the acidic and oxidative conditions, and diene degradation occurs. As with the weaker EDGs, *meta* substitution gives higher yields than substitution at the *ortho* or *para* position, while yields of *ortho* and *para* substitution are similar. One of the aromatic rings can also be replaced with larger aromatic ring systems (**1k**–**1m**), whereby the reaction time increases. In the case of pyrene, the DA reaction appears to proceed more slowly compared to decomposition of **1m**, resulting in only 18% yield. Replacing an aromatic ring with alkyl groups also decreased the yield (**1x** and **1y**), most likely due to the increased level of degradation under the acidic conditions. We also tested dienes with identical substituents on both aryl groups (*p*-methyl and *p*-bromo) and observed the same trends as for the singly substituted diaryl dienes. While the yield of **4z** decreases slightly compared to that of **4e**, the yield of **4aa** increases slightly compared to that of **4r**. We assume that double substitution with other functional groups will follow this trend. Almost all of the 5,8-dihydro-1,4-NQs **4** can be converted into their corresponding 1,4-NQs **5** with MnO_2_; nitrile **3v** is unsuitable. Our reaction conditions also allow the use of various ethyl dienoates and monosubstituted dienes. For all tested dienes, isolation of **4** via column chromatography was not possible. To obtain **4ab** and **4ac**, we used a combination of reduction to the corresponding 5,8-dihydro-1,4-naphthalenediol, purification, and then oxidation with Ag_2_O. In contrast, we employed direct dehydrogenation to the corresponding 1,4-NQs **5** for **1ad**–**1af**. Both methods also demonstrate that subsequent reduction or dehydrogenation can follow the catalyzed DA reaction.

**Scheme 2:**
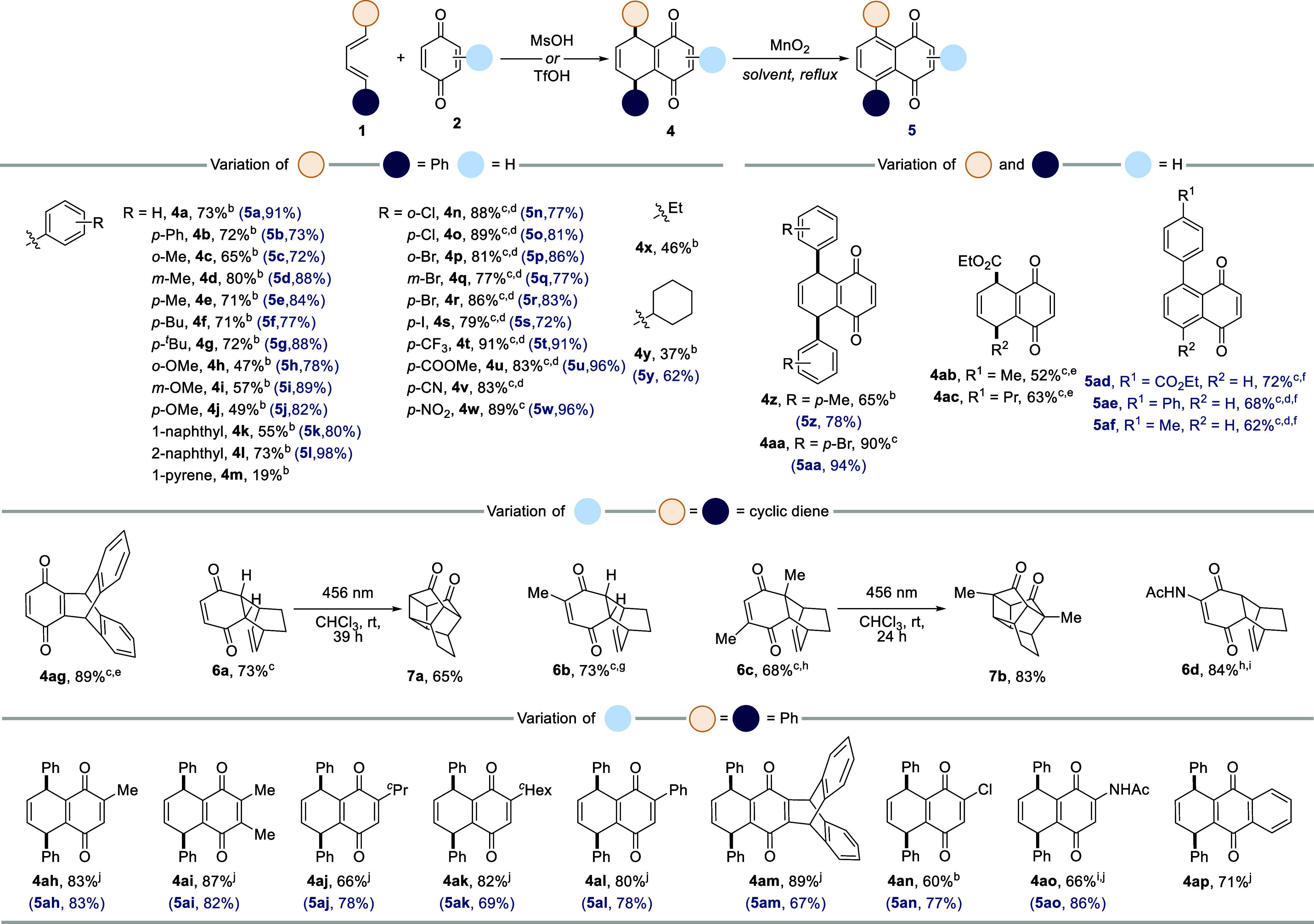
Reaction Scope^a^

a DA reactions were carried out at least on a 0.2 mmol scale.
b MsOH (20 mol %), **2** (2.0 equiv), DCM, rt.
c TfOH (2.5 mol %), **2** (4.0 equiv), DCM, 0 °C.
d Fed-batch process.
e Yield refers to two-step (three-step for **4ag**) synthesis (see the Supporting Information for details).
f Dehydrogenation with MnO_2_ after workup.
g With 2.0 equiv of **2**.
h With 1.3 equiv of **2**.
i An equimolar amount of TfOH was added to **2**.
j TfOH (5 mol %), **2** (2.0 equiv), DCM, 0 °C.

Gratifyingly, the reaction conditions can also be applied to cyclic dienes such as anthracene and cyclohexadiene. For anthracene, the DA adduct was first isomerized and then oxidized to obtain triptycene quinone **4ag** in 89% yield over three steps. Acceleration of the DA reaction with cyclohexadiene is possible as is the variation from *p*BQ to 2-substituted and 2,5-substituted *p*BQ. In all cases, similar yields are obtained, although ratios of starting materials were adjusted. Tricyclic **6a** and **6c** were converted into compounds **7a** and **7b**, respectively, by [2+2] photocyclization at 456 nm.

In addition to variations of the diene, various functional groups on *p*BQ **2** are tolerated. In particular, substitution with EDGs on one double bond gives good yields (**4ah**–**4am**). For **4an**, MsOH was used as an acid, while for **4ao**, an equimolar amount of TfOH was necessary. Instead of *p*BQ, 1,4-NQ can also be used as the dienophile but with somewhat lower yields (76% for *p*BQ to 71% for 1,4-NQ).

Since we already explored the reactivity of substituted *p*BQs, we chose similar reaction conditions for the reaction of **4a** with representative 1,3-butadienes as for the first DA reaction utilizing a 1:1 ratio of dienes **1** and **4a**. We noticed that, upon complete consumption of **4a**, a significant amount of diene did not react. Analysis showed the formation of the corresponding DA adduct, dehydrogenated adduct **8**, and reduced **4a**. We hypothesized that **4a** dehydrogenates the DA adduct to **8** while being reduced. This aligns with the difficulties reported in the acid-catalyzed reaction of a 1:1 mixture of *p*BQ and **1a**.^54^ In the first DA reaction, this problem was circumvented by using excess *p*BQ, but for the second DA reaction, using excess **4a** would not be economical. We assumed that the addition of 1 equiv of *p*BQ would regenerate **4a** to react with unreacted **1** and would convert the DA adduct into **8** (Scheme S2). This proved to be correct, and after adjustment, similar trends and yields are observed as those in the first DA reaction (Scheme 3A). We also realized a one-pot synthesis, through combination of the MsOH conditions for the first DA and the reaction conditions for the second DA reaction (Scheme 3B). Using this approach, **8a** was obtained in 63% yield, compared to a 54% yield for the two-step procedure. Note that the TfOH conditions for the first step are only suitable if 2 equiv instead of 4 equiv of *p*BQ is used.

**Scheme 3:**
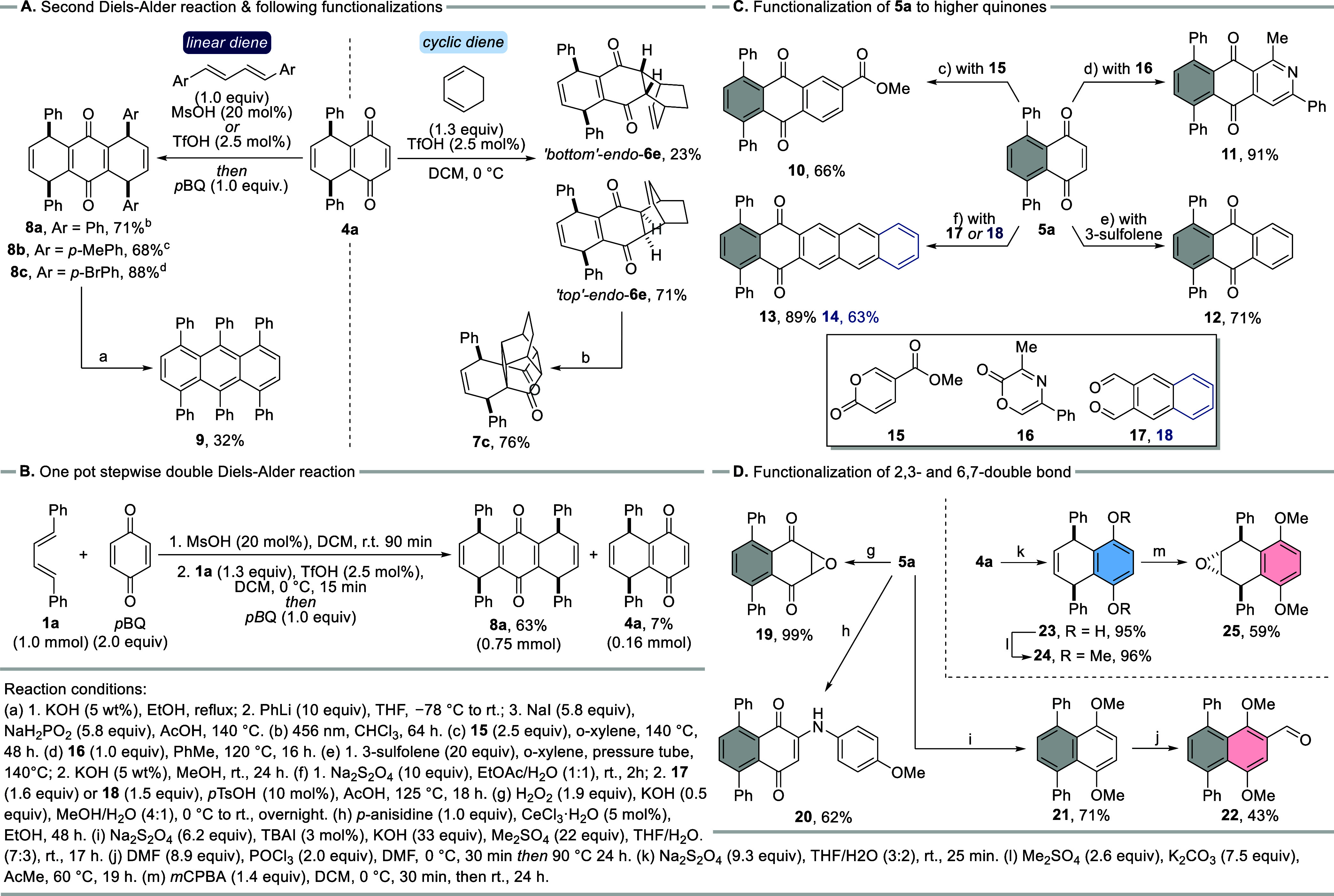
Functionalizations of **4a** and **5a** ^a^

a DA reactions were performed on a 0.2 mmol scale. For other reactions, see the Supporting Information.
b TfOH (2.5 mol %).
c MsOH (20 mol %).
d TfOH (5 mol %).

In addition, we demonstrate the transformation of **8a** into challenging 1,4,5,8,9,10-hexaphenylanthracene **9**.^34^ Dehydrogenation under basic oxidative conditions yielded the corresponding 9,10-AQ, which was then converted in two steps into **9** according to literature procedures. Starting from **1a** and *p*BQ, **9** was synthesized in four steps in 20% overall yield. In comparison, the reported three-step synthesis afforded **8** in 13% overall yield.^34^ In the case of cyclohexadiene, two *endo* products were obtained in a 1:3 ratio, resulting from bottom or top cycloaddition (**6e**). Intramolecular [2+2] photocyclization at 456 nm of “top” *endo*-**6e** gives **7c** in 76% yield.

Structure **5a** enables the synthesis of larger polycyclic quinones (Scheme 3C). Thermal DA reactions with 2-pyrone derivatives or 3-sulfolene afford 9,10-AQs **10**–**12**. **5a** can also be used in a modular approach for the synthesis of substituted acenequinones. We synthesized naphthacenedione **13** and pentacenedione **14** in condensations with dialdehyde **17** or **18**, after reduction of **5a**, in 89% and 63% yield, respectively. Besides the functionalization of the 2,3-double bond via DA reactions, **5a** can be used to functionalize the 2,3-double bond, through nucleophilic additions, giving access to **19** or **20** (Scheme 3D). Additionally, the reduction of the quinone moiety with subsequent methyl protection enables, e.g., electrophilic aromatic substitutions, such as the Vilsmeier–Haack reaction. In contrast, **4a** can be used to functionalize the 6,7-double bond, after reduction and methyl protection of the quinone moiety. This was demonstrated by epoxidation of **24** to synthesize **25** in 59% yield. Note that dehydrogenation or reduction can be performed on the crude reaction mixture of the DA reaction, as shown for some substrates in Scheme 2.

In summary, we developed protocols for the DA reaction of 1,4-disubstituted 1,3-butadienes **1** and *p*BQs **2** to synthesize either 5,8-substituted 1,4-NQs **4** or 1,4,5,8-substituted 9,10-AQs **8**. Depending on the diene, we employed either MsOH (for electron rich dienes) or TfOH (for electron poor dienes), allowing the reaction to take place at rt or 0 °C. 9,10-AQ cores can be synthesized in one pot. Through various functionalizations, we demonstrate that **4** and **8** are useful building blocks en route to other polycyclic quinones or acenes. The possibility of using these building blocks in combination with other known DA protocols allows the synthesis of 9,10-AQs with a large variety of substitution patterns.

## Supplementary Material



## Data Availability

The data underlying this study are available in the published article and its Supporting Information.

## Notes

Full experimental procedures and compound characterization (PDF)
