# Supplementary material for: Preparation of 1,4-Naphthoquinones and 9,10-Anthraquinones through Brønsted Acid-Catalyzed [4+2] Cycloaddition
Source: Org Lett. 2026 Jul 19;28(30):9742–7. doi: 10.1021/acs.orglett.6c02602 (PMC13435309; doi:10.1021/acs.orglett.6c02602)
Supplement: Supplementary file 1 [file ol6c02602_si_001.pdf]

Electronic Supporting Information for

Preparation of 1,4-Naphthoquinones and 9,10-Anthraquinones through Brønsted-Acid Catalyzed [4+2] Cycloaddition

Kai Feuer[a] and Peter R. Schreiner*[a]

*Corresponding author

[a]Institute of Organic Chemistry, Justus Liebig University, 35392 Giessen, Germany

E-mail: prs@uni-giessen.de

Table of Contents

1. General Information 3

2. Analytical Methods 3

3. General Procedures 3

4. Reaction of (1*E*,3*E*)-1,4-diphenyl-1,3-butadiene 1a and *p*-benzoquinone at 10 mmol scale 5

5. Optimization of Catalyzed Diels-Alder Reaction 6

6. Proposed Mechanism and Reaction Sequence 8

7. Synthetic Procedures and Analytical Data 10

7.1 Overview of Substrate Synthesis 10

7.2 Synthesis and Analytical Data of Dienes 12

7.2.1 (1*E*,3*E*)-1,4-Diphenyl-1,3-butadiene (1a) 12

7.2.2 1-Substituted (1*E*,3*E*)-4-phenyl-1,3-butadienes (1b to 1y) 12

7.2.3 1,4-Diaryl substituted (1*E*,3*E*)-1,3-butadienes (1z and 1aa) 23

7.2.4 Substituted ethyl (1*E*,3*E*)-1,3-butadienoates (1ab to 1ad) 27

7.2.5 Substituted (*E*)-1,3-butadiene (1ae and 1af) 28

7.3. Synthesis and Analytical Data of Substituted *p*-Benzoquinones (2) 30

7.4 Synthesis and Analytical Data of 5,8-Dihydronaphthalene-1,4-dione (4) 31

7.5 Synthesis and Analytical Data of 1,4-Naphthoquinones (5) 53

7.6 Synthesis and Analytical Data of 1,4,4a,8a-Tetrahydro-1,4-ethanonaphthalene-5,8-diones (6) . 70

7.7 Synthesis and Analytical Data of 1,4,5,8-Tetrahydroanthracene-9,10-diones (8 and 6e) 73

7.8 Synthesis and Analytical Data of [2+2]-Cycloaddition Products (7) 76

7.9 Synthesis and Analytical Data of Anthra-, Naphthacene- and Pentacenediones 77

7.9.1 Synthesis and Analytical Data of Reagents (16 and 18) 77

7.9.2 Synthesis and Analytical Data of Anthracene-9,10-diones (10 to 12) 78

7.9.3 Synthesis and Analytical Data of Naphthacene- and Pentacenedione (13 and 14) 80

7.10 Synthesis and Analytical Data of Functionalized 5,8-Dihydro-5,8-diphenylnaphthalene-1,4-dione and 5,8-Diphenylnaphthalene-1,4-dione 81

8. NMR Spectra 86

9. References 245

1. General Information

All Chemicals were purchased from abcr GmbH, Acros Organics, BLDpharm, Carl Roth, TCI or Sigma Aldrich in the highest purity grade possible and, unless otherwise noted, used without purification. *p*-Benzoquinone was sublimed prior to use and stored in the dark under inert atmosphere. All solvents were distilled prior to use. Dry solvents were purchased from Acros Organics. All catalytic reactions were carried out under nitrogen or argon atmosphere. Column chromatography was conducted using Machery Nagel silica gel 60 (0.040 – 0.063 mm) or Merck silica gel 60 (0.040 – 0.063 mm).

2. Analytical Methods

Thin Layer Chromatography (TLC) was performed on plastic-backed silica gel 60 plates coated with a fluorescence indicator by Machery Nagel. Visualization was performed by UV light (254 nm or 356 nm) and/or dinitrophenylhydrazine, potassium permanganate ($KMnO_4$) or ceric ammonium molybdate (CAM) stain.

Dinitrophenylhydrazine stain prepared from 12 g of 2,4-dinitrophenylhydrazine, 60 mL conc. sulfuric acid, 80 mL water and 200 mL ethanol.

Potassium permanganate stain prepared from 1.5 g of $KMnO_4$, 10 g K_2CO_3 and 1.25 mL 10% NaOH and 200 mL water.

CAM stain prepared from 5.0 g of ammonium molybdate tetrahydrate, 2.0 g ammonium cerium(IV) sulfate dihydrate, 20 mL conc. sulfuric acid and 180 mL water.

Nuclear Magnetic Resonance spectra were recorded on a Bruker Avance II 400 MHz, Avance III HD 400 MHz, Avance III HD 600 MHz or Avance Neo 700 MHz at 298 K. The chemical shift in ppm is reported relative to the residual $CHCl_3$ (7.26 ppm/77.16 ppm), CH_2Cl_2 (5.32 ppm/53.84 ppm), AcMe (2.05 ppm/29.84 ppm) or DMSO (2.50 ppm/39.52 ppm) signal. The multiplicity of signal is reported as follows: s – singulet, d – doublet, t – triplet, q – quartet, quint – quintet, sext - sextet or as combinations thereof. ^{31}P-NMR spectra were referenced to H_2PO_4 (0.00 ppm). ^{19}F-NMR spectra are given without reference.

Infrared Spectroscopy (IR) was performed on a Bruker Alpha spectrometer by ATR technique. Signal intensity is assigned as follows: s – strong, m – medium, w – weak.

High Resolution Mass spectrometry (HRMS) was performed on a Bruker Mikro-TOF or Impact II.

Melting points were measured with a Krüss melting point meter KSP1N.

3. General Procedures

General Procedure 1 (GP1) for the Horner-Wadsworth-Emmons reaction with NaH

In an oven-dried Schlenk flask under an inert gas atmosphere, NaH (9.0 mmol, 1.2 equiv; either 90% dry or 60% dispersed in mineral oil, washed three times with dry pentane) was dissolved in 37 mL anhydrous THF and cooled to 0 °C. Then diethyl phosphonate (7.5 mmol, 1.2 equiv) was added carefully and the resulting mixture was stirred for 15 to 30 min. The respective aldehyde (3.5 mmol, 1.0 equiv) was added in one portion and stirred overnight (the cooling bath was not removed, but was allowed to warm to r.t. overnight). After complete consumption of the aldehyde, 30 mL of a 1:1 mixture of sat. NH_4Cl and water was added. The mixture was transferred into a separatory funnel, the phases separated and the aqueous phase extracted with Et_2O (3 x 30 mL). The combined organic phases were dried over $MgSO_4$, filtered and the solvents removed under reduced pressure. The residue was purified by flash column chromatography to yield the desired product.

General Procedure 2 (GP2) for the Arbusow reaction

Under an inert gas atmosphere, the respective halogen (1.0 equiv) and triethylphosphite (1.0 to 1.2 equiv) were placed in a flask with reflux condenser and heated in an oil bath (either first to 90 °C for 2 h, followed by heating to 140 °C or directly to 160 °C). After complete reaction, excessive triethylphosphite was removed by distillation under reduced pressure (approx. 20 Torr and 60 °C), followed by distillation under reduced pressure to obtain the product.

General Procedure 3 (GP3) for the Horner-Wadsworth-Emmons reaction with nBuLi

In an oven-dried Schlenk flask under an inert gas atmosphere diethyl phosphonate (4.2 mmol, 1.2 equiv) was dissolved in 30 mL anhydrous THF and cooled to −78 °C. Then *n*BuLi (1.6 or 2.5 M in hexane, 4.2 mmol, 1.2 equiv) was added carefully and the resulting mixture was stirred for 30 min, maintaining the temperature below −70 °C. The respective aldehyde (3.5 mmol, 1.0 equiv) was added in one portion and stirred overnight (the cooling bath was not removed, but was allowed to warm to r.t. overnight). After complete consumption of the aldehyde, 30 mL of a 1:1 mixture of sat. NH_4Cl and water was added. The mixture was transferred into a separatory funnel, the phases separated and the aqueous phase extracted with Et_2O (3 x 40 mL). The combined organic phases were dried over $MgSO_4$, filtered and the solvents removed under reduced pressure. The residue was purified by flash column chromatography to yield the desired product.

General Procedure 4 (GP4) for the C-H functionalization of p-benzoquinone

In a round-bottom flask under air, *p*-Benzoquinone (2.0 mmol, 1.0 equiv), $AgNO_3$ (0.4 mmol, 0.2 eqiv), the corresponding carboxylic acid (4.0 mmol, 2.0 equiv) *or* the corresponding boronic acid (3.0 equiv, 1.5 equv) and ammonium persulfate (4.0 mmol, 2.0 equiv) *or* potassium persulfate (in the case of boronic acid; 4.0 mmol, 2.0 equiv) weren stirred in 20 mL DCE/H_2O (1:1) *or* 20 mL DCM/H_2O (1:1) until no further reaction is observed. The reaction mixture was poured into 20 mL 5% $NaHCO_3$, the phases were separated and the aqueous phase was extracted with DCM (3 x 25 mL). The combined organic phases were dried over $MgSO_4$, filtered and the solvent was removed under reduced pressure. The residue was purified by flash column chromatography to yield the desired product.

General Procedure 5 (GP5) for the catalyzed Diels-Alder reaction

Under an inert gas atmosphere, the respective diene (0.2 mmol, 1.0 equiv) and respective *p*-benzoquinone (0.4 mmol or 0.8 mmol, 2.0 equiv or 4.0 equiv) were dissolved in 2 mL anhydrous DCM (0.1 M). Then MsOH (0.04 mmol, 0.2 equiv) *or* TfOH (0.005 mmol, 2.5 mol%) was added and the reaction was stirred at r.t. until completion. The reaction was ended by the addition of 2 mL sat. $NaHCO_3$ and was diluted with 2 mL water and 2 mL DCM or DCM/EtOAc (3:1). The phases were separated and the aqueous phase was extracted with DCM or DCM/EtOAc (3:1; 3 x 4 mL). The combined organic phases were dried over $MgSO_4$, filtered and the solvent was removed under reduced pressure. The residue was adsorbed on Celite® and purified by flash column chromatography to yield the desired product.

General Procedure 6 (GP6) for the fed-batch catalyzed Diels-Alder reaction

In a pear-shaped flask under an inert gas atmosphere, the respective diene (0.2 mmol, 1.0 equiv.) was dissolved in anhydrous DCM (for exact values see below) and taken up into a syringe. In a second flask under an inert gas atmosphere, the respective *p*-benzoquinone (0.8 mmol, 4.0 equiv) was dissolved in anhydrous DCM and cooled with an ice-water bath to 0 °C. Then MsOH (0.04 mmol, 0.2 equiv) *or* TfOH (0.005 mmol, 2.5 mol%) was added to the *p*-benzoquinone solution, followed by the dropwise addition of diene *via* syringe pump (for addition rates see below). The reaction was further stirred at r.t. until completion, checked by TLC. Then 2 mL sat. $NaHCO_3$ and 2 mL DCM or DCM/EtOAc (3:1) were added. The phases were separated and the aqueous phase was extracted with DCM or DCM/EtOAc (3:1; 3 x 4 mL). The combined organic phases were dried over $MgSO_4$, filtered and the solvent was removed

under reduced pressure. The residue was adsorbed on Celite® and purified by flash column chromatography to yield the desired product.

Notes for GP5 and GP6:

- Excess *p*-benzoquinone can sublime out of the mixture while removing traces of solvent under reduced pressure on the rotary evaporator. On large scales, it is advised to sublime excessive *p*-benzoquinone out of the crude mixture in a separate step under reduced pressure at slightly elevated temperature (we used about 1 Torr and 60 °C; a heating gun was used very carefully in this process to keep the glass surface of the flask hot).
- Adsorption of the crude mixtures on silica gel at elevated temperature can cause isomerization of the product and therefore reduce the yield of 5,8-dihydro-1,4-naphthoquinones **4**. Therefore, adsorption on Celite® was used.
- TfOH can be added either by a micro pipette or from a freshly prepared stock solution.
- In some cases, complete consumption of diene is faster than the transformation of the Diels-Alder adduct to its corresponding 5,8-dihydro-1,4-naphthoquinone and additional stirring was necessary. This was performed at r.t. The addition of another 2.5 mol% TfOH can also be used to accelerate this reaction.
- It is advised to run quick analyses to determine the complete reaction to 5,8-dihydro-1,4-naphthoquinone or to run the reaction on a small scale first to determine the reaction time. In our cases, when using TLC with toluene as mobile phase, the Diels-Alder adducts usually run below their corresponding 5,8-dihydro-1,4-naphthoquinones.
- If the corresponding 1,4-naphthoquinone is the desired product, complete conversion of the Diels-Alder adduct is not necessary. After removal of solvent and the majority of *p*-benzoquinone, the mixture can be used for the dehydrogenation reaction.

General Procedure 7 (GP7) for the dehydrogenation with MnO_2

The respective 5,8-dihydro-1,4-naphthoquinone (1.0 equiv) and activated MnO_2 (5 to 12 equiv) were suspended in toluene *or* $CHCl_3$ and heated in an oil bath until completion of the reaction, which was checked by TLC. After complete reaction, the suspension was cooled to r.t.. Celite® was added, the solvent was removed under reduced pressure and the residue was purified by flash column chromatography to yield the desired product.

4. Reaction of (1*E*,3*E*)-1,4-diphenyl-1,3-butadiene 1a and *p*-benzoquinone at 10 mmol scale

In a 50 mL pear shaped flask under N_2 atmosphere, 2090 mg (1*E*,3*E*)-1,4-diphenyl-1,3-butadiene **1a** (10.1 mmol, 1.0 equiv.) was dissolved in 50 mL anhydrous DCM and taken up into a 50 mL syringe. In a second Schlenk-flask under N_2 atmosphere, 4385 mg *p*-benzoquinone **2a** (40.6 mmol, 4.0 equiv) was dissolved in 50 mL anhydrous DCM and cooled with an ice-water bath to 0 °C. Then 22.2 µL TfOH (38.0 mg, 0.25 mmol, 2.5 mol%) was added with a micropipette to the *p*-benzoquinone solution, followed by the dropwise addition of diene solution in 15 min *via* syringe pump (200 mL/h). After complete addition, another 5 mL anhydrous DCM was taken up in the syringe and added to the reaction flask. The reaction was further stirred at rt. for 45 min. Then 50 mL sat. $NaHCO_3$ was carefully added, followed by 10 mL EtOAc to dissolve the black precipitate. The phases were separated and the aqueous phase was extracted with DCM/EtOAc (3:1; 3 x 50 mL). The combined organic phases were dried over $MgSO_4$, filtered and the solvent was removed under reduced pressure. Excess *p*-benzoquinone **2a** was removed from the resulting mixture by a vacuum flask-to-flask sublimation set-up. The flask containing the crude material was connected to the receiving flask (a two-neck flask with a gas inlet or a Schlenk

flask) with a bent glass adapter (containing two inner joints). A vacuum line was connected to the receiving flask, and the flask was cooled to at least – 20 °C using a liquid-nitrogen ethanol cooling bath. The pressure was carefully reduced to about 1 Torr and the flask containing the crude material was heated to 60 °C with a water bath (Note, a heating gun was carefully used to keep the glass in front of the receiving flask warm and to transfer *p*-benzoquinone completely to the receiving flask). After about one hour the sublimation was stopped and 1158 mg *p*-benzoquinone was recovered. The remaining crude solid was taken up in toluene and purified by flash column chromatography (220 g SiO_2, toluene) to yield 2393 mg (7.66 mmol, 76%) of a yellow amorphous solid.

5. Optimization of Catalyzed Diels-Alder Reaction

Table S1 Evaluation of different Lewis and Brønsted Acids as Catalysts[a]

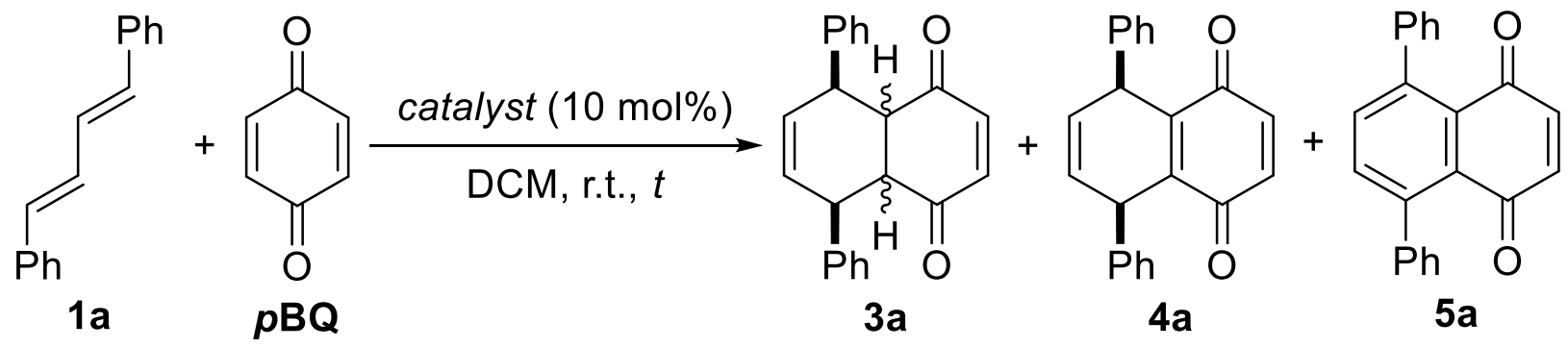

entry	*catalyst*	*t*/h	Conversion[b]	**3a**[b]/%	**4a**[b]/%	**5a**[b]/%
1[c]	none	3	100	n.d.	47	30
2	$BF_3 \cdot OEt_2$	24	100	23	32	n.d.
3	$AlCl_3$	24	90	66	14	n.d.
4	$Cu(OTf)_2$	24	66	35	19	n.d.
5	H_2SO_4	24	65	28	31	n.d.
6	MsOH	4	100	n.d.	75	n.d.
7	TfOH	0.25	100	n.d.	53	n.d.

[a] Reactions performed according to GP5 on a 0.2 mmol scale without purification by flash column chromatography. [b] Yield determined by ^{1}H-NMR with dibenzyl ether (0.1 mmol) as internal standard. [c] Acetic acid as solvent.

Table S2 Optimization of Reaction with Methanesulfonic acid as Catalyst[a]

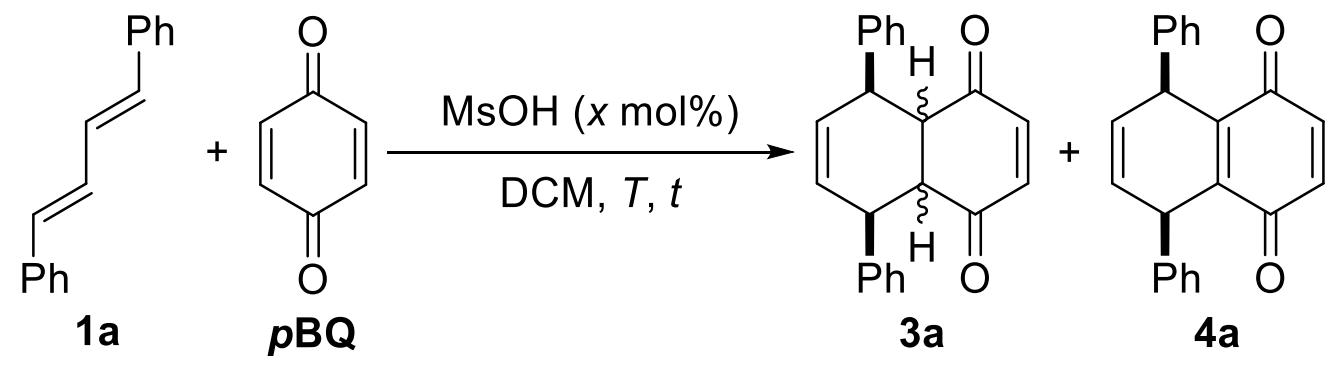

entry	**1a**:***p*BQ**	*x*/mol%	*c*/mol L^{-1}	*T*/°C	*t*/min	**3a**[b]/%	**4a**[b]/%
1	1:2	10	0.1	rt	240	n.d.	75
2	1:2	10	0.5	rt	100	n.d.	73
3	1:3	10	0.5	rt	90	28	51

4	1:4	10	0.5	rt	90	34	45
5	1:3	10	0.5	rt	150	6	72
6	1:2	10	0.5	50	60	n.d.	72
7	1:2	10	0.5	80	45	n.d.	70
8	1:2	20	0.1	rt	90	2	75 (73%[d])

[a] Reactions performed according to GP5 on a 0.2 mmol scale without purification by flash column chromatography. [b] Yield determined by ^{1}H-NMR with dibenzyl ether (0.1 mmol) as internal standard. [d] Yield of isolated product.

Table S3 Optimization of Reaction with Triflic acid as Catalyst

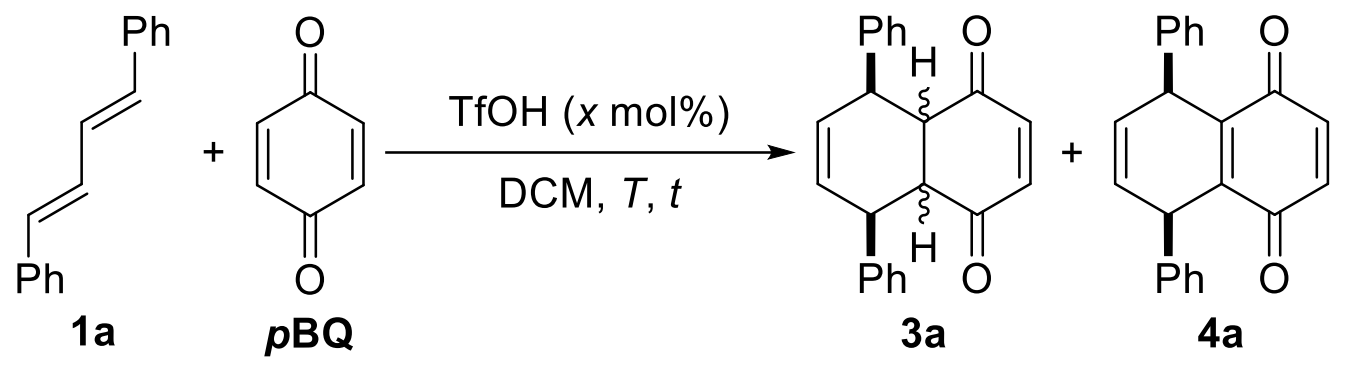

entry	**1a**:***p*****BQ**	x/mol%	*c*/mol L^{-1}	*T*/°C	*t*/min	**3a**[a]/%	**4a**[a]/%
1[b]	1:2	10	0.1	rt	15	n.d.	53
2[b]	1:2	10	0,1	0	15	n.d.	47
3[b]	1:4	10	0,1	0	15	7	47
4[b]	1:4	2.5	0,1	0	15	50	17
5[b]	1:4	2.5	0.5	0	15	18	35
6[c]	1:4	2.5	0.1	0	30[d]+20[e]	52	27
7[c,f]	1:4	5.7	0.1	0	30[d]	45	38
8[c,f]	1:4	2.7	0.1	0	30[d]	33	49
9[c]	1:4	2.5	0.1	0	15[d]+45[g]	n.d.	77[h]

[a] Yield determined by ^{1}H-NMR with dibenzyl ether (0.1 mmol) as internal standard. [b] Reaction performed according to GP5 on a 0.2 mmol scale without purification by flash column chromatography. [c] Reaction performed according to GP6 on a 0.2 mmol scale; [d] Time of diene addition. [e] Time of additional stirring at 0 °C. [f] Freshly distilled triflic acid, added from a freshly prepared stock solution. [g] Time of additional stirring at r.t. [h] Yield of isolated product.

6. Proposed Mechanism and Reaction Sequence

Scheme S1. Proposed Mechanism

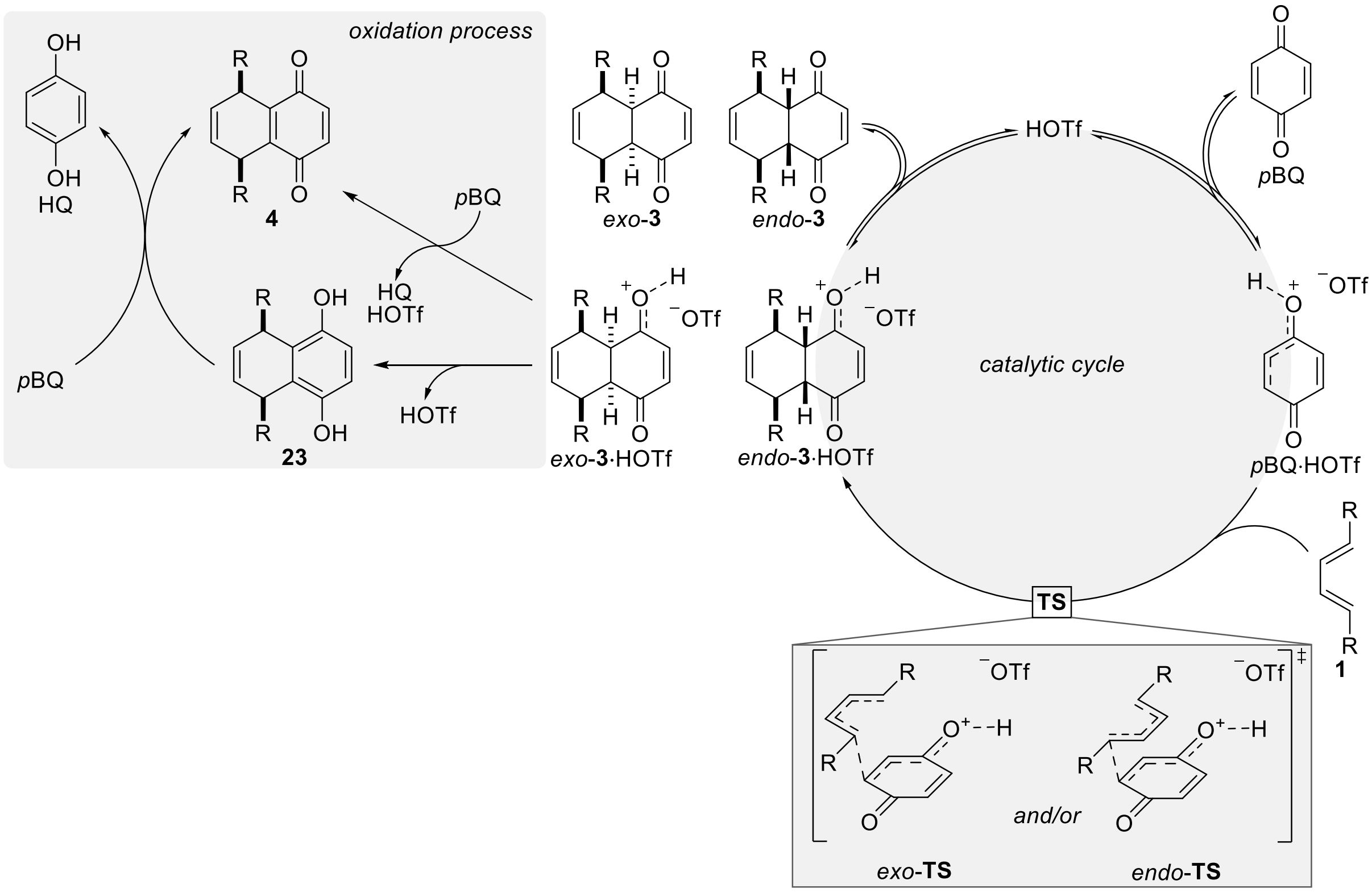

For the mechanism we propose the activation of one of the double bonds of *p*BQ through protonation of one of the carbonyl functions, followed by the C-C bond formation through an *endo* or *exo* transition state (TS). Computations on *α*,*β*-unsaturated enones show that an asynchronous TS can't be ruled out.[1] Since we observed **3a** in crude mixtures of ^{1}H-NMR samples of the reaction of *p*BQ and **1a**, it seems feasible an equilibrium between **3**·HOTf and **3** exists. The formation of **4** happens through an oxidation process, in which several pathways are possible. One might be the acid catalyzed isomerization to **23**, which afterwards is oxidized to **4** with *p*BQ. Another possible path is the acid-mediated dehydrogenation from **3**·HOTf to **4** in a single step without the isomerization.

Scheme S2. Reaction sequence for first and second Diels-Alder reaction.

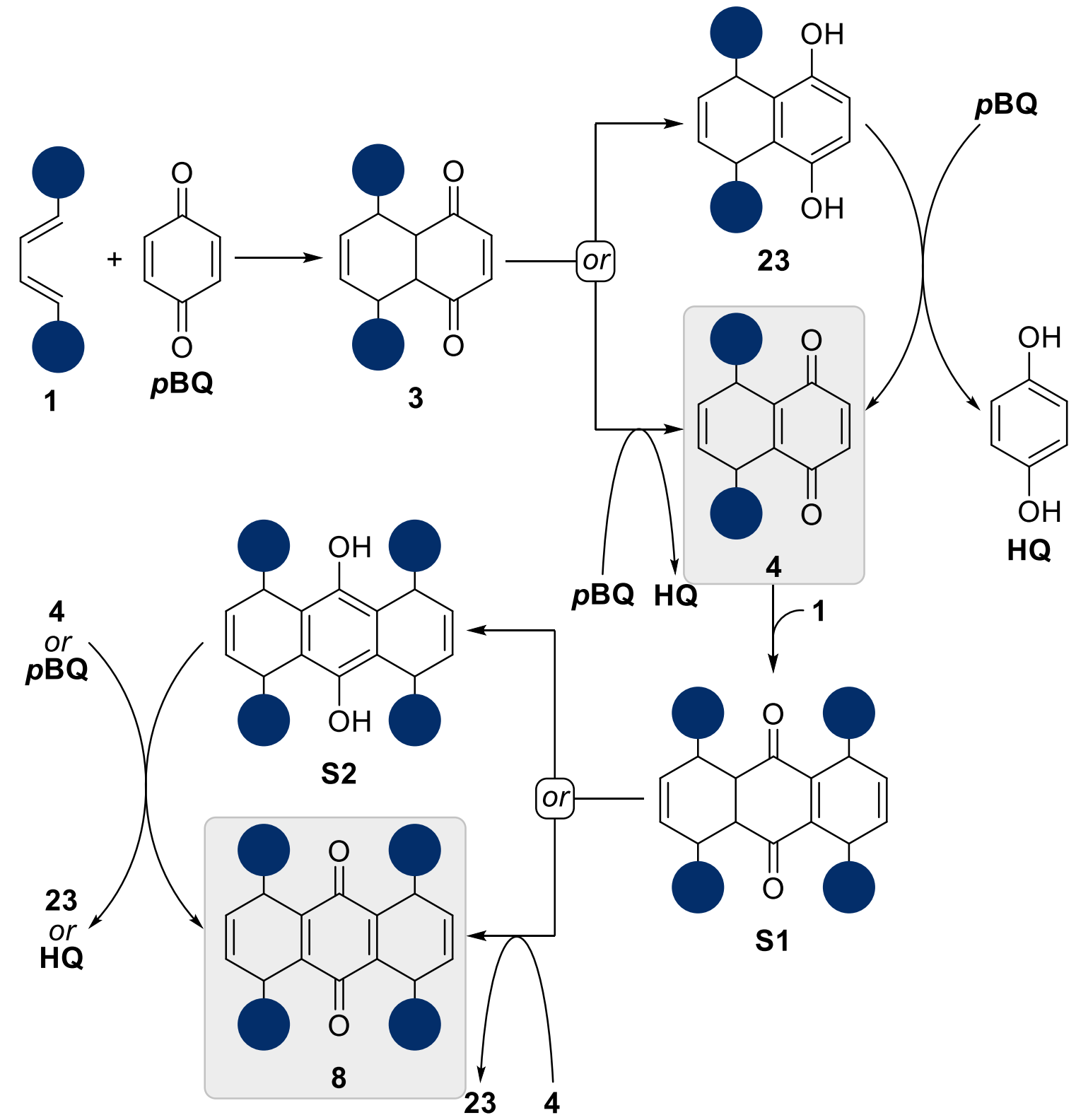

7. Synthetic Procedures and Analytical Data

7.1 Overview of Substrate Synthesis

Scheme S3. Synthesis of 1,4-diphenyl-1,3-butadiene 1a.

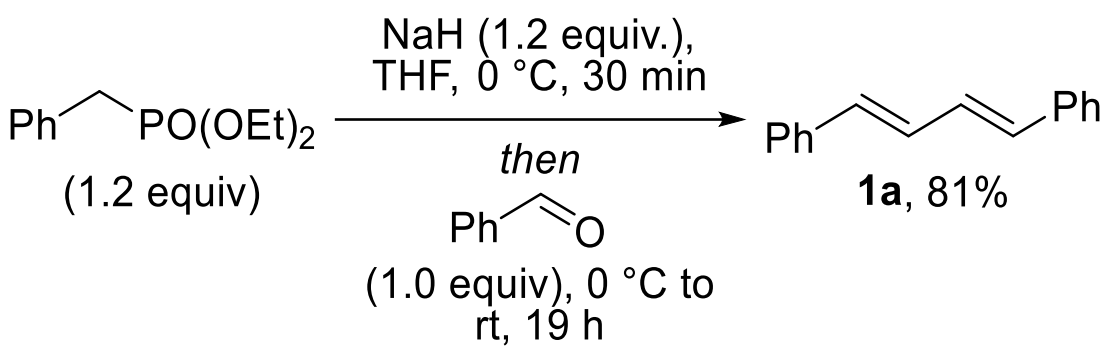

Scheme S4. Synthesis of 4-substituted 1-phenyl-1,3-butadienes 1b to 1y.

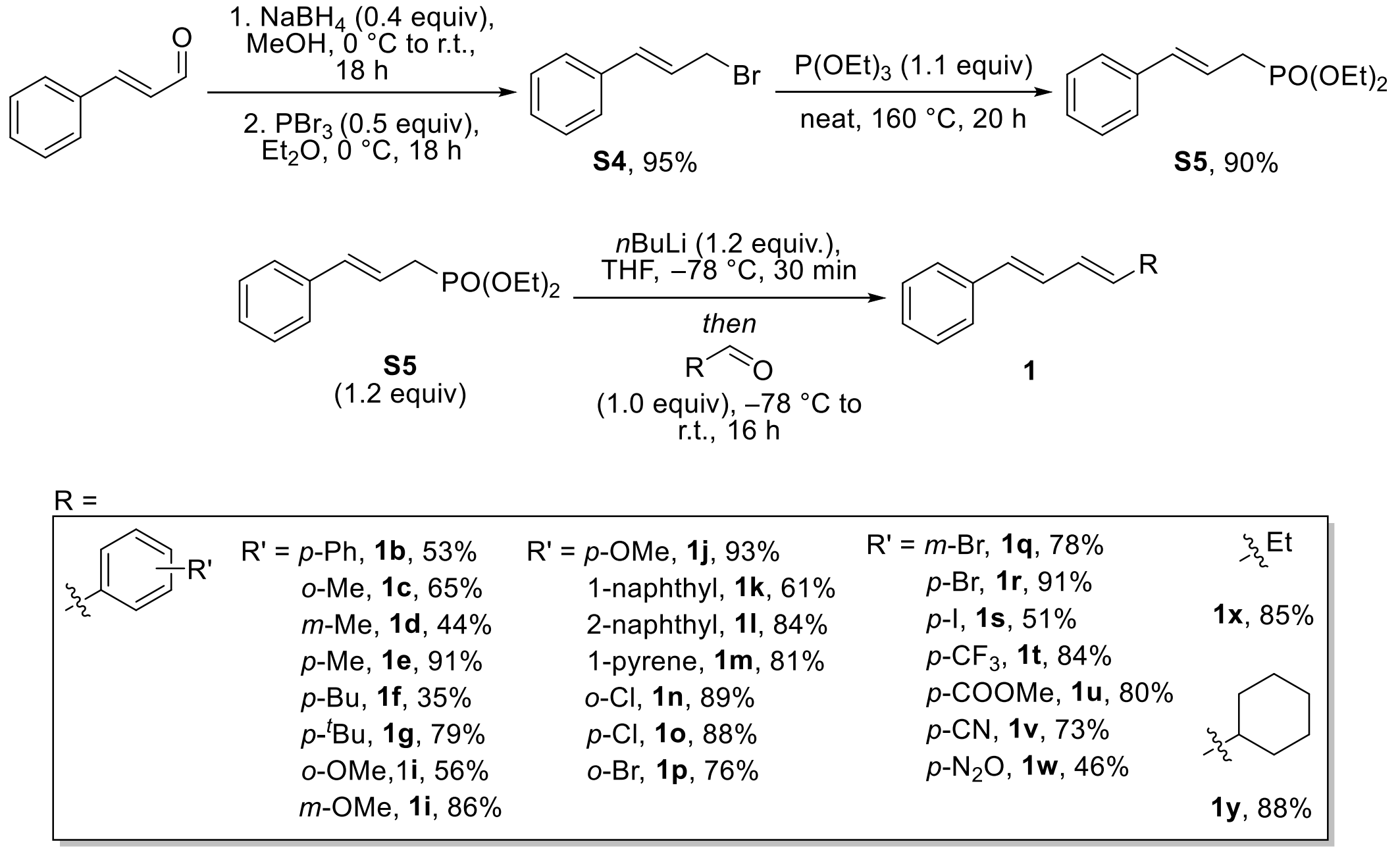

Scheme S5. Synthesis of Symmetric 1,4-Diaryl-1,3-butadienes 1z and 1aa.

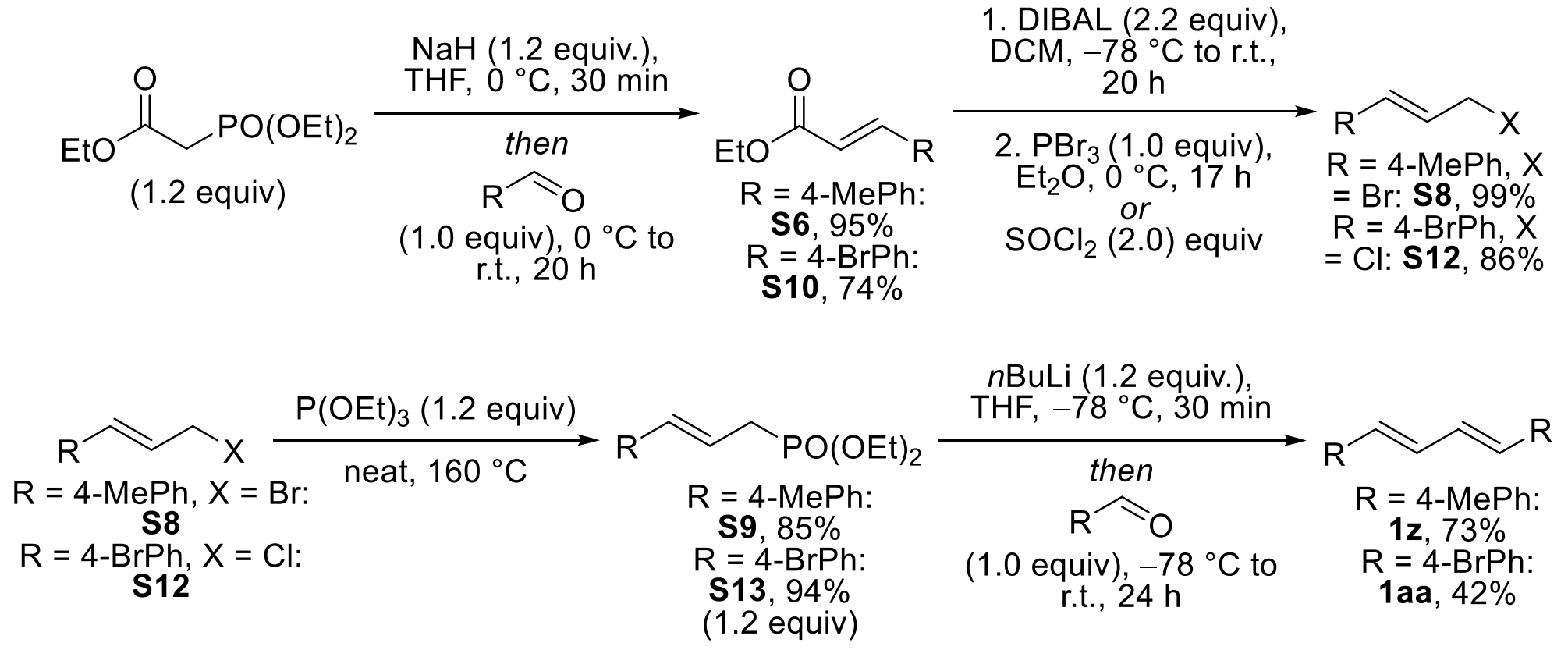

Scheme S6. Synthesis of ethyl 1,3-dienoates 1ab to 1ad.

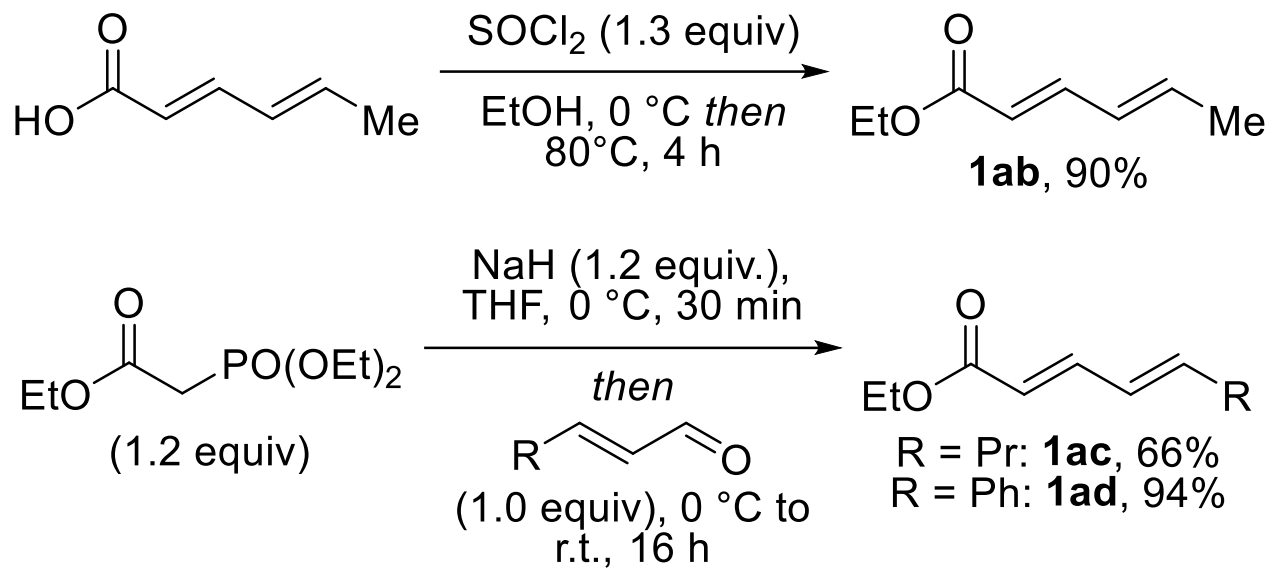

Scheme S7. Synthesis of 1-aryl-1,3-butadienes 1ae and 1af.

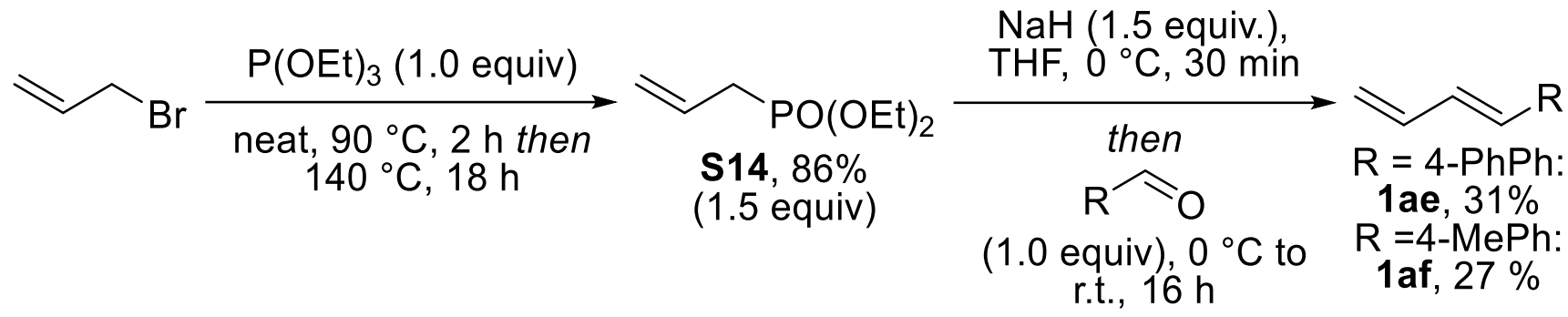

Scheme S8. Synthesis of 2-substituted *p*-benzoquinones 2.

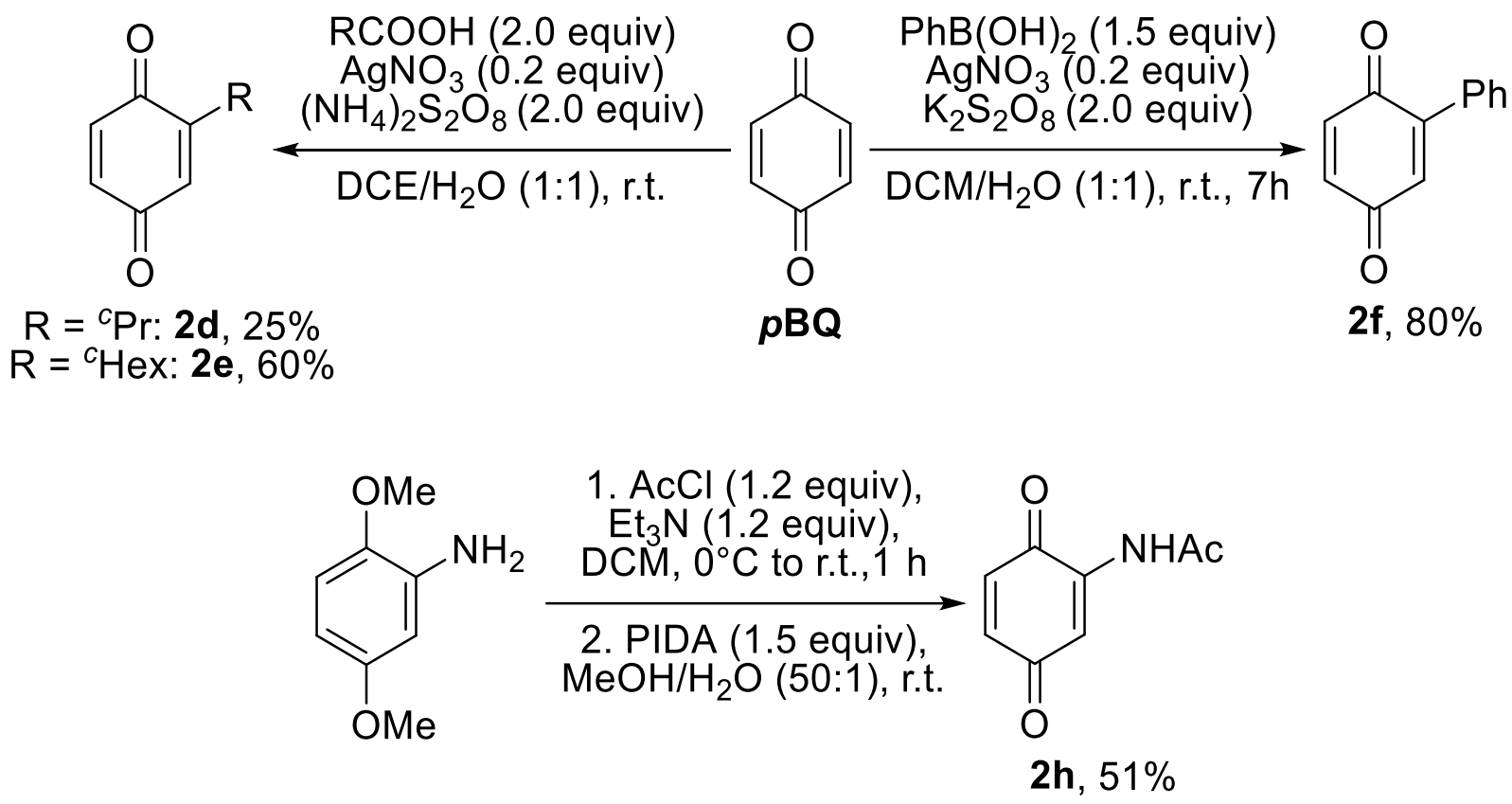

Scheme S9. Miscellaneous.

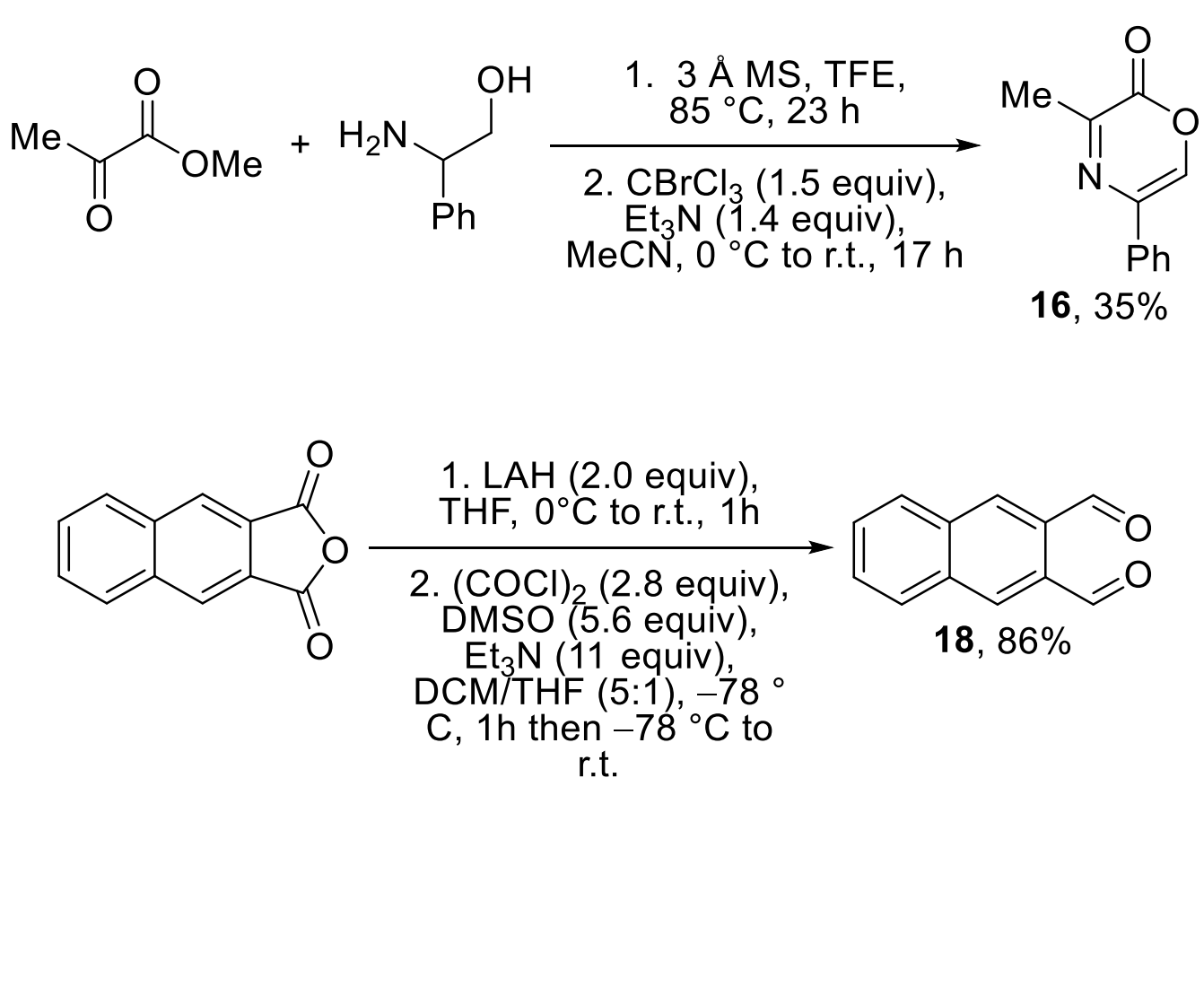

7.2 Synthesis and Analytical Data of Dienes

7.2.1 (1*E*,3*E*)-1,4-Diphenyl-1,3-butadiene (1a)

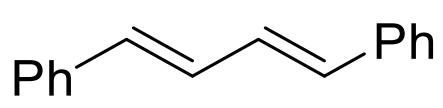

Obtained as a white amorphous solid according to GP1:

- 1.05 g, 34.3 mmol, 1.0 equiv *trans*-cinnamic aldehyde
- 9.30 g, 40.7 mmol, 1.2 equiv diethyl benzylphosphonate
- 1.09 g, 40.7 mmol, 1.2 equiv NaH (90% dry)
- 340 mL anhydrous THF as solvent
- Purified by flash column chromatography (SiO_2, 10% Et_2O in *n*-pentane)

Yield: 5.72 mg (27.7 mmol, 80%).

^{1}H NMR (400 MHz, CD_2Cl_2): δ 7.50 – 7.43 (m, 4H), 7.38 – 7.31 (m, 4H), 7.27 – 7.22 (m, 2H), 7.04 – 6.95 (m, 2H), 6.75 – 6.65 (m, 2H).

^{13}C NMR (101 MHz, CD_2Cl_2): δ 137.8, 133.2, 129.6, 129.1, 129.0, 126.8.

IR (ATR, cm^{-1}): 1443 (m), 1073 (m), 982 (s), 912 (m), 736 (s), 687 (s), 492 (m).

HRMS (APCI): calc. for $C_{16}H_{15}^+$ $[M+H]^+$: 207.1168; found: 207.1170.

Spectral data are in accordance with those reported in the literature.[2]

7.2.2 1-Substituted (1*E*,3*E*)-4-phenyl-1,3-butadienes (1b to 1y)

7.2.2.1 Synthesis of P[(2E)-3-phenyl-2-propen-1-yl]phosphonate

Step 1: *trans*-cinnamyl alcohol (**S3**)

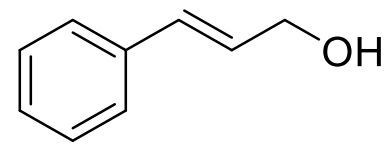

In a round-bottom flask, 26.67 g freshly distilled *trans*-cinnamaldehyde (201.8 mol, 1.0 equiv) was dissolved in 200 mL MeOH and cooled to 0 °C. 3.15 g $NaBH_4$ (83.3 mmol, 0.41 equiv) was added in portions and the resulting suspension was warmed to r.t. and stirred until completion. Then 50 mL 1 M HCl was added carefully, followed by dilution with water and DCM (approx. 200 mL each). The mixture was transferred to a separatory funnel, the phases were separated and the aqueous phase was extracted with DCM (2 x 200 mL). The combined organic phases were washed with brine, dried over $MgSO_4$, filtered and the solvent removed under reduced pressure to yield 26.97 g of a white amorphous solid. The product was used in the next step without further purification.

Yield: 26.97 g (201.0 mmol, 99%).

^{1}H NMR (400 MHz, $CDCl_3$): δ 7.43 – 7.37 (m, 2H), 7.36 – 7.29 (m, 2H), 7.28 – 7.21 (m, 1H), 6.62 (dt, *J* = 15.9, 1.7 Hz, 1H), 6.37 (dt, *J* = 15.9, 5.7 Hz, 1H), 4.33 (dd, *J* = 5.7, 1.6 Hz, 1H), 1.67 – 1.57 (m, 1H).

^{13}C NMR (101 MHz, $CDCl_3$): δ 136.8, 131.3, 128.7, 128.6, 127.8, 126.6, 63.9.

Spectral data are in accordance with those reported in the literature.[3]

Step 2: *trans*-cinnamyl bromide (**S4**)

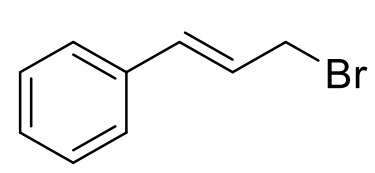

Under an inert gas atmosphere in a round-bottom flask 26.97 g *trans*-cinnamyl alcohol (201.0 mol, 1.0 equiv) was dissolved in 290 mL Et_2O, then 10 mL PBr_3 (28.50 g, 105.3 mmol, 0.52 equiv) was added dropwise and the resulting mixture was stirred overnight. The mixture was cooled to 0 °C, 100 mL sat. $NaHCO_3$ was added carefully and then transferred into a separatory funnel. The phases were separated and the aqueous phase was extracted

with Et_2O (3 x 200 mL). The combined organic phases were dried over $MgSO_4$, filtered and the solvent was removed under reduced pressure to yield 37.93 g of a white amorphous solid. The product was used in the next step without further purification.

Yield: 37.93 g (192.5 mmol, 96%).

^{1}H NMR (400 MHz, $CDCl_3$): δ 7.42 – 7.37 (m, 2H), 7.33 (ddd, *J* = 8.1, 6.8, 1.7 Hz, 2H), 7.30 – 7.24 (m, 1H), 6.65 (d, *J* = 15.6 Hz, 1H), 6.40 (dt, *J* = 15.5, 7.8 Hz, 1H), 4.17 (dd, *J* = 7.8, 1.0 Hz, 2H).

^{13}C NMR (101 MHz, $CDCl_3$): δ 136.0, 134.7, 128.8, 128.5, 126.9, 125.4, 33.6.

Spectral data are in accordance with those reported in the literature.[4]

Step 3: *P*[(2*E*)-3-phenyl-2-propen-1-yl]phosphonate (**S5**)

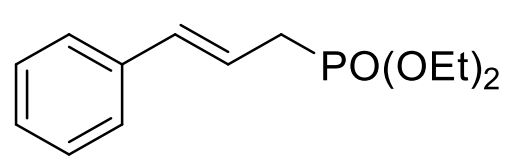

Obtained as a colorless liquid according to GP2:

- 37.93 g, 192.5 mmol, 1.0 equiv *trans*-cinnamyl bromide
- 35.33 g, 212.6 mmol, 1.1 equiv triethyl phosphite
- Purified by distillation (0.92 Torr, 150 to 155 °C)

Yield: 44.20 g (173.8 mmol, 90%).

^{1}H NMR (400 MHz, $CDCl_3$): δ 7.38 – 7.27 (m, 4H), 7.25 – 7.20 (m, 1H), 6.52 (ddt, *J* = 15.9, 5.3, 1.5 Hz, 1H), 6.16 (dq, *J* = 15.7, 7.4 Hz, 1H), 4.19 – 4.05 (m, 4H), 2.76 (ddd, *J* = 22.1, 7.6, 1.4 Hz, 2H), 1.32 (t, *J* = 7.0 Hz, 6H).

^{13}C NMR (101 MHz, $CDCl_3$): δ 136.9 (d, *J* = 3.6 Hz), 134.8 (d, *J* = 15.0 Hz), 128.7, 127.7, 126.3 (d, *J* = 2.3 Hz), 118.9 (d, *J* = 11.8 Hz), 62.2 (d, *J* = 6.8 Hz), 31.2 (d, *J* = 139.9 Hz), 16.6 (d, *J* = 5.9 Hz).

^{31}P NMR (162 MHz, $CDCl_3$): δ 27.8.

IR (ATR, cm^{-1}): 1247 (m), 1019 (s), 956 (s), 877 (m), 782 (m), 745 (m), 693 (m), 543 (m), 483 (m).

HRMS (ESI): calc. for $C_{13}H_{19}NaO_3P^+$ $[M+Na]^+$: 277.0964; found: 277.0965.

Spectral data are in accordance with those reported in the literature.[5]

*7.2.2.2 4-[(1E,3E)-4-phenyl-1,3-butadien-1-yl]-1,1'-biphenyl (**1b**)*

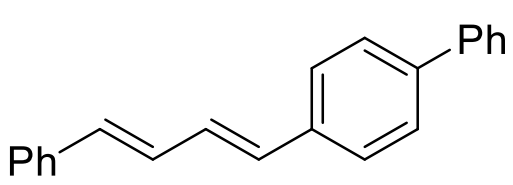

Obtained as a white amorphous solid according to GP3:

- 637 mg, 3.50 mmol, 1.0 equiv biphenyl-4-carbaldehyde
- 1058 mg, 4.16 mmol, 1.2 equiv diethyl *P*[(2*E*)-3-phenyl-2-propen-1-yl]phosphonate
- 2.6 mL, 4.16 mmol, 1.2 equiv *n*-BuLi (1.6 M in *n*-hexane)
- 30 mL anhydrous THF as solvent
- Purified by flash column chromatography (SiO_2, 10% Et_2O in *n*-pentane)

Yield: 522 mg (1.85 mmol, 53%).

^{1}H NMR (400 MHz, CD_2Cl_2): δ 7.69 – 7.58 (m, 4H), 7.58 – 7.52 (m, 2H), 7.50 – 7.41 (m, 4H), 7.40 – 7.31 (m, 3H), 7.29 – 7.19 (m, 1H), 7.09 – 6.97 (m, 2H), 6.80 – 6.66 (m, 2H).

^{13}C NMR (101 MHz, CD_2Cl_2): δ 140.9, 140.5, 137.8, 136.9, 133.3, 132.7, 129.8, 129.6, 129.2, 129.1, 128.0, 127.8, 127.6, 127.2, 127.2, 126.8.

IR (ATR, cm^{-1}): 985 (m), 844 (m), 763 (m), 744 (s), 688 (s), 476 (m).

HRMS (APCI): calc. for $C_{22}H_{19}^+$ $[M+H]^+$: 283.1483; found: 283.1484.

*7.2.2.3 1-Methyl-2-[(1E,3E)-4-phenyl-1,3-butadien-1-yl]benzene (**1c**)*

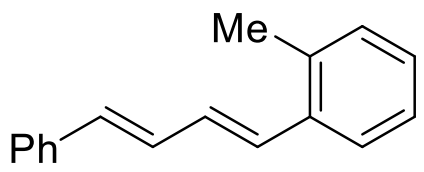

Obtained as a white amorphous solid according to GP3:

- 841 mg, 7.00 mmol, 1.0 equiv 2-methylbenzaldehyde
- 2161 mg, 8.50 mmol, 1.2 equiv diethyl *P*[(2*E*)-3-phenyl-2-propen-1-yl]phosphonate
- 3.5 mL, 8.33 mmol, 1.2 equiv *n*-BuLi (2.38 M in *n*-hexane)
- 60 mL anhydrous THF as solvent
- Purified by flash column chromatography (SiO_2, *n*-pentane)

Yield: 1006 mg (4.57 mmol, 65%).

^{1}H NMR (400 MHz, CD_2Cl_2): δ 7.60 – 7.54 (m, Hz, 1H), 7.50 – 7.43 (m, 2H), 7.34 (dd, *J* = 8.4, 6.9 Hz, 2H), 7.28 – 7.10 (m, 4H), 7.04 (ddd, *J* = 15.4, 7.8, 2.1 Hz, 1H), 7.00 – 6.85 (m, 2H), 6.70 (d, *J* = 15.4 Hz, 1H), 2.40 (s, 3H).

^{13}C NMR (101 MHz, CD_2Cl_2): δ 137.8, 136.5, 136.1, 133.0, 130.9, 130.8, 130.6, 130.0, 129.1, 127.9, 127.9, 126.7, 126.5, 125.3, 20.0.

IR (ATR, cm^{-1}): 1480 (m), 1444 (m), 989 (s), 746 (s), 691 (s), 502 (m), 452 (m).

HRMS (APCI): calc. for $C_{17}H_{17}^+$ [M+H]$^+$: 221.1325; found: 221.1325.

Spectral data are in accordance with those reported in the literature.[2]

*7.2.2.4 1-Methyl-3-[(1E,3E)-4-phenyl-1,3-butadien-1-yl]benzene (**1d**)*

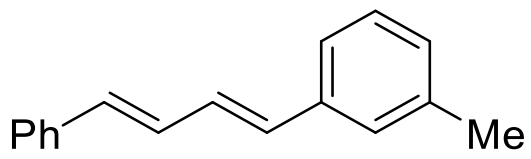

Obtained as a white amorphous solid according to GP3:

- 420 mg, 3.50 mmol, 1.0 equiv 3-methylbenzaldehyde
- 1061 mg, 4.17 mmol, 1.2 equiv diethyl P[(2*E*)-3-phenyl-2-propen-1-yl]phosphonate
- 2.6 mL, 4.16 mmol, 1.2 equiv *n*-BuLi (1.6 M in *n*-hexane)
- 30 mL anhydrous THF as solvent
- Purified by flash column chromatography (SiO_2, *n*-pentane)

Yield: 337 mg (1.53 mmol, 44%).

^{1}H NMR (400 MHz, CD_2Cl_2): δ 7.49 – 7.43 (m, 2H), 7.38 – 7.29 (m, 2H), 7.31 – 7.18 (m, 4H), 7.09 – 7.04 (m, 1H), 7.04 – 6.92 (m, 2H), 6.75 – 6.60 (m, 2H), 2.36 (s, 3H).

^{13}C NMR (101 MHz, CD_2Cl_2): δ 138.8, 137.8, 137.7, 133.4, 133.0, 129.7, 129.4, 129.1, 128.9, 128.8, 127.9, 127.5, 126.7, 123.9, 21.5.

IR (ATR, cm^{-1}): 989 (s), 905 (m), 830 (m), 779 (s), 747 (s), 687 (s), 635 (s), 504 (m), 453 (m), 435 (m).

HRMS (APCI): calc. for $C_{17}H_{17}^+$ [M+H]$^+$: 221.1325; found: 221.1327.

Spectral data are in accordance with those reported in the literature.[2]

*7.2.2.5 1-Methyl-4-[(1E,3E)-4-phenyl-1,3-butadien-1-yl]benzene (**1e**)*

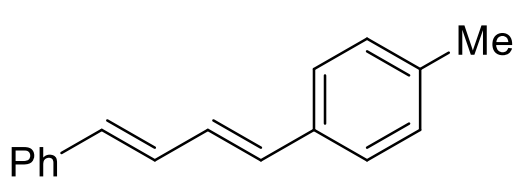

Obtained as a white amorphous solid according to GP3:

- 841 mg, 7.00 mmol, 1.0 equiv 4-methylbenzaldehyde
- 2139 mg, 8.41 mmol, 1.2 equiv diethyl *P*[(2*E*)-3-phenyl-2-propen-1-yl]phosphonate
- 3.3 mL, 8.25 mmol, 1.2 equiv *n*-BuLi (2.5 M in *n*-hexane)
- 60 mL anhydrous THF as solvent
- Purified by flash column chromatography (SiO_2, 20% Et_2O in *n*-pentane).

Yield: 1410 mg (6.40 mmol, 91%).

^{1}**H NMR** (400 MHz, CD_2Cl_2): δ 7.48 – 7.42 (m, 2H), 7.37 – 7.30 (m, 4H), 7.27 – 7.18 (m, 1H), 7.18 – 7.13 (m, 2H), 7.04 – 6.88 (m, 2H), 6.71 – 6.62 (m, 2H), 2.34 (s, 3H).

^{13}**C NMR** (101 MHz, CD_2Cl_2): δ 138.1, 137.9, 135.0, 133.2, 132.6, 129.8, 129.8, 129.0, 128.6, 127.9, 126.7, 21.4.

IR (ATR, cm^{-1}): 1445 (m), 992 (s), 912 (m), 842 (m), 803 (m), 745 (s), 689 (s), 621 (w), 498 (s).

HRMS (APCI): calc. for $C_{17}H_{17}^{+}$ $[M+H]^{+}$: 221.1325; found: 221.1328.

Spectral data are in accordance with those reported in the literature.[2]

*7.2.2.6 1-Butyl-4-[(1E,3E)-4-phenyl-1,3-butadien-1-yl]benzene (**1f**)*

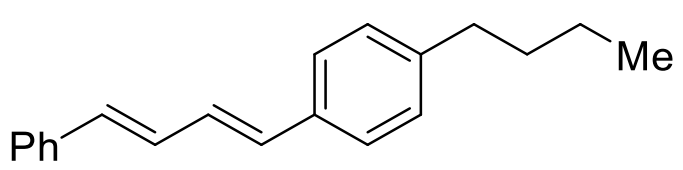

Obtained as a white amorphous solid according to GP3:

- 557 mg, 3.43 mmol, 1.0 equiv 4-butylbenzaldehyde
- 1056 mg, 4.15 mmol, 1.2 equiv diethyl *P*[(2*E*)-3-phenyl-2-propen-1-yl]phosphonate
- 2.6 mL, 4.16 mmol, 1.2 equiv *n*-BuLi (1.6 M in *n*-hexane)
- 30 mL anhydrous THF as solvent
- Purified by flash column chromatography (SiO_2, *n*-pentane)

Yield: 319 mg (1.21 mmol, 35%).

^{1}**H NMR** (400 MHz, CD_2Cl_2): δ 7.48 – 7.42 (m, 2H), 7.41 – 7.29 (m, 4H), 7.27 – 7.18 (m, 1H), 7.20 – 7.12 (m, 2H), 7.04 – 6.89 (m, 2H), 6.72 – 6.62 (m, 2H), 2.65 – 2.56 (m, 2H), 1.65 – 1.53 (m, 2H), 1.36 (dq, J = 14.6, 7.3 Hz, 2H), 0.93 (t, J = 7.3 Hz, 3H).

^{13}**C NMR** (101 MHz, CD_2Cl_2): δ 143.2, 137.9, 135.2, 133.3, 132.6, 129.8, 129.2, 129.0, 128.7, 127.8, 126.7, 35.8, 34.0, 22.8, 14.1.

IR (ATR, cm^{-1}): 2953 (m), 2921 (m), 2854 (m), 988 (s), 847 (m), 811 (m), 747 (s), 689 (s), 535 (m), 504 (m).

HRMS (APCI): calc. for $C_{20}H_{23}^{+}$ $[M+H]^{+}$: 263.1795; found: 263.1799.

Spectral data are in accordance with those reported in the literature.[6]

*7.2.2.7 1-tert-Butyl-4-[(1E,3E)-4-phenyl-1,3-butadien-1-yl]benzene (**1g**)*

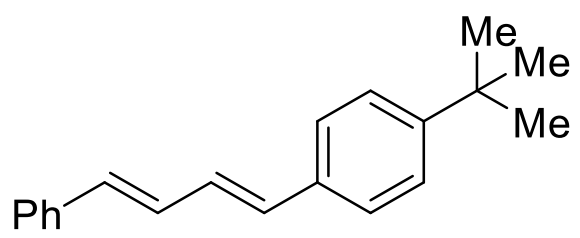

Obtained as a white amorphous solid according to GP3:

- 567 mg, 3.49 mmol 4-*tert*-butylbenzaldehyde;
- 1060 mg, 4.17 mmol diethyl *P*[(2*E*)-3-phenyl-2-propen-1-yl]phosphonate;
- 2.6 mL, 4.16 mmol *n*-BuLi (1.6 M in *n*-hexane);
- 30 mL anhydrous THF as solvent.
- Purified by flash column chromatography (SiO_2, *n*-pentane).

Yield: 725 mg (2.76 mmol, 79%).

^{1}**H NMR** (400 MHz, CD_2Cl_2): δ 7.49 – 7.42 (m, 2H), 7.43 – 7.29 (m, 6H), 7.26 – 7.20 (m, 1H), 7.04 – 6.89 (m, 2H), 6.74 – 6.62 (m, 2H), 1.32 (s, 9H).

^{13}**C NMR** (101 MHz, CD_2Cl_2): δ 151.3, 137.9, 135.0, 133.1, 132.6, 129.8, 129.0, 128.8, 127.9, 126.7, 126.5, 126.0, 34.9, 31.4.

IR (ATR, cm^{-1}): 3014 (m), 983 (s), 965 (m), 850 (m), 839 (m), 810 (m), 748 (s), 731 (m), 689 (m), 555 (m), 509 (m).

HRMS (APCI): calc. for $C_{20}H_{23}^{+}$ $[M+H]^{+}$: 263.1795; found: 263.1798.

Spectral data are in accordance with those reported in the literature.[6]

*7.2.2.8 1-Methoxy-2-[4-phenyl-1,3-butadien-1-yl]benzene (**1h**)*

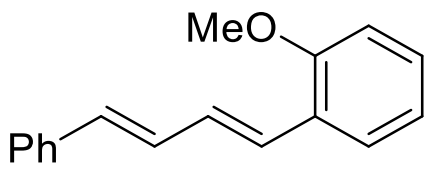

Obtained as a white amorphous solid according to GP3:

- 480 mg, 3.50 mmol, 1.0 equiv 2-methoxybenzaldehyde;
- 1084 mg, 4.26 mmol, 1.2 equiv diethyl *P*[(2*E*)-3-phenyl-2-propen-1-yl]phosphonate;
- 2.6 mL, 4.16 mmol, 1.2 equiv *n*-BuLi (1.6 M in *n*-hexane);
- 30 mL anhydrous THF as solvent).
- Purified by flash column chromatography (SiO_2, 1% Et_2O in *n*-pentane).

Yield: 466 mg (15:1.5:1 d.r., 1.97 mmol, 56%).

^{1}H NMR (600 MHz, CD_2Cl_2): δ 7.53 (dd, *J* = 7.7, 1.7 Hz, 1H), 7.48 – 7.44 (m, 2H), 7.36 – 7.32 (m, 2H), 7.25 – 7.21 (m, 2H), 7.05 – 7.00 (m, 3H), 6.97 – 6.89 (m, 2H), 6.70 – 6.65 (m, 1H), 3.88 (s, 4H).

^{13}C NMR (151 MHz, CD_2Cl_2): δ 157.3, 138.0, 132.5, 130.5, 130.1, 129.1, 129.0, 128.1, 127.8, 126.8, 126.7, 121.1, 111.4, 55.9.

IR (ATR, cm^{-1}): 1484 (m), 1460 (m), 1436 (m), 1238 (s), 1099 (m), 1025 (m), 996 (s), 746 (s), 728 (s), 690 (s).

HRMS (APCI): calc. for $C_{17}H_{17}O^+$ [M+H]$^+$: 237.1274; found: 237.1273.

Spectral data are in accordance with those reported in the literature.[7]

*7.2.2.9 1-Methoxy-3-[(1E,3E)-4-phenyl-1,3-butadien-1-yl]benzene (**1i**)*

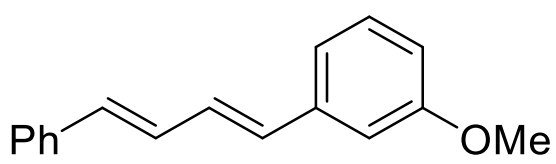

Obtained as a white amorphous solid according to GP3:

- 477 mg, 3.50 mmol, 1.0 equiv 3-methoxybenzaldehyde;
- 1082 mg, 4.25 mmol, 1.2 equiv diethyl *P*[(2*E*)-3-phenyl-2-propen-1-yl]phosphonate;
- 2.6 mL, 4.16 mmol, 1.2 equiv *n*-BuLi (1.6 M in *n*-hexane);
- 30 mL anhydrous THF as solvent).
- Purified by flash column chromatography (SiO_2, 10% Et_2O in *n*-pentane).

Yield: 713 mg (3.02 mmol, 86%).

^{1}H NMR (400 MHz, $CDCl_3$): δ 7.48 – 7.41 (m, 2H), 7.38 – 7.31 (m, 2H), 7.30 – 7.20 (m, 2H), 7.07 – 7.02 (m, 1H), 7.00 – 6.91 (m, 3H), 6.80 (ddd, *J* = 8.2, 2.6, 0.9 Hz, 1H), 6.74 – 6.60 (m, 2H), 3.84 (s, 3H).

^{13}C NMR (101 MHz, $CDCl_3$): δ 160.0, 139.0, 137.5, 133.2, 132.8, 129.8, 129.7, 129.3, 128.8, 127.8, 126.6, 119.3, 113.5, 111.7, 55.4.

IR (ATR, cm^{-1}): 1568 (m), 1449 (m), 1434 (m), 1289 (m), 1252 (m), 966 (s), 773 (m), 748 (s), 684 (s), 507 (m), 453 (m).

HRMS (APCI): calc. for $C_{17}H_{17}O^+$ [M+H]$^+$: 237.1274; found: 237.1274.

Spectral data are in accordance with those reported in the literature.[2]

*7.2.2.10 1-Methoxy-4-[(1E,3E)-4-phenyl-1,3-butadien-1-yl]benzene (**1j**)*

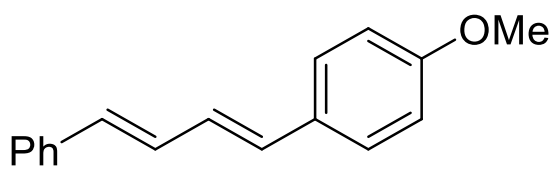

Obtained as a white amorphous solid according to GP3:

- 953 mg, 7.00 mmol, 1.0 equiv 4-methoxybenzaldehyde;
- 2145 mg, 8.43 mmol, 1.2 equiv diethyl *P*[(2*E*)-3-phenyl-2-propen-1-yl]phosphonate;
- 3.4 mL, 8.50 mmol, 1.2 equiv *n*-BuLi (2.5 M in *n*-hexane);

▪ 60 mL anhydrous THF as solvent.
▪ Purified by flash column chromatography (SiO_2, 10% Et_2O in *n*-pentane).

Yield: 1545 mg (6.54 mmol, 93%).

^{1}H NMR (400 MHz, CD_2Cl_2): δ 7.49 – 7.36 (m, 4H), 7.37 – 7.28 (m, 2H), 7.26 – 7.18 (m, 1H), 6.97 (dd, *J* = 15.2, 10.6 Hz, 1H), 6.91 – 6.81 (m, 3H), 6.64 (dd, *J* = 15.3, 2.8 Hz, 2H), 3.81 (s, 3H).

^{13}C NMR (101 MHz, CD_2Cl_2): δ159.9, 138.0, 132.8, 132.0, 130.5, 129.9, 129.0, 128.0, 127.7, 127.6, 126.6, 114.5, 55.7.

IR (ATR, cm^{-1}): 1595 (m), 1507 (m), 1248 (m), 1173 (m), 1028 (m), 990 (s), 839 (s), 807 (m), 746 (s), 687 (s), 506 (m).

HRMS (APCI): calc. for $C_{17}H_{17}O^+$ $[M+H]^+$: 237.1274; found: 237.1275.

Spectral data are in accordance with those reported in the literature.[2]

*7.2.2.11 1-[(1E,3E)-4-phenyl-1,3-butadien-1-yl]naphthalene (**1k**)*

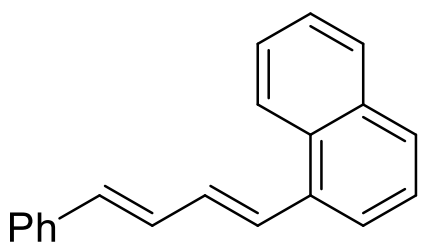

Obtained as a white amorphous solid according to GP3:

▪ 546 mg, 3.50 mmol, 1.0 equiv 1-naphthaldehyde
▪ 1040 mg, 4.09 mmol, 1.2 equiv diethyl *P*[(2*E*)-3-phenyl-2-propen-1-yl]phosphonate
▪ 1.6 mL, 4.00 mmol, 1.1 equiv *n*-BuLi (2.5 M in *n*-hexane)
▪ 30 mL anhydrous THF as solvent
▪ Purified by flash column chromatography (SiO_2, 50% DCM in *n*-hexane)

Yield: 543 mg (2.12 mmol, 61%).

^{1}H NMR (400 MHz, CD_2Cl_2): δ 8.22 (d, *J* = 9.7 Hz, 1H), 7.91 – 7.85 (m, 1H), 7.78 (dd, *J* = 13.0, 7.7 Hz, 2H), 7.60 – 7.45 (m, 6H), 7.37 (t, *J* = 7.6 Hz, 2H), 7.30 – 7.24 (m, 1H), 7.21 – 7.04 (m, 2H), 6.76 (d, *J* = 15.1 Hz, 1H).

^{13}C NMR (101 MHz, CD_2Cl_2): δ 137.8, 134.9, 134.3, 133.5, 132.3, 131.5, 129.9, 129.7, 129.1, 129.0, 128.4, 128.1, 126.8, 126.5, 126.2, 126.1, 123.9, 123.5.

IR (ATR, cm^{-1}): 988 (s), 794 (s), 773 (s), 753 (s), 744 (s), 688 (s), 557 (m), 5011 (m), 426 (m).

HRMS (APCI): calc. for $C_{20}H_{17}^+$ $[M+H]^+$: 257.1325; found: 257.1324.

Spectral data are in accordance with those reported in the literature.[8]

*7.2.2.12 2-[(1E,3E)-4-phenyl-1,3-butadien-1-yl]naphthalene (**1l**)*

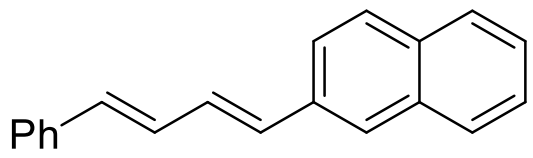

Obtained as a white amorphous solid according to GP3:

▪ 546 mg, 3.50 mmol, 1.0 equiv 2-naphthaldehyde
▪ 1072 mg, 4.21 mmol, 1.2 equiv diethyl *P*[(2*E*)-3-phenyl-2-propen-1-yl]phosphonate
▪ 1.7 mL, 4.25 mmol, 1.2 equiv *n*-BuLi (2.5 M in *n*-hexane)
▪ 30 mL anhydrous THF as solvent
▪ Purified by flash column chromatography (SiO_2, 20% Et_2O in *n*-pentane)

Yield: 752 mg (2.93 mmol, 84%).

^{1}H NMR (400 MHz, CD_2Cl_2): δ 7.88 – 7.77 (m, 4H), 7.69 (dd, *J* = 8.5, 1.8 Hz, 1H), 7.52 – 7.42 (m, 4H), 7.35 (t, *J* = 7.6 Hz, 2H), 7.25 (t, *J* = 7.3 Hz, 1H), 7.18 – 7.00 (m, 2H), 6.87 (d, *J* = 14.4 Hz, 1H), 6.75 (d, *J* = 14.7 Hz, 1H).

^{13}C NMR (101 MHz, CD_2Cl_2): δ 137.8, 135.4, 134.1, 133.4, 133.4, 133.2, 130.1, 129.7, 129.1, 128.6, 128.3, 128.0, 126.8, 126.3, 123.8.

IR (ATR, cm^{-1}): 994 (s), 967 (m), 906 (m), 861 (m), 815 (s), 747 (s), 688 (s), 505 (m), 480 (s).

HRMS (APCI): calc. for $C_{20}H_{17}^+$ $[M+H]^+$: 257.1325; found: 257.1328.

Spectral data are in accordance with those reported in the literature.[2]

*7.2.2.13 1-[(1E,3E)-4-phenyl-1,3-butadien-1-yl]pyrene (**1m**)*

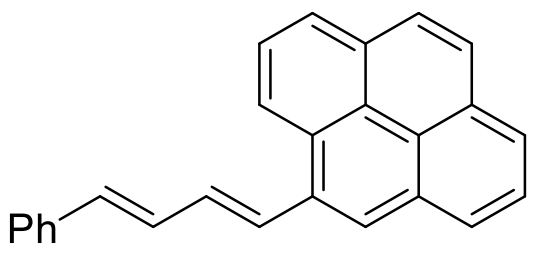

Obtained as a yellow amorphous solid according to GP3:

- 805 mg, 3.50 mmol, 1.0 equiv 1-pyrenecarboxaldehyde
- 1076 mg, 4.23 mmol, 1.2 equiv diethyl *P*[(2*E*)-3-phenyl-2-propen-1-yl]phosphonate
- 1.7 mL, 4.25 mmol, 1.2 equiv *n*-BuLi (2.5 M in *n*-hexane)
- 30 mL anhydrous THF as solvent
- Purified by crystallization from 40 mL *n*-hexane/toluene (1:1).

Yield: 938 mg (2.84 mmol, 81%).

^{1}H NMR (400 MHz, $CDCl_3$): δ 8.46 (d, J = 9.3 Hz, 1H), 8.29 (d, J = 8.1 Hz, 1H), 8.22 – 8.10 (m, 4H), 8.04 (s, 2H), 8.02 – 7.97 (m, 1H), 7.82 – 7.72 (m, 1H), 7.57 – 7.48 (m, 2H), 7.38 (dd, J = 8.4, 6.9 Hz, 2H), 7.32 – 7.16 (m, 3H), 6.87 – 6.76 (m, 1H).

^{13}C NMR (101 MHz, $CDCl_3$): δ 137.6, 133.5, 132.2, 131.8, 131.7, 131.1, 131.0, 129.9, 129.6, 128.9, 128.3, 127.8, 127.8, 127.6, 127.4, 126.6, 126.2, 125.5, 125.3, 125.3, 125.2, 125.1, 123.4, 123.0.

IR (ATR, cm^{-1}): 983 (s), 837 (s), 808 (m), 748 (s), 710 (s), 692 (s), 607 (m), 512 (m), 503 (m), 492 (m).

HRMS (APCI): calc. for $C_{26}H_{19}^+$ $[M+H]^+$: 331.1481; found: 331.1508.

Spectral data are in accordance with those reported in the literature.[8]

*7.2.2.14 1-Chloro-2-[(1E,3E)-4-pheynl-1,3-butadien-1-yl]benzene (**1n**)*

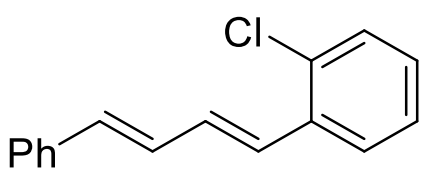

Obtained as a white amorphous solid according to GP3:

- 985 mg, 7.00 mmol, 1.0 equiv 2-chlorobenzaldehyde
- 2139 mg, 8.41 mmol, 1.2 equiv diethyl P[(2*E*)-3-phenyl-2-propen-1-yl]phosphonate
- 3.4 mL, 8.50 mmol, 1.2 equiv *n*-BuLi (2.5 M in *n*-hexane)
- 60 mL anhydrous THF as solvent
- Purified by flash column chromatography (SiO_2, *n*-pentane)

Yield: 1496 mg (6.22 mmol, 89%).

^{1}H NMR (400 MHz, CD_2Cl_2): δ 7.67 (dd, J = 7.8, 1.7 Hz, 1H), 7.51 – 7.45 (m, 2H), 7.42 – 7.30 (m, 3H), 7.32 – 7.21 (m, 2H), 7.19 (td, J = 7.6, 1.7 Hz, 1H), 7.14 – 6.94 (m, 3H), 6.75 (d, J = 15.3 Hz, 1H).

^{13}C NMR (101 MHz, CD_2Cl_2): δ 137.6, 135.6, 134.4, 133.4, 132.1, 130.2, 129.5, 129.1, 128.9, 128.6, 128.3, 127.4, 126.9, 126.6.

IR (ATR, cm^{-1}): 1469 (m), 1439 (m), 1032 (m), 985 (s), 823 (m), 758 (m), 742 (s), 698 (m), 687 (s), 504 (m), 450 (m).

HRMS (APCI): calc. for $C_{16}H_{14}Cl^+$ $[M+H]^+$: 241.0779; found: 241.0781.

Spectral data are in accordance with those reported in the literature.[9]

*7.2.2.15 1-Chloro-4-[(1E,3E)-4-phenyl-1,3-butadien-1-yl]benzene (**1o**)*

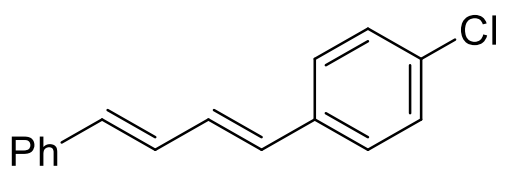

Obtained as a white amorphous solid according to GP3:

- 492 mg, 3.50 mmol, 1.0 equiv 4-chlorobenzaldehyde
- 1090 mg, 4.29 mmol, 1.2 equiv diethyl *P*[(2*E*)-3-phenyl-2-propen-1-yl]phosphonate
- 2.7 mL, 4.32 mmol, 1.2 equiv *n*-BuLi (1.6 M in *n*-hexane)
- 30 mL anhydrous THF as solvent
- Purified by flash column chromatography (SiO_2, 10% Et_2O in *n*-pentane)

Yield: 744 mg (3.09 mmol, 88%).

^{1}H NMR (400 MHz, $CDCl_3$): δ 7.48 – 7.41 (m, 2H), 7.40 – 7.20 (m, 7H), 7.00 – 6.86 (m, 2H), 6.74 – 6.56 (m, 2H).

^{13}C NMR (101 MHz, $CDCl_3$): δ 137.3, 136.0, 133.6, 133.2, 131.5, 130.0, 129.0, 129.0, 128.8, 127.9, 127.6, 126.6.

IR (ATR, cm^{-1}): 1486 (m), 1447 (m), 1405 (m), 1092 (m), 1073 (m), 983 (s), 847 (m), 840 (m), 809 (m), 746 (s), 689 (s), 513 (m), 493 (s).

HRMS (APCI): calc. for $C_{16}H_{14}Cl^+$ $[M+H]^+$: 241.0779; found: 241.0780.

Spectral data are in accordance with those reported in the literature.[2]

*7.2.2.16 1-Bromo-2-[(1E,3E)-4-phenyl-1,3-butadien-1-yl]benzene (**1p**)*

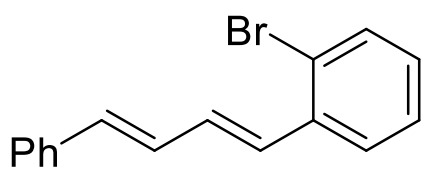

Obtained as a white amorphous solid according to GP3:

- 648 mg, 3.50 mmol, 1.0 equiv 2-bromobenzaldehyde
- 1061 mg, 4.17 mmol, 1.2 equiv diethyl *P*[(2*E*)-3-phenyl-2-propen-1-yl]phosphonate
- 1.7 mL, 4.25 mmol, 1.2 equiv *n*-BuLi (2.5 M in *n*-hexane)
- 30 mL anhydrous THF as solvent
- Purified by flash column chromatography (SiO_2, 1% Et_2O in *n*-pentane)

Yield: 758 mg (2.66 mmol, 76%).

^{1}H NMR (400 MHz, CD_2Cl_2): δ 7.65 (dd, J = 7.9, 1.7 Hz, 1H), 7.58 (dd, J = 8.0, 1.3 Hz, 1H), 7.50 – 7.45 (m, 2H), 7.38 – 7.23 (m, 4H), 7.14 – 7.02 (m, 3H), 7.00 – 6.91 (m, 1H), 6.75 (d, J = 15.9 Hz, 1H).

^{13}C NMR (101 MHz, CD_2Cl_2): δ 137.6, 137.3, 134.4, 133.5, 132.3, 131.3, 129.4, 129.2, 129.1, 128.3, 128.0, 126.9, 126.8, 124.2.

IR (ATR, cm^{-1}): 1435 (m), 1020 (m), 984 (s), 822 (m), 741 (s), 687 (s), 673 (m), 629 (m), 501 (m), 465 (m), 443 (m).

HRMS (APCI): calc. for $C_{16}H_{14}Br^+$ $[M+H]^+$: 285.0273; found: 285.0270.

Spectral data are in accordance with those reported in the literature.[10]

*7.2.2.17 1-Bromo-3-[(1E,3E)-4-phenyl-1,3-butadien-1-yl]benzene (**1q**)*

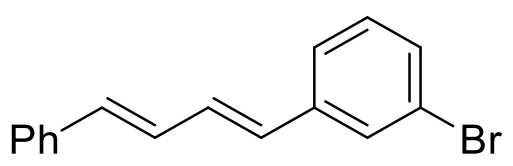

Obtained as a white amorphous solid according to GP3:

- 643 mg, 3.48 mmol, 1.0 equiv 3-bromobenzaldehyde
- 1075 mg, 4.23 mmol, 1.2 equiv diethyl *P*[(2*E*)-3-phenyl-2-propen-1-yl]phosphonate
- 1.7 mL, 4.25 mmol, 1.2 equiv *n*-BuLi (2.5 M in *n*-hexane)
- 30 mL anhydrous THF as solvent
- Purified by flash column chromatography (SiO_2, 1% Et_2O in *n*-pentane)

Yield: 774 mg (2.71 mmol, 78%).

^{1}H NMR (400 MHz, CD_2Cl_2): δ 7.61 (t, J = 1.8 Hz, 1H), 7.49 – 7.44 (m, 2H), 7.42 – 7.31 (m, 4H), 7.30 – 7.18 (m, 2H), 7.04 – 6.92 (m, 2H), 6.78 – 6.57 (m, 2H).

^{13}C NMR (101 MHz, CD_2Cl_2): δ 140.1, 137.5, 134.3, 131.4, 131.1, 130.6, 129.5, 129.1, 128.2, 126.9, 125.4, 123.1.

IR (ATR, cm^{-1}): 1422 (m), 1071 (m), 987 (s), 880 (m), 829 (m), 781 (m), 748 (s), 694 (m), 681 (m), 673 (m), 502 (m), 430 (m).

HRMS (APCI): calc. for $C_{16}H_{14}Br^+$ $[M+H]^+$: 285.0273; found: 285.0272.

*7.2.2.18 1-Bromo-4-[(1E,3E)-4-phenyl-1,3-butadien-1-yl]benzene (**1r**)*

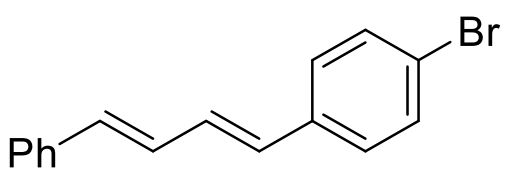

Obtained as a white amorphous solid according to GP3:

- 651 mg, 3.52 mmol, 1.0 equiv 4-bromobenzaldehyde
- 1081 mg, 4.23 mmol, 1.2 equiv diethyl *P*[(2*E*)-3-phenyl-2-propen-1-yl]phosphonate
- 2.8 mL, 4.48 mmol. 1.3 equiv *n*-BuLi (1.6 M in *n*-hexane)
- 30 mL anhydrous THF as solvent
- Purified by flash column chromatography (SiO_2, 10% Et_2O in *n*-pentane)

Yield: 908 mg (3.18 mmol, 91%).

^{1}H NMR (400 MHz, $CDCl_3$): δ 7.50 – 7.41 (m, 4H), 7.40 – 7.21 (m, 5H), 6.99 – 6.89 (m, 2H), 6.75 – 6.54 (m, 2H).

^{13}C NMR (101 MHz, $CDCl_3$): δ 137.3, 136.5, 133.7, 131.9, 131.5, 130.1, 129.0, 128.9, 128.0, 127.9, 126.6, 121.4.

IR (ATR, cm^{-1}): 1483 (m), 1447 (m), 1399 (m), 1070 (m), 990 (s), 846 (m), 806 (m), 746 (s), 689 (s), 498 (m), 482 (m).

HRMS (APCI): calc. for $C_{16}H_{14}Br^+$ $[M+H]^+$: 285.0273; found: 285.0274.

Spectral data are in accordance with those reported in the literature.[2]

*7.2.2.19 1-Iodo-4-[(1E,3E)-4-phenyl-1,3-butadien-1-yl]benzene (**1s**)*

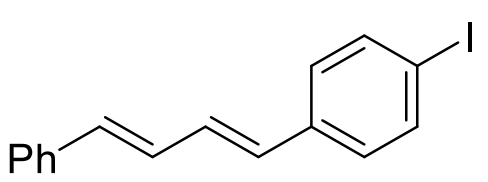

Obtained as a white amorphous solid according to GP3:

- 812 mg, 3.50 mmol, 1.0 equiv 4-iodobenzaldehyde
- 1066 mg, 4.19 mmol, 1.2 equiv diethyl *P*[(2*E*)-3-phenyl-2-propen-1-yl]phosphonate
- 2.6 mL, 4.16 mmol. 1.3 equiv *n*-BuLi (1.6 M in *n*-hexane)
- 30 mL anhydrous THF as solvent
- Purified by flash column chromatography (SiO_2, 5% Et_2O in *n*-pentane)

Yield: 591 mg (1.78 mmol, 51%).

^{1}H NMR (400 MHz, CD_2Cl_2): δ 7.72 – 7.63 (m, 2H), 7.49 – 7.42 (m, 2H), 7.38 – 7.30 (m, 2H), 7.29 – 7.16 (m, 3H), 7.05 – 6.92 (m, 2H), 6.76 – 6.67 (m, 1H), 6.66 – 6.56 (m, 1H).

^{13}C NMR (101 MHz, CD_2Cl_2): δ 138.1, 137.6, 137.4, 133.9, 131.8, 130.5, 129.2, 129.1, 128.5, 128.2, 126.8, 92.9.

IR (ATR, cm^{-1}): 1480 (m), 1446 (m), 1396 (m), 986 (s), 912 (m), 844 (m), 802 (s), 743 (s), 688 (s), 496 (s), 474 (m).

HRMS (APCI): calc. for $C_{16}H_{14}I^{+}$ $[M+H]^{+}$: 333.0135; found: 333.0137.

Spectral data are in accordance with those reported in the literature.[11]

*7.2.2.20 1-Trifluoromethyl-4-[(1E,3E)-4-phenyl-1,3-butadien-1-yl]benzene (**1t**)*

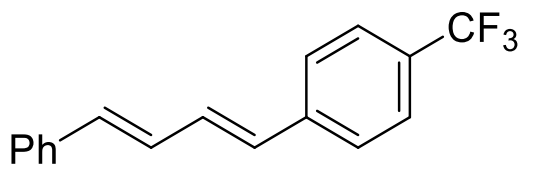

Obtained as a white amorphous solid according to GP3:

- 610 mg, 3.50 mmol, 1.0 equiv 4-(trifluoromethyl)benzaldehyde
- 1080 mg, 4.25 mmol, 1.2 equiv diethyl *P*[(2*E*)-3-phenyl-2-propen-1-yl]phosphonate
- 2.8 mL, 4.48 mmol, 1.3 equiv *n*-BuLi (1.6 M in *n*-hexane)
- 30 mL anhydrous THF as solvent
- Purified by flash column chromatography (SiO_2, 10% Et_2O in *n*-pentane)

Yield: 804 mg (2.93 mmol, 84%).

^{1}H NMR (400 MHz, $CDCl_3$): δ 7.58 (d, *J* = 8.3 Hz, 2H), 7.52 (d, *J* = 8.7 Hz, 2H), 7.48 – 7.44 (m, 2H), 7.38 – 7.32 (m, 2H), 7.29 – 7.23 (m, 1H), 7.08 – 6.91 (m, 2H), 6.71 (dd, *J* = 24.9, 14.8 Hz, 2H).

^{13}C NMR (101 MHz, $CDCl_3$): δ 141.0, 137.1, 134.7, 131.8, 131.2, 129.4, 128.9, 128.8, 128.2, 126.7, 126.5, 125.8 (q, *J* = 4.1 Hz), 123.0.

^{19}F NMR (377 MHz, $CDCl_3$): δ – 62.5.

IR (ATR, cm^{-1}): 1320 (m), 1161 (m), 1106 (s), 1067 (m), 985 (s), 969 (m), 852 (m), 844 (m), 754 (s), 733 (m), 693 (m), 597 (m), 504 (m).

HRMS (APCI): calc. for $C_{17}H_{14}F_3^{+}$ $[M+H]^{+}$: 275.1042; found: 275.1042.

Spectral data are in accordance with those reported in the literature.[2]

*7.2.2.21 Methyl-4-[(1E,3E)-4-phenyl-1,3-butadien-1-yl]benzoate (**1u**)*

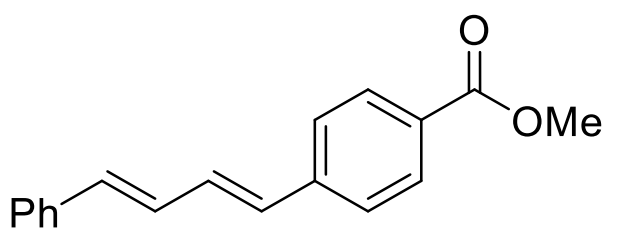

Obtained as a white amorphous solid according to GP3:

- 565 mg, 3.44 mmol methyl, 1.0 equiv 4-fromylbenzaldehyde
- 1065 mg, 4.19 mmol, 1.2 equiv diethyl *P*[(2*E*)-3-phenyl-2-propen-1-yl]phosphonate
- 1.7 mL, 4.25 mmol, 1.2 equiv *n*-BuLi (2.5 M in *n*-hexane)
- 30 mL anhydrous THF as solvent
- Purified by flash column chromatography (SiO_2, 20% Et_2O in *n*-pentane)

Yield: 740 mg (2.80 mmol, 81%).

^{1}H NMR (400 MHz, $CDCl_3$): δ 8.02 – 7.97 (m, 2H), 7.52 – 7.43 (m, 4H), 7.38 – 7.31 (m, 2H), 7.29 – 7.23 (m, 1H), 7.11 – 6.92 (m, 2H), 6.72 (dd, *J* = 21.0, 15.0 Hz, 2H), 3.92 (s, 3H).

^{13}C NMR (101 MHz, $CDCl_3$): δ 167.0, 142.0, 137.2, 134.7, 131.9, 131.7, 130.2, 128.9, 128.9, 128.1, 126.7, 126.3, 52.2.

IR (ATR, cm^{-1}): 1708 (m), 1413 (m), 1278 (m), 1102 (m), 985 (s), 974 (m), 860 (m), 747 (s), 692 (s), 499 (m).

HRMS (APCI): calc. for $C_{18}H_{17}O_2^{+}$ $[M+H]^{+}$: 265.1223; found: 265.1224.

Spectral data are in accordance with those reported in the literature.[6]

*7.2.2.22 4-[(1E,3E)-4-phenyl-1,3-butadien-1-yl]benzonitrile (**1v**)*

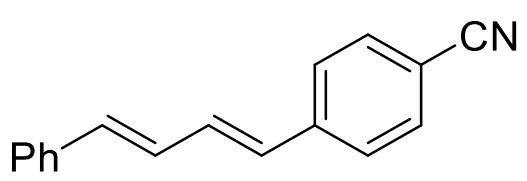

Obtained as a white amorphous solid according to GP3:

- 461 mg, 3.52 mmol, 1.0 equiv 4-cyanobenzaldehyde
- 1069 mg, 4.20 mmol, 1.2 equiv diethyl *P*[(2*E*)-3-phenyl-2-propen-1-yl]phosphonate
- 1.7 mL, 4.25 mmol, 1.2 equiv *n*-BuLi (2.5 M in *n*-hexane)
- 30 mL anhydrous THF as solvent
- Purified by flash column chromatography (SiO_2, 50% DCM in *n*-hexane)

Yield: 593 mg (2.56 mmol, 73%).

^{1}H NMR (400 MHz, CD_2Cl_2): δ 7.65 – 7.60 (m, 2H), 7.56 – 7.51 (m, 2H), 7.50 – 7.45 (m, 2H), 7.39 – 7.33 (m, 2H), 7.30 – 7.24 (m, 1H), 7.15 – 6.94 (m, 2H), 6.79 (d, *J* = 15.2 Hz, 1H), 6.69 (d, *J* = 15.2 Hz, 1H).

^{13}C NMR (101 MHz, CD_2Cl_2): δ 142.3, 137.3, 135.7, 133.2, 132.9, 131.0, 129.1, 128.8, 128.6, 127.1, 127.0, 119.4, 110.8.

IR (ATR, cm^{-1}): 987 (s), 967 (m), 854 (m), 843 (m), 815 (m), 751 (s), 718 (m), 693 (s). 554 (s), 508 (m), 473 (m).

HRMS (APCI): calc. for $C_{17}H_{14}N^+$ [M+H]$^+$: 232.1121; found: 232.1121.

Spectral data are in accordance with those reported in the literature.[6]

*7.2.2.23 1-Nitro-4-[(1E,3E)-4-phenyl-1,3-butadien-1-yl]benzene (**1w**)*

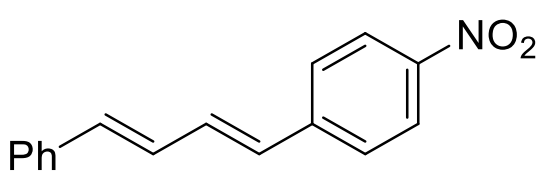

Obtained as an orange amorphous solid according to GP3:

- 1052 mg, 6.96 mmol, 1.0 equiv 4-nitrobenzaldehyde
- 2121 mg, 8.34 mmol, 1.2 equiv diethyl *P*[(2*E*)-3-phenyl-2-propen-1-yl]phosphonate
- 3.4 mL, 8.50 mmol, 1.2 equiv *n*-BuLi (2.5 M in *n*-hexane)
- 60 mL anhydrous THF as solvent
- Purified by flash column chromatography (SiO_2, 10% Et_2O in *n*-pentane)

Yield: 800 mg (3.18 mmol, 46%).

^{1}H NMR (400 MHz, CD_2Cl_2): δ 8.22 – 8.14 (m, 2H), 7.61 – 7.56 (m, 2H), 7.49 (dd, *J* = 7.2, 1.8 Hz, 2H), 7.36 (td, *J* = 6.7, 6.1, 1.6 Hz, 2H), 7.31 – 7.26 (m, 1H), 7.16 (dd, *J* = 15.4, 10.6 Hz, 1H), 7.02 (dd, *J* = 15.1, 10.6 Hz, 1H), 6.83 (d, *J* = 15.4 Hz, 1H), 6.75 (d, *J* = 15.4 Hz, 1H).

^{13}C NMR (101 MHz, CD_2Cl_2): δ 147.0, 144.4, 137.2, 136.3, 134.2, 130.5, 129.2, 128.8, 128.7, 127.1, 124.4.

IR (ATR, cm^{-1}): 1586 (m), 1511 (s), 1341 (s), 1108 (m), 992 (s), 857 (m), 760 (m), 743 (s), 688 (m), 490 (m).

HRMS (ESI): calc. for $C_{16}H_{13}NNaO_2^+$ [M+Na]$^+$: 274.0839; found: 274.0839.

Spectral data are in accordance with those reported in the literature.[6]

*7.2.2.24 1,3-Hexadien-1-yl]benzene (**1x**)*

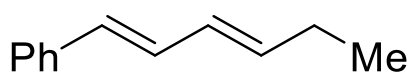

Obtained as a colorless liquid according to GP3:

- 413 mg, 6.99 mmol, 1.0 equiv propanal
- 2135 mg, 8.40 mmol, 1.2 equiv diethyl *P*[(2*E*)-3-phenyl-2-propen-1-yl]phosphonate
- 3.5 mL, 8.33 mmol, 1.2 equiv *n*-BuLi (2.38 M in *n*-hexane)
- 60 mL anhydrous THF as solvent

▪ Purified by flash column chromatography (SiO_2, *n*-pentane)

Yield: 939 mg (11:1 d.r., 5.93 mmol, 85%).

^{1}H NMR (400 MHz, CD_2Cl_2): δ 7.41 – 7.36 (m, 2H), 7.34 – 7.26 (m, 2H), 7.22 – 7.16 (m, 1H), 6.79 (dd, *J* = 15.7, 10.3 Hz, 1H), 6.45 (d, *J* = 15.6 Hz, 1H), 6.28 – 6.17 (m, 1H), 5.90 (dt, *J* = 15.2, 6.7 Hz, 1H), 2.18 (quint, *J* = 7.5 Hz, 2H), 1.05 (t, *J* = 7.5 Hz, 3H).

^{13}C NMR (101 MHz, CD_2Cl_2): δ 138.1, 138.0, 130.2, 129.9, 129.9, 128.9, 127.5, 126.5, 26.3, 13.7.

IR (ATR, cm^{-1}): 3021 (w), 2963 (m), 1495 (w), 1448 (m), 983 (s), 743 (s), 689 (s), 506 (m).

HRMS (APCI): calc. for $C_{12}H_{15}^+$ $[M+H]^+$: 159.1168; found: 159.1174.

Spectral data are in accordance with those reported in the literature.[12]

*7.2.2.25 [(1E,3E)-4-Cyclohexyl-1,3-butadien-1-yl]benzene (**1y**)*

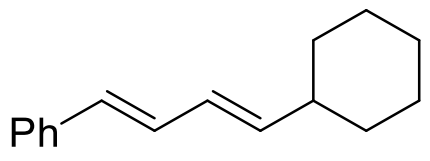

Obtained as a colorless liquid according to GP3:

- 785 mg, 7.00 mmol, 1.0 equiv cyclohexanecarboxaldehyde
- 2129 mg, 8.37 mmol, 1.2 equiv diethyl *P*[(2*E*)-3-phenyl-2-propen-1-yl]phosphonate
- 3.5 mL, 8.33 mmol, 1.2 equiv *n*-BuLi (2.38 M in *n*-hexane)
- 60 mL anhydrous THF as solvent
- Purified by flash column chromatography (SiO_2, *n*-pentane)

Yield: 1311 mg (6.17 mmol, 88%).

^{1}H NMR (400 MHz, CD_2Cl_2): δ 7.41 – 7.35 (m, 2H), 7.33 – 7.26 (m, 2H), 7.23 – 7.16 (m, 1H), 6.77 (dd, *J* = 16.1, 10.8 Hz, 1H), 6.45 (d, *J* = 15.6 Hz, 1H), 6.19 (dd, *J* = 15.6, 10.1 Hz, 1H), 5.81 (dd, *J* = 15.4, 7.0 Hz, 1H), 2.16 – 2.00 (m, 1H), 1.82 – 1.71 (m, 4H), 1.70 – 1.62 (m, 1H), 1.40 – 1.07 (m, 5H).

^{13}C NMR (101 MHz, CD_2Cl_2): δ 142.3, 138.2, 130.3, 130.2, 128.9, 128.3, 127.4, 126.4, 41.4, 33.3, 26.6, 26.4.

IR (ATR, cm^{-1}): 3022 (w), 2920 (m), 2849 (m), 1596 (w), 1496 (w), 1446 (m), 984 (s), 892 (w), 743 (s), 689 (s), 509 (m).

HRMS (APCI): calc. for $C_{16}H_{21}^+$ $[M+H]^+$: 213.1638; found: 213.1650.

Spectral data are in accordance with those reported in the literature.[13]

7.2.3 1,4-Diaryl substituted (1*E*,3*E*)-1,3-butadienes (1z and 1aa)

*7.2.3.1 (1E,3E)-1,4-Di(4-methylphenyl)-1,3-butadien (**1z**)*

Step 1: Ethyl (2*E*)-3-(4-methylphenyl)-2-propenoate (**S6**)

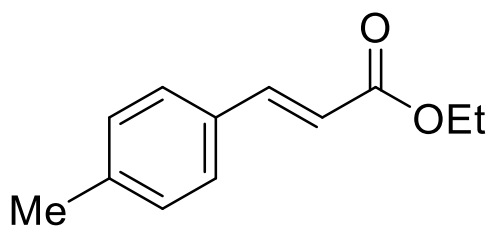

Obtained as a white amorphous solid according to GP1:

- 1201 mg, 10.00 mmol, 1.0 equiv 4-methylbenzaldehyde
- 2691 mg, 12.00 mmol, 1.2 equiv triethyl phosphonoacetate
- 329 mg, 12.33 mmol, 1.2 equiv NaH (90% dry)
- 45 mL anhydrous THF as solvent
- Purified by flash column chromatography (SiO_2, 10% Et_2O in *n*-pentane)

Yield: 1812 mg (9.52 mmol, 95%).

^{1}H NMR (400 MHz, $CDCl_3$): δ 7.66 (d, *J* = 16.0 Hz, 1H), 7.42 (d, *J* = 8.2 Hz, 2H), 7.19 (d, *J* = 7.8 Hz, 1H), 6.39 (d, *J* = 16.0 Hz, 1H), 4.26 (q, *J* = 7.1 Hz, 2H), 2.37 (s, 3H), 1.34 (t, *J* = 7.1 Hz, 3H).

^{13}C NMR (101 MHz, $CDCl_3$): δ 167.4, 144.7, 140.8, 131.9, 129.8, 128.2, 117.3, 60.6, 21.6, 14.5.

Spectral data are in accordance with those reported in the literature.[14]

Step 2: (2*E*)-3-(4-Methylphenyl)-2-propen-1-ol (**S7**)

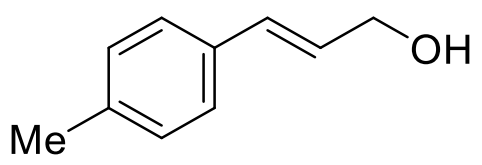

In an oven dried Schlenk-flask under an inert gas atmosphere 1798 mg ethyl (2*E*)-3-(4-methylphenyl)-2-propenoate (9.45 mmol, 1.0 equiv) was dissolved in 48 mL anhydrous DCM and cooled to –78 °C. 21 mL DIBAL (1 M in DCM, 21.0 mmol, 2.2 equiv) was added dropwise and the resulting mixture was warmed to r.t. and stirred overnight. After complete reaction 100 mL 10% aq. NaOH solution was added carefully. The resulting mixture was stirred for 20 min at r.t. and then transferred to a separatory funnel. The phases were separated and the aqueous phase was extracted with DCM (2 x 80 mL). The combined organic phases were dried over $MgSO_4$, filtered and the solvent was removed under reduced pressure to yield a white amorphous solid. The product was used in the next step without further purification.

Yield: 1398 mg (9.43 mmol, 99%).

^{1}H NMR (400 MHz, $CDCl_3$): δ 7.29 (d, *J* = 8.2 Hz, 2H), 7.13 (d, *J* = 7.8 Hz, 2H), 6.59 (d, *J* = 15.9 Hz, 1H), 6.32 (dt, *J* = 15.9, 5.9 Hz, 1H), 4.31 (dd, *J* = 5.9, 1.5 Hz, 2H), 2.34 (s, 3H).

^{13}C NMR (101 MHz, $CDCl_3$): δ 137.7, 134.0, 131.4, 129.4, 127.6, 126.5, 64.0, 21.3.

Spectral data are in accordance with those reported in the literature.[15]

Step 3: 1-[(1*E*)-3-Bromo-1-propen-1-yl]-4-methylbenzene (**S8**)

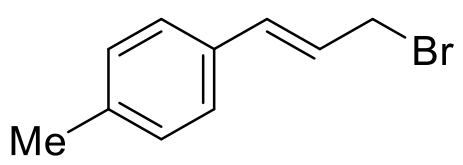

Under an inert gas atmosphere in a round-bottom flask 1398 mg (2*E*)-3-(4-methylphenyl)-2-propen-1-ol (9.43 mol, 1.0 equiv) was dissolved in 20 mL Et_2O, then 1 mL PBr_3 (2850 mg, 10.53 mmol, 1.1 equiv) was added dropwise and the resulting mixture was stirred overnight. The mixture was cooled to 0 °C, 10 mL sat. $NaHCO_3$ was added carefully and then transferred into a separatory funnel. The phases were separated and the aqueous phase was extracted with Et_2O (3 x 20 mL). The combined organic phases were dried over $MgSO_4$, filtered and the solvent was removed under reduced pressure to yield a white amorphous solid. The product was used in the next step without further purification.

Yield: 1964 mg (9.30 mmol, 99%).

^{1}H NMR (400 MHz, $CDCl_3$): δ 7.29 (d, *J* = 8.2 Hz, 2H), 7.14 (d, *J* = 7.7 Hz, 2H), 6.62 (d, *J* = 15.5 Hz, 1H), 6.35 (dt, *J* = 15.5, 7.8 Hz, 1H), 4.17 (dd, *J* = 7.8, 1.0 Hz, 2H), 2.35 (s, 3H).

^{13}C NMR (101 MHz, $CDCl_3$): δ 138.5, 134.7, 133.2, 129.5, 126.8, 124.3, 34.0, 21.4.

Spectral data are in accordance with those reported in the literature.[16]

Step 4: Diethyl *P*[(2*E*)-3-(4-methylphenyl)-2-propenyl]phosphonate (**S9**)

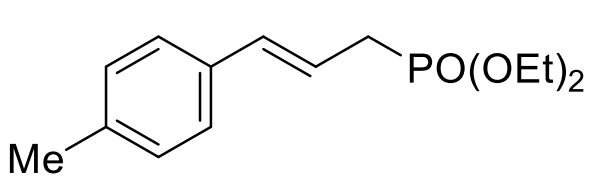

Obtained as a colorless liquid according to GP2:

- 1964 mg, 9.30 mmol, 1.0 equiv 1-[(1*E*)-Bromo-1-propen-1-yl}-4-methylbenzene
- 1.92 g, 11.56 mmol, 1.2 equiv triethyl phosphite
- Purified by distillation (0.95 Torr, 140 to 143 °C)

Yield: 1673 mg (7.92 mmol, 85%).

^{1}H NMR (400 MHz, $CDCl_3$): δ 7.26 (d, *J* = 8.1 Hz, 2H), 7.11 (d, *J* = 7.9 Hz, 2H), 6.49 (dd, *J* = 15.8, 5.2 Hz, 1H), 6.11 (dq, *J* = 15.6, 7.4 Hz, 1H), 4.21 – 4.03 (m, 4H), 2.78 (dd, *J* = 7.6, 1.4 Hz, 1H), 2.72 (dd, *J* = 7.6, 1.4 Hz, 1H), 2.33 (s, 3H), 1.32 (t, *J* = 7.0 Hz, 6H).

^{13}C NMR (101 MHz, $CDCl_3$): δ 137.6 (d, *J* = 1.5 Hz), 134.7 (d, *J* = 15.0 Hz), 134.2 (d, *J* = 3.5 Hz), 129.4, 126.3 (d, *J* = 1.9 Hz), 117.8 (d, *J* = 11.9 Hz), 62.19 (d, *J* = 6.6 Hz), 31.24 (d, *J* = 139.9 Hz), 21.32, 16.63 (d, *J* = 5.8 Hz).

^{31}P NMR (162 MHz, $CDCl_3$): δ 28.0.

Spectral data are in accordance with those reported in the literature.[5]

Step 5: (1*E*,3*E*)-1,4-Di(4-methylphenyl)-1,3-butadien (**1z**)

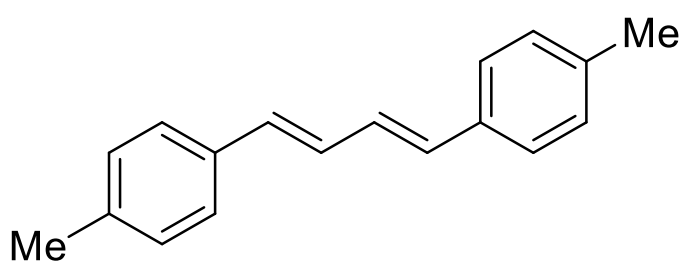

Obtained as a white amorphous solid according to GP3:

- 603 mg, 5.02 mmol, 1.0 equiv 4-methylbenzaldehyde
- 1615 mg, 6.02 mmol, 1.2 equiv diethyl *P*[(2*E*)-3-(4-methylphenyl)-2-propen-1-yl]phosphonate
- 2.4 mL, 6.00 mmol, 1.2 equiv *n*-BuLi (2.5 M in *n*-hexane)
- 46 mL anhydrous THF as solvent
- Purified by flash column chromatography (SiO_2, 20% Et_2O in *n*-pentane)

Yield: 863 mg (3.68 mmol, 73%).

^{1}H NMR (400 MHz, $CDCl_3$): δ 7.33 (d, *J* = 10.4 Hz, 4H), 7.14 (d, *J* = 8.1 Hz, 4H), 6.96 – 6.85 (m, 2H), 6.69 – 6.56 (m, 2H), 2.35 (s, 6H).

^{13}C NMR (101 MHz, $CDCl_3$): δ 137.5, 134.9, 132.4, 129.5, 128.7, 126.4, 21.4.

IR (ATR, cm^{-1}): 1507 (m), 991 (s), 962 (m), 849 (m), 832 (m), 791 (s), 499 (s).

HRMS (APCI): calc. for $C_{18}H_{19}^+$ $[M+H]^+$: 235.1481; found: 235.1484.

Spectral data are in accordance with those reported in the literature.[17]

*7.2.3.2 (1E,3E)-1,4-Di(4-bromophenyl)-1,3-butadien (**1aa**)*

Step 1: Ethyl (2*E*)-3-(4-bromophenyl)-2-propenoate (**S10**)

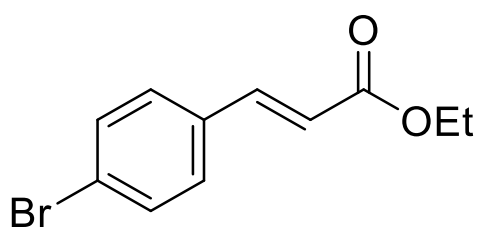

Obtained as a colorless liquid according to GP1:

- 3727 mg, 20.14 mmol, 1.0 equiv 4-bromobenzaldehyde
- 5860 mg, 26.13 mmol, 1.3 equiv triethyl phosphonoacetate
- 750 mg, 28.13 mmol, 1.4 equiv NaH (90% dry)
- 100 mL anhydrous THF as solvent
- Purified by flash column chromatography (SiO_2, 20% Et_2O in *n*-pentane)

Yield: 3821 mg (14.98 mmol, 74%).

^{1}H NMR (400 MHz, $CDCl_3$): δ 7.61 (d, *J* = 16.0 Hz, 1H), 7.55 – 7.49 (m, 2H), 7.42 – 7.35 (m, 2H), 6.42 (d, *J* = 16.0 Hz, 1H), 4.26 (q, *J* = 7.1 Hz, 2H), 1.33 (t, *J* = 7.2 Hz, 3H).

^{13}C NMR (101 MHz, $CDCl_3$): δ 166.9, 143.3, 133.5, 132.3, 129.6, 124.6, 119.1, 60.8, 14.5.

Spectral data are in accordance with those reported in the literature.[14]

Step 2: (2*E*)-3-(4-Bromophenyl)-2-propen-1-ol (**S11**)

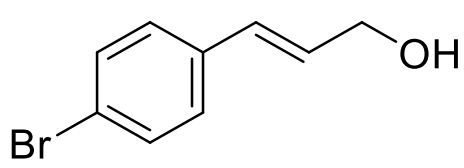

In an oven dried Schlenk-flask under an inert gas atmosphere 3821 mg ethyl (2*E*)-3-(4-bromophenyl)-2-propenoate (14.98 mmol, 1.0 equiv) was dissolved in 100 mL anhydrous DCM and cooled to –78 °C. 33 mL DIBAL (1 M in DCM, 33.0 mmol, 2.2 equiv) was added dropwise and the resulting mixture was warmed to r.t. and stirred overnight. After complete reaction 200 mL 10% aq. NaOH solution was added carefully. The resulting mixture was

stirred for 20 minutes at r.t. and then transferred to a separatory funnel. The phases were separated and the aqueous phase was extracted with DCM (2 x 80 mL). The combined organic phases were dried over $MgSO_4$, filtered and the solvent was removed under reduced pressure to yield a white amorphous solid. The product was used in the next step without further purification.

Yield: 3006 mg (14.11 mmol, 94%).

^{1}H NMR (400 MHz, $CDCl_3$): δ 7.48 – 7.40 (m, 2H), 7.29 – 7.21 (m, 2H), 6.56 (d, *J* = 15.9 Hz, 1H), 6.35 (dt, *J* = 15.9, 5.6 Hz, 1H), 4.32 (dd, *J* = 5.6, 1.5 Hz, 2H).

^{13}C NMR (101 MHz, $CDCl_3$): δ 135.8, 131.9, 129.9, 129.5, 128.1, 121.6, 63.7.

Spectral data are in accordance with those reported in the literature.[18]

Step 3:1-[(1*E*)-3-Bromo-1-propen-1-yl]-4-methylbenzene (**S12**)

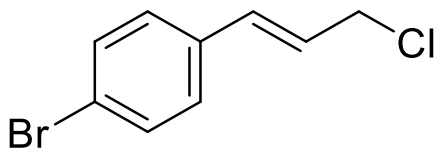

Under an inert gas atmosphere in a round-bottom flask 3000 mg (2*E*)-3-(4-bromophenyl)-2-propen-1-ol (14.08 mol, 1.0 equiv) was dissolved in 50 mL anhydrous DCM, then 2 mL $SOCl_2$ (3272 mg, 27.50 mmol, 2.0 equiv) was added dropwise and the resulting mixture was stirred overnight at 50 °C. The reaction was cooled to 0°C, quenched with 75 mL water and then transferred to a separatory funnel. The phases were separated and the organic phase washed with sat. $NaHCO_3$ and brine (75 mL each). The organic phase was dried over $MgSO_4$, filtered and the solvent was removed under reduced pressure to yield a colorless liquid. The product was used in the next step without further purification.

Yield: 2995 mg (12.94 mmol, 92%).

^{1}H NMR (400 MHz, $CDCl_3$): δ 7.49 – 7.43 (m, 2H), 7.29 – 7.23 (m, 2H), 6.60 (d, *J* = 15.7 Hz, 1H), 6.31 (dt, *J* = 15.7, 7.1 Hz, 1H), 4.23 (dd, *J* = 7.1, 1.2 Hz, 3H).

^{13}C NMR (101 MHz, $CDCl_3$): δ 134.98, 133.03, 131.95, 128.35, 125.84, 122.30, 45.27.

Spectral data are in accordance with those reported in the literature.[18]

Step 4: Diethyl *P*[(2*E*)-3-(4-bromophenyl)-2-propenyl]phosphonate (**S13**)

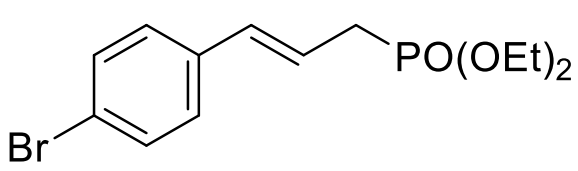

<u>Obtained as a colorless liquid according to GP2</u>:

- 2990 mg, 12.91 mmol, 1.0 equiv 1-[(1*E*)-Chloro-1-propen-1-yl}-4-bromobenzene
- 2880 mg, 17.33 mmol, 1.3 equiv triethyl phosphite
- Purified by flash column chromatography (SiO_2, EtOAc)

Yield: 4026 mg (12.08 mmol, 94%).

^{1}H NMR (400 MHz, $CDCl_3$): δ 7.46 – 7.40 (m, 2H), 7.22 (d, *J* = 8.7 Hz, 2H), 6.46 (dd, *J* = 15.8, 5.2 Hz, 1H), 6.22 – 6.10 (m, 1H), 4.19 – 4.05 (m, 4H), 2.75 (ddd, *J* = 22.4, 7.6, 1.5 Hz, 2H), 1.32 (t, *J* = 7.0 Hz, 6H).

^{13}C NMR (101 MHz, $CDCl_3$): δ 135.9 (d, *J* = 3.6 Hz), 133.7 (d, *J* = 15.0 Hz), 131.8, 127.9 (d, *J* = 2.3 Hz), 121.5 (d, *J* = 1.2 Hz), 119.9 (d, *J* = 11.8 Hz), 62.2 (d, *J* = 6.8 Hz), 31.3 (d, *J* = 139.4 Hz), 16.6 (d, *J* = 5.9 Hz).

^{31}P NMR (162 MHz, $CDCl_3$): δ 27.4.

Spectral data are in accordance with those reported in the literature.[18]

Step5: (1*E*,3*E*)-1,4-Di(4-bromophenyl)-1,3-butadien (**1aa**)

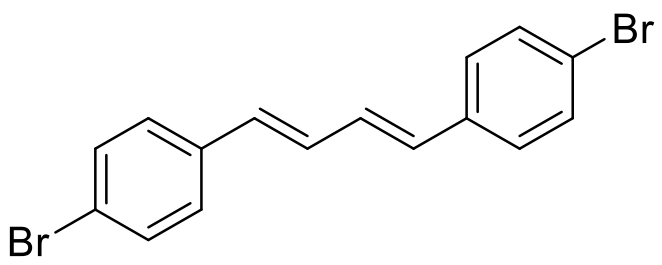

Obtained as a white amorphous solid according to GP1:

- 933 mg, 5.04 mmol, 1.0 equiv 4-bromobenzaldehyde
- 1932 mg, 5.80 mmol, 1.2 equiv diethyl *P*[(2*E*)-3-(4-bromphenyl)-2-propen-1-yl]phosphonate
- 190 mg, 7.13 mmol, 1.41 equiv NaH (90% dry)
- 34 mL anhydrous THF as solvent
- Purified by flash column chromatography (SiO_2, 20% Et_2O in *n*-pentane), followed by crystallization from toluene

Yield: 779 mg (2.14 mmol, 42%).

^{1}H NMR (400 MHz, $CDCl_3$): δ 7.48 – 7.42 (m, 4H), 7.32 – 7.27 (m, 4H), 6.96 – 6.86 (m, 2H), 6.66 – 6.57 (m, 2H).

^{13}C NMR (101 MHz, $CDCl_3$): δ 136.3, 132.2, 132.0, 129.7, 128.0, 121.6.

IR (ATR, cm^{-1}): 1579 (m), 1483 (m), 1397 (m), 1072 (m), 1007 (m), 989 (s), 851 (s), 829 (m), 799 (s), 516 (m), 494 (m).

HRMS (APCI): calc. for $C_{16}H_{13}Br_2^+$ $[M+H]^+$: 362.9379; found: 362.9372.

Spectral data are in accordance with those reported in the literature.[17]

7.2.4 Substituted ethyl (1*E*,3*E*)-1,3-butadienoates (1ab to 1ad)

*7.2.4.1 Ethyl sorbate (**1ab**)*

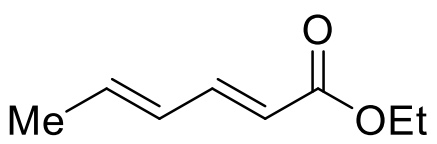

Under an inert gas atmosphere in a round-bottom flask 1124 mg sorbic acid (10.02 mol, 1.0 equiv) was dissolved in 10 mL EtOH and was cooled to 0 °C. Then 0.95 mL thionyl chloride (1556 mg, 13.08 mmol, 1.3 equiv) was added dropwise and the mixture was with an o to 80 °C in an oil bath and stirred for 4 h. After cooling to r.t. 10 mL DCM was added, followed by 10 mL sat. $NaHCO_3$. The mixture was transferred to a separatory funnel with 20 mL DCM, the phases were separated and the organic phase was washed with sat. $NaHCO_3$ (2 x 10 mL), dried over $MgSO_4$, filtered and the solvent removed under reduced pressure. The product was obtained after flash column chromatography (SiO_2, 5% Et_2O in *n*-pentane) as a colorless liquid.

Yield: 1267 mg (9.9:1 d.r., 9.04 mmol, 90%).

^{1}H NMR (400 MHz, CD_2Cl_2): δ 7.29 – 7.18 (m, 1H), 6.28 – 6.10 (m, 2H), 5.80 – 5.73 (m, 1H), 4.15 (q, J = 7.2 Hz, 2H), 1.84 (d, J = 6.4 Hz, 3H), 1.26 (t, J = 7.2 Hz, 3H).

^{13}C NMR (101 MHz, CD_2Cl_2): δ 167.4, 145.1, 139.7, 130.1, 119.5, 60.5, 18.8, 14.5.

IR (ATR, cm^{-1}): 1709 (s), 1645 (m), 1619 (m), 1326 (m), 1259 (m), 1241 (s), 1186 (m), 1136 (s), 1035 (m), 998 (m), 869 (m).

HRMS (ESI): calc. for $C_8H_{12}NaO_2^+$ $[M+Na]^+$: 163.0730; found: 163.0732.

Spectral data are in accordance with those reported in the literature.[19]

*7.2.4.2 Ethyl (2E,4E)-2,4-octadienoate (**1ac**)*

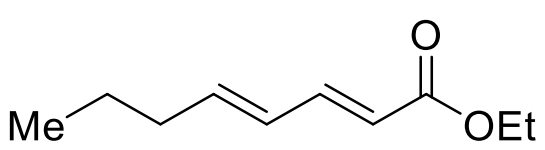

Obtained as a colorless liquid according to GP1:

- 736 mg, 7.50 mmol, 1.0 equiv *trans*-hexan-2-al
- 2588 mg, 11.54 mmol, 1.5 equiv triethyl phosphonoacetate

- 421 mg, 10.52 mmol, 1.4 equiv NaH (60% dispersion in mineral oil)
- 15 mL anhydrous THF as solvent
- Purified by flash column chromatography (SiO_2, 40% DCM in *n*-hexane)

Yield: 836 mg (4.97 mmol, 66%).

^{1}H NMR (400 MHz, CD_2Cl_2): δ 7.29 – 7.19 (m, 1H), 6.26 – 6.09 (m, 2H), 5.77 (d, *J* = 15.9 Hz, 1H), 4.15 (q, *J* = 7.1 Hz, 2H), 2.15 (q, *J* = 6.8 Hz, 2H), 1.46 (sext, *J* = 7.3 Hz, 2H), 1.26 (t, *J* = 7.2 Hz, 3H), 0.92 (t, *J* = 7.3 Hz, 3H).

^{13}C NMR (101 MHz, CD_2Cl_2): δ 167.4, 145.3, 145.0, 128.8, 119.6, 60.5, 35.4, 22.4, 14.5, 13.8.

IR (ATR, cm^{-1}): 1710 (s), 1643 (m), 1302 (m), 1258 (m), 1245 (m), 1218 (m), 1182 (m), 1137 (s), 1039 (m), 998 (m).

HRMS (ESI): calc. for $C_{10}H_{16}NaO_2^+$ $[M+Na]^+$: 191.1043; found: 191.1046.

Spectral data are in accordance with those reported in the literature.[9]

*7.2.4.3 Ethyl (2E,4E)-5-phenyl-2,4-pentadienoate (**1ad**)*

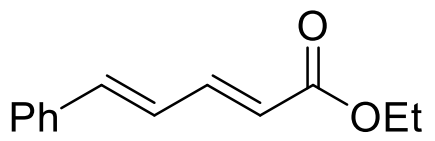

Obtained as a white amorphous solid according to GP1:

- 992 mg, 7.51 mmol, 1.0 equiv *trans*-cinnamaldehyde
- 2138 mg, 9.53 mmol, 1.3 equiv triethyl phosphonoacetate
- 247 mg, 9.26 mmol, 1.2 equiv NaH (90% dry)
- 37.5 mL anhydrous THF as solvent
- Purified by flash column chromatography (SiO_2, 40% DCM in *n*-hexane)

Yield: 1421 mg (7.03 mmol, 94%).

^{1}H NMR (400 MHz, $CDCl_3$): δ 7.49 – 7.41 (m, 3H), 7.40 – 7.28 (m, 3H), 6.94 – 6.82 (m, 2H), 5.99 (d, *J* = 15.3 Hz, 1H), 4.23 (q, *J* = 7.1 Hz, 2H), 1.32 (t, *J* = 7.1 Hz, 3H).

^{13}C NMR (101 MHz, CD_2Cl_2): δ 167.2, 144.7, 140.5, 136.2, 129.2, 129.0, 127.3, 126.4, 121.5, 60.5, 14.5.

IR (ATR, cm^{-1}): 1702 (s), 1624 (s), 1257 (m), 1234 (s), 1174 (m), 1126 (s), 1034 (m), 995 (s), 754 (m), 712 (m), 688 (m).

HRMS (ESI): calc. for $C_{13}H_{14}NaO_2^+$ $[M+Na]^+$: 225.0886; found: 225.0888.

Spectral data are in accordance with those reported in the literature.[20]

7.2.5 Substituted (*E*)-1,3-butadiene (1ae and 1af)

*7.2.5.1 Diethyl P-2-propen-1-ylphosphonate (**S14**)*

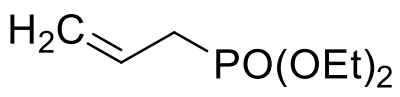

Obtained as a colorless liquid according to GP2:

- 12.1 g, 100.0 mmol, 1.0 equiv allyl bromide
- 16.8 g, 101.1 mmol, 1.0 equiv triethyl phosphite
- Purified by distillation (5.2 Torr, 91 to 94 °C)

Yield: 17.4 g (97.9 mmol, 98%).

^{1}H NMR (400 MHz, $CDCl_3$): δ 5.88 – 5.71 (m, 1H), 5.27 – 5.15 (m, 2H), 4.18 – 4.02 (m, 4H), 2.61 (ddt, *J* = 21.9, 7.4, 1.3 Hz, 2H), 1.32 (t, *J* = 7.1 Hz, 6H).

^{13}C NMR (101 MHz, $CDCl_3$): δ 127.7, 127.6, 120.1, 120.0, 62.1, 62.0, 32.7, 31.3, 16.6, 16.5.

^{31}P NMR (162 MHZ, $CDCl_3$): δ 28.0.

IR (ATR, cm^{-1}): 1250 (m), 1020 (s), 954 (s), 848 (m), 825 (m), 801 (m), 776 (m), 621 (m), 487 (m).

HRMS (ESI): calc. for $C_7H_{15}NaO_3P^+$ [M+Na]$^+$: 201.0651; found: 201.0650.

Spectral data are in accordance with those reported in the literature.[21]

*7.2.5.2 1,1'-Biphenyl-4-(1E)-1,3-butadien-1-yl (**1ae**)*

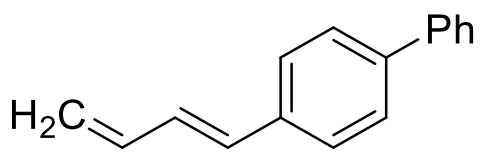

Obtained as a white amorphous solid according to GP1:

- 907 mg, 4.98 mmol, 1.0 equiv Biphenyl-4-carbaldehyd
- 1341 mg, 7.53 mmol, 1.5 equiv diethyl *P*-2-propen-1-ylphosphonate
- 302 mg, 7.55 mmol, 1.5 equiv NaH (60% dispersion in mineral oil)
- 60 mL anhydrous THF as solvent
- Purified by flash column chromatography (SiO_2, *n*-pentane)

Yield: 318 mg (1.54 mmol, 31%).

^{1}H NMR (400 MHz, CD_2Cl_2): δ 7.67 – 7.55 (m, 4H), 7.53 – 7.48 (m, 2H), 7.47 – 7.41 (m, 2H), 7.37 – 7.32 (m, 1H), 6.87 (ddt, *J* = 15.6, 10.5, 0.9 Hz, 1H), 6.66 – 6.49 (m, 2H), 5.41 – 5.34 (m, 1H), 5.23 – 5.17 (m, 2H).

^{13}C NMR (101 MHz, CD_2Cl_2): δ 140.9, 140.6, 137.6, 136.6, 132.7, 130.1, 129.2, 127.8, 127.6, 127.3, 127.2, 118.0.

IR (ATR, cm^{-1}): 1004 (m), 945 (m), 898 (m), 873 (m), 828 (m), 762 (s), 723 (m), 692 (m), 644 (m), 485 (m).

HRMS (APCI): calc. for $C_{16}H_{15}^+$ [M+H]$^+$: 207.1168; found: 207.1162.

Spectral data are in accordance with those reported in the literature.[22]

*7.2.5.2 1-(1E)-1,3-Butadien-1-yl-4-methylbenzene (**1af**)*

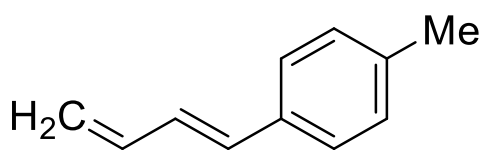

Obtained as a yellow liquid according to GP1:

- 600 mg, 5.0 mmol, 1.0 equiv *p*-tolualdehyde
- 1350 mg, 7.58 mmol, 1.5 equiv diethyl *P*-2-propen-1-ylphosphonate
- 300 mg, 7.50 mmol, 1.5 equiv NaH (60% dispersion in mineral oil)
- 60 mL anhydrous THF as solvent

Purified by flash column chromatography (SiO_2, *n*-pentane)

Yield: 195 mg (1.35 mmol, 27%).

^{1}H NMR (400 MHz, CD_2Cl_2): δ 7.35 – 7.27 (m, 2H), 7.17 – 7.10 (m, 2H), 6.82 – 6.71 (m, 1H), 6.60 – 6.45 (m, 2H), 5.36 – 5.26 (m, 1H), 5.17 – 5.12 (m, 1H), 2.33 (s, 3H).

^{13}C NMR (101 MHz, CD_2Cl_2): δ 138.1, 137.7, 134.7, 133.2, 129.7, 129.0, 126.7, 117.2, 21.3.

IR (ATR, cm^{-1}): 1599 (m), 1510 (m), 1000 (s), 968 (m), 950 (m), 895 (m), 865 (m), 805 (s), 713 (m), 641 (m), 500 (m).

HRMS (APCI): calc. for $C_{11}H_{13}^+$ [M+H]$^+$: 145.1012; found: 145.1013.

Spectral data are in accordance with those reported in the literature.[22]

7.3. Synthesis and Analytical Data of Substituted *p*-Benzoquinones (2)

*7.3.1 2-Cyclopropyl-1,4-benzoquinone (**2d**)*

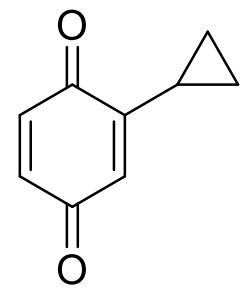

Obtained as a yellow amorphous solid according to GP4:

- 218 mg, 2.02 mmol, 1.0 equiv *p*-benzoquinone **2a**
- 348 mg, 4.05 mmol, 2.0 equiv cyclopropane carboxylic acid
- 68 mg, 0.40 mmol, 0.2 equiv $AgNO_3$
- 915 mg, 4.01 mmol, 2.0 equiv ammonium persulfate
- 10.0 mL DCE and 10.0 mL H_2O as solvents
- Purified by flash column chromatography (SiO_2, 5% Et_2O in *n*-pentane)

Yield: 75 mg (0.51 mmol, 25%).

^{1}H NMR (400 MHz, CD_2Cl_2): δ 6.75 (d, *J* = 10.0 Hz, 1H), 6.66 (dd, *J* = 10.1, 2.5 Hz, 1H), 6.13 (dd, *J* = 2.6, 0.9 Hz, 1H), 2.03 (dddd, *J* = 13.6, 8.3, 5.2, 0.8 Hz, 1H), 1.17 – 1.01 (m, 2H), 0.82 – 0.69 (m, 2H).

^{13}C NMR (101 MHz, CD_2Cl_2): δ 187.8, 187.1, 152.6, 137.1, 136.6, 126.8, 11.1, 9.4.

IR (ATR, cm^{-1}): 1650 (s), 1589 (m), 1403 (m), 1314 (m), 1294 (m), 1087 (m), 1043 (m), 1022 (m), 909 (s), 884 (m), 829 (s), 799 (m), 425 (s).

HRMS (ESI): calc. for $C_9H_9O_2^+$ $[M+H]^+$: 149.0597; found: 149.0603.

*7.3.2 2-Cyclohexyl-1,4-benzoquinone (**2e**)*

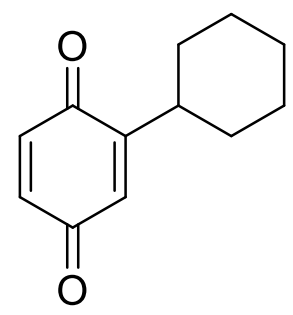

Obtained as a yellow amorphous solid according to GP4:

- 216 mg, 2.00 mmol, 1.0 equiv *p*-benzoquinone **2a**
- 507 mg, 4.02 mmol, 2.0 equiv cyclohexane carboxylic acid
- 69 mg, 0.41 mmol, 0.2 equiv $AgNO_3$
- 923 mg, 4.04 mmol, 2.0 equiv ammonium persulfate
- 10.0 mL DCE and 10.0 mL H_2O as solvents
- Purified by flash column chromatography (SiO_2, 10% Et_2O in *n*-pentane)

Yield: 229 mg (1.21 mmol, 60%).

^{1}H NMR (400 MHz, CD_2Cl_2): δ 6.73 (d, *J* = 10.1 Hz, 1H), 6.67 (dd, *J* = 10.1, 2.4 Hz, 1H), 6.49 (dd, *J* = 2.4, 1.1 Hz, 1H), 2.67 (ttd, *J* = 11.7, 3.1, 1.2 Hz, 1H), 1.87 – 1.69 (m, 5H), 1.47 – 1.33 (m, 2H), 1.29 – 1.12 (m, 3H).

^{13}C NMR (101 MHz, CD_2Cl_2): δ 188.6, 187.5, 154.3, 137.4, 136.3, 131.2, 36.8, 32.4, 26.8, 26.4.

IR (ATR, cm^{-1}): 2931 (m), 2852 (m), 1646 (s), 1596 (m), 1447 (m), 1306 (m), 1296 (m), 1282 (m), 1123 (m), 1071 (m), 994 (m), 907 (m), 886 (m), 832 (m), 424 (m).

HRMS (ESI): calc. for $C_{12}H_{14}NaO_2^+$ $[M+Na]^+$: 213.0886; found: 213.0884.

Spectral data are in accordance with those reported in the literature.[23]

*7.3.3 2-Phenyl-1,4-benzoquinone (**2f**)*

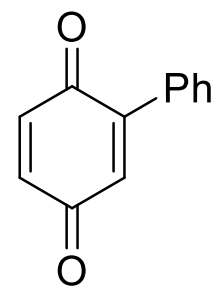

Obtained as a yellow amorphous solid according to GP4:

- 214 mg, 1.98 mmol, 1.0 equiv *p*-benzoquinone **2a**
- 367 mg, 3.01 mmol, 1.5 equiv phenylboronic acid
- 69 mg, 0.40 mmol, 0.2 equiv $AgNO_3$
- 1090 mg, 4.03 mmol, 2.0 equiv potassium persulfate

- 10.0 mL DCM and 10.0 mL H_2O as solvents
- Purified by flash column chromatography (SiO_2, 10% Et_2O in *n*-pentane)

Yield: 293 mg (1.59 mmol, 80%).

^{1}H NMR (400 MHz, CD_2Cl_2): δ 7.52 – 7.42 (m, 5H), 6.90 – 6.79 (m, 3H).

^{13}C NMR (101 MHz, CD_2Cl_2): δ 188.0, 187.1, 146.2, 137.4, 136.7, 133.3, 133.2, 130.4, 129.7, 128.8.

IR (ATR, cm^{-1}): 1642 (s), 1589 (m), 976 (m), 936 (m), 913 (m), 842 (m), 747 (m), 693 (s), 434 (m), 420 (m).

HRMS (ESI): calc. for $C_{12}H_9O_2^+$ $[M+H]^+$: 185.0597; found: 185.0598.

Spectral data are in accordance with those reported in the literature.[24]

*7.3.4 2-Acetamido-1,4-benzoquinone (**2h**)*

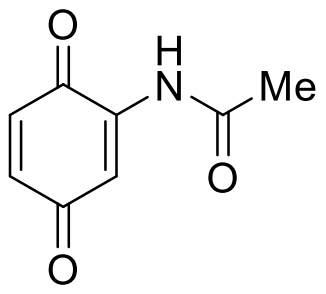

In a round-bottom flask, 758 mg 2,5-dimethoxyaniline (4.95 mol, 1.0 equiv) and 0.84 mL triethylamine (613 mg, 6.06 mmol, 1.2 equiv) were dissolved in 19 mL anhydrous DCM and were cooled to 0 °C. Then 0.43 mL acetyl chloride (471 mg, 6.00 mmol, 1.2 equiv) was added dropwise and the mixture was stirred at r.t. for 1 hour. The mixture was transferred to a separatory funnel, was washed with water (3 x 10 mL), sat. $NaHCO_3$ (10 mL) and brine (10 mL), dried over $MgSO_4$, was filtered and the solvent removed under reduced pressure. The residue was suspended in 25 mL methanol, then 1610 mg PIDA (5.00 mmol, 1.0 equiv) was added and the mixture was stirred until completion. The mixture was transferred to a separatory funnel, diluted with 20 mL water and extracted with Et_2O (3 x 50 mL). The combined organic phases were dried over $MgSO_4$, filtered and the solvent was removed under reduced pressure. The product was obtained after flash column chromatography (SiO_2, 70% Et_2O in *n*-pentane) as a yellow amorphous solid.

Yield: 422 mg (2.56 mmol, 51%).

^{1}H NMR (400 MHz, $CDCl_3$): δ 8.01 (br, 1H), 7.59 (d, *J* = 2.3 Hz, 1H), 6.84 – 6.69 (m, 2H), 2.25 (s, 3H).

^{13}C NMR (101 MHz, CD_2Cl_2): δ 188.1, 182.8, 169.4, 138.4, 133.3, 115.0, 25.1.

IR (ATR, cm^{-1}): 1656 (m), 1505 (m), 1323 (m), 1234 (m), 1202 (m), 1095 (m), 890 (s), 763 (m), 732 (m), 438 (m), 425 (m).

HRMS (ESI): calc. for $C_8H_7NNaO_3^+$ $[M+Na]^+$: 188.0318; found: 188.0321.

Spectral data are in accordance with those reported in the literature.[25]

7.4 Synthesis and Analytical Data of 5,8-Dihydronaphthalene-1,4-dione (4)

*7.4.1 5,8-Dihydro-5,8-diphenylnaphthalene-1,4-dione (**4a**)*

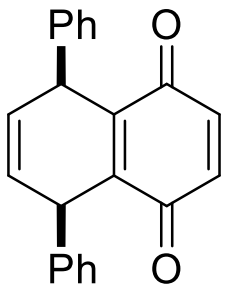

Obtained as a yellow amorphous solid according to GP5 with MsOH:

- 41.9 mg, 0.203 mmol, 1.0 equiv (1*E*,3*E*)-1,4-diphenyl-1,3-butadiene **1a**
- 43.9 mg, 0.406 mmol, 2.0 equiv *p*-benzoquinone **2a**
- 2.64 µL, 3.9 mg, 0.041 mmol, 0.2 equiv MsOH
- 2.0 mL anhydrous DCM as solvent)
- Stirred at r.t. for 90 min
- Purified by flash column chromatography (8 g SiO_2, toluene)

Yield: 46.1 mg (0.148 mmol, 73%).

Obtained as a yellow amorphous solid according to GP6 with TfOH:

- 41.8 mg, 0.203 mmol, 1.0 equiv (1*E*,3*E*)-1,4-diphenyl-1,3-butadiene **1a**
- 88.1 mg, 0.815 mmol, 4.0 equiv *p*-benzoquinone **2a**
- 0.446 µL, 0.8 mg, 0.005 mmol, 2.5 mol% TfOH
- **1a** dissolved in 1.0 mL anhydrous DCM and added *via* syringe pump in 15 min (5 mL/h) to *p*-benzoquinone and TfOH dissolved in 1.0 mL anhydrous DCM at 0 °C
- After complete addition, stirred for another 45 min at r.t.
- Purified by flash column chromatography (8 g SiO_2, toluene)

Yield: 48.5 mg (0.155 mmol, 77%).

Obtained as a yellow amorphous solid according to GP6 on a 10 mmol scale:

- 2090 mg, 10.1 mmol, 1.0 equiv (1*E*,3*E*)-1,4-diphenyl-1,3-butadiene **1a**
- 4385 mg, 40.6 mmol, 4.0 equiv *p*-benzoquinone **2a**
- 22.2 µL, 38.0 mg, 0.25 mmol, 2.5 mol% TfOH
- **1a** dissolved in 50 mL anhydrous DCM and added *via* syringe pump in 15 min (200 mL/h) to *p*-benzoquinone and TfOH dissolved in 50 mL anhydrous DCM at 0 °C
- After complete addition, stirred for another 45 minutes at r.t.
- After removal of the solvent *p*-benzoquinone was removed by sublimation at 60 °C and 1 Torr to recover 1158 mg *p*-benzoquinone
- Purified by flash column chromatography (220 g SiO_2, toluene)

Yield: 2393 mg (7.66 mmol, 76%).

Melting point: 179 – 180 °C.

***R*f** (toluene): 0.29.

^{1}H NMR (400 MHz, CD_2Cl_2): δ 7.34 – 7.28 (m, 8H), 7.27 – 7.20 (m, 2H), 6.68 (s, 2H), 5.90 (d, *J* = 2.8 Hz, 2H), 4.75 (d, *J* = 2.8 Hz, 2H).

^{13}C NMR (101 MHz, CD_2Cl_2): δ 186.8, 142.4, 142.4, 136.8, 129.0, 128.8, 127.2, 126.9, 41.1.

IR (ATR, cm^{-1}): 1649 (m), 1599 (m), 1297 (m), 1117 (m), 1028 (m), 866 (s), 772 (m), 743 (s), 698 (s), 687 (s), 499 (s).

HRMS (ESI): calc. for $C_{22}H_{16}NaO_2^+$ $[M+Na]^+$: 335.1043; found: 335.1041.

*7.4.2 5,8-Dihydro-5-(4-biphenyl)-8-phenylnaphthalene-1,4-dione (**4b**)*

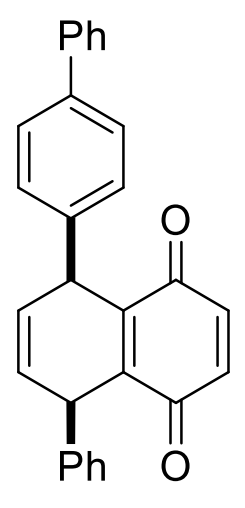

Obtained as a yellow amorphous solid according to GP5 with MsOH:

- 56.6 mg, 0.200 mmol, 1.0 equiv 4-[(1*E*,3*E*)-4-phenyl-1,3-butadien-1-yl]-1,1'-biphenyl **1b**
- 54.5 mg, 0.504 mmol, 2.5 equiv *p*-benzoquinone
- 2.60 µL, 3.9 mg, 0.040 mmol, 0.2 equiv MsOH
- 2.0 mL anhydrous DCM as solvent)
- Stirred at r.t. for 120 min
- Purified by flash column chromatography (8 g SiO_2, 10% cyclohexane in toluene)

Yield: 55.9 mg (0.144 mmol, 72%).

Melting point: 92 – 94 °C.

***R*f** (toluene): 0.33.

^{1}H NMR (400 MHz, CD_2Cl_2): δ 7.63 – 7.53 (m, 4H), 7.46 – 7.29 (m, 9H), 7.27 – 7.21 (m, 1H), 6.70 (s, 2H), 5.99 – 5.90 (m, 2H), 4.84 – 4.74 (m, 2H).

^{13}C NMR (101 MHz, CD_2Cl_2): δ 186.8, 186.8, 142.4, 142.3, 141.5, 141.0, 140.1, 136.9, 136.8, 129.3, 129.2, 129.1, 128.8, 127.7, 127.7, 127.4, 127.2, 127.2, 126.8, 41.1, 40.8.

IR (ATR, cm^{-1}): 1652 (s), 1485 (m), 1290 (m), 1112 (m), 1028 (m), 859 (m), 836 (m), 755 (m), 696 (s), 498 (m).

HRMS (ESI): calc. for $C_{28}H_{20}NaO_2^+$ $[M+Na]^+$: 411.1356; found: 411.1361.

*7.4.3 5,8-Dihydro-5-(2-methylphenyl)-8-phenylnaphthalene-1,4-dione (**4c**)*

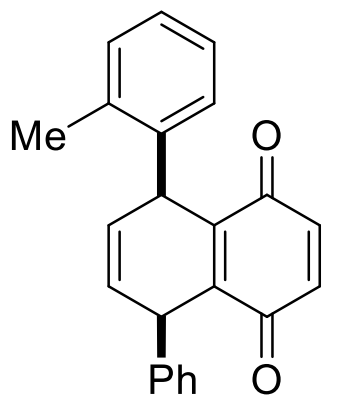

Obtained as a yellow amorphous solid according to GP5 with MsOH:

- 44.5 mg, 0.202 mmol, 1.0 equiv 1-methyl-2-[(1*E*,3*E*)-4-phenyl-1,3-butadien-1-yl]benzene **1c**
- 54.7 mg, 0.506 mmol, 2.5 equiv *p*-benzoquinone
- 2.62 µL, 3.9 mg, 0.040 mmol, 0.2 equiv MsOH
- 2.0 mL anhydrous DCM as solvent
- Stirred at r.t. for 98 min
- Purified by flash column chromatography (8 g SiO_2, toluene)

Yield: 42.6 mg (0.131 mmol, 65%).

Melting point: 180 – 182 °C.

***R*f** (toluene): 0.36.

^{1}H NMR (600 MHz, CD_2Cl_2): δ 7.41 – 7.38 (m, 2H), 7.34 – 7.29 (m, 2H), 7.26 – 7.21 (m, 2H), 7.13 (td, *J* = 7.4, 1.6 Hz, 1H), 7.09 (td, *J* = 7.4, 1.0 Hz, 1H), 7.00 (dd, *J* = 7.6, 1.6 Hz, 1H), 6.69 (d, *J* = 10.0 Hz, 1H), 6.64 (d, *J* = 10.1 Hz, 1H), 5,89 (ddd, *J* = 9.9, 3.8, 1.5 Hz, 1H), 5.81 (ddd, *J* = 10.0, 3.9, 1.6 Hz, 1H), 4.89 (ddd, *J* = 6.8, 3.8, 1.6 Hz, 1H), 4.75 (ddd, *J* = 6.7, 3.9, 1.5 Hz, 1H), 2.59 (s, 3H).

^{13}C NMR (151 MHz, CD_2Cl_2): δ 186.9, 186.8, 143.0, 142.7, 142.4, 141.5, 136.9, 136.6, 136.1, 131.2, 129.3, 128.9, 127.4, 127.2, 127.0, 126.7, 126.3, 125.7, 40.9, 37.7, 19.8.

IR (ATR, cm^{-1}): 1650 (s), 1600 (m), 1297 (m), 863 (m), 770 (m), 741 (m), 698 (m), 688 (m), 507 (m), 467 (m).

HRMS (ESI): calc. for $C_{23}H_{18}NaO_2^+$ $[M+Na]^+$: 349.1199; found: 349.1203.

*7.4.4 5,8-Diyhdro-5-(3-methylphenyl)-8-phenylnaphthalene-1,4-dione (**4d**)*

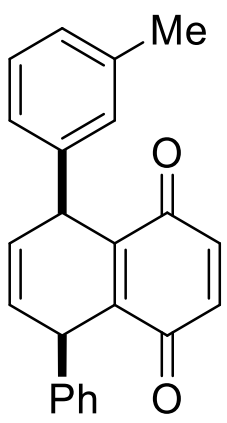

Obtained as a yellow amorphous solid according to GP5 with MsOH:

- 44.3 mg, 0.201 mmol, 1.0 equiv 1-methyl-3-[(1*E*,3*E*)-4-phenyl-1,3-butadien-1-yl]benzene **1d**
- 54.5 mg, 0.504 mmol, 2.5 equiv *p*-benzoquinone
- 2.60 µL, 3.9 mg, 0.040 mmol, 0.2 equiv MsOH
- 2.0 mL anhydrous DCM as solvent
- Stirred at r.t. for 120 min
- Purified by flash column chromatography (8 g SiO_2, toluene)

Yield: 52.2 mg (0.160 mmol, 80%).

Melting point: 142 – 144 °C.

***R*f** (toluene): 0.29.

^{1}H NMR (400 MHz, CD_2Cl_2): δ 7.35 – 7.27 (m, 4H), 7.26 – 7.16 (m, 2H), 7.14 – 7.07 (m, 2H), 7.06 – 7.01 (m, 1H), 6.68 (s, 2 H), 5.94 – 5.85 (m, 2H), 4.78 – 4.73 (m, 1H), 4.72 – 4.68 (m, 1H), 2.28 (s, 3H).

^{13}C NMR (101 MHz, CD_2Cl_2): δ 186.8, 186.8, 142.5, 142.4, 142.2, 138.8, 136.8, 129.5, 128.9, 128.9, 127.9, 127.2, 127.2, 126.8, 125.8, 41.0, 21.5.

IR (ATR, cm^{-1}): 1651 (s), 1296 (m), 860 (m), 778 (m), 765 (m), 750 (m), 700 (m), 682 (m), 510 (m), 441 (m).

HRMS (ESI): calc. for $C_{23}H_{18}NaO_2^+$ $[M+Na]^+$: 349.1199; found: 349.1204.

*7.4.5 5,8-Dihydro-5-(4-methylphenyl)-8-phenylnaphthalene-1,4-dione **(4e)***

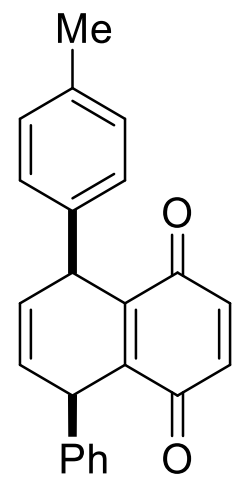

Obtained as a yellow amorphous solid according to GP5 with MsOH:

- 44.5 mg, 0.202 mmol, 1.0 equiv 1-methyl-4-[(1*E*,3*E*)-4-phenyl-1,3-butadien-1-yl]benzene **1e**
- 54.8 mg, 0.507 mmol, 2.5 equiv *p*-benzoquinone
- 2.62 µL, 3.9 mg, 0.040 mmol, 0.2 equiv MsOH
- 2.0 mL anhydrous DCM as solvent
- Stirred at r.t. for 120 min
- Purified by flash column chromatography (8 g SiO_2, toluene)

Yield: 46.7 mg (0.143 mmol, 71%).

Melting point: 164 – 167 °C.

***R*f** (toluene): 0.29.

^{1}H NMR (400 MHz, CD_2Cl_2): δ 7.33 – 7.27 (m, 4H), 7.26 – 7.21 (m, 1H), 7.21 – 7.16 (m, 2H), 7.15 – 7.09 (m, 2H), 6.66 (s, 2H), 5.91 – 5.84 (m, 2H), 4.76 – 4.68 (m, 1H) 2.31 (s, 3H).

^{13}C NMR (101 MHz, CD_2Cl_2): δ 186.8, 142.5, 142.5, 142.2, 139.3, 137.0, 136.8, 136.8, 129.7, 128.99, 128.8, 128.7, 127.1, 127.1, 126.8, 41.1, 40.7, 21.1.

IR (ATR, cm^{-1}): 1649 (s), 1598 (m), 1296 (m), 1116 (m), 1024 (m), 866 (m), 824 (m), 813 (m), 738 (m), 699 (m), 685 (m), 501 (s).

HRMS (ESI): calc. for $C_{23}H_{28}NaO_2^+$ $[M+Na]^+$: 349.1199; found: 349.1203.

*7.4.6 5,8-Dihydro-5-(4-butylphenyl)-8-phenylnaphthalene-1,4-dione **(4f)***

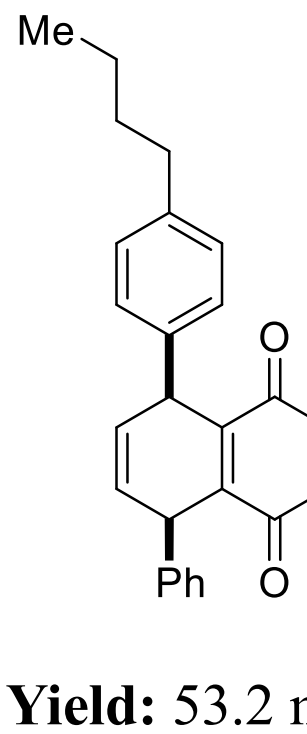

Obtained as a yellow amorphous solid according to GP5 with MsOH:

- 52.9 mg, 0.202 mmol, 1.0 equiv 1-butyl-4-[(1*E*,3*E*)-4-phenyl-1,3-butadien-1-yl]benzene **1f**
- 54.9 mg, 0.508 mmol, 2.5 equiv *p*-benzoquinone **2a**
- 2.62 µL, 3.9 mg, 0.040 mmol, 0.2 equiv MsOH
- 2.0 mL anhydrous DCM as solvent
- Stirred at r.t. for 120 min
- Purified by flash column chromatography (8 g SiO_2, toluene)

Yield: 53.2 mg (0.144 mmol, 71%).

Melting point: 110 – 112 °C.

***R*f** (20% Et_2O in *n*-pentane): 0.28; ***R*f** (toluene): 0.38.

^{1}H NMR (400 MHz, CD_2Cl_2): δ 7.34 – 7.28 (m, 4H), 7.26 -7.17 (m, 3 H), 7.14 – 7.09 (m, 2H), 6.67 (s, 2H), 5.92 – 5.85 (m, 2H), 4.77 – 4.68 (m, 2H), 2.58 (t, *J* = 7.8 Hz, 2H), 1.62 – 1.53 (m, 2H), 1.35 (dq, *J* = 14.6, 7.3 Hz, 2H), 0.93 (t, *J* = 7.3 Hz, 3H).

^{13}C NMR (101 MHz, CD_2Cl_2): δ 186.8, 186.8, 142.6, 142.5, 142.2, 142.0, 139.5, 136.9, 136.8, 129.0, 129.0, 128.8, 128.7, 127.1, 127.1, 126.8, 41.1, 40.7, 35.6, 34.1, 22.9, 14.1.

IR (ATR, cm^{-1}): 1651 (s), 1597 (m), 1292 (m), 1117 (m), 864 (m), 832 (m), 770 (m9,752 (m), 703 (m), 685 (m), 510 (m), 492 (m).

HRMS (ESI): calc. for $C_{26}H_{24}NaO_2^+$ $[M+Na]^+$: 391.1669; found: 391.1667.

*7.4.7 5,8-Dihydro-5-(4-tert-butylphenyl)-8-phenylnaphthalene-1,4-dione (**4g**)*

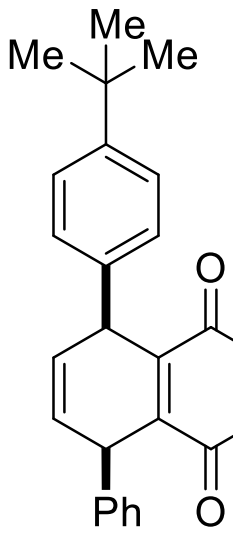

Obtained as a yellow amorphous solid according to GP5 with MsOH:

- 53.1 mg, 0.202 mmol, 1.0 equiv 1-*tert*-butyl-4-[(1*E*,3*E*)-4-phenyl-1,3-butadien-1-yl]benzene **1g**
- 45.9 mg, 0.435 mmol, 2.1 equiv *p*-benzoquinone **2a**
- 2.60 µL, 3.9 mg, 0.040 mmol, 0.2 equiv MsOH
- 2.0 mL anhydrous DCM as solvent
- Stirred at r.t. for 70 min
- Purified by flash column chromatography (8 g SiO_2, toluene)

Yield: 53.7 mg (0.146 mmol, 72%).

Melting point: 145 – 147 °C.

***R*f** (toluene): 0.34.

^{1}H NMR (400 MHz, CD_2Cl_2): δ 7.37 – 7.29 (m, 6H), 7.27 – 7.20 (m, 3 H), 6.67 (s, 2H), 5.88 (d, *J* = 2.7 Hz, 2H), 4.76 – 4.69 (m, 2H), 1.30 (s, 9H).

^{13}C NMR (101 MHz, CD_2Cl_2): δ 186.9, 186.8, 150.1, 142.5, 142.2, 139.3, 136.9, 136.8, 129.0, 128.8, 128.4, 127.1, 127.1, 126.8, 126.0, 41.1, 40.6, 34.7, 31.5.

IR (ATR, cm^{-1}):1652 (s), 1599 (m), 1295 (m), 80 (m), 834 (m), 745 (m), 702 (m), 676 (m), 573 (m), 536 (m), 487 (m).

HRMS (ESI): calc. for $C_{26}H_{24}NaO_2^+$ $[M+Na]^+$: 391.1669; found: 391.1673.

*7.4.8 5,8-Dihydro-5-(2-methoxyphenyl)-8-phenylnaphthalene-1,4-dione (**4h**)*

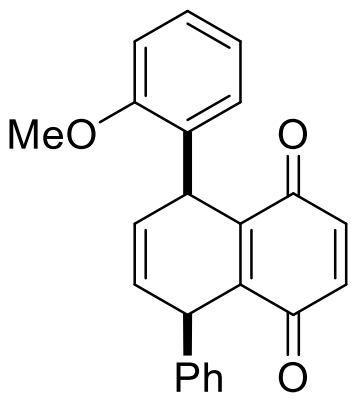

Obtained as an orange amorphous solid according to GP6 with MsOH:

- 47.9 mg, 0.203 mmol, 1.0 equiv 1-methoxy-2-[(1*E*,3*E*)-4-phenyl-1,3-butadien-1-yl]benzene **1h**
- 46.2 mg, 0.427 mmol, 2.1 equiv *p*-benzoquinone **2a**
- 2.62 µL, 3.9 mg, 0.040 mmol, 0.2 equiv MsOH
- **1e** dissolved in 1 mL anhydrous DCM and added *via* syringe pump in 15 min (4 mL/h) to *p*-benzoquinone and MsOH dissolved in 1 mL anhydrous DCM at r.t.
- After complete addition, stirred for another 60 min
- Purified by flash column chromatography (9 g SiO_2, toluene)

Yield: 32.6 mg (0.095 mmol, 47%).

Melting point: 194 – 196 °C.

***R*f** (toluene): 0.19.

^{1}H NMR (400 MHz, CD_2Cl_2): δ 7.38 – 7.31 (m, 2H), 7.30 – 7.16 (m, 4H), 6.98 (d, *J* = 8.2 Hz, 1H), 6.91 (dd, *J* = 7.6, 1.9 Hz, 1H), 6.86 (td, *J* = 7.3, 1.1 Hz, 1H), 6.71 – 6.63 (m, 2H), 6.07 (ddd, *J* = 9.9, 4.0, 1.3

Hz, 1H), 5.76 (ddd, J = 10.0, 4.2, 1.6 Hz, 1H), 5.08 (ddd, J = 6.0, 3.9, 1.5 Hz, 1H), 4.71 (ddd, J = 6.1, 4.0, 1.5 Hz, 1H), 3.94 (s, 3H).

^{13}C NMR (101 MHz, CD_2Cl_2): δ 186.8, 186.7, 143.8, 142.3, 142.0, 137.0, 136.7, 131.4, 129.5, 128.8, 128.2, 127.8, 127.2, 126.2, 126.0, 121.0, 111.3, 55.9, 41.0, 35.4.

IR (ATR, cm^{-1}): 1651 (s), 1596 (m), 1489 (m), 1293 (m), 1244 (m), 1106 (m), 1027 (m), 860 (m), 755 (s), 729 (m), 704 (m), 688 (m), 495 (m), 486 (m).

HRMS (ESI): calc. for $C_{23}H_{18}NaO_3^+$ $[M+Na]^+$: 365.1148; found: 365.1155.

*7.4.9 5,8-Dihydro-5-(3-methoxyphenyl)-8-phenylnaphthalene-1,4-dione (**4i**)*

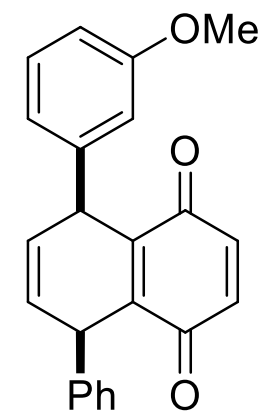

Obtained as an orange amorphous solid according to GP6 with MsOH:

- 48.2 mg, 0.204 mmol, 1.0 equiv 1-methoxy-3-[(1*E*,3*E*)-4-phenyl-1,3-butadien-1-yl]benzene **1i**
- 47.1 mg, 0.436 mmol, 2.1 equiv *p*-benzoquinone **2a**
- 2.64 µL, 3.9 mg, 0.040 mmol, 0.2 equiv MsOH
- **1e** dissolved in 1 mL anhydrous DCM and added *via* syringe pump in 30 min (2 mL/h) to *p*-benzoquinone and MsOH dissolved in 1 mL anhydrous DCM at r.t.
- After complete addition, stirred for another 10 min
- Purified by flash column chromatography (9 g SiO_2, 0.5% EtOAc in toluene)

Yield: 39.8 mg (0.116 mmol, 57%).

Melting point: 167 – 169 °C.

R_f (toluene): 0.11.

^{1}H NMR (400 MHz, CD_2Cl_2): δ 7.34 – 7.27 (m, 4H), 7.22 (s, 2H), 6.90 (dt, J = 7.6, 1.4 Hz, 1H), 6.81 (t, J = 2.1 Hz, 1H), 6.77 (ddd, J = 8.2, 2.6, 0.9 Hz, 1H), 6.68 (s, 2H), 5.95 – 5.76 (m, 2H), 4.79 – 4.68 (m, 2H), 3.72 (s, 3H).

^{13}C NMR (101 MHz, CD_2Cl_2): δ 186.8, 186.7, 160.4, 143.9, 142.4, 142.4, 142.3, 130.0, 129.0, 128.9, 127.2, 127.0, 126.9, 121.2, 114.2, 112.8, 55.5, 41.1, 41.1.

IR (ATR, cm^{-1}): 1651 (s), 1562 (m), 1296 (m), 1248 (m), 1156 (m), 1115 (m), 860 (m), 843 (m), 797 (m), 781 (m), 768 (m), 754 (m), 741 (m), 699 (m), 682 (m), 483 (m).

HRMS (ESI): calc. for $C_{23}H_{18}NaO_3^+$ $[M+Na]^+$: 365.1148; found: 365.1146.

*7.4.10 5,8-Dihydro-5-(4-methoxyphenyl)-8-phenylnaphthalene-1,4-dione (**4j**)*

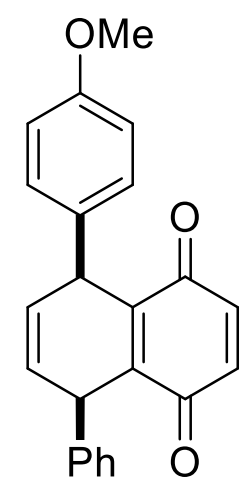

Obtained as an orange amorphous solid according to GP6 with MsOH:

- 47.4 mg, 0.200 mmol, 1.0 equiv 1-methoxy-4-[(1*E*,3*E*)-4-phenyl-1,3-butadien-1-yl]benzene **1j**
- 46.2 mg, 0.427 mmol, 2.1 equiv *p*-benzoquinone **2a**
- 2.60 µL, 3.9 mg, 0.040 mmol, 0.2 equiv MsOH
- **1e** dissolved in 2 mL anhydrous DCM and added *via* syringe pump in 30 min (4 mL/h) to *p*-benzoquinone and MsOH dissolved in 0.75 mL anhydrous DCM at r.t.
- After complete addition, stirred for another 10 min
- Purified by flash column chromatography (10 g SiO_2, toluene)

Yield: 33.7 mg (0.098 mmol, 49%).

Melting point: 143 – 145 °C.

***R*f** (toluene): 0.10.

¹H NMR (400 MHz, CD_2Cl_2): δ 7.35 – 7.26 (m, 4H), 7.26 – 7.19 (m, 3H), 6.86 – 6.81 (m, 2H), 6.66 (s, 2H), 5.94 – 5.82 (m, 2H), 4.77 – 4.66 (m, 2H), 3.77 (s, 3H).

¹³C NMR (101 MHz, CD_2Cl_2): δ 186.9, 186.9, 159.0, 142.7, 142.6, 141.9, 136.9, 136.8, 134.2, 130.0, 129.0, 128.7, 127.1, 127.1, 126.9, 114.3, 55.6, 41.1, 40.2.

IR (ATR, cm^{-1}): 1651 (s), 1509 8m), 1289 (m), 1248 (m), 1032 (m), 863 (m), 826 (m), 809 (m), 764 (m), 702 (m), 685 (m).

HRMS (ESI): calc. for $C_{23}H_{18}NaO_3^+$ $[M+Na]^+$: 365.1148; found: 365.1154.

*7.4.11 5,8-Dihydro-5-(1-naphthyl)-8-phenylnaphthalene-1,4-dione (**4k**)*

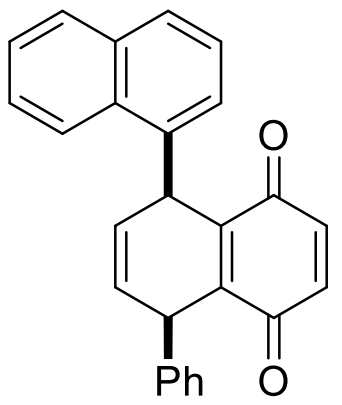

Obtained as an orange amorphous solid according to GP5 with MsOH:

- 51.4 mg, 0.200 mmol, 1.0 equiv 1-[(1*E*,3*E*)-4-phenyl-1,3-butadien-1-yl]naphthalene **1k**
- 54.9 mg, 0.508 mmol, 2.5 equiv *p*-benzoquinone **2a**
- 2.60 µL, 3.9 mg, 0.040 mmol, 0.2 equiv MsOH
- 2.0 mL anhydrous DCM as solvent
- Stirred at r.t. for 24 h
- Purified by flash column chromatography (9 g SiO_2, toluene)

Yield: 39.9 mg (0.110 mmol, 55%).

Melting point: 196 – 198 °C.

***R*f** (toluene): 0.29.

¹H NMR (400 MHz, CD_2Cl_2): δ 8.36 (d, *J* = 8.4 Hz, 1H), 7.94 (d, *J* = 9.8 Hz, 1H), 7.78 (d, *J* = 8.2 Hz, 1H), 7.71 – 7.63 (m, 1H), 7.61 – 7.55 (m, 1H), 7.43 – 7.35 (m, 3H), 7.33 – 7.26 (m, 2H), 7.26 – 7.19 (m, 1H), 7.18 – 7.13 (m, 1H), 6.74 (d, *J* = 10.1 Hz, 1H), 6.67 (d, *J* = 10.0 Hz, 1H), 6.18 (ddd, *J* = 9.9,4.0, 1.5 Hz, 1H), 5.83 (ddd, *J* = 9.9, 4.1, 1.6 Hz, 1H), 5.57 – 5.50 (m, 1H), 4.85 – 4.77 (m, 1H).

¹³C NMR (101 MHz, CD_2Cl_2): δ 186.7, 186.7, 143.7, 142.2, 141.9, 139.4, 137.1, 136.7, 134.6, 131.3, 129.6, 129.4, 128.9, 127.8, 127.3, 127.0, 126.4, 126.0, 125.9, 124.9, 123.5, 41.1, 36.9.

IR (ATR, cm^{-1}): 1651 (s), 1295 (m), 860 (m), 790 (m), 768 (m), 704 (m), 684 (m), 457 (m), 431 (m).

HRMS (ESI): calc. for $C_{26}H_{18}NaO_2^+$ $[M+Na]^+$: 385.1199; found: 385.1197.

*7.4.12 5,8-Dihydro-5-(2-naphthyl)-8-phenylnaphthalene-1,4-dione (**4l**)*

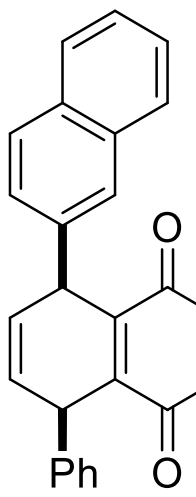

Obtained as an orange amorphous solid according to GP5 with MsOH:

- 51.4 mg, 0.201 mmol, 1.0 equiv 2-[(1*E*,3*E*)-4-phenyl-1,3-butadien-1-yl]naphthalene **1l**
- 45.9 mg, 0.425 mmol, 2.1 equiv *p*-benzoquinone **2a**
- 2.60 µL, 3.9 mg, 0.040 mmol, 0.2 equiv MsOH
- 2.0 mL anhydrous DCM as solvent
- Stirred at r.t. for 24 h
- Purified by flash column chromatography (8 g SiO_2, 10% cyclohexane in toluene)

Yield: 52.8 mg (0.146 mmol, 73%).

Melting point: 89 – 92 °C.

***R*f** (toluene): 0.33.

^{1}**H NMR** (400 MHz, CD_2Cl_2): δ 7.85 – 7.78 (m, 2H), 7.73 – 7.66 (m, 2H), 7.52 (dd, *J* = 8.5, 1.9 Hz, 1H), 7.48 – 7.42 (m, 2H), 7.38 – 7.29 (m, 4H), 7.28 – 7.22 (m, 1H), 6.73 – 6.65 (m, 2H), 6.03 – 5.95 (m, 2H), 4.92 (dd, *J* = 5.9, 3.1 Hz, 1H), 4.81 (dd, *J* = 5.9 Hz, *J* = 3.1 Hz, 1H).

^{13}**C NMR** (101 MHz, CD_2Cl_2): δ 186.8, 186.8, 142.5, 142.4, 142.3, 139.8, 136.9, 136.8, 133.9, 132.8, 129.0, 128.9, 128.6, 128.0, 127.9, 127.5, 127.4, 127.2, 126.9, 126.6, 126.2, 41.2, 41.1.

IR (ATR, cm^{-1}): 1651 (m), 1598 (m), 1289 (m), 857 (m), 816 (m), 751 (m), 699 (m), 477 (m).

HRMS (ESI): calc. for $C_{26}H_{18}NaO_2^+$ $[M+Na]^+$: 385.1199; found: 385.1202.

*7.4.13 5,8-Dihydro-5-phenyl-8-(1-pyrenyl)naphthalene-1,4-dione (**4m**)*

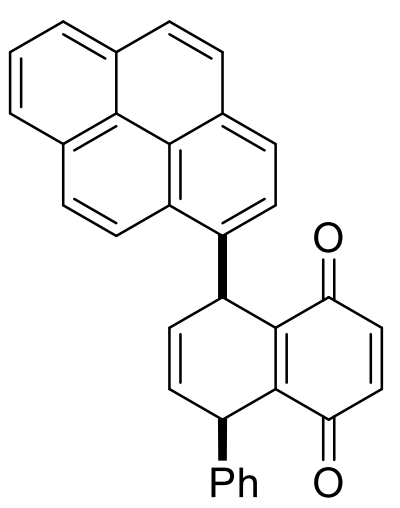

Obtained as an orange amorphous solid according to GP5 with MsOH:

- 66.9 mg, 0.203 mmol, 1.0 equiv 1-[(1*E*,3*E*)-4-phenyl-1,3-butadien-1-yl]pyrene **1m**
- 55.2 mg, 0.511 mmol, 2.5 equiv *p*-benzoquinone **2a**
- 2.62 µL, 3.9 mg, 0.040 mmol, 0.2 equiv MsOH
- 3.0 mL anhydrous DCE as solvent
- Stirred at 80 °C for 24 h
- Purified by flash column chromatography (8 g SiO_2, 20% cyclohexane in toluene)

Yield: 17.1 mg (0.039 mmol, 19%).

Melting point: 193 – 195 °C.

R

$\boldsymbol{R_f}$ (toluene): 0.29.

^{1}**H NMR** (400 MHz, CD_2Cl_2): δ 8.61 (d, *J* = 9.7 Hz, 1H), 8.32 – 8.19 (m, 3H), 8.17 – 8.00 (m, 4H), 7.73 (d, *J* = 7.9 Hz, 1H), 7.49 – 7.40 (m, 2H), 7.38 – 7.29 (m, 2H), 7.29 – 7.21 (m, 1H), 6.75 (d, *J* = 10.0 Hz, 1H), 6.66 (d, *J* = 10.0 Hz, 1H), 6.21 (ddd, *J* = 9.8, 4.0, 1.5 Hz, 1H), 5.87 (ddd, *J* = 9.9, 4.0, 1.6 Hz, 1H), 5.83 – 5.78 (m, 1H), 4.89 – 4.81 (m, 1H).

^{13}**C NMR** (101 MHz, CD_2Cl_2): δ 186.8, 186.7, 143.7, 142.3, 142.1, 137.1, 136.8, 136.7, 131.3, 130.6, 129.5, 128.9, 128.5, 128.4, 127.7, 127.6, 127.3, 126.7, 126.6, 126.4, 125.8, 125.7, 125.5, 125.5, 125.3, 123.1, 41.1, 37.5.

IR (ATR, cm^{-1}): 1651 (s), 1599 (m), 1291 (m), 1259 (m), 1111 (m), 1027 (m), 839 (s), 816 (m), 755 (m), 715 (m), 699 (m), 685 (m), 497 (m).

HRMS (ESI): calc. for $C_{32}H_{20}NaO_2^+$ $[M+Na]^+$: 459.1356; found: 459.1357.

*7.4.14 5,8-Dihydro-5-(2-chlorophenyl)-8-phenylnaphthalene-1,4-dione (**4n**)*

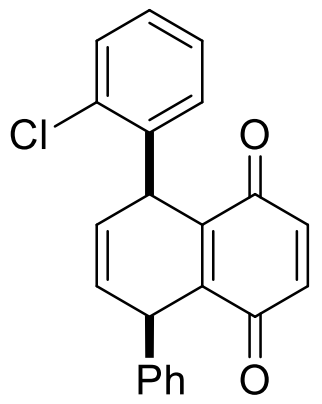

Obtained as an orange amorphous solid according to GP6 with TfOH:

- 48.6 mg, 0.202 mmol, 1.0 equiv 1-chloro-2-[(1*E*,3*E*)-4-phenyl-1,3-butadien-1-yl]benzene **1n**
- 87.5 mg, 0.809 mmol, 4.0 equiv *p*-benzoquinone **2a**
- 0.44 µL, 0.8 mg, 0.005 mmol, 2.5 mol% TfOH
- **1n** dissolved in 1.5 mL anhydrous DCM and added *via* syringe pump in 15 min (6 mL/h) to *p*-benzoquinone and TfOH dissolved in 0.75 mL anhydrous DCM at 0 °C
- After complete addition, stirred for another 2 h at r.t.
- Purified by flash column chromatography (10 g SiO_2, toluene)

Yield: 61.6 mg (0.178 mmol, 88%).

Melting point: 182 – 183 °C.

R_f (toluene): 0.26.

^{1}H NMR (400 MHz, CD_2Cl_2): δ 7.49 – 7.45 (m, 1H), 7.38 – 7.33 (m, 2H), 7.32 – 7.26 (m, 2H), 7.25 – 7.16 (m, 3H), 7.07 – 7.01 (m, 1H), 6.69 (q, *J* = 9.6 Hz, 2H), 6.07 (ddd, *J* = 9.9, 4.0, 1.5 Hz, 1H), 5.84 (ddd, *J* = 10.0, 4.1, 1.7 Hz, 1H), 5.16 (ddd, *J* = 6.6, 3.9, 1.6 Hz, 1H), 4.79 – 4.73 (m, 1H).

^{13}C NMR (101 MHz, CD_2Cl_2): δ 186.6, 186.4, 144.0, 141.9, 141.2, 140.5, 137.1, 136.6, 133.7, 130.4, 129.4, 128.9, 128.7, 128.4, 127.7, 127.3, 126.9, 124.9, 40.9, 38.4.

IR (ATR, cm^{-1}): 1650 (s), 1598 (m), 1467 (m), 1452 (m), 1438 (m), 1298 (m), 1118 (m), 1029 (m), 860 (s), 705 (s), 687 (s), 507 (m), 488 (m), 456 (m).

HRMS (ESI): calc. for $C_{22}H_{15}ClNaO_2^+$ $[M+Na]^+$: 369.0653; found: 369.0648.

*7.4.15 5,8-Dihydro-5-(4-chlorophenyl)-8-phenylnaphthalene-1,4-dione (**4o**)*

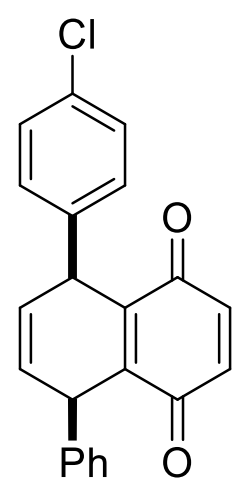

Obtained as an orange amorphous solid according to GP6 with TfOH:

- 48.5 mg, 0.202 mmol, 1.0 equiv 1-chloro-4-[(1*E*,3*E*)-4-phenyl-1,3-butadien-1-yl]benzene **1o**
- 87.4 mg, 0.809 mmol, 4.0 equiv *p*-benzoquinone **2a**
- 0.44 µL, 0.8 mg, 0.005 mmol, 2.5 mol% TfOH
- **1o** dissolved in 1.5 mL anhydrous DCM and added *via* syringe pump in 15 min (6 mL/h) to *p*-benzoquinone and TfOH dissolved in 0.75 mL anhydrous DCM at 0 °C
- After complete addition, stirred for another 60 min at r.t.
- Purified by flash column chromatography (10 g SiO_2, 20% cyclohexane in toluene)

Yield: 62.7 mg (0.181 mmol, 89%).

Melting point: 75 – 77 °C.

R_f (toluene): 0.28.

^{1}H NMR (400 MHz, CD_2Cl_2): δ 7.35 – 7.20 (m, 9H), 6.68 (s, 2H), 5.97 – 5.92 (m, 1H), 5.89 – 5.83 (m, 1H) ,4.78 – 4.70 (m, 2H)

^{13}C NMR (101 MHz, CD_2Cl_2): δ 186.7, 142.4, 142.2, 142.0, 140.8, 136.9, 136.8, 132.9, 130.4, 129.1, 129.0, 128.7, 127.6, 127.2, 126.4, 41.1, 40.5.

IR (ATR, cm^{-1}): 1652 (s), 1599 (m), 1487 (m),1290 (m), 1179 (w), 1112 (m), 1091 (m), 1028 (m), 1014 (m), 860 (s), 829 (m), 748 (m), 699 (m), 676 (m), 501 (m)

HRMS (ESI): calc. for $C_{22}H_{15}ClNaO_2^+$ $[M+Na]^+$: 369.0653; found: 369.0658.

*7.4.16 5,8-Dihydro-5-(2-bromophenyl)-8-phenylnaphthalene-1,4-dione (**4p**)*

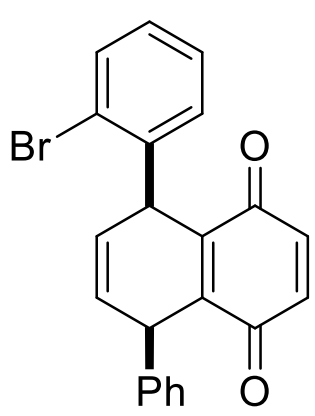

Obtained as an orange amorphous solid according to GP6 with TfOH:

- 57.2 mg, 0.201 mmol, 1.0 equiv 1-bromo-2-[(1*E*,3*E*)-4-phenyl-1,3-butadien-1-yl]benzene **1p**
- 86.4 mg, 0.799 mmol, 4.0 equiv *p*-benzoquinone **2a**
- 0.44 µL, 0.8 mg, 0.005 mmol, 2.5 mol% TfOH
- **1p** dissolved in 1.5 mL anhydrous DCM and added *via* syringe pump in 15 min (6 mL/h) to *p*-benzoquinone and TfOH dissolved in 0.75 mL anhydrous DCM at 0 °C
- After complete addition, stirred for another 2 h at r.t.
- Purified by flash column chromatography (10 g SiO_2, toluene)

Yield: 63.6 mg (0.163 mmol, 81%).

Melting point: 185 – 187 °C

***R*f** (toluene): 0.26.

^{1}H NMR (400 MHz, CD_2Cl_2): δ 7.66 (dd, *J* = 8.0, 1.3 Hz, 1H), 7.39 – 7.34 (m, 2H), 7.33 – 7.27 (m, 2H), 7.26 – 7.20 (m, 2H), 7.13 (ddd, *J* = 8.0, 7.4, 1.7 Hz, 1H), 7.05 (dd, *J* = 7.7, 1.7 Hz, 1H), 6.69 (q, *J* = 10.1 Hz, 2H), 6.07 (ddd, *J* = 9.9, 3.9, 1.5 Hz, 1H), 5.84 (ddd, *J* = 10.0, 4.0, 1.6 Hz, 1H), 5.15 (ddd, *J* = 6.6, 3.9, 1.6 Hz, 1H), 4.75 (ddd, *J* = 6.6, 4.0, 1.5 Hz, 1H).

^{13}C NMR (101 MHz, CD_2Cl_2): δ 186.7, 186.4, 143.9, 142.2, 141.9, 141.3, 137.1, 136.6, 133.8, 129.4, 128.9, 128.8, 128.7, 128.4, 127.3, 126.9, 124.9, 124.4, 41.0, 41.0.

IR (ATR, cm^{-1}): 1655 (s), 1599 (m), 1298 (m), 1118 (m), 1021 (m), 862 (m), 752 (s), 703 (m), 688 (m), 680 (m), 505 (m), 448 (m).

HRMS (ESI): calc. for $C_{22}H_{15}BrNaO_2^+$ $[M+Na]^+$: 413.0148; found: 413.0145.

*7.4.17 5,8-Dihydro-5-(3-bromophenyl)-8-phenylnaphthalene-1,4-dione (**4q**)*

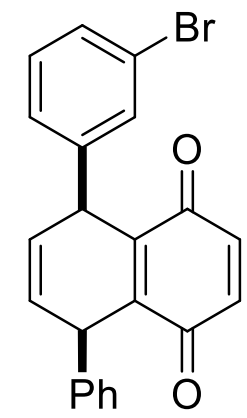

Obtained as an orange amorphous solid according to GP6 with TfOH:

- 57.2 mg, 0.201 mmol, 1.0 equiv 1-bromo-3-[(1*E*,3*E*)-4-phenyl-1,3-butadien-1-yl]benzene **1q**
- 87.4 mg, 0.809 mmol, 4.0 equiv *p*-benzoquinone **2a**
- 0.44 µL, 0.8 mg, 0.005 mmol, 2.5 mol% TfOH
- **1q** dissolved in 1.5 mL anhydrous DCM and added *via* syringe pump in 15 min (6 mL/h) to *p*-benzoquinone and TfOH dissolved in 0.75 mL anhydrous DCM at 0 °C
- After complete addition, stirred for another 30 min at r.t.
- Purified by flash column chromatography (10 g SiO_2, 20% cyclohexane in toluene)

Yield: 60.6 mg (0.155 mmol, 77%).

Melting point: 181 – 182 °C.

***R*f** (toluene): 0.26.

^{1}H NMR (400 MHz, CD_2Cl_2): δ 7.44 (t, *J* = 1.8 Hz, 1H), 7.40 – 7.16 (m, 8H), 6.69 (s, 2H), 5.97 (ddd, *J* = 9.9, 3.9, 1.0 Hz, 1H), 5.87 (ddd, *J* = 9.8, 3.9, 1.1 Hz, 1H), 4.79 – 4.69 (m, 2H).

^{13}C NMR (101 MHz, CD_2Cl_2): δ 186.6, 186.6, 144.5, 142.5, 142.1, 141.8, 136.9, 136.8, 132.0, 130.6, 130.3, 128.7, 127.8, 127.8, 127.3, 126.3, 122.9, 41.0, 40.8.

IR (ATR, cm^{-1}): 1651 (s), 1595 (m), 1584 (m), 1295 (m), 1115 (m), 860 (m), 793 (m), 761 (m), 741 (m), 702 (m), 689 (m), 510 (m), 479 (m), 438 (m).

HRMS (ESI): calc. for $C_{22}H_{15}BrNaO_2^+$ $[M+Na]^+$: 413.0148; found: 413.0153.

*7.4.18 5,8-Dihydro-5-(4-bromophenyl)-8-phenylnaphthalene-1,4-dione (**4r**)*

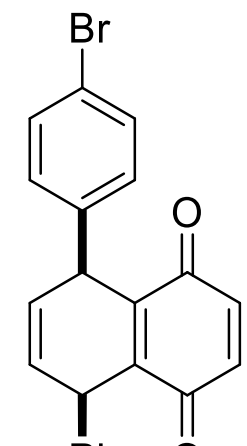

Obtained as an orange amorphous solid according to GP6 with TfOH:

- 57.5 mg, 0.202 mmol, 1.0 equiv 1-bromo-4-[(1*E*,3*E*)-4-phenyl-1,3-butadien-1-yl]benzene **1r**
- 87.2 mg, 0.806 mmol, 4.0 equiv *p*-benzoquinone **2a**
- 0.44 µL, 0.8 mg, 0.005 mmol, 2.5 mol% TfOH
- **1r** dissolved in 1.5 mL anhydrous DCM and added *via* syringe pump in 15 min (6 mL/h) to *p*-benzoquinone and TfOH dissolved in 0.75 mL anhydrous DCM at 0 °C
- After complete addition, stirred for another 60 min at r.t.
- Purified by flash column chromatography (10 g SiO_2, 20% cyclohexane in toluene)

Yield: 67.7 mg (0.173 mmol, 86%).

Melting point: 76 – 78 °C.

***R*f** (toluene): 0.26.

^{1}H NMR (400 MHz, CD_2Cl_2): δ 7.46 – 7.40 (m, 2H), 7.35 – 7.17 (m, 7H), 6.68 (s, 2H), 5.95 (ddd, *J* = 9.8, 3.8, 0.9 Hz, 1H), 5.86 (ddd, *J* = 9.8, 3.7, 1.0 Hz, 1H), 4.78 – 4.69 (m, 2H).

^{13}C NMR (101 MHz, CD_2Cl_2): δ 186.6, 142.4, 142.2, 142.0, 141.3, 136.9, 136.8, 132.0, 130.8, 129.1, 128.7, 127.6, 127.2, 126.3, 121.0, 41.0, 40.5.

IR (ATR, cm^{-1}): 1652 (s), 1599 (m), 1483 (m), 1451 (m), 1290 (m), 1113 (m), 1072 (m), 1028 (m), 1009 (m), 860 (s), 826 (m), 747 (m), 700 (m), 671 (m), 499 (m).

HRMS (ESI): calc. for $C_{22}H_{15}BrNaO_2^+$ $[M+Na]^+$: 413.0148; found: 413.0145.

*7.4.19 5,8-Dihydro-5-(4-iodophenyl)-8-phenylnaphthalene-1,4-dione **(4s)***

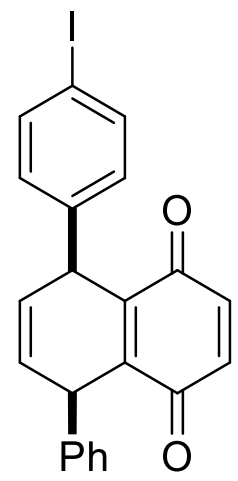

<u>Obtained as an orange amorphous solid according to GP6 with TfOH</u>:

- 66.8 mg, 0.201 mmol, 1.0 equiv 1-iodo-4-[(1*E*,3*E*)-4-phenyl-1,3-butadien-1-yl]benzene **1s**
- 88.3 mg, 0.817 mmol, 4.0 equiv *p*-benzoquinone **2a**
- 0.44 µL, 0.8 mg, 0.005 mmol, 2.5 mol% TfOH
- **1s** dissolved in 1.5 mL anhydrous DCM and added *via* syringe pump in 15 min (6 mL/h) to *p*-benzoquinone and TfOH dissolved in 0.75 mL anhydrous DCM at 0 °C
- After complete addition, stirred for another 60 min at r.t.
- Purified by flash column chromatography (10 g SiO_2, 20% cyclohexane in toluene)

Yield: 70.5 mg (0.161 mmol, 79%).

Melting point: 74 – 76 °C.

***R*f** (toluene): 0.29.

^{1}H NMR (400 MHz, CD_2Cl_2): δ 7.66 – 7.61 (m, 2H), 7.35 – 7.21 (m, 5H), 7.11 – 7.05 (m, 2H), 6.68 (s, 2H), 5.94 (ddd, *J* = 9.9, 3.9, 1.1 Hz, 1H), 5.86 (ddd, *J* = 9.8, 3.9, 1.2 Hz, 1H), 4.77- 4.73 (m, 1H), 4.72 – 4.68 (m, 1H).

^{13}C NMR (101 MHz, CD_2Cl_2): δ 186.6, 142.5, 142.2, 142.1, 141.9, 138.1, 136.9, 136.8, 131.1, 129.1, 128.7, 127.6, 127.3, 126.3, 92.4, 41.0, 40.7.

IR (ATR, cm^{-1}):1651 (s), 1599 (m), 1480 (m), 1289 (m), 1028 (m), 1005 (m), 859 (s), 808 (m), 748 (m), 699 (m), 667 (m), 498 (m).

HRMS (ESI): calc. for $C_{22}H_{15}INaO_2^+$ $[M+Na]^+$: 461.0009; found: 461.0012.

*7.4.20 5,8-Dihydro-5-(4-trifluoromethylphenyl)-8-phenylnaphthalene-1,4-dione **(4t)***

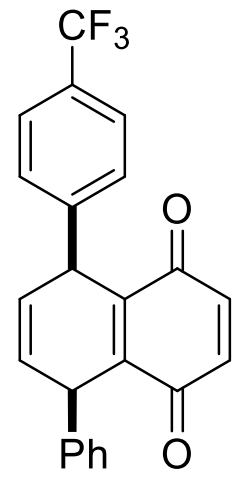

<u>Obtained as an orange amorphous solid according to GP6 with TfOH</u>:

- 55.3 mg, 0.202 mmol, 1.0 equiv 1-trifluoromethyl-4-[(1*E*,3*E*)-4-phenyl-1,3-butadien-1-yl]benzene **1t**
- 88.3 mg, 0.817 mmol, 4.0 equiv *p*-benzoquinone **2a**
- 0.44 µL, 0.8 mg, 0.005 mmol, 2.5 mol% TfOH
- **1t** dissolved in 1.5 mL anhydrous DCM and added *via* syringe pump in 15 min (6 mL/h) to *p*-benzoquinone and TfOH dissolved in 0.75 mL anhydrous DCM at 0 °C
- After complete addition, stirred for another 45 min at r.t.

▪ Purified by flash column chromatography (9 g SiO_2, toluene)

Yield: 69.7 mg (0.183 mmol, 91%).

Melting point: 147 – 149 °C.

***R*f** (toluene): 0.33.

¹H NMR (400 MHz, CD_2Cl_2): δ 7.57 (d, *J* = 8.1 Hz, 2H), 7.45 (d, *J* = 8.2 Hz, 2H), 7.35 – 7.22 (m, 5H), 6.70 (d, *J* = 0.7 Hz, 2H), 5.97 (ddd, *J* = 9.8, 4.0, 1.2 Hz, 1H), 5.88 (ddd, *J* = 9.9, 3.9, 1.2, 1H), 4.85 – 4.75 (m, 2H).

¹³C NMR (101 MHz, CD_2Cl_2): δ 186.6, 146.5, 142.8, 142.1, 141.7, 137.0, 136. 8, 129.4, 129.1, 128.7, 127.9, 127.3, 126.1, 125.9 (q, *J* = 3.9 Hz), 41.0.

¹⁹F NMR (377MHz, CD_2Cl_2): δ -62.75.

IR (ATR, cm^{-1}): 1654 (s), 1324 (s), 1302 (m), 1292 (m), 1109 (s), 1066 (m), 1016 (m), 869 (m), 860 (m), 837 (m), 826 (m), 706 (m), 502 (m).

HRMS (ESI): calc. for $C_{23}H_{15}F_3NaO_2^+$ $[M+Na]^+$: 403.0916; found: 403.0931.

*7.4.21 5,8-Dihydro-5-(4-methyl benzoate)-8-phenylnaphthalene-1,4-dione (**4u**)*

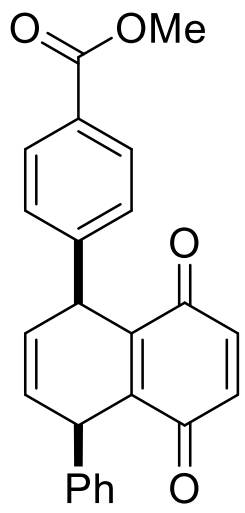

Obtained as an orange amorphous solid according to GP6 with TfOH:

▪ 53.1 mg, 0.201 mmol, 1.0 equiv methyl 4-[(1*E*,3*E*)-4-phenyl-1,3-butadien-1-yl]benzoate **1u**
▪ 87.7 mg, 0.811 mmol, 4.0 equiv *p*-benzoquinone **2a**
▪ 0.44 µL, 0.8 mg, 0.005 mmol, 2.5 mol% TfOH
▪ **1u** dissolved in 2.0 mL anhydrous DCM and added *via* syringe pump in 20 min (6 mL/h) to *p*-benzoquinone and TfOH dissolved in 0.75 mL anhydrous DCM at 0 °C
▪ After complete addition, stirred for another 60 min at r.t.
▪ Purified by flash column chromatography (10 g SiO_2, 4% EtOAc in toluene)

Yield: 62.0 mg (0.167 mmol, 83%).

Melting point: 138 – 140 °C.

***R*f** (toluene/EtOAc, 39:1): 0.23.

¹H NMR (400 MHz, CD_2Cl_2): δ 7.99 – 7.93 (m, 2H), 7.42 – 7.36 (m, 2H), 7.35 – 7.20 (m, 5H), 6.72 – 6.66 (m, 2H), 5.95 (ddd, *J* = 9.7, 3.7, 1.0 Hz, 1H), 5.89 5.95 (ddd, *J* = 10.0, 3.7, 1.0 Hz, 1H), 4.84 – 4.74 (m, 2H), 3.87 (s, 3H).

¹³C NMR (101 MHz, CD_2Cl_2): δ 186.6, 167.0, 147.5, 142.8, 142.1, 141.7, 137.0, 136.8, 130.2, 129.3, 129.1, 128.8, 127.7, 127.3, 126.2, 52.4, 41.2, 41.1.

IR (ATR, cm^{-1}): 1720 (s), 1655 (s), 1601 (m), 1278 (s), 1178 (m), 1116 (m), 1102 (m), 1026 (m), 1018 (m), 874 (m), 861 (m), 845 (m), 777 (m), 754 (m), 702 (m), 691 (m), 679 (m), 499 (m).

HRMS (ESI): calc. for $C_{24}H_{18}NaO_4^+$ $[M+Na]^+$: 393.1097; found: 393.1086.

*7.4.22 5,8-Dihydro-5-(4-cyanophenyl)-8-phenylnaphthalene-1,4-dione (**4v**)*

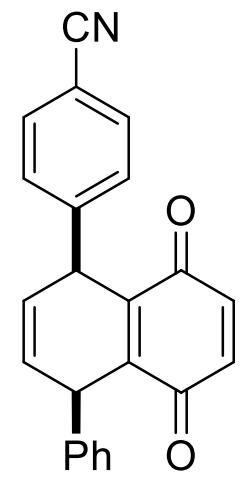

Obtained as an orange amorphous solid according to GP6 with TfOH:

- 46.5 mg, 0.201 mmol, 1.0 equiv 4-[(1*E*,3*E*)-4-phenyl-1,3-butadien-1-yl]benzonitril **1v**
- 87.5 mg, 0.809 mmol, 4.0 equiv *p*-benzoquinone **2a**
- 0.44 µL, 0.8 mg, 0.005 mmol, 2.5 mol% TfOH
- **1v** dissolved in 1.5 mL anhydrous DCM and added *via* syringe pump in 60 min (1.5 mL/h) to *p*-benzoquinone and TfOH dissolved in 1.0 mL anhydrous DCM at 0 °C
- After complete addition, stirred for another 60 min at r.t.
- Purified by flash column chromatography (9 g SiO_2, EtOAc in toluene gradient: starting with 4 CVs toluene, then 0.5% steps, each step two CVs)

Yield: 56.6 mg (0.168 mmol, 83%).

Melting point: 154 – 156 °C.

***R*f** (2.5% EtOAc in toluene): 0.23.

^{1}H NMR (400 MHz, CD_2Cl_2): δ 7.65 – 7.58 (m, 2H), 7.45 – 7.41 (m, 2H), 7.36 – 7.28 (m, 2H), 7.28 – 7.22 (m, 3H), 6.70 (d, *J* = 1.2 Hz, 2H), 5.99 (ddd, *J* = 9.5, 3.7, 1.0 Hz, 1H), 5.86 (ddd, *J* = 10.0, 3.7, 1.0 Hz, 1H), 4.84 – 4.75 (m, 2H).

^{13}C NMR (101 MHz, CD_2Cl_2): δ 186.5, 186.5, 147.5, 142.9, 141.9, 141.3 137.0, 136.7, 132.8, 129.7, 129.2, 128.6, 128.2, 127.4, 125.6, 119.0, 111.1, 41.2, 41.0.

IR (ATR, cm^{-1}): 1653 (s), 1600 (m), 1291 (m), 859 (m), 836 (m), 753 (m), 734 (m), 700 (m), 685 (m), 565 (m), 535 (m), 485 (m).

HRMS (ESI): calc. for $C_{23}H_{15}NNaO_2^+$ $[M+Na]^+$: 360.0995; found: 360.0990.

*7.4.23 5,8-Dihydro-5-(4-nitrophenyl)-8-phenylnaphthalene-1,4-dione (**4w**)*

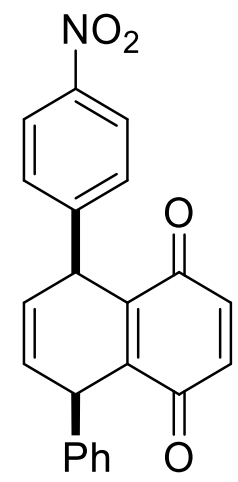

Obtained as an orange amorphous solid according to GP5 with TfOH:

- 51.8 mg, 0.206 mmol, 1.0 equiv 1-nitro-4-[(1*E*,3*E*)-4-phenyl-1,3-butadien-1-yl]benzene **1w**
- 90.5 mg, 0.837 mmol, 4.1 equiv *p*-benzoquinone **2a**
- 0.45 µL, 0.8 mg, 0.005 mmol, 2.5 mol% TfOH
- 2.0 mL anhydrous DCM as solvent
- Stirred at 0 °C for 2 h, then at r.t. for 3 h
- Purified by flash column chromatography (9 g SiO_2, EtOAc in toluene gradient: starting with 2 CVs toluene, then 0.5% steps, each step two CVs)

Yield: 65.5 mg (0.183 mmol, 89%).

Melting point: 181 – 182 °C.

***R*f** (toluene): 0.16.

^{1}H NMR (400 MHz, CD_2Cl_2): δ 8.18 – 8.11 (m, 2 H), 7.53 – 7.45 (m, 2H), 7.36 – 7.30 (m, 2H), 7.29 – 7.23 (m, 3H), 6.75 – 6.67 (m, 2H), 6.01 (ddd, *J* = 9.8, 4.0, 1.3 Hz, 1H), 5.88 (ddd, *J* = 9.9, 4.0, 1.3 Hz, 1H), 4.90 – 4.83 (m, 1H), 4.82 – 4.77 (m, 1H).

^{13}C NMR (101 MHz, CD_2Cl_2): δ 186.5, 186.4, 149.6, 147.2, 143.0, 141.8, 141.2, 137.1, 136.7, 129.8, 129.2, 128.6, 128.4, 127.4, 125.5, 124.2, 41.1, 41.0.

IR (ATR, cm^{-1}): 1656 (s), 1603 (m),1592 (m), 1512 (s), 1344 (s), 1291 (s), 1027 (m), 860 (s), 845 (s), 829 (m), 749 (s), 706 (s), 692 (m), 676 (m), 500 (m).

HRMS (ESI): calc. for $C_{22}H_{15}NNaO_4^+$ $[M+Na]^+$: 380.0893; found: 380.0899.

*7.4.24 5,8-Dihydro-5-ethyl-8-phenylnaphthalene-1,4-dione (**4x**)*

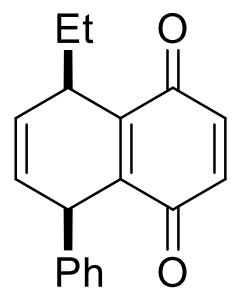

<u>Obtained as a yellow amorphous solid according to GP5 with MsOH:</u>

- 32.3 mg, 0.204 mmol, 1.0 equiv [(1*E*,3*E*)-1,3-hexadien-1-yl]benzene **1x**
- 48.2 mg, 0.446 mmol, 2.2 equiv *p*-benzoquinone **2a**
- 2.64 µL, 3.9 mg, 0.041 mmol, 0.2 equiv MsOH
- 2.0 mL anhydrous DCM as solvent
- Stirred at r.t. for 4 h
- Purified by flash column chromatography (8 g SiO_2, toluene)

Yield: 24.8 mg (0.094 mmol, 46%).

***R*f** (toluene): 0.34.

¹H NMR (400 MHz, CD_2Cl_2): δ 7.32 – 7.23 (m, 4 H), 7.23 – 7.17 (m, 1H), 6.73 (d, *J* = 10.0 Hz, 1H), 6.64 (d, *J* = 10.0 Hz, 1H), 5.98 (ddd, *J* = 9.9, 4.2, 1.1 HZ, 1H), 5.88 (dd, *J* = 10.4 Hz, 4.8 Hz, 1H), 4.63 (t, *J* = 4.7 Hz, 1H), 3.35 (sext., *J* = 4.5 Hz, 1H), 2.00 – 1.88 (m, 1H), 1.53 – 1.41 (m, 1 H), 1.07 (t, *J* = 7.5Hz, 3H).

¹³C NMR (101 MHz, CD_2Cl_2): δ 187.3, 186.8, 144.6, 142.7, 141.7, 136.9, 136.7, 129.0, 128.6, 128.1, 127.0, 126.7, 41.5, 36.2, 30.7, 12.2.

IR (ATR, cm^{-1}): 2963 (m), 1654 (s), 1646 (s), 1594 (m), 1492 (m), 1453 (m), 1293 (m), 1118 (m), 1028 (m), 859 (s), 834 (m), 758 (m), 701 (m), 672 (m) 536 (m), 476 (m).

HRMS (ESI): calc. for $C_{18}H_{16}NaO_2^+$ $[M+Na]^+$: 287.1043; found: 287.1041.

*7.4.25 5,8-Dihydro-5-cyclohexyl-8-phenylnaphthalene-1,4-dione (**4y**)*

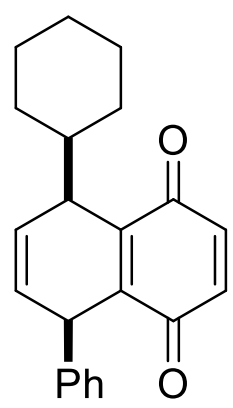

<u>Obtained as an orange amorphous solid according to GP6 with MsOH</u>:

- 44.4 mg, 0.209 mmol, 1.0 equiv [(1*E*,3*E*)-4-cyclohexyl-1,3-butadien-1-yl]benzene **1y**
- 90.7 mg, 0.839 mmol, 4.0 equiv *p*-benzoquinone **2a**
- 2.70 µL, 4.0 mg, 0.042 mmol, 0.2 equiv MsOH
- **1y** dissolved in 1.0 mL anhydrous DCM and added *via* syringe pump in 8 h (0.125 mL/h) to *p*-benzoquinone and MsOH dissolved in 1.0 mL anhydrous DCM at r.t.
- After complete addition, stirred for another 12 h at r.t.
- Purified by flash column chromatography (8 g SiO_2, toluene)

Yield: 24.9 mg (0.078 mmol, 37%).

***R*f** (toluene): 0.36.

¹H NMR (400 MHz, CD_2Cl_2): δ 7.33 – 7.15 (m, 5H), 6.76 (d, *J* = 10.0 Hz, 1H), 6.66 (d, *J* = 10.0 Hz, 1H), 5.96 – 5.86 (m, 2H), 4.66 (dd, *J* = 6.1, 3.8 Hz, 1H), 3.46 – 3.39 (m, 1H), 1.85 – 1.55 (m, 5H), 1.35 – 0.92 (m, 6H).

¹³C NMR (101 MHz, CD_2Cl_2): δ 187.4, 186.6, 145.2, 143.2, 142.5, 137.0, 136.7, 128.9, 128.6, 128.3, 126.8, 125.2, 43.7, 41.5, 40.4, 32.5, 30.4, 27.4, 26.8, 26.6.

IR (ATR, cm^{-1}): 2924 (w), 1651 (s), 1598 (w), 1445 (w), 1314 (w), 1296 (w), 1286 (w), 1254 (w), 1219 (w), 1025 (w), 952 (w), 858 (m), 836 (m), 757 (m), 698 (s), 670 (m), 507 (w), 484 (m).

HRMS (ESI): calc. for $C_{22}H_{22}NaO_2^+$ $[M+Na]^+$: 341.1512; found: 341.1512.

*7.4.26 5,8-Dihydro-5,8-di(4-methylphenyl)naphthalene-1,4-dione (**4z**)*

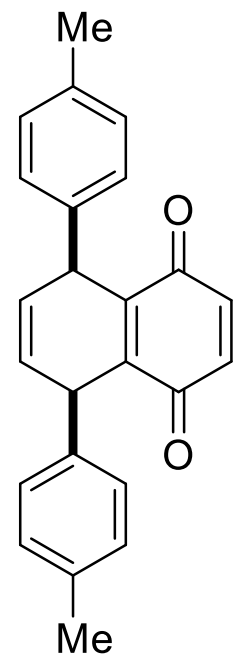

Obtained as a yellow amorphous solid according to GP5 with MsOH:

- 47.5 mg, 0.203 mmol, 1.0 equiv (1*E*,3*E*)-1,4-di(4-methylphenyl)-1,3-butadien **1z**
- 48.2 mg, 0.446 mmol, 2.2 equiv *p*-benzoquinone **2a**
- 2.62 µL, 3.9 mg, 0.041 mmol, 0.2 equiv MsOH
- 2.0 mL anhydrous DCM as solvent
- Stirred at r.t. for 90 min
- Purified by flash column chromatography (8 g SiO_2, 10% cyclohexane in toluene)

Yield: 44.7 mg (0.131 mmol, 65%).

Melting point: 183 – 185 °C.

***R*f** (toluene): 0.28.

^{1}H NMR (400 MHz, CD_2Cl_2): δ 7.18 (d, *J* = 8.2 Hz, 4H), 7.12 (d, *J* = 7.9 Hz, 4H), 6.65 (s, 2H), 5.88 – 5.83 (m, 2H), 4.71 – 4.67 (m, 2H), 2.31 (s, 6H).

^{13}C NMR (101 MHz, CD_2Cl_2): δ 186.9, 142.3, 139.4, 136.9, 136.8, 129.7, 128.7, 126.9, 40.7, 21.1.

IR (ATR, cm^{-1}): 1654 (s), 1510 (m), 1294 (m), 865 (s), 839 (m), 808 (s), 791 (m), 682 (m), 513 (m), 498 (m).

HRMS (ESI): calc. for $C_{24}H_{18}NaO_2^+$ [M+Na]$^+$: 363.1356; found: 363.1367.

*7.4.27 5,8-Dihydro-5,8-di(4-bromophenyl)naphthalene-1,4-dione (**4aa**)*

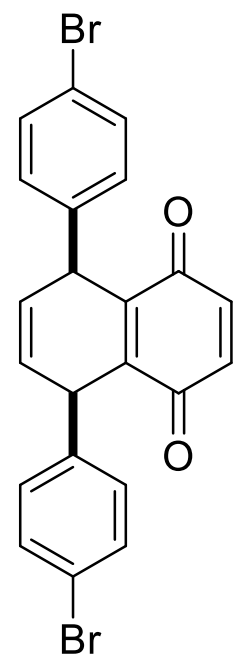

Obtained as an orange amorphous solid according to GP5 with TfOH:

- 73.8 mg, 0.203 mmol, 1.0 equiv (1*E*,3*E*)-1,4-di(4-bromophenyl)-1,3-butadien **1aa**
- 91.6 mg, 0.847 mmol, 4.2 equiv *p*-benzoquinone **2a**
- 0.44 µL, 0.8 mg, 0.005 mmol, 2.5 mol% TfOH
- 2.0 mL anhydrous DCM as solvent
- Stirred at 0 °C for 15 min, then at r.t. for 90 min
- Purified by flash column chromatography (9 g SiO_2, 20% cyclohexane in toluene)

Yield: 85.5 mg (0.182 mmol, 90%).

***R*f** (toluene): 0.37.

^{1}H NMR (400 MHz, CD_2Cl_2): δ 7.47 – 7.40 (m, 4H), 7.20 – 7.13 (m, 4H), 6.69 (s, 2H), 5.91 (d, *J* = 2.8 Hz, 2H), 4.72 (d, *J* = 2.5 Hz, 2H).

^{13}C NMR (101 MHz, CD_2Cl_2): δ 186.5, 142.0, 141.1, 136.9, 132.1, 130.6, 126.9, 121.1, 40.5.

IR (ATR, cm^{-1}): 1655 (s), 1483 (m), 1292 (m), 1072 (m), 1009 (m), 862 (s), 834 (m), 814 (s), 647 (m), 506 (m), 495 (m).

HRMS (ESI): calc. for $C_{22}H_{14}Br_2NaO_2^+$ [M+Na]$^+$: 492.9232; found: 492.9267.

Spectral data are in accordance with those reported in the literature.[26]

*7.4.28 5,8-Dihydro-5-ethoxycarbonyl-8-methylnaphthalene-1,4-dione (**4ab**)*

Step 1: 5,8-Dihydro-5-ethoxycarbonyl-8-methylnaphthalene-1,4-diol (**S15**)

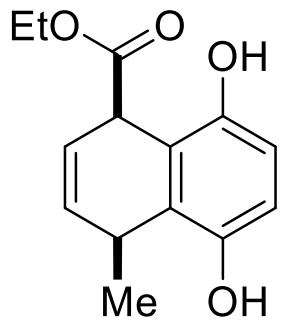

Under an inert gas atmosphere, 28.6 mg ethyl sorbate (0.204 mmol, 1.0 equiv) and 88.7 mg *p*-benzoquinone (0.821 mmol, 4.0 equiv) were dissolved in 2 mL anhydrous DCM and cooled to 0 °C with a cryostat. Then 0.448 µL TfOH (0.77 mg, 0.005 mmol, 2.5mol%) was added and the reaction was stirred at 0 °C for 16 h. The reaction was ended by the addition of 2 mL sat. $NaHCO_3$, diluted with 2 mL water, the phases were separated and the aqueous phase was extracted with DCM/EtOAc (3:1; 3 x 4 mL). The combined organic phases were dried over $MgSO_4$, filtered and the solvent was removed under reduced pressure. The residue was taken up in 1.5 mL AcMe and while stirring an aq. $TiCl_3$ solution was added until a dark purple color persisted. The mixture was diluted with 3 mL water and extracted with EtOAc (3 x 5 mL). The combined organic phases were washed with sat. $NaHCO_3$, water and brine (each 5 mL), dried over $MgSO_4$, filtered and the solvent removed under reduced pressure. The residue was purified by flash column chromatography (8 g SiO_2, 5% AcMe in DCM) to yield the product as a white amorphous solid.

Yield: 26.5 mg (0.107 mmol, 52%).

^{1}H NMR (400 MHz, AcMe-d6): δ 7.81 (s, 1H), 7.71 (s, 1H), 6.62 (d, *J* = 8.4 Hz, 1H), 6.54 (d, *J* = 8.4 Hz, 1H), 6.09 (ddd, *J* = 9.8, 5.1, 1.5 Hz, 1H), 5.90 (dd, *J* = 10.6, 5.1 Hz, 1H), 4.46 – 4.41 (m, 1H), 4.17 – 3.99 (m, 2H), 3.68 – 3.56 (m, 1H), 1.30 (d, *J* = 6.8 Hz, 3H), 1.19 (t, *J* = 7.2 Hz, 3H).

^{13}C NMR (101 MHz, AcMe-d6): δ 172.9, 148.5, 148.0, 135.0, 129.1, 122.4, 121.8, 114.4, 112.9, 61.0, 43.5, 30.8, 22.4, 14.5.

IR (ATR, cm^{-1}): 3462 (m), 1711 (s), 1487 (m), 1286 (s), 1267 (m), 1253 (m), 1199 (s), 1051 (m), 1032 (m), 1021 (m), 830 (m), 815 (m), 766 (m), 741 (m), 706 (m).

HRMS (ESI): calc. for $C_{14}H_{16}NaO_4^+$ $[M+Na]^+$: 271.0941; found: 271.0943.

Step 2: 5,8-Dihydro-5-ethoxycarbonyl-8-methylnaphthalene-1,4-dione (**4ab**)

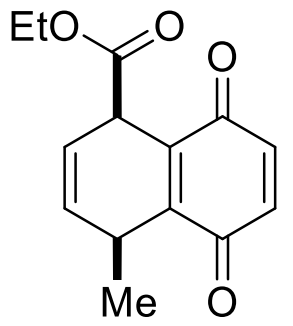

Under an inert gas atmosphere, 23.0 mg 5,8-dihydro-5-ethoxycarbonyl-8-methyl-1,4-naphthalenediol **S15** (0.093 mmol, 1.0 equiv), 35.0 mg Ag_2O (0.151 mmol, 1.6 equiv) and a small spatula $MgSO_4$ were suspended in 2 mL Et_2O and stirred overnight. After complete reaction the mixture was filtered through a pad of Celite® with Et_2O as eluent. The solvent was removed under reduced pressure to afford the product as an orange amorphous solid.

Yield: 22.8 mg (0.093 mmol, 99%).

***R*f** (toluene): 0.21.

^{1}H NMR (400 MHz, $CDCl_3$): δ 6.77 (s, 2H), 5.98 (ddd, *J* = 9.8, 4.5, 1.3 Hz, 1H), 5.88 (dd, *J* = 9.7, 3.9 Hz, 1H), 4.30 (td, *J* = 4.5, 1.3 Hz, 1H), 4.26 – 4.07 (m, 2H), 3.53 – 3.39 (m, 1H), 1.26 (t, *J* = 7.2 Hz, 6H).

^{13}C NMR (101 MHz, $CDCl_3$): δ 186.7, 186.4, 170.4, 145.4, 137.1, 137.0, 136.2, 132.5, 120.6, 61.7, 41.9, 29.6, 22.0, 14.2.

IR (ATR, cm^{-1}):1732 (s), 1653 (s), 1296 (s), 1250 (m), 1178 (s), 1117 (m), 1096 (m), 1064 (m), 1028 (m), 842 (s).

HRMS (ESI): calc. for $C_{14}H_{14}NaO_4^+$ $[M+Na]^+$: 269.0784; found: 269.0783.

*7.4.29 5,8-Dihydro-5-ethoxycarbonyl-8-propylnaphthalene-1,4-dione (**4ac**)*

Step 1: 5,8-Dihydro-5-ethoxycarbonyl-8-propylnaphthalene-1,4-diol (**S16**)

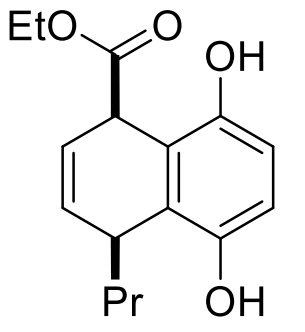

Under an inert gas atmosphere, 35.1 mg ethyl (2*E*,4*E*)-2,4-octadienoate (0.209 mmol, 1.0 equiv) and 91.2 mg *p*-benzoquinone (0.844 mmol, 4.0 equiv) were dissolved in 2 mL anhydrous DCM and cooled to 0 °C with a cryostat. Then 0.458 µL TfOH (0.8 mg, 0.005 mmol, 2.5mol%) was added and the reaction was stirred at 0 °C for 16 h. The reaction was ended by the addition of 2 mL sat. $NaHCO_3$, diluted with 2 mL water, the phases were separated and the aqueous phase was extracted with DCM/EtOAc (3:1; 3 x 4 mL). The combined organic phases were dried over $MgSO_4$, filtered and the solvent was removed under reduced pressure. The residue was taken up in 1.5 mL AcMe and while stirring an aq. $TiCl_3$ solution was added until a dark purple color persisted. The mixture was diluted with 3 mL water and extracted with EtOAc (3 x 5 mL). The combined organic phases were washed with sat. $NaHCO_3$, water and brine (each 5 mL), dried over $MgSO_4$, filtered and the solvent removed under reduced pressure. The residue was purified by flash column chromatography (8 g SiO_2, 5% AcMe in DCM) to yield the product as a white amorphous solid.

Yield: 38.9 mg (0.141 mmol, 68%).

^{1}H NMR (400 MHz, AcMe-d6): δ 7.80 (s, 1H), 7.69 (s, 1H), 6.62 (d, *J* = 8.4 Hz, 1H), 6.54 (d, *J* = 8.4 Hz, 1H), 6.16 (ddd, *J* = 9.9, 5.1, 1.5 Hz, 1H), 5.97 (dd, *J* = 9.8, 5.1 Hz, 1H), 4.47 – 4.42 (m, 1H), 4.16 – 4.01 (m, 2H), 3.66 – 3.59 (m, 1H), 1.90 – 1.80 (m, 1H), 1.67 – 1.56 (m, 1H), 1.46 – 1.33 (m, 1H), 1.19 (t, *J* = 7.1 Hz, 3H), 0.88 (t, *J* = 7.3 Hz, 3H).

^{13}C NMR (101 MHz, AcMe-d6): δ 172.7, 148.5, 147.9, 133.3, 128.5, 123.3, 122.1, 114.5, 112.9, 61.0, 43.6, 39.9, 35.5, 20.6, 14.6, 14.5.

IR (ATR, cm^{-1}): 3447 (m), 1711 (s), 1486 (m), 1286 (m), 1264 (m), 1195 (s), 1155 (m), 1025 (m), 1005 (m), 812 (m).

HRMS (ESI): calc. for $C_{16}H_{20}NaO_4^+$ [M+Na]$^+$: 299.1254; found: 299.1251.

Step 2: 5,8-Dihydro-5-ethoxycarbonyl-8-propylnaphthalene-1,4-dione (**4ac**)

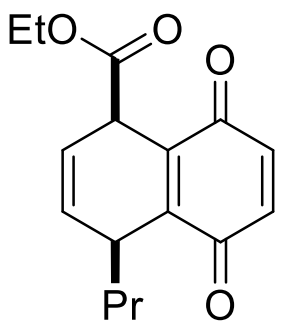

Under an inert gas atmosphere, 36.3 mg 5,8-dihydro-5-ethoxycarbonyl-8-propyl-1,4-naphthalenediol **S16** (0.131 mmol, 1.0 equiv), 47.0 mg Ag_2O (0.203 mmol, 1.5 equiv) and a small spatula $MgSO_4$ were suspended in 2 mL Et_2O and stirred overnight. After complete reaction, the mixture was filtered through a pad of Celite® with Et_2O as eluent. The solvent was removed under reduced pressure to afford the product as an orange amorphous solid.

Yield: 33.6 mg (0.122 mmol, 93%).

***R*$_f$** (toluene/EtOAc 39:1): 0.26.

^{1}H NMR (400 MHz, $CDCl_3$): δ 6.76 (s, 2H), 6.01 (ddd, *J* = 9.9, 4.4, 1.3 Hz, 1H), 5.93 (dd, *J* = 10.3, 4.7 Hz, 1H), 4.30 (td, *J* = 4.6, 1.3 Hz, 1H), 4.23 − 4.07 (m, 2H), 3.45 (dq, *J* = 8.6, 4.3 Hz, 1H), 1.71 – 1.59 (m, 1H), 1.54 – 1.43 (m, 1H), 1.42 – 1.28 (m, 2H), 1.24 (t, *J* = 7.2 Hz, 3H), 0.88 (t, *J* = 7.3 Hz, 3H).

^{13}C NMR (101 MHz, $CDCl_3$): δ 186.7, 186.2, 170.3, 144.7, 137.6, 137.0, 136.2, 130.9, 121.4, 61.7, 41.9, 38.3, 34.4, 19.8, 14.2, 14.2.

IR (ATR, cm^{-1}): 1733 (m), 1654 (s), 1294 (m), 1248 (m), 1179 (s), 1118 (m), 1095 (m), 1037 (m), 843 (m).

HRMS (ESI): calc. for $C_{16}H_{18}NaO_4^+$ [M+Na]$^+$: 297.1097; found: 297.1096.

*7.4.30 9,10-Dihydro-9,10[1',2']-benzenoanthracene-1,4-dione (4**ag**)*

Step 1: 9,10-Dihydro-9,10[1',2']-benzenoanthracene-1,4-diol (**S17**)

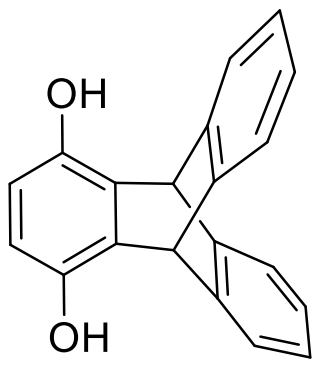

Under an inert gas atmosphere, 356 mg anthracene (2.00 mmol, 1.0 equiv) and 874 mg *p*-benzoquinone (8.09 mmol, 4.0 equiv) were dissolved in 20 mL anhydrous DCM and cooled to 0 °C. Then 8.76 µL TfOH (15.0 mg, 0.10 mmol, 2.5 mol%) was added and the reaction was stirred at 0 °C for 20 min. The reaction was ended by the addition of 15 mL sat. $NaHCO_3$ and the mixture was transferred to a separatory funnel. The phases were separated and the aqueous phase was extracted with DCM/EtOAc (3:1; 3 x 20 mL). The combined organic phases were dried over $MgSO_4$, filtered and the solvent was removed under reduced pressure. *p*-Benzoquinone was sublimed out of the mixture under reduced pressure and the residue was filtered through a silica plug (20 g) with DCM as eluent (approx. 300 mL). The obtained solid was suspended in 7 mL acetic acid and heated to reflux in an oil bath. Then 0.5 mL 48% aq. HBr (4.39 mmol, 2.2 equiv) was added and the resulting suspension was stirred at 130 °C until completion (approx. 30 min). After the suspension was cooled to r.t., it was filtered and the solid was washed with water. The residue was purified by flash column chromatography (35 g SiO_2, Et_2O in *n*-pentane gradient: starting from 50% Et_2O in *n*-pentane to pure Et_2O in 5% steps, each step one CV) to yield the product as a white amorphous solid.

Yield: 506 mg (1.77 mmol, 89%).

^{1}H NMR (400 MHz, AcMe-*d6*): δ 7.82 (s, 2H), 7.41 (dd, *J* = 5.3, 3.2 Hz, 4H), 6.97 (dd, *J* = 5.3, 3.1 Hz, 4H), 6.38 (s, 2H), 5.93 (s, 2H).

^{13}C NMR (101 MHz, AcMe-*d6*): δ 147.1, 146.1, 146.0, 133.6, 133.5, 125.6, 124.4, 114.0, 113.8, 48.3.

Spectral data are in accordance with those reported in the literature.[27]

Step 2: 9,10-Dihydro-9,10[1',2']-benzenoanthracene-1,4-dione (**4ag**)

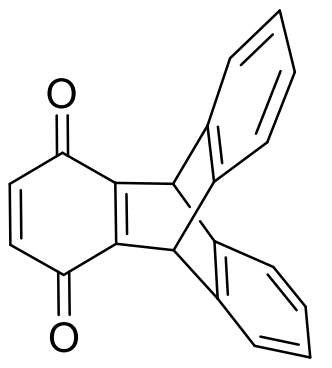

Under an inert gas atmosphere, 502 mg 9,10-dihydro-9,10[1',2']-benzenoanthracene-1,4-diol **S17** (1.75 mmol, 1.0 equiv) and 447 mg Ag_2O (1.93 mmol, 1.1 equiv) were suspended in 28 mL AcMe and stirred at r.t. until completion. The solvent was removed under reduced pressure and the residue was purified by flash column chromatography (SiO_2, toluene) to yield a yellow amorphous solid.

Yield: 498 mg (1.75 mmol, 99%).

***R*$_f$** (toluene): 0.32.

^{1}H NMR (400 MHz, $CDCl_3$): δ 7.43 (dd, *J* = 5.4, 3.2 Hz, 4H), 7.04 (dd, *J* = 5.4, 3.2 Hz, 4H), 6.60 (s, 2H), 5.80 (s, 2H).

^{13}C NMR (101 MHz, $CDCl_3$): δ 183.7, 152.1, 143.7, 135.6, 125.7, 124.6, 47.5.

IR (ATR, cm^{-1}): 1652 (s), 1592 (m), 1580 (m), 1455 (m), 1307 (m), 988 (m), 837 (m), 771 (m), 747 (m), 108 (m), 633 (m), 509 (s).

HRMS (ESI): calc. for $C_{20}H_{12}NaO_2^+$ $[M+H]^+$: 307.0730; found: 307.0731.

Spectral data are in accordance with those reported in the literature.[27]

*7.4.31 5,8-Dihydro-2-methyl-5,8-diphenylnaphthalene-1,4-dione (**4ah**)*

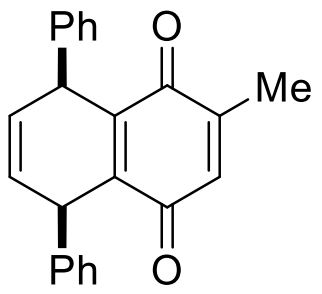

Obtained as an orange amorphous solid according to GP5 with TfOH:

- 103.7 mg, 0.503 mmol, 1.0 equiv (1*E*,3*E*)-1,4-diphenyl-1,3-butadiene **1a**
- 128.6 mg, 1.053 mmol, 2.1 equiv *p*-toluquinone **2b**
- 2.20 µL, 3.8 mg, 0.025 mmol, 5 mol% TfOH
- 5.0 mL anhydrous DCM as solvent
- Stirred at r.t. for 35 min
- Purified by flash column chromatography (20 g SiO_2, 10% cyclohexane in toluene)

Yield: 136 mg (0.415 mmol, 83%).

Melting point: 128 – 130 °C.

***R*f** (toluene): 0.30.

^{1}H NMR (400 MHz, CD_2Cl_2): δ 7.35 -7.26 (m, 8H), 7.26 – 7.19 (m, 2H), 6.52 (q, *J* = 1.6 Hz, 1H), 5.93 – 5.85 (m, 2H), 4.78 – 4.71 (m, 2H), 1.96 (d, *J* = 1.6 Hz, 3H).

^{13}C NMR (101 MHz, CD_2Cl_2): δ 187.3, 186.7, 146.1, 142.7, 142.6, 142.3, 142.2, 133.7, 129.0, 128.9, 128.8, 127.1, 127.1, 126.9, 41.3, 41.0, 15.9.

IR (ATR, cm^{-1}):1650 (s), 1609 (m), 1492 (m), 1373 (m), 1274 (m, 1178 (m), 896 (m), 844 (m), 737 (m), 717 (m), 694 (s), 677 (m), 636 (m), 493 (m).

HRMS (ESI): calc. for $C_{23}H_{18}NaO_2^+$ [M+Na]$^+$: 349.1199; found: 349.1193.

*7.4.32 5,8-Dihydro-2,3-dimethyl-5,8-diphenylnaphthalene-1,4-dione (**4ai**)*

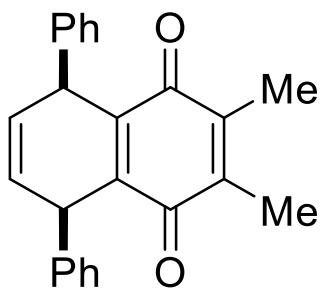

Obtained as an orange amorphous solid according to GP5 with TfOH:

- 100.4 mg, 0.487 mmol, 1.0 equiv (1*E*,3*E*)-1,4-diphenyl-1,3-butadiene **1a**
- 132.7 mg, 0.975 mmol, 2.0 equiv 2,3-dimethylbenzoquinone **2c**
- 1.068 µL, 1.8 mg, 0.012 mmol, 2.5 mol% TfOH
- 9.5 mL anhydrous DCM as solvent
- Stirred at r.t. for 75 min
- Purified by flash column chromatography (SiO_2, 40% DCM in *n*-hexane)

Yield: 144.6 mg (0.425 mmol, 87%).

Melting point: 146 – 148 °C.

***R*f** (toluene): 0.31.

^{1}H NMR (400 MHz, CD_2Cl_2): δ 7.35 – 7.27 (m, 8H), 7.25 – 7.18 (m, 2H), 5.90 – 5.84 (m, 2H), 4.78 – 4.72 (m, 2H), 1.93 (s, 6H).

^{13}C NMR (101 MHz, CD_2Cl_2): δ 186.7, 142.9, 141.9, 141.2, 129.0, 128.8, 127.1, 127.0, 41.3, 12.4.

IR (ATR, cm^{-1}): 1649 (s), 1615 (m), 1281 (m), 841 (m), 738 (m), 726 (m), 693 (s), 649 (m), 523 (m), 424 (m).

HRMS (ESI): calc. for $C_{24}H_{20}NaO_2^+$ [M+Na]$^+$: 363.1356; found: 363.1357.

*7.4.33 5,8-Dihydro-2-cyclopropyl-5,8-diphenylnaphthalene-1,4-dione (**4aj**)*

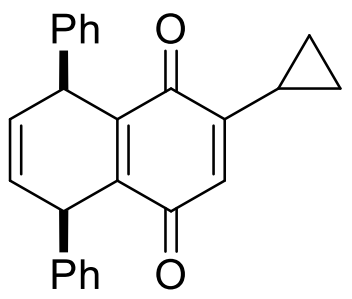

Obtained as an orange amorphous solid according to GP5 with TfOH:

- 41.8 mg, 0.203 mmol, 1.0 equiv (1*E*,3*E*)-1,4-diphenyl-1,3-butadiene **1a**
- 63.2 mg, 0.427 mmol, 2.1 equiv 2-cyclopropylbenzoquinone **2d**
- 0.888 µL, 1.5 mg, 0.010 mmol, 5 mol% TfOH
- 2.0 mL anhydrous DCM as solvent
- Stirred at r.t. for 35 min
- Purified by flash column chromatography (10 g SiO_2, 10% Et_2O in *n*-pentane)

Yield: 47.4 mg (0.134 mmol, 66%).

Melting point: 139 – 141 °C.

***R*$_f$** (20% Et_2O in *n*-pentane): 0.32; ***R*$_f$** (toluene): 0.32.

^{1}H NMR (400 MHz, CD_2Cl_2): δ 7.35 – 7.27 (m, 8H), 7.26 – 7.18 (m, 2H), 6.06 – 6.02 (m, 1H), 5.92 – 5.85 (m, 2H), 4.82 – 4.69 (m, 2H), 2.04 – 1.91 (m, 1H), 1.12 – 0.93 (m, 2H), 0.74 – 0.63 (m, 2H).

^{13}C NMR (101 MHz, CD_2Cl_2): δ 186.6, 186.3, 152.6, 142.7, 142.7, 142.3, 142.1, 129.0, 129.0, 128.8, 128.8, 127.2, 127.1, 127.0, 126.9, 126.5, 41.4, 41.0, 11.4, 11.0, 9.2.

IR (ATR, cm^{-1}): 1644 (m), 1606 (m), 1452 (m), 1280 (m), 916 (m), 845 (m), 747 (m), 727 (m), 694 (s), 504 (m).

HRMS (ESI): calc. for $C_{25}H_{20}NaO_2^+$ $[M+Na]^+$: 375.1356; found: 375.1348.

*7.4.34 5,8-Dihydro-2-cyclohexyl-5,8-diphenylnaphthalene-1,4-dione (**4ak**)*

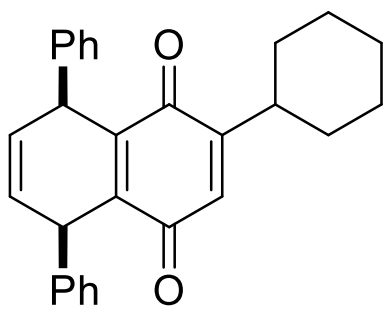

Obtained as an orange amorphous solid according to GP5 with TfOH:

- 41.4 mg, 0.201 mmol, 1.0 equiv (1*E*,3*E*)-1,4-diphenyl-1,3-butadiene **1a**
- 81.6 mg, 0.429 mmol, 2.1 equiv 2-cyclohexylbenzoquinone **2e**
- 0.880 µL, 1.5 mg, 0.010 mmol, 5 mol% TfOH
- 2.0 mL anhydrous DCM as solvent
- Stirred at r.t. for 35 min
- Purified by flash column chromatography (10 g SiO_2, 20% cyclohexane in toluene)

Yield: 65.1 mg (0.165 mmol, 82%).

Melting point: 74 – 76 °C.

***R*$_f$** (toluene): 0.53.

^{1}H NMR (400 MHz, CD_2Cl_2): δ 7.34 – 7.26 (m, 8H), 7.25 – 7.19 (m, 2H), 6.41 (d, *J* = 1.2 Hz, 1H), 5.94 – 5.84 (m, 2H), 4.80 – 4.70 (m, 2H), 2.66 – 2.55 (m, 1H), 1.85 – 1.64 (m, 4H), 1.60 – 1.53 (m, 1H), 1.43 – 1.03 (m, 5H).

^{13}C NMR (101 MHz, CD_2Cl_2): δ 187.3, 186.7, 154.2, 142.8, 142.6, 142.4, 141.9, 131.0, 129.0, 128.9, 128.9, 128.6, 127.3, 127.0, 126.8, 41.4, 41.0, 36.8, 32.6, 32.2, 26.7, 26.7, 26.4.

IR (ATR, cm^{-1}): 2925 (m), 2851 (m), 1651 (s), 1608 (m), 1492 (m), 1272 (m), 750 (m), 731 (m), 696 (m).

HRMS (ESI): calc. for $C_{28}H_{26}NaO_2^+$ $[M+Na]^+$: 417.1825; found: 417.1828.

*7.4.35 5,8-Dihydro-2,5,8-triphenylnaphthalene-1,4-dione (**4al**)*

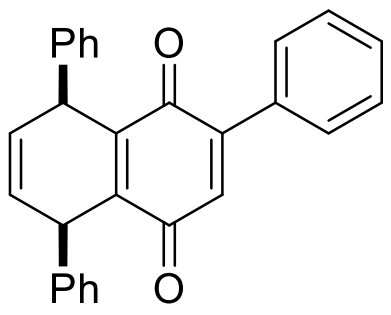

Obtained as an orange amorphous solid according to GP5 with TfOH:

- 41.7 mg, 0.202 mmol, 1.0 equiv (1*E*,3*E*)-1,4-diphenyl-1,3-butadiene **1a**
- 77.0 mg, 0.418 mmol, 2.1 equiv 2-phenylbenzoquinone **2f**
- 0.887 µL, 1.5 mg, 0.010 mmol, 5 mol% TfOH
- 2.0 mL anhydrous DCM as solvent
- Stirred at r.t. for 60 min
- Purified by flash column chromatography (10 g SiO_2, 40% DCM in *n*-hexane)

Yield: 62.9 mg (0.162 mmol, 80%).

***R*$_f$** (toluene): 0.36.

^{1}H NMR (400 MHz, CD_2Cl_2): δ 7.49 – 7.26 (m, 13H), 7.28 – 7.19 (m, 2H), 6.78 (s, 1H), 5.99 – 5.89 (m, 2H), 4.89 – 4.84 (m, 1H), 4.83 – 4.77 (m, 1H).

^{13}C NMR (101 MHz, CD_2Cl_2): δ 186.8, 186.4, 146.3, 142.7, 142.6, 142.5, 142.3, 133.4, 132.8, 130.2, 129.6, 129.1, 129.0, 128.9, 128.8, 128.7, 127.2, 127.1, 126.9, 41.4, 41.1.

IR (ATR, cm^{-1}):1645 (s), 1597 (m), 1490 (m), 1447 (m), 1271 (m), 1205 (m), 897 (m), 835 (m), 747 (m), 721 (m), 693 (s), 510 (m).

HRMS (ESI): calc. for $C_{28}H_{20}NaO_2^+$ $[M+Na]^+$: 411.1356; found: 411.1353.

*7.4.36 5,7,10,12-Tetrahydro-7,10-diphenyl-5,12[1',2']benzenonaphthacene-6,11-dione (**4am**)*

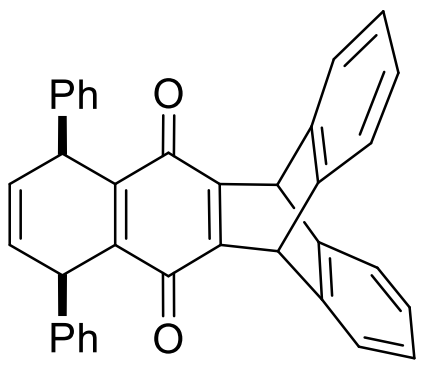

Obtained as a yellow amorphous solid according to GP5 with TfOH:

- 41.3 mg, 0.200 mmol, 1.0 equiv (1*E*,3*E*)-1,4-diphenyl-1,3-butadiene **1a**
- 117.1 mg, 0.412 mmol, 2.1 equiv 9,10-dihydro-9,10[1',2']benzenoanthracene-1,4-dione **4ag**
- 0.878 µL, 1.5 mg, 0.010 mmol, 5 mol% TfOH
- 4.0 mL anhydrous DCM as solvent
- Stirred at r.t. for 15 min
- Purified by flash column chromatography (8 g SiO_2, 20% cyclohexane in toluene)

Yield: 87.2 mg (0.178 mmol, 89%).

Melting point: 290 – 294 °C.

***R*$_f$** (toluene): 0.54.

^{1}H NMR (400 MHz, CD_2Cl_2): δ 7.39 (dd, *J* = 5.3, 3.2 Hz, 2H), 7.34 (dd, *J* = 5.4, 3.2 Hz, 2H), 7.30 – 7.24 (m, 8H), 7.24 – 7.15 (m, 2H), 6.99 (dt, *J* = 5.6, 2.8 Hz, 4H), 5.82 (d, *J* = 2.9 Hz, 2H), 5.73 (s, 2H), 4.70 (d, *J* = 2.8 Hz, 2H).

^{13}C NMR (101 MHz, CD_2Cl_2): δ 183.0, 152.0, 144.6, 144.0, 142.6, 141.2, 129.0, 128.9, 127.1, 126.9, 125.8, 125.8, 124.8, 124.6, 47.8, 41.5.

IR (ATR, cm^{-1}):1645 (s), 1598 (m), 1456 (m), 1287 (m), 842 (m), 747 (m), 739 (m), 725 (m), 695 (m), 680 (m), 591 (s), 564 (m), 518 (m).

HRMS (ESI): calc. for $C_{36}H_{24}NaO_2^+$ $[M+Na]^+$: 511.1669; found: 511.1651.

*7.4.37 5,8-Dihydro-2-chloro-5,8-diphenylnaphthalene-1,4-dione (**4an**)*

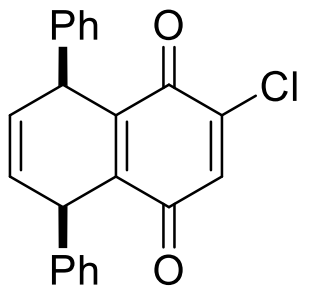

Obtained as a red solid according to GP6 with MsOH:

- 41.3 mg, 0.200 mmol, 1.0 equiv (1*E*,3*E*)-1,4-diphenyl-1,3-butadiene **1a**
- 57.6 mg, 0.404 mmol, 2.0 equiv 2-chlorobenzoquinone **2g**
- 2.56 µL, 3.8 mg, 0.039 mmol, 0.2 equiv MsOH
- **1a** dissolved in 1.25 mL anhydrous DCM and added *via* syringe pump in 3 h (0.43 mL/h) to *p*-benzoquinone and MsOH dissolved in 0.75 mL anhydrous DCM at r.t.
- After complete addition, stirred for another 1.5 h at r.t.
- Purified by flash column chromatography (10 g SiO_2, Et_2O in *n*.pentane gradient, starting from 5% Et_2O in *n*-pentane two CVs, then 1% steps, each step two CVs)

Yield: 41.3 mg (0.119 mmol, 60%).

Melting point: 75 – 77 °C.

***R*f** (10% Et_2O in *n*-pentane): 0.39; ***R*f** (toluene): 0.43.

^{1}H NMR (400 MHz, CD_2Cl_2): δ 7.39 – 7.21 (m, 10H), 6.92 (s, 1H), 5.96 – 5.88 (m, 2H), 4.82 – 4.74 (m, 2H).

^{13}C NMR (101 MHz, CD_2Cl_2): δ 184.5, 179.4, 143.9, 142.9, 142.6, 142.1, 141.9, 134.1, 129.1, 129.1, 128.9, 128.7, 127.4, 127.3, 126.8, 126.7, 41.8, 41.3.

IR (ATR, cm^{-1}):1666 (m), 1650 (m), 1593 (m), 1268 (m), 1208 (m), 1045 (m), 885 (m), 831 (m), 747 (m), 721 (m), 694 (s), 518 (m).

HRMS (ESI): calc. for $C_{22}H_{15}ClNaO_2^+$ $[M+Na]^+$: 369.0653; found: 369.0621.

*7.4.38 5,8-Dihydro-2-acetamido-5,8-diphenylnaphthalene-1,4-dione (**4ao**)*

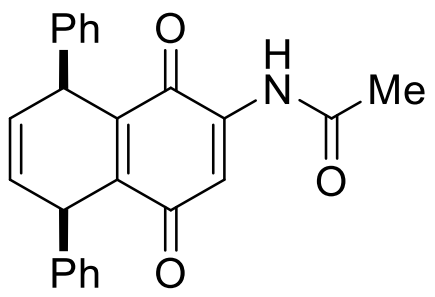

Obtained as a yellow amorphous solid according to GP6 with TfOH:

- 41.2 mg, 0.200 mmol, 1.0 equiv (1*E*,3*E*)-1,4-diphenyl-1,3-butadiene **1a**
- 78.4 mg, 0.402 mmol, 2.0 equiv 2-acetamido-1,4-benzoquinone **2h**
- 36.0 µL, 61.6 mg, 0.410 mmol, 2.1 equiv TfOH
- **11** dissolved in 1.0 mL anhydrous DCM and added in 3 min to 2-acetamido-1,4-benzoquinone **2h** and TfOH dissolved in 1.0 mL anhydrous DCM at 0 °C
- After complete addition, stirred for another 2 h at r.t.
- Extraction with EtOAc
- Purified by flash column chromatography (10 g SiO_2, 20% EtOAc in toluene)

Yield: 48.8 mg (0.132 mmol, 66%).

Melting point: 188 – 190 °C.

***R*f** (20% EtOAc in toluene): 0.42.

^{1}H NMR (400 MHz, CD_2Cl_2): δ 7.99 (s, 1H), 7.38 (s, 1H), 7.31 (d, *J* = 4.2 Hz, 8H), 7.27 – 7.19 (m, 2H), 5.92 – 5.86 (m, 2H), 4.82 – 4.76 (m, 1H), 4.74 – 4.69 (m, 1H), 2.13 (s, 3H).

^{13}C NMR (101 MHz, CD_2Cl_2): δ 187.0, 182.7, 169.7, 144.0, 142.5, 142.4, 139.4, 138.6, 129.1, 129.0, 128.8, 128.7, 127.3, 127.2, 126.9, 126.8, 114.9, 41.4, 41.1, 25.0.

IR (ATR, cm^{-1}): 1656 (m), 1634 (m), 1491 (s), 1451 (m), 1314 (m), 1226 (m), 1194 (m), 1183 (m), 875 (m), 716 (m), 691 (s), 510 (s).

HRMS (ESI): calc. for $C_{24}H_{19}NNaO_3^+$ $[M+Na]^+$: 392.1257; found: 392.1259.

*7.4.39 1,4-Dihydro-1,4-diphenylanthracene-9,10-dione (**4ap**)*

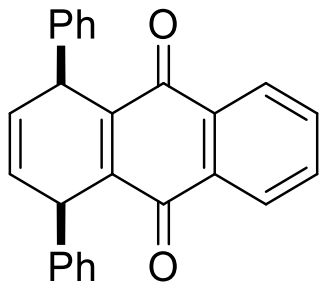

Obtained as a yellow amorphous solid according to GP5 with TfOH:

- 41.7 mg, 0.202 mmol, 1.0 equiv (1*E*,3*E*)-1,4-diphenyl-1,3-butadiene **1a**
- 64.8 mg, 0.410 mmol, 2.1 equiv 1,4-naphthoquinone
- 0.878 µL, 1.5 mg, 0.010 mmol, 5 mol% TfOH
- 2.0 mL anhydrous DCM as solvent
- Stirred at r.t. for 2 h
- Purified by flash column chromatography (9 g SiO_2, 15% cyclohexane in toluene)

Yield: 52.2 mg (0.144 mmol, 71%).

Melting point: 206 – 207°C.

$\boldsymbol{R}_\mathbf{f}$ (10% cyclohexane in toluene): 0.28.

^{1}H NMR (400 MHz, CD_2Cl_2): δ 8.00 – 7.93 (m, 2H), 7.72 – 7.65 (m, 2H), 7.39 – 7.28 (m, 6H), 7.26 – 7.19 (m, 4H), 5.95 (d, *J* = 2.8 Hz, 2H), 4.96 (d, *J* = 2.7 Hz, 2H).

^{13}C NMR (101 MHz, CD_2Cl_2): δ 184.4, 144.7, 142.8, 134.0, 132.6, 129.0, 128.8, 127.1, 127.0, 126.6, 41.6.

IR (ATR, cm^{-1}): 1661 (s), 1592 (m), 1285 (s), 850 (m), 790 (m), 736 (s), 726 (s), 692 (s), 669 (s), 611 (m), 517 (m).

HRMS (ESI): calc. for $C_{26}H_{18}NaO_2^+$ [M+Na]$^+$: 385.1199; found: 385.1199.

7.5 Synthesis and Analytical Data of 1,4-Naphthoquinones (5)

*7.5.1 5,8-Diphenylnaphthalene-1,4-dione (**5a**)*

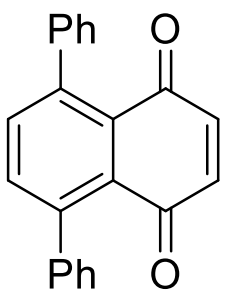

Obtained as an orange amorphous solid according to GP7:

- 472 mg, 1.51 mmol, 1.0 equiv 5,8-dihydro-5,8-diphenylnaphthalene-1,4-dione **4a**
- 786 mg, 9.04 mmol, 6.0 equiv MnO_2
- 15 mL toluene as solvent
- Stirred at 110 °C for 19 h
- Purified by flash column chromatography (40 g SiO_2, cyclohexane in toluene gradient: starting with two CVs 10% cyclohexane in toluene, 2.5% steps, each step two CVs, then 4% EtOAc in toluene)

Yield: 424 mg (1.37 mmol, 91%).

Melting point: 139 – 141 °C.

$\boldsymbol{R}_\mathbf{f}$ (toluene): 0.12.

^{1}H NMR (400 MHz, CD_2Cl_2): δ 7.52 (s, 2H), 7.48 – 7.36 (m, 6H), 7.32 – 7.25 (m, 4H), 6.78 (s, 2H).

^{13}C NMR (101 MHz, CD_2Cl_2): δ 186.0, 143.5, 142.1, 138.9, 136.6, 131.4, 128.5, 128.4, 127.5.

IR (ATR, cm^{-1}): 3052 (w), 1663 (s), 1315 (m), 1240 (m), 1105 (m), 864 (m), 846 (m), 761 (s), 697 (s), 525 (m), 419 (m).

HRMS (ESI): calc. for $C_{22}H_{14}NaO_2^+$ [M+Na]$^+$: 333.0886; found: 333.0890.

*7.5.2 5-(4-Biphenyl)-8-phenylnaphthalene-1,4-dione (**5b**)*

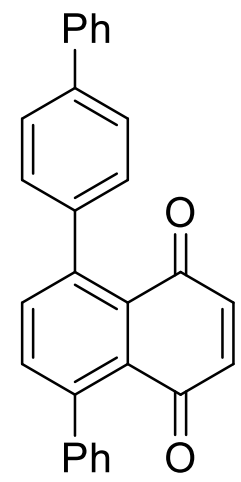

Obtained as an orange amorphous solid according to GP7:

- 59.4 mg, 0.15 mmol, 1.0 equiv 5,8-dihydro-5-(4-biphenyl)-8-phenylnaphthalene-1,4-dione **4b**
- 152 mg, 1.75 mmol, 11.4 equiv MnO_2
- 1.5 mL toluene as solvent
- Stirred at 110 °C for 3 h
- Purified by flash column chromatography (4g SiO_2, toluene)

Yield: 43.1 mg (0.11 mmol, 73%).

Melting point: 176 – 177 °C.

***R*f** (toluene): 0.24.

^{1}H NMR (400 MHz, CD_2Cl_2): δ 7.74 – 7.65 (m, 4H), 7.60 – 7.53 (m, 8H), 7.52 – 7.35 (m, 2H), 7.32 – 7.26 (m, 2H), 6.81 (d, *J* = 1.3 Hz, 2H).

^{13}C NMR (101 MHz, CD_2Cl_2): δ 186.1, 186.0, 143.6, 143.2, 142.1, 141.2, 141.1, 140.3, 139.0, 138.9, 136.7, 136.6, 131.5, 131.4, 129.2, 129.1, 128.5, 128.4, 127.8, 127.5, 127.4, 127.1.

IR (ATR, cm^{-1}):1662 (s), 1625 (m), 1485 (m), 1462 (m), 1321 (m), 1243 (m), 1102 (m), 1006 (m), 862 (m), 850 (m), 828 (m), 763 (s), 731 (m), 701 (s), 663 (m), 557 (m), 528 (m), 511 (m).

HRMS (ESI): calc. for $C_{28}H_{18}NaO_2^+$ $[M+Na]^+$: 409.1199; found: 409.1197.

*7.5.3 5-(2-Methylphenyl)-8-phenylnaphthalene-1,4-dione (**5c**)*

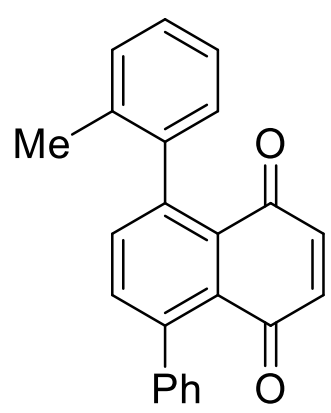

Obtained as an orange amorphous solid according to GP7:

- 35.5 mg, 0.109 mmol, 1.0 equiv 5,8-dihydro-5-(2-methylphenyl)-8-phenylnaphthalene-1,4-dione **4c**
- 98 mg, 1.13 mmol, 10.3 equiv MnO_2
- 1.5 mL toluene as solvent
- Stirred at 110 °C for 4 h
- Purified by flash column chromatography (4g SiO_2, toluene)

Yield: 25.4 mg (0.078 mmol, 72%).

Melting point: 77 – 79 °C.

***R*f** (toluene): 0.22.

^{1}H NMR (400 MHz, CD_2Cl_2): δ 7.54 (d, *J* = 7.8 Hz, 1H), 7.48 – 7.37 (m, 4H), 7.35 – 7.21 (m, 5H), 7.05 (d, *J* = 7.0 Hz, 1H), 6.81 – 6.72 (m, 2H), 2.05 (s, 3H).

^{13}C NMR (101 MHz, CD_2Cl_2): δ 186.2, 185.9, 143.5, 142.8, 142.2, 142.0, 139.1, 138.7, 136.9, 136.4, 135.5, 131.3, 131.1, 129.9, 128.6, 128.4, 127.9, 127.6, 127.5, 126.0, 20.2.

IR (ATR, cm^{-1}): 1661 (s), 1619 (m), 1601 (m), 1318 (m), 1242 (m), 1096 (m), 1026 (m), 847 (m), 754 (s), 726 (m), 698 (m), 662 (m), 520 (m), 453 (m), 440 (m).

HRMS (ESI): calc. for $C_{23}H_{16}NaO_2^+$ $[M+Na]^+$: 347.1043; found: 347.1059.

*7.5.4 5-(3-Methylphenyl)-8-phenylnaphthalene-1,4-dione (**5d**)*

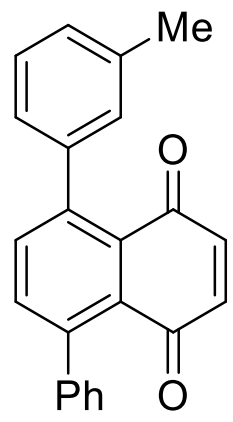

Obtained as an orange amorphous solid according to GP7:

- 47.4 mg, 0.145 mmol, 1.0 equiv 5,8-dihydro-5-(3-methylphenyl)-8-phenylnaphthalene-1,4-dione **4d**
- 134 mg, 1.54 mmol, 10.36 equiv MnO_2
- 1.5 mL toluene as solvent
- Stirred at 110 °C for 3 h
- Purified by flash column chromatography (4g SiO_2, toluene)

Yield: 41.3 mg (0.127 mmol, 88%).

Melting point: 140 – 141 °C.

***R*f** (toluene): 0.21.

^{1}H NMR (400 MHz, CD_2Cl_2): δ 7.51 (s, 2H), 7.48 – 7.38 (m, 3H), 7.36 – 7.19 (m, 4H), 7.14 – 7.03 (m, 2H), 6.78 (s, 2H), 2.41 (s, 3H).

^{13}C NMR (101 MHz, CD_2Cl_2): δ 186.1, 186.0, 143.7, 143.4, 142.2, 142.1, 138.9, 138.9, 138.2, 136.6, 136.5, 131.4, 131.3, 129.1, 128.5, 128.4, 128.3, 128.2, 127.5, 125.7, 21.6.

IR (ATR, cm^{-1}): 1661 (s), 1620 (m), 1602 (m), 1538 (m), 1460 (m), 1443 (m), 1320 (m), 1267 (m), 1241 (m), 1102 (m), 1058(m), 1039 (m), 1019 (m), 847 (m), 796 (m), 780 (m), 760 (m), 698 (m), 659 (m), 582 (m), 523 (m), 440 (m).

HRMS (ESI): calc. for $C_{23}H_{16}NaO_2^+$ [M+Na]$^+$: 347.1043; found: 347.1045.

*7.5.5 5-(4-Methylphenyl)-8-phenylnaphthalene-1,4-dione (**5e**)*

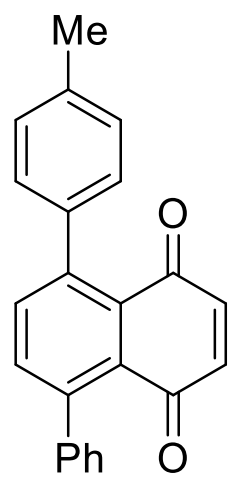

Obtained as an orange amorphous solid according to GP7:

- 43.4 mg, 0.133 mmol, 1.0 equiv 5,8-dihydro-5-(4-methylphenyl)-8-phenylnaphthalene-1,4-dione **4e**
- 119 mg, 1.36 mmol, 10.3 equiv MnO_2
- 1.5 mL toluene as solvent
- Stirred at 100 °C for 4 h
- Purified by flash column chromatography (4g SiO_2, toluene)

Yield: 36.2 mg (0.112 mmol, 84%).

Melting point:167 – 169 °C.

***R*f** (*n*-pentane/Et_2O, 4:1): 0.19; ***R*f** (toluene): 0.17.

^{1}H NMR (400 MHz, CD_2Cl_2): δ 7.50 (s, 2H), 7.47 – 7.37 (m, 3H), 7.31 – 7.22 (m, 4H), 7.20 – 7.14 (m, 2H), 6.77 (s, 2H), 2.44 (s, 3H).

^{13}C NMR (101 MHz, CD_2Cl_2): δ 186.1, 186.1, 143.6, 143.3, 142.2, 139.1, 139.0, 138.8, 137.4, 136.7, 136.5, 131.4, 129.1, 128.5, 128.5, 128.4, 127.4, 21.4.

IR (ATR, cm^{-1}): 1657 (s), 1615, (m), 1326 (m), 1241 (m), 1102 (m), 1034 (m), 1018 (m), 854 (m), 831 (m), 812 (m), 761 (m), 695 (m), 524(m), 423 (m).

HRMS (ESI): calc. for $C_{23}H_{16}NaO_2^+$ [M+Na]$^+$: 347.1043; found: 347.1044.

*7.5.6 5-(4-Butylphenyl)-8-phenylnaphthalene-1,4-dione (**5f**)*

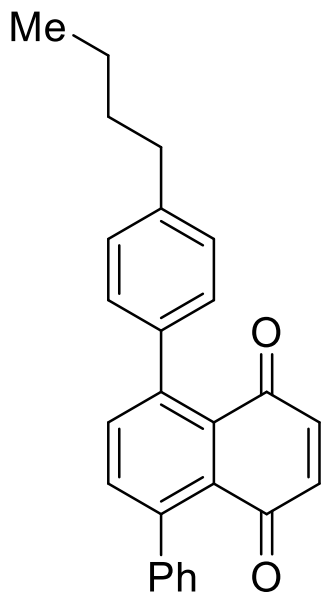

<u>Obtained as an orange amorphous solid according to GP7</u>:

- 49.9 mg, 0.135 mmol, 1.0 equiv 5,8-dihydro-5-(4-butylphenyl)-8-phenylnaphthalene-1,4-dione **4f**
- 121 mg, 1.39 mmol, 10.3 equiv MnO_2
- 1.5 mL toluene as solvent
- Stirred at 110 °C for 22 h
- Purified by flash column chromatography (6 g SiO_2, toluene)

Yield: 38.1 mg (0.104 mmol, 77%).

Melting point: 80 – 81 °C.

***R*f** (toluene): 0.24.

^{1}H NMR (400 MHz, CD_2Cl_2): δ 7.55 – 7.48 (m, 2H), 7.46 – 7.37 (m, 3H), 7.31 – 7.23 (m, 4H), 7.21 – 7.16 (m, 2H), 6.77 (d, *J* = 0.9 Hz, 2H), 2.70 (t, *J* = 7.9 Hz, 2H), 1.68 (quint, *J* = 7.6 Hz, 2H), 1.43 (sext, *J* = 7.5 Hz, 2H), 0.98 (t, *J* = 7.4 Hz, 3H).

^{13}C NMR (101 MHz, CD_2Cl_2): δ 186.2, 186.1, 143.7, 143.3, 142.5, 142.2, 139.2, 139.0, 138.8, 136.8, 136.5, 131.4, 128.5, 128.5, 128.4, 127.4, 35.8, 34.1, 22.9, 14.2.

IR (ATR, cm^{-1}): 1662 (s), 1320 (m), 1242 (m), 1099 (m), 1053 (m), 1034 (m), 1019 (m), 850 (m), 833 (m), 761 (m), 734 (m), 698 (m), 664 (m), 543 (m), 523 (m).

HRMS (ESI): calc. for $C_{26}H_{22}NaO_2^+$ $[M+Na]^+$: 389.1512; found: 389.1508.

*7.5.7 5-(4-tert-Butylphenyl)-8-phenylnaphthalene-1,4-dione (**5g**)*

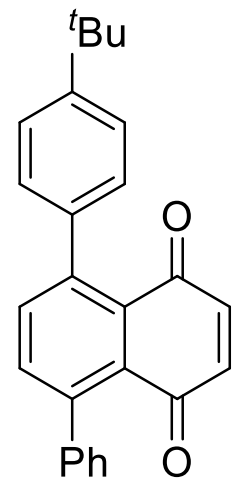

<u>Obtained as orange amorphous solid according to GP7</u>:

- 44.0 mg, 0.119 mmol, 1.0 equiv 5,8-dihydro-5-(4-*tert*-butylphenyl)-8-phenylnaphthalene-1,4-dione **4g**
- 112 mg, 1.29 mmol, 10.8 equiv MnO_2
- 1.5 mL toluene as solvent
- Stirred at 110 °C for 3 h
- Purified by flash column chromatography (4 g SiO_2, toluene)

Yield: 38.5 mg (0.105 mmol, 88%).

Melting point: 75 – 77 °C.

***R*f** (toluene): 0.29.

^{1}H NMR (400 MHz, CD_2Cl_2): δ 7.56 – 7.49 (m, 2H), 7.48 – 7.37 (m, 5H), 7.31 – 7.19 (m, 4H), 6.78 (d, *J* = 1.1 Hz, 2H), 1.40 (s, 9H).

^{13}C NMR (101 MHz, CD_2Cl_2): δ 186.2, 186.1, 150.6, 143.6, 143.3, 142.2, 139.0, 139.0, 138.9, 136.8, 136.6, 131.4, 128.6, 128.4, 128.3, 127.5, 125.4, 34.9, 31.6.

IR (ATR, cm^{-1}): 1662 (s), 1320 (m), 1264 (m), 1242 (m), 1098 (m), 1019 (m), 850 (m), 836 (m), 822 (m), 762 (m), 698 (m), 560 (m), 525 (m).

HRMS (ESI): calc. for $C_{26}H_{22}KO_2^+$ $[M+K]^+$: 405.1251; found: 405.1251.

*7.5.8 5-(2-Methoxyphenyl)-8-phenylnaphthalene-1,4-dione (**5h**)*

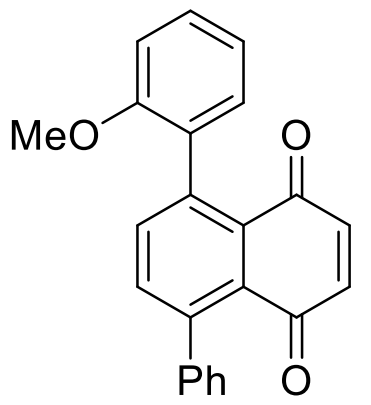

Obtained as an orange amorphous solid according to GP7:

- ▪ 28.2 mg, 0.082 mmol, 1.0 equiv 5,8-dihydro-5-(2-methoxyphenyl)-8-phenylnaphthalene-1,4-dione **4h**
- ▪ 47 mg, 0.541 mmol, 6.6 equiv MnO_2
- ▪ 3.0 mL $CHCl_3$ as solvent
- ▪ Stirred at 62 °C for 18 h
- ▪ Purified by flash column chromatography (8 g SiO_2, 1% EtOAc in toluene)

Yield: 21.9 mg (0.064 mmol, 78%).

Melting point: 131 – 133 °C.

***R*f** (1% EtOAc in toluene): 0.13.

^{1}H NMR (400 MHz, CD_2Cl_2): δ 7.55 – 7.47 (m, 2H), 7.47 – 7.37 (m, 4H), 7.33 – 7.26 (m, 2H), 7.19 (dd, *J* = 7.4, 1.8 Hz, 1H), 7.07 (td, *J* = 7.4, 1.0 Hz, 1H), 6.96 (d, *J* = 8.3 Hz, 1H), 6.81 – 6.74 (m, 2H), 3.70 (s, 3H).

^{13}C NMR (101 MHz, CD_2Cl_2): δ 186.1, 185.9, 156.4, 143.2, 142.2, 139.2, 139.0, 138.7, 136.7, 136.6, 131.1, 130.7, 129.4, 129.2, 128.6, 128.4, 127.4, 121.2, 55.7.

IR (ATR, cm^{-1}): 1663 (s), 1492 (m), 1465 (m, 1433 (m), 1317 (m), 1243 (m), 1096 (m), 1016 (m), 846 (m), 752 (s), 700 (m), 690 (m).

HRMS (ESI): calc. for $C_{23}H_{16}NaO_3^+$ $[M+Na]^+$: 363.0992; found: 363.0993.

*7.5.9 5-(3-Methoxyphenyl)-8-phenylnaphthalene-1,4-dione (**5i**)*

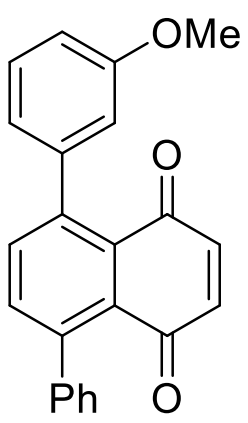

Obtained as an orange amorphous solid according to GP7:

- ▪ 33.6 mg, 0.098 mmol, 1.0 equiv 5,8-dihydro-5-(3-methoxyphenyl)-8-phenylnaphthalene-1,4-dione **4i**
- ▪ 65 mg, 0.748 mmol, 7.6 equiv MnO_2
- ▪ 3.0 mL $CHCl_3$ as solvent
- ▪ Stirred at 62 °C for 20 h
- ▪ Purified by flash column chromatography (8 g SiO_2, 2% EtOAc in toluene)

Yield: 29.6 mg (0.087 mmol, 89%).

Melting point: 111 – 112 °C.

***R*f** (1% EtOAc in toluene): 0.12.

^{1}H NMR (600 MHz, CD_2Cl_2): δ 7.54 – 7.50 (m, 2H), 7.47 – 7.38 (m, 3H), 7.36 – 7.32 (m, 1H), 7.29 – 7.26 (m, 2H), 6.94 (ddd, *J* = 8.3, 2.6, 1.0 Hz, 1H), 6.85 (ddd, *J* = 7.5, 1.6, 0.9 Hz, 1H), 6.81 (dd, *J* = 2.6, 1.6 Hz, 1H), 6.78 (d, *J* = 1.2 Hz, 2H), 3.83 (s, 3H).

^{13}C NMR (151 MHz, CD_2Cl_2): δ 186.0, 185.9, 159.9, 143.6, 143.5, 143.3, 142.1, 138.9, 138.9, 136.6, 136.4, 131.4, 131.3, 129.4, 128.5, 128.4, 127.5, 120.9, 114.4, 112.6, 55.6.

IR (ATR, cm^{-1}): 2922 (m), 1661 (s), 1599 (m), 1578 (m), 1462 (m), 1319 (m), 1243 (m), 1210 (m), 1101 (m), 1044 (m), 1030 (m), 846 (m), 761 (m), 697 (m).

HRMS (ESI): calc. for $C_{23}H_{16}NaO_3^+$ $[M+Na]^+$: 363.0992; found: 363.0971.

*7.5.10 5-(4-Methoxyphenyl)-8-phenylnaphthalene-1,4-dione (**5j**)*

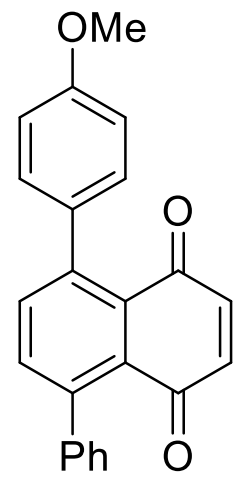

Obtained as an orange amorphous solid according to GP7:

- 29.6 mg, 0.086 mmol, 1.0 equiv 5,8-dihydro-5-(4-methoxyphenyl)-8-phenylnaphthalene-1,4-dione **4j**
- 77 mg, 0.886 mmol, 10.2 equiv MnO_2
- 3.0 mL $CHCl_3$ as solvent
- Stirred at 62 °C for 18 h
- Purified by flash column chromatography (8 g SiO_2, toluene)

Yield: 24.2 mg (0.071 mmol, 82%).

Melting point: 150 – 152 °C.

***R*f** (1% EtOAc in toluene): 0.12.

^{1}H NMR (400 MHz, CD_2Cl_2): δ 7.52 (d, *J* = 7.8 Hz, 1H), 7.49 (d, *J* = 7.9 Hz, 1H), 7.45 – 7.38 (m, 3H), 7.28 – 7.26 (m, 2H), 7.23 – 7.20 (m, 2H), 6.98 – 6.95 (m, 2H), 6.77 (d, *J* = 2.9 Hz, 2H), 3.87 (s, 3H).

^{13}C NMR (101 MHz, CD_2Cl_2): δ 186.2, 186.1, 159.5, 143.3, 143.2, 142.2, 139.0, 138.8, 136.9, 136.6, 134.1, 131.5, 131.4, 129.9, 128.5, 128.4, 127.4, 113.9, 55.7.

IR (ATR, cm^{-1}): 1657 (s), 1606 (m), 1513 (m), 1460 (m), 1326 (m), 1241 (s), 1181 (m), 1103 (m), 1038 (m), 1026 (m), 854 (m), 831 (m), 822 (m), 763 (m), 682 (m), 541 (m), 527 (m).

HRMS (ESI): calc. for $C_{23}H_{16}NaO_3^+$ $[M+Na]^+$: 363.0992; found: 363.0983.

*7.5.11 5-(1-Naphthyl)-8-phenylnaphthalene-1,4-dione (**5k**)*

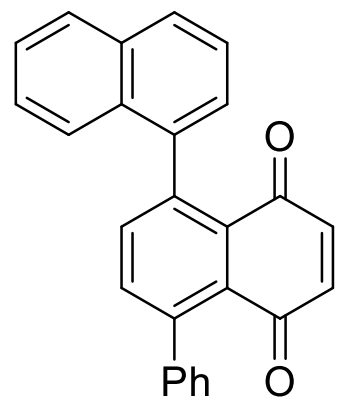

Obtained as an orange amorphous solid according to GP7:

- 37.4 mg, 0.119 mmol, 1.0 equiv 5,8-dihydro-5-(1-naphthyl)-8-phenylnaphthalene-1,4-dione **4k**
- 88 mg, 1.01 mmol, 10.0 equiv MnO_2
- 1.0 mL toluene as solvent
- Stirred at 110 °C for 3 h
- Purified by flash column chromatography (4 g SiO_2, toluene)

Yield: 29.3 mg (0.081 mmol, 80%).

Melting point: 152 – 154 °C.

***R*f** (toluene): 0.18.

^{1}H NMR (400 MHz, CD_2Cl_2): δ 7.94 (t, *J* = 9.3 Hz, 2H), 7.68 – 7.27 (m, 12H), 6.80 (d, *J* = 10.1 Hz, 1H), 6.67 (d, *J* = 10.3 Hz, 1H).

^{13}C NMR (101 MHz, CD_2Cl_2): δ 186.2, 185.4, 143.9, 142.1, 141.4, 140.3, 139.1, 138.7, 137.2, 137.0, 133.7, 132.2, 132.2, 131.2, 128.8, 128.6, 128.5, 127.8, 127.6, 126.4, 126.2, 125.9, 125.5, 125.3.

IR (ATR, cm^{-1}): 1662 (s), 1318 (m), 1244 (m), 1105 (m), 1086 (m), 1044 (m), 1020 (m), 849 (m), 801 (m), 782 (m), 762 (m), 698 (m), 664 (m), 579 (m), 433 (m).

HRMS (ESI): calc. for $C_{26}H_{16}NaO_2^+$ $[M+Na]^+$: 383.1043; found: 383.1056.

*7.5.12 5-(2-Naphthyl)-8-phenylnaphthalene-1,4-dione (**5l**)*

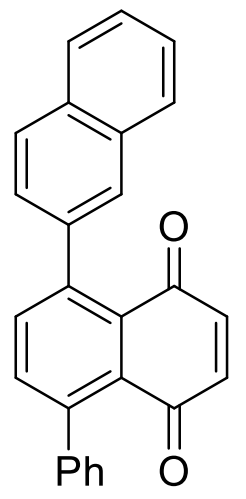

Obtained as an orange amorphous solid according to GP7:

- 39.8 mg, 0.108 mmol, 1.0 equiv 5,8-dihydro-5-(2-naphthyl)-8-phenylnaphthalene-1,4-dione **4l**
- 84 mg, 0.966 mmol, 8.9 equiv MnO_2
- 1.0 mL toluene as solvent
- Stirred at 110 °C for 18 h
- Purified by flash column chromatography (6 g SiO_2, toluene)

Yield: 38.0 mg (0.105 mmol, 98%).

Melting point: 185 – 187 °C.

***R*f** (toluene): 0.17.

^{1}H NMR (400 MHz, CD_2Cl_2): δ 7.96 – 7.85 (m, 3H), 7.77 (m, 1H), 7.63 (d, *J* = 7.9 Hz, 1H), 7.59 – 7.52 (m, 3H), 7.48 – 7.36 (m, 4H), 7.33 – 7.28 (m, 2H), 6.80 (d, *J* = 1.1 Hz, 2H).

^{13}C NMR (101 MHz, CD_2Cl_2): δ 186.1, 186.0, 143.7, 143.4, 142.1, 139.9, 139.0, 138.9, 136.9, 136.8, 133.8, 132.9, 131.4, 128.6, 128.5, 128.5, 128.3, 128.1, 127.6, 127.5, 127.5, 126.7, 126.5, 126.5.

IR (ATR, cm^{-1}): 1660 (s), 1620 (m), 1320 (m), 1243 (m), 1100 (m), 864 (m), 849 (m), 821 (m), 791 (m), 749 (m), 695 (m), 662 (m), 524 (m), 476 (m), 441 (m).

HRMS (ESI): calc. for $C_{26}H_{16}NaO_2^+$ [M+Na]$^+$: 383.1043; found: 383.1056.

*7.5.10 5-(2-Chlorophenyl)-8-phenylnaphthalene-1,4-dione (**5n**)*

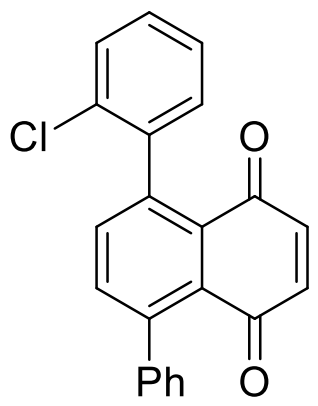

Obtained as a yellow amorphous solid according to GP7:

- 53.4 mg, 0.154 mmol, 1.0 equiv 5,8-dihydro-5-(2-chlorophenyl)-8-phenylnaphthalene-1,4-dione **4n**
- 160 mg, 1.61 mmol, 10.5 equiv MnO_2
- 3.0 mL toluene as solvent
- Stirred at 110 °C for 21 h
- Purified by flash column chromatography (8 g SiO_2, toluene)

Yield: 40.9 mg (0.119 mmol, 77%).

Melting point: 122 – 123 °C.

***R*f** (toluene): 0.17.

^{1}H NMR (400 MHz, CD_2Cl_2): δ 7.58 (d, *J* = 7.8 Hz, 1H), 7.50 – 7.36 (m, 7H), 7.32 – 7.28 (m, 2H), 7.27 – 7.22 (m, 1H), 6.79 (d, *J* = 1.2 Hz, 2H).

^{13}C NMR (101 MHz, CD_2Cl_2): δ 185.9, 185.6, 144.1, 141.9, 141.0, 139.9, 139.3, 138.5, 137.0, 136.1, 132.5, 131.7, 130.9, 129.8, 129.4, 129.0, 128.6, 128.4, 127.6, 127.3.

IR (ATR, cm^{-1}): 3052 (vw), 3021 (wv), 2922 (vw), 2852 (vw), 1659 (s), 1321 (m), 1242 (m), 1104 (m), 1026 (m), 847 (m), 754 (s), 694 (m), 518 (m), 458 (m), 444 (m).

HRMS (ESI): calc. for $C_{22}H_{13}ClNaO_2^+$ [M+Na]$^+$: 367.0496; found: 367.0505.

*7.5.11 5-(4-Chlorophenyl)-8-phenylnaphthalene-1,4-dione (**5o**)*

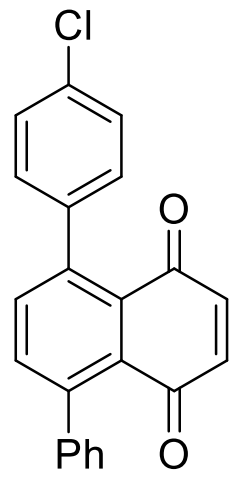

Obtained as a yellow amorphous solid according to GP7:

- 53.4 mg, 0.154 mmol, 1.0 equiv 5,8-dihydro-5-(4-chlorophenyl)-8-phenylnaphthalene-1,4-dione **4o**
- 137 mg, 1.58 mmol, 10.2 equiv MnO_2
- 1.5 mL toluene as solvent
- Stirred at 110 °C for 18 h
- Purified by flash column chromatography (8 g SiO_2, toluene)

Yield: 43.2 mg (0.125 mmol, 81%).

Melting point: 124 – 126 °C.

***R*f** (toluene): 0.16.

^{1}H NMR (400 MHz, CD_2Cl_2): δ 7.53 (d, *J* = 7.9 Hz, 1H), 7.49 (d, *J* = 7.9 Hz, 1H), 7.47 – 7.37 (m, 5H), 7.30 – 7.25 (m, 2H), 7.25 – 7.20 (m, 2H), 6.79 (s, 2H).

^{13}C NMR (101 MHz, CD_2Cl_2): δ 186.0, 185.9, 143.9, 142.2, 142.0, 140.7, 139.0, 138.8, 136.7, 136.4, 133.4, 131.4, 131.3, 130.0, 128.6, 128.5, 128.4, 127.6.

IR (ATR, cm^{-1}): 2922 (w), 2852 (w), 1659 (s), 1322 (m), 1242 (m), 1100 (m), 1086 (m), 1011 (m), 853 (m), 830 (m), 815 (m), 764 (m), 701 (m), 527 (m).

HRMS (ESI): calc. for $C_{22}H_{13}ClNaO_2^+$ $[M+Na]^+$: 367.0496; found: 367.0488.

*7.5.12 5-(2-Bromophenyl)-8-phenylnaphthalene-1,4-dione (**5p**)*

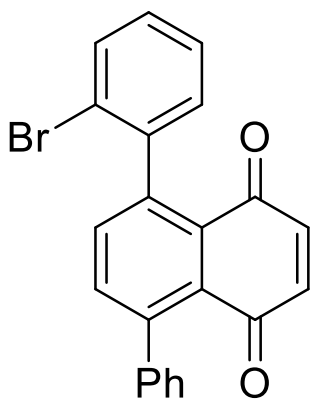

Obtained as a brown solid according to GP7:

- 60.6 mg, 0.155 mmol, 1.0 equiv 5,8-dihydro-5-(2-bromophenyl)-8-phenylnaphthalene-1,4-dione **4p**
- 138 mg, 1.59 mmol, 10.2 equiv MnO_2
- 3.0 mL toluene as solvent
- Stirred at 110 °C for 20 h
- Purified by flash column chromatography (8 g SiO_2, toluene)

Yield: 51.6 mg (0.133 mmol, 86%).

Melting point: 121 – 122 °C.

***R*f** (toluene): 0.19.

^{1}H NMR (400 MHz, CD_2Cl_2): δ 7.67 (dd, *J* = 8.0, 1.2 Hz, 1H), 7.58 (d, *J* = 7.9 Hz, 1H), 7.48 – 7.40 (m, 5H), 7.33 – 7.27 (m, 3H), 7.24 (dd, *J* = 7.6, 1.7 Hz, 1H), 6.79 (d, *J* = 1.7 Hz, 2H).

^{13}C NMR (101 MHz, CD_2Cl_2): δ 185.9, 185.5, 144.1, 143.0, 141.9, 141.6, 139.3, 138.5, 137.0, 136.1, 132.6, 131.6, 130.8, 129.7, 129.2, 128.6, 128.4, 127.9, 127.6, 122.6.

IR (ATR, cm^{-1}): 3046 (w), 2922 (w), 2852 (w), 1658 (s), 1321 (m), 1247 (m), 1103 (m), 1103 (m), 1016 (m), 848 (m), 755 (s), 693 (s), 659 (m), 517 (m), 449 (m).

HRMS (ESI): calc. for $C_{22}H_{13}BrNaO_2^+$ $[M+Na]^+$: 410.9991; found: 410.9997.

*7.5.13 5-(3-Bromophenyl)-8-phenylnaphthalene-1,4-dione (**5q**)*

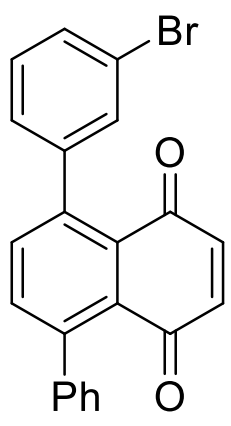

Obtained as an orange amorphous solid according to GP7:

- 68.4 mg, 0.175 mmol, 1.0 equiv 5,8-dihydro-5-(3-bromophenyl)-8-phenylnaphthalene-1,4-dione **4q**
- 156 mg, 1.79 mmol, 10.3 equiv MnO_2
- 3.0 mL toluene as solvent
- Stirred at 110 °C for 20 h
- Purified by flash column chromatography (8 g SiO_2, toluene)

Yield: 52.4 mg (0.135 mmol, 77%).

Melting point: 162 – 164 °C.

***R*f** (toluene): 0.17.

^{1}H NMR (400 MHz, CD_2Cl_2): δ 7.57 – 7.52 (m, 2H), 7.49 (d, *J* = 7.8 Hz, 1H), 7.47 – 7.39 (m, 4H), 7.33 (t, *J* = 7.8 Hz, 1H), 7.29 – 7.25 (m, 2H), 7.22 (ddd, *J* = 7.7, 1.7, 1.1 Hz, 1H), 6.79 (s, 2H).

^{13}C NMR (101 MHz, CD_2Cl_2): δ 185.8, 185.8, 144.2, 144.0, 141.9, 141.7, 139.1, 138.8, 136.8, 136.3, 131.3, 131.2, 130.5, 130.0, 128.5, 128.5, 127.6, 127.4, 122.2.

IR (ATR, cm^{-1}): 1662 (s), 1317 (m), 1240 (m), 1105 (m), 862 (m), 846 (m), 791 (m), 760 (m), 732 (m), 695 (m), 526 (m), 431 (m).

HRMS (ESI): calc. for $C_{22}H_{13}BrNaO_2^+$ $[M+Na]^+$: 410.9991; found: 410.9991.

*7.5.14 5-(4-Bromophenyl)-8-phenylnaphthalene-1,4-dione (**5r**)*

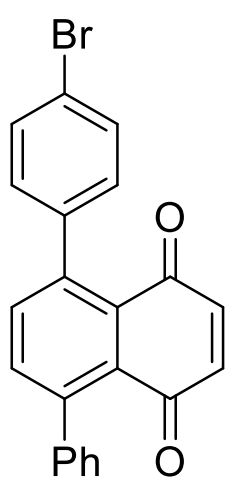

Obtained as an orange amorphous solid according to GP7:

- 58.5 mg, 0.150 mmol, 1.0 equiv 5,8-dihydro-5-(4-bromophenyl)-8-phenylnaphthalene-1,4-dione **4r**
- 136 mg, 1.56 mmol, 10.5 equiv MnO_2
- 3.0 mL toluene as solvent
- Stirred at 110 °C for 19 h
- Purified by flash column chromatography (8 g SiO_2, toluene)

Yield: 48.1 mg (0.124 mmol, 83%).

Melting point: 144 – 146 °C.

***R*f** (toluene): 0.17.

^{1}H NMR (400 MHz, CD_2Cl_2): δ 7.59 – 7.55 (m, 2H), 7.53 (d, *J* = 7.9 Hz, 1H), 7.49 (d, *J* = 7.9 Hz, 1H), 7.46 – 7.38 (m, 3H), 7.30 – 7.24 (m, 2H), 7.19 – 7.14 (m, 2H), 6.79 (s, 2H).

^{13}C NMR (101 MHz, CD_2Cl_2): δ 185.9, 185.9, 143.9, 142.2, 142.0, 141.1, 139.0, 138.8, 136.8, 136.3, 131.5, 131.4, 131.2, 130.3, 128.5, 128.4, 127.6, 121.6.

IR (ATR, cm^{-1}): 1658 (s), 1321 (m), 1243 (m), 1101 (m), 1006 (m), 847 (m), 831 (m), 816 (m), 763 (m), 696 (m), 522 (m).

HRMS (ESI): calc. for $C_{22}H_{13}BrNaO_2^+$ $[M+Na]^+$: 410.9991; found: 411.0032.

*7.5.15 5-(4-Iodophenyl)-8-phenylnaphthalene-1,4-dione (**5s**)*

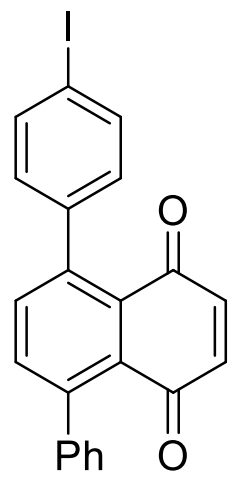

Obtained as a yellow amorphous solid according to GP7:

- 64.6 mg, 0.150 mmol, 1.0 equiv 5,8-dihydro-5-(4-iodophenyl)-8-phenylnaphthalene-1,4-dione **4s**
- 68 mg, 0.782 mmol, 10.5 equiv MnO_2
- 3.0 mL $CHCl_3$ as solvent
- Stirred at 62 °C for 20 h
- Purified by flash column chromatography (8 g SiO_2, toluene)

Yield: 46.5 mg (0.107 mmol, 72%).

Melting point: 133 – 135°C.

***R*f** (toluene): 0.17.

^{1}H NMR (400 MHz, CD_2Cl_2): δ 7.81 – 7.74 (m, 2H), 7.53 (d, *J* = 7.8 Hz, 1H), 7.48 (d, *J* = 7.9 Hz, 1H), 7.46 – 7.38 (m, 3H), 7.30 – 7.24 (m, 2H), 7.07 – 7.01 (m, 2H), 6.79 (s, 2H).

^{13}C NMR (101 MHz, CD_2Cl_2): δ 185.9, 185.9, 143.9, 142.2, 141.9, 141.8, 139.0, 138.8, 137.5, 136.8, 136.3, 131.4, 131.2, 130.5, 128.5, 128.4, 127.6, 93.0.

IR (ATR, cm^{-1}): 1655 (s), 1320 (m), 1241 (m), 1101 (m), 1000 (m), 848 (m), 814 (m), 766 (m), 704 (m), 524 (m).

HRMS (ESI): calc. for $C_{22}H_{13}INaO_2^+$ $[M+Na]^+$: 458.9852; found: 458.9857.

*7.5.16 5-(4-Trifluoromethylphenyl)-8-phenylnaphthalene-1,4-dione (**5t**)*

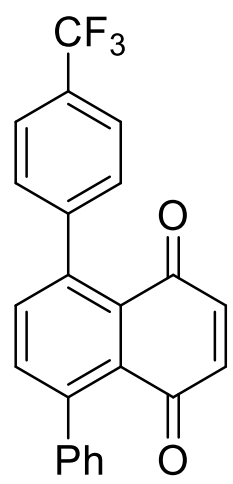

Obtained as a yellow amorphous solid according to GP7:

- 60.6 mg, 0.159 mmol, 1.0 equiv 5,8-dihydro-5-(4-trifluoromethylphenyl)-8-phenylnaphthalene-1,4-dione **4t**
- 90 mg, 1.04 mmol, 6.5 equiv MnO_2
- 1.5 mL $CHCl_3$ as solvent
- Stirred at 62 °C for 15 h
- Purified by flash column chromatography (8 g SiO_2, toluene)

Yield: 54.9 mg (0.145 mmol, 91%).

Melting point: 132 – 134°C.

***R*f** (toluene): 0.19.

^{1}H NMR (400 MHz, CD_2Cl_2): δ 7.71 (d, *J* = 7.9 Hz, 2H), 7.57 (d, *J* = 7.8 Hz, 1H), 7.50 (d, *J* = 7.8 Hz, 1H), 7.48 – 7.39 (m, 5H), 7.31 – 7.26 (m, 2H), 6.84 – 6.76 (m, 2H).

^{13}C NMR (101 MHz, CD_2Cl_2): δ 185.8, 185.8, 146.2, 144.2, 141.9, 141.8, 139.2, 138.7, 136.9, 136.1, 131.4, 131.3, 129.4 (d, *J* = 32.4 Hz), 129.0, 128.5, 128.5, 127.6, 125.3 (q, *J* = 3.9 Hz), 124.9 (d, *J* = 271.7 Hz).

^{19}F NMR (377 MHz, CD_2Cl_2): δ -62.64.

IR (ATR, cm^{-1}): 2922 (w), 2853 (w), 1656 (s), 1320 (s), 1163 (m), 1126 (s), 1105 (s), 1066 (s), 834 (m), 827 (m), 707 (m).

HRMS (ESI): calc. for $C_{23}H_{13}F_3NaO_2^+$ $[M+Na]^+$: 401.0760; found: 401.0760.

*7.5.17 5-(4-Methyl benzoate)-8-phenylnaphthalene-1,4-dione (**5u**)*

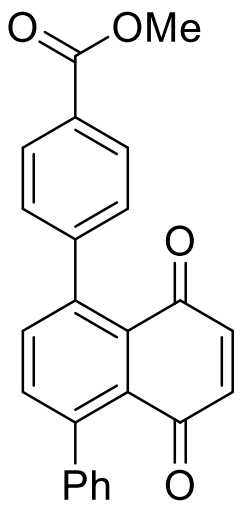

<u>Obtained as an orange amorphous solid according to GP7</u>:

- 57.5 mg, 0.155 mmol, 1.0 equiv 5,8-dihydro-5-(4-methyl benzoate)-8-phenylnaphthalene-1,4-dione **4u**
- 130 mg, 1.50 mmol, 9.6 equiv MnO_2
- 2.0 mL $CHCl_3$ as solvent
- Stirred at 62 °C for 17 h
- Purified by flash column chromatography (8 g SiO_2, 5% EtOAc in toluene)

Yield: 54.7 mg (0.148 mmol, 96%).

Melting point: 169 – 171 °C.

***R*f** (5% EtOAc in toluene): 0.26.

^{1}H NMR (400 MHz, CD_2Cl_2): 8.11 – 8.06 (m, 2H), 7.55 (d, *J* = 7.8 Hz, 1H), 7.51 (d, *J* = 7.9 Hz, 1H), 7.48 – 7.40 (m, 3H), 7.38 – 7.33 (m, 2H), 7.31 – 7.26 (m, 2H), 6.79 (d, *J* = 1.1 Hz, 2H), 3.94 (s, 3H).

^{13}C NMR (101 MHz, CD_2Cl_2): δ 185.8, 167.1, 147.0, 144.0, 142.4, 141.9, 139.1, 138.7, 136.8, 136.1, 131.3, 131.3, 129.6, 129.4, 128.6, 128.5, 128.5, 127.6, 52.4.

IR (ATR, cm^{-1}): δ1704 (s), 1660 (s), 1279 (s), 1246 (s), 1098 (s), 847 (s), 767 (s), 703 (s), 523 (m).

HRMS (ESI): calc. for $C_{24}H_{16}NaO_4^+$ $[M+Na]^+$: 391.0941; found: 391.0949.

*7.5.18 5-(4-Nitrophenyl)-8-phenylnaphthalene-1,4-dione (**5w**)*

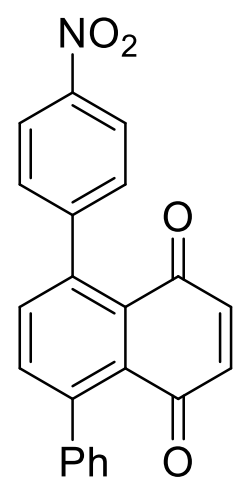

<u>Obtained as an orange amorphous solid according to GP7</u>:

- 57.7 mg, 0.161 mmol, 1.0 equiv 5,8-dihydro-5-(4-nitrophenyl)-8-phenylnaphthalene-1,4-dione **4w**
- 73 mg, 0.840 mmol, 5.2 equiv MnO_2
- 2.0 mL $CHCl_3$ as solvent
- Stirred at 62 °C for 17 h
- Purified by flash column chromatography (8 g SiO_2, 5% EtOAc in toluene)

Yield: 55.3 mg (0.156 mmol, 96%).

Melting point: 207 – 208°C.

***R*f** (5% EtOAc in toluene): 0.38.

^{1}H NMR (400 MHz, CD_2Cl_2): δ 8.29 (d, *J* = 8.8 Hz, 2H), 7.59 (d, *J* = 7.8 Hz, 1H), 7.50 (d, *J* = 7.9 Hz, 1H), 7.48 – 7.39 (m, 5H), 7.31 – 7.26 (m, 2H), 6.86 – 6.77 (m, 2H).

^{13}C NMR (101 MHz, CD_2Cl_2): δ 185.7, 185.6, 149.3, 147.5, 144.6, 141.6, 141.1, 139.4, 138.5, 137.0, 135.7, 131.3, 131.2, 129.5, 128.5, 128.5, 127.7, 123.7.

IR (ATR, cm^{-1}): 1656 (m), 1506 (s), 1341 (s), 1322 (s), 1242 (m), 1101 (m), 862 (m), 850 (s), 832 (m), 766 (m9, 755 (s) 524 (m).

HRMS (ESI): calc. for $C_{22}H_{13}NNaO_4^+$ $[M+Na]^+$: 378.0737; found: 378.0752.

*7.5.19 5-Cyclohexyl-8-phenylnaphthalene-1,4-dione (**5y**)*

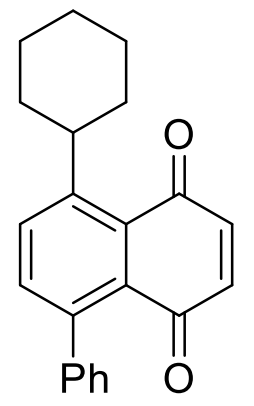

Obtained as a yellow amorphous solid according to GP7:

- 24.9 mg, 0.078 mmol, 1.0 equiv 5,8-dihydro-5-cyclohexyl-8-phenylnaphthalene-1,4-dione **4y**
- 70 mg, 0.805 mmol, 10.3 equiv MnO_2
- 1.0 mL $CHCl_3$ as solvent
- Stirred at 62 °C for 24 h
- Purified by flash column chromatography (8 g SiO_2, 10% cyclohexane in toluene)

Yield: 15.3 mg (0.048 mmol, 62%).

R

$\boldsymbol{R_f}$ (10% cyclohexane in toluene): 0.17.

^{1}H NMR (400 MHz, CD_2Cl_2): δ 7.73 (d, *J* = 8.3 Hz, 1H), 7.45 (d, *J* = 8.2 Hz, 1H), 7.42 – 7.33 (m, 3H), 7.24 – 7.17 (m, 2H), 6.84 (d, *J* = 10.1 Hz, 1H), 6.71 (d, *J* = 10.3 Hz, 1H), 3.94 – 3.83 (m, 1H), 1.97 – 1.77 (m, 5H), 1.63 – 1.43 (m, 4H), 1.39 – 1.29 (m, 1H).

^{13}C NMR (101 MHz, CD_2Cl_2): δ 188.2, 186.6, 150.8, 142.7, 141.8, 139.7, 138.1, 137.1, 132.3, 131.6, 131.0, 128.5, 128.3, 127.2, 39.9, 34.6, 27.5, 26.7.

IR (ATR, cm^{-1}): 2919 (m), 1655 (s), 1444 (m), 1312 (m), 1257 (m), 1082 (m), 846 (m), 839 (m), 764 (m), 756 (m), 737 (m), 700 (s), 660 (m).

HRMS (ESI): calc. for $C_{22}H_{20}NaO_2^+$ $[M+Na]^+$: 339.1356; found: 339.1357.

*7.5.20 5,8-Di(4-methylphenyl)naphthalene-1,4-dione (**5z**)*

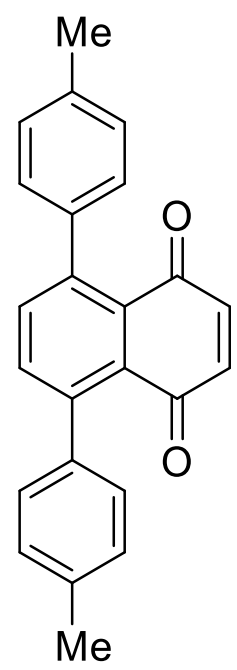

Obtained as an orange amorphous solid according to GP7:

- 38.6 mg, 0.113 mmol, 1.0 equiv 5,8-dihydro-5,8-di(4-methylphenyl)naphthalene-1,4-dione **4z**
- 98 mg, 1.13 mmol, 9.9 equiv MnO_2
- 3.0 mL $CHCl_3$ as solvent
- Stirred at 62 °C for 19 h
- Purified by flash column chromatography (8 g SiO_2 1% EtOAc in toluene)

Yield: 29.9 mg (0.088 mmol, 78%).

Melting point: 175 – 176 °C.

$\boldsymbol{R_f}$ (toluene): 0.10.

^{1}H NMR (400 MHz, CD_2Cl_2): δ 7.49 (s, 2H), 7.25 (d, *J* = 7.8 Hz, 4H), 7.16 (d, *J* = 8.2 Hz, 4H), 6.77 (s, 2H), 2.43 (s, 6H).

^{13}C NMR (101 MHz, CD_2Cl_2): δ 186.2, 143.4, 139.1, 138.9, 137.4, 136.7, 131.5, 129.1, 128.5, 21.4.

IR (ATR, cm^{-1}): 1659 (m), 1619 (m), 1320 (m), 1238 (m), 1100 (m), 849 (m), 831 (m), 810 (s), 526 (s), 425 (m).

HRMS (ESI): calc. for $C_{24}H_{18}KO_2^+$ $[M+K]^+$: 377.0938; found: 377.0937.

*7.5.21 5,8-Di(4-bromophenyl)naphthalene-1,4-dione (**5aa**)*

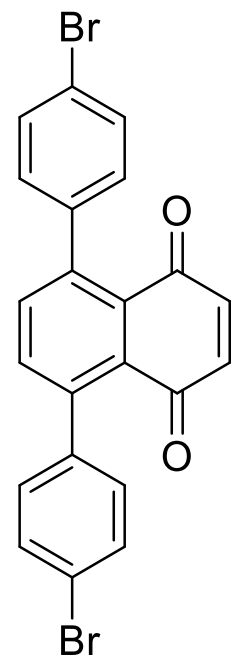

<u>Obtained as an orange amorphous solid according to GP7</u>:

- 66.3 mg, 0.141 mmol, 1.0 equiv 5,8-dihydro-5,8-di(4-bromophenyl)naphthalene-1,4-dione **4aa**
- 92 mg, 1.06 mmol, 7.5 equiv MnO_2
- 3.0 mL $CHCl_3$ as solvent
- Stirred at 62 °C for 19 h
- Purified by flash column chromatography (8 g SiO_2, toluene)

Yield: 61.7 mg (0.132 mmol, 94%).

Melting point: 224 – 225 °C.

***R*f** (toluene): 0.20.

^{1}H NMR (400 MHz, CD_2Cl_2): δ 7.61 – 7.53 (m, 4H), 7.50 (s, 2H), 7.19 – 7.13 (m, 4H), 6.79 (s, 2H).

^{13}C NMR (101 MHz, CD_2Cl_2): δ 185.8, 142.5, 141.0, 138.9, 136.5, 131.6, 131.2, 130.3, 121.6.

IR (ATR, cm^{-1}): 1664 (m), 1316 (m), 1240 (m), 1093 (m), 1065 (m), 1003 (m), 852 (m), 831 (m), 811 (s), 521 (m).

HRMS (ESI): calc. for $C_{22}H_{12}Br_2NaO_2^+$ $[M+Na]^+$: 490.9076; found: 490.9071.

*7.5.22 5-Ethoxycarbonyl-8-phenylnaphthalene-1,4-dione (**5ad**)*

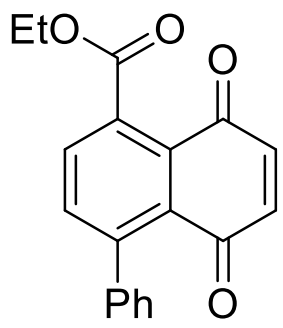

Under an inert gas atmosphere in a pear-shaped flask, 41.3 mg ethyl (2*E*,4*E*)-5-phenyl-2,4-pentadienoate **1ad** (0.204 mmol, 1.0 equiv.) and 88.6 mg *p*-benzoquinone (0.820 mmol, 4.0 equiv) were dissolved in 2.0 mL anhydrous DCM and cooled with an ice-water bath to 0 °C. Then 0.448 µL TfOH (0.8 mg, 0.005 mmol, 2.5 mol%) was added and the mixture was stirred for 20 min at 0 °C. The cooling bath was removed and the mixture was stirred for an additional 15 h. Then 1.5 mL sat. $NaHCO_3$ was added, together with 2 mL DCM/EtOAc (3:1). The phases were separated and the aqueous phase was extracted with DCM (3 x 4 mL). The combined organic phases were dried over $MgSO_4$, filtered and the solvent was removed under reduced pressure. Under an inert atmosphere, the obtained residue was taken up in 2 mL $CHCl_3$ and 200 mg MnO_2 (2.30 mmol, 11.2 equiv) was added. The suspension was heated to 62 °C in an oil bath and stirred for 17 h. After cooling to r.t. Celite® was added and the solvent was removed under reduced pressure. The residue was purified by flash column chromatography (8 g SiO_2, 2.5 mol% EtOAc in toluene) to yield the product as an orange amorphous solid.

Yield: 45.6 mg (0.148 mmol, 72%).

Melting point: 139 – 141 °C.

***R*f** (30% Et_2O in *n*-pentane): 0.16.

^{1}H NMR (400 MHz, CD_2Cl_2): δ 7.65 (d, *J* = 7.9 Hz, 1H), 7.59 (d, *J* = 7.9 Hz, 1H), 7.47 – 7.38 (m, 3H), 7.27 – 7.21 (m, 2H), 6.94 (d, *J* = 10.3 Hz, 1H), 6.83 (d, *J* = 10.3 Hz, 1H), 4.44 (q, *J* = 7.1 Hz, 2H), 1.39 (t, *J* = 7.2 Hz, 3H).

^{13}C NMR (101 MHz, CD_2Cl_2): δ 184.9, 184.6, 169.3, 145.2, 141.1, 140.3, 137.6, 134.6, 131.5, 131.0, 130.1, 128.5, 127.9, 62.4, 14.2.

IR (ATR, cm^{-1}): 1720 (m), 1663 (s), 1265 (m), 1250 (m), 1142 (m), 1107 (m), 1075 (m), 842 (m), 757 (m), 699 (m).

HRMS (ESI): calc. for $C_{19}H_{14}NaO_4^+$ $[M+Na]^+$: 329.0784; found: 329.0788.

*7.5.23 5-Biphenylnaphthalene-1,4-dione (**5ae**)*

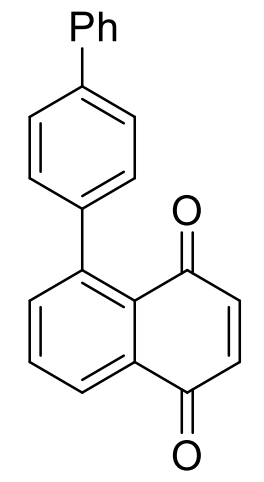

Under an inert gas atmosphere in a pear-shaped flask, 41.6 mg 1,1'-biphenyl-4-(1*E*)-1,3-butadien-1-yl **1ae** (0.202 mmol, 1.0 equiv.) was dissolved in 1.5 mL anhydrous DCM and taken up into a syringe. In the meantime, 88.9 mg *p*-benzoquinone (0.822 mmol, 4.1 equiv) was dissolved in 0.75 mL anhydrous DCM and cooled with an ice-water bath to 0 °C. Then 0.438 µL TfOH (0.8 mg, 0.005 mmol, 2.5mol%) was added to the *p*-benzoquinone solution, followed by the dropwise addition of diene by syringe pump in 15 min (6 mL/h). After complete addition the reaction was stirred at r.t. for 15 min. Then 1.5 mL sat. $NaHCO_3$ was added, together with 2 mL DCM/EtOAc (3:1). The phases were separated and the aqueous phase was extracted with DCM (3 x 4 mL). The combined organic phases were dried over $MgSO_4$, filtered and the solvent was removed under reduced pressure. Under an inert atmosphere, the obtained residue was taken up in 2 mL $CHCl_3$ and 175 mg MnO_2 (2.01 mmol, 10.0 equiv) was added. The suspension was heated to 62 °C in an oil bath and stirred for 17 h. After cooling to r.t. Celite® was added and the solvent was removed under reduced pressure. The residue was purified by flash column chromatography (9 g SiO_2, toluene) to yield the product as an orange amorphous solid.

Yield: 42.3 mg (0.136 mmol, 68%).

Melting point: 142 – 144 °C.

$\boldsymbol{R_f}$ (toluene): 0.22.

^{1}H NMR (400 MHz, CD_2Cl_2): δ 8.16 (dd, *J* = 7.7, 1.5 Hz, 1H), 7.77 (t, *J* = 7.7 Hz, 1H), 7.72 – 7.65 (m, 4H), 7.61 (dd, *J* = 7.6, 1.4 Hz, 1H), 7.52 – 7.45 (m, 2H), 7.40 – 7.37 (m, 1H), 7.37 – 7.33 (m, 2H), 6.95 (d, *J* = 10.3 Hz, 1H), 6.85 (d, *J* = 10.3 Hz, 1H).

^{13}C NMR (101 MHz, CD_2Cl_2): δ 185.7, 185.4, 143.5, 141.1, 140.7, 140.7, 140.4, 137.9, 137.4, 133.8, 133.2, 129.6, 129.3, 129.2, 127.8, 127.4, 127.0, 126.7.

IR (ATR, cm^{-1}):3051 (w), 3027 (w), 2921 (w), 2851 (w), 1660 (s), 1610 (m), 1326 (m), 1281 (m), 1265 (m), 1251 (m), 1112 (m), 1087 (m), 827 (m), 790 (m), 762 (m), 727 (m), 691 (m).

HRMS (ESI): calc. for $C_{22}H_{14}NaO_2^+$ $[M+Na]^+$: 333.0886; found: 333.0886.

*7.5.24 5-(4-Methylphenyl)naphthalene-1,4-dione (**5af**)*

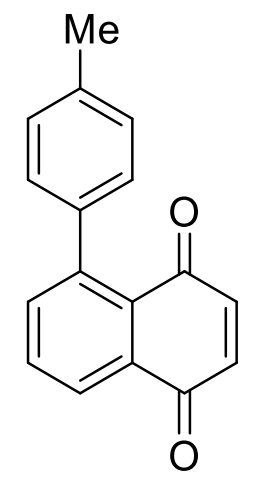

Under an inert gas atmosphere in a pear-shaped flask, 41.6 mg 1,1'-biphenyl-4-(1*E*)-1,3-butadien-1-yl **1af** (0.202 mmol, 1.0 equiv.) was dissolved in 1.5 mL anhydrous DCM and taken up into a syringe. In the meantime, 83.9 mg *p*-benzoquinone (0.822 mmol, 4.1 equiv) was dissolved in 0.75 mL anhydrous DCM and cooled with an ice-water bath to 0 °C. Then 0.438 µL TfOH (0.8 mg, 0.005 mmol, 2.5mol%) was added to the *p*-benzoquinone solution, followed by the dropwise addition of diene by syringe pump in 15 min (6 mL/h). After complete addition the reaction was stirred at r.t. for 15 min. Then 1.5 mL sat. $NaHCO_3$ was added, together with 2 mL DCM/EtOAc (3:1). The phases were separated and the aqueous phase was extracted with DCM (3 x 4 mL). The combined organic phases were dried over $MgSO_4$, filtered and the solvent was removed under reduced pressure. Under an inert atmosphere, the obtained residue was taken up in 2 mL $CHCl_3$ and 175 mg MnO_2 (2.01 mmol, 10.0 equiv) was added. The suspension was heated to 62 °C in an oil bath and stirred for 17 h. After cooling to r.t. Celite® was added and the solvent was removed under reduced pressure. The residue was purified by flash column chromatography (9 g SiO_2, toluene) to yield the product as an orange amorphous solid.

Yield: 29.7 mg (0.120 mmol, 62%).

Melting point: 146 – 148 °C.

$\boldsymbol{R_f}$ (toluene): 0.20.

^{1}**H NMR** (400 MHz, CD_2Cl_2): δ 8.12 (dd, *J* = 7.7, 1.3 Hz, 1H), 7.73 (t, *J* = 7.7 Hz, 1H), 7.54 (dd, *J* = 7.6, 1.4 Hz, 1H), 7.23 (d, *J* = 8.4 Hz, 2H), 7.14 (d, *J* = 8.2 Hz, 2H), 6.92 (d, *J* = 10.3 Hz, 1H), 6.81 (d, *J* = 10.3 Hz, 1H), 2.43 (s, 3H).

^{13}**C NMR** (101 MHz, CD_2Cl_2): δ 185.7, 185.5, 144.0, 140.7, 138.6, 138.0, 137.5, 137.3, 133.8, 133.1, 129.6, 129.0, 128.6, 126.4, 21.4.

IR (ATR, cm^{-1}): 1660 (s), 1611 (m), 1325 (m), 1281 (m), 1258 (m), 860 (m), 839 8m), 812 (m), 789 (s), 525 (m), 445 (m).

HRMS (ESI): calc. for $C_{17}H_{12}NaO_2^+$ $[M+Na]^+$: 271.0730; found: 271.0732.

*7.5.25 2-Methyl-5,8-diphenylnaphthalene-1,4-dione (**5ah**)*

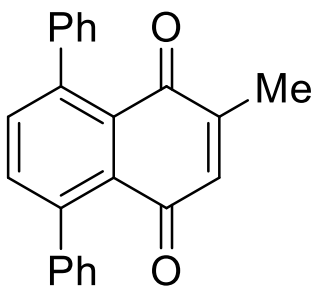

Obtained as a yellow amorphous solid according to GP7:

- 58.0 mg, 0.178 mmol, 1.0 equiv 5,8-dihydro-2-methyl-5,8-diphenylnaphthalene-1,4-dione **4ah**
- 165 mg, 1.90 mmol, 10.7 equiv MnO_2
- 1.5 mL $CHCl_3$ as solvent
- Stirred at 62 °C for 19 h
- Purified by flash column chromatography (8 g SiO_2, toluene)

Yield: 47.6 mg (0.147 mmol, 83%).

Melting point:102 – 104 °C.

R_f (toluene): 0.13.

^{1}**H NMR** (400 MHz, CD_2Cl_2): δ 7.48 (s, 2H), 7.47 – 7.37 (m, 6H), 7.30 – 7.23 (m, 4H), 6.65 (q, *J* = 1.5 Hz, 1H), 2.02 (d, *J* = 1.6 Hz, 3H).

^{13}**C NMR** (101 MHz, CD_2Cl_2): δ 186.6, 185.8, 148.2, 143.6, 143.2, 142.6, 142.3, 136.3, 136.3, 136.0, 131.7, 131.6, 128.6, 128.4, 128.4, 128.3, 127.4, 127.3, 16.3.

IR (ATR, cm^{-1}): 1659 (s), 1319 (m), 1220 (m), 1150 (m), 1010 (m), 952 (m), 756 (s), 696 (s), 595 (m), 508 (m).

HRMS (ESI): calc. for $C_{23}H_{16}NaO_2^+$ $[M+Na]^+$: 347.1043; found: 347.1044.

*7.5.26 2,3-Dimethyl-5,8-diphenylnaphthalene-1,4-dione (**5ai**)*

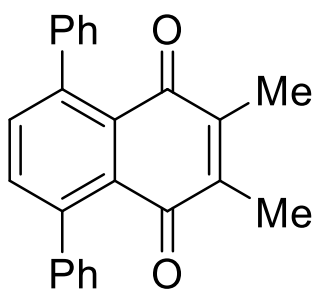

Obtained as an orange amorphous solid according to GP7:

- 44.9 mg, 0.132 mmol, 1.0 equiv 5,8-dihydro-2,3-dimethyl-5,8-diphenylnaphthalene-1,4-dione **4ai**
- 116 mg, 1.33 mmol, 10.1 equiv MnO_2
- 1.5 mL $CHCl_3$ as solvent
- Stirred at 62 °C for 23 h
- Purified by flash column chromatography (8 g SiO_2, toluene)

Yield: 36.5 mg (0.108 mmol, 82%).

Melting point: 163 – 165 °C.

R_f (toluene): 0.19.

^{1}**H NMR** (400 MHz, CD_2Cl_2): δ 7.46 – 7.36 (m, 8H), 7.29 – 7.23 (m, 4H), 2.01 (s, 6H).

^{13}**C NMR** (101 MHz, CD_2Cl_2): δ 186.1, 143.6, 143.1, 142.8, 135.9, 131.8, 128.5, 128.3, 127.2, 13.0.

IR (ATR, cm^{-1}): 2923 (w), 1648 (s), 1634 (s), 1444 (m), 1377 (m), 1319 (s), 1207 (m), 1091 (m), 797 (m), 755 (s), 739 (m), 694 (m), 500 (m), 424 (m).

HRMS (ESI): calc. for $C_{24}H_{18}NaO_2^+$ $[M+Na]^+$: 361.1199; found: 361.1201.

*7.5.27 2-Cyclopropyl-5,8-diphenylnaphthalene-1,4-dione (**5aj**)*

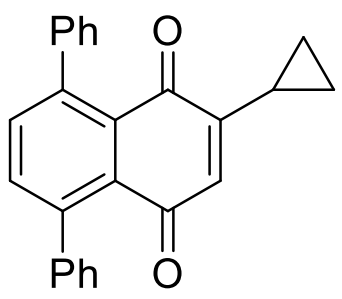

Obtained as an orange amorphous solid according to GP7:

- 41.5 mg, 0.118 mmol, 1.0 equiv 5,8-dihydro-2-cyclopropyl-5,8-diphenylnaphthalene-1,4-dione **4aj**
- 106 mg, 1.22 mmol, 10.4 equiv MnO_2
- 3.0 mL $CHCl_3$ as solvent
- Stirred at 62 °C for 17 h
- Purified by flash column chromatography (8 g SiO_2, toluene)

Yield: 32.3 mg (0.092 mmol, 78%).

Melting point: 135 – 137 °C.

***R*f** (toluene): 0.16.

^{1}H NMR (400 MHz, CD_2Cl_2): δ 7.48 (s, 2H), 7.47 – 7.35 (m, 6H), 7.33 – 7.24 (m, 4H), 6.17 (d, *J* = 0.9 Hz, 1H), 2.03 (ttd, *J* = 8.4, 5.2, 0.7 Hz, 1H), 1.11 – 1.00 (m, 2H), 0.79 – 0.72 (m, 2H).

^{13}C NMR (101 MHz, CD_2Cl_2): δ 186.0, 185.7, 154.5, 143.6, 143.1, 142.6, 142.3, 136.4, 136.1, 132.1, 131.4, 129.0, 128.6, 128.5, 128.4, 128.3, 127.4, 127.3, 10.9, 9.6.

IR (ATR, cm^{-1}): 1652 (s), 1625 (m), 1457 (w), 1443 (w), 1378 (w), 1321 (m) 1230 (m), 1140 (m), 1021 (m), 954 (m), 921 (m), 853 (m), 844 (m), 754 (s), 692 (s), 669 (m), 598 (m), 506 (m).

HRMS (ESI): calc. for $C_{25}H_{18}NaO_2^+$ $[M+Na]^+$: 373.1199; found: 373.1177.

*7.5.28 2-Cyclohexyl-5,8-diphenylnaphthalene-1,4-dione (**5ak**)*

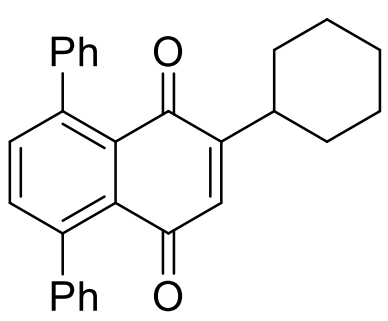

Obtained as a yellow amorphous solid according to GP7:

- 56.0 mg, 0.142 mmol, 1.0 equiv 5,8-dihydro-2-cyclohexyl-5,8-diphenylnaphthalene-1,4-dione **4ak**
- 120 mg, 1.38 mmol, 9.7 equiv MnO_2
- 3.0 mL $CHCl_3$ as solvent
- Stirred at 62 °C for 24 h
- Purified by flash column chromatography (8 g SiO_2, 10% cyclohexane in toluene)

Yield: 38.4 mg (0.098 mmol, 69%).

Melting point: 152 – 154 °C.

***R*f** (toluene): 0.38.

^{1}H NMR (400 MHz, CD_2Cl_2): δ 7.49 (d, *J* = 0.7 Hz, 2H), 7.47 – 7.37 (m, 6H), 7.32 – 7.24 (m, 4H), 6.53 (d, *J* = 1.2 Hz, 1H), 2.77 – 2.66 (m, 1H), 1.86 – 1.68 (m, 5H), 1.46 – 1.31 (m, 2H), 1.30 – 1.15 (m, 3H).

^{13}C NMR (101 MHz, CD_2Cl_2): δ 186.5, 186.3, 156.4, 143.5, 143.0, 142.6, 142.3, 133.2, 132.6, 131.3, 128.6, 128.5, 128.5, 128.3, 127.4, 127.3, 37.0, 32.3, 26.8, 26.4.

IR (ATR, cm^{-1}):2929 (m),1667 (m), 1654 (s), 1623 (m), 1445 (m), 1319 (m), 1214 (s), 1146 (m), 946 (m), 845 (m), 768 (m), 756 (m), 698 (s), 676 (m), 605 (m), 528 (m).

HRMS (ESI): calc. for $C_{28}H_{24}NaO_2^+$ $[M+Na]^+$: 415.1669; found: 415.1666.

*7.5.29 2,5,8-Triphenylnaphthalene-1,4-dione (**5al**)*

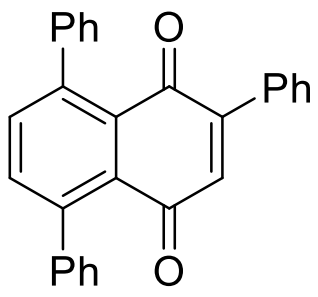

Obtained as an orange amorphous solid according to GP7:

- 55.2 mg, 0.142 mmol, 1.0 equiv 5,8-dihydro-2,5,8-triphenylnaphthalene-1,4-dione **4al**
- 120 mg, 1.45 mmol, 10.2 equiv MnO_2
- 1.5 mL $CHCl_3$ as solvent
- Stirred at 62 °C for 24 h
- Purified by flash column chromatography (8 g SiO_2, 10% cyclohexane in toluene)

Yield: 40.6 mg (0.111 mmol, 78%).

Melting point: 179 – 172 °C.

***R*f** (toluene): 0.28.

^{1}H NMR (400 MHz, CD_2Cl_2): δ 7.59 – 7.51 (m, 2H), 7.51 – 7.38 (m, 11H), 7.35 – 7.30 (m, 4H), 6.92 (s, 1H).

^{13}C NMR (101 MHz, CD_2Cl_2): δ 186.6, 185.9, 148.3, 143.7, 143.0, 142.3, 142.1, 136.5, 136.4, 134.8, 133.6, 133.0, 131.3, 130.3, 129.6, 128.8, 128.7, 128.6, 128.4, 127.5, 127.5.

IR (ATR, cm^{-1}):1657 (s), 1321 (m), 1206 (m), 1150 (m), 757 (s), 731 (m), 696 (s), 687 (s), 587 (m), 522 (m), 503 (m).

HRMS (ESI): calc. for $C_{28}H_{18}NaO_2^+$ [M+Na]$^+$: 409.1199; found: 409.1196.

*7.5.30 5,12-Dihydro-7,10-diphenyl-5,12[1',2']benzenonaphthacene-6,11-dione (**5am**)*

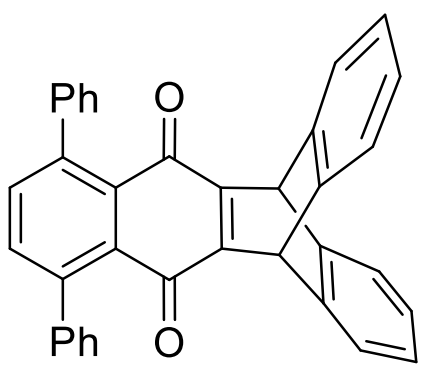

Obtained as an orange amorphous solid according to GP7:

- 85.7 mg, 0.175 mmol, 1.0 equiv 5,7,10,12-tetrahydro-7,10-diphenyl-5,12[1',2']benzenonaphthacene-6,11-dione **4am**
- 160 mg, 1.84 mmol, 10.2 equiv MnO_2
- 1.5 mL toluene as solvent
- Stirred at 110 °C for 24 h
- Purified by flash column chromatography (8 g SiO_2, 50% cyclohexane in toluene)

Yield: 57.1 mg (0.117 mmol, 67%).

Melting point: 332 – 335 °C.

***R*f** (toluene): 0.41.

^{1}H NMR (400 MHz, CD_2Cl_2): δ 7.46 – 7.35 (m, 12H), 7.25 – 7.17 (m, 4H), 7.04 – 6.99 (m, 4H), 5.81 (s, 2H).

^{13}C NMR (101 MHz, CD_2Cl_2): δ 182.1, 153.9, 144.4, 143.8, 142.7, 136.5, 130.9, 128.4, 128.3, 127.3, 125.8, 124.7, 48.1.

IR (ATR, cm^{-1}): 1655 (s), 1628 (m), 1456 (m), 1318 (m), 1201 (m), 1181 (m), 755 (s), 748 (s), 731 (m), 695 (s), 598 (s), 541 (m), 522 (m).

HRMS (ESI): calc. for $C_{36}H_{22}NaO_2^+$ [M+Na]$^+$: 509.1512; found: 509.1506.

*7.5.31 2-Chloro-5,8-diphenylnaphthalene-1,4-dione (**5an**)*

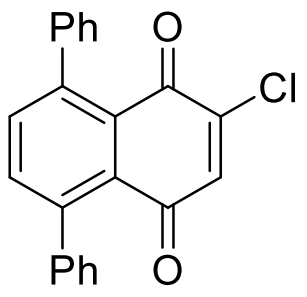

Obtained as an orange amorphous solid according to GP7:

- 30.6 mg, 0.088 mmol, 1.0 equiv 5,8-dihydro-2-chloro-5,8-diphenylnaphthalene-1,4-dione **4an**
- 82 mg, 0.943 mmol, 10.7 equiv MnO_2
- 1.5 mL $CHCl_3$ as solvent
- Stirred at 62 °C for 24 h
- Purified by flash column chromatography (8 g SiO_2, toluene)

Yield: 23.5 mg (0.068 mmol, 77%).

Melting point: 146 – 148 °C.

***R*f** (toluene): 0.29.

^{1}H NMR (400 MHz, CD_2Cl_2): δ 7.59 – 7.52 (m, 2H), 7.49 – 7.38 (m, 6H), 7.30 – 7.24 (m, 4H), 7.05 (s, 1H).

^{13}C NMR (101 MHz, CD_2Cl_2): δ 183.5, 178.8, 145.7, 144.6, 144.0, 141.8, 141.7, 137.3, 136.8, 136.4, 131.1, 130.5, 128.6, 128.5, 128.5, 128.3, 127.7, 127.7.

IR (ATR, cm^{-1}): 1678 (m), 1660 (s), 1320 (m), 1205 (m), 885 (m), 864 (m), 757 (s), 739 (m), 729 (m), 696 (s), 532 (m).

HRMS (ESI): calc. for $C_{22}H_{13}ClNaO_2^+$ $[M+Na]^+$: 367.0496; found: 367.0484.

*7.5.32 2-Acetamido-5,8-diphenylnaphthalene-1,4-dione (**5ao**)*

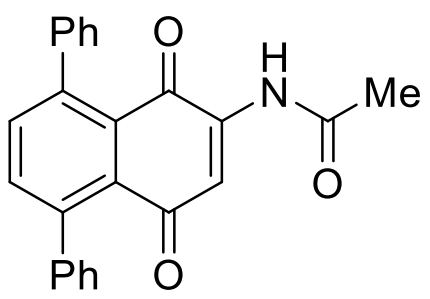

Obtained as an orange amorphous solid according to GP7:

- 41.4 mg, 0.112 mmol, 1.0 equiv 5,8-dihydro-2-acetammido-5,8-diphenylnaphthalene-1,4-dione **4ao**
- 96 mg, 1.10 mmol, 9.9 equiv MnO_2
- 1.5 mL THF as solvent
- Stirred at 65 °C for 24 h
- Purified by flash column chromatography (8 g SiO_2, 20% EtOAc in toluene)

Yield: 35.4 mg (0.096 mmol, 86%).

Melting point: 206 – 209 °C.

***R*f** (20% EtOAc in toluene): 0.39.

^{1}H NMR (400 MHz, CD_2Cl_2): δ 8.18 (s, 1H), 7.58 – 7.53 (m, 2H), 7.48 (d, J = 7.9 Hz, 1H), 7.46 – 7.36 (m, 6H), 7.30 – 7.25 (m, 4H), 2.14 (s, 3H).

^{13}C NMR (101 MHz, CD_2Cl_2): δ 186.1, 181.6, 169.8, 144.2, 143.5, 142.3, 140.0, 137.8, 136.0, 131.6, 129.0, 128.5,128.4, 128.3, 127.6, 127.4, 117.6, 25.1.

IR (ATR, cm^{-1}): 3290 (w), 1714 (m), 1669 (m), 1644 (m), 1630 (m), 1497 (s), 1328 (m), 1220 (m), 1194 (m), 1150 (m), 753 (m), 694 (s), 516 (m).

HRMS (ESI): calc. for $C_{24}H_{17}NNaO_3^+$ $[M+Na]^+$: 390.1101; found: 390.1103.

7.6 Synthesis and Analytical Data of 1,4,4a,8a-Tetrahydro-1,4-ethanonaphthalene-5,8-diones (6)

*7.6.1 1,4,4a,8a-Tetrahydro-1,4-ethanonaphthalene-5,8-dione (**6a**)*

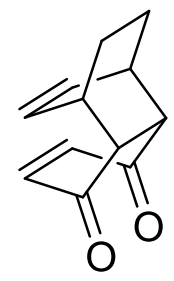

Obtained as a white amorphous solid according to GP6 with TfOH:

- 78.1 mg, 0.975 mmol, 1.0 equiv cyclohexa-1,3-dien
- 422 mg, 3.90 mmol, 4.0 equiv *p*-benzoquinone **2a**
- 2.140 µL, 3.7 mg, 0.024 mmol, 2.5 mol% TfOH
- cyclohexa-1,3-dien dissolved in 2.5 mL anhydrous DCM and added *via* syringe pump in 15 min (10 mL/h) to *p*-benzoquinone and TfOH dissolved in 7.5 mL anhydrous DCM at 0 °C
- After complete addition, stirred for another 5 min
- Purified by flash column chromatography (20 g SiO_2, 2% EtOAc in toluene)

Yield: 134.7 mg (0.716 mmol, 73%).

Melting point: 92 – 93 °C.

***R*f** (30% Et_2O in *n*-pentane): 0.15.

^{1}H NMR (400 MHz, $CDCl_3$): δ 6.63 (s, 2H), 6.21 (dd, *J* = 4.5, 3.1 Hz, 2H), 3.24 – 3.18 (m, 2H), 2.98 (s, 2H), 1.76 – 1.66 (m, 2H), 1.40 – 1.33 (m, 2H)

^{13}C NMR (101 MHz, $CDCl_3$): δ 199.5, 142.1, 133.6, 49.5, 35.5, 24.9.

IR (ATR, cm^{-1}): 1662 (s), 1611 (m), 1276 (m), 1257 (m), 1107 (m), 1044 (m), 876 (m), 721 (m), 710 (s), 417 (m).

HRMS (ESI): calc. for $C_{12}H_{12}NaO_2^+$ $[M+Na]^+$: 211.0730; found: 211.0729.

Spectral data are in accordance with those reported in the literature.[28]

*7.6.2 1,4,4a,8a-Tetrahydro-6-methyl-1,4-ethanonaphthalene-5,8-dione (**6b**)*

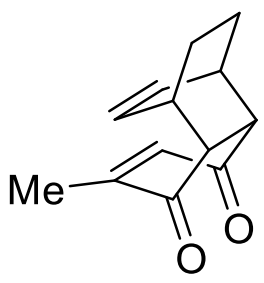

Obtained as a white amorphous solid according to GP5 with TfOH:

- 13.7 mg, 0.171 mmol, 1.0 equiv cyclohexa-1,3-diene
- 42.0 mg, 0.344 mmol, 2.0 equiv 2-methyl-1,4-benzoquinone **2b**
- 0.374 µL, 0.6 mg, 0.004 mmol, 2.5 mol% TfOH
- 2.0 mL anhydrous DCM as solvent
- Stirred at 0 °C for 15 min
- Purified by flash column chromatography (8 g SiO_2, 30% Et_2O in *n*-pentane)

Yield: 25.3 mg (0.125 mmol, 73%).

***R*f** (30% Et_2O in *n*-pentane): 0.23

^{1}H NMR (400 MHz, $CDCl_3$): δ 6.54 (q, *J* = 1.5 Hz, 1H), 6.23 – 6.13 (m, 2H), 3.23 – 3.14 (m, 2H), 2.96 (s, 2H), 1.93 (d, *J* = 1.5 Hz, 3H), 1.75 – 1.61 (m, 2H), 1.44 – 1.29 (m, 2H).

^{13}C NMR (101 MHz, $CDCl_3$): δ 199.8, 199.2, 151.8, 139.8, 133.8, 133.3, 50.0, 49.4, 35.7, 35.4, 24.9, 16.8.

IR (ATR, cm^{-1}): 1673 (m), 1648 (s), 1626 (m), 1258 (m), 1219 (m), 1193 (m), 1175 (m), 911 (m), 899 (m), 859 (m), 717 (m), 677 (m), 459 (m).

HRMS (ESI): calc. for $C_{13}H_{14}NaO_2^+$ $[M+Na]^+$: 225.0886; found: 225.0883.

Spectral data are in accordance with those reported in the literature.[28]

*7.6.3 1,4,4a,8a-Tetrahydro-4a,7-dimethyl-1,4-ethanonaphthalene-5,8-dione (**6c**)*

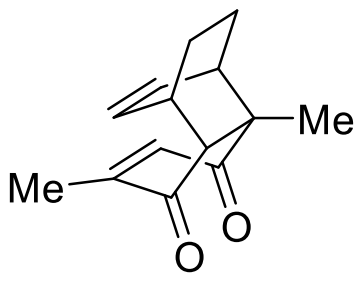

Obtained as a white amorphous solid according to GP6 with TfOH:

- 16.0 mg, 0.199 mmol, 1.0 equiv cyclohexa-1,3-dien
- 33.9 mg, 0.249 mmol, 1.3 equiv 2,5-dimethyl-1,4-benzoquinone **2i**
- 0.436 µL, 0.8 mg, 0.005 mmol, 2.5 mol% TfOH
- cyclohexa-1,3-dien dissolved in 0.5 mL anhydrous DCM and added *via* syringe pump in 30 min (1 mL/h) to 2,5-dimethyl-1,4-benzoquinone and TfOH dissolved in 1.5 mL anhydrous DCM at 0 °C
- After complete addition, stirred for another 5 min
- Purified by flash column chromatography (10 g SiO_2, 10% Et_2O in *n*-pentane)

Yield: 29.4 mg (0.136 mmol, 68%).

Melting point: 61 – 62 °C.

***R*f** (20% Et_2O in *n*-pentane): 0.26

^{1}H NMR (400 MHz, $CDCl_3$): δ 6.50 (q, *J* = 1.5 Hz, 1H), 6.28 (ddd, *J* = 8.1, 6.5, 1.5 Hz, 1H), 6.09 (ddd, *J* = 7.9, 6.2, 1.3 Hz, 1H), 3.03 – 2.98 (m, 1H), 2.92 – 2.87 (m, 1H), 2.50 (d, *J* = 2.9 Hz, 1H), 1.93 (d, *J* = 1.5 Hz, 3H), 1.91 – 1.81 (m, 1H), 1.78 – 1.68 (m, 1H), 1.39 – 1.33 (m, 1H), 1.31 (s, 3H), 1.24 – 1.14 (m, 1H).

^{13}C NMR (101 MHz, $CDCl_3$): δ 203.0, 200.6, 150.3, 139.3, 136.6, 132.1, 58.4, 51.1, 39.3, 37.2, 26.4, 26.1, 18.9, 16.5.

IR (ATR, cm^{-1}): 2954 (m), 1660 (s), 1633 (m), 1260 (m), 1236 (m), 1215 (m), 1139 (m), 894 (m), 720 (m), 680 (m).

HRMS (ESI): calc. for $C_{14}H_{16}NaO_2^+$ $[M+Na]^+$: 239.1043; found: 239.1044.

Spectral data are in accordance with those reported in the literature.[29]

*7.6.4 1,4,4a,8a-Tetrahydro-6-acetamido-1,4-ethanonaphthalene-5,8-dione (**6d**)*

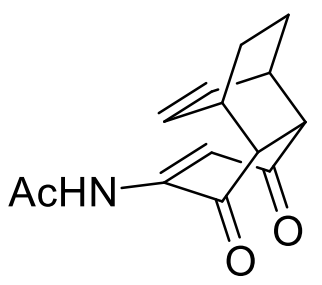

Obtained as a white amorphous solid according to GP5 with TfOH:

- 39.8 mg, 0.204 mmol, 1.0 equiv 2-acetamido-1,4-benzoquinone **2h**
- 24.5 mg, 0.306 mmol, 1.5 equiv cyclohexa-1,3-diene
- 18.0 µL, 30.8 mg, 0.205 mmol, 1.0 equiv TfOH
- 2.0 mL anhydrous DCM as solvent
- Stirred at 0 °C for 15 min
- Purified by flash column chromatography (8 g SiO_2, AcMe in DCM gradient: starting with two CVs DCM, then 0.5% steps to 2.5% each step two CVs, then 2.5% AcMe in DCM)

Yield: 42.2 mg (0.172 mmol, 84%).

Melting point: 208 – 210 °C.

^{1}H NMR (400 MHz, $CDCl_3$): δ 8.09 (br, 1H), 7.65 (s, 1H), 6.25 (ddd, *J* = 7.9, 6.4, 1.4 Hz, 1H), 6.17 – 6.11 (m, 1H), 3.27 – 3.19 (m, 2H), 3.06 (dd, *J* = 9.1, 2.6 Hz, 1H), 2.95 (dd, *J* = 9.1, 2.5 Hz, 1H), 2.19 (s, 3H), 1.78 – 1.66 (m, 2H), 1.44 – 1.31 (m, 2H).

^{13}C NMR (101 MHz, $CDCl_3$): δ 199.2, 195.5, 169.4, 142.4, 134.6, 132.4, 122.1, 48.8, 48.5, 36.3, 35.7, 25.1, 24.9, 24.9.

IR (ATR, cm^{-1}): 1678 (s), 1639 (s), 1625 (m), 1519 (s), 1313 (m), 1298 (m), 1230 (m), 1216 (m), 1161 (m), 1137 (m), 1055 (m), 898 (m), 719 (m).

HRMS (ESI): calc. for $C_{14}H_{15}NNaO_3^+$ $[M+Na]^+$: 268.0944; found: 268.0947.

7.7 Synthesis and Analytical Data of 1,4,5,8-Tetrahydroanthracene-9,10-diones (8 and 6e)

*7.7.1 1,4,5,8-Tetrahydro-1,4,5,8-tetraphenylanthracene-9,10-dione (**8a**)*

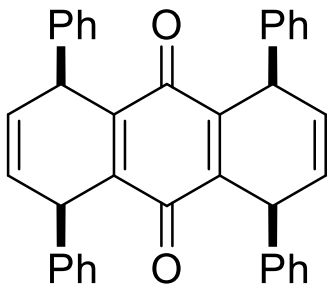

Under an inert gas atmosphere, 1109 mg 5,8-dihydro-5,8-diphenylnaphthalene-1,4-dione **4a** (3.55 mmol, 1.0 equiv) was dissolved in 18 mL anhydrous DCM and cooled to 0 °C. Then 7.80 µL TfOH (13.3 mg, 0.09 mmol, 2.5 mol%) was added, followed by the dropwise addition of 735 mg (1*E*,3*E*)-1,4-diphenyl-1,3-butadien **1a** (3.56 mmol, 1.00 equiv) in 18 mL anhydrous DCM (prepared under inert gas atmosphere in a pear-shaped flask) *via* syringe pump in 15 min (72 mL/h) at 0 °C. After the addition was complete, the mixture was stirred for 15 min. Then 386 mg *p*-benzoquinone (3.57 mmol, 1.0 equiv) was added and the mixture was stirred for 30 min. The reaction was ended by the addition of 40 mL sat. $NaHCO_3$ and transferred to a separatory funnel with 20 mL DCM. The phases were separated and the aqueous phase was extracted with DCM (3 x 40 mL). The combined organic phases were dried over $MgSO_4$, filtered and the solvent was removed under reduced pressure. The residue was adsorbed on Celite® and purified by flash column chromatography (SiO_2, 30% cyclohexane in toluene) to yield the product as a yellow amorphous solid.

Yield: 1308 mg (2.53 mmol, 71%).

Note: 430 mg of 5,8-dihydro-5,8-diphenylnaphthalene-1,4-dione **4a** (1.38 mmol) was reisolated.

*One-pot procedure starting from p-benzoquinone **2a**:*

Under an inert gas atmosphere, 206.8 mg (1*E*,3*E*)-1,4-diphenyl-1,3-butadien **1a** (1.00 mmol, 1.00 equiv) and *p*-benzoquinone (2.01 mmol 2.0 equiv) were dissolved in 2 mL anhydrous DCM. Then 6.5 µL MsOH (9.6 mg, 0.10 mmol, 0.1 equiv) was added and the reaction was stirred at r.t.. After 90 min, the mixture was diluted with 3 mL anhydrous DCM and cooled to 0 °C in 15 min. 4.40 µL TfOH (7.5 mg, 0.05 mmol, 5 mol%) was added, followed by the dropwise addition of 278.3 mg (1*E*,3*E*)-1,4-diphenyl-1,3-butadien **1a** (1.35 mmol, 1.35 equiv) in 5 mL anhydrous DCM (prepared under inert gas atmosphere in a pear-shaped flask) *via* syringe pump in 20 min (15 mL/h) at 0 °C. After complete addition, 135.0 mg *p*-benzoquinone (1.25 mmol, 1.25 equiv) was added and the mixture was stirred for 15 min at 0 °C. The reaction was ended by the addition of 10 mL sat. $NaHCO_3$ and transferred to a separatory funnel with 5 mL DCM. The phases were separated and the aqueous phase was extracted with DCM (3 x 15 mL). The combined organic phases were dried over $MgSO_4$, filtered and the solvent was removed under reduced pressure. The residue was adsorbed on Celite® and purified by flash column chromatography (SiO_2, 30% cyclohexane in toluene) to yield the product as a yellow amorphous solid.

Yield: 385.3 mg (0.75 mmol, 63% based on (1*E*,3*E*)-1,4-diphenyl-1,3-butadien **1a** used).

Melting point: 184 – 186 °C.

***R*f** (30% cyclohexane in toluene): 0.21.

^{1}H NMR (400 MHz, CD_2Cl_2): δ 7.17 – 7.07 (m, 12H), 7.06 – 7.00 (m, 8H), 5.87 (d, *J* = 2.7 Hz, 4H), 4.76 (d, *J* = 2.7 Hz, 4H).

^{13}C NMR (101 MHz, CD_2Cl_2): δ 186.0, 142.3, 142.0, 128.9, 128.3,126.9, 126.9, 40.4.

IR (ATR, cm^{-1}): 1659 (m), 1489 (m), 1450 (m), 1272 (m), 853 (m), 758 (m), 729 (m), 693 (s), 655 (m), 512 (m).

HRMS (ESI): calc. for $C_{38}H_{28}NaO_2^+$ $[M+Na]^+$: 539.1982; found: 539.1982.

*7.7.2 1,4,5,8-Tetrahydro-1,4-di(4-methylphenyl)-5,8-diphenylanthracene-9,10-dione (**8b**)*

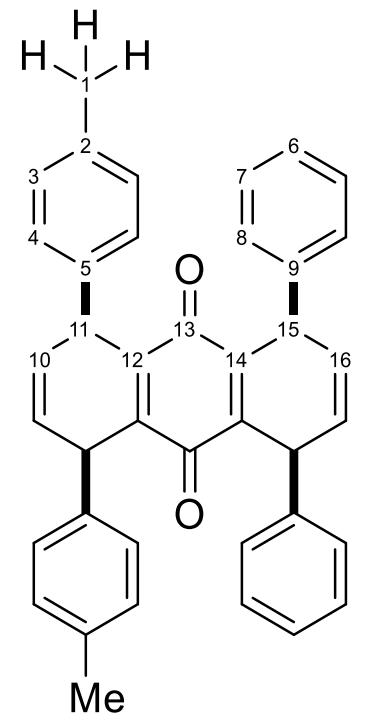

Under an inert gas atmosphere, 46.6 mg (1*E*,3*E*)-1,4-di(4-methylphenyl)-1,3-butadien **1z** (0.199 mmol, 1.00 equiv) and 5,8-dihydro-5,8-diphenylnaphthalene-1,4-dione **4a** (0.209 mmol 1.05 equiv) were dissolved in 2 mL anhydrous DCM. Then 2.58 µL MsOH (3.8 mg, 0.04 mmol, 0.2 equiv) was added and the reaction was stirred at r.t. for 50 min. Then 21.6 mg *p*-benzoquinone (0.200 mmol, 1.0 equiv) was added and the mixture was stirred at r.t. for another 60 min. The reaction was ended by the addition of 2 mL sat. $NaHCO_3$ and was diluted with 2 mL water and 2 mL DCM. The phases were separated and the aqueous phase was extracted with DCM (3 x 4 mL). The combined organic phases were dried over $MgSO_4$, filtered and the solvent was removed under reduced pressure. The residue was adsorbed on Celite® and purified by flash column chromatography (8 g SiO_2, 30% cyclohexane in toluene) to yield the product as a yellow amorphous solid.

Note: The relative stereochemistry was assigned by 2D-NMR experiments. The spectra for **8b** are provided in section 8.

Yield: 73.8 mg (0.136 mmol, 68%).

Melting point: 170 – 172 °C.

***R*f** (toluene): 0.18.

^{1}H NMR (700 MHz, CD_2Cl_2): δ 7.14 – 7.11 (m, H6 & H7, 6H), 7.05 – 7.00 (m, H8, 4H), 6.95 – 6.89 (m, H3 & H4, 8H), 5.86 (d, *J* = 2.4 Hz, H16, 2H), 5.83 (d, *J* = 2.4 Hz, H10, 2H), 4.74 (d, *J* = 2.3 Hz, H15, 2H), 4.69 (d, *J* = 2.3 Hz, H16, 2H), 2.26 (s, H1, 6H).

^{13}C NMR (APT) (176 MHz, CD_2Cl_2): δ 186.1 (C13), 142.2 (C12/C14), 142.2 (C12/C14), 142.1 (C9), 139.0 (C5), 136.5 (C2), 129.5 (C3), 128.9 (C6/C7), 128.3 (C8), 128.2 (C4), 126.9 (C16), 126.8 (C10), 126.8 (C6/C7), 40.4 (C15), 40.0 (C11), 21.1 (C1).

IR (ATR, cm^{-1}):1656 (s), 1509 (m), 1268 (m), 1180 (m), 856 (m), 807 (m), 796 (m), 752 (m), 695 (s), 657 (m), 519 (s).

HRMS (ESI): calc. for $C_{40}H_{32}KO_2^+$ $[M+K]^+$: 583.2034; found: 583.2033.

*7.7.3 1,4,5,8-Tetrahydro-1,4-di(4-bromophenyl)-5,8-diphenyl-9,10-anthracene-9,10-dione (**8c**)*

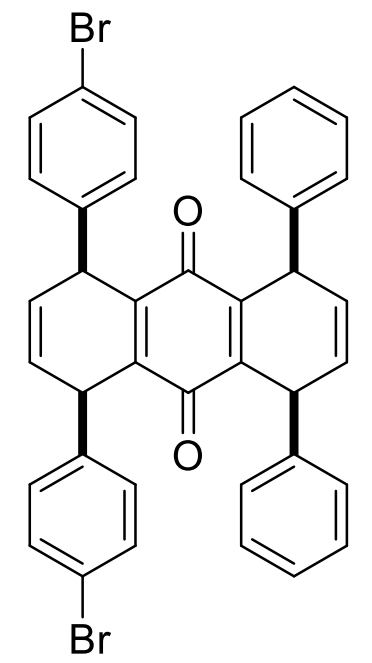

Under an inert gas atmosphere, 73.3 mg (1*E*,3*E*)-1,4-di(4-bromophenyl)-1,3-butadien **1aa** (0.201 mmol, 1.0 equiv) and 66.3 mg 5,8-dihydro-5,8-diphenylnaphthalene-1,4-dione **4a** (0.212 mmol 1.05 equiv) were dissolved in 2 mL anhydrous DCM and cooled to 0 °C. Then 0.882 µL TfOH (1.5 mg, 0.010 mmol, 5 mol%) was added and the reaction was stirred at 0°C for 15 min. Then 22.0 mg *p*-benzoquinone (0.204 mmol, 1.0 equiv) was added and the mixture was stirred at 0 °C for another 15 min. The reaction was ended by the addition of 2 mL sat. $NaHCO_3$ and was diluted with 2 mL water and 2 mL DCM. The phases were separated and the aqueous phase was extracted with DCM (3 x 4 mL). The combined organic phases were dried over $MgSO_4$, filtered and the solvent was removed under reduced pressure. The residue was adsorbed on Celite® and purified by flash column chromatography (8 g SiO_2, 30% cyclohexane in toluene) to yield the product as a yellow amorphous solid.

Yield: 119.4 mg (0.177 mmol, 88%).

Melting point: 202 – 204 °C.

***R*f** (toluene): 0.26.

^{1}H NMR (400 MHz, CD_2Cl_2): δ 7.25 – 7.11 (m, 10H), 7.03 – 6.97 (m, 4H), 6.90 – 6.84 (m, 4H), 5.88 – 5.83 (m, 4H), 4.74 – 4.66 (m, 4H).

^{13}C NMR (101 MHz, CD_2Cl_2): δ 186.0, 142.3, 141.9, 141.7, 140.7, 132.0, 130.2, 129.0, 128.4, 127.0, 126.8, 126.7, 120.9, 40.4, 39.7.

IR (ATR, cm^{-1}): 1656 (m), 1464 (m), 1274 (m), 1008 (m), 857 (m), 812 (m), 755 (m), 737 (m), 729 (m), 697 (s), 607 (m), 519 (s).

HRMS (ESI): calc. for $C_{38}H_{26}Br_2NaO_2^+$ $[M+Na]^+$: 697.0171; found: 697.0160.

*7.7.4 1,4,4a,5,8,9a-Hexahydro-1,4-ethano-5,8-diphenylanthracene-9,10-dione (**6e**)*

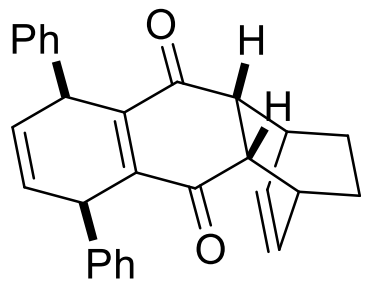

'bottom'-endo-**6e**

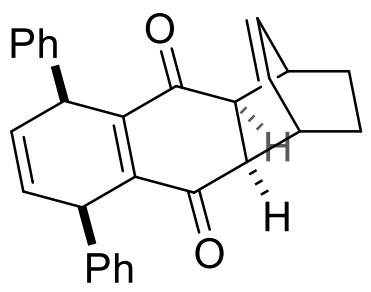

'top'-endo-**6e**

Under an inert gas atmosphere, 67.6 mg 5,8-dihydro-5,8-diphenylnaphthalene-1,4-dione **4a** (0.216 mmol, 1.0 equiv) was dissolved in 1.5 mL anhydrous DCM and cooled to 0 °C. Then 0.437 µL TfOH (13.3 mg, 0.09 mmol, 2.5 mol%) was added, followed by the dropwise addition of 22.9 mg cyclohexa-1,3-diene (0.285 mmol, 1.3 equiv) in 0.5 mL anhydrous DCM (prepared under inert gas atmosphere in a pear-shaped flask) *via* syringe pump in 15 min (2 mL/h) at 0 °C. After the addition was complete, the mixture was stirred for 10 min. The reaction was ended by the addition of 2 mL sat. $NaHCO_3$ and was diluted with 2 mL DCM. The phases were separated and the aqueous phase was extracted with DCM (3 x 4 mL). The combined organic phases were dried over $MgSO_4$, filtered and the solvent was removed under reduced pressure. The residue was adsorbed on Celite® and purified by flash column chromatography (9 g SiO_2, 30% Et_2O in *n*-pentane) to yield a more unpolar '*bottom*'-*endo*-product as a white amorphous solid and a more polar '*top*'-*endo*-product as a yellow amorphous solid.

Note: The relative stereochemistry was assigned by 2D-NMR experiments. The spectra for both products are provided in section 8.

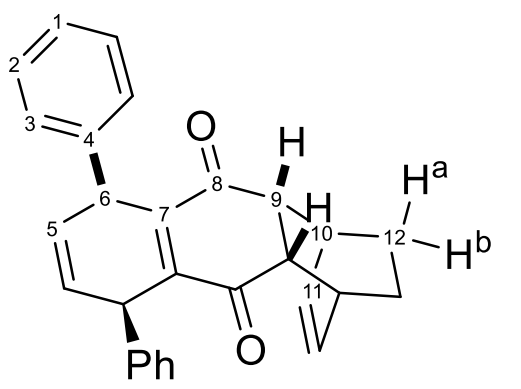

bottom-endo-**6e**

Yield ('*bottom*'-*endo*-**6e**): 19.3 mg (0.049 mmol, 23%).

Melting point:116 – 119 °C.

***R*f** (30% Et_2O in *n*-pentane): 0.50.

^{1}H NMR (600 MHz, CD_2Cl_2): δ 7.36 – 7.31 (m, H3, 4H), 7.31 – 7.27 (m, H2, 4H), 7.27 – 7.22 (m, H1, 2H), 6.19 (dd, *J* = 4.6, 3.0 Hz, H11, 2H), 5.81 (d, *J* = 2.1 Hz, H5, 2H), 4.76 (d, *J* = 2.0 Hz, H6, 2H), 3.34 – 3.28 (m, H10, 2H), 2.87 – 2.82 (m, H9, 2H), 1.54 – 1.49 (m, $H12^a$, 2H), 1.33 – 1.26 (m, $H12^b$, 2H).

^{13}C NMR (APT) (151 MHz, CD_2Cl_2): δ 196.9 (C8), 147.4 (C7), 142.1 (C4), 134.6 (C11), 129.1 (C2), 128.9 (C3), 127.3 (C1), 126.7 (C5), 50.6 (C9), 41.1 (C6), 31.7 (C10), 24.3 (C12).

IR (ATR, cm^{-1}): 1674 (s), 1490 (m), 1451 (m), 1269 (m), 1234 (m), 851 (m), 750 (m), 732 (m), 695 (s), 520 (m).

HRMS (ESI): calc. for $C_{28}H_{24}NaO_2^+$ $[M+Na]^+$: 415.1669; found: 415.1669.

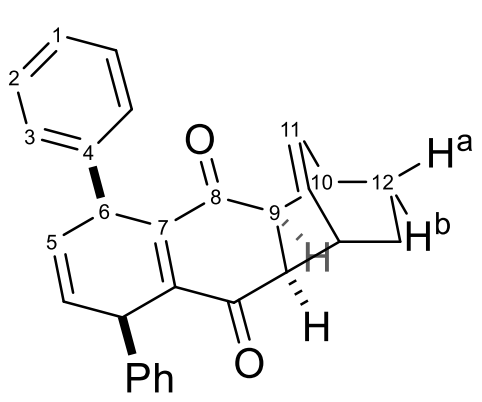

top-endo-**6e**

Yield ('*top*'-*endo*-**6e**): 60.1 mg (0.153 mmol, 71%).

Melting point: 175 – 177 °C.

***R*f** (30% Et_2O in *n*-pentane): 0.23.

^{1}H NMR (700 MHz, CD_2Cl_2) δ 7.36 – 7.32 (m, H2, 4H), 7.32 – 7.29 (m, H3, 4H), 7.27 – 7.23 (m, H1, 2H), 5.83 – 5.81 (m, H5, 2H), 5.65 (dd, *J* = 4.6, 3.1 Hz,

H11, 2H), 4.73 (d, J = 2.2 Hz, H6, 2H), 2.93 (t, J = 1.3 Hz, H11, 2H), 2.60 – 2.56 (m, H10, 2H), 1.62 – 1.58 (m, $H12^b$, 2H), 1.18 – 1.14 (m, $H12^a$, 2H).

^{13}C NMR (APT)(176 MHz, CD_2Cl_2): δ 198.3 (C8), 148.4 (C7), 141.9 (C4), 133.2 (C11), 129.1 (C3), 128.8 (C2), 127.1 (C1), 126.7 (C5), 50.2 (C9), 41.2 (C6), 36.7 (C10), 25.4 (C12).

IR (ATR, cm^{-1}):1660 (s), 1371 (m), 1253 (m), 1186 (m), 1171 (m), 850 (m), 743 (m), 721 (m), 696 (s), 685 (s), 501 (m).

HRMS (ESI): calc. for $C_{28}H_{24}NaO_2^+$ $[M+Na]^+$: 415.1669; found: 415.1669.

7.8 Synthesis and Analytical Data of [2+2]-Cycloaddition Products (7)

*7.8.1 Pentacyclo[6.2.2.0^{2,7}.0^{4,10}.0^{5,9}]dodecane-3,6-dione (7**a**)*

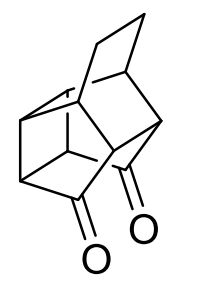

In a reaction vial 128.3 mg 1,4,4a,8a-tetrahydro-1,4-ethanonaphthalene-5,8-dione **6a** (0.68 mmol, 1.0 equiv) was dissolved in 10.0 mL anhydrous $CHCl_3$ and degassed with Argon for at least 15 min. The mixture was irradiated with 456 nm (Kessil PR160L, distance to light source 5 cm; cooled with a fan) for 39 h. The solvent was removed under reduced pressure. The residue was taken up in 0.8 mL toluene and carefully heated with a heating gun until dissolved. After cooling to r.t. the resulting solid was filtered to yield the product as a white amorphous solid.

Yield: 82.9 mg (0.44 mmol, 65%).

^{1}H NMR (400 MHz, $CDCl_3$): δ 3.14 – 3.06 (m, 2H), 3.01 – 2.94 (m, 2H), 2.52 (s, 2H), 2.14 – 2.05 (m, 2H), 1.94 – 1.85 (m, 2H), 1.77 – 1.68 (m, 2H).

^{13}C NMR (101 MHz, $CDCl_3$): δ 211.6, 48.2, 47.5, 35.6, 31.5, 16.7.

IR (ATR, cm^{-1}): 1748 (s), 1728 (s), 1710 (s), 1233 (m), 1051 (s), 926 (m), 906 (m), 868 (m), 502 (m), 436 (s).

HRMS (ESI): calc. for $C_{12}H_{12}NaO_2^+$ $[M+Na]^+$: 211.0730; found: 211.0730.

*7.8.2 2,5-Dimethylpentacyclo[6.2.2.0^{2,7}.0^{4,10}.0^{5,9}]dodecane-3,6-dione (7**b**)*

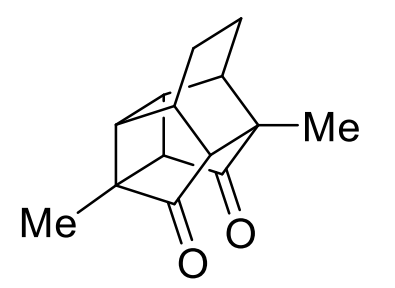

In a reaction vial 25.6 mg 1,4,4a,8a-tetrahydro-4a,7-dimethyl-1,4-ethanonaphthalene-5,8-dione **6c** (0.118mmol, 1.0 equiv) was dissolved in 2.0 mL anhydrous $CHCl_3$ and degassed with Argon for at least 15 min. The mixture was irradiated with 456 nm (Kessil PR160L, distance to light source 5 cm; cooled with a fan) for 24 h. The solvent was removed under reduced pressure and the residue was purified by flash column chromatography (5 g SiO_2, 20% EtOAc in *n*-hexane) to yield the product as a white amorphous solid.

Yield: 21.3 mg (0.098 mmol, 83%).

Melting point: 76 – 77 °C.

^{1}H NMR (400 MHz, $CDCl_3$): δ 3.10 – 3.01 (m, 1H), 2.73 – 2.63 (m, 1H), 2.58 (d, J = 6.5 Hz, 1H), 2.08 – 1.99 (m, 2H), 1.95 – 1.76 (m, 2H), 1.73 – 1.57 (m, 3H), 1.20 (s, 3H), 1.12 (s, 3H).

^{13}C NMR (101 MHz, $CDCl_3$): δ 213.8, 212.7, 55.9, 52.6, 52.2, 49.0, 40.6, 36.8, 32.2, 31.6, 16.9, 16.4, 15.8, 15.2.

IR (ATR, cm^{-1}): 2950 (m), 2923 (m), 2897 (m), 2863 (m), 1745 (s), 1727 (s), 1450 (m), 1135 (m), 1058 (m), 490 (m), 452 (m).

HRMS (ESI): calc. for $C_{14}H_{16}NaO_2^+$ $[M+Na]^+$: 239.1043; found: 239.1043.

*7.8.3 5,8-Diphenylhexacyclo[10.2.2.0^{2,11}.0^{4,9}.0^{4,14}.0^{9,13}]hexadec-6,7-ene-3,10-dione (**7c**)*

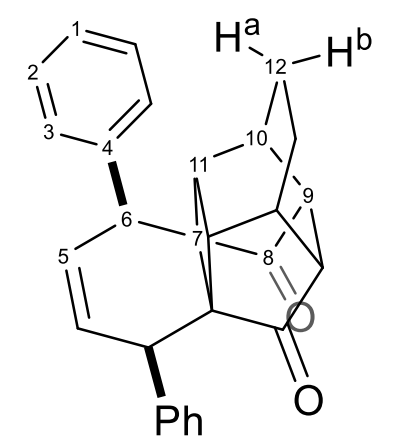

In a reaction vial 26.2 mg 1,4,4a,5,8,9a-hexahydro-1,4-ethano-5,8-diphenylanthracene-9,10-dione *endo*-**6e** (0.067mmol, 1.0 equiv) was dissolved in 2.0 mL anhydrous $CHCl_3$ and degassed with Argon for at least 15 min. The mixture was irradiated with 456 nm (Kessil PR160L, distance to light source 5 cm; cooled with a fan) for 64 h. The solvent was removed under reduced pressure and the residue was purified by flash column chromatography (4 g SiO_2, 30% Et_2O in *n*-pentane) to yield the product as a white amorphous solid.

Note: The relative stereochemistry was assigned by 2D-NMR experiments. The spectra for **7c** are provided in section 8.

Yield: 20.0 mg (0.051 mmol, 76%).

Melting point: 217 – 220 °C.

***R*f** (*n*-pentane/Et_2O): 0.35.

^{1}H NMR (700 MHz, CD_2Cl_2): δ 7.31 – 7.27 (m, H2, 4H), 7.27 – 7.25 (m, H3, 4H), 7.23 – 7.20 (m, H1 2H), 6.17 (d, *J* = 1.3 Hz, H5, 2H), 3.99 (d, *J* = 1.3 Hz, H6, 2H), 2.59 (dt, *J* = 3.0, 2.0 Hz, H11, 2H), 2.42 (q, *J* = 1.9 Hz, H9, 2H), 1.63 – 1.58 (m, H12^b, 2H), 1.48 – 1.42 (m, H12^a, 2H), 1.26 – 1.21 (m, H10, 2H).

^{13}C NMR (176 MHz, CD_2Cl_2): δ 212.8 (C8), 141.0 (C4), 130.9 (C5), 129.7 (C3), 128.5 (C2), 127.0 (C1), 60.5 (C7), 48.7 (C9), 37.4 (C6), 35.3 (C11), 30.3 (C10), 16.8 (C12).

IR (ATR, cm^{-1}): 1750 (s), 1730 (m), 1076 (m), 749 (m), 728 (m),702 (s), 659 (m), 562 (m), 497 (m), 473 (m).

HRMS (ESI): calc. for $C_{28}H_{24}NaO_2^+$ $[M+Na]^+$: 415.1669; found: 415.1669.

7.9 Synthesis and Analytical Data of Anthra-, Naphthacene- and Pentacenediones

7.9.1 Synthesis and Analytical Data of Reagents (16 and 18)

*7.9.1.1 3-Methyl-5-phenyl-2H-1,4-oxazin-2-one (**16**)*

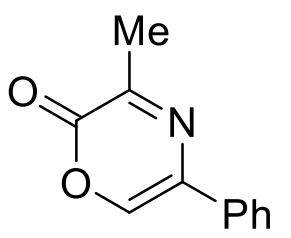

In an oven dried Schlenk flask under inert gas atmosphere 1.09 g phenyl glycinol (8.0 mmol, 1.0 equiv), 723 µL methyl pyruvate (8.0 mmol, 1.0 equiv) and 3.2 g 3Å molecular sieve (dried at 150 °C under high vacuum for 3 days) were suspended in 16 mL trifluoroethanol. The flask was sealed with a glass stopper, heated to 85 °C in an oil bath (after stirring at 85 °C for about one minute, the side arm valve was closed) and stirred for 23 h. After cooling to r.t. the mixture was filtered through Celite® with DCM as eluent. The solvent is removed under reduced pressure. The flask containing the obtained residue was evacuated and backfilled with inert gas three times, then dissolved in 40 mL anhydrous MeCN and cooled to 0 °C. 1.2 mL $BrCCl_3$ (12.2 mmol, 1.5 equiv) and 1.5 mL Et_3N (10.8 mmol, 1.3 equiv) were sequentially added and the resulting mixture was stirred for 17 h while warming to r.t.. 160 mL of a 1:1 mixture of *n*-hexane/Et_2O was added, the mixture was transferred into a separatory funnel and washed with 160 mL 1 M HCl. The phases were separated and the aqueous phase was extracted with *n*-hexane/Et_2O (1:1, 2 x 80 mL). The combined organic phases were washed with sat. $NaHCO_3$ and brine (80 mL each), dried over $MgSO_4$, filtered and the solvent removed under reduced pressure. The residue was purified by flash column chromatography (60 g SiO_2, 20% Et_2O in *n*-pentane) to yield the product as a white amorphous solid.

Yield: 523 mg (2.8 mmol, 35%).

^{1}H NMR (400 MHz, CD_2Cl_2): δ 7.75 – 7.66 (m, 3H), 7.48 – 7.35 (m, 3H), 2.52 (d, *J* = 1.0 Hz, 3H).

^{13}C NMR (101 MHz, CD_2Cl_2): δ 155.6, 154.0, 136.7, 133.8, 132.3, 129.2, 129.0, 125.5, 21.4.

IR (ATR, cm^{-1}): 1715 (s), 1619 (m), 1494 (m), 1451 (m), 1178 (m), 1075 (m), 1049 (m), 961 (m), 920 (m), 843 (m), 772 (s), 756 (m), 692 (m), 614 (m), 594 (m).

HRMS (ESI): calc. for $C_{11}H_9NNaO_2^+$ [M+Na]$^+$: 210.0526; found: 210.0525.

Spectral data are in accordance with those reported in the literature.[30]

*7.9.1.2 Naphthalene-2,3-dicarboxaldeyhde (**18**)*

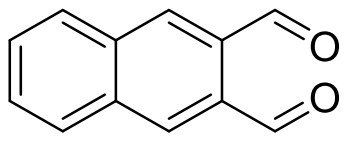

In an oven-dried Schlenk flask under an inert atmosphere, 79.0 mg lithium aluminium hydride (2.08 mmol, 2.1 equiv) was suspended in 2 mL anhydrous THF, then 198 mg 2,3-naphthalenedicarboxylic anhydride (0.99 mmol, 1.0 equiv) was added in portions and the resulting mixture was stirred for one hour. The mixture was cooled with an ice-water bath, then 2 mL 1 M HCl was added. The mixture was transferred to a separatory funnel, the phases were separated and the aqueous phase was extracted with DCM (3 x 5 mL). The combined organic phases were washed with sat. $NaHCO_3$ and brine, dried over $MgSO_4$, filtered and the solvent was removed under reduced pressure to yield 178.5 mg of a solid. The resulting solid was used without further purification.

In an oven-dried Schlenk flask under an inert gas atmosphere 0.375 mL DMSO (412.5 mg, 5.28 mmol, 5.6 equiv) in 1.2 mL DCM was stirred and cooled to – 78 °C. Then 0.240 mL $(COCl)_2$ (355.2 mg, 2.80 mmol, 3.0 equiv) in 4 mL DCM was added and the resulting mixture was stirred for 5 min. 178.5 mg 2,3-bis(hydroxymethyl)naphthalene (0.95 mmol, 1.0 equiv) in 1.4 mL THF/DMSO (9:1) was added dropwise and the resulting mixture was stirred at – 78 °C. After one hour 1.5 mL Et_3N (1095 mg, 10.5 mmol, 11.1 equiv) was added and the mixture allowed to warm to r.t. and stirred overnight. The reaction mixture was poured into 10 mL H_2O, the phases were separated and the aqueous phase was extracted with DCM (3 x 10 mL). The combined organic phases were washed twice with brine, dried over $MgSO_4$, filtered and the solvent was removed under reduced pressure. The residue was purified by flash column chromatography SiO_2, 20% Et_2O in *n*-pentane) to yield the product as a white amorphous solid.

Yield: 158 mg (0.86 mmol, 86%).

^{1}H NMR (400 MHz, $CDCl_3$): δ 10.65 (s, 2H), 8.47 (s, 2H), 8.07 (dd, J = 6.1, 3.1 Hz, 2H), 7.75 (dd, J = 6.2, 3.2 Hz, 2H).

^{13}C NMR (101 MHz, $CDCl_3$): δ 192.6, 134.6, 133.0, 130.2, 129.8.

IR (ATR, cm^{-1}): 1679 (s), 1620 (m), 1461 (m), 1445 (m), 1089 (m), 1015 (m), 847 (m), 797 (m), 782 (m), 752 (m), 736 (m), 479 (m).

HRMS (ESI): calc. for $C_{12}H_8NaO_2^+$ [M+Na]$^+$: 207.0417; found: 207.0416.

Spectral data are in accordance with those reported in the literature.[31]

7.9.2 Synthesis and Analytical Data of Anthracene-9,10-diones (10 to 12)

*7.9.2.1 Methyl 1,4-Diphenyl-9,10-dioxoanthracene-6-carboxylate (**10**)*

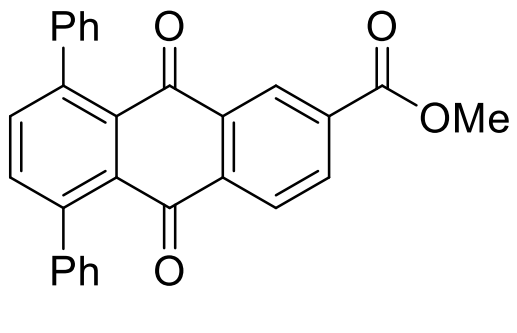

In a round-bottom flask with reflux condenser, 71.0 mg 5,8-diphenylnaphthalene-1,4-dione **5a** (0.229 mmol, 1.0 equiv) and 72.4 mg methyl 2-oxo-2H-pyran-5-carboxylate (0.470 mmol, 2.0 equiv) were suspended in 1.0 ml *o*-xylene, heated to 140 °C in an oil bath and stirred for 48 h. After cooling to r.t., Celite® was added to the mixture and the solvent was removed under reduced pressure. Purification by flash column chromatography (8 g SiO_2, 0.5% EtOAc in toluene) yielded the product as a yellow amorphous solid.

Yield: 63.2 mg (0.151 mmol, 66%).

Melting point: 188 – 190 °C

$\boldsymbol{R_f}$ (1% EtOAc in toluene): 0.16.

^{1}H NMR (400 MHz, CD_2Cl_2): δ 8.65 (d, J = 1.3 Hz, 1H), 8.32 (dd, J = 8.1, 1.8 Hz, 1H), 8.10 (d, J = 8.1 Hz, 1H), 7.60 (d, J = 1.0 Hz, 2H), 7.51 – 7.40 (m, 6H), 7.37 – 7.31 (m, 4H), 3.94 (s, 3H)

^{13}C NMR (101 MHz, CD_2Cl_2): δ 183.9, 183.7, 165.8, 144.4, 142.6, 142.6, 137.1, 137.0, 137.0, 135.3, 134.6, 134.4, 133.3, 128.5, 128.5, 128.3, 127.5, 127.3, 52.9.

IR (ATR, cm^{-1}): 1730 (s), 1666 (s), 1298 (m), 1241 (s), 1229 (s), 940 (m), 758 (m), 737 (m), 728 (s), 703 (s), 696 (s).

HRMS (ESI): calc. for $C_{28}H_{18}NaO_4^+$ $[M+Na]^+$: 441.1097; found: 441.1101.

*7.9.2.2 3-Methyl-1,6,9-triphenylbenz[g]isoquinoline-5,10-dione (**11**)*

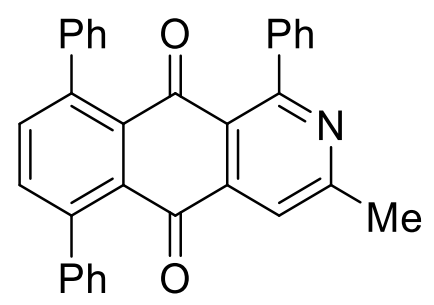

In a round-bottom flask with reflux condenser, 63.3 mg 5,8-diphenylnaphthalene-1,4-dione **5a** (0.204 mmol, 1.0 equiv) and 39.8 mg 3-methyl-5-phenyl-2H-1,4-oxin-2-one **16** (0.213 mmol, 1.0 equiv) were suspended in 1.0 ml toluene, heated to 120 °C in an oil bath and stirred for 16 h. After cooling to r.t., Celite® was added to the mixture and the solvent was removed under reduced pressure. Purification by flash column chromatography (10 g SiO_2, toluene) yielded the product as an orange amorphous solid.

Yield: 87.4 mg (0.194 mmol, 91%).

Melting point: 183 – 186 °C.

$\boldsymbol{R_f}$ (toluene): 0.17.

^{1}H NMR (400 MHz, CD_2Cl_2): δ 8.20 – 8.15 (m, 3H), 7.65 (d, J = 7.9 Hz, 1H), 7.56 (d, J = 7.8 Hz, 1H), 7.54 – 7.42 (m, 9H), 7.41 – 7.32 (m, 4H), 2.88 (s, 3H).

^{13}C NMR (101 MHz, CD_2Cl_2): δ 186.6, 184.6, 160.4, 160.3, 143.7, 143.5, 142.4, 142.2, 142.0, 138.2, 137.3, 136.2, 135.8, 132.1, 130.7, 129.3, 128.8, 128.7, 128.7, 128.5, 127.7, 127.7, 127.6, 125.3, 113.8, 25.4.

IR (ATR, cm^{-1}): 1668 (m), 1572 (m), 1540 (m), 1373 (m), 1330 (s), 1307 (m), 1230 (m), 1186 (m), 1121 (m), 902 (m), 745 (m), 691 (s).

HRMS (ESI): calc. for $C_{32}H_{21}NNaO_2^+$ $[M+Na]^+$: 474.1465; found: 474.1454.

*7.9.2.3 1,4-Diphenylanthracene-9,10-dione (**12**)*

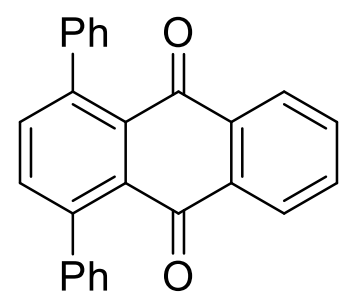

In a pressure tube, 31.1 mg 5,8-diphenylnaphthalene-1,4-dione **5a** (0.100 mmol, 1.0 equiv), 280 mg 3-sulfolene (2.37 mmol, 24 equiv) and 0.2 mg hydroquinone were suspended in 0.3 mL toluene, heated to 140 °C in an oil bath and stirred for 48 h. After cooling to r.t., the solvent was removed under reduced pressure and the residue taken up in 1 mL KOH solution (5 wt.% in water) and stirred under air atmosphere for 24 h. The suspension was neutralized with 1 M HCl and extracted with DCM (4 x 5 mL). The combined organic phases were dried over $MgSO_4$, filtered and the solvent removed under reduced pressure. The residue was purified by flash column chromatography (10 g SiO_2, 20% Et_2O in n-pentane) to yield the product as a yellow amorphous solid.

Yield: 25.7 mg (0.071 mmol, 71%).

Melting point: 204 – 207 °C.

$\boldsymbol{R_f}$ (20% Et_2O in n-pentane): 0.24; $\boldsymbol{R_f}$ (toluene): 0.18.

^{1}**H NMR** (400 MHz, CD_2Cl_2): δ 8.03 (dd, *J* = 5.8, 3.3 Hz, 2H), 7.73 (dd, *J* = 5.8, 3.3 Hz, 2H), 7.56 (s, 2H), 7.50 – 7.39 (m, 6H), 7.35 – 7.30 (m, 4H).

^{13}**C NMR** (101 MHz, CD_2Cl_2): δ 184.4, 144.2, 142.9, 136.7, 134.5, 134.2, 133.3, 128.5, 128.5, 127.4, 126.9.

IR (ATR, cm^{-1}): 2960 (m), 1661 (m), 1446 (m), 1411 (m), 1260 (m), 1244 (m), 1210 (s), 1168 (m), 1116 (m), 1002 (m), 796 (m), 723 (m), 695 (m), 655 (m), 510 (m).

HRMS (ESI): calc. for $C_{26}H_{16}NaO_2^+$ $[M+Na]^+$: 383.1043; found: 383.1046.

7.9.3 Synthesis and Analytical Data of Naphthacene- and Pentacenedione (13 and 14)

*7.9.3.1 1,4-Diphenylnaphthacene-5,12-dione (**13**)*

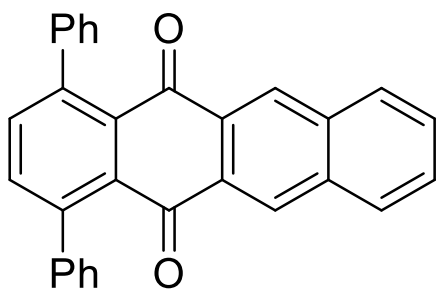

Under an inert gas atmosphere in a round-bottom flask, 32.8 mg 5,8-diphenylnaphthalene-1,4-dione **5a** (0.106 mmoL, 1.0 equiv) and 188 mg sodium dithionite (1.08 mmol, 10 equiv) were suspended in 0.75 mL degassed EtOAc and 0.75 mL degassed water and stirred for 2 h. After diluting the mixture with 2 mL EtOAc and 2 mL water, the phases were separated and the aqueous phase was extracted with DCM/EtOAc (1:1, 3 x 2 mL). The combined organic phases were washed with water and brine (each 2 mL), dried over MgSO4, filtered and the solvent removed under reduced pressure. The residue was taken up in 0.75 mL acetic acid, 23.0 mg phtalaldehyde (0.171 mmol, 1.6 equiv) and 2.0 mg *p*-TsOH (0.011 mmol, 0.1 equiv) were added. Then heated to 125 °C in an oil bath and stirred for 18 h. After cooling to r.t., the red-orange precipitate was filtered, washed twice with water, dissolved in DCM and adsorbed on Celite®. Purification with flash column chromatography (SiO_2, 50% DCM in *n*-hexane) yielded the product as an orange amorphous solid.

Yield: 38.6 mg (0.094 mmol, 89%).

Melting point: 309 – 310 °C.

$\boldsymbol{R_f}$ (toluene): 0.21.

^{1}**H NMR** (400 MHz, CD_2Cl_2): δ 8.58 (s, 2H), 8.05 (dd, *J* = 6.2, 3.2 Hz, 2H), 7.67 (dd, *J* = 6.3, 3.2 Hz, 2H), 7.59 (s, 2H), 7.51 – 7.41 (m, 6H), 7.39 – 7.33 (m, 4H).

^{13}**C NMR** (101 MHz, CD_2Cl_2): δ 184.4, 144.4, 143.1, 136.7, 135.5, 134.5, 131.1, 130.3, 129.6, 128.9, 128.6, 128.5, 127.4.

IR (ATR, cm^{-1}): 1671 (m), 1308 (m), 1264 (m), 1243 (m) 1195 (m), 974 (m), 772 (m), 755 (s), 698 (s), 522 (m), 475 (m).

HRMS (ESI): calc. for $C_{30}H_{18}NaO_2^+$ $[M+Na]^+$: 433.1199; found: 433.1198.

*7.9.3.2 1,4-Diphenylpentacene-5,14-dione (**14**)*

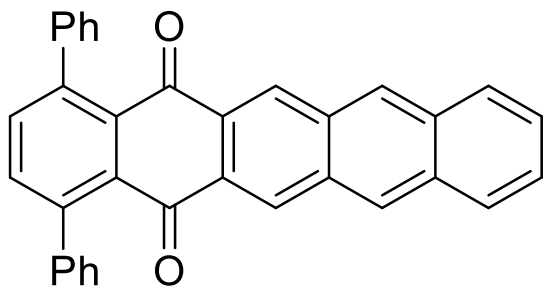

Under an inert gas atmosphere in a round-bottom flask, 62.8 mg 5,8-diphenylnaphthalene-1,4-dione **5a** (0.202 mmol, 1.0 equiv) and 300 mg sodium dithionite (1.72 mmol, 8.6 equiv) were suspended in 2.0 mL degassed EtOAc and 2.0 mL degassed water and stirred for 2 h. After diluting the mixture with 2 mL EtOAc and 2 mL water, the phases were separated and the aqueous phase was extracted with DCM/EtOAc (1:1, 3 x 5 mL). The combined organic phases were washed with water and brine (each 5 mL), dried over MgSO4, filtered and the solvent removed under reduced pressure. The residue was taken up in 1.5 mL acetic acid, 56.7 mg 2,3-naphthalenedicarboxaldehyde **18** (0.308 mmol, 1.5 equiv) and 3.3 mg *p*-TsOH (0.017 mmol, 6 mol%) were added. Then heated to 125 °C in an oil bath and stirred for 18 h. After cooling to r.t., the suspension

was neutralized with sat. $NaHCO_3$ and DCM was added until the red-orange precipitate was dissolved. The phases were separated and the aqueous phase was extracted with DCM. The combined organic phases were dried over $MgSO_4$, filtered and the solvent removed under reduced pressure. The residue was adsorbed on Celite® and purified with flash column chromatography (SiO_2, toluene) to yield the product as an orange amorphous solid.

Yield: 58.8 mg (0.128 mmol, 63%).

Melting point: 354 – 357 °C.

***R*f** (toluene): 0.23.

^{1}H NMR (400 MHz, CD_2Cl_2): δ 8.79 (s, 2H), 8.66 (s, 2H), 8.08 (dd, *J* = 6.5, 3.3 Hz, 2H), 7.65 – 7.56 (m, 4H), 7.53 – 7.34 (m, 10H).

^{13}C NMR (101 MHz, CD_2Cl_2): δ 184.2, 144.4, 143.2, 136.7, 134.9, 133.9, 132.2, 130.4, 130.2, 130.0, 128.9, 128.5, 128.4, 127.8, 127.3.

IR (ATR, cm^{-1}): 1671 (m), 1419 (m), 1315 (m), 1221 (s), 976 (m), 924 (m), 765 (m), 745 (s), 699 (s), 497 (m), 463 (m).

HRMS (ESI): calc. for $C_{34}H_{20}NaO_2^+$ $[M+Na]^+$: 483.1356; found: 483.1357.

7.10 Synthesis and Analytical Data of Functionalized 5,8-Dihydro-5,8-diphenylnaphthalene-1,4-dione and 5,8-Diphenylnaphthalene-1,4-dione

*7.10.1 1,4,5,8,9,10-Hexaphenylanthracene (**9**)*

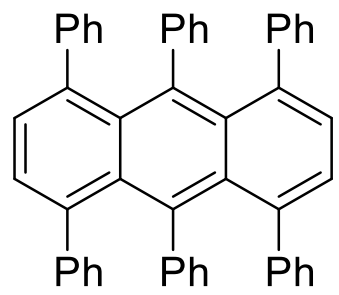

Under oxygen atmosphere 150.8 mg 1,4,5,8-tetrahydro-1,4,5,8-tetraphenylanthracene-9,10-dione (0.292 mmol, 1.0 equiv) was suspended in 20 mL 5 wt.% KOH in ethanol and heated to reflux in an oil bath. The reaction was run for 24 h (completion was monitored by TLC) and then cooled to r.t.. The resulting solid was filtered, then washed with water, methanol and Et_2O (5 mL each). The solid was transferred to a round-bottom flask and dried under vacuum and used without further purification (*Note*: 1,4,5,8-Tetraphenylanthracene-9,10-dione is barely soluble. Formation of 1,4,5,8-tetraphenylanthracene-9,10-dione was checked and confirmed by HRMS (HRMS (ESI): calc. for $C_{38}H_{24}NaO_2^+$ $[M+Na]^+$: 535.1669; found: 535.1663)).

The flask containing the solid was placed under argon atmosphere, suspended in 5 mL anhydrous THF and cooled to 0 °C. 2.2 mL PhLi (1.9 M in *n*-butylether; 4.18 mmol, 14.3 equiv) was added dropwise and the resulting mixture was stirred and warmed to r.t. overnight. The mixture was cooled to 0 °C and 5 mL 1 M HCl was added to end the reaction. The resulting mixture was transferred to a separatory funnel, the phases were separated and the aqueous phase was extracted with DCM (3 x 10 mL). The combined organic phases were washed with sat. $NaHCO_3$, water and brine (10 mL each), dried over $MgSO_4$, filtered and the solvent removed under reduced pressure. The resulting solid was taken up in approx. 1.5 mL Et_2O. The resulting white solid was filtered and washed with small portions Et_2O. The white solid was transferred to a round-bottom flask and dried under vacuum.

255 mg NaI (1.70 mmol, 5.8 equiv) and 180 mg NaH_2PO_2 (1.70 mmol, 5.8 equiv) were added to the solid. The flask containing a reflux condenser was evacuated and refilled with argon three times. 4.4 mL glacial acetic acid was added and the resulting suspension was heated to 140 °C in an oil bath. After 3 h, the mixture was cooled to r.t., and the yellow precipitate was filtered and washed with water and methanol. The crude solid was purified by flash column chromatography (SiO_2, 1% Et_2O and 2% toluene in *n*-pentane) to yield a yellow amorphous solid.

Yield: 58.9 mg (0.093 mmol, 32%).

^{1}**H NMR** (400 MHz, $CDCl_3$): δ 7.20 (s, 4H), 6.93 – 6.74 (m, 24H), 6.62 – 6.48 (m, 6H).

^{13}**C NMR** (101 MHz, $CDCl_3$): δ 144.0, 140.6, 140.3, 135.8, 135.6, 131.2, 129.6, 128.7, 127.1, 126.9, 126.4, 125.4.

IR (ATR, cm^{-1}): 1442 (m), 1025 (m), 835 (m), 821(m), 760 (m), 751 (m), 739 (m), 692 (s), 603 (m), 551 (m), 525 (m).

HRMS (APCI): calc. for $C_{50}H_{35}^+$ $[M+H]^+$: 635.2733; found: 635.2729.

Spectral data are in accordance with those reported in the literature.[32]

*7.10.2 2,3-Dihydro-2,3-epoxy-5,8-diphenylnaphthalene-1,4-dione (**19**)*

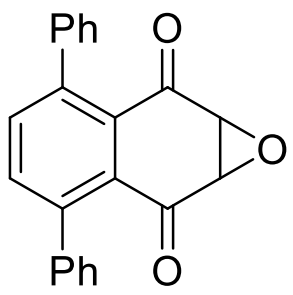

In a pear-shaped flask, 64.3 mg 5,8-diphenylnaphthalene-1,4-dione **5a** (0.207 mmol, 1.0 equiv) was suspended in 0.8 mL MeOH/H_2O (4:1) and cooled to 0 °C. Then 52 µL 2 M KOH solution (0.104 mmol, 0.5 equiv) and 40 µL H_2O_2 (33%, 0.392 mmol, 1.9 equiv) were added and the mixture was warmed to r.t. and stirred overnight. The mixture was diluted with 4 mL water and extracted with DCM (3 x 5 mL). The combined organic phases were dried over $MgSO_4$, filtered and the solvent removed under reduced pressure. The residue was purified by flash column chromatography (SiO_2, 50% DCM in *n*-hexane) to yield the product as a yellow amorphous solid.

Yield: 67.1 mg (0.206 mmol, 99%).

Melting point: 162 – 164 °C.

$\boldsymbol{R}_\mathbf{f}$ (toluene): 0.16.

^{1}**H NMR** (400 MHz, CD_2Cl_2): δ 7.63 (s, 2H), 7.48 – 7.38 (m, 8H), 7.35 – 7.29 (m, 2H), 4.04 (s, 2H).

^{13}**C NMR** (101 MHz, CD_2Cl_2): δ 193.6, 142.5, 140.1, 136.0, 132.0, 129.2, 128.7, 128.0, 56.0.

IR (ATR, cm^{-1}): 1699 (s), 1316 (m), 1235 (m), 874 (m), 833 (m), 761 (m), 744 (m), 698 (s), 569 (m), 505 (m).

HRMS (ESI): calc. for $C_{22}H_{14}NaO_3^+$ $[M+Na]^+$: 349.0835; found: 349.0837.

*7.10.3 2-(4-Methoxyphenyl)amino-5,8-diphenylnaphthalene-1,4-dione (**20**)*

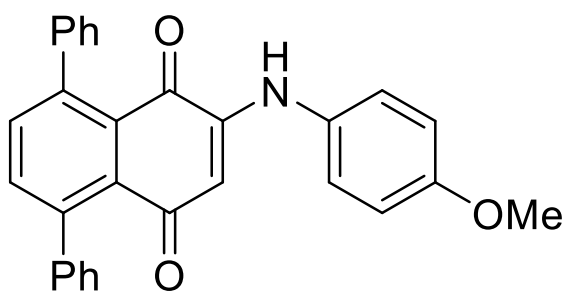

In a reaction vial 62.2 mg 5,8-diphenylnaphthalene-1,4-dione **5a** (0.20 mmol, 1.0 equiv), 25.0 mg *p*-anisidine (0.20, 1.0 equiv) and 4 mg $CeCl_3 \cdot 7\, H_2O$ (0.01 equiv, 5 mol%) were suspended in 1.6 mL EtOH and stirred for 48 h. The solvent was removed under reduced pressure and the residue purified by flash column chromatography (8 g SiO_2, DCM) to yield the product as a red solid.

Yield: 53.2 mg (0.12 mmol, 62%).

Melting point: 94 – 96 °C.

$\boldsymbol{R}_\mathbf{f}$ (DCM): 0.56.

^{1}**H NMR** (400 MHz, CD_2Cl_2): δ 7.51 (d, *J* = 7.8 Hz, 1H), 7.48 – 7.25 (m, 11H), 7.21 (s, 1H), 7.18 – 7.11 (m, 2H), 6.94 – 6.89 (m, 2H), 6.05 (s, 1H), 3.79 (s, 3H).

^{13}**C NMR** (101 MHz, CD_2Cl_2): δ 184.4, 182.7, 157.8, 145.5, 143.9, 143.2, 143.0, 142.7, 137.7, 135.0, 132.8, 130.9, 129.6, 128.5, 128.4, 128.4, 128.1, 127.4, 127.0, 124.6, 115.2, 103.5, 55.9.

IR (ATR, cm^{-1}): 1617 (s), 1579 (m), 1509 (s), 1337 (m), 1240 (s), 1151 (m), 1024 (m), 833 (m), 822 (m), 754 (s), 697 (s), 520 (m).

HRMS (ESI): calc. for $C_{29}H_{21}NNaO_3^+$ $[M+Na]^+$: 454.1414; found: 454.1413.

*7.10.4 1,4-Dimethoxy-5,8-diphenylnaphthalene (**21**)*

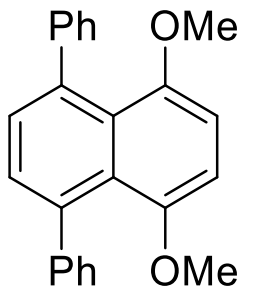

Under an inert gas atmosphere, 510 mg sodium dithionite (2.95 mmol, 6.2 equiv) was added to 147 mg 5,8-diphenylnaphthalene-1,4-dione **5a** (0.475 mmol, 1.0 equiv) and 5 mg tetrabutylammonium iodide (0.01 mmol, 2.9 mol%) in 1.7 mL degassed THF/H_2O (7:3). The mixture was stirred at r.t. for 30 min, then 888 mg potassium hydroxide (15.8 mmol, 33 equiv) was added, stirred for another 10 min, before 1.0 mL dimethyl sulfate (1330 mg, 10.5 mmol, 22 equiv) was added. After 17 h, the mixture was diluted with 20 mL water and 20 mL DCM, transferred to a separatory funnel and the phases were separated. The aqueous phase was extracted with DCM (3 x 20 mL), the combined organic phases were dried with $MgSO_4$, filtered and the solvent removed under reduced pressure. The residue was purified by flash column chromatography (20 g SiO_2, 50% cyclohexane in toluene) to yield the product as a white amorphous solid.

Yield: 116 mg (0.339 mmol, 71%).

Melting point: 130 – 132 °C.

***R*f** (30% cyclohexane in toluene): 0.37.

^{1}H NMR (400 MHz, CD_2Cl_2): δ 7.39 – 7.34 (m, 4H), 7.33 – 7.28 (m, 6H), 7.27 (s, 2H), 6.80 (s, 2H), 3.39 (s, 6H).

^{13}C NMR (101 MHz, CD_2Cl_2): δ 151.6, 146.2, 138.6, 129.2, 128.8, 127.1, 126.0, 125.8, 107.5, 56.2.

IR (ATR, cm^{-1}): 1454 (m), 1443 (m), 1384 (m), 1253 (s), 1083 (m), 1057 (m), 1005 (m), 968 (m),798 (s), 755 (s), 734 (m), 712 (m), 696 (s).

HRMS (ESI): calc. for $C_{24}H_{20}NaO_2^+$ $[M+Na]^+$: 363.1356; found: 363.1356.

*7.10.5 1,4-Dimethoxy-5,8-diphenyl-2-napthalenecarboxaldehyde (**22**)*

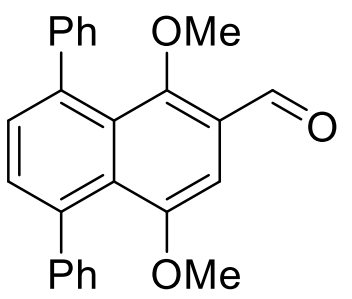

Under an inert gas atmosphere in an oven-dried Schlenk tube, 0.2 mL DMF (2.6 mmol, 8.9 equiv) was cooled to 0 °C. Then 54.0 µL $POCl_3$ (0.59 mmol, 2.0 equiv) was added and the resulting mixture stirred for 30 min. 99.1 mg 1,4-dimethoxy-5,8-diphenylnaphthalene **21** (0.29 mmol, 1.0 equiv) was added, followed by another 0.2 mL DMF. The suspension was heated to 90 °C in an oil bath and stirred for 24 h. After cooling the mixture to r.t., it was poured in 10 mL ice-cold water and transferred to a separatory funnel with EtOAc. After separation of the organic phase, the aqueous phase was extracted with EtOAc (3 x 10 mL). The combined organic phases were washed with sat. $NaHCO_3$, water and brine (each 10 mL), dried over $MgSO_4$, filtered and the solvent removed under reduced pressure. The residue was purified by flash column chromatography (20 g SiO_2, cyclohexane to toluene gradient: starting with two CVs cyclohexane, 10% steps, each step one CV) to yield the product as a white amorphous solid.

Yield: 46.2 mg (0.13 mmol, 43%).

^{1}H NMR (400 MHz, CD_2Cl_2): δ 10.43 (s, 1H), 7.49 – 7.25 (m, 12H), 7.13 (s, 1H), 3.49 (s, 3H), 3.20 (s, 3H).

^{13}C NMR (101 MHz, CD_2Cl_2): δ 190.1, 158.1, 154.2, 145.4, 143.9, 139.8, 139.6, 132.1, 131.0, 129.6, 129.4, 128.7, 128.1, 127.5, 127.3, 127.0, 126.4, 126.4, 101.2, 65.2, 55.7.

IR (ATR, cm^{-1}): 1671 (s), 1602 (m), 1366 (s), 1084 (m), 1071 (m), 1003 (m), 976 (m), 843 (m), 754 (s), 699 (s), 687 (m), 663 (m), 633 (m).

HRMS (ESI): calc. for $C_{25}H_{20}NaO_3^+$ $[M+Na]^+$: 391.1305; found: 391.1299.

*7.10.6 5,8-Dihydro-5,8-diphenyl-1,4-naphthalenediol (**23**)*

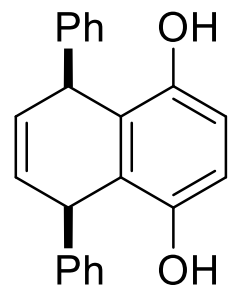

Under an inert gas atmosphere, 312 mg 5,8-dihydro-5,8-diphenylnaphthalene-1,4-dione **4a** (1.00 mmol, 1.0 equiv) and 1620 mg sodium dithionite (9.30 mmol, 9.3 equiv) were suspended in 20 mL degassed THF/H_2O (3:2) and stirred at r.t. for 25 min. The suspension was transferred to a separatory funnel with water and EtOAc, the phases were separated and the aqueous phase was extracted with EtOAc (3 x 20 mL). The combined organic phases were dried with $MgSO_4$, filtered and the solvent removed under reduced pressure. The residue was purified by flash column chromatography (30 g SiO_2, 50% Et_2O in *n*-pentane) to yield the product as a white amorphous solid.

Yield: 300 mg (0.95 mmol, 95%).

Melting point: 209 – 211 °C.

^{1}H NMR (400 MHz, $(CD_3)_2SO_2$): δ 8.55 (s, 2H), 7.20 – 7.13 (m, 8H), 7.12 – 7.05 (m, 2H), 6.54 (s, 2H), 5.96 (d, *J* = 3.3 Hz, 2H), 4.75 (d, *J* = 3.2 Hz, 2H).

^{13}C NMR (101 MHz, $(CD_3)_2SO_2$): δ 147.3, 144.7, 128.2, 127.9, 127.7, 125.4, 124.7, 113.1, 40.5.

IR (ATR, cm^{-1}): 3228 (br), 1492 (m), 1391 (m), 1373 (m), 1284 (m), 1230 (m), 848 (m), 793 (m), 753 (s), 728 (m), 704 (s), 689 (s), 533 (m), 499 (m).

HRMS (ESI): calc. for $C_{22}H_{18}NaO_2^+$ $[M+Na]^+$: 337.1199; found: 337.1201.

*7.10.7 5,8-Dihydro-1,4-dimethoxy-5,8-diphenylnaphthalene (**24**)*

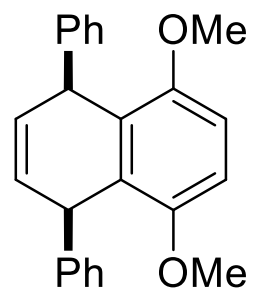

Under an inert gas atmosphere, 288 mg 5,8-dihydro-5,8-diphenyl-1,4-naphthalenediol **23** (0.917 mmol, 1.0 equiv) and 958 mg potassium carbonate (6.93 mmol, 7.5 equiv) were suspended in 15 mL AcMe and stirred. Carefully 0.25 mL dimethylsulfate (333 mg, 2.64 mmol, 2.9 equiv) was added, the suspension was heated to reflux in an oil bath and stirred for 19 h. After cooling to r.t., the solvent was removed under reduced pressure and the residue taken up in DCM and water (each approx. 20 mL). The phases were separated and the aqueous phase was extracted with DCM (3 x 20 mL). The combined organic phases were dried with $MgSO_4$, filtered and the solvent removed under reduced pressure. The residue was purified by flash column chromatography (30 g SiO_2, 5% Et_2O in *n*-pentane) to yield the product as a white amorphous solid.

Yield: 300 mg (0.877 mmol, 96%).

Melting point: 133 – 135 °C.

***R*$_f$** (20% Et_2O in *n*-pentane): 0.41; ***R*$_f$** (toluene): 0.55.

^{1}H NMR (400 MHz, CD_2Cl_2): δ 7.26 – 7.07 (m, 10H), 6.77 (s, 2H), 5.98 (d, *J* = 3.2 Hz, 2H), 4.87 (d, *J* = 3.2 Hz, 2H), 3.60 (s, 6H).

^{13}C NMR (101 MHz, CD_2Cl_2): δ 151.8, 145.3, 128.5, 128.4, 128.3, 127.9, 126.1, 109.6, 56.0, 41.5.

IR (ATR, cm^{-1}):1600 (w), 1745 (m), 1462 (m), 1433 (m), 1382 (m), 1253 (s), 1101 (m), 1090 (m), 1080 (m), 845 (m), 799 (m), 754 (m), 742 (m), 705 (s), 693 (s), 520 (m).

HRMS (ESI): calc. for $C_{24}H_{22}NaO_2^+$ $[M+Na]^+$: 365.1512; found: 365.1542.

*7.10.8 1,2,3,4-Tetrahydro-2,3-epoxy-5,8-dimethoxy-1,4-diphenylnaphthalene (**25**)*

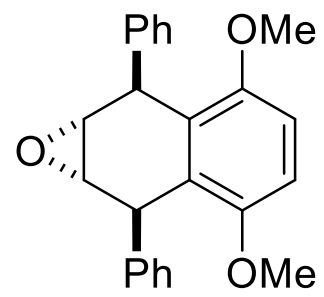

In a pear-shaped flask, 69.2 mg 5,8-dihydro-1,4-dimethoxy-5,8-diphenylnaphthalene **24** (0.202 mmol, 1.0 equiv) was dissolved in 1.0 mL DCM and cooled to 0 °C. Then 70.0 mg *m*-CPBA (70%, 0.284 mmol, 1.4 equiv) was added and after stirring for 30 min at 0 °C, the mixture was warmed to r.t. and stirred for 24 h. To the mixture 1 mL sat. $NaHCO_3$

was added, followed by 4 mL water and 4 mL DCM. The phases were separated and the aqueous phase was extracted with DCM (3 x 4 mL). The combined organic phases were washed with sat. $NaHCO_3$ (2 x 10 mL) and 10% $NaHSO_4$ (2 x 10 mL), dried over $MgSO_4$, filtered and the solvent was removed under reduced pressure. The residue was purified by flash column chromatography (8 g SiO_2, toluene) to yield the product as a white amorphous solid.

Yield: 43.0 mg (0.120 mmol, 59%).

Melting point: 148 – 150 °C.

R

R_f (toluene): 0.14.

^{1}H NMR (400 MHz, CD_2Cl_2): δ 7.23 – 7.12 (m, 10H), 6.80 (s, 2H), 4.96 (t, J = 1.6 Hz, 2H), 3.67 (s, 6H), 3.57 (t, J = 1.5 Hz, 2H)

^{13}C NMR (101 MHz, CD_2Cl_2): δ 152.3, 141.1, 129.3, 128.4, 126.9, 124.8, 109.8, 56.5, 56.1, 40.4.

IR (ATR, cm^{-1}): 1601(w), 1492 (m), 1474 (m), 1465 (m), 1451 (m), 1434 (m), 1255 (s), 1092 (s), 1021 (m), 823 (m), 792 (m), 748 (m), 719 (m), 695 (s), 518 (m).

HRMS (ESI): calc. for $C_{24}H_{22}NaO_3^+$ $[M+Na]^+$: 381.1461; found: 381.1464.

8. NMR Spectra

(1*E*,3*E*)-1,4-Diphenyl-1,3-butadiene (**1a**)

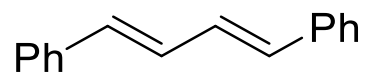

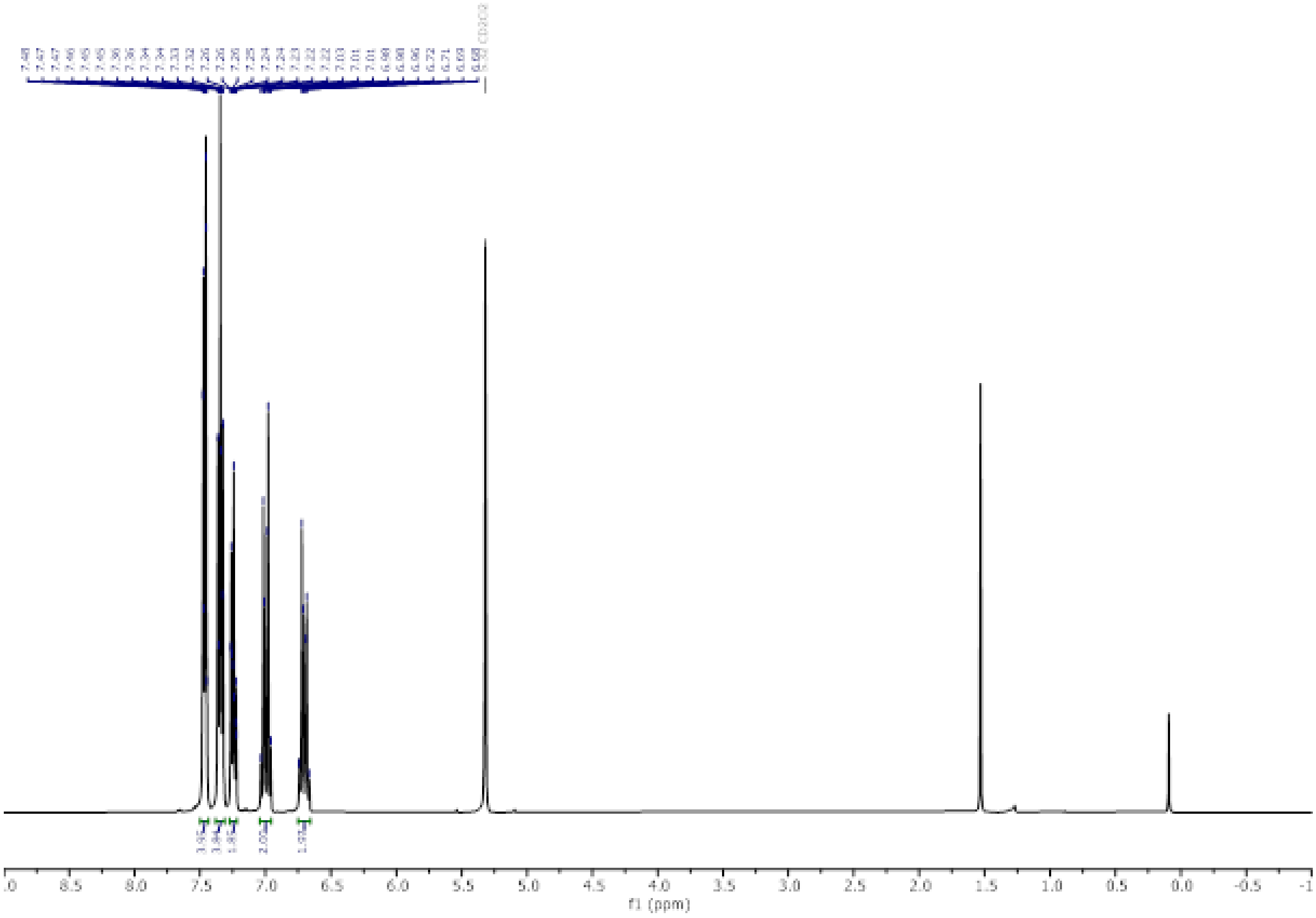

^{1}H NMR spectrum (400 MHz, CD_2Cl_2) of **1a**.

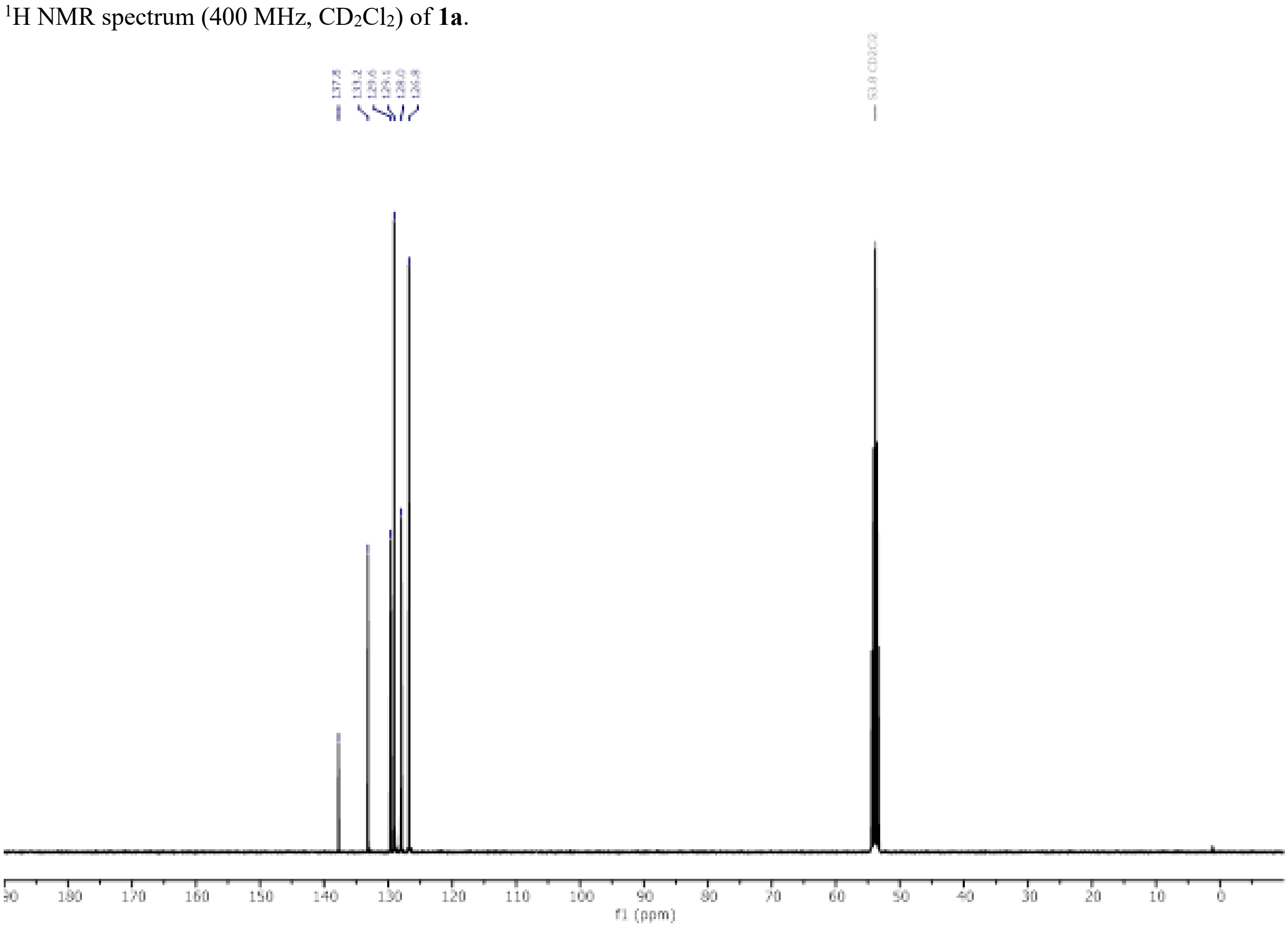

^{13}C NMR spectrum (101 MHz, CD_2Cl_2) of **1a**.

4-[(1*E*,3*E*)-4-phenyl-1,3-butadien-1-yl]-1,1'-biphenyl (**1b**)

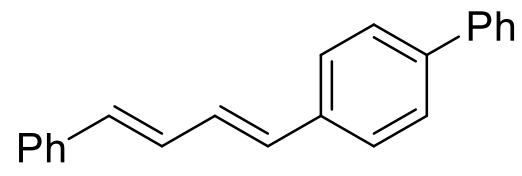

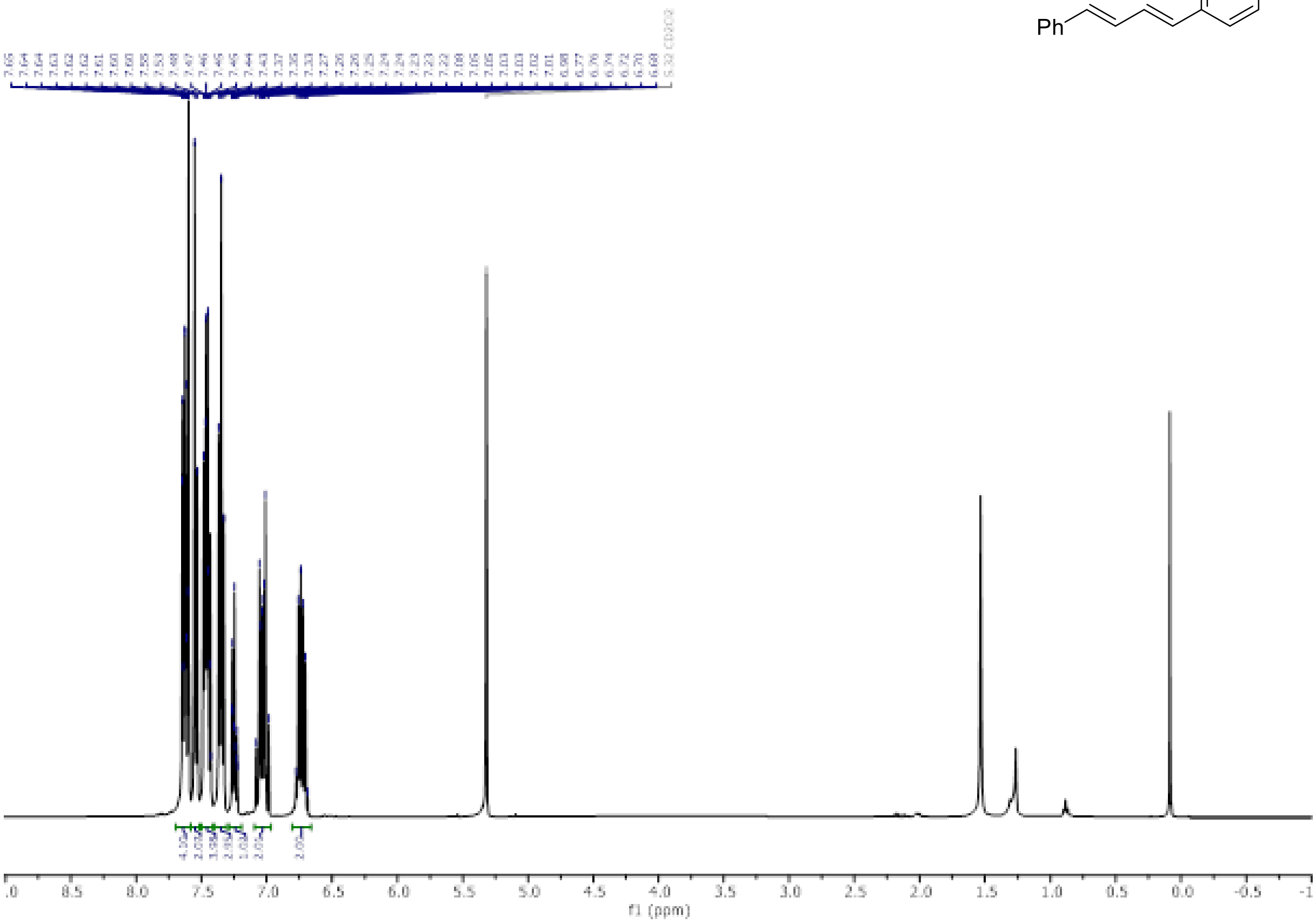

^{1}H NMR spectrum (400 MHz, CD_2Cl_2) of **1b**.

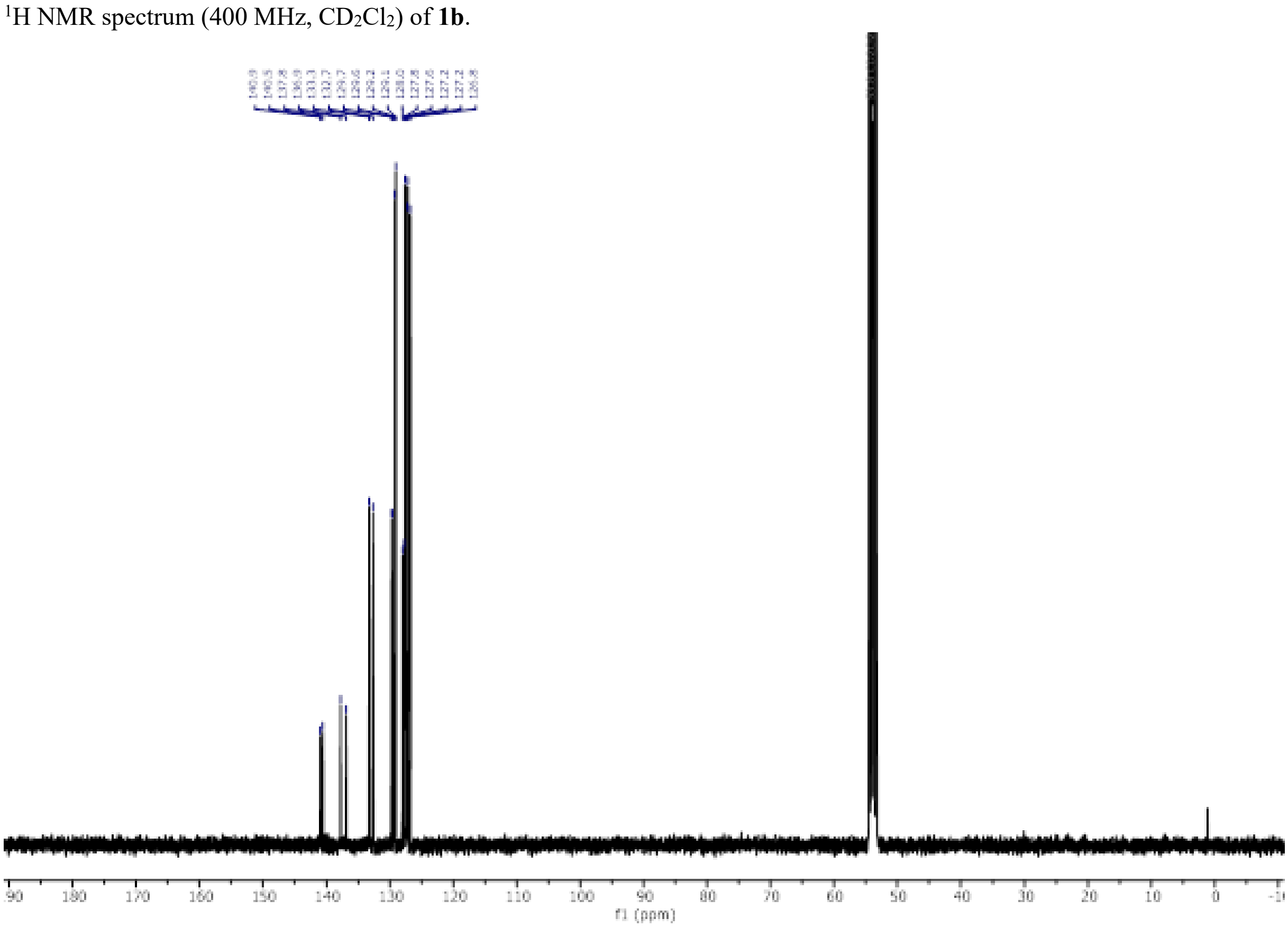

^{13}C NMR spectrum (101 MHz, CD_2Cl_2) of **1b**.

1-Methyl-2-[(1*E*,3*E*)-4-phenyl-1,3-butadien-1-yl]benzene (**1c**)

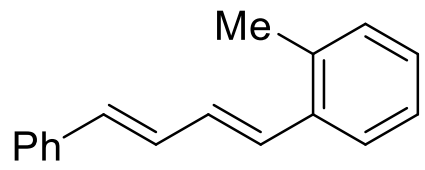

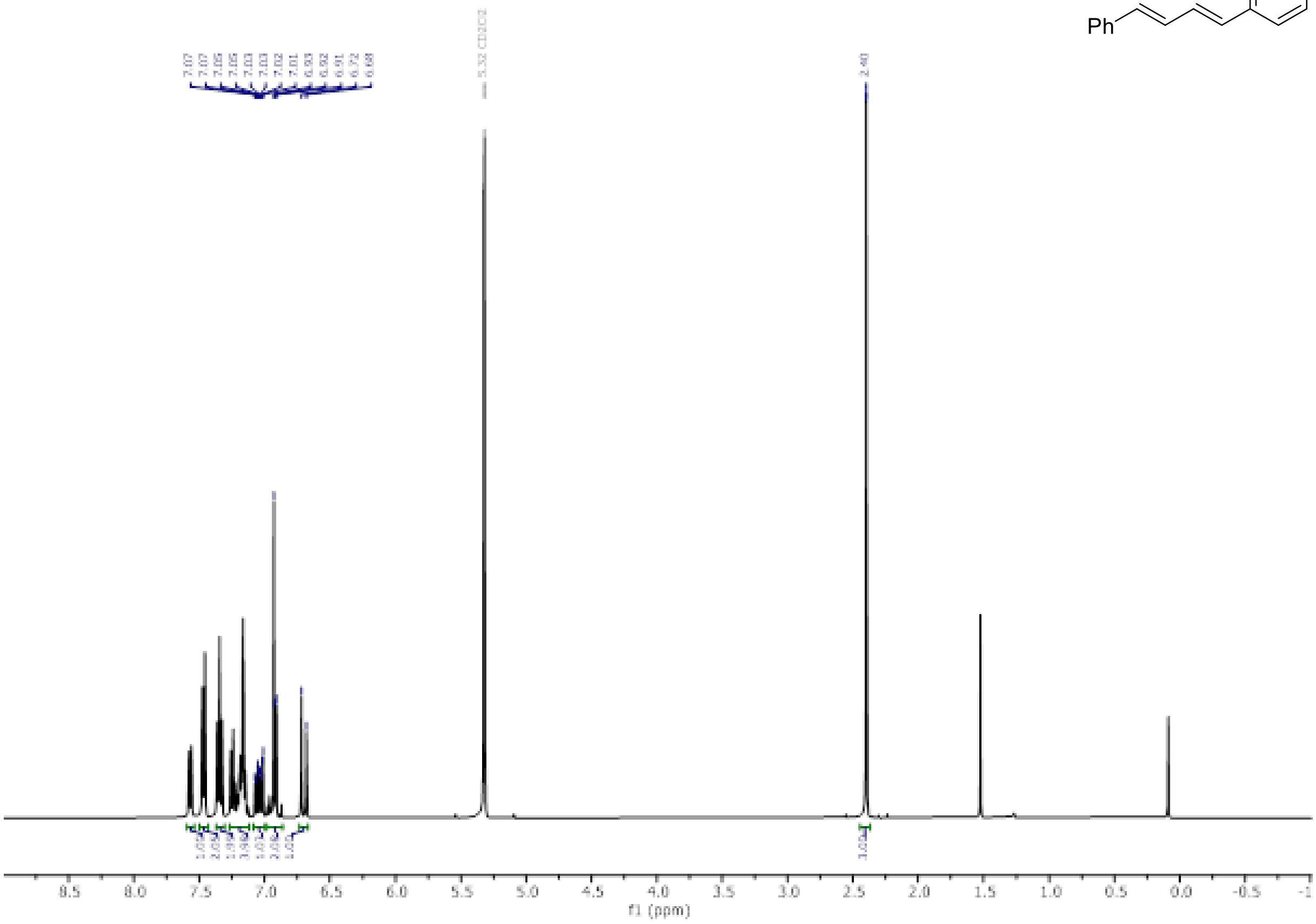

^{1}H NMR spectrum (400 MHz, CD_2Cl_2) of **1c**.

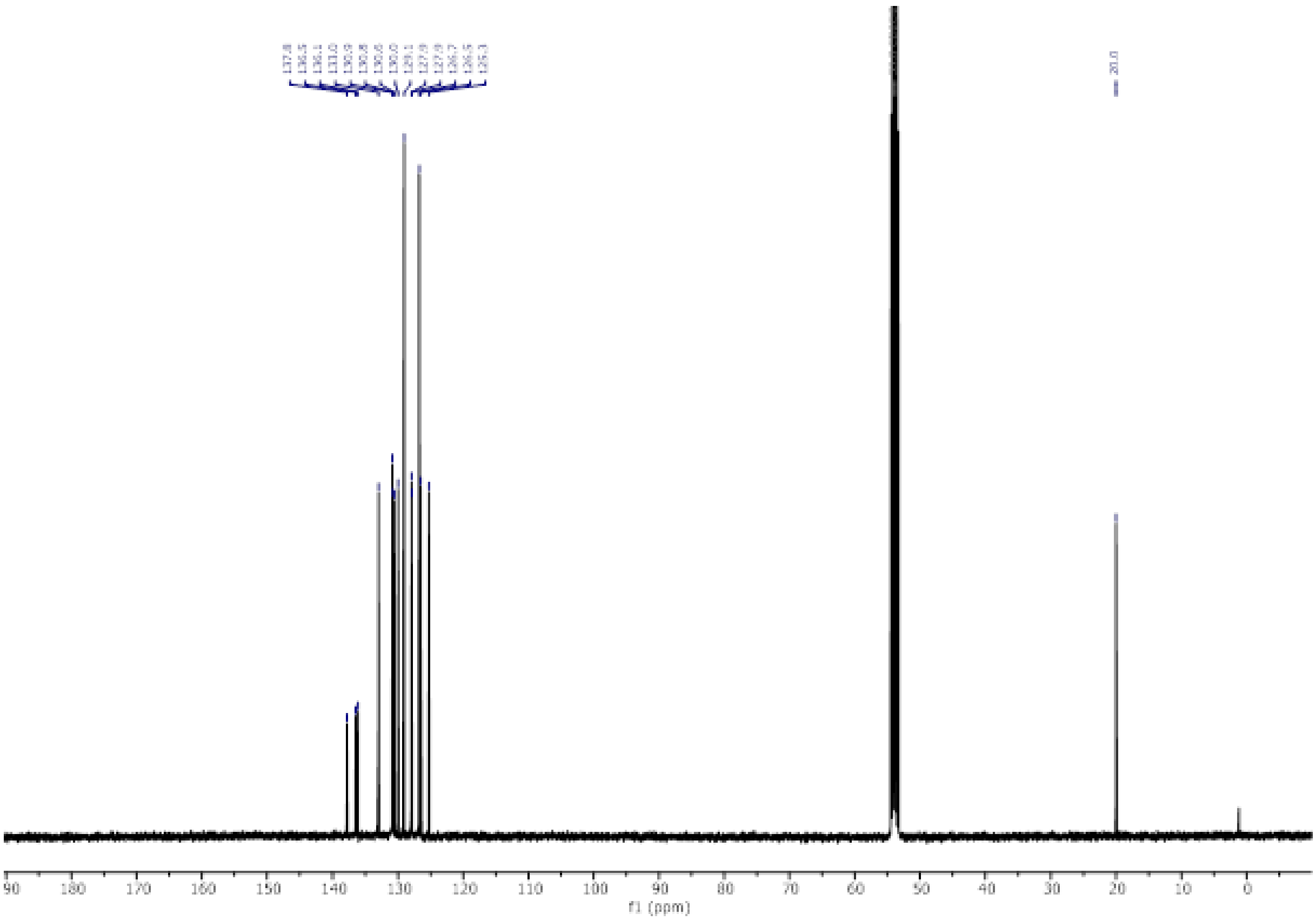

^{13}C NMR spectrum (101 MHz, CD_2Cl_2) of **1c**.

1-Methyl-3-[(1*E*,3*E*)-4-phenyl-1,3-butadien-1-yl]benzene (**1d**)

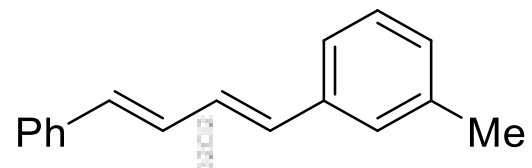

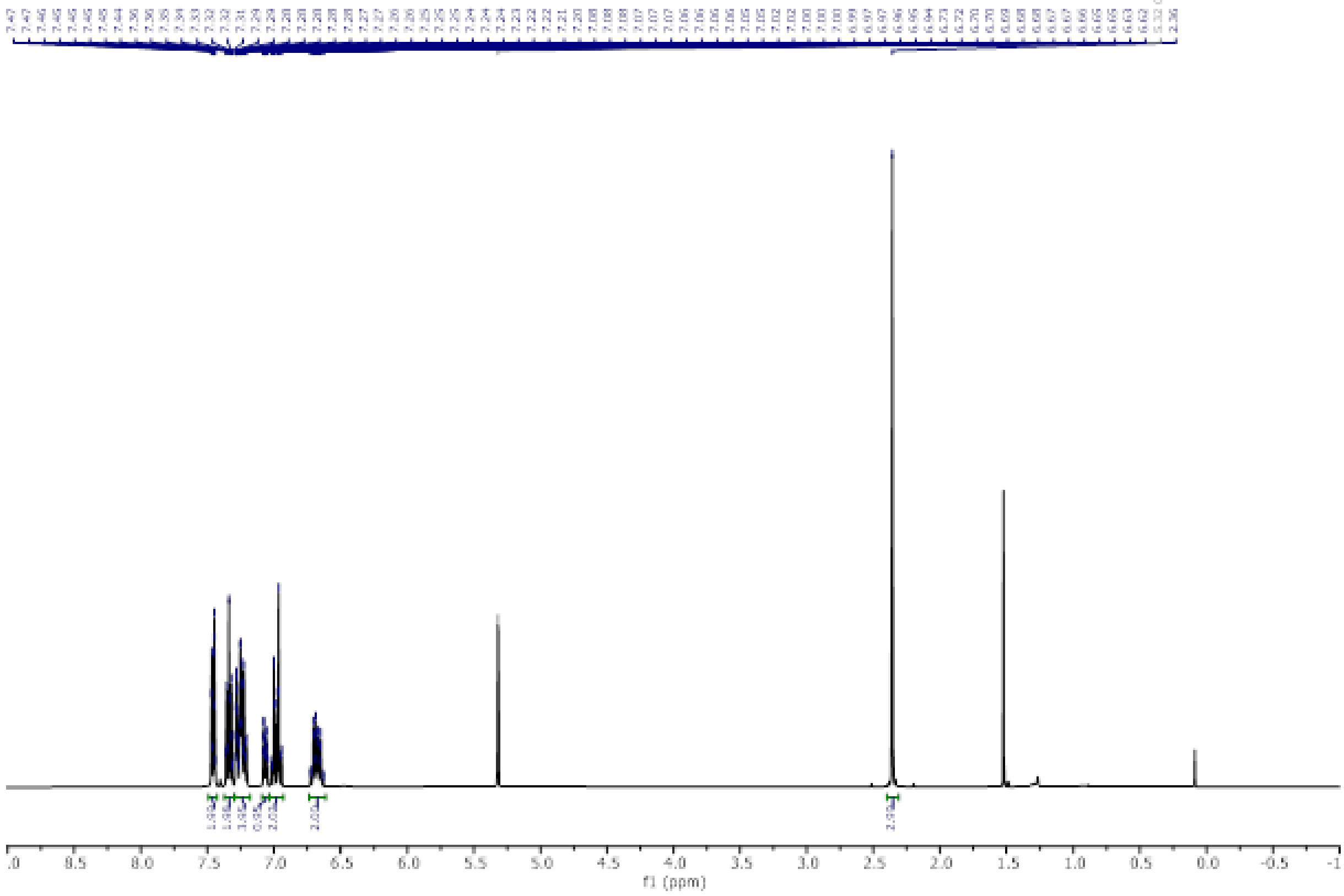

^{1}H NMR spectrum (400 MHz, CD_2Cl_2) of **1d**.

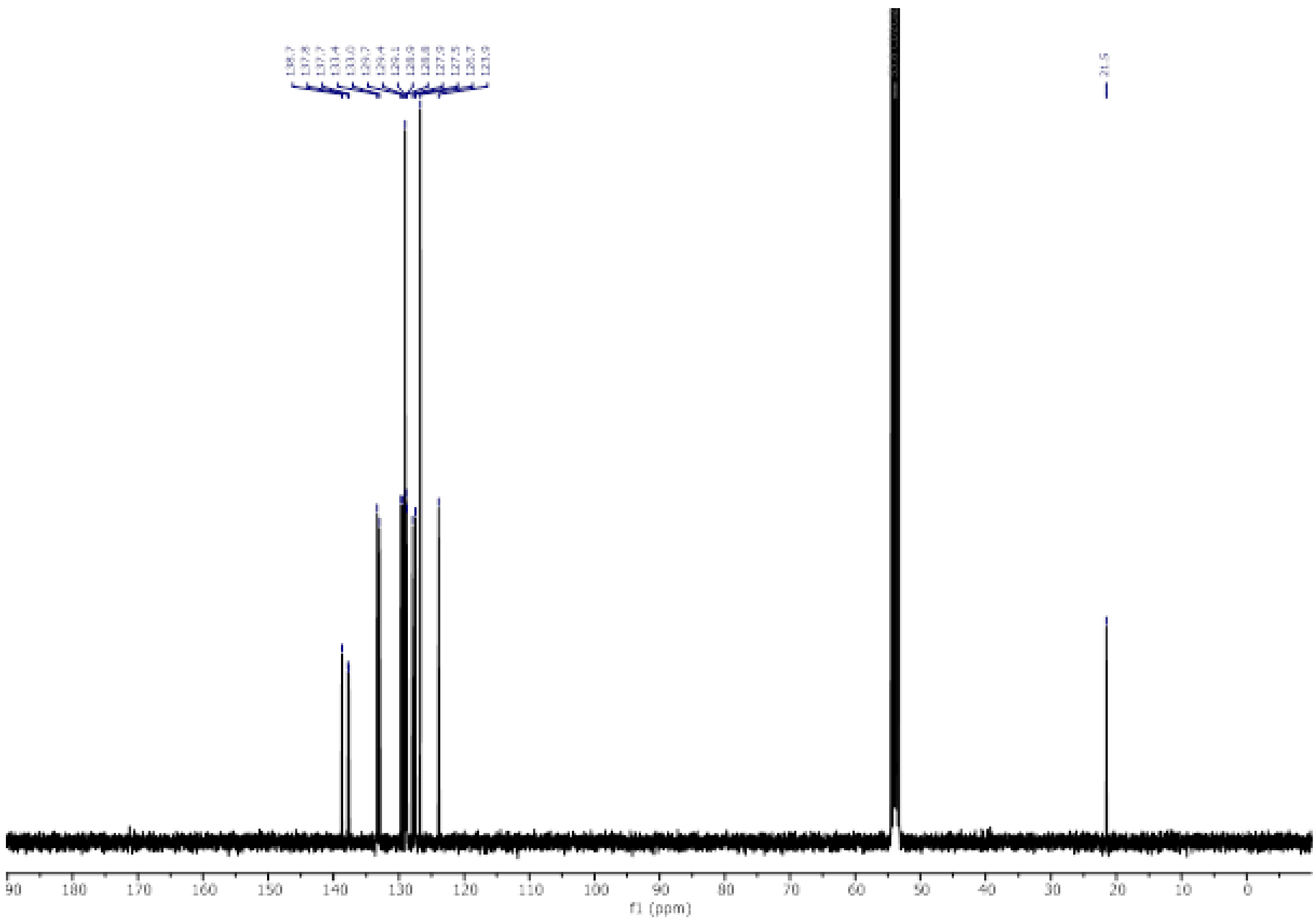

^{13}C NMR spectrum (101 MHz, CD_2Cl_2) of **1d**.

1-Methyl-4-[(1*E*,3*E*)-4-phenyl-1,3-butadien-1-yl]benzene (**1e**)

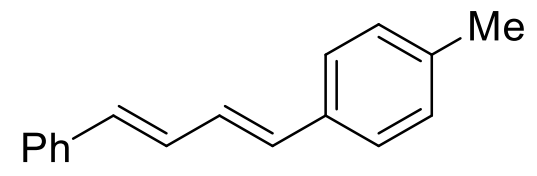

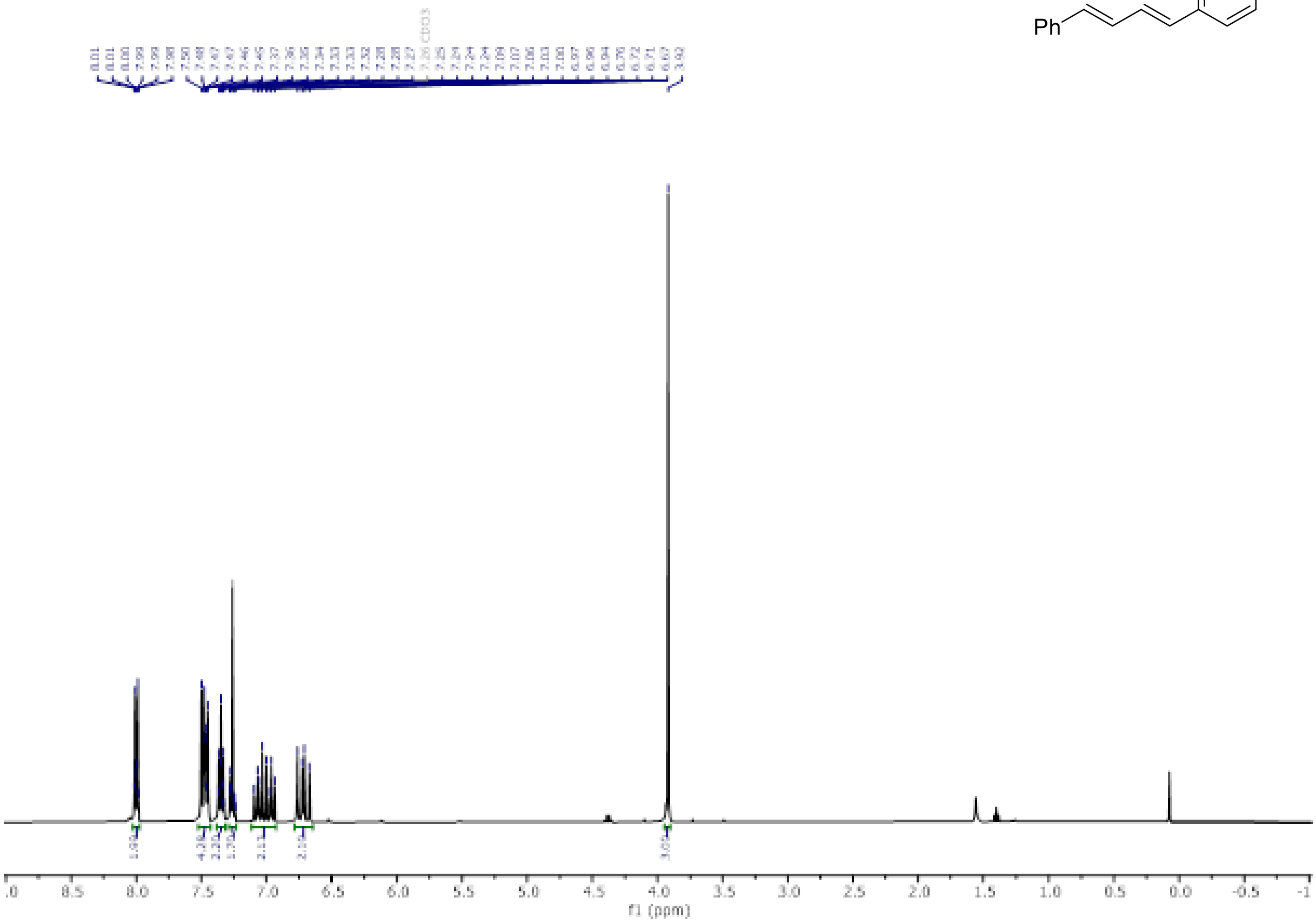

^{1}H NMR spectrum (400 MHz, CD_2Cl_2) of **1e**.

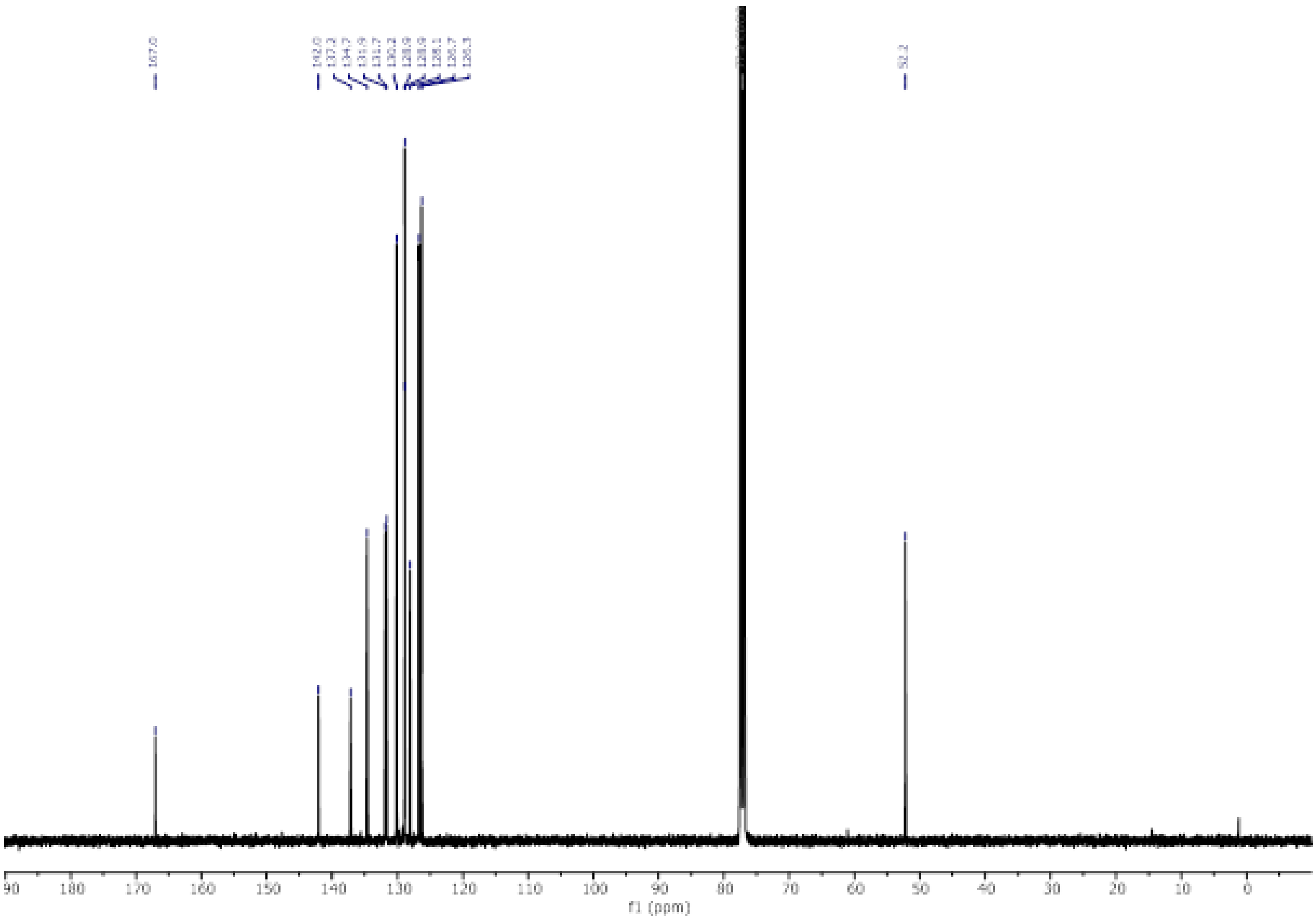

^{13}C NMR spectrum (101 MHz, $CDCl_3$) of **1e**.

1-Butyl-4-[(1*E*,3*E*)-4-phenyl-1,3-butadien-1-yl]benzene (**1f**)

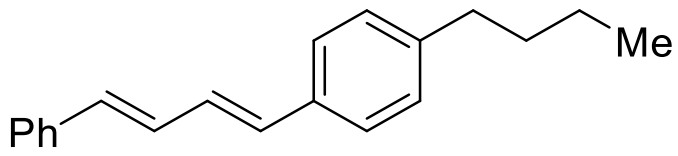

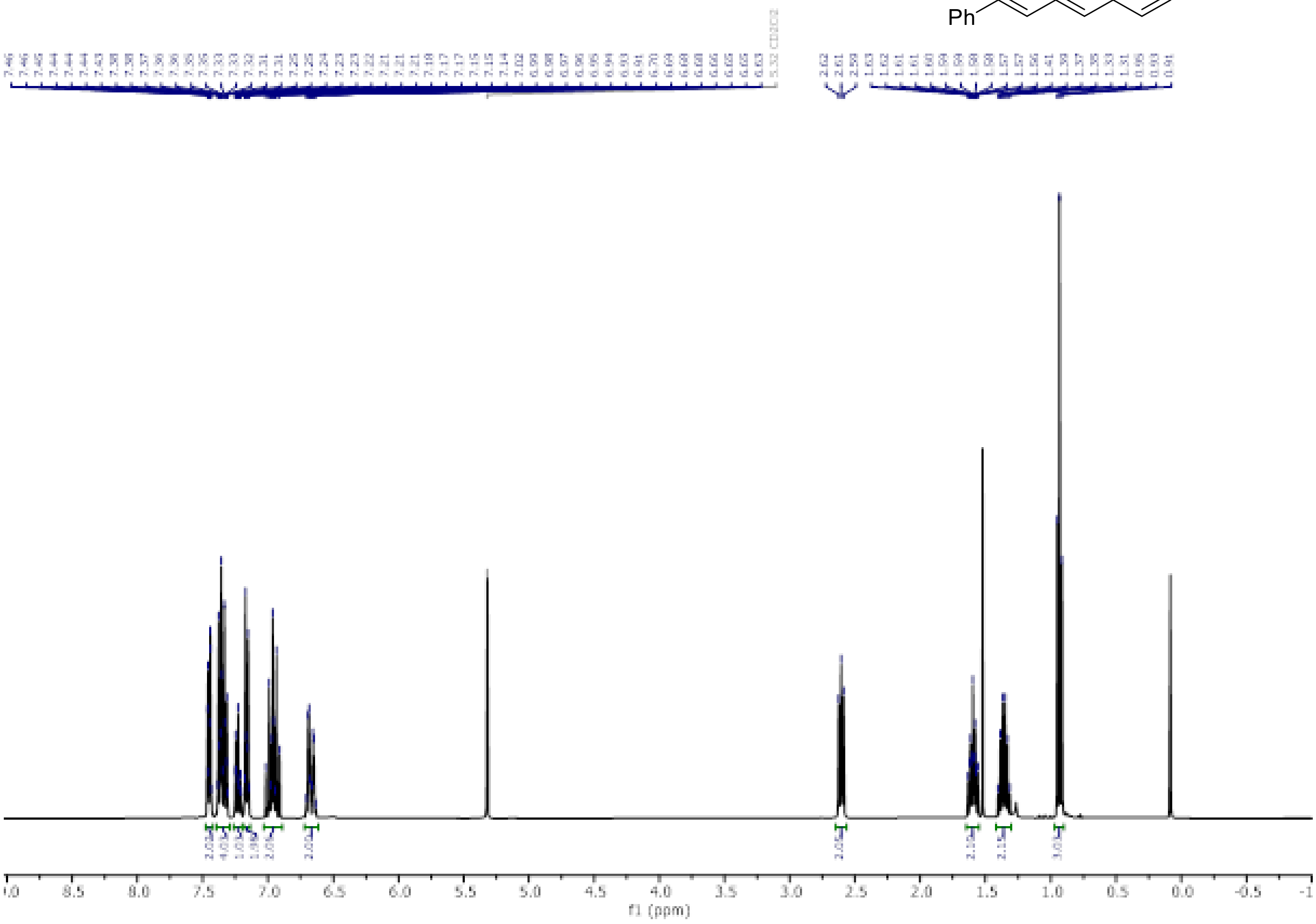

^{1}H NMR spectrum (400 MHz, CD_2Cl_2) of **1f**.

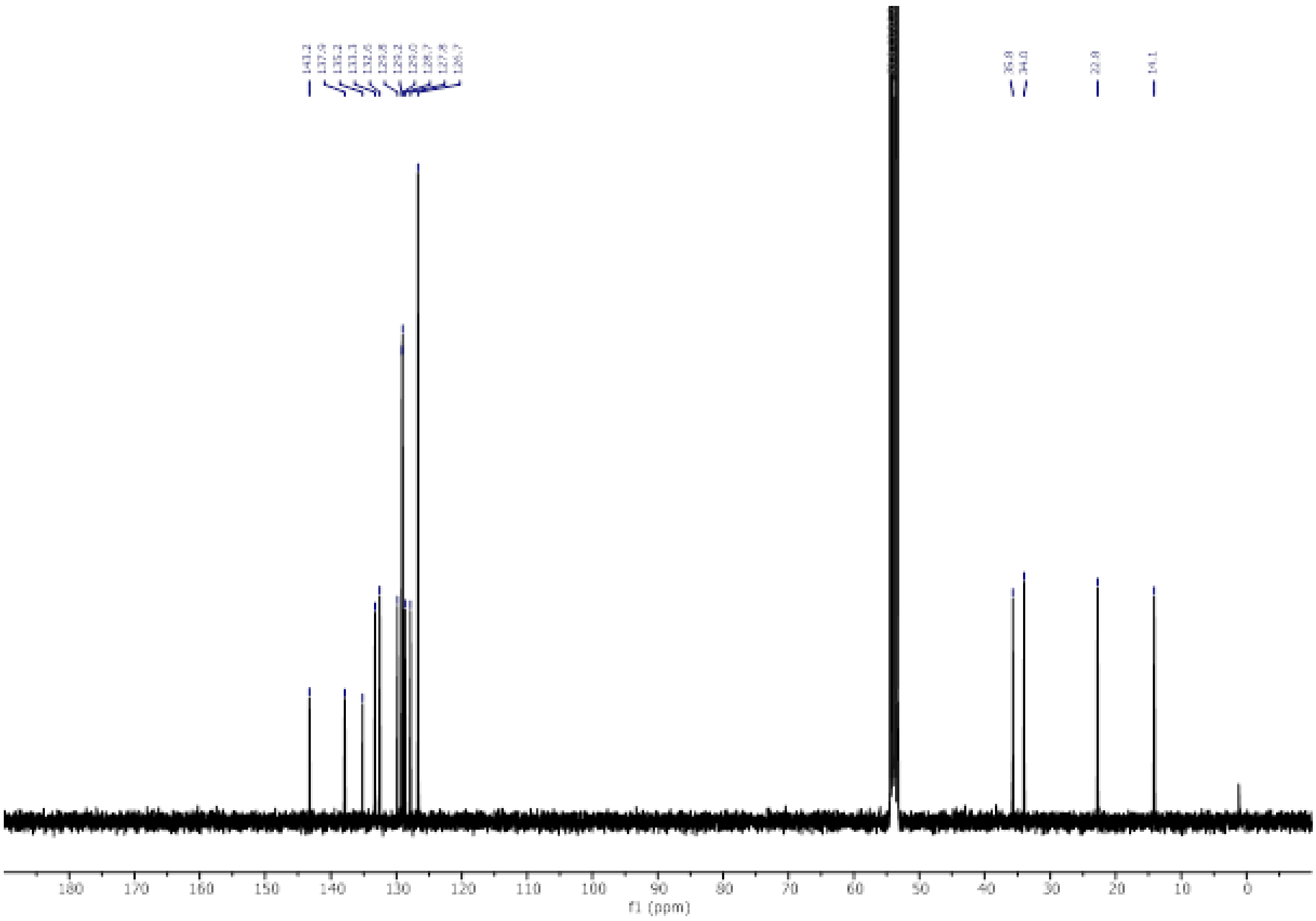

^{13}C NMR spectrum (101 MHz, CD_2Cl_2) of **1f**.

1-*tert*-Butyl-4-[(1*E*,3*E*)-4-phenyl-1,3-butadien-1-yl]benzene (**1g**)

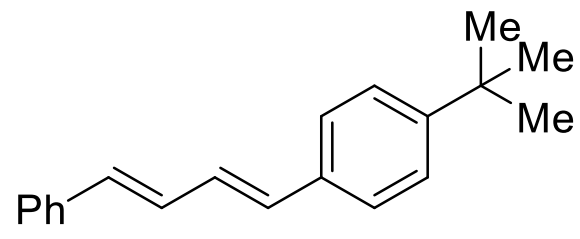

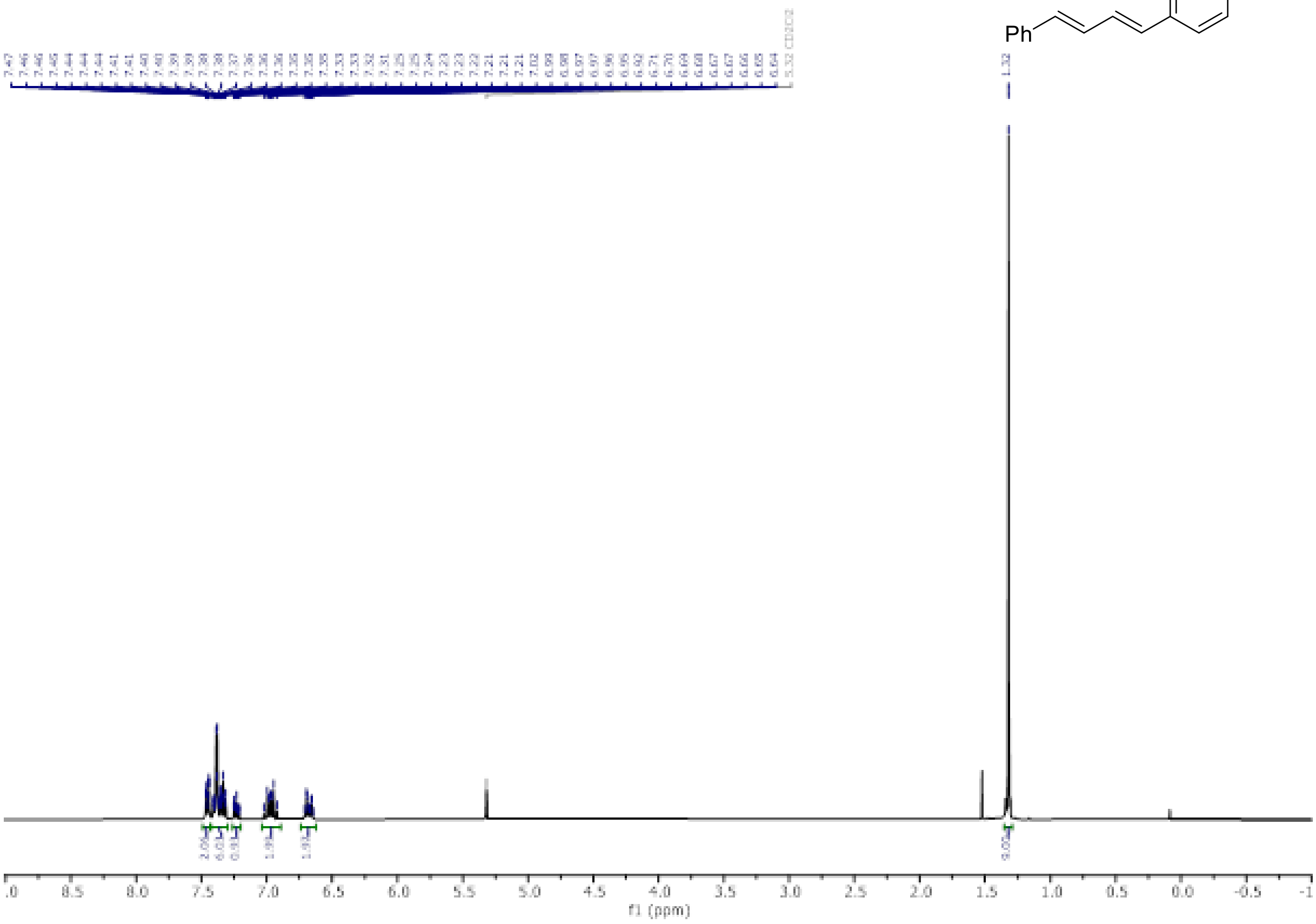

^{1}H NMR spectrum (400 MHz, CD_2Cl_2) of **1g**.

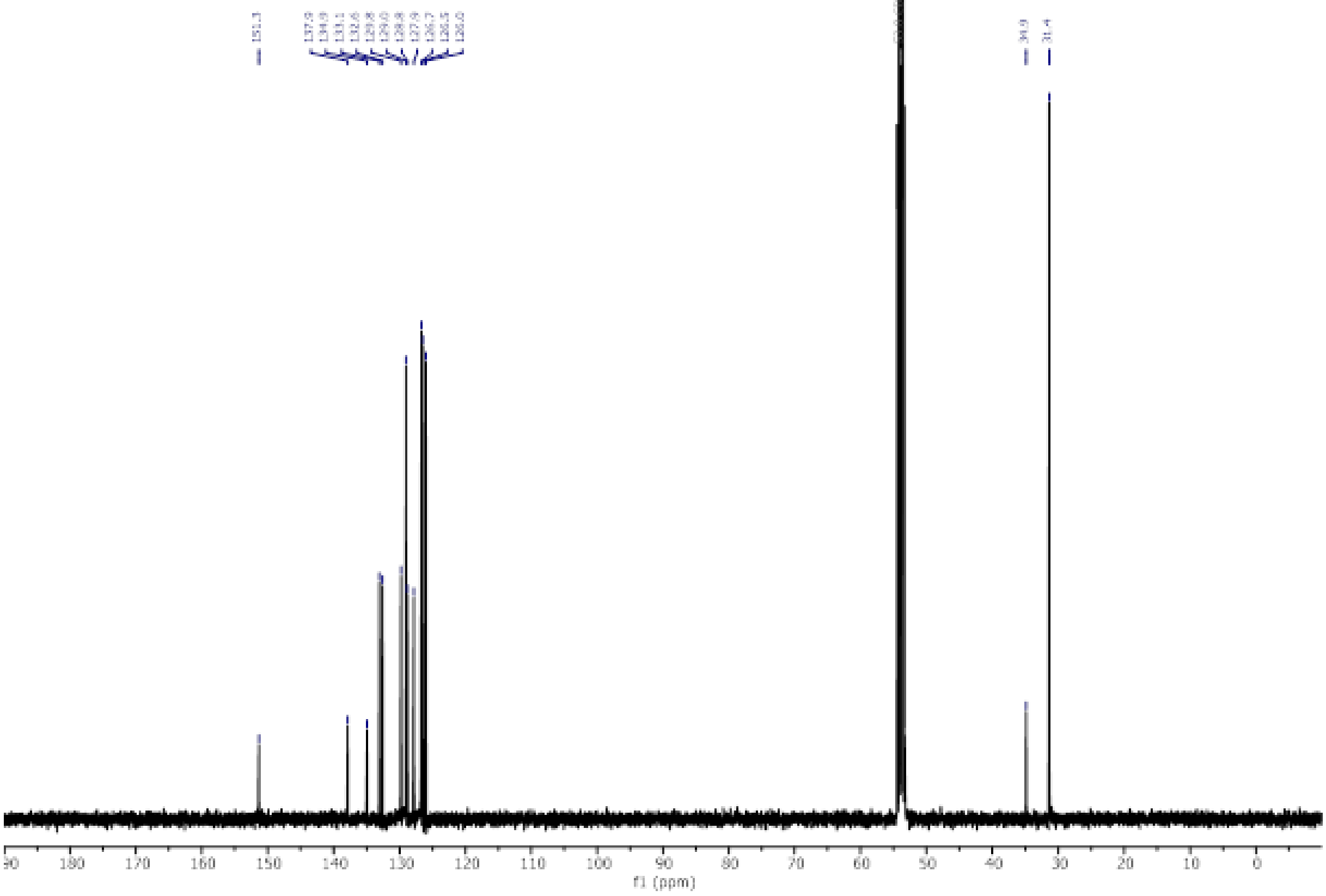

^{13}C NMR spectrum (101 MHz, CD_2Cl_2) of **1g**.

1-Methoxy-2-[4-phenyl-1,3-butadien-1-yl]benzene (**1h**)

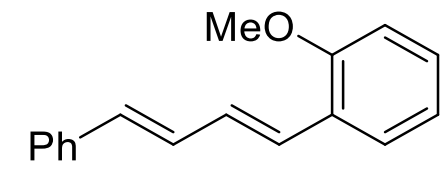

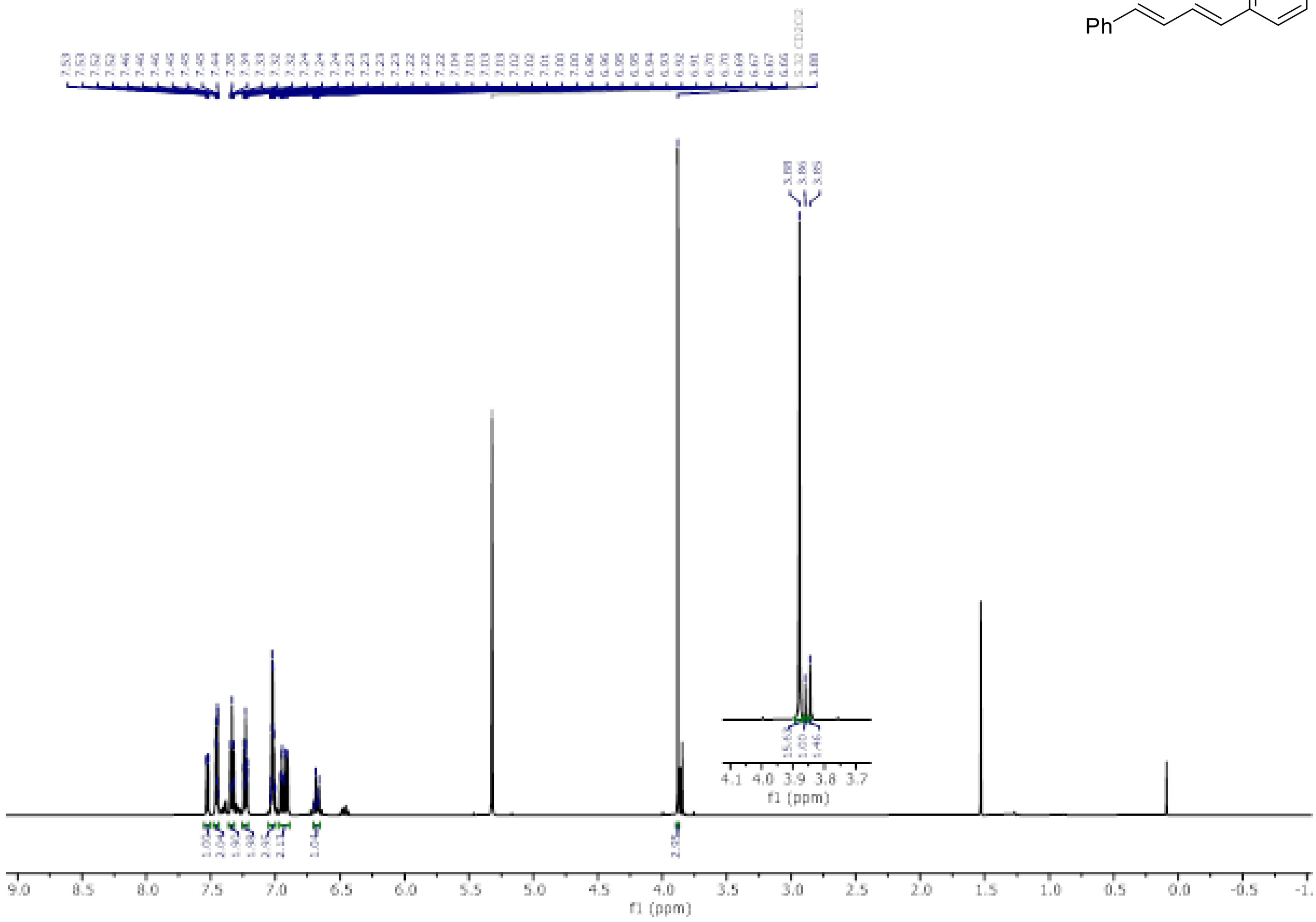

^{1}H NMR spectrum (600 MHz, CD_2Cl_2) of **1h**.

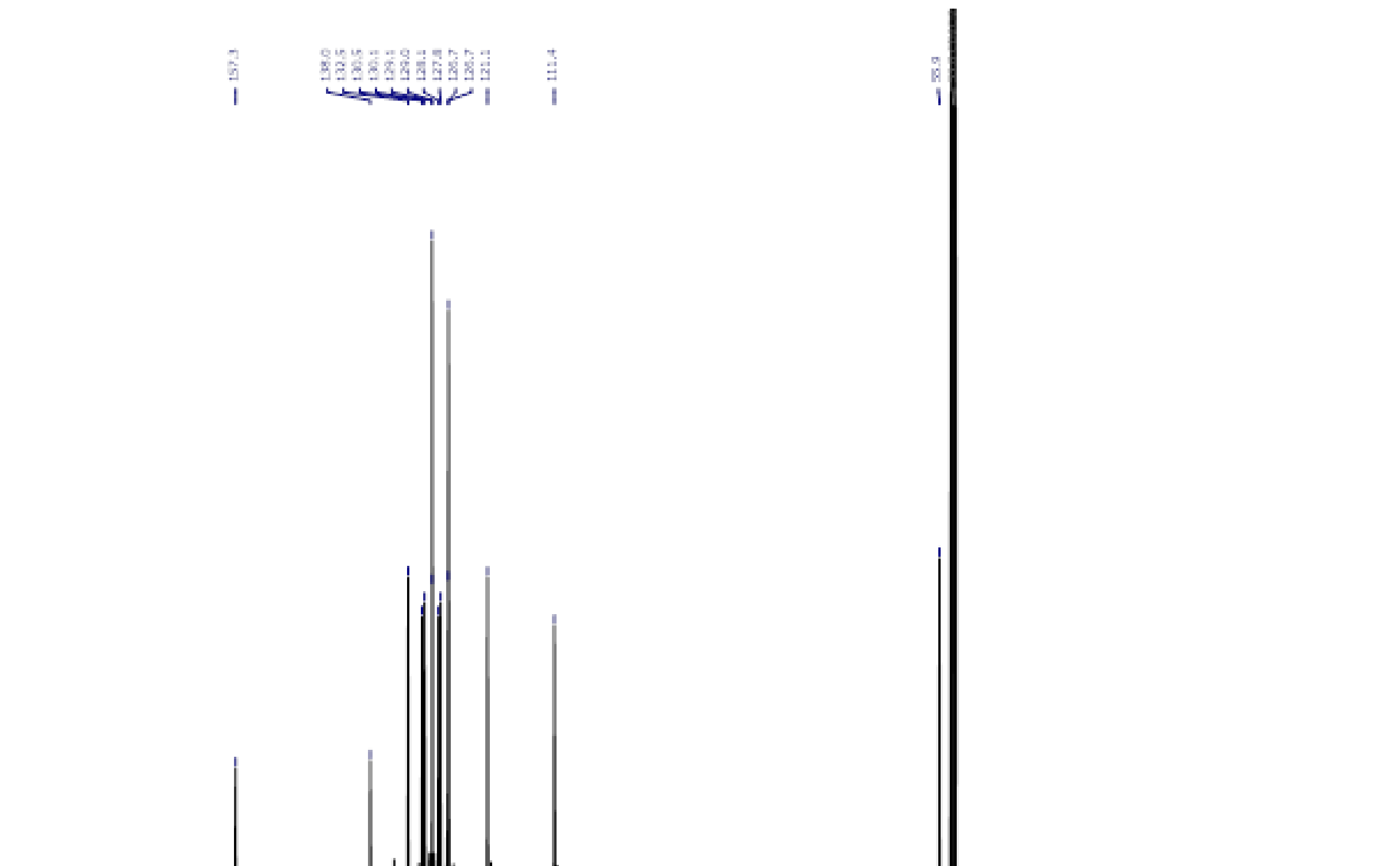

^{13}C NMR spectrum (151 MHz, CD_2Cl_2) of **1h**.

1-Methoxy-3-[(1*E*,3*E*)-4-phenyl-1,3-butadien-1-yl]benzene (**1i**)

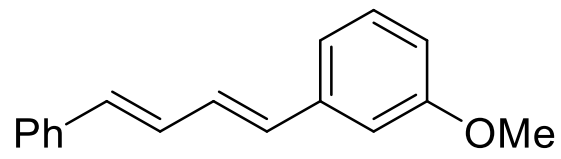

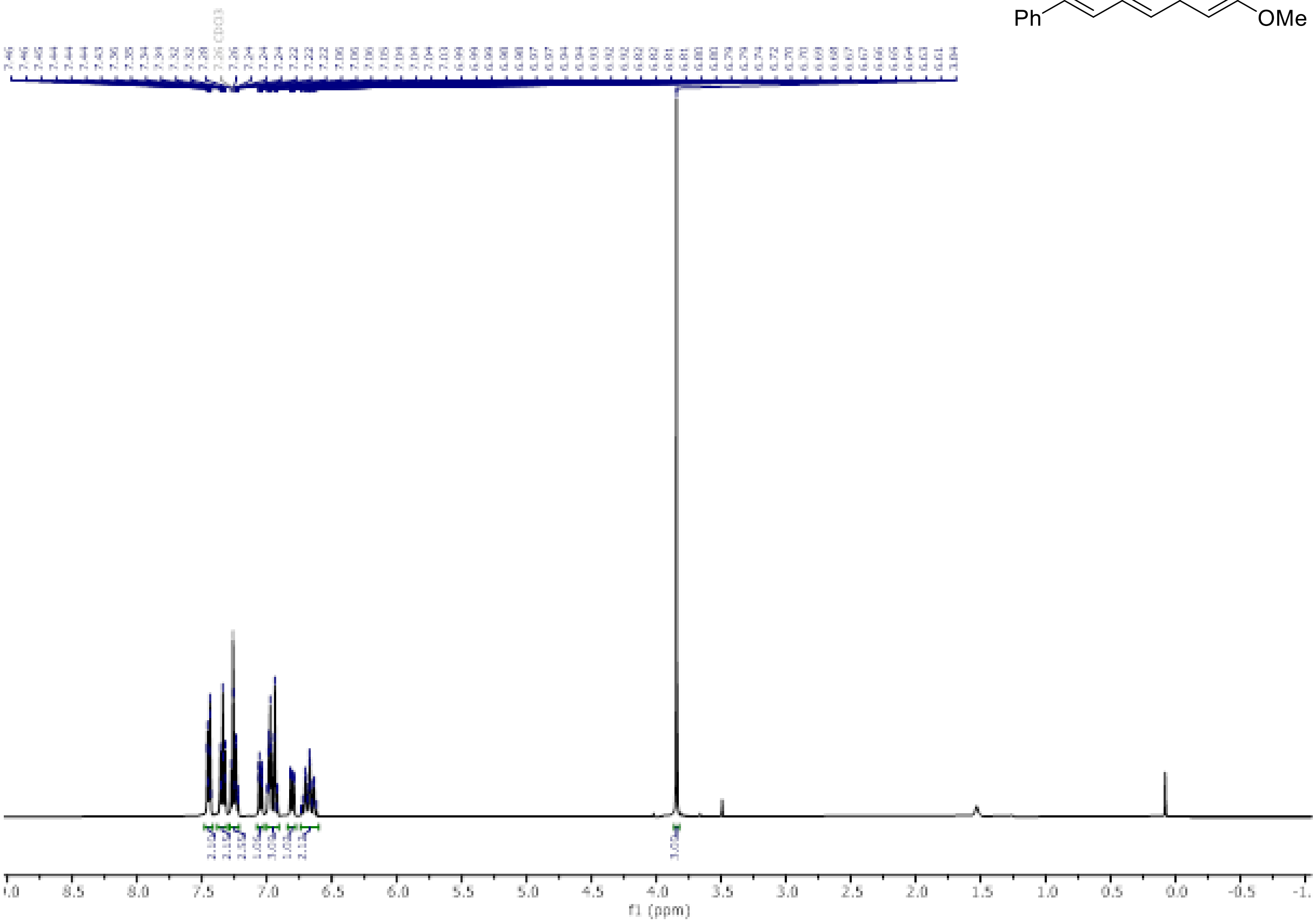

^{1}H NMR spectrum (400 MHz, CD_2Cl_2) of **1i**.

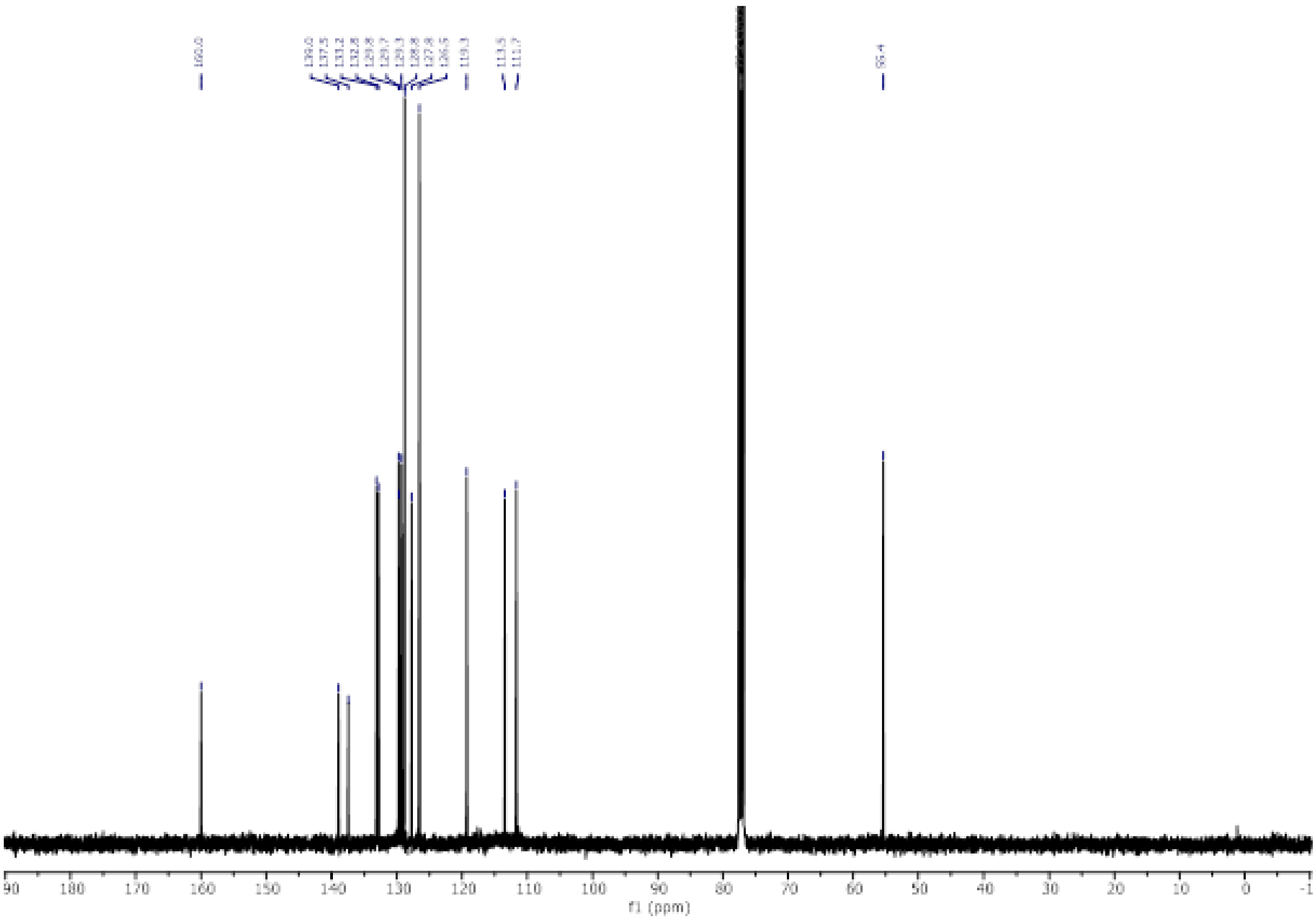

^{13}C NMR spectrum (101 MHz, $CDCl_3$) of **1i**.

1-Methoxy-4-[(1*E*,3*E*)-4-phenyl-1,3-butadien-1-yl]benzene (**1j**)

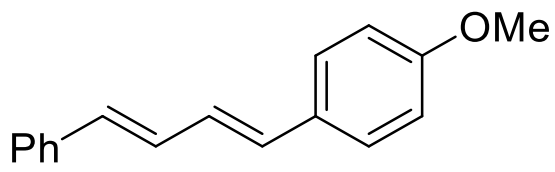

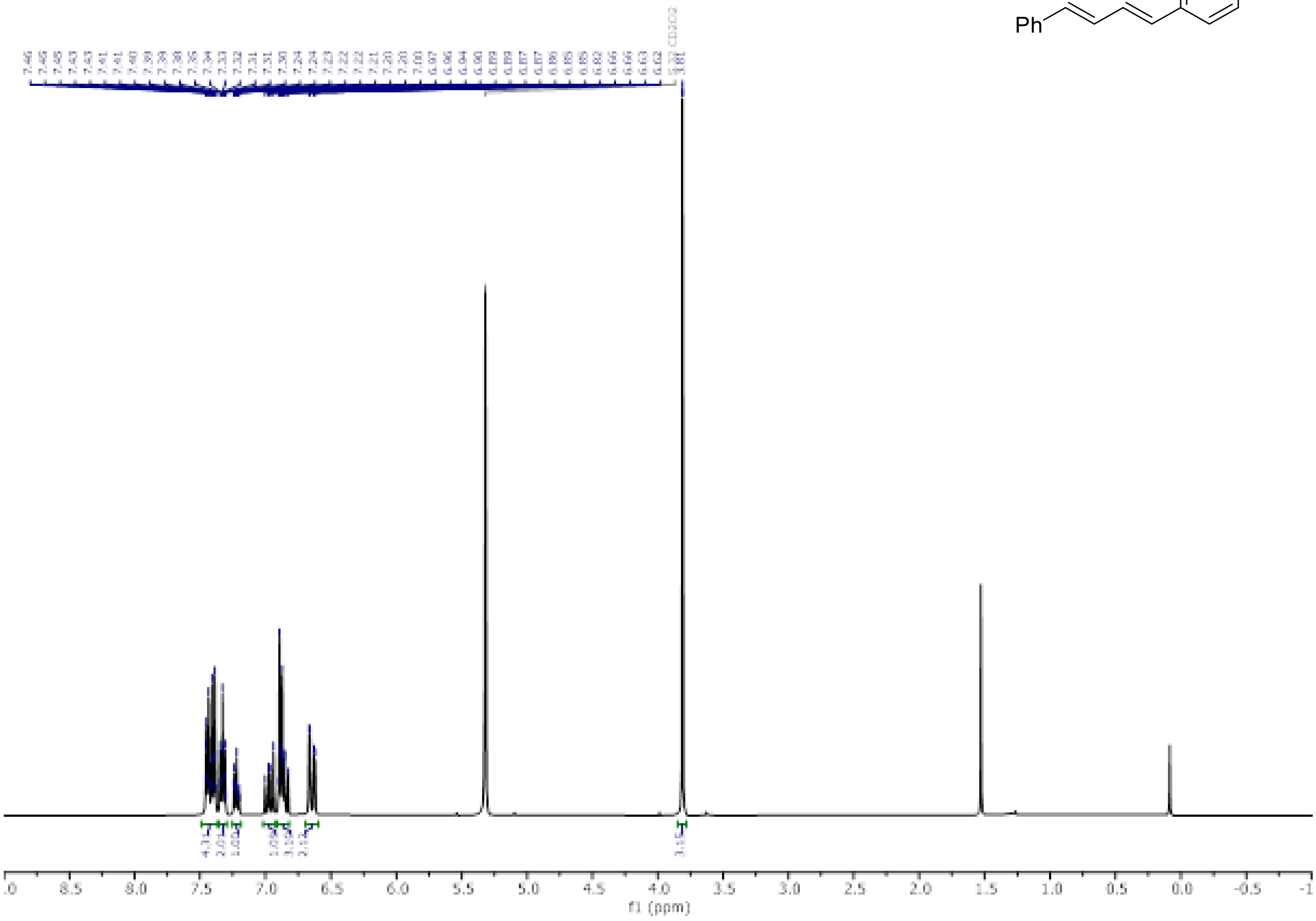

^{1}H NMR spectrum (400 MHz, CD_2Cl_2) of **1j**.

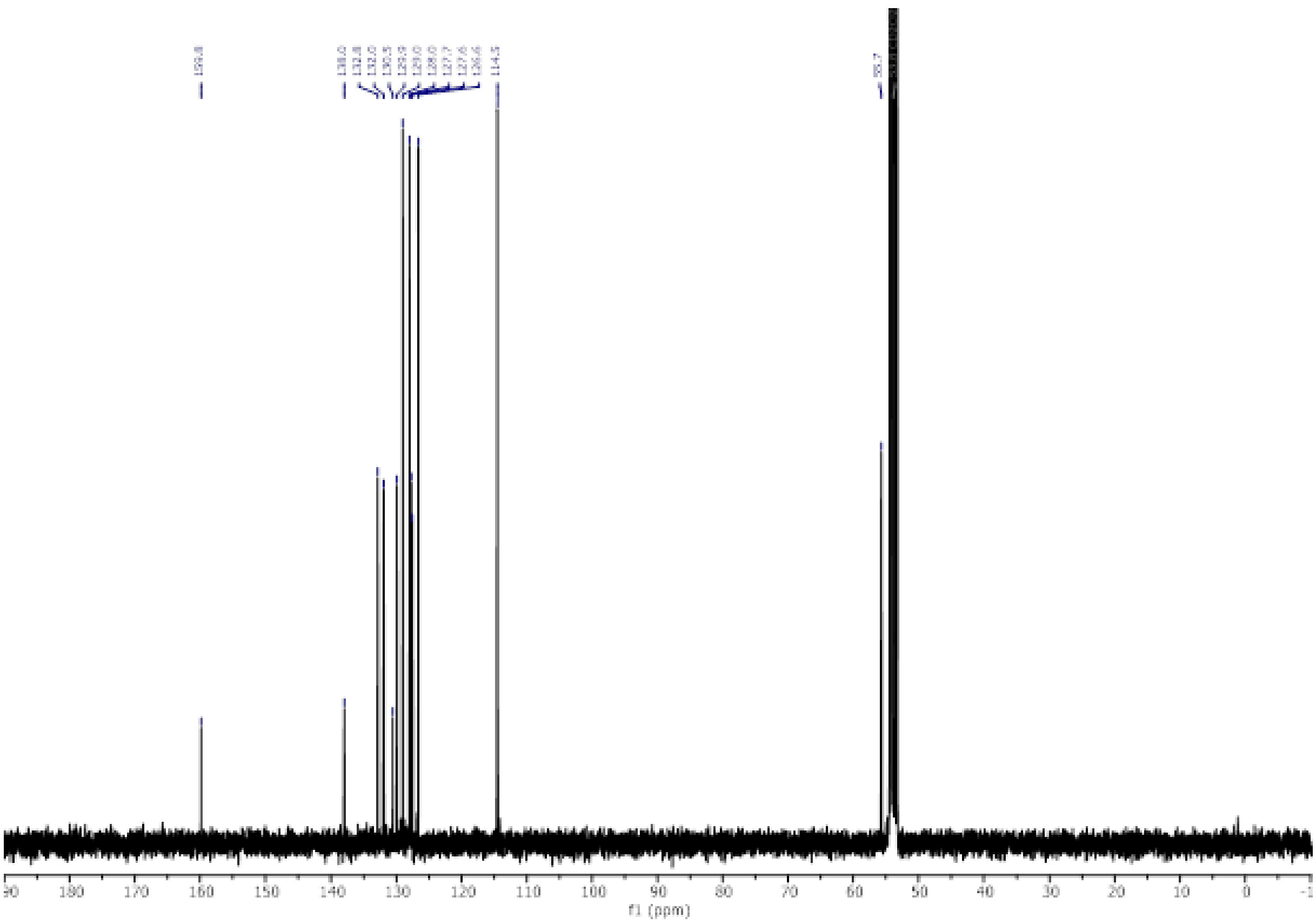

^{13}C NMR spectrum (101 MHz, CD_2Cl_2) of **1j**.

1-[(1*E*,3*E*)-4-phenyl-1,3-butadien-1-yl]naphthalene (**1k**)

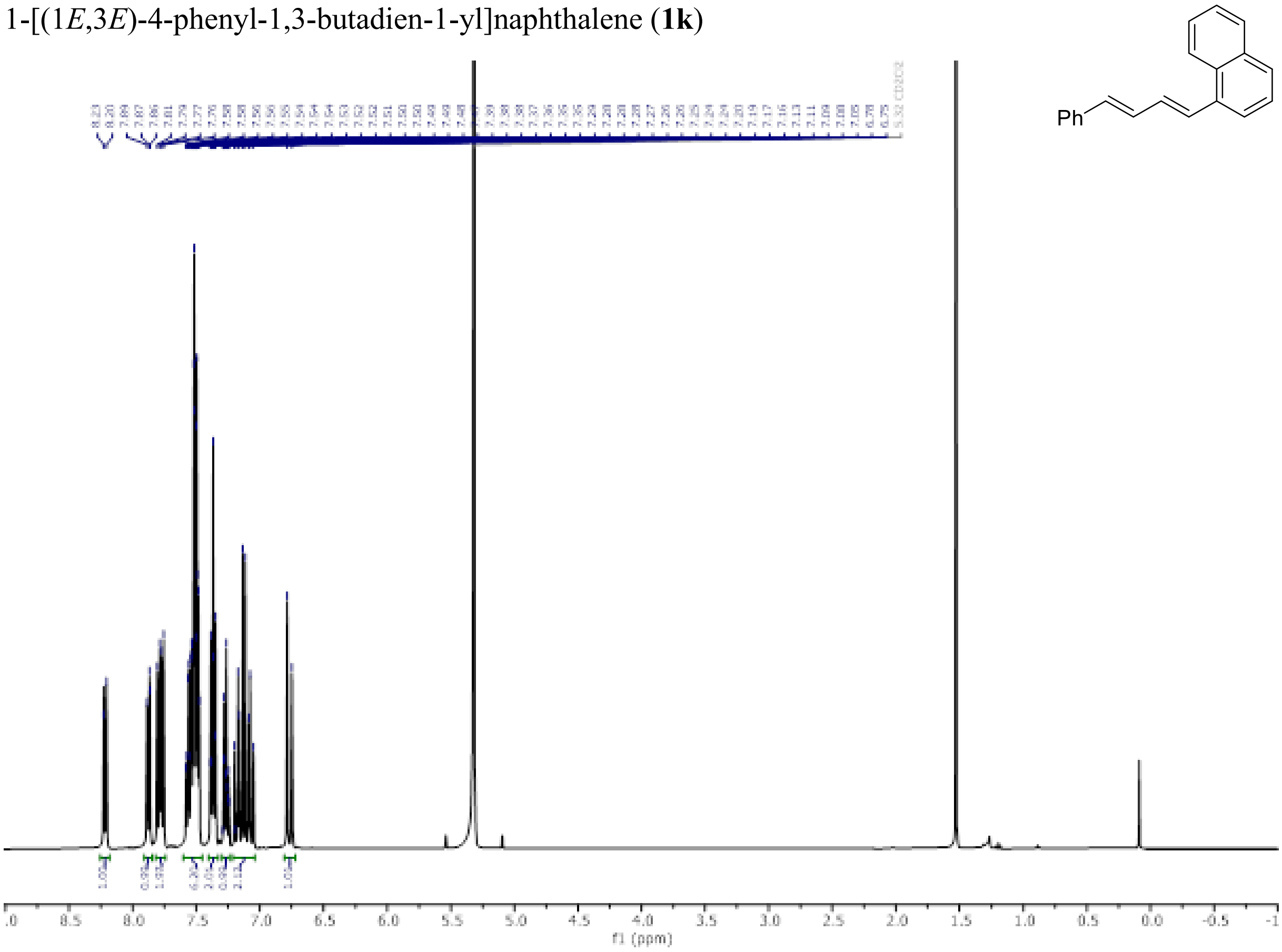

^{1}H NMR spectrum (400 MHz, CD_2Cl_2) of **1k**.

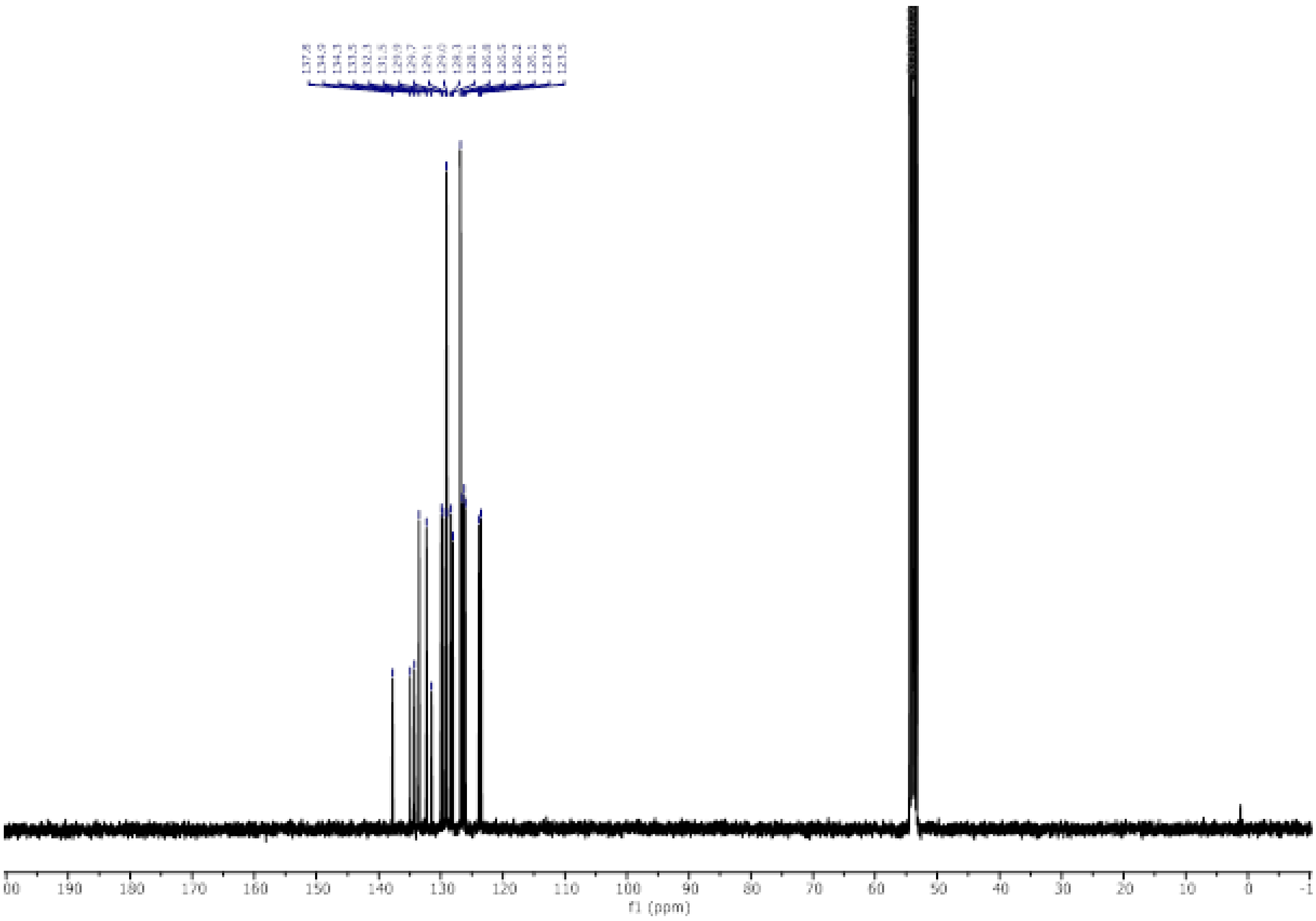

^{13}C NMR spectrum (101 MHz, CD_2Cl_2) of **1k**.

2-[(1*E*,3*E*)-4-phenyl-1,3-butadien-1-yl]naphthalene (**1l**)

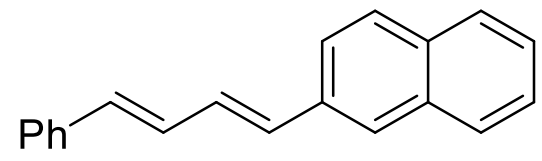

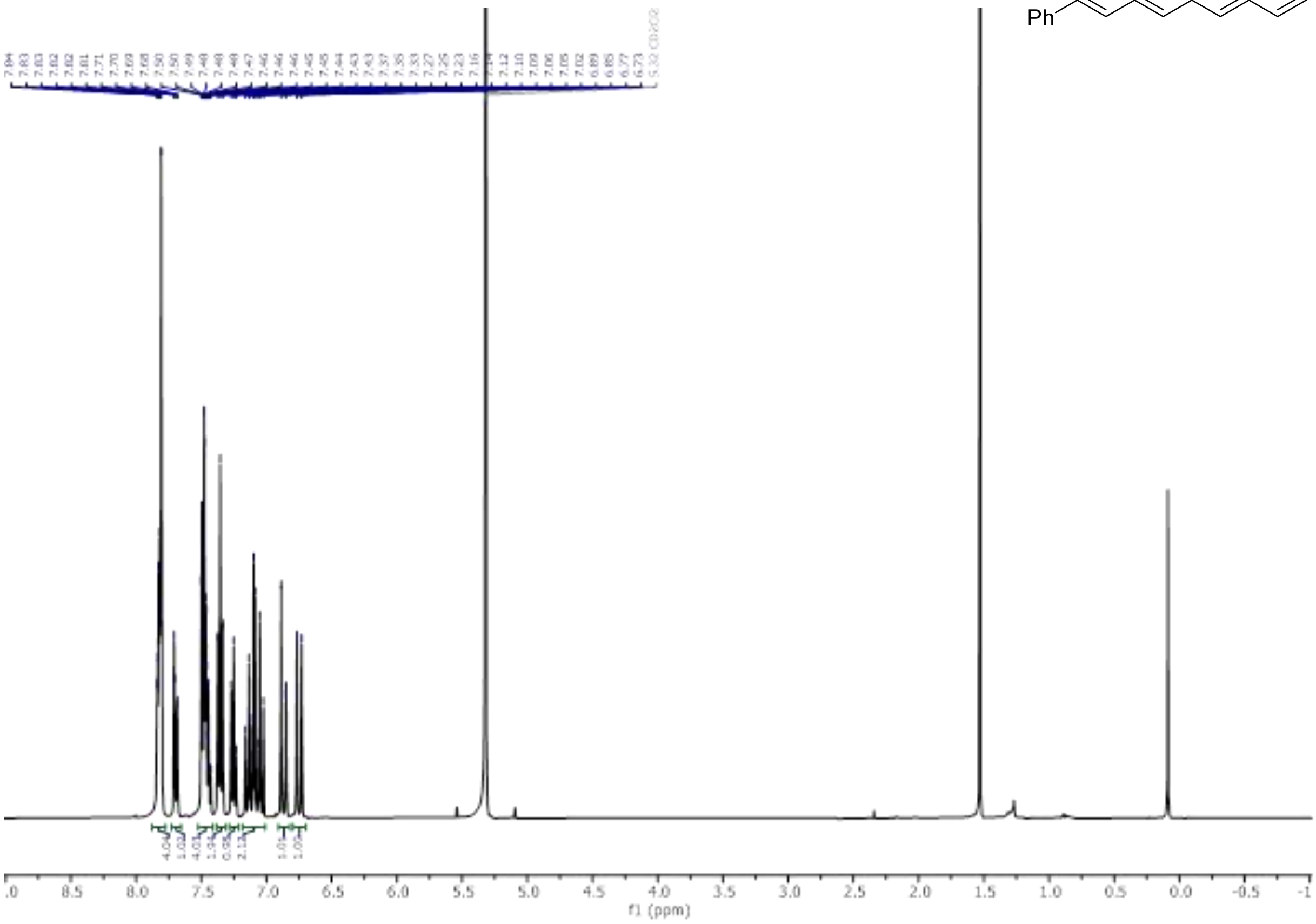

^{1}H NMR spectrum (400 MHz, CD_2Cl_2) of **1l**.

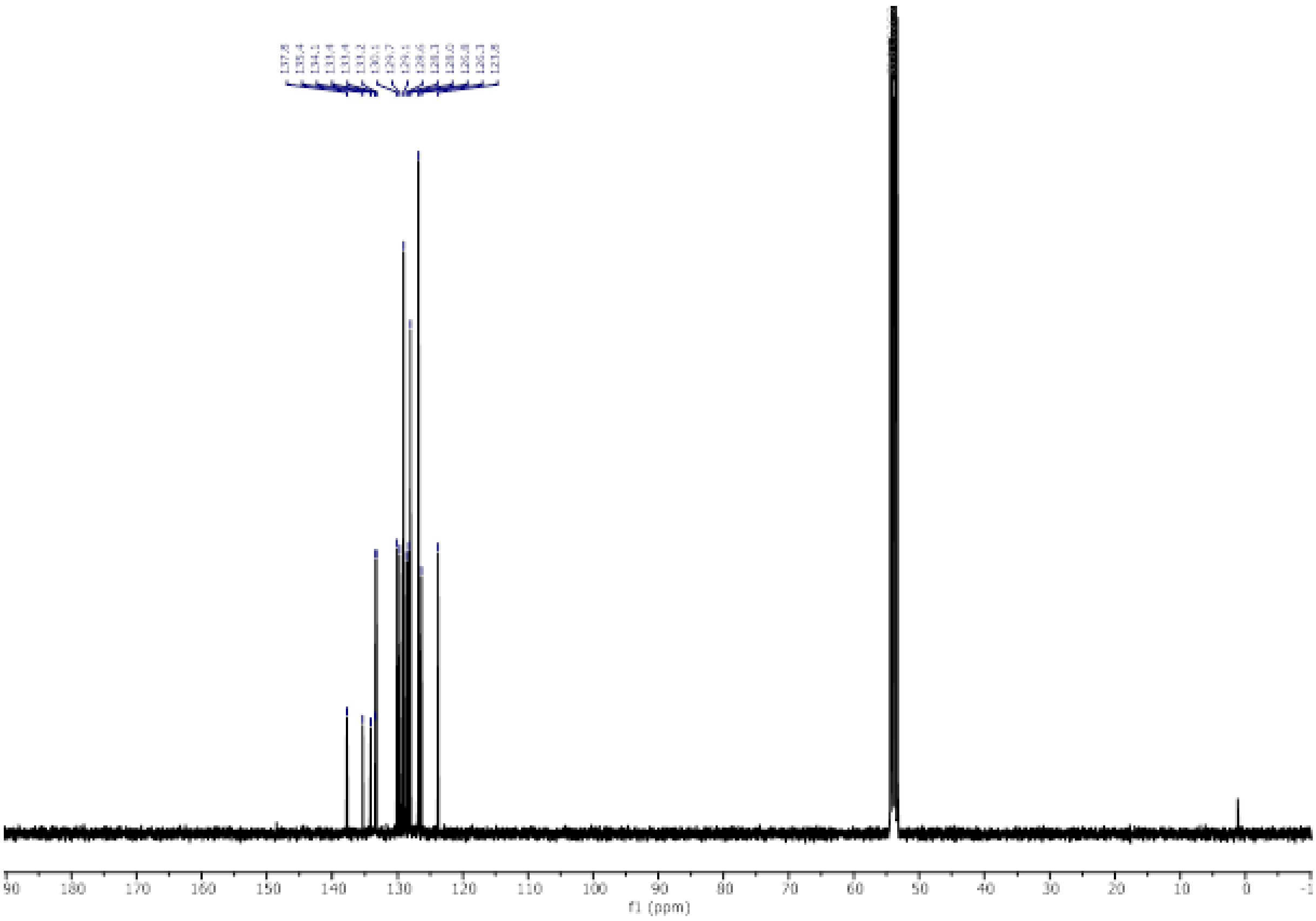

^{13}C NMR spectrum (101 MHz, CD_2Cl_2) of **1l**.

1-[(1*E*,3*E*)-4-phenyl-1,3-butadien-1-yl]pyrene (**1m**)

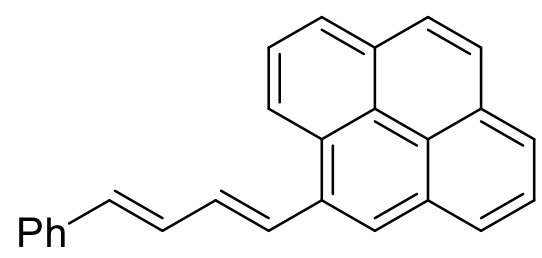

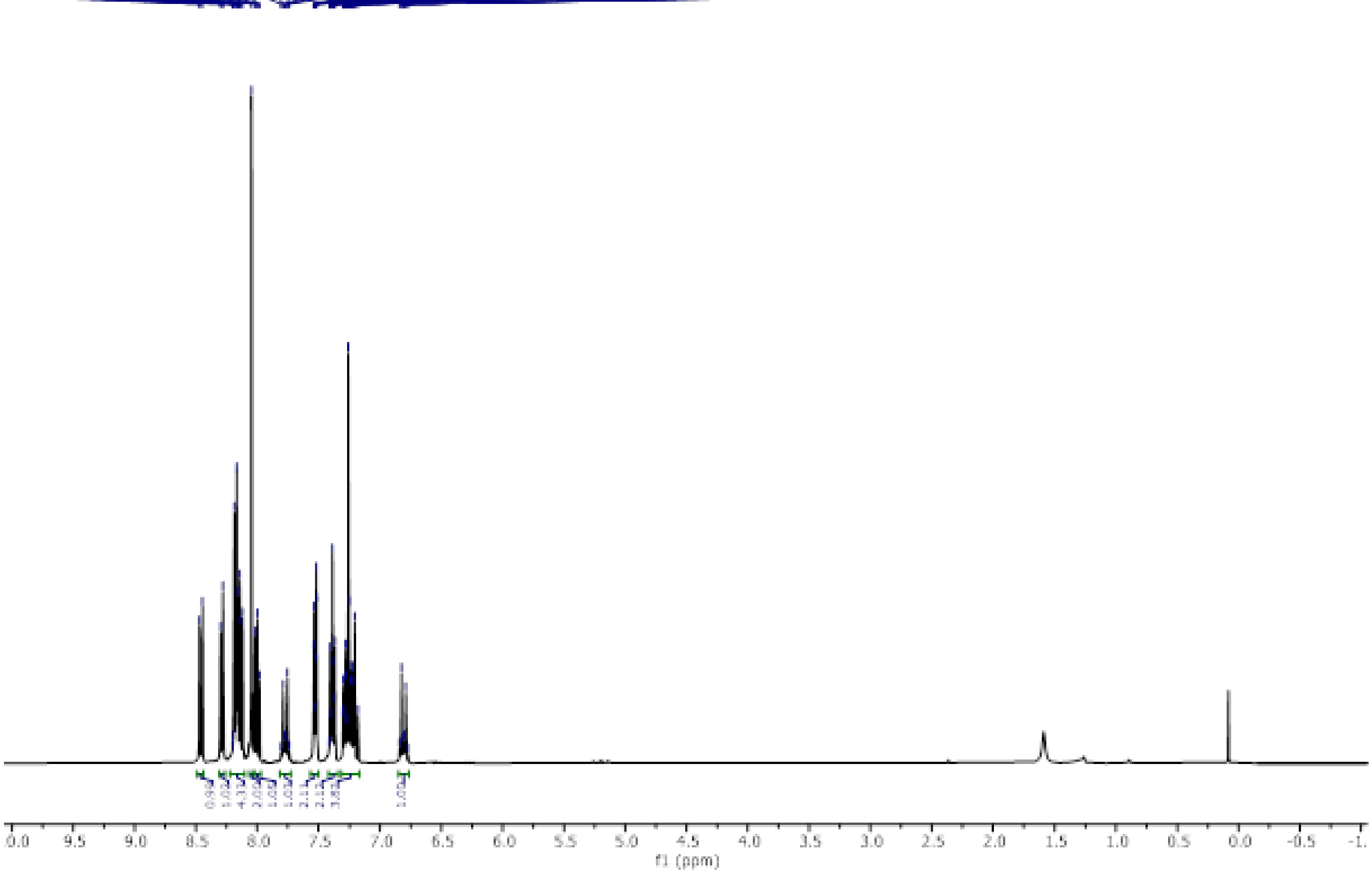

^{1}H NMR spectrum (400 MHz, $CDCl_3$) of **1m**.

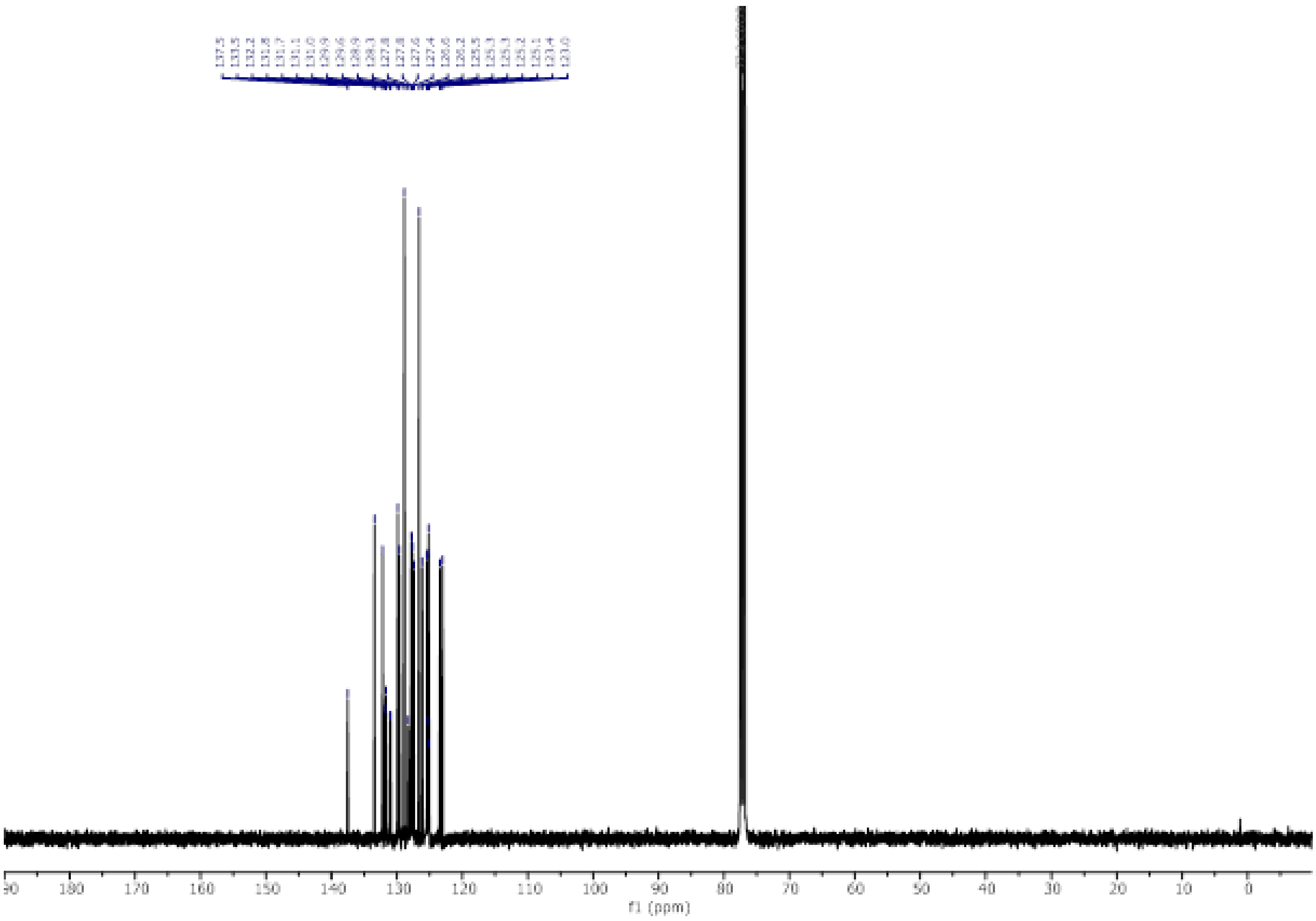

^{13}C NMR spectrum (101 MHz, $CDCl_3$) of **1m**.

1-Chloro-2-[(1*E*,3*E*)-4-pheynl-1,3-butadien-1-yl]benzene (**1n**)

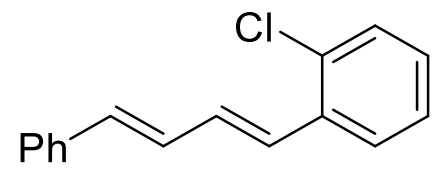

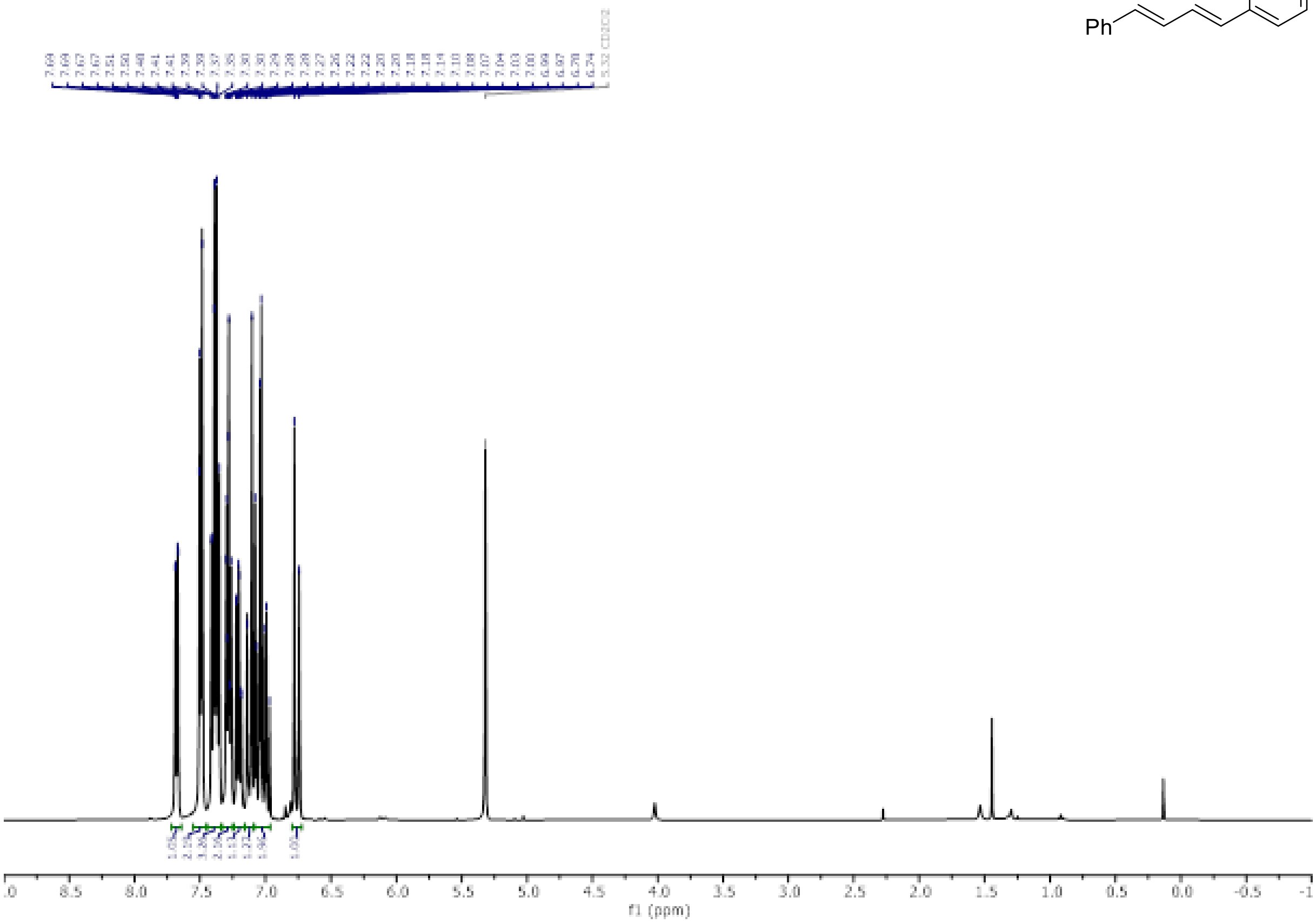

^{1}H NMR spectrum (400 MHz, CD_2Cl_2) of **1n**.

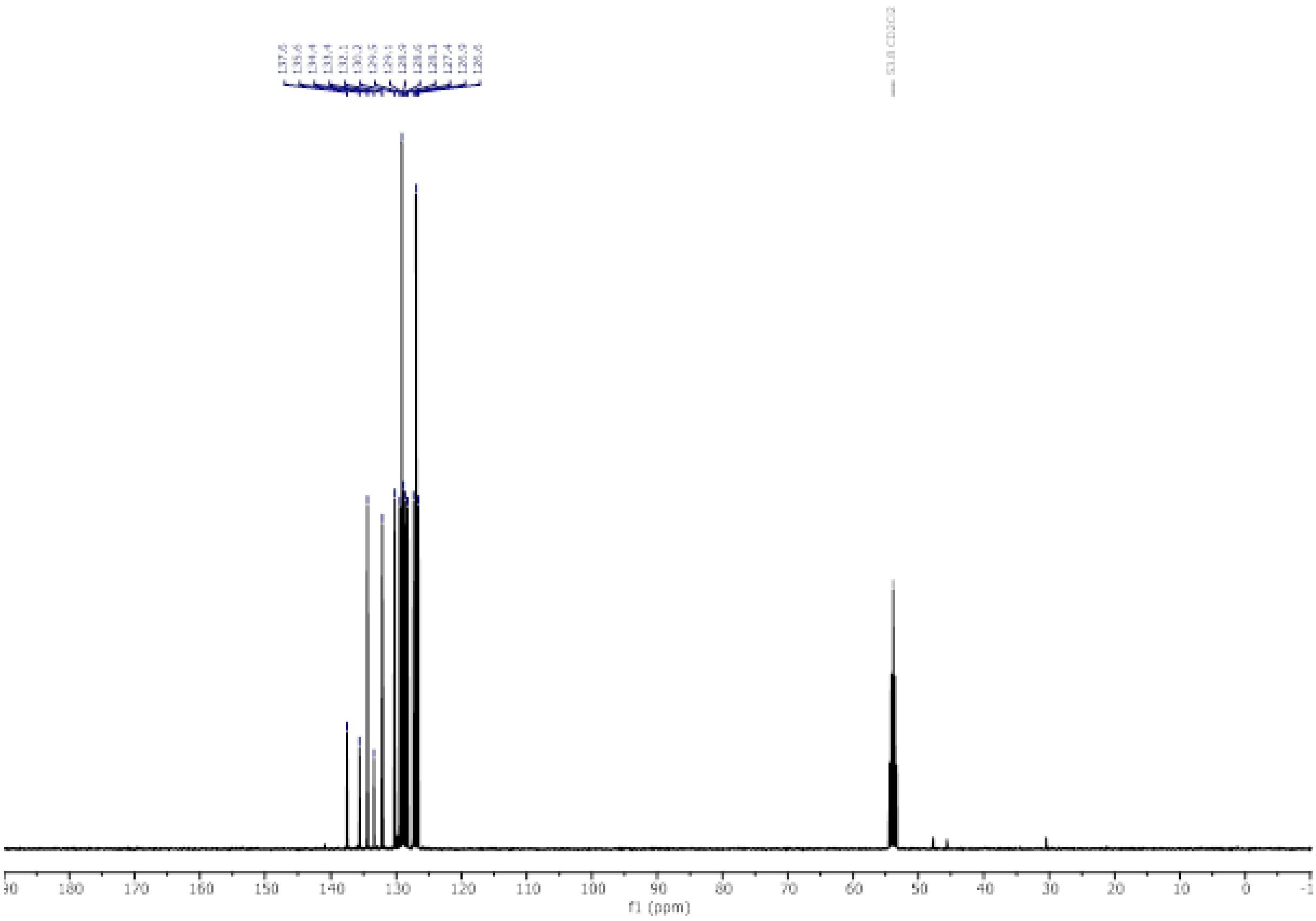

^{13}C NMR spectrum (101 MHz, CD_2Cl_2) of **1n**.

1-Chloro-4-[(1*E*,3*E*)-4-phenyl-1,3-butadien-1-yl]benzene (**1o**)

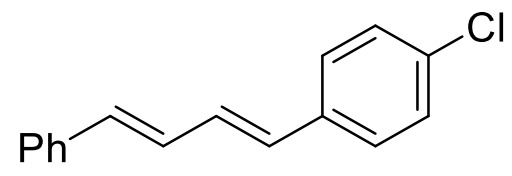

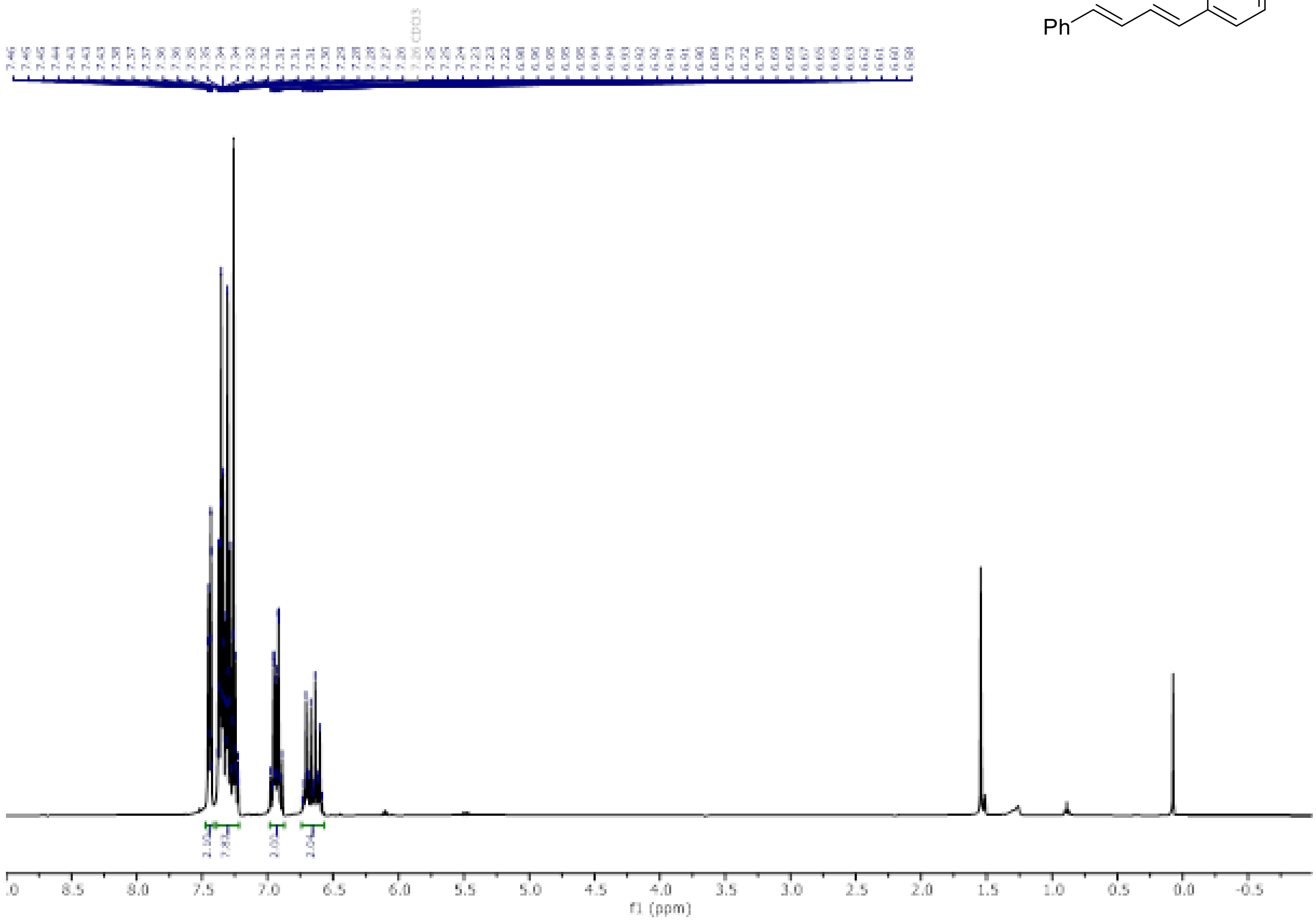

^{1}H NMR spectrum (400 MHz, $CDCl_3$) of **1o**.

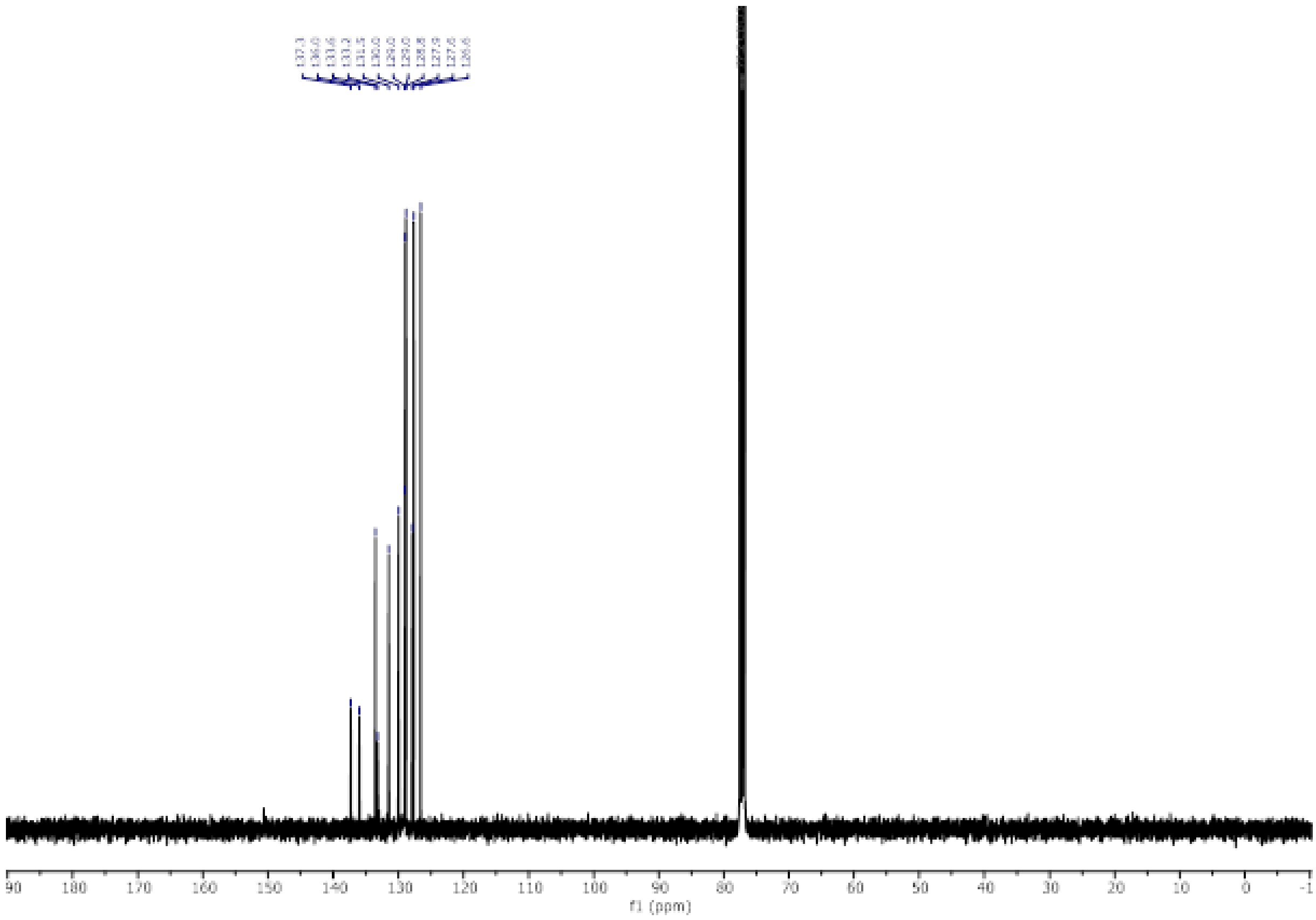

^{13}C NMR spectrum (101 MHz, $CDCl_3$) of **1o**.

1-Bromo-2-[(1*E*,3*E*)-4-phenyl-1,3-butadien-1-yl]benzene (**1p**)

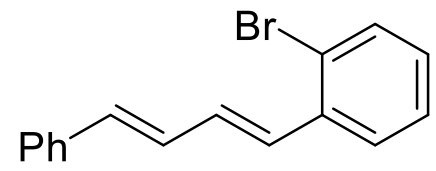

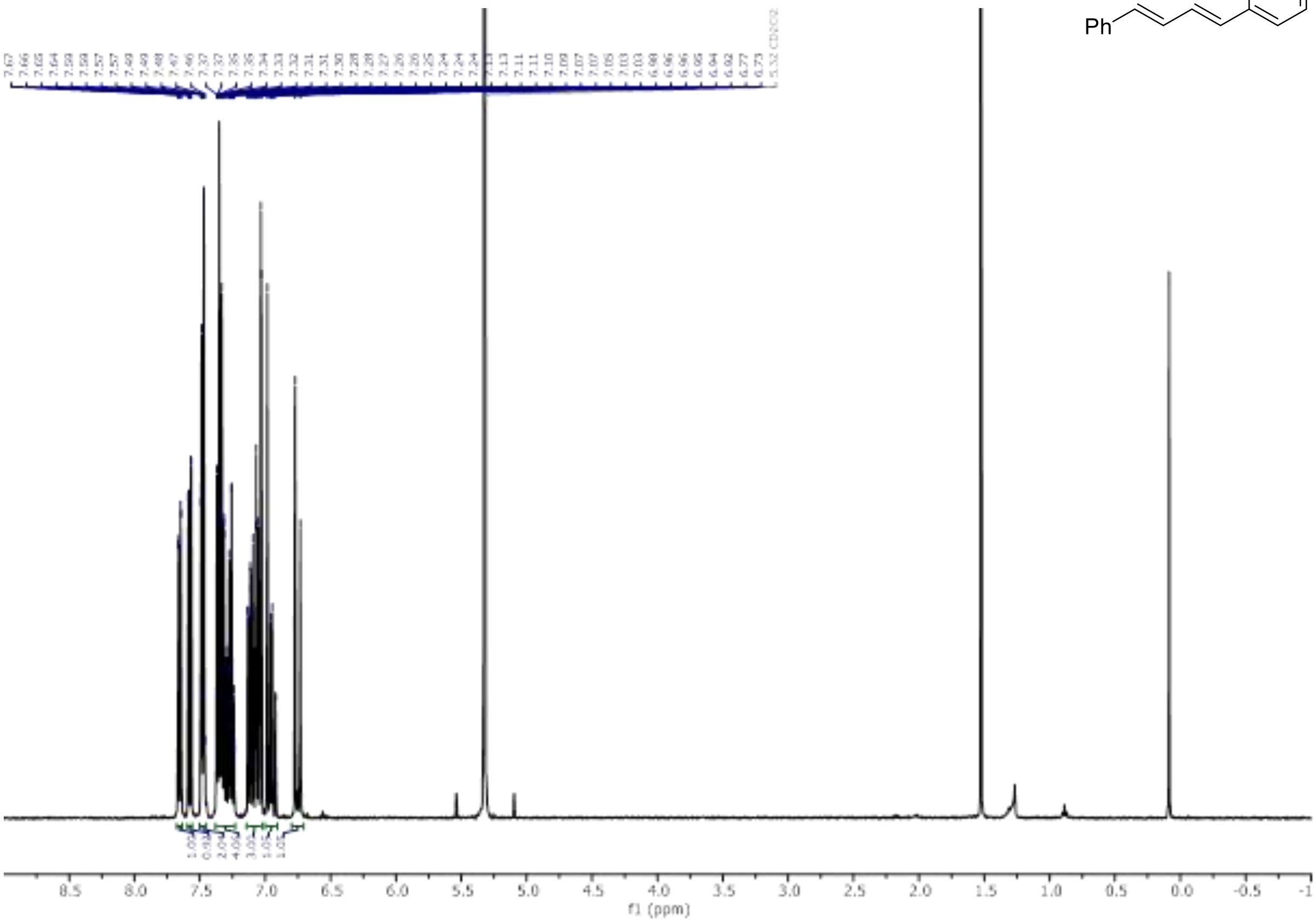

^{1}H NMR spectrum (400 MHz, CD_2Cl_2) of **1p**.

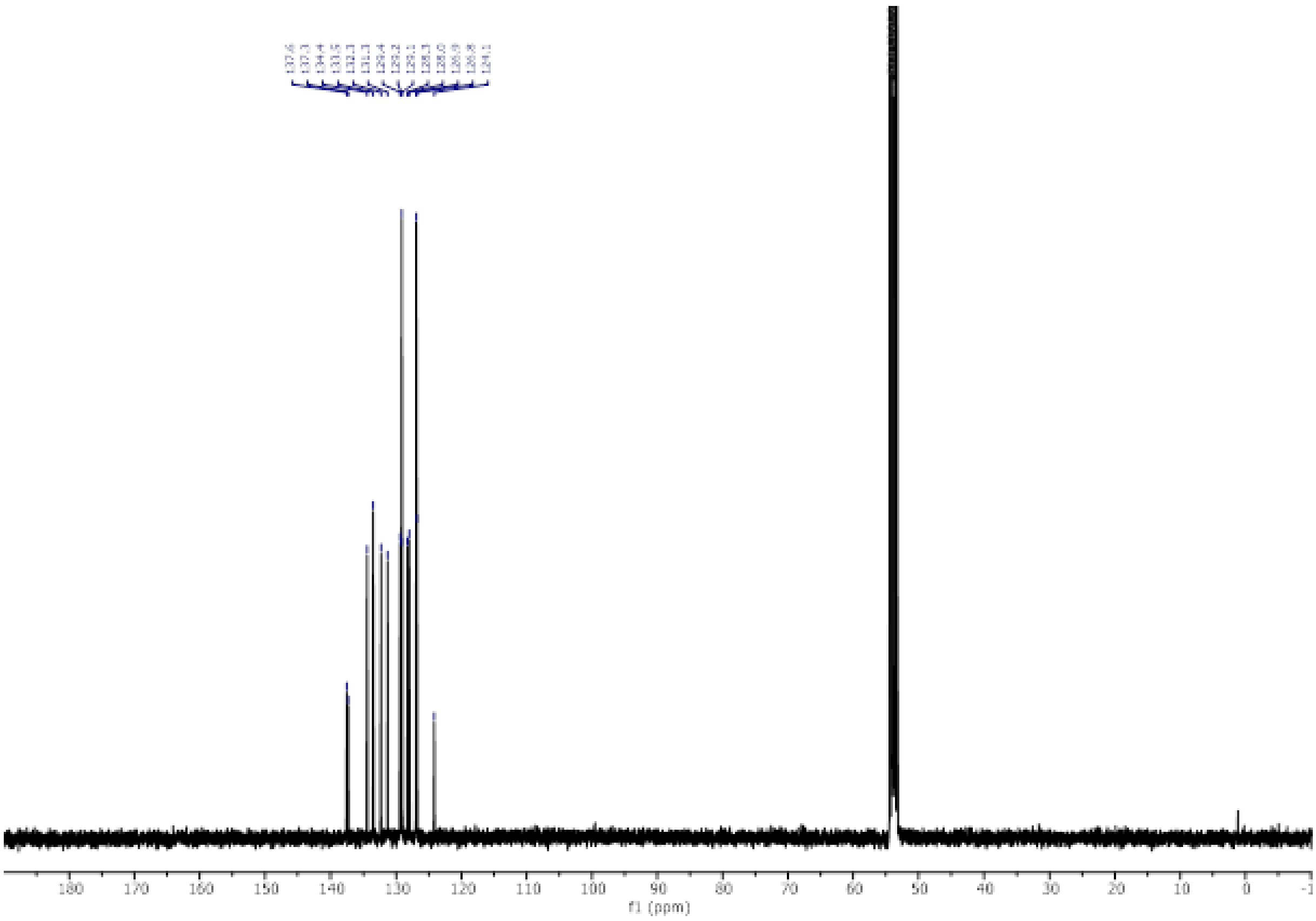

^{13}C NMR spectrum (101 MHz, CD_2Cl_2) of **1p**.

1-Bromo-3-[(1*E*,3*E*)-4-phenyl-1,3-butadien-1-yl]benzene (**1q**)

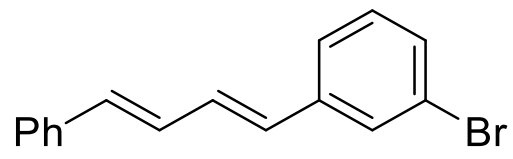

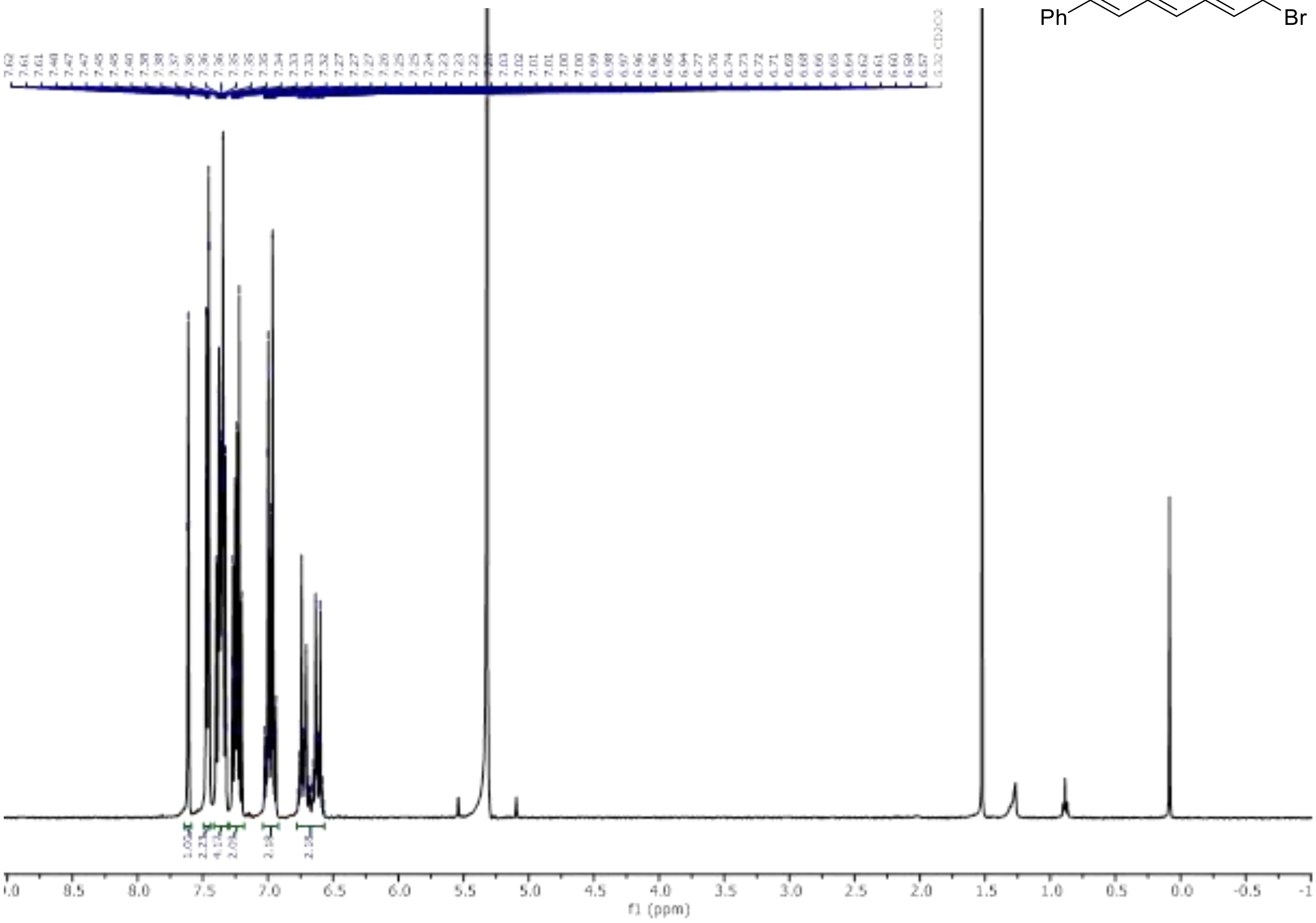

^{1}H NMR spectrum (400 MHz, CD_2Cl_2) of **1q**.

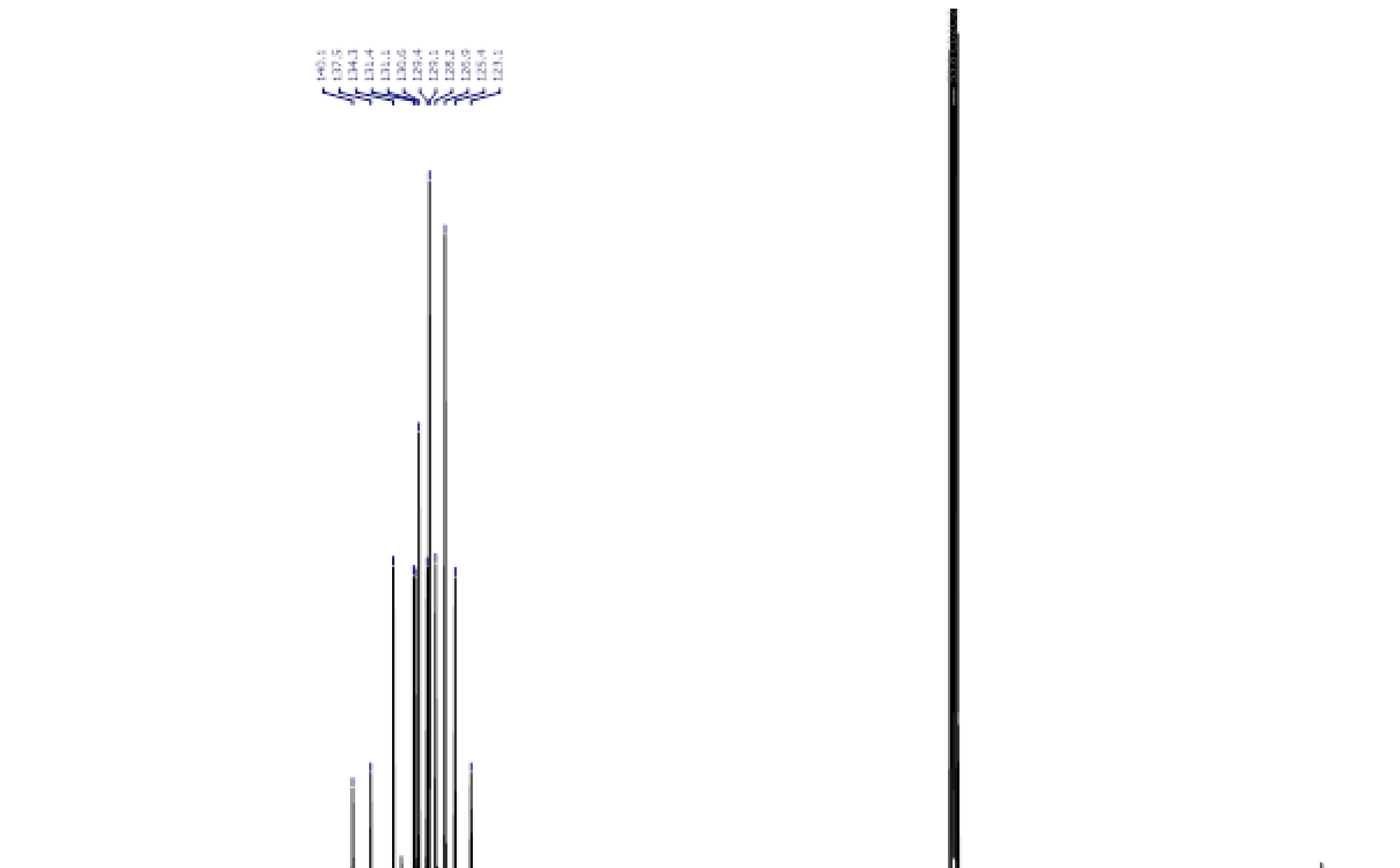

^{13}C NMR spectrum (101 MHz, CD_2Cl_2) of **1q**.

1-Bromo-4-[(1*E*,3*E*)-4-phenyl-1,3-butadien-1-yl]benzene (**1r**)

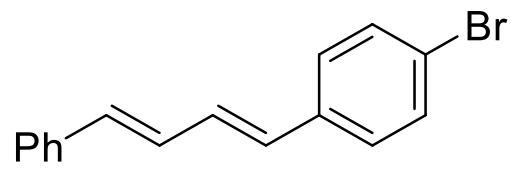

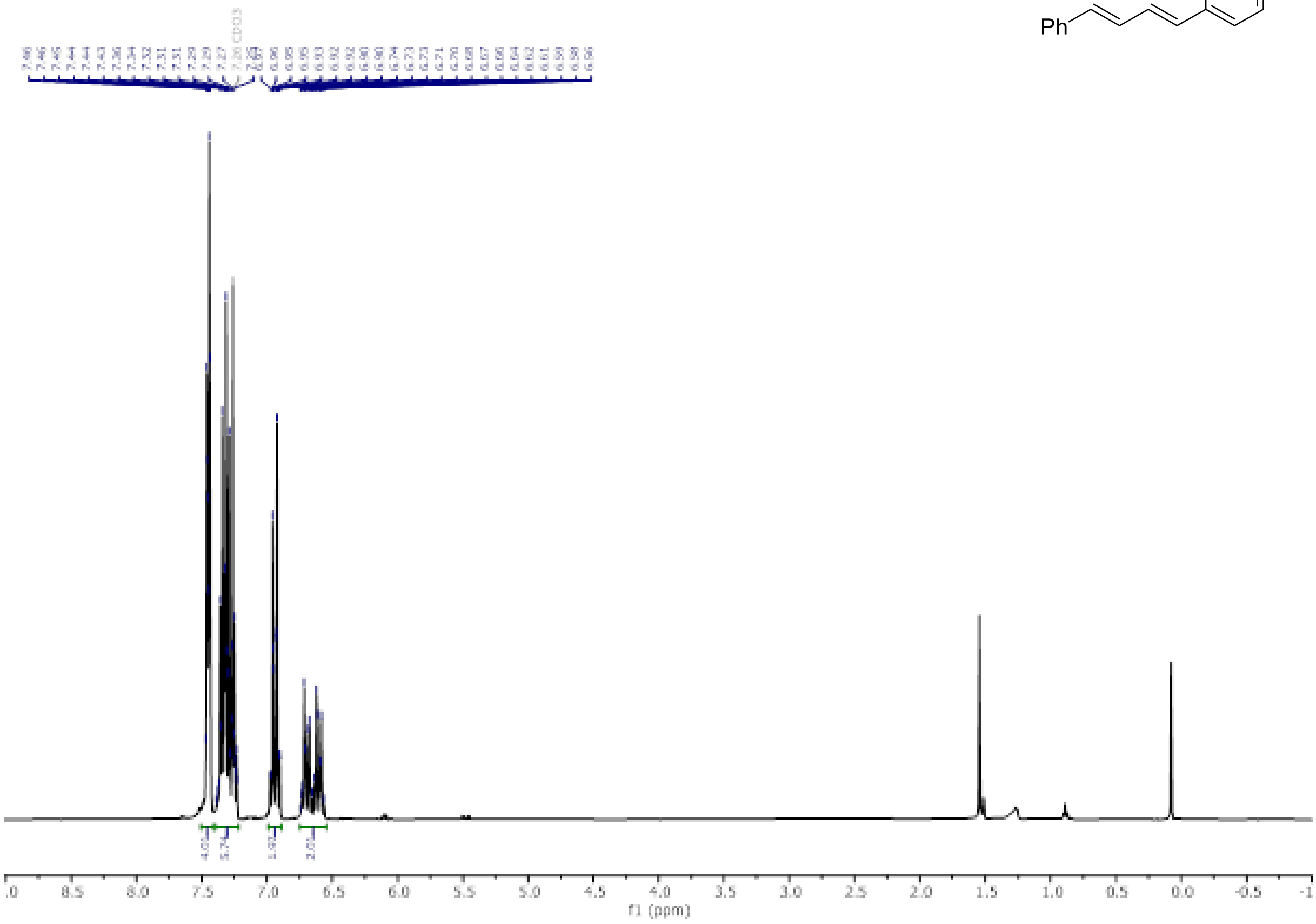

^{1}H NMR spectrum (400 MHz, $CDCl_3$) of **1r**.

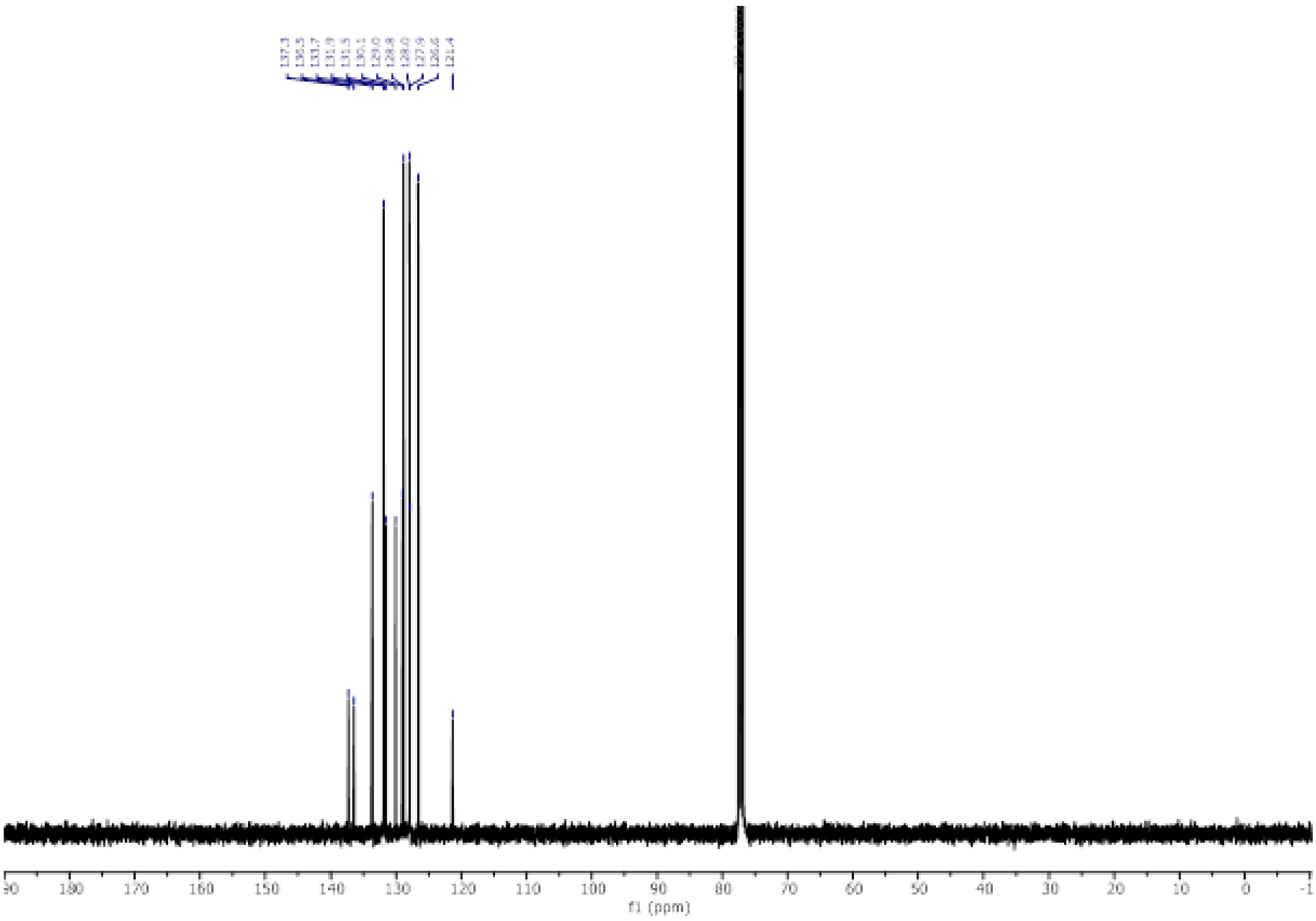

^{13}C NMR spectrum (101 MHz, $CDCl_3$) of **1r**.

1-Iodo-4-[(1*E*,3*E*)-4-phenyl-1,3-butadien-1-yl]benzene (**1s**)

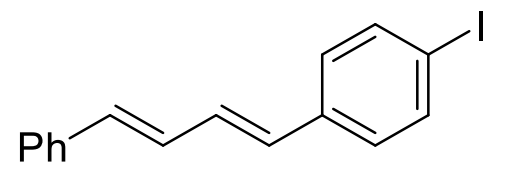

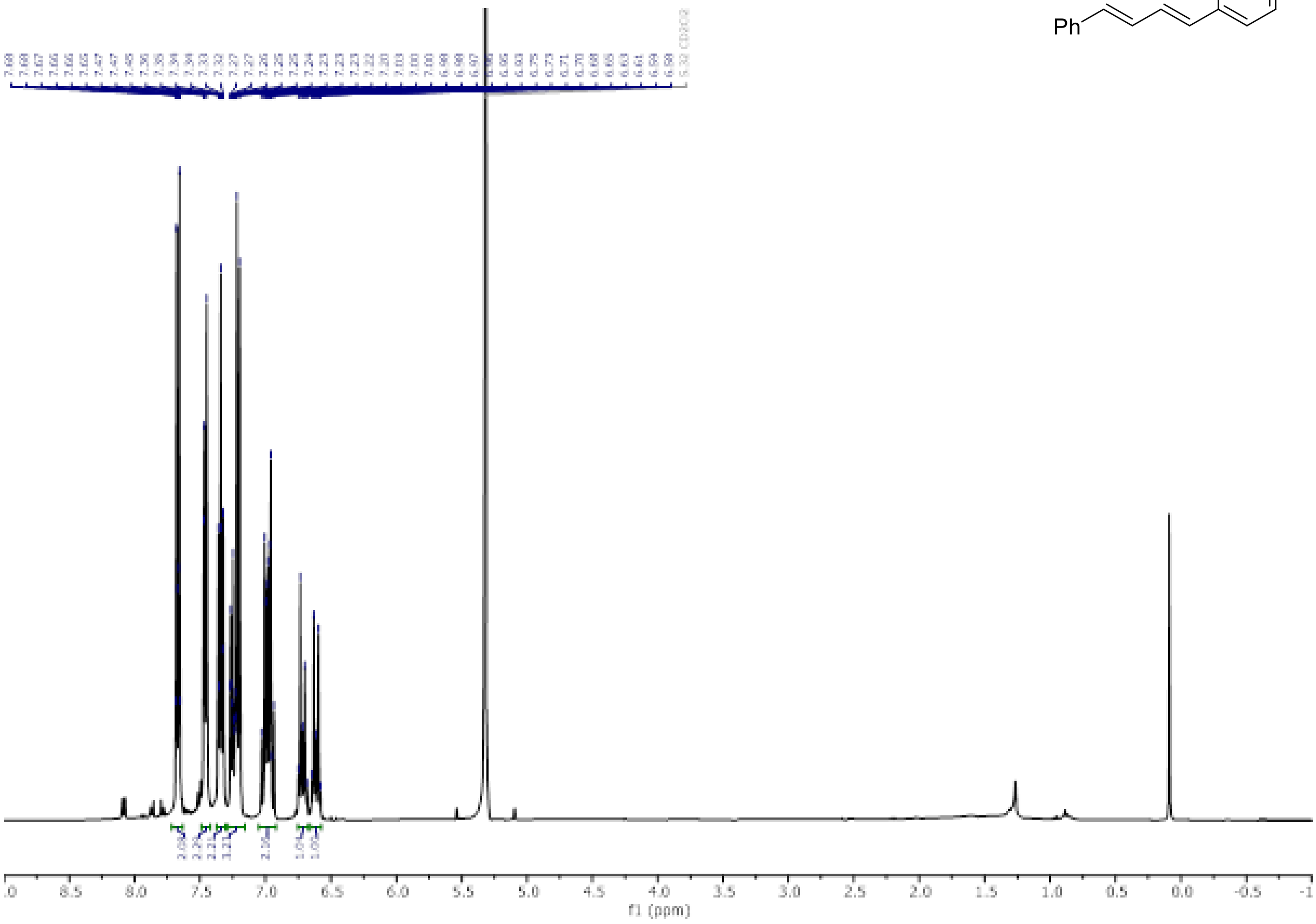

^{1}H NMR spectrum (400 MHz, CD_2Cl_2) of **1s**.

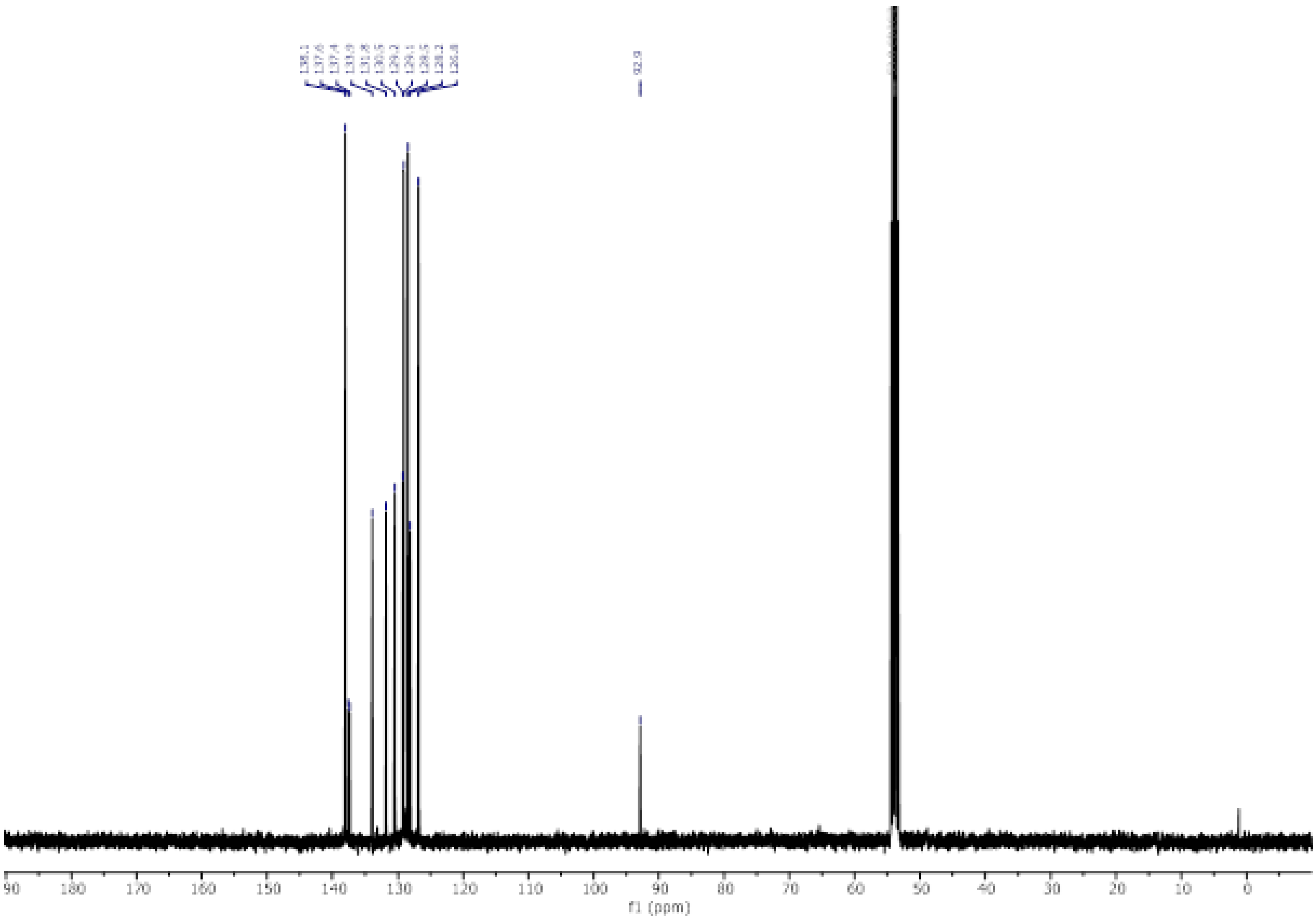

^{13}C NMR spectrum (101 MHz, CD_2Cl_2) of **1s**.

1-Trifluoromethyl-4-[(1*E*,3*E*)-4-phenyl-1,3-butadien-1-yl]benzene (**1t**)

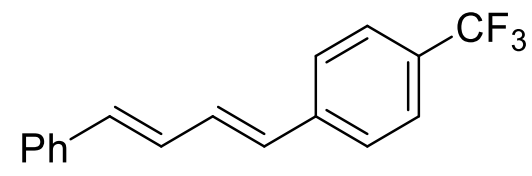

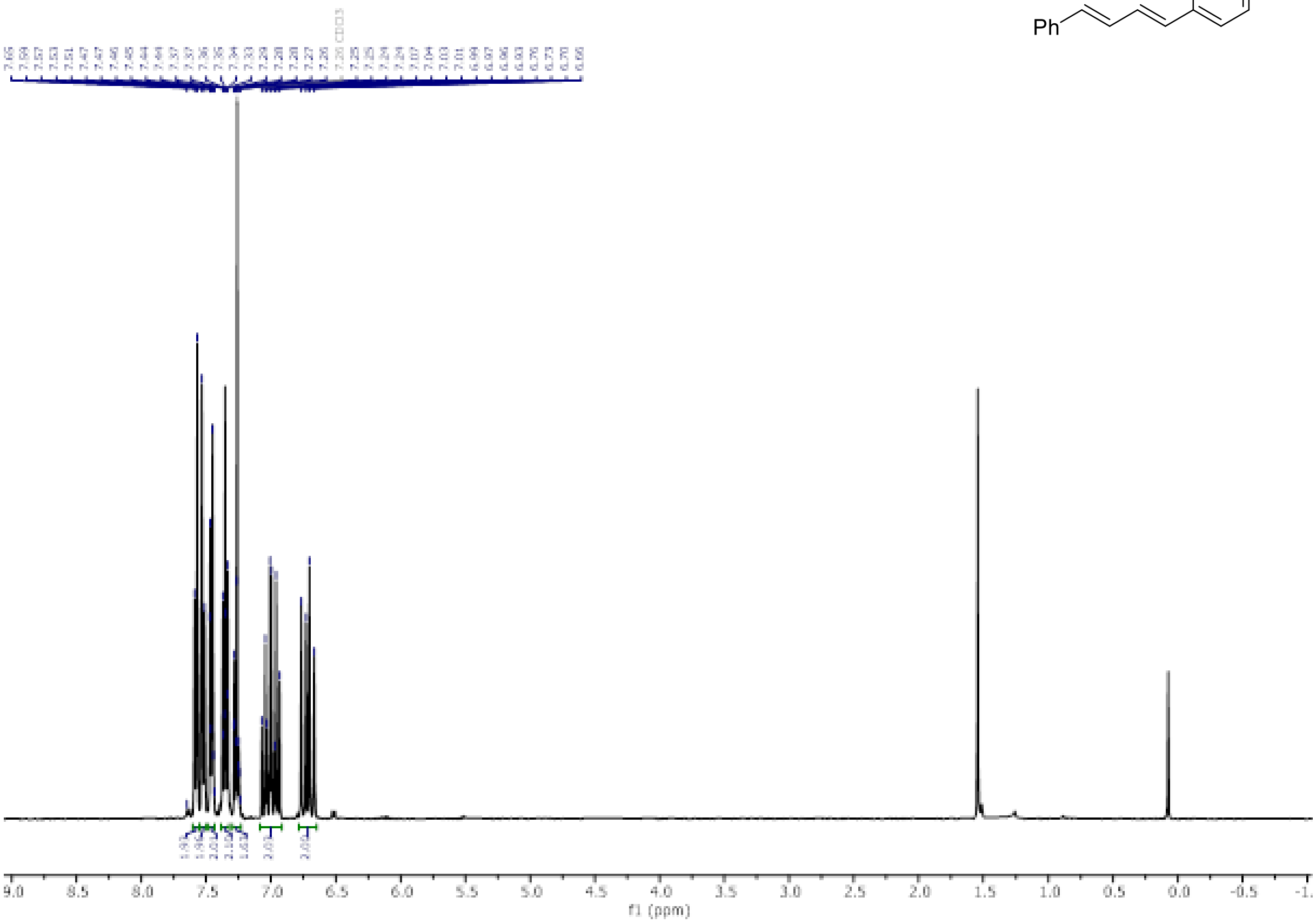

^{1}H NMR spectrum (400 MHz, $CDCl_3$) of **1t**.

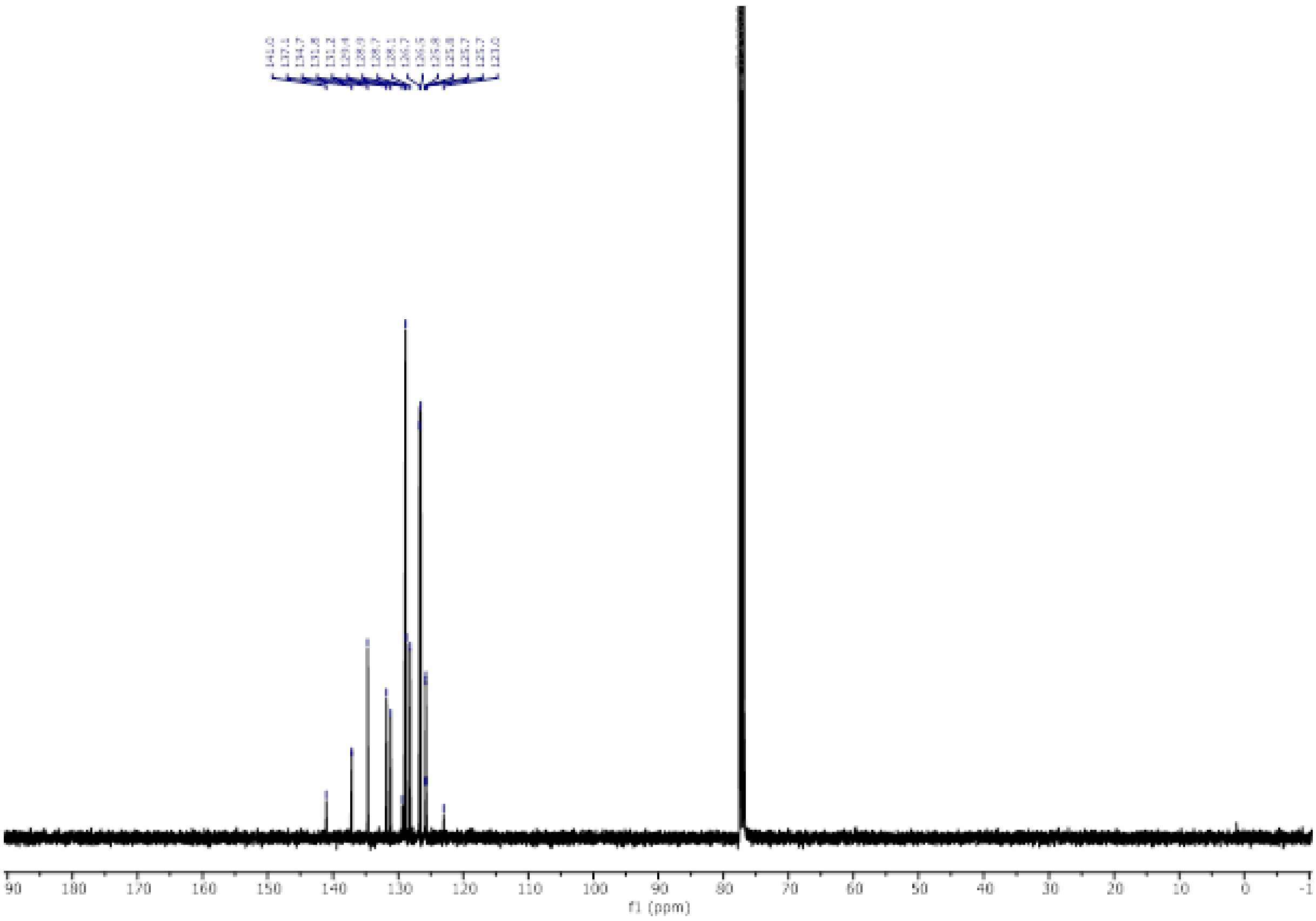

^{13}C NMR spectrum (101 MHz, $CDCl_3$) of **1t**.

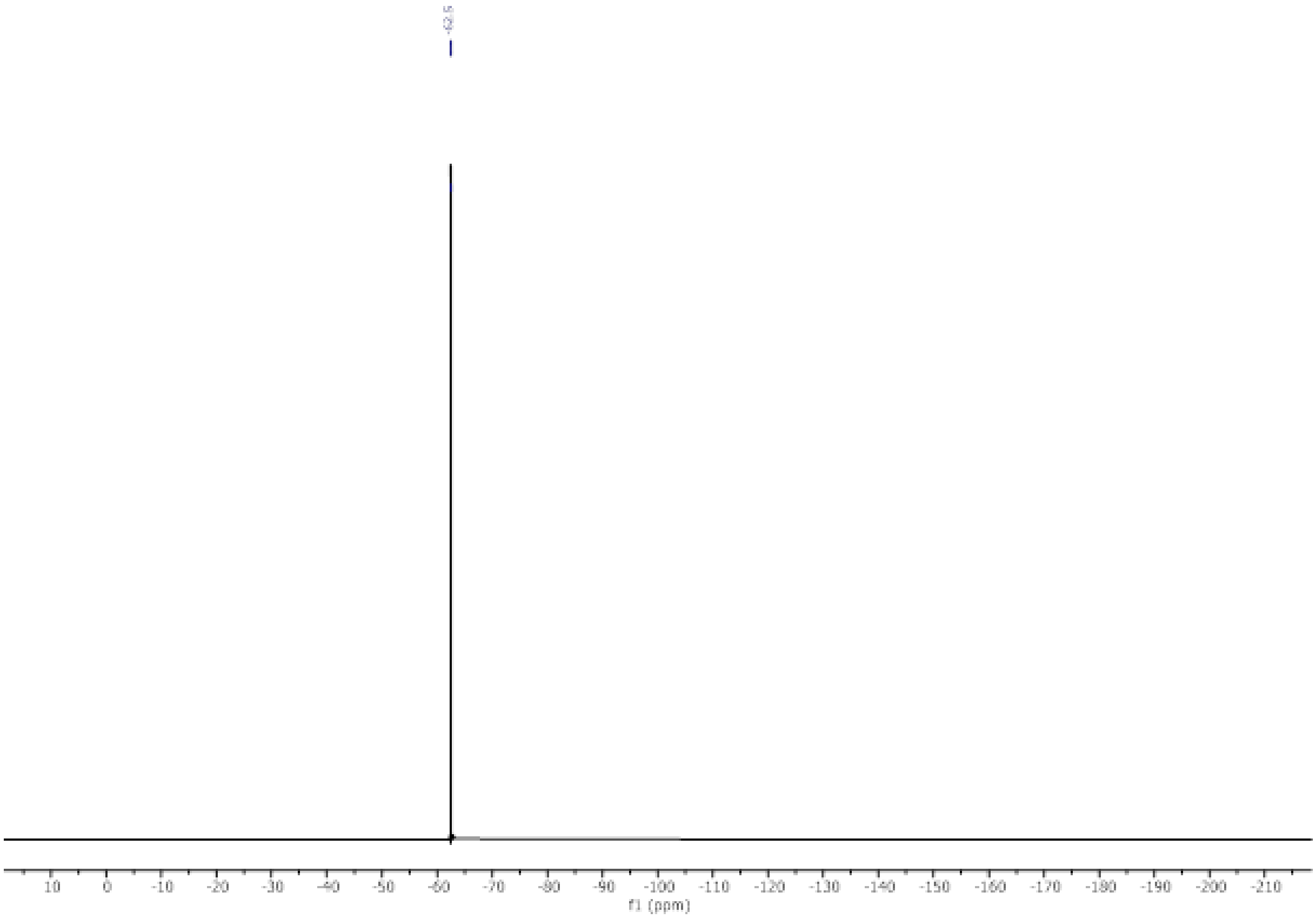

^{19}F NMR spectrum (344 MHz, $CDCl_3$) of **1t**.

Methyl 4-[(1*E*,3*E*)-4-phenyl-1,3-butadien-1-yl]benzoate (**1u**)

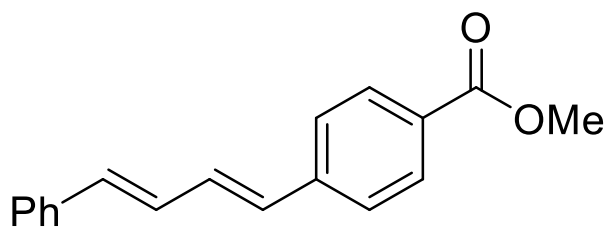

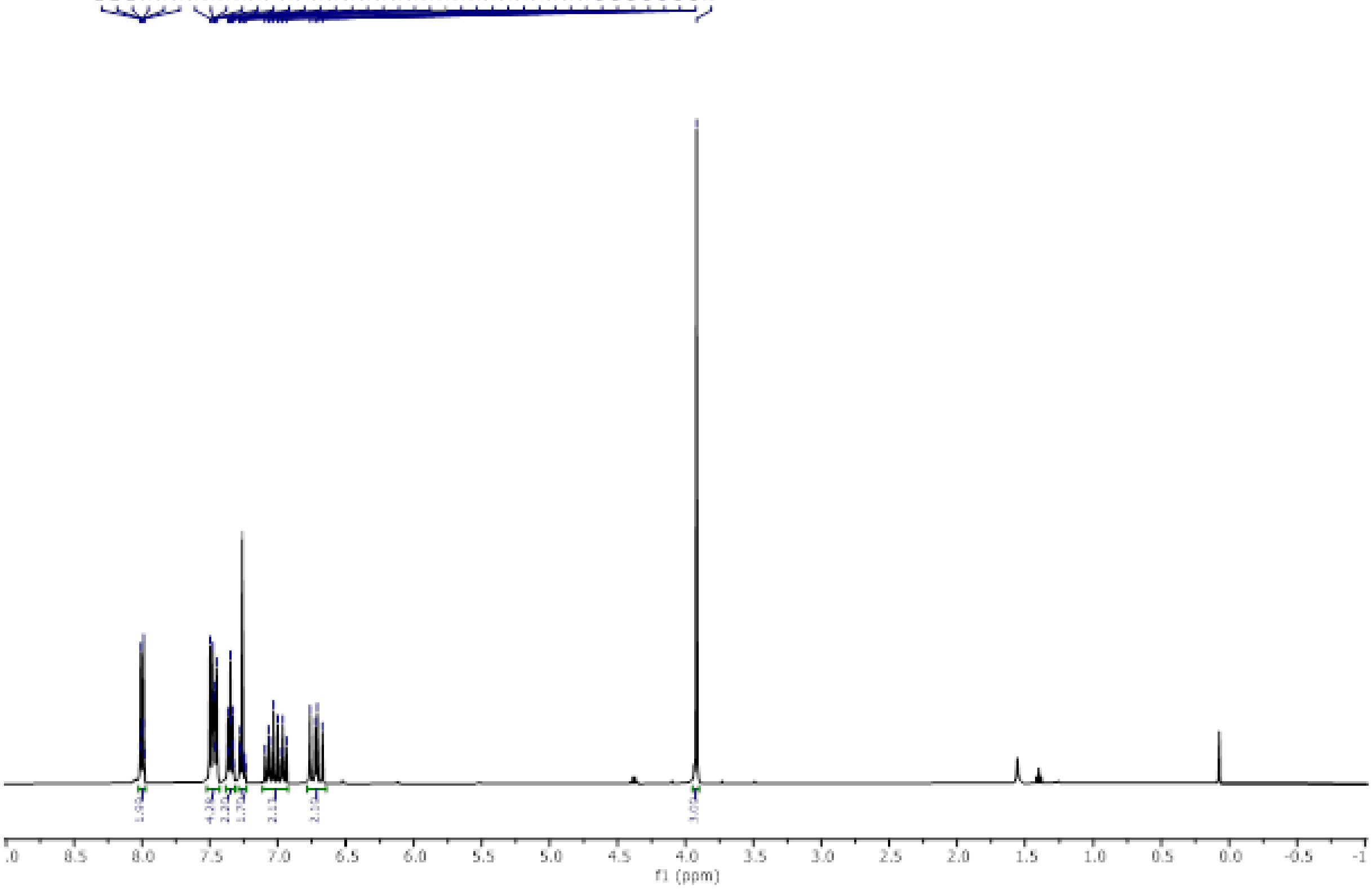

^{1}H NMR spectrum (400 MHz, $CDCl_3$) of **1u**.

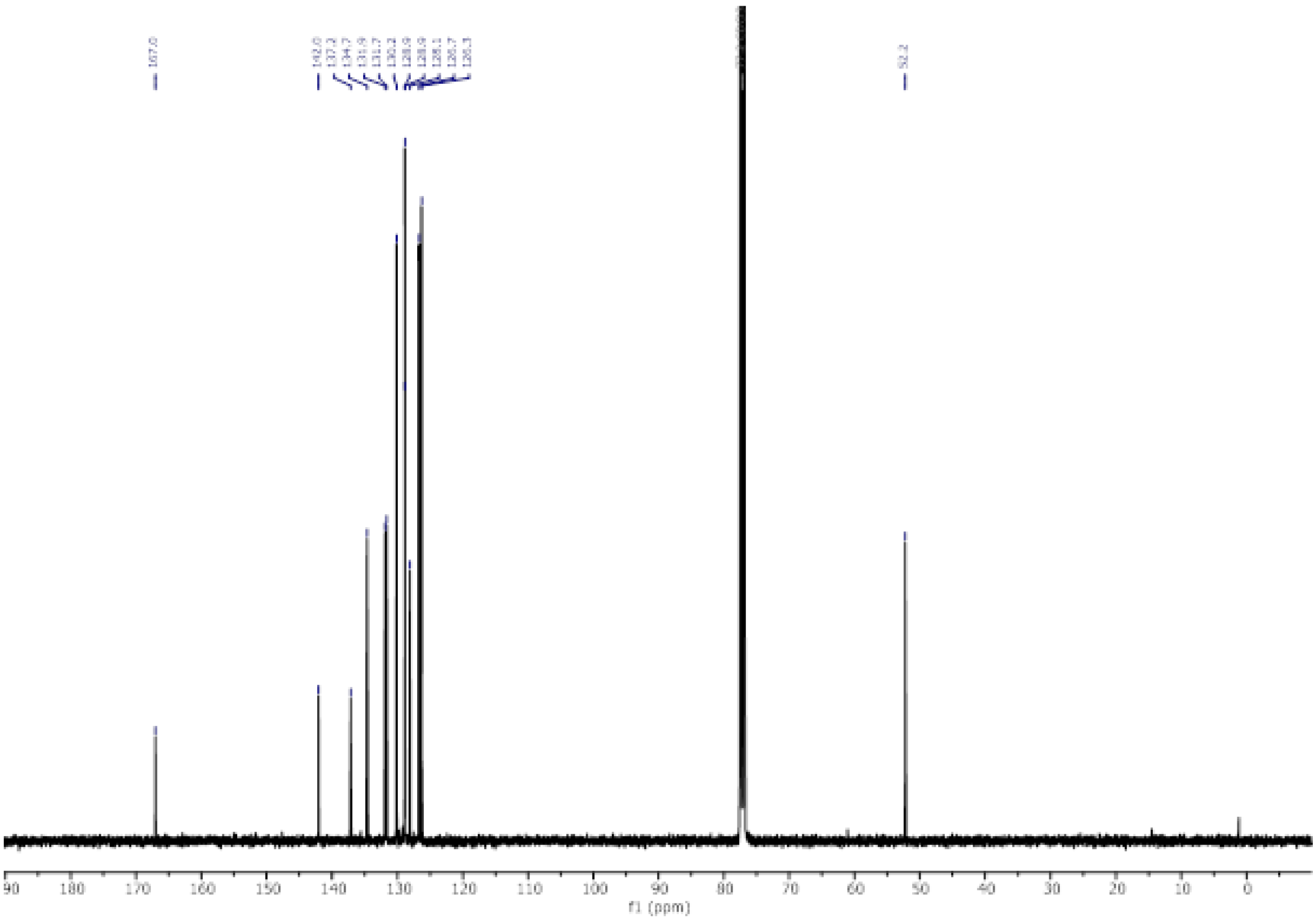

^{13}C NMR spectrum (101 MHz, $CDCl_3$) of **1u**.

4-[(1*E*,3*E*)-4-phenyl-1,3-butadien-1-yl]benzonitrile (**1v**)

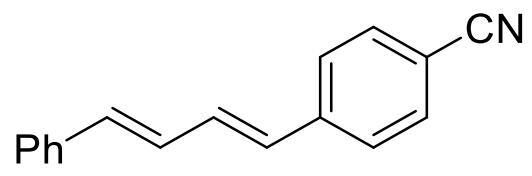

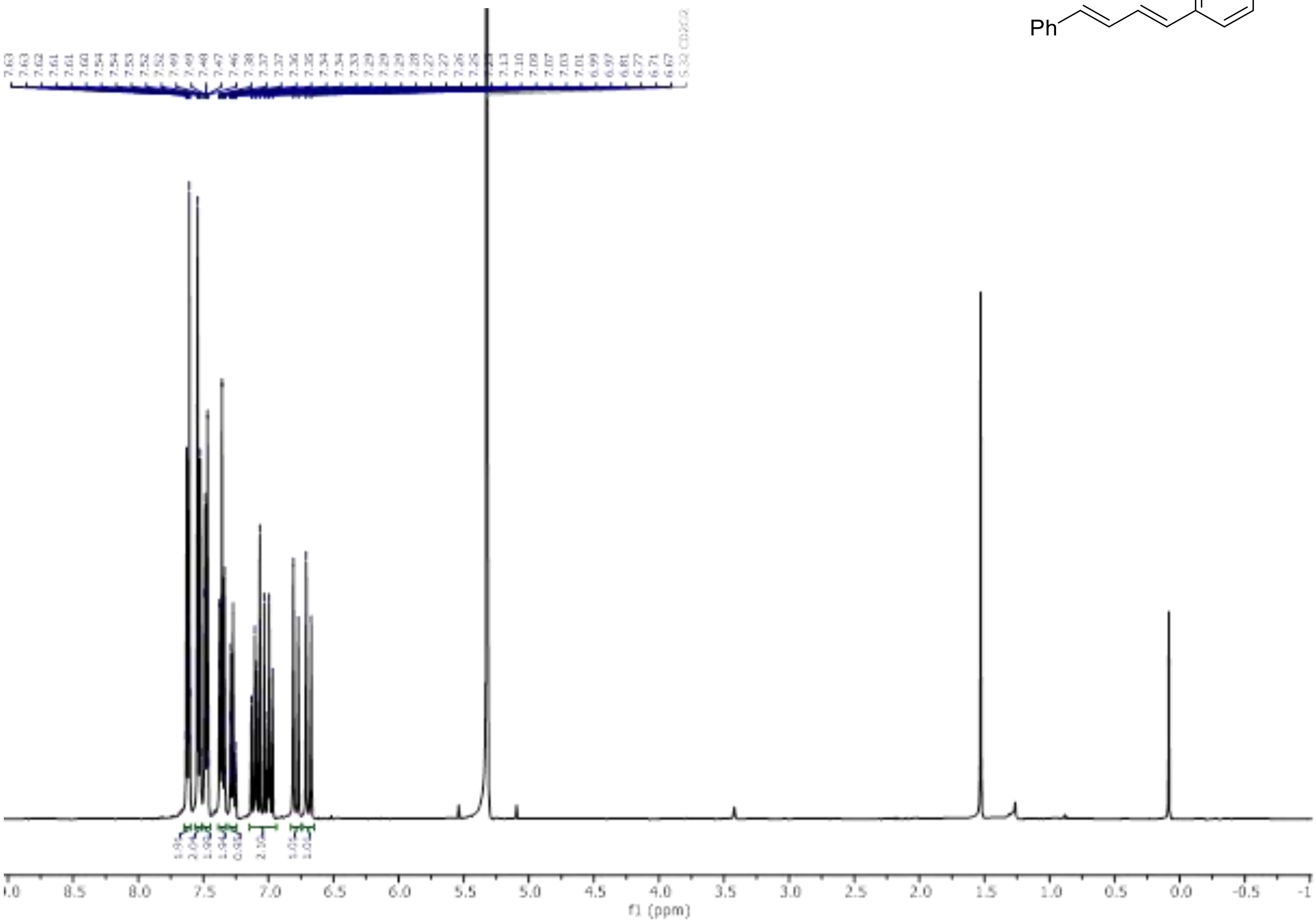

^{1}H NMR spectrum (400 MHz, CD_2Cl_2) of **1v**.

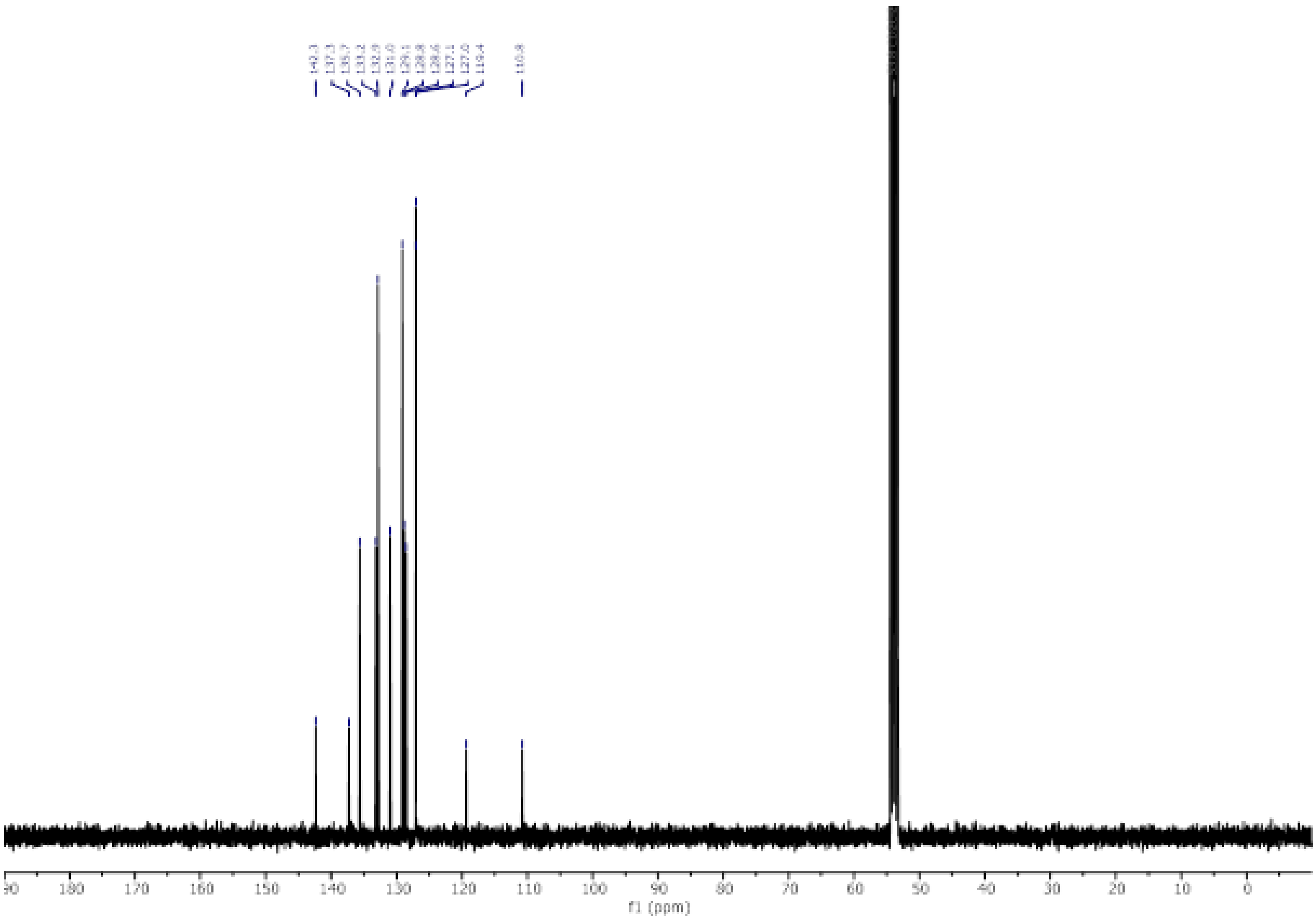

^{13}C NMR spectrum (101 MHz, CD_2Cl_2) of **1v**.

1-Nitro-4-[(1*E*,3*E*)-4-phenyl-1,3-butadien-1-yl]benzene (**1w**)

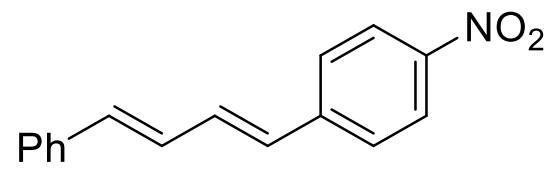

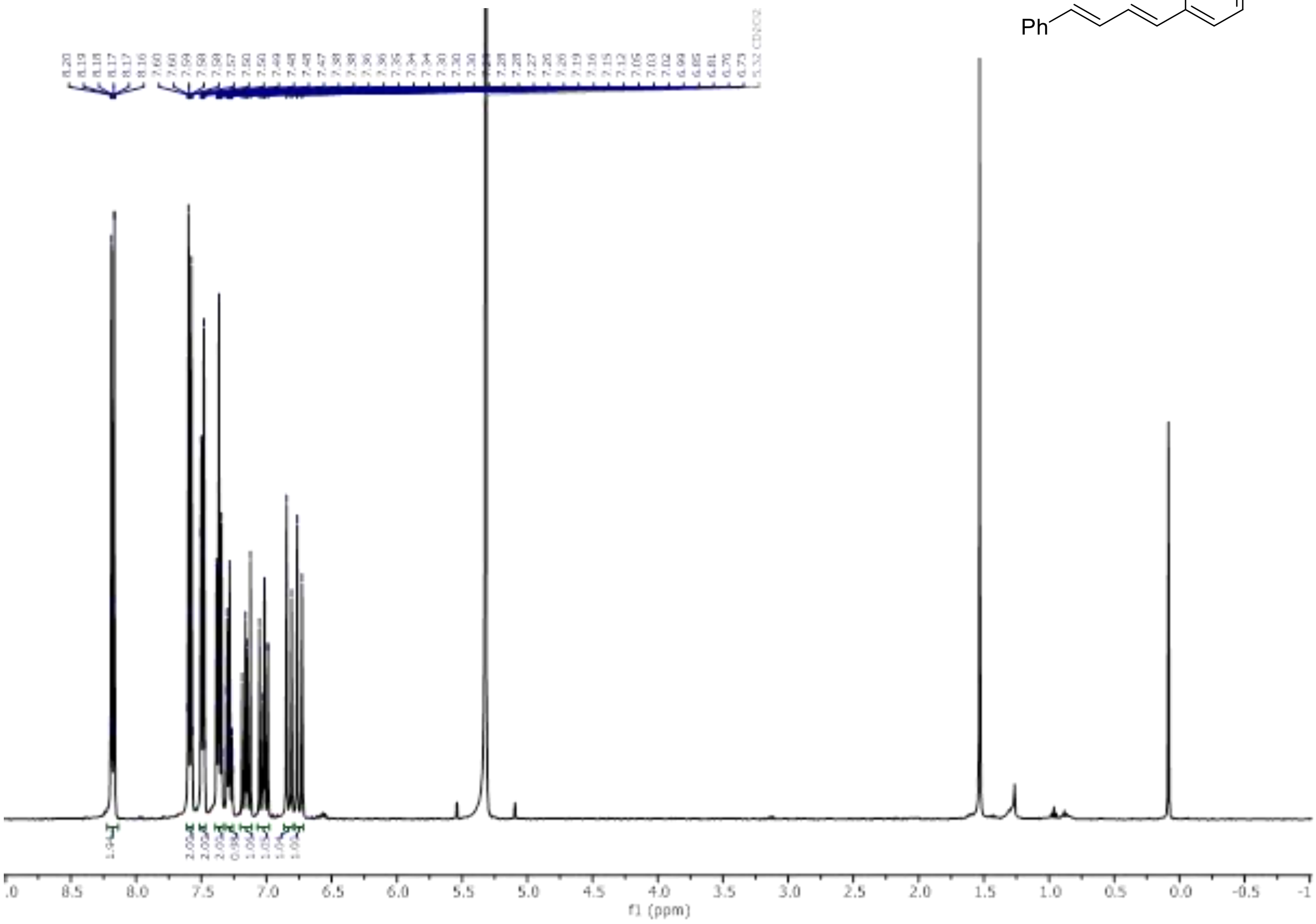

^{1}H NMR spectrum (400 MHz, CD_2Cl_2) of **1w**.

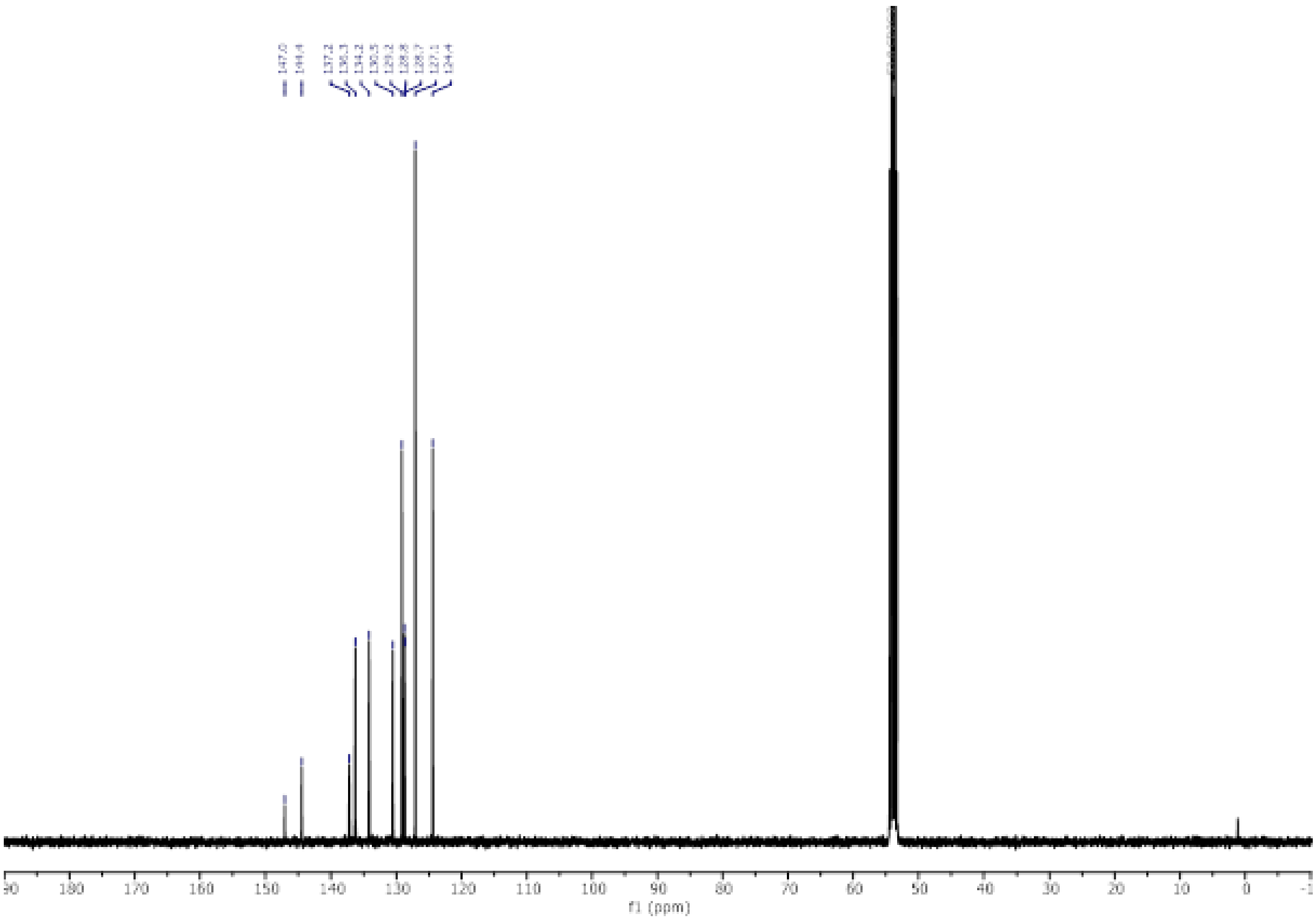

^{13}C NMR spectrum (101 MHz, CD_2Cl_2) of **1w**.

[1,3-Hexadien-1-yl]benzene

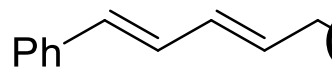

(1x)

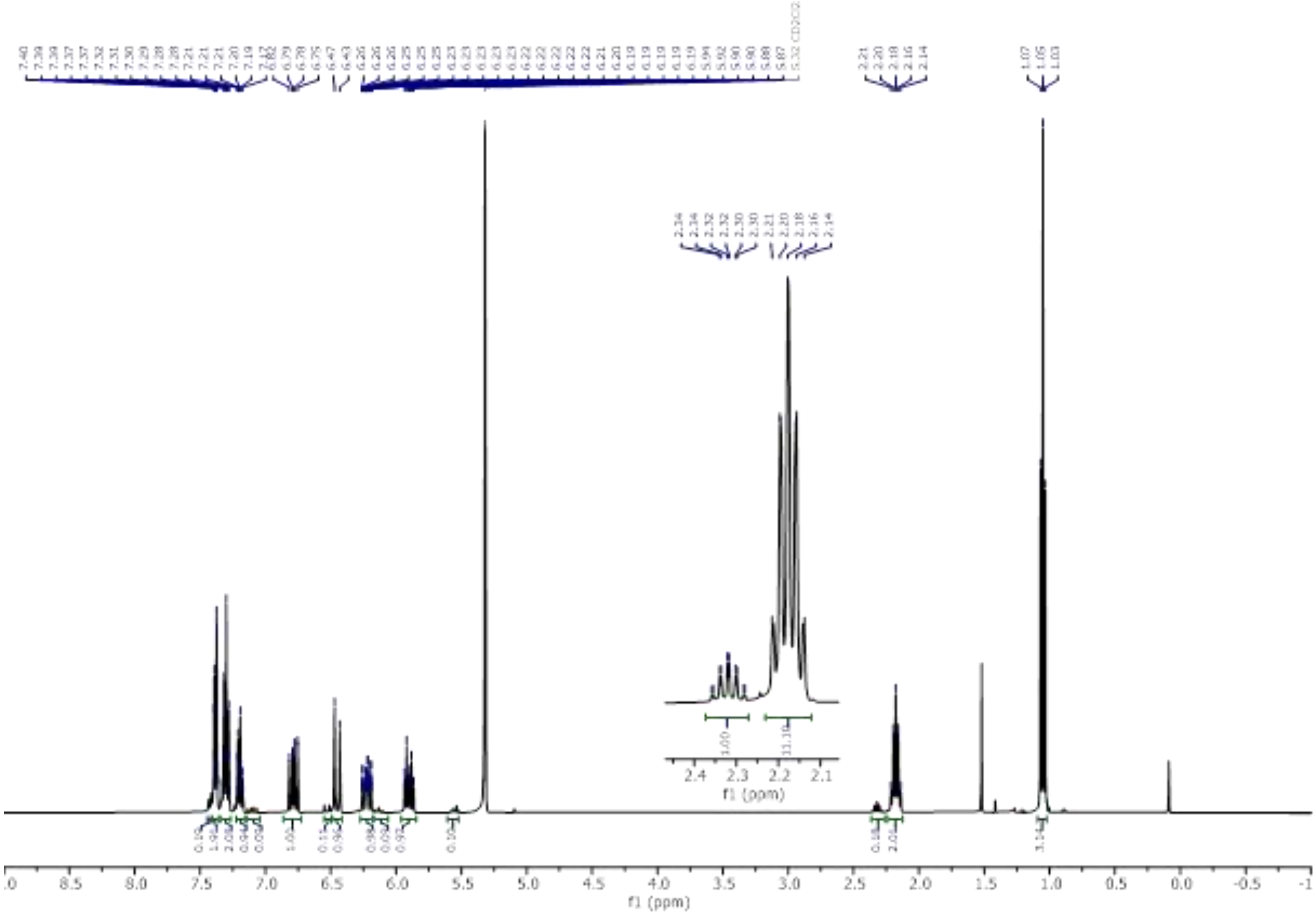

^{1}H NMR spectrum (400 MHz, CD_2Cl_2) of **1x**.

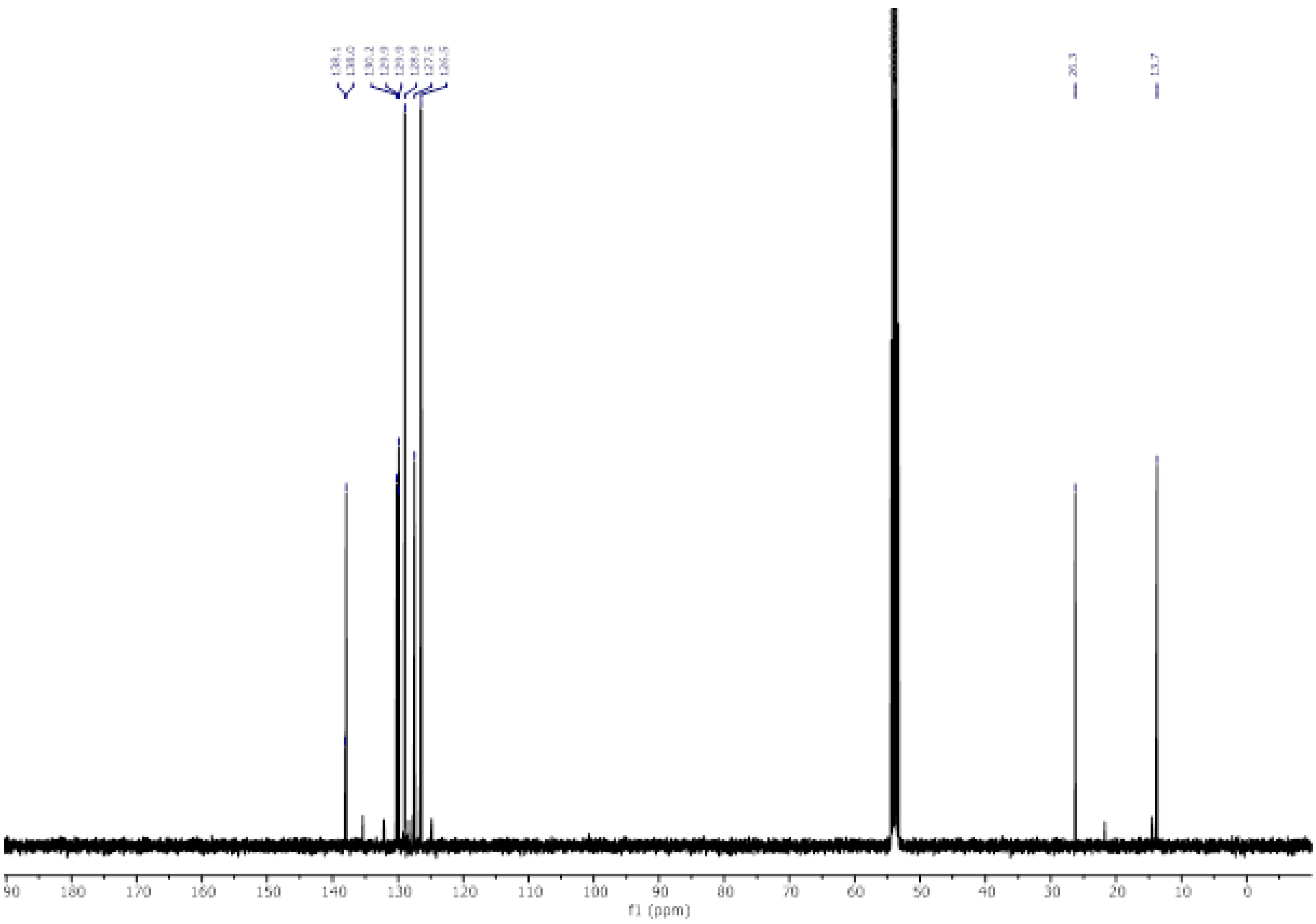

^{13}C NMR spectrum (101 MHz, CD_2Cl_2) of **1x**.

[(1*E*,3*E*)-4-Cyclohexyl-1,3-butadien-1-yl]benzene (**1y**)

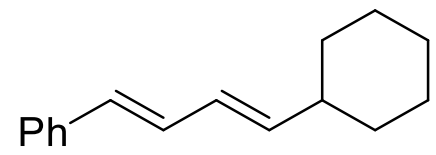

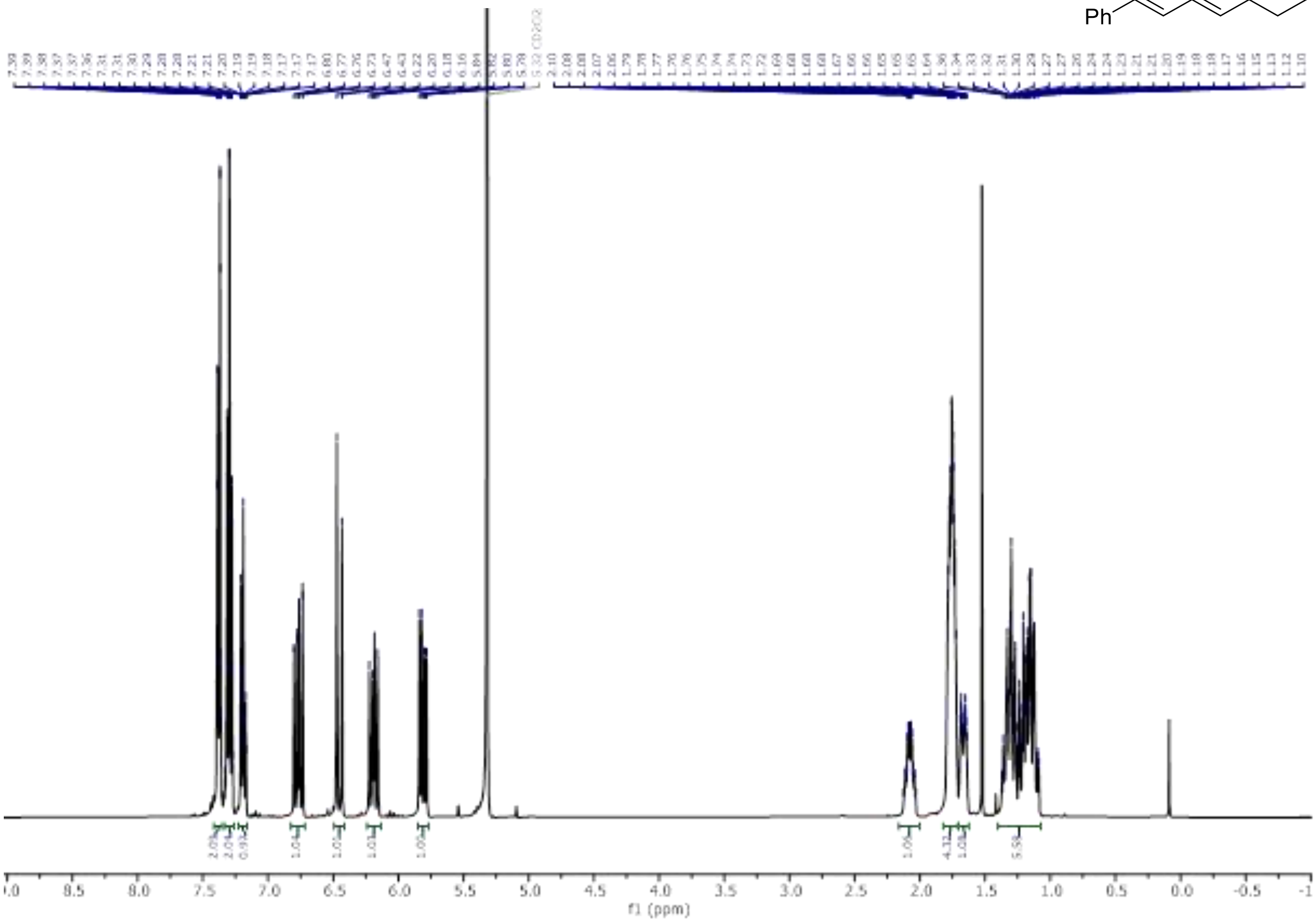

^{1}H NMR spectrum (400 MHz, CD_2Cl_2) of **1y**.

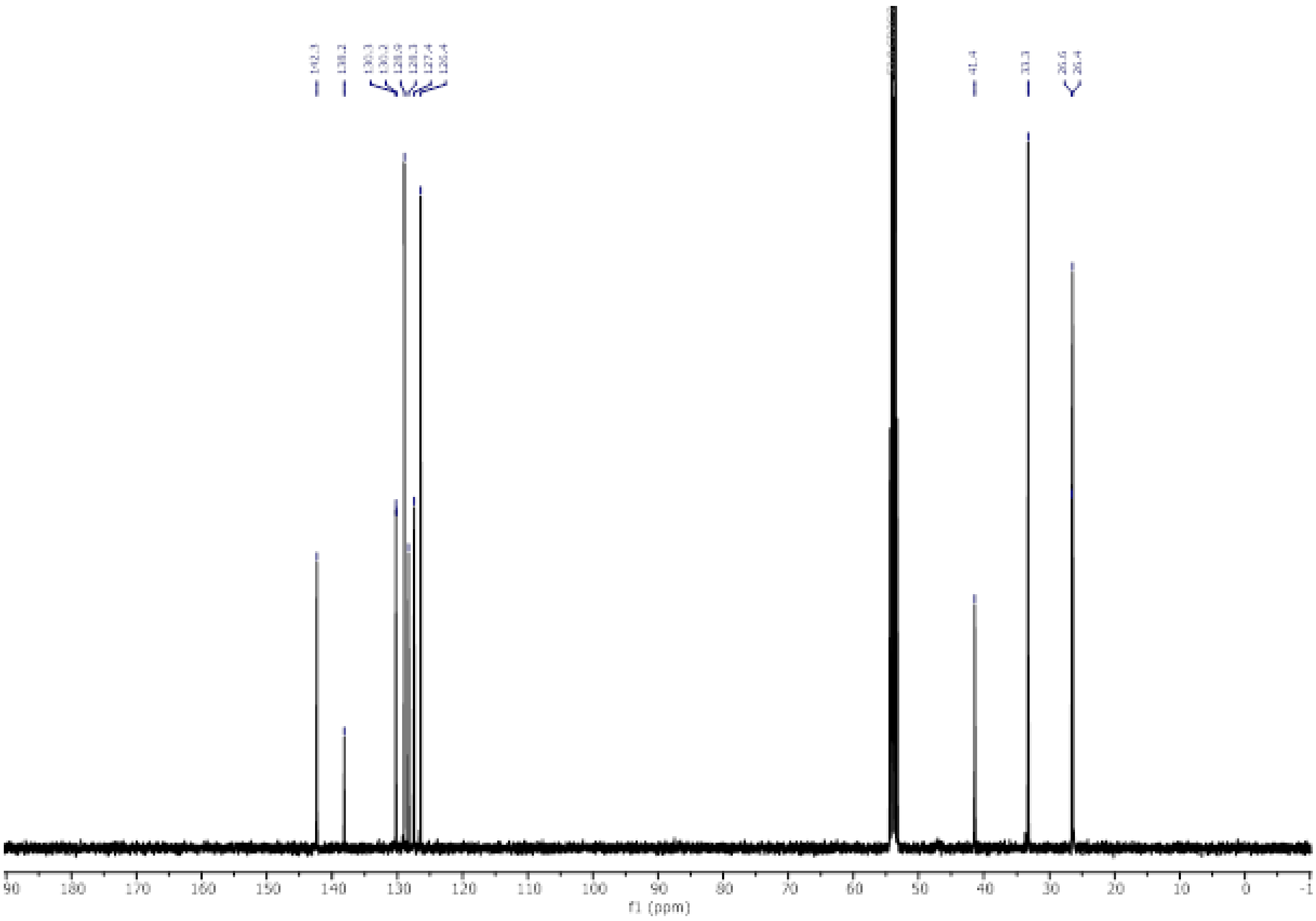

^{13}C NMR spectrum (101 MHz, CD_2Cl_2) of **1y**.

(1*E*,3*E*)-1,4-Di(4-methylphenyl)-1,3-butadien (**1z**)

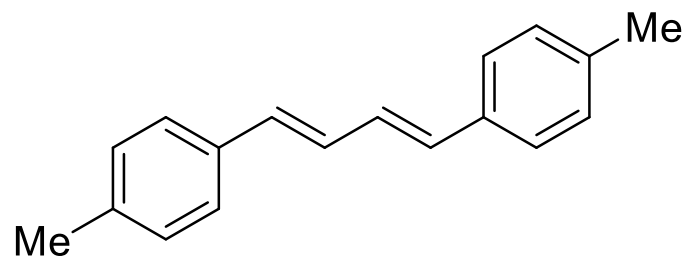

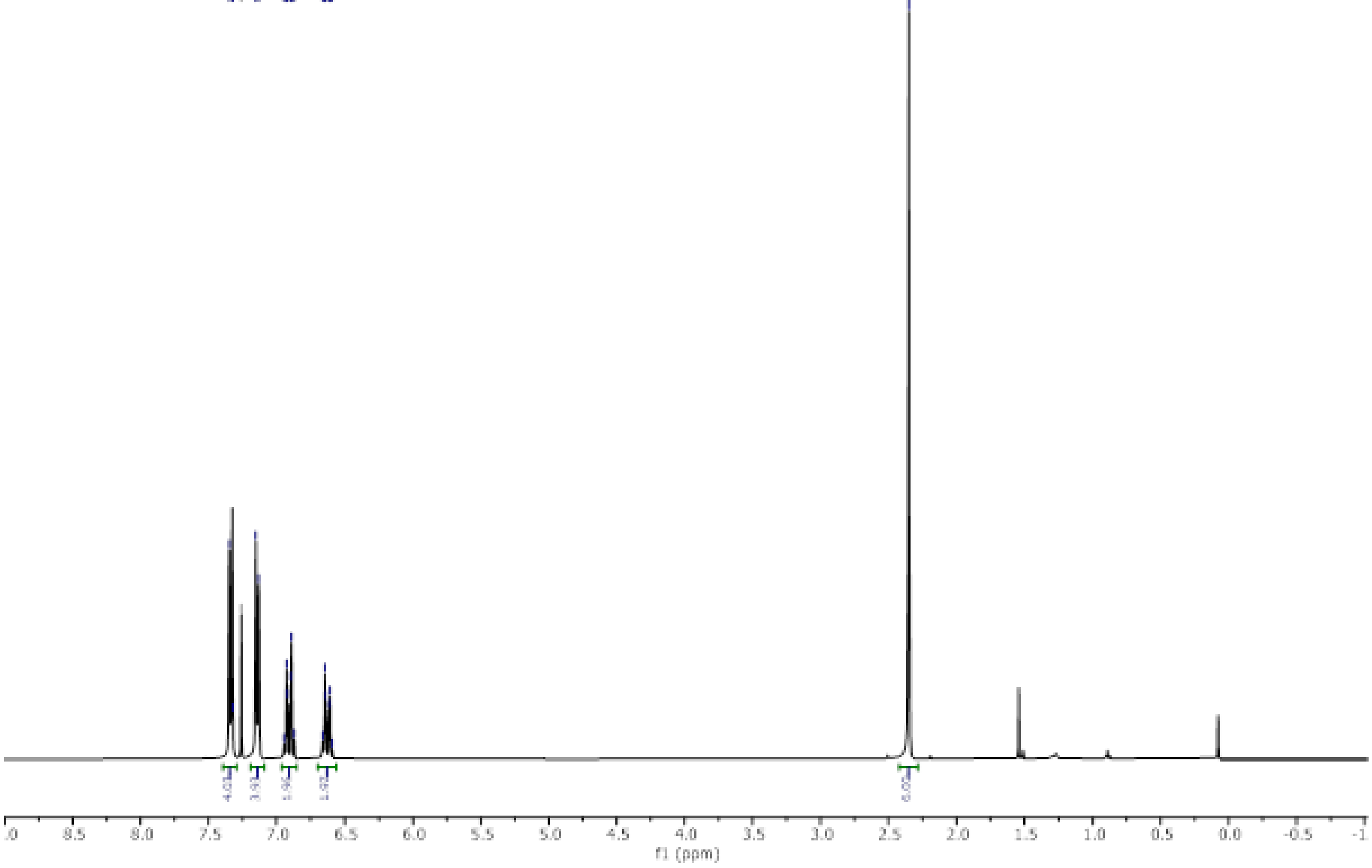

^{1}H NMR spectrum (400 MHz, $CDCl_3$) of **1z**.

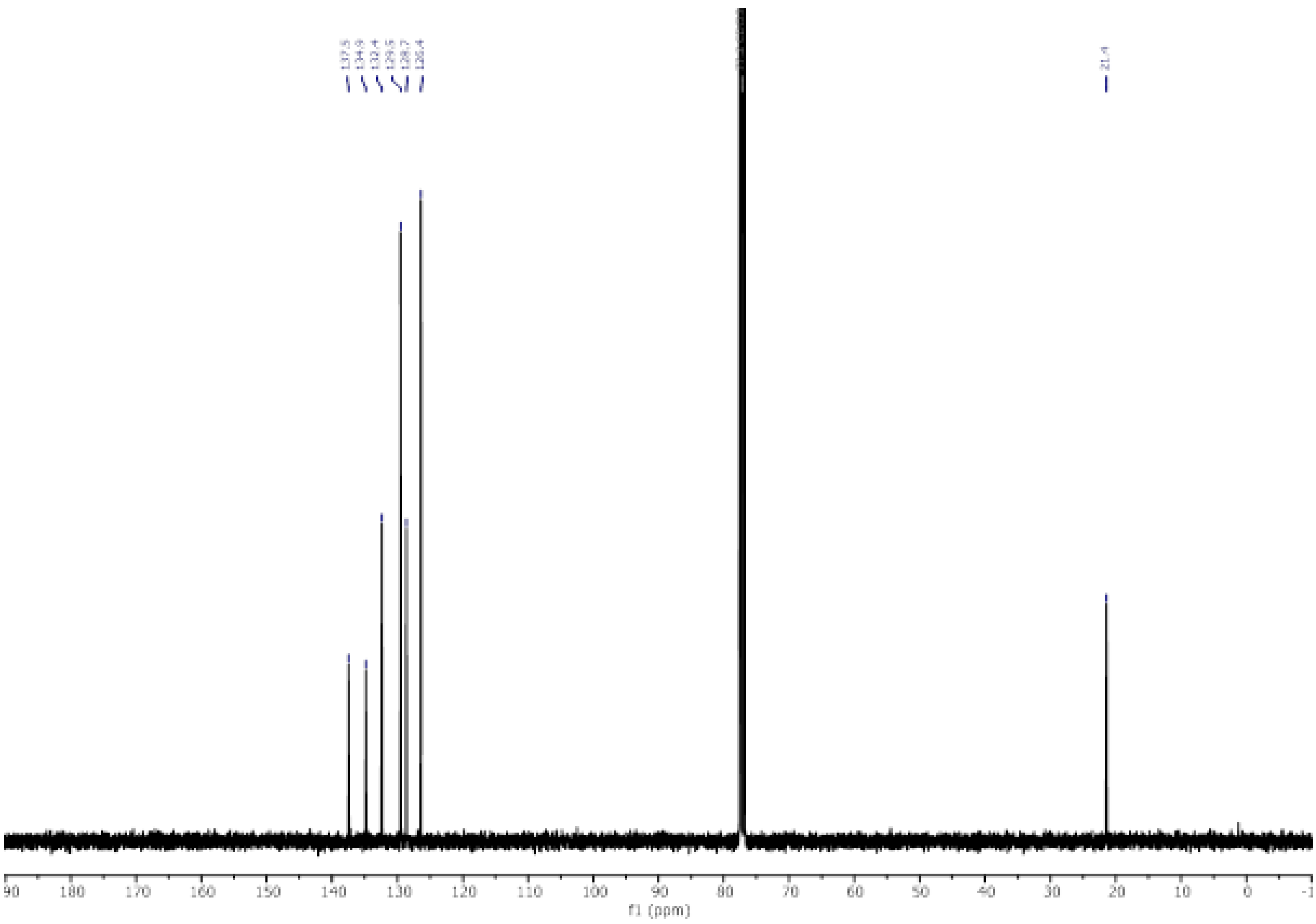

^{13}C NMR spectrum (101 MHz, $CDCl_3$) of **1z**.

(1*E*,3*E*)-1,4-Di(4-bromophenyl)-1,3-butadien (**1aa**)

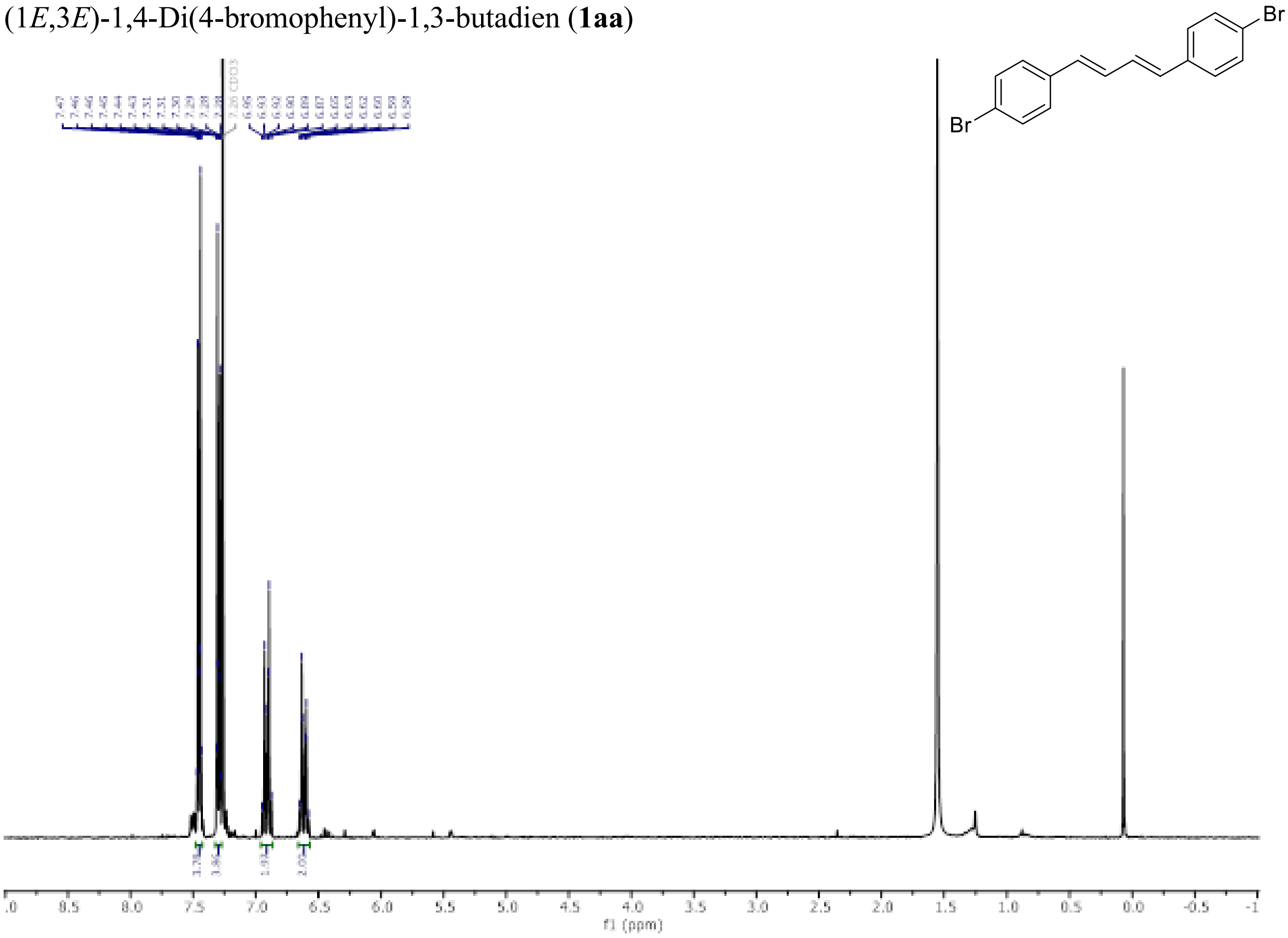

^{1}H NMR spectrum (400 MHz, $CDCl_3$) of **1aa**.

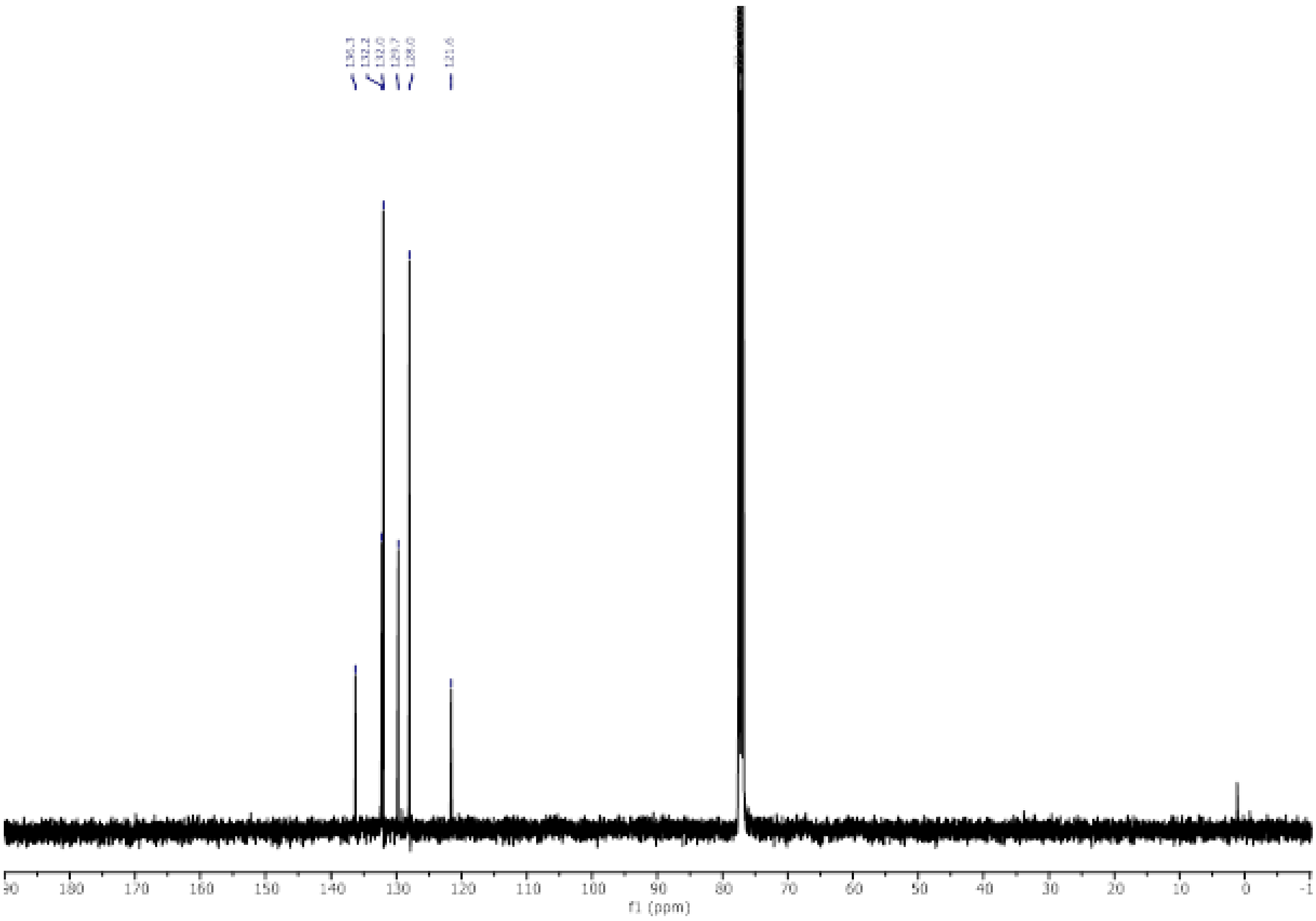

^{13}C NMR spectrum (101 MHz, $CDCl_3$) of **1aa**.

Ethyl sorbate (**1ab**)

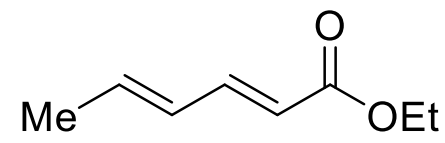

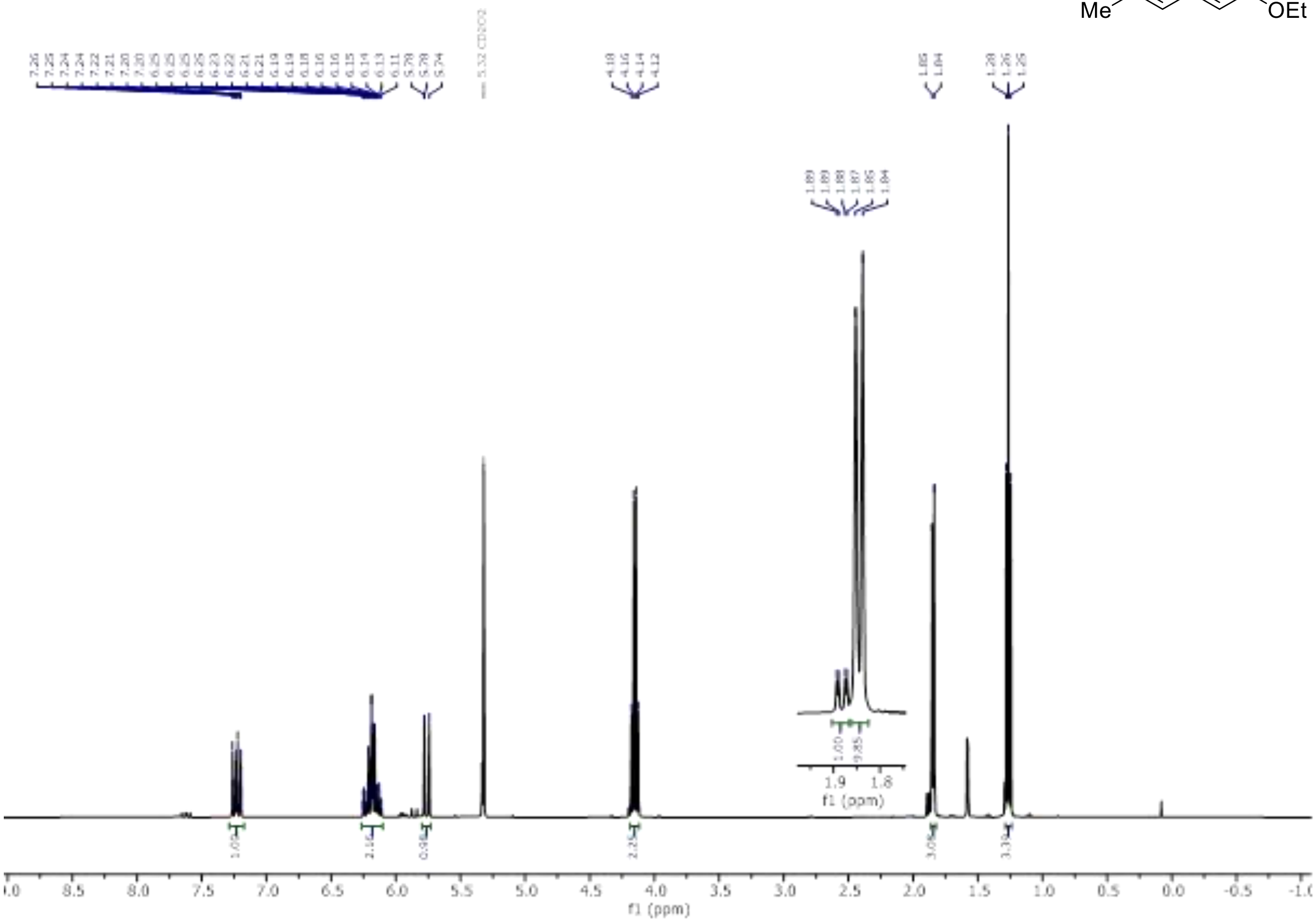

^{1}H NMR spectrum (400 MHz, CD_2Cl_2) of **1ab**.

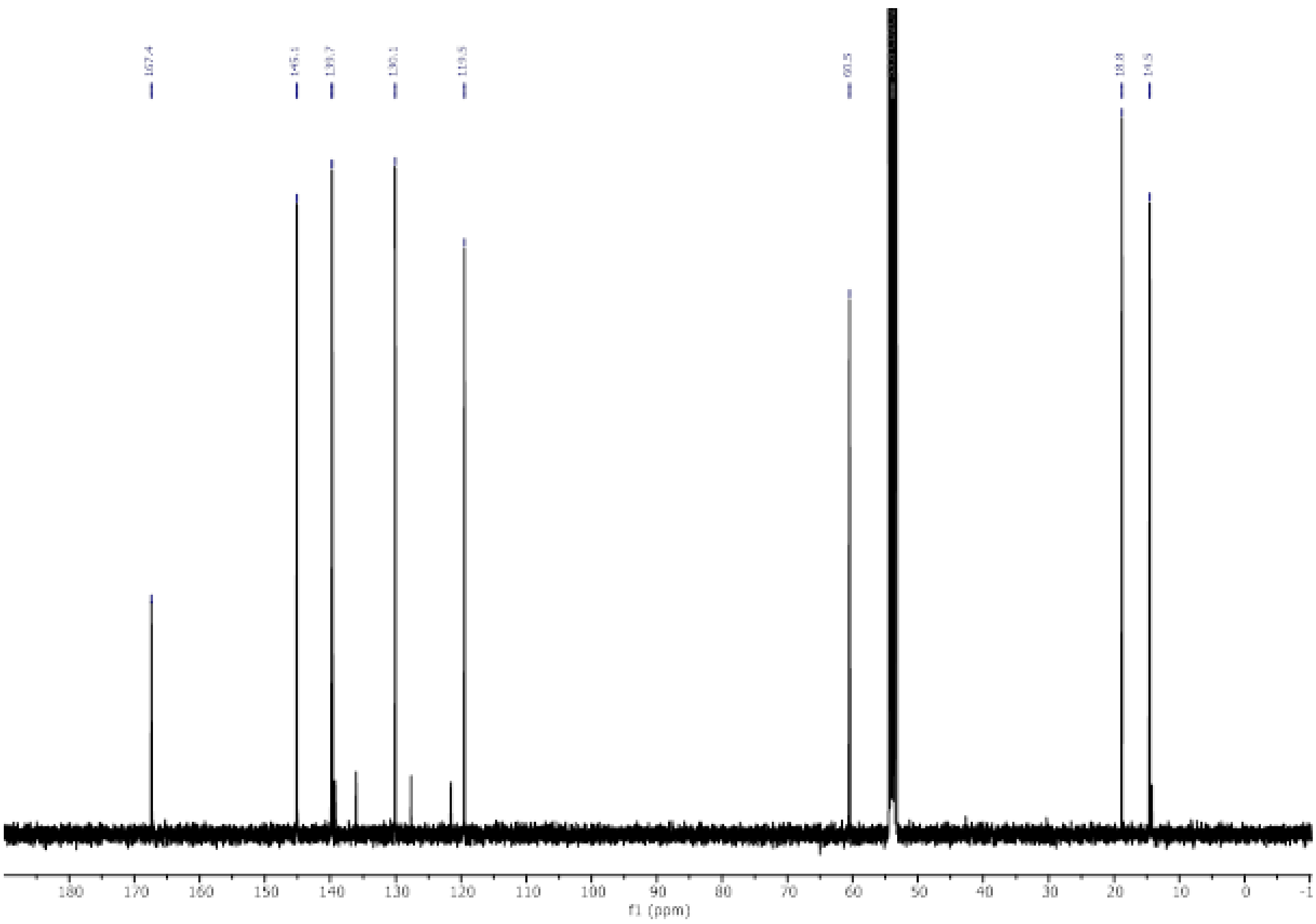

^{13}C NMR spectrum (101 MHz, CD_2Cl_2) of **1ab**.

Ethyl (2*E*,4*E*)-2,4-octadienoate (**1ac**)

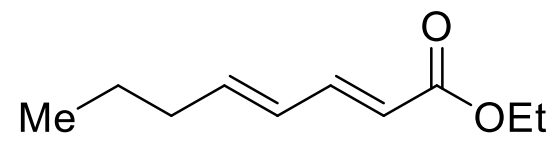

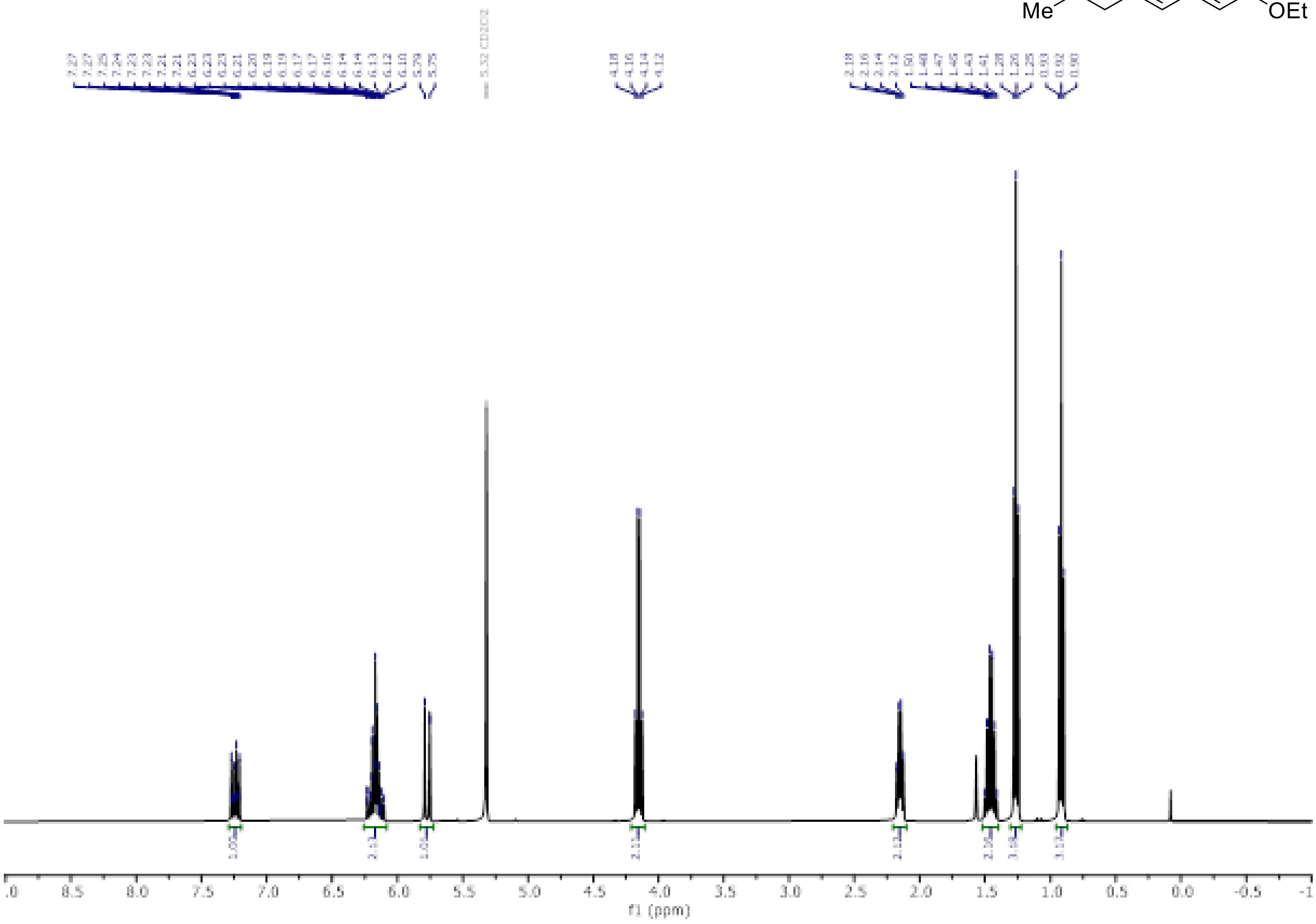

^{1}H NMR spectrum (400 MHz, CD_2Cl_2) of **1ac**.

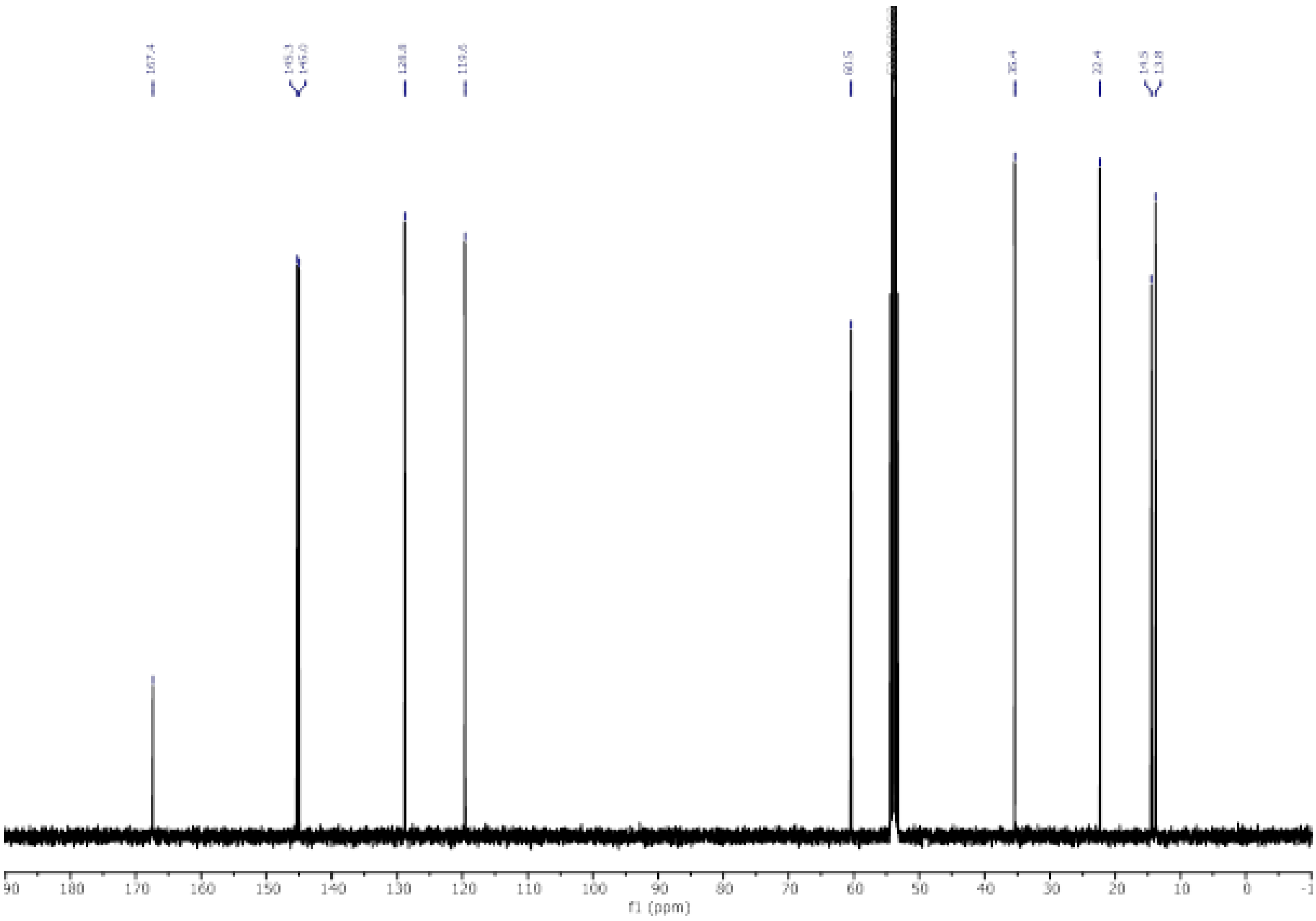

^{13}C NMR spectrum (101 MHz, CD_2Cl_2) of **1ac**.

Ethyl (2*E*,4*E*)-5-phenyl-2,4-pentadienoate (**1ad**)

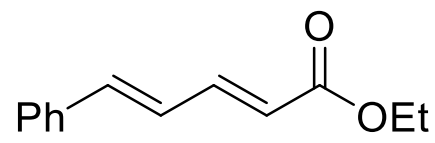

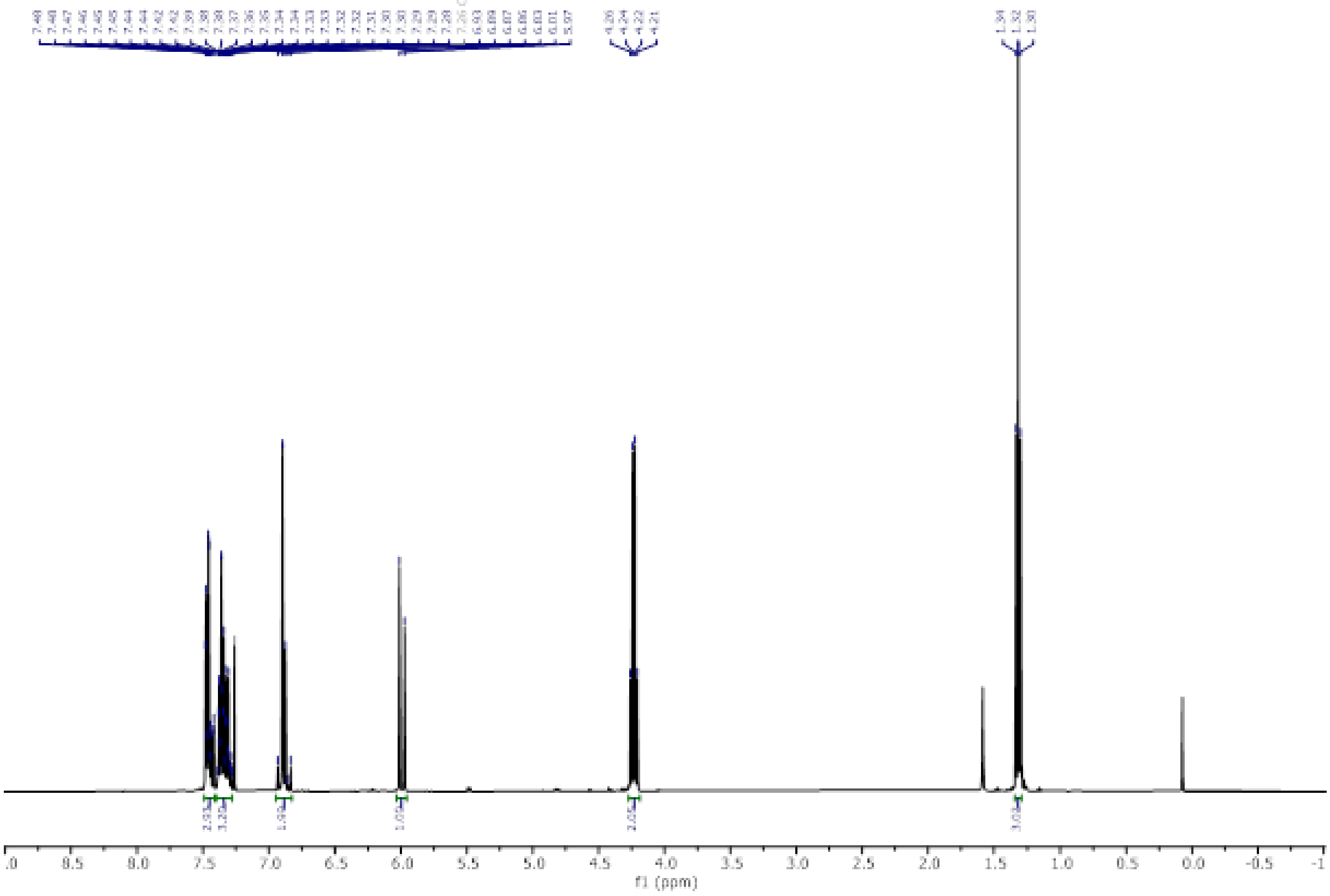

^{1}H NMR spectrum (400 MHz, CDCl3) of **1ad**.

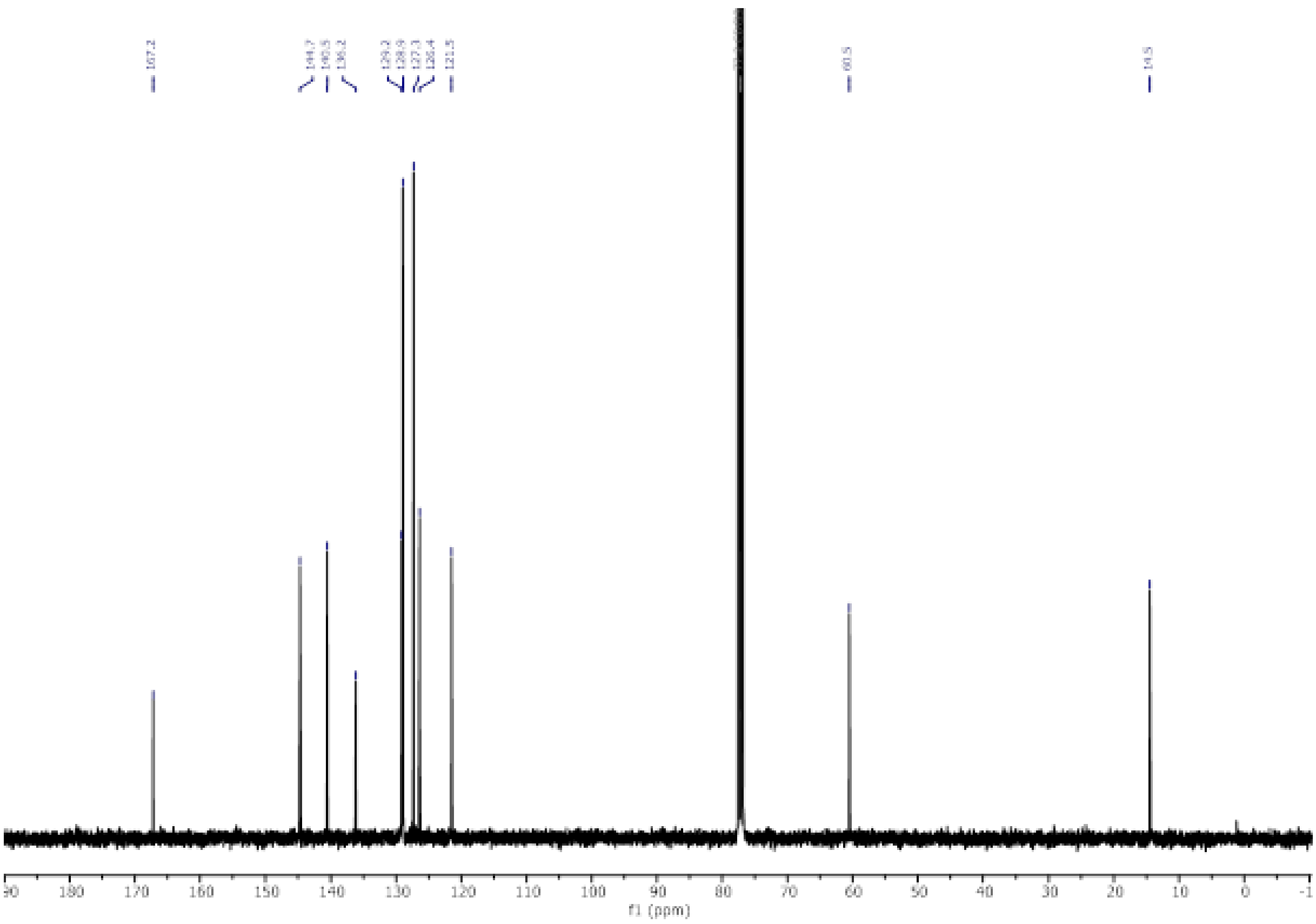

^{13}C NMR spectrum (101 MHz, CDCl3) of **1ad**.

1,1’-Biphenyl-4-(1*E*)-1,3-butadien-1-yl (**1ae**)

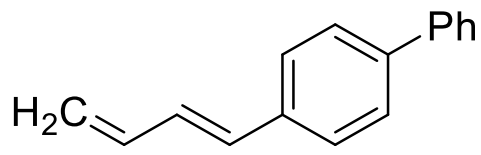

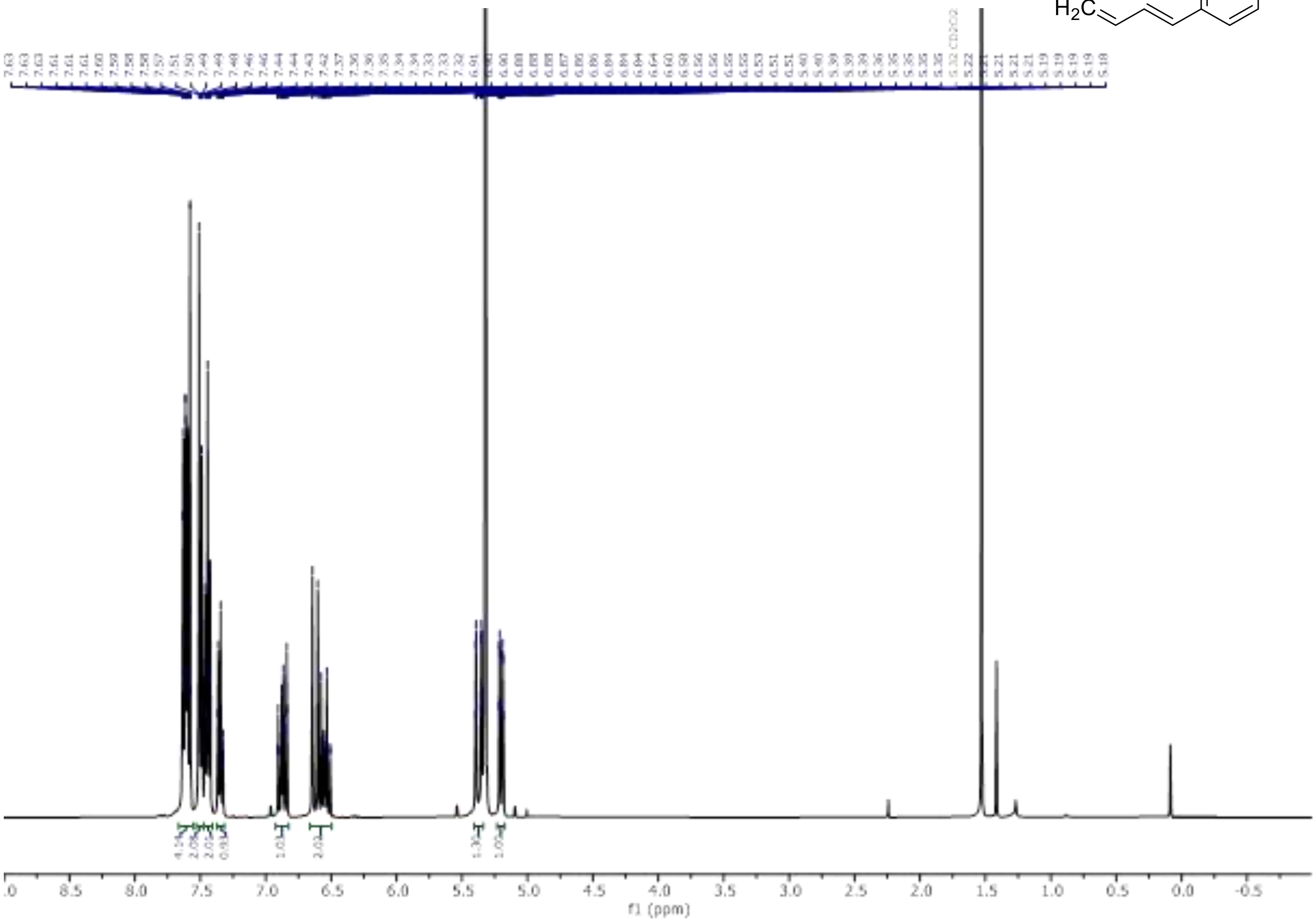

^{1}H NMR spectrum (400 MHz, CD_2Cl_2) of **1ae**.

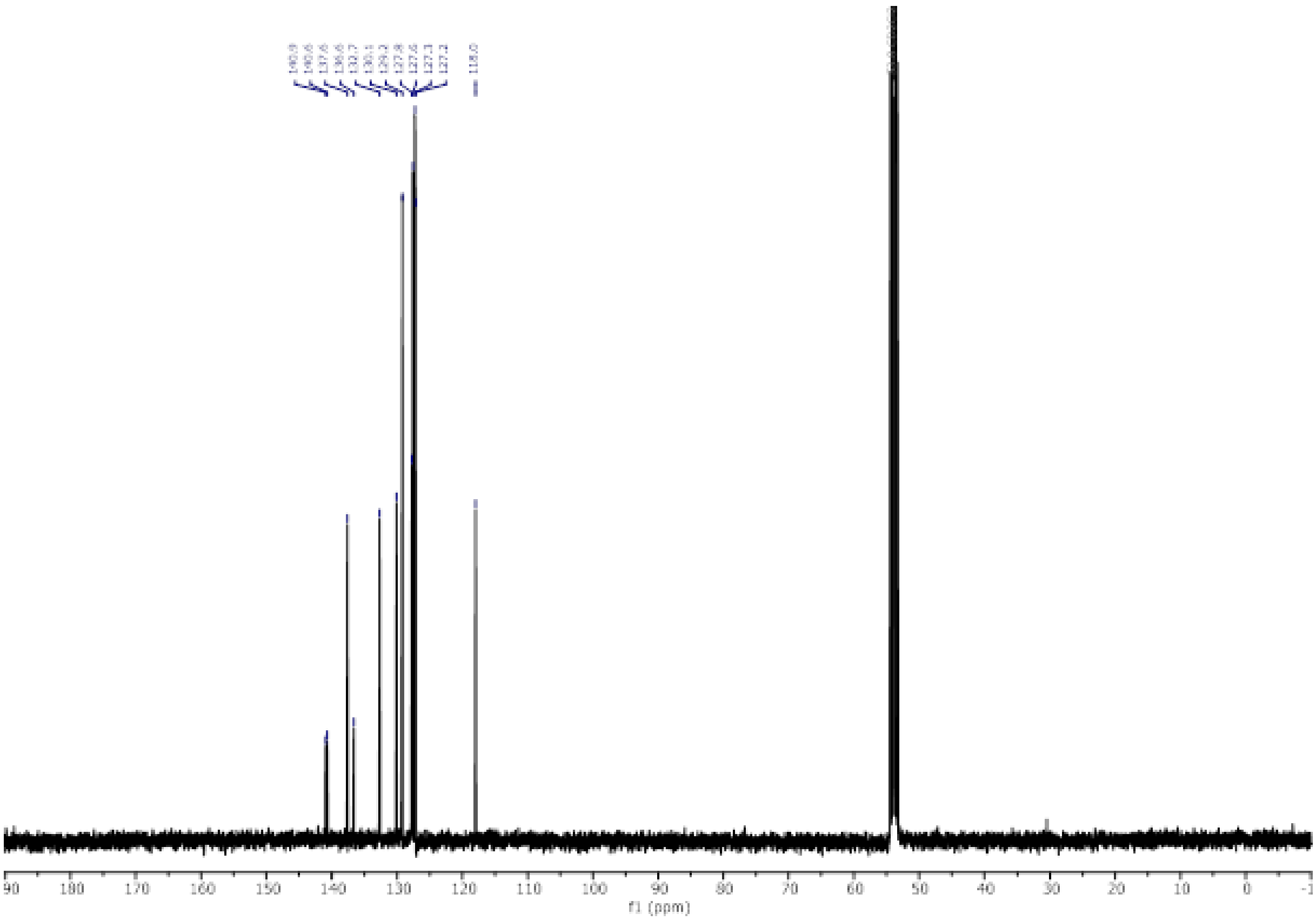

^{13}C NMR spectrum (101 MHz, CD_2Cl_2) of **1ae**.

1-(1*E*)-1,3-Butadien-1-yl-4-methylbenzene (**1af**)

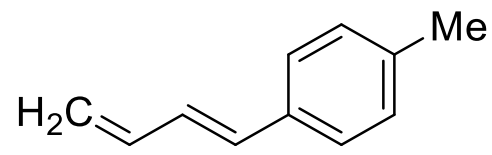

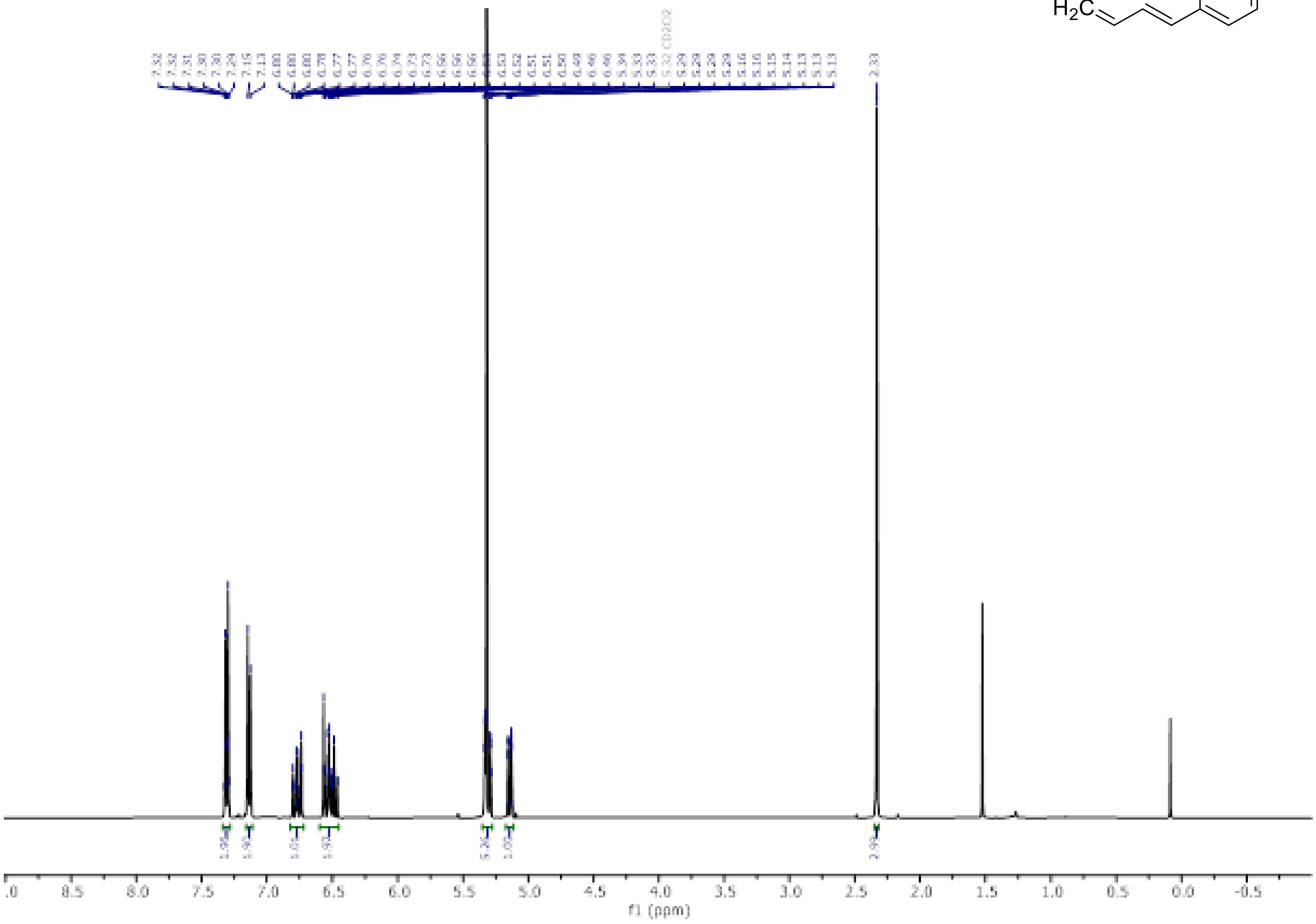

^{1}H NMR spectrum (400 MHz, CD_2Cl_2) of **1af**.

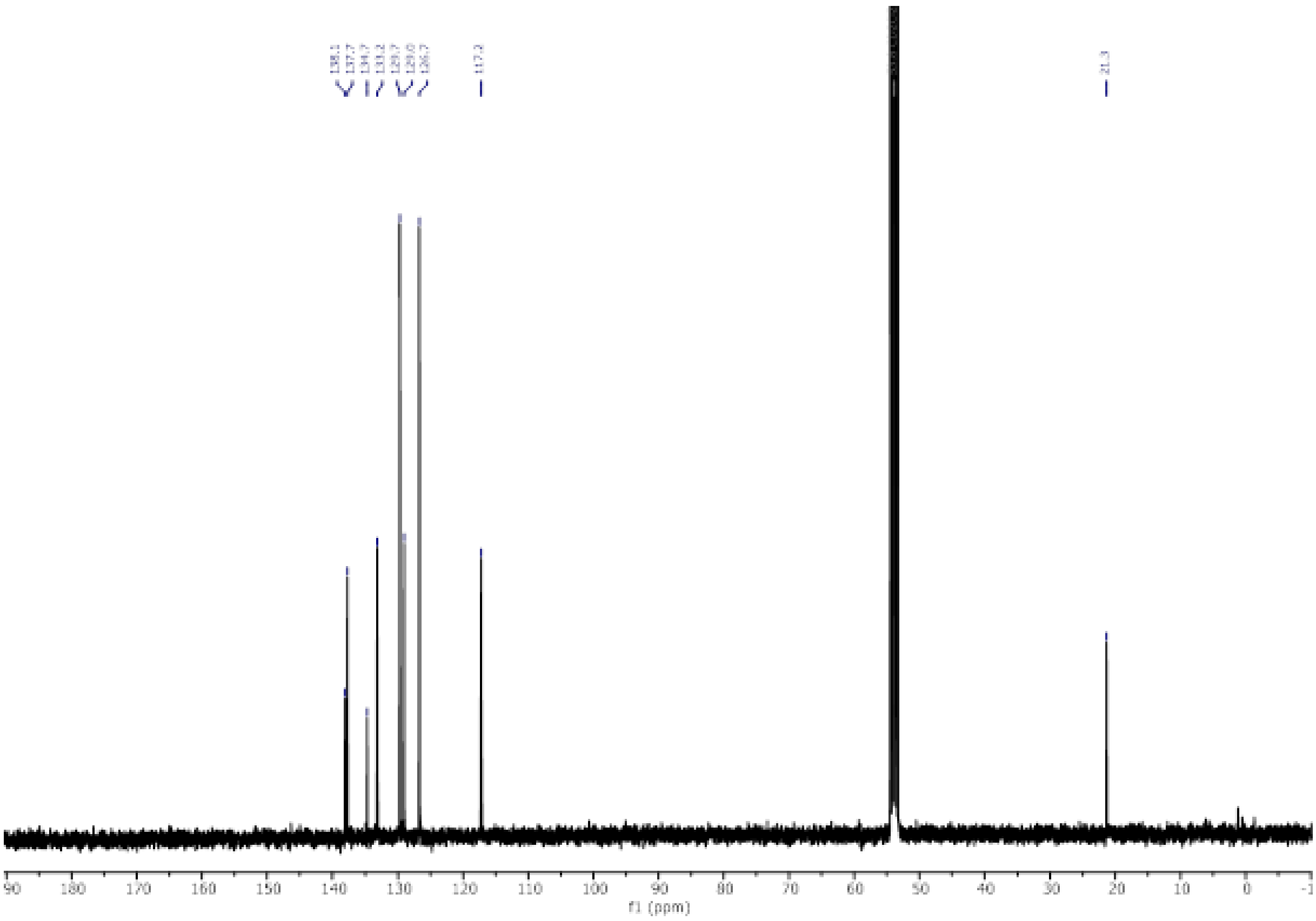

^{13}C NMR spectrum (101 MHz, CD_2Cl_2) of **1af**.

2-Cyclopropyl-1,4-benzoquinone (**2d**)

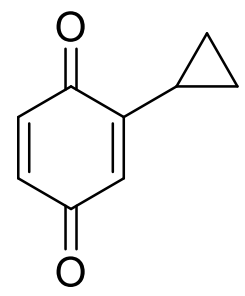

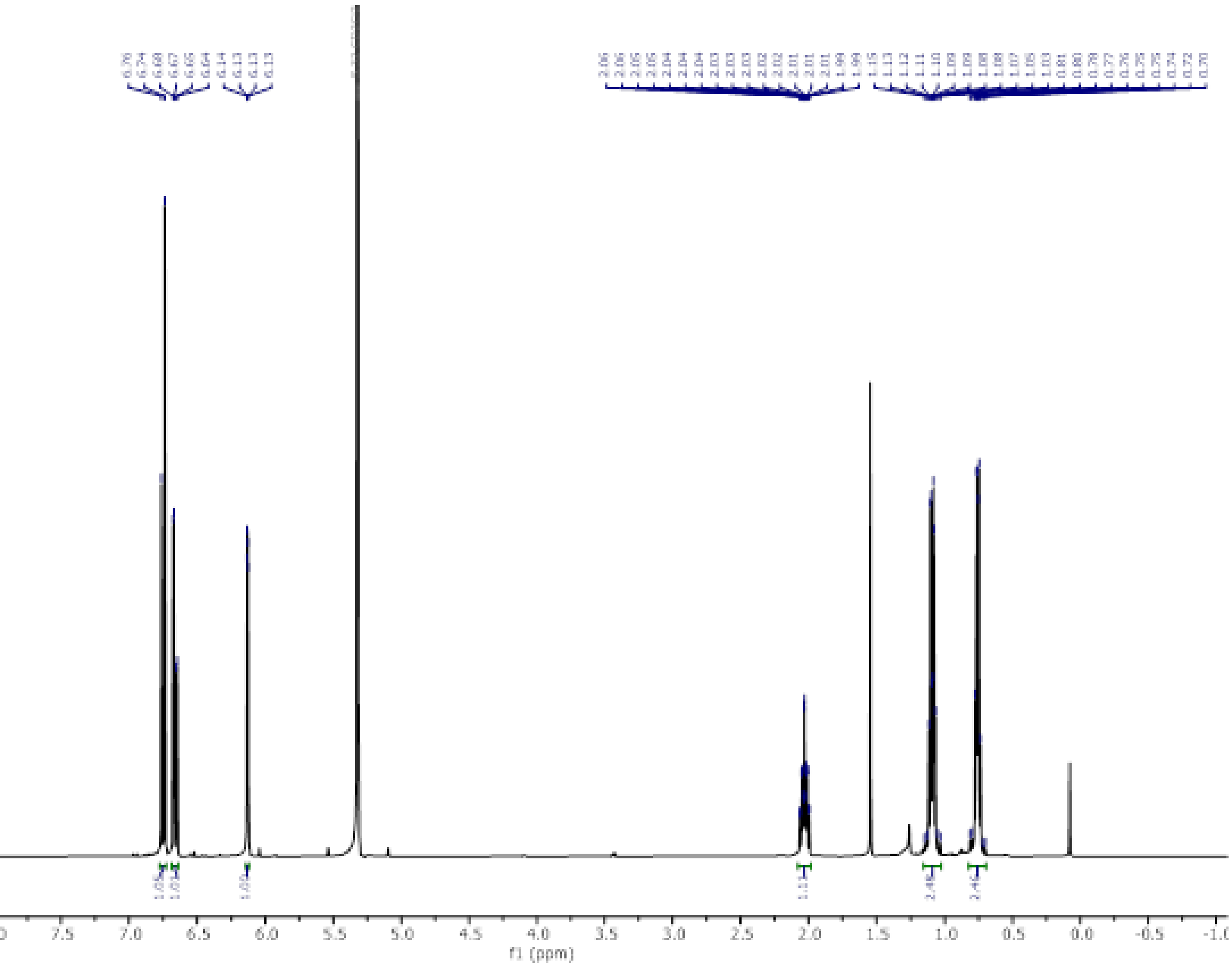

^{1}H NMR spectrum (400 MHz, CD_2Cl_2) of **2d**.

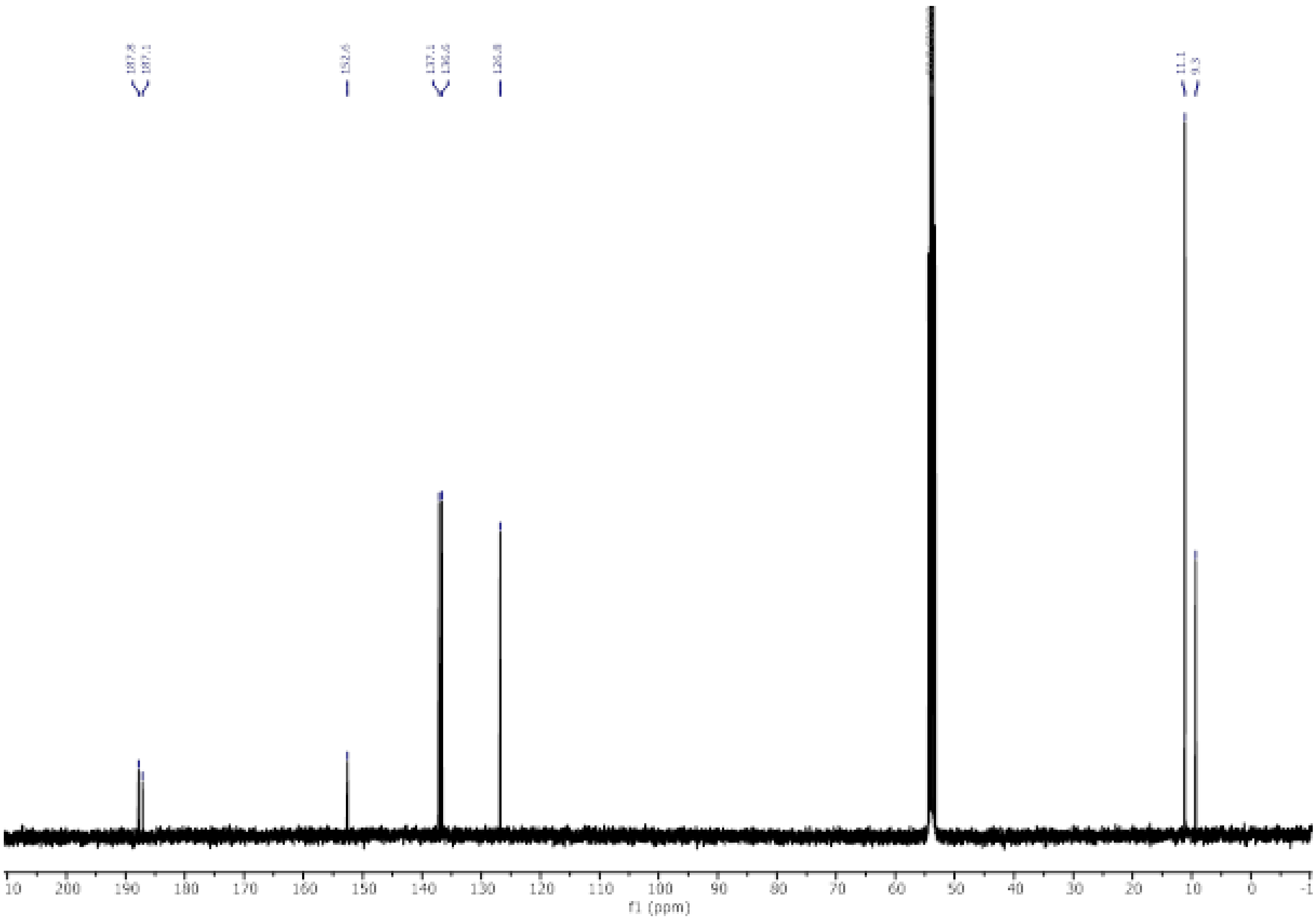

^{13}C NMR spectrum (101 MHz, CD_2Cl_2) of **2d**.

2-Cyclohexyl-1,4-benzoquinone (**2e**)

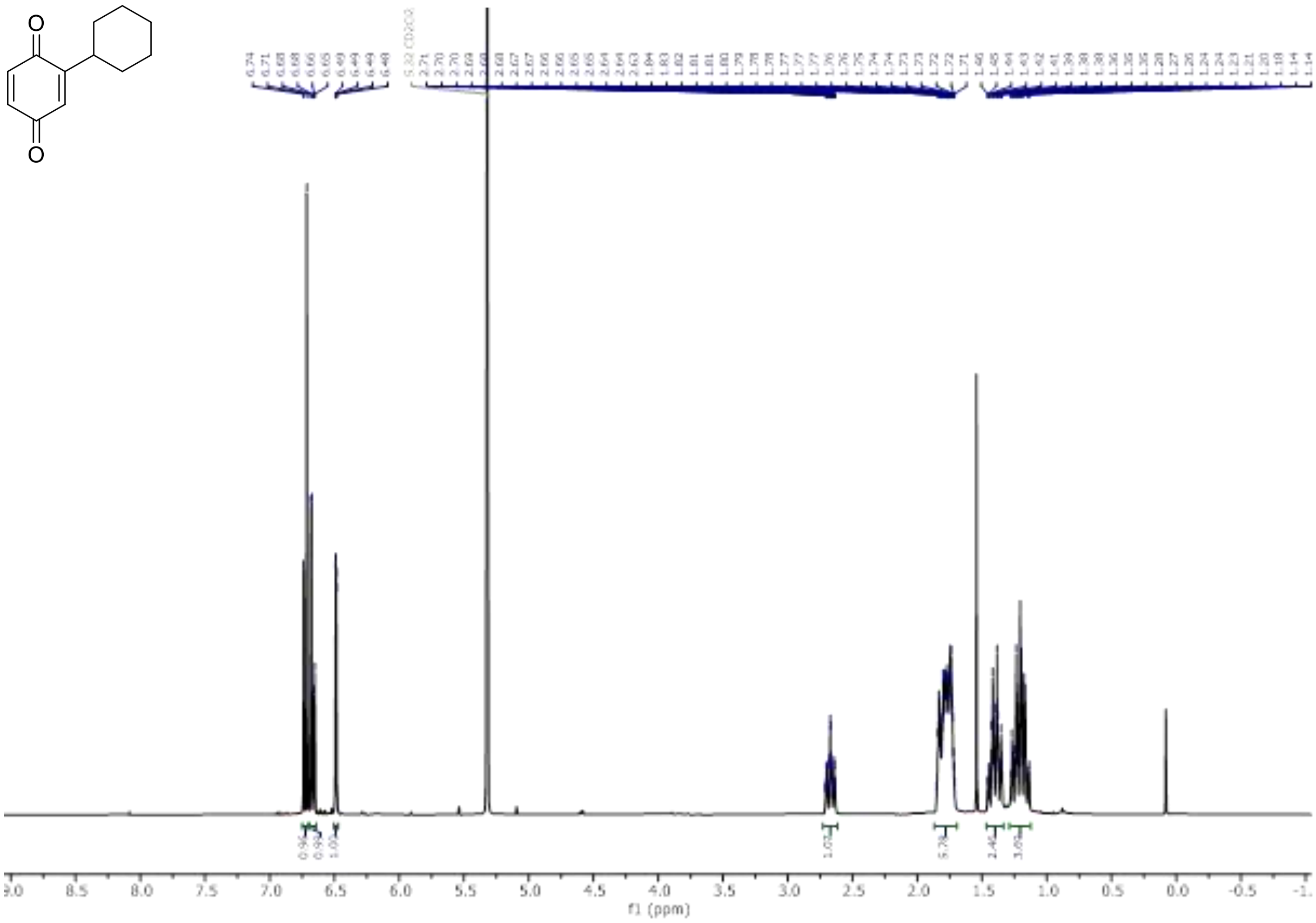

^{1}H NMR spectrum (400 MHz, CD_2Cl_2) of **2e**.

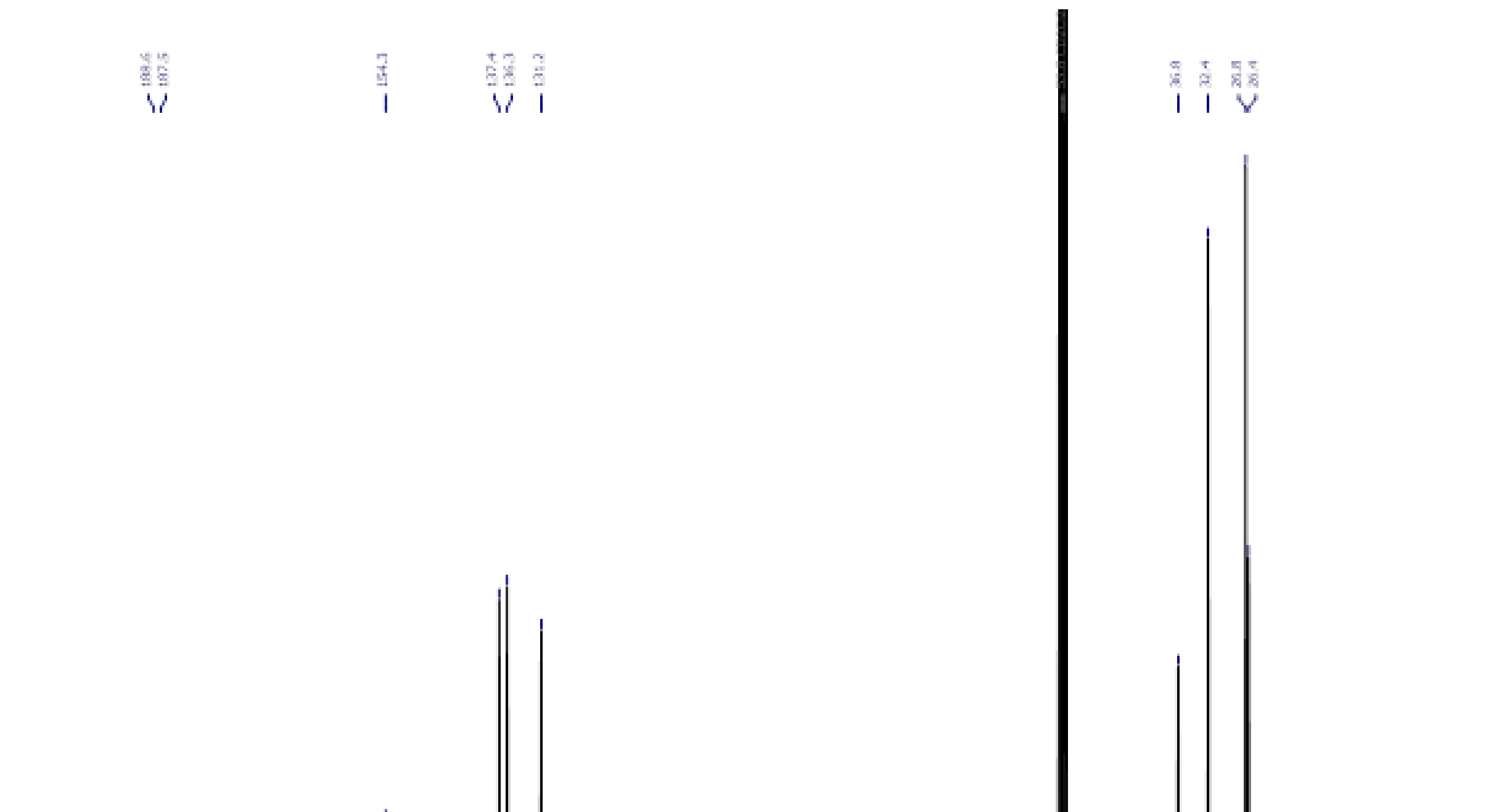

^{13}C NMR spectrum (101 MHz, CD_2Cl_2) of **2e**.

2-Phenyl-1,4-benzoquinone (**2f**)

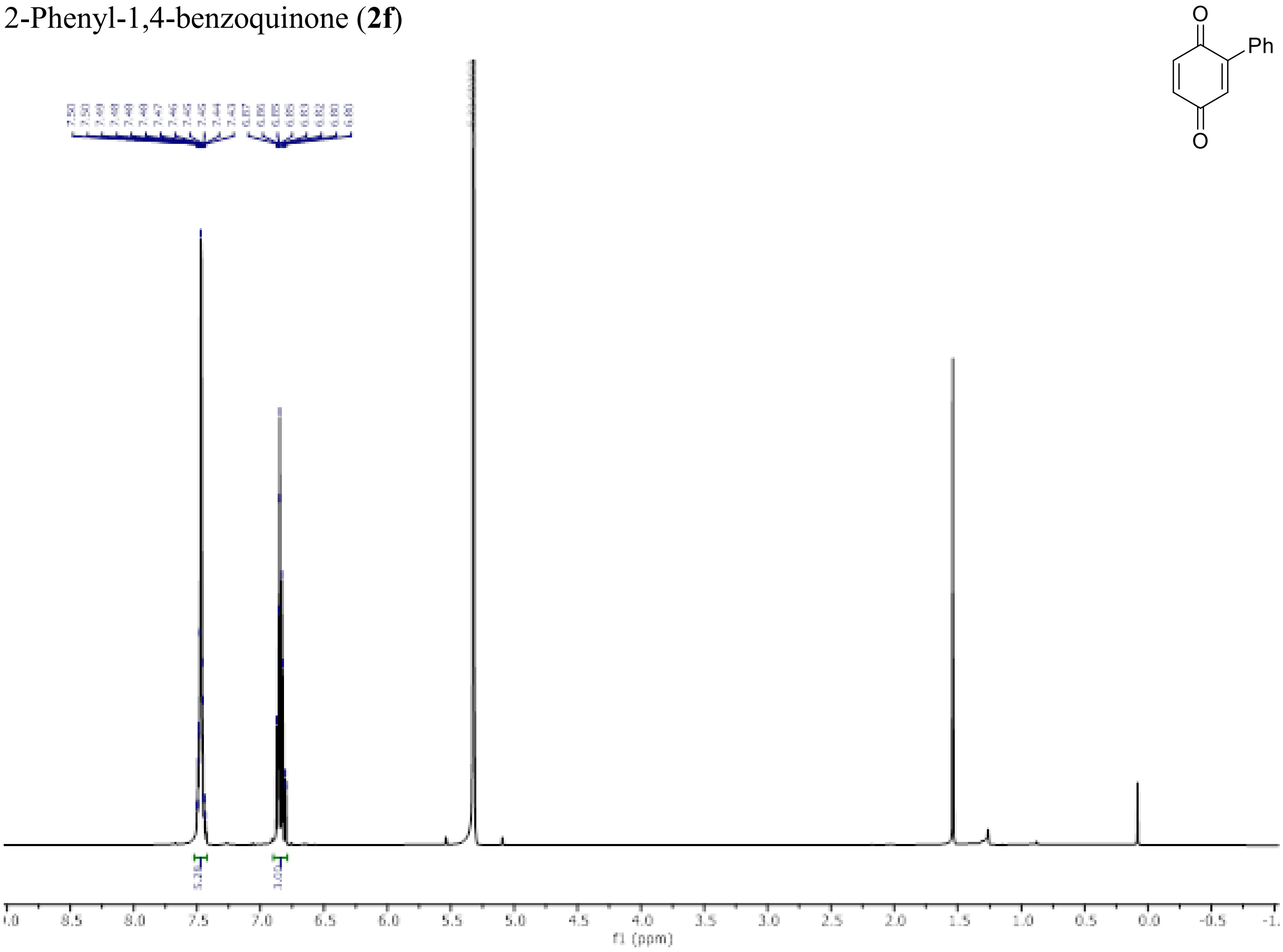

^{1}H NMR spectrum (400 MHz, CD_2Cl_2) of **2f**.

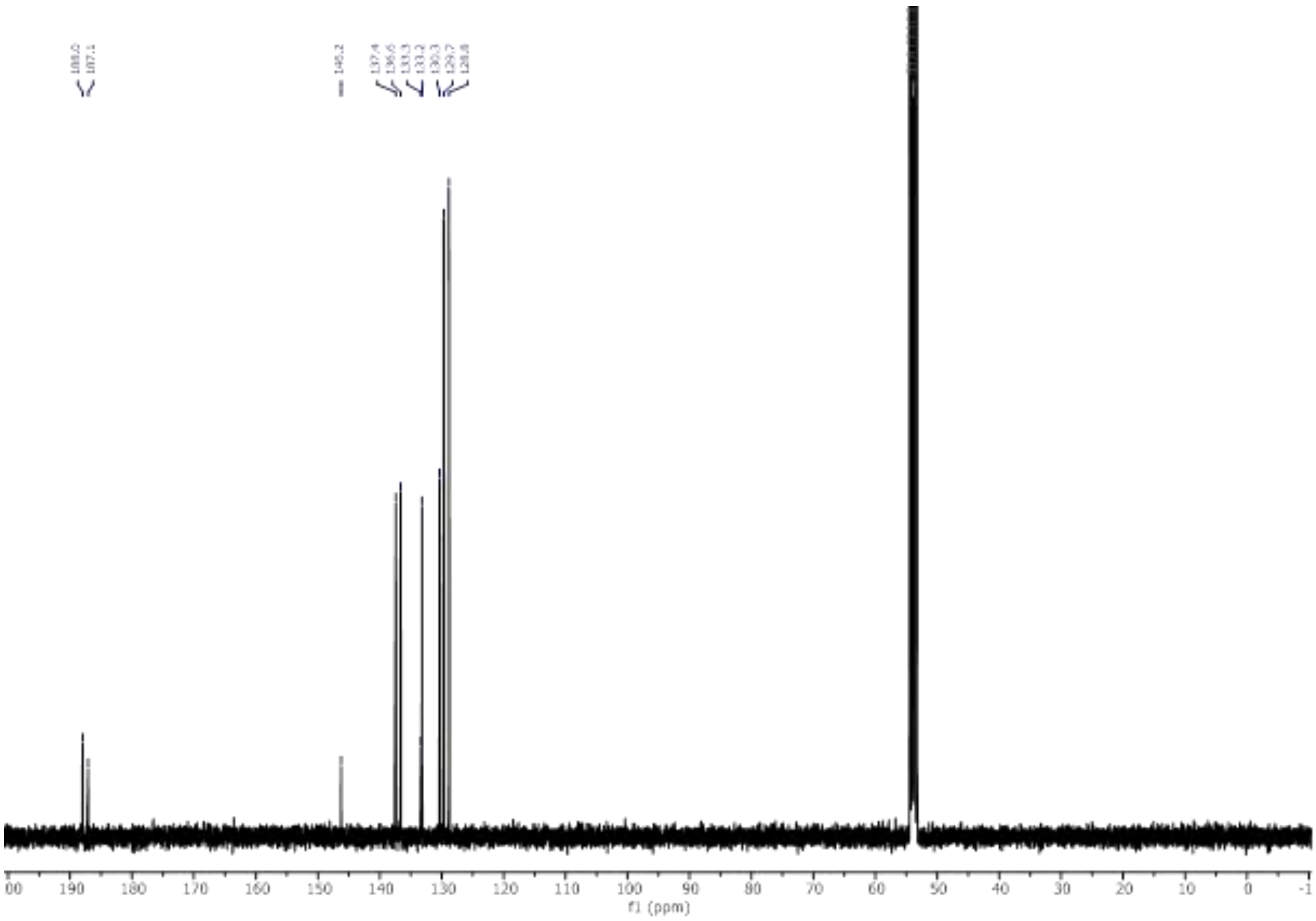

^{13}C NMR spectrum (101 MHz, CD_2Cl_2) of **2f**.

2-Acetamido-1,4-benzoquinone (**2h**)

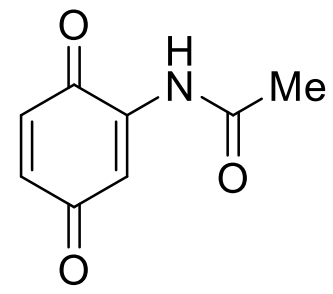

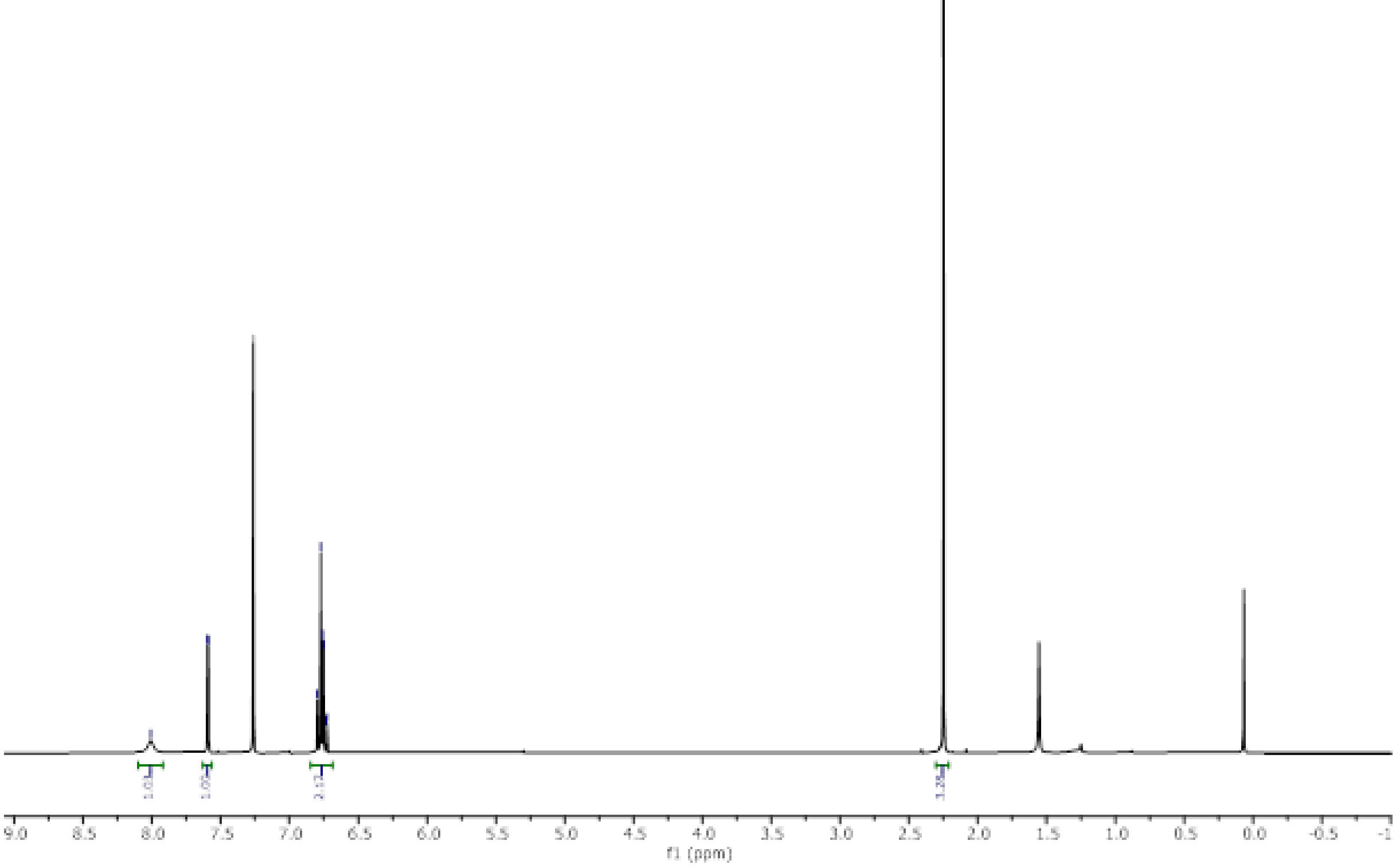

^{1}H NMR spectrum (400 MHz, $CDCl_3$) of **2h**.

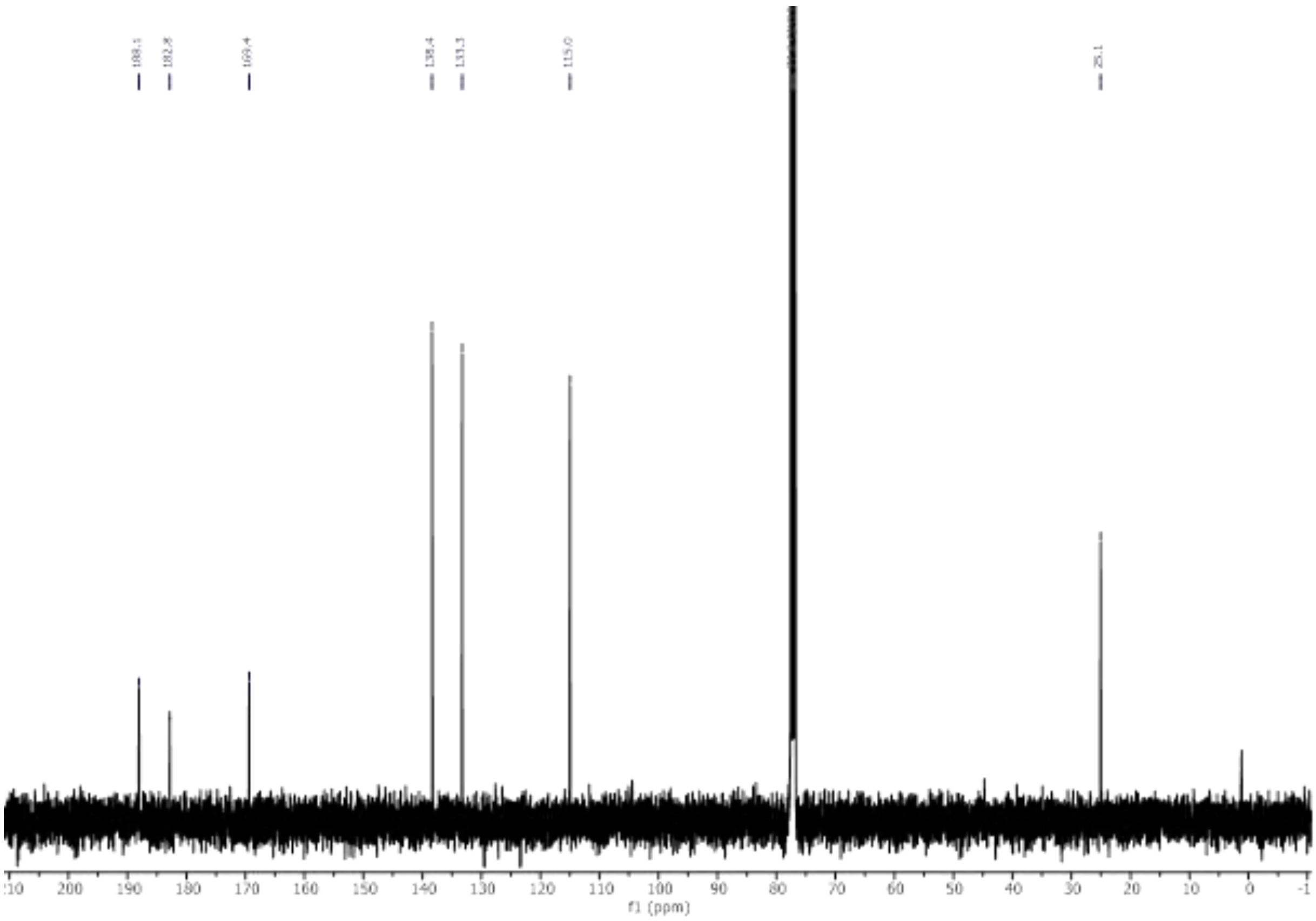

^{13}C NMR spectrum (101 MHz, $CDCl_3$) of **2h**.

5,8-Dihydro-5,8-diphenylnaphthalene-1,4-dione (**4a**)

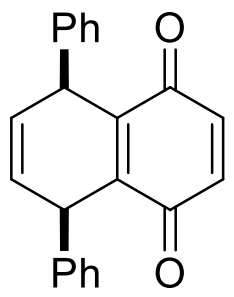

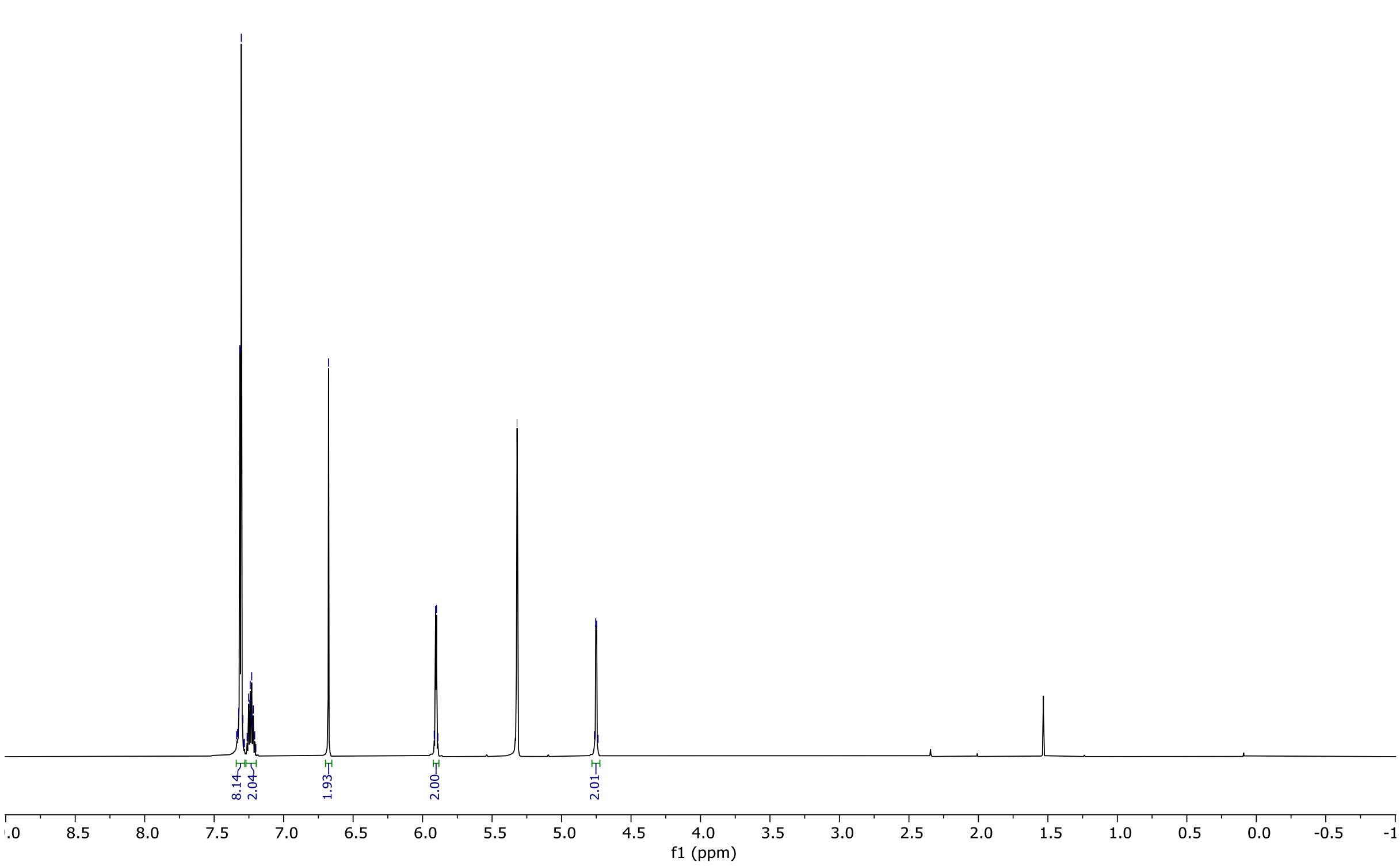

^{1}H NMR spectrum (400 MHz, CD_2Cl_2) of **4**.

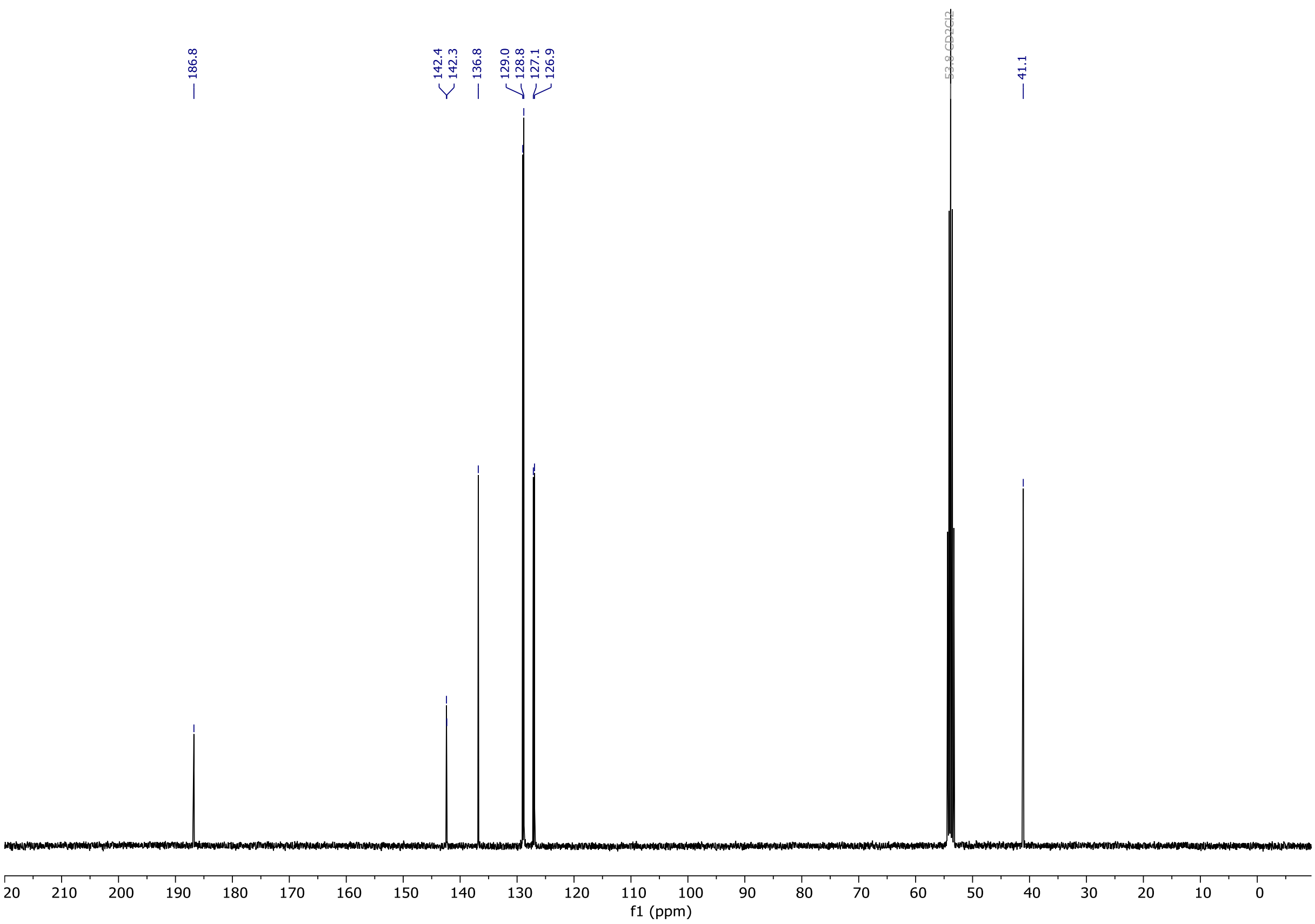

^{13}C NMR spectrum (101 MHz, CD_2Cl_2) of **4a**.

5,8-Dihydro-5-(4-biphenyl)-8-phenylnaphthalene-1,4-dione (**4b**)

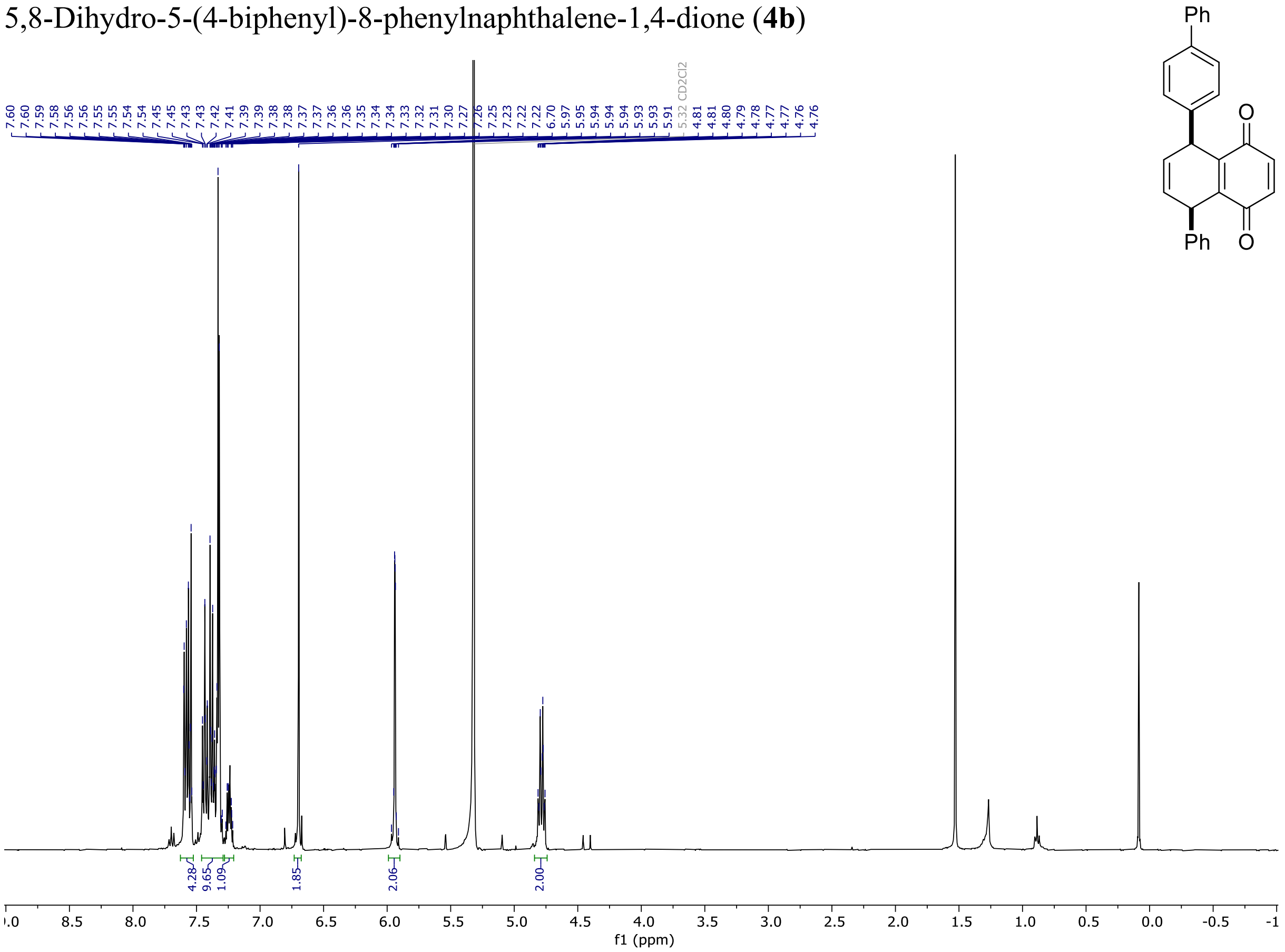

^{1}H NMR spectrum (400 MHz, CD_2Cl_2) of **4b**.

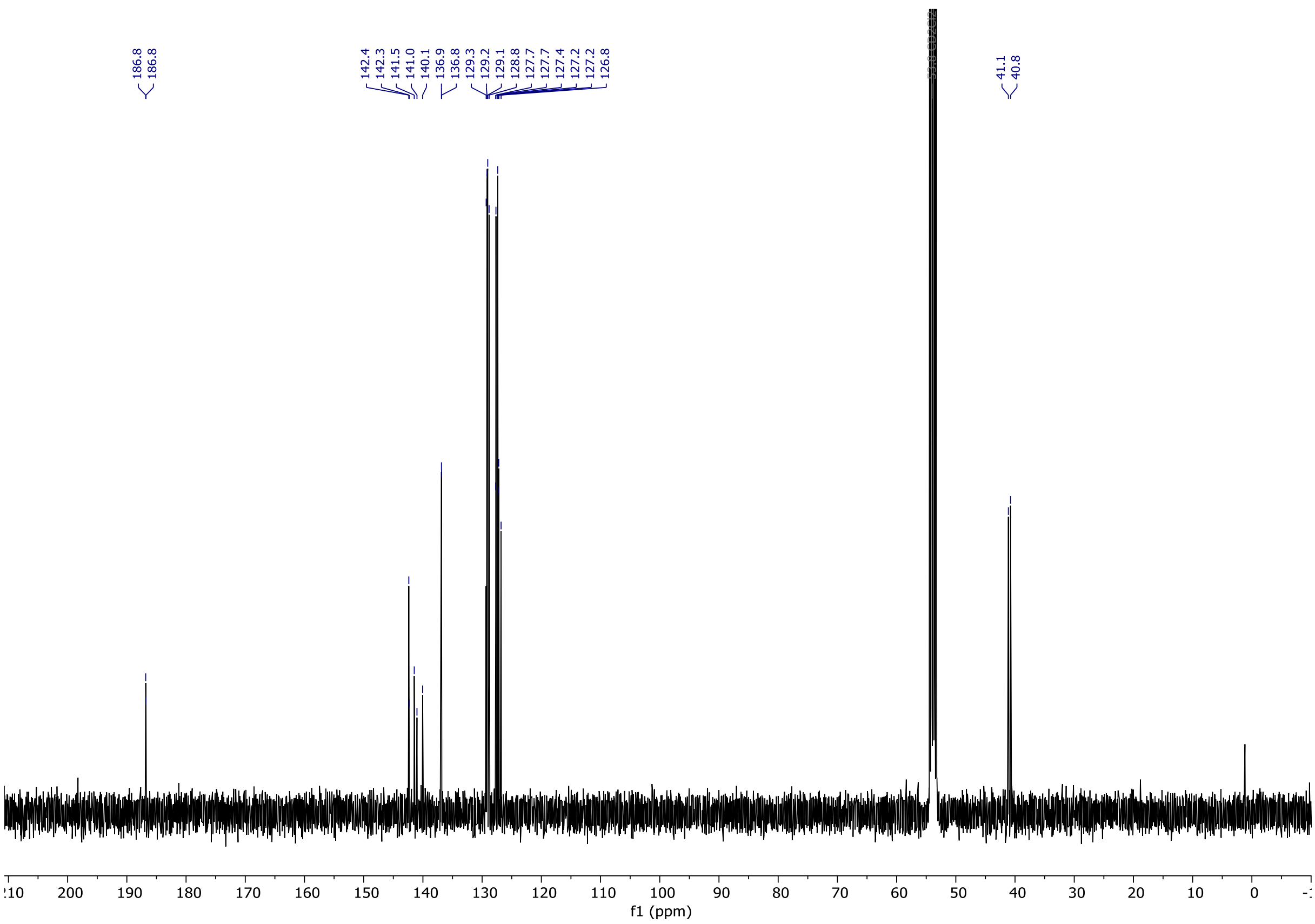

^{13}C NMR spectrum (101 MHz, CD_2Cl_2) of **4b**.

5,8-Dihydro-5-(2-methylphenyl)-8-phenylnaphthalene-1,4-dione (**4c**)

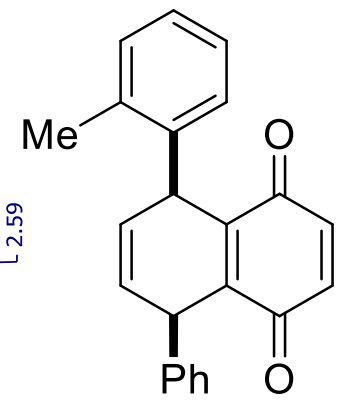

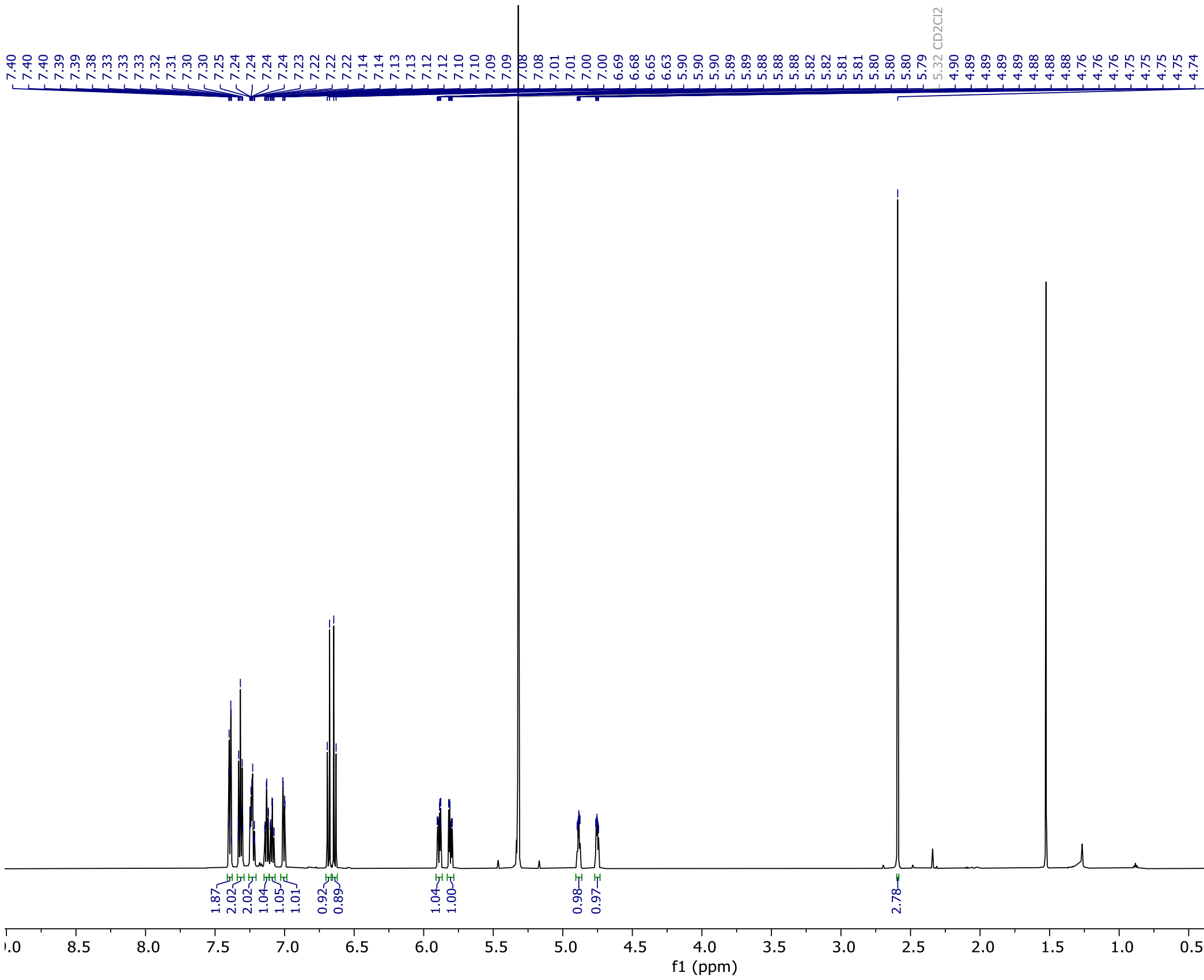

^{1}H NMR spectrum (600 MHz, CD_2Cl_2) of **4c**.

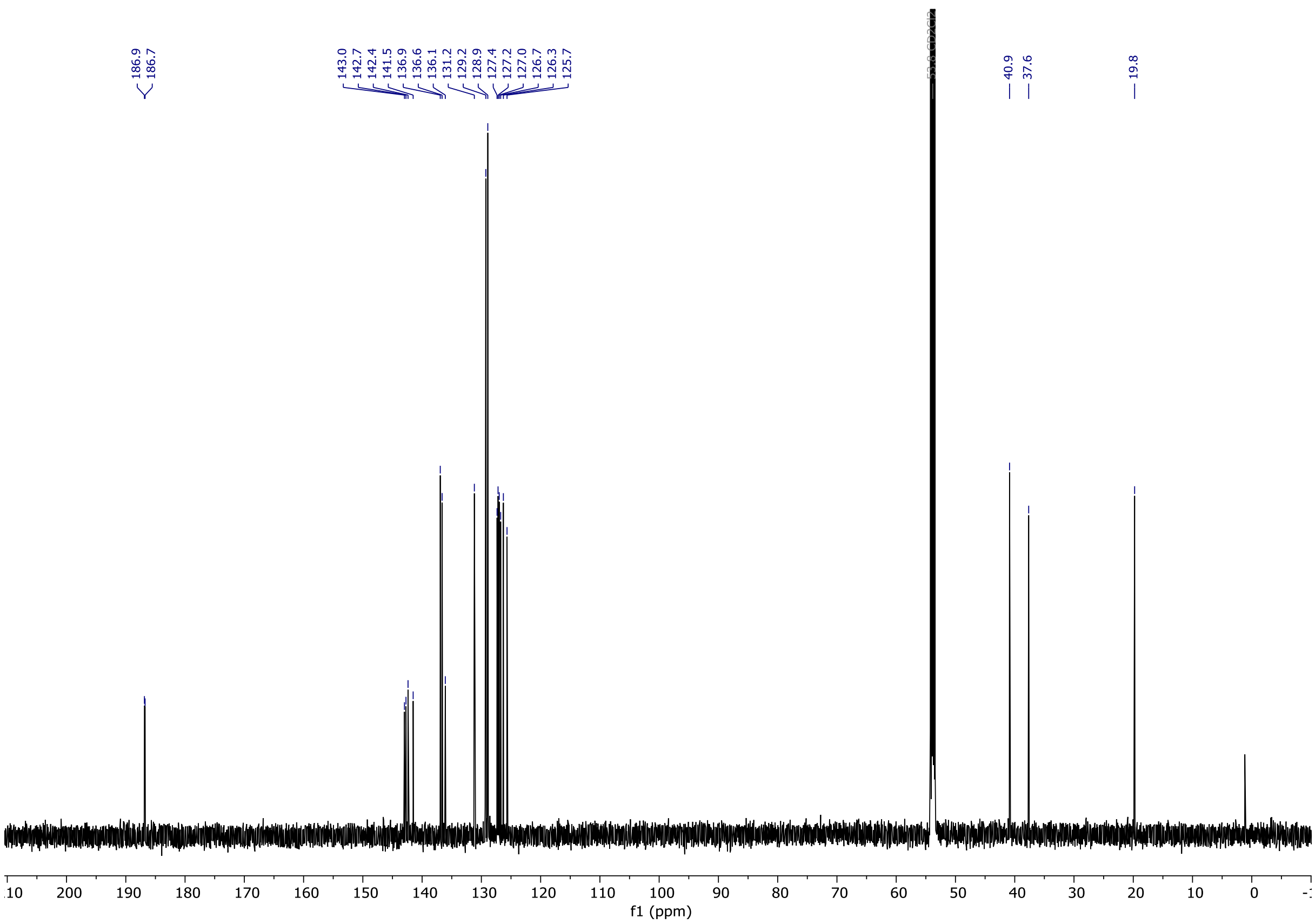

^{13}C NMR spectrum (151 MHz, CD_2Cl_2) of **4c**.

5,8-Diyhdro-5-(3-methylphenyl)-8-phenylnaphthalene-1,4-dione (**4d**)

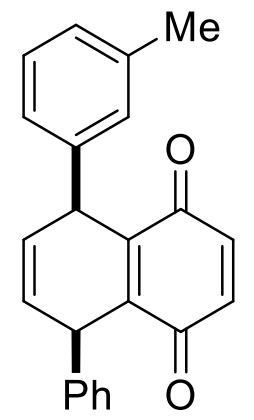

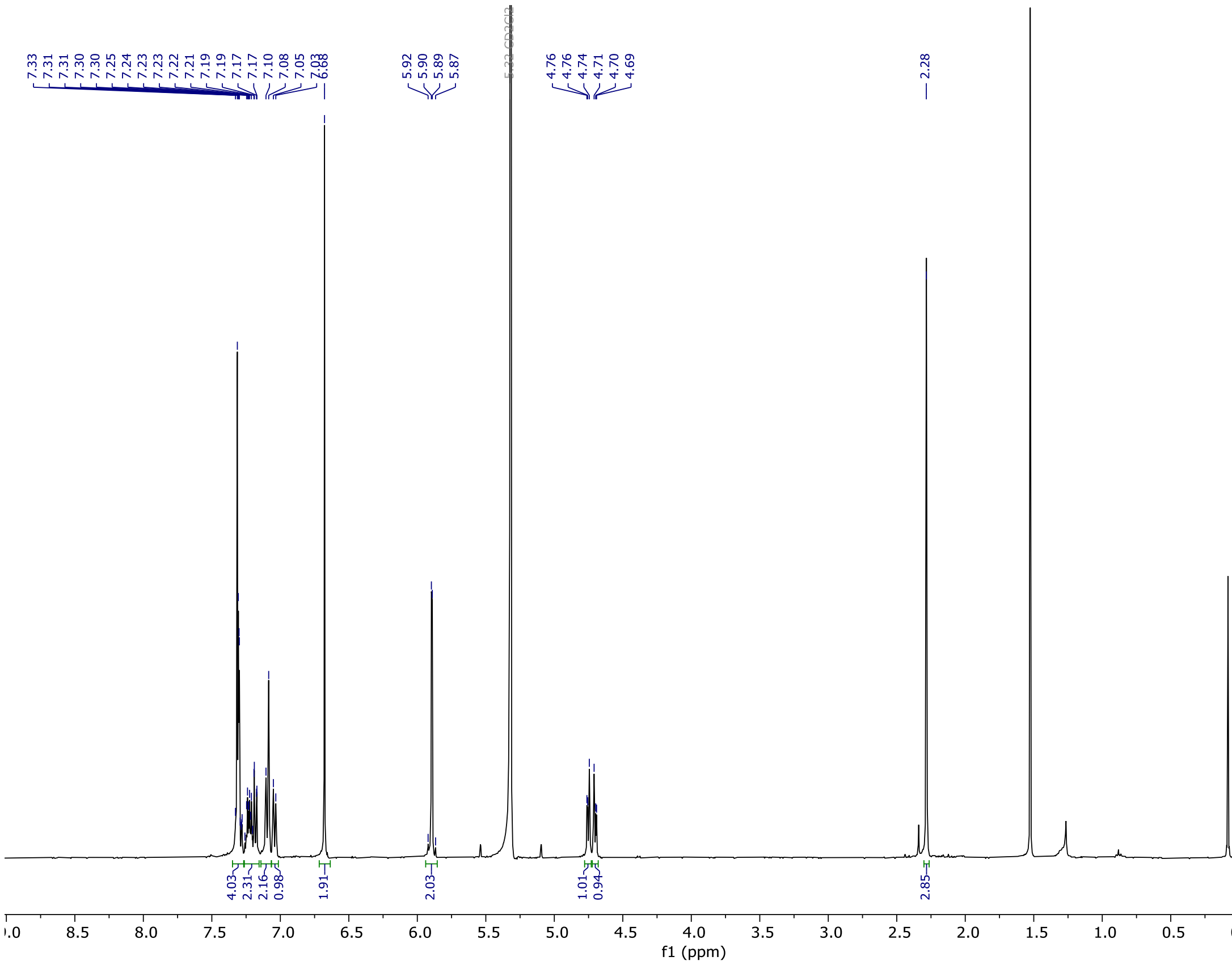

^{1}H NMR spectrum (400 MHz, CD_2Cl_2) of **4d**.

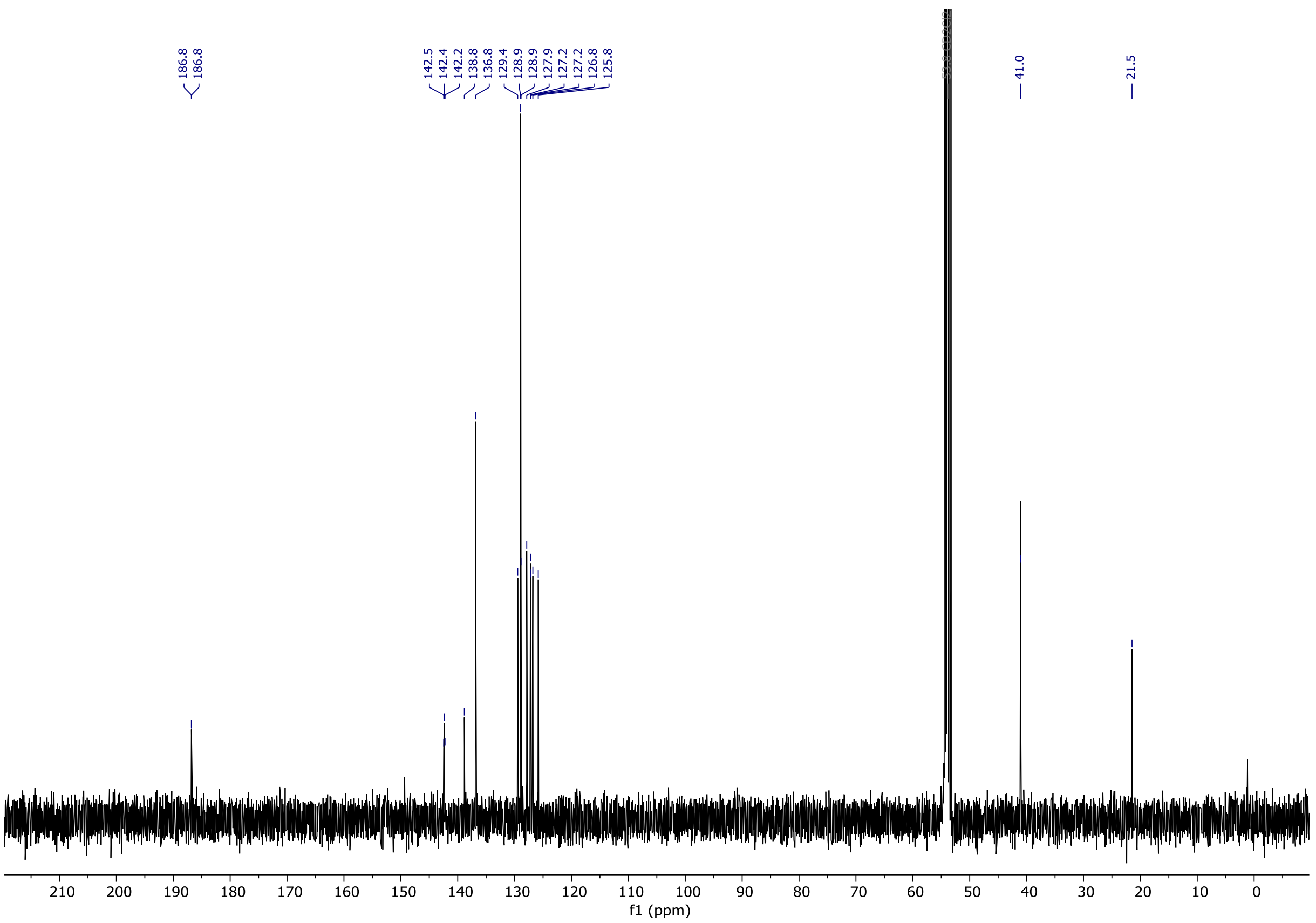

^{13}C NMR spectrum (101 MHz, CD_2Cl_2) of **4d**.

5,8-Dihydro-5-(4-methylphenyl)-8-phenylnaphthalene-1,4-dione (**4e**)

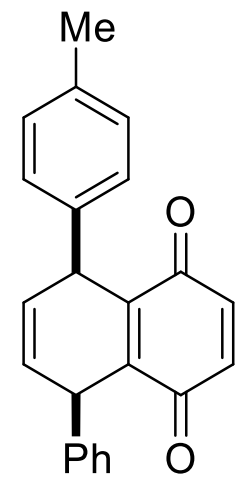

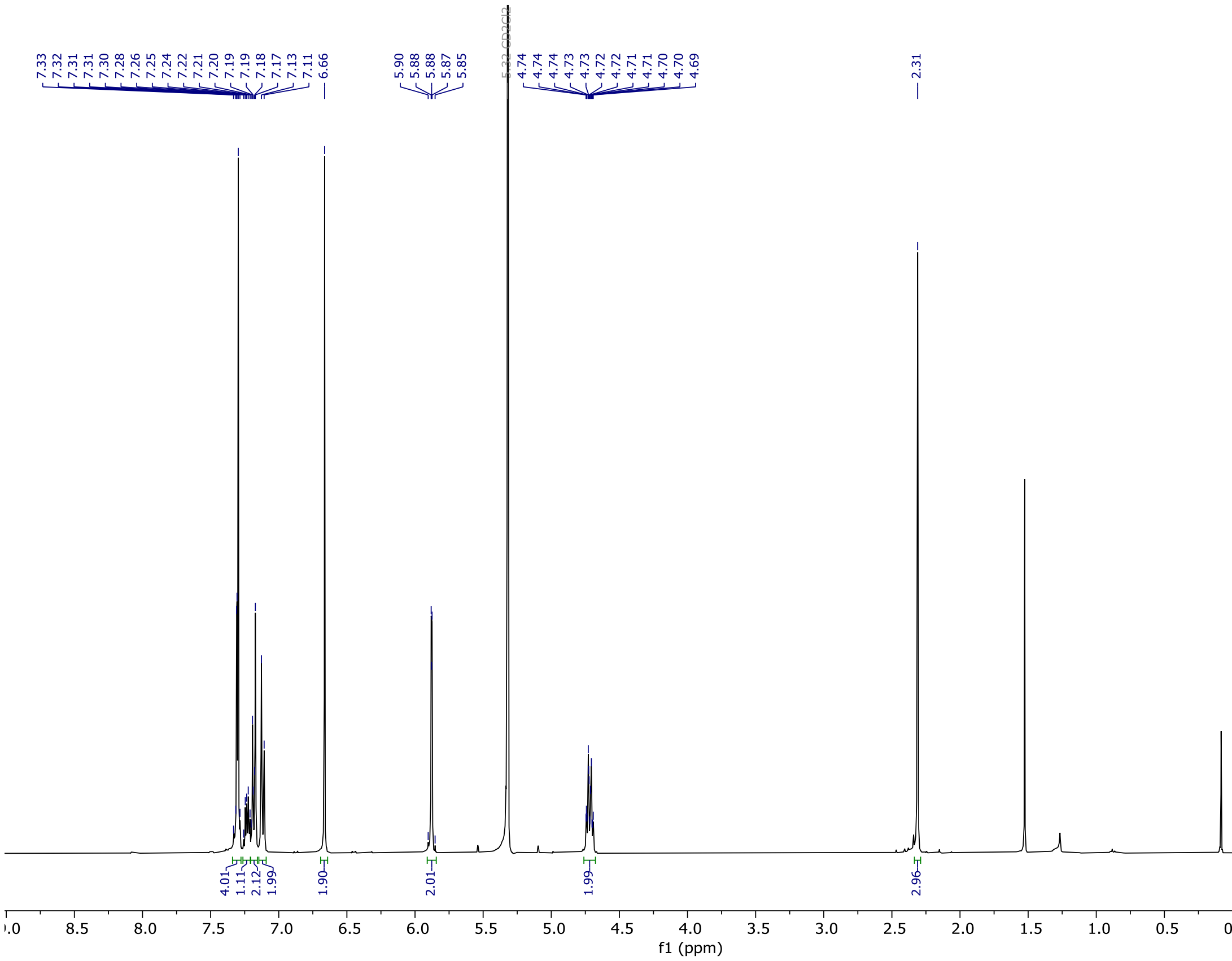

^{1}H NMR spectrum (400 MHz, CD_2Cl_2) of **4e**.

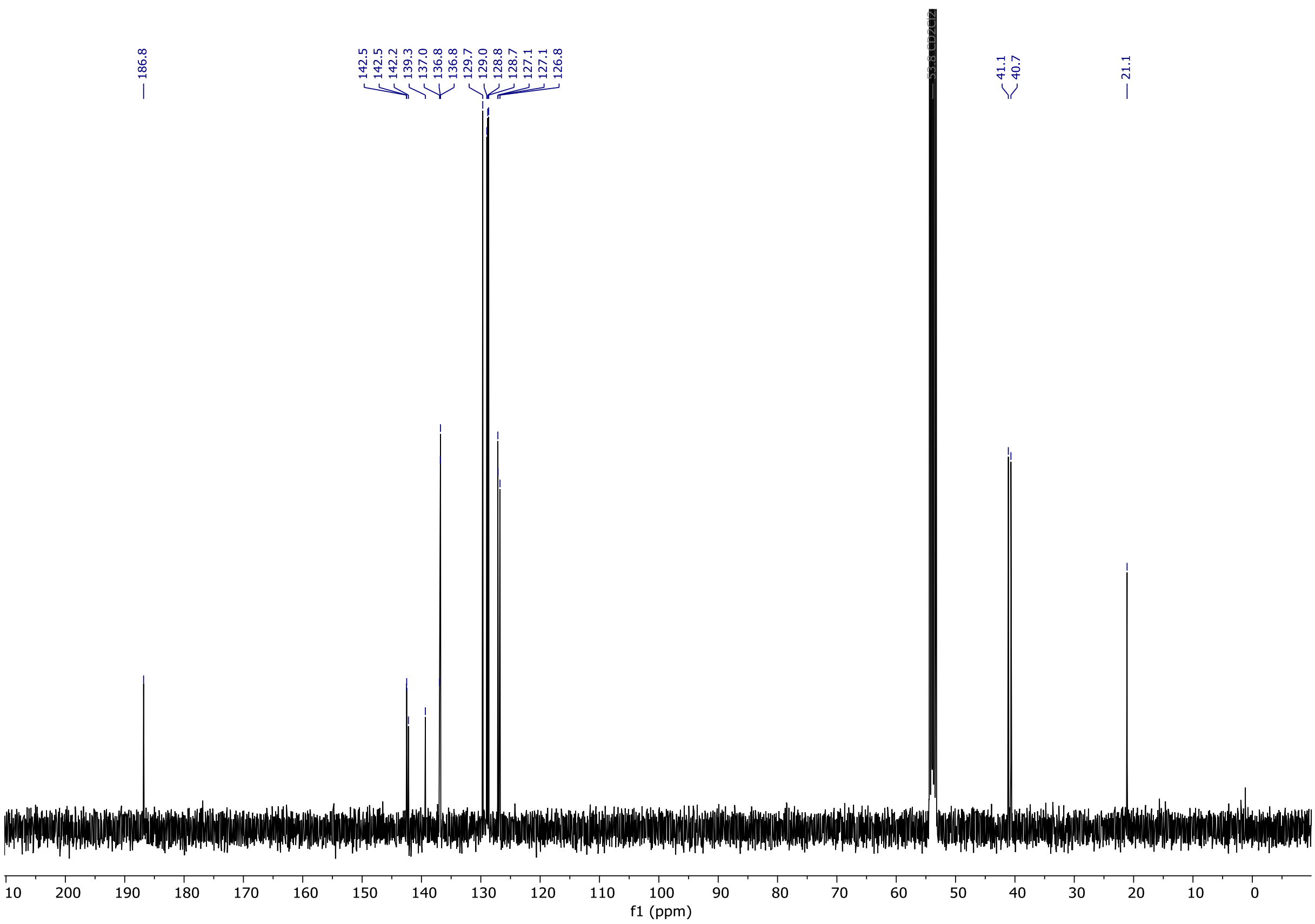

^{13}C NMR spectrum (101 MHz, CD_2Cl_2) of **4e**.

5,8-Dihydro-5-(4-butylphenyl)-8-phenylnaphthalene-1,4-dione (**4f**)

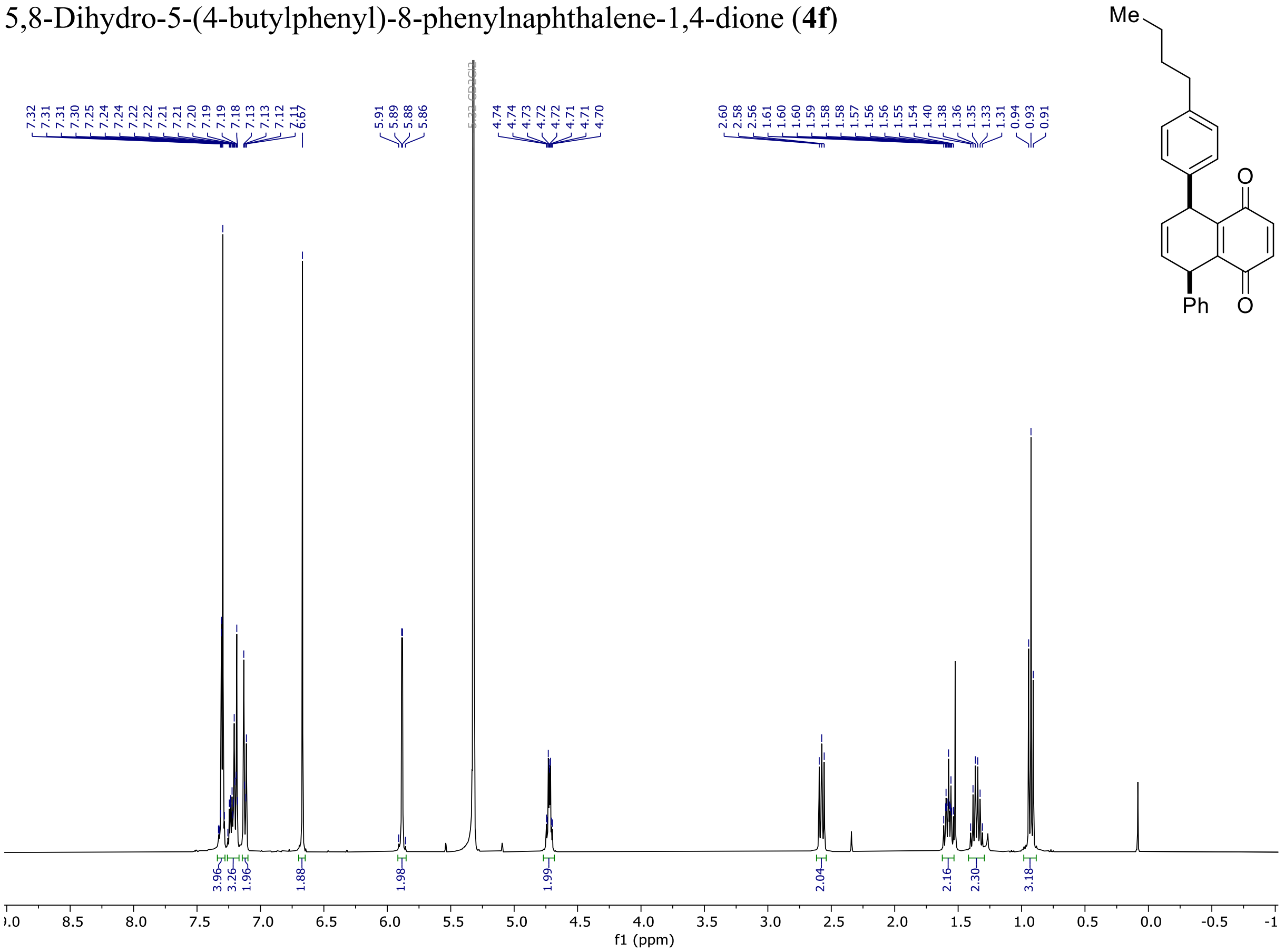

^{1}H NMR spectrum (400 MHz, CD_2Cl_2) of **4f**.

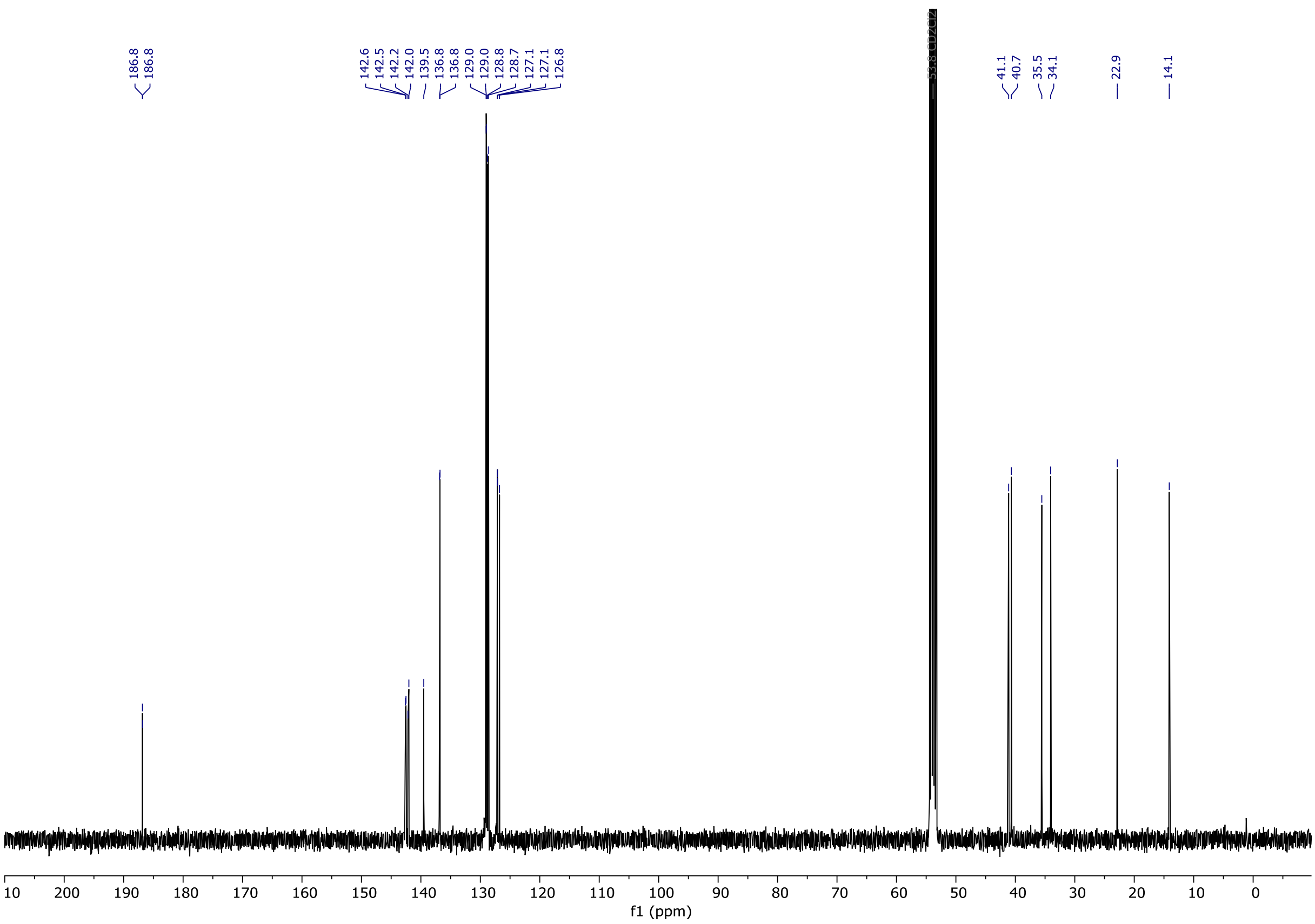

^{13}C NMR spectrum (101 MHz, CD_2Cl_2) of **4f**.

5,8-Dihydro-5-(4-tert-butylphenyl)-8-phenylnaphthalene-1,4-dione (**4g**)

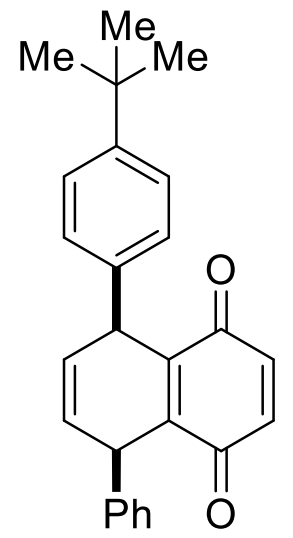

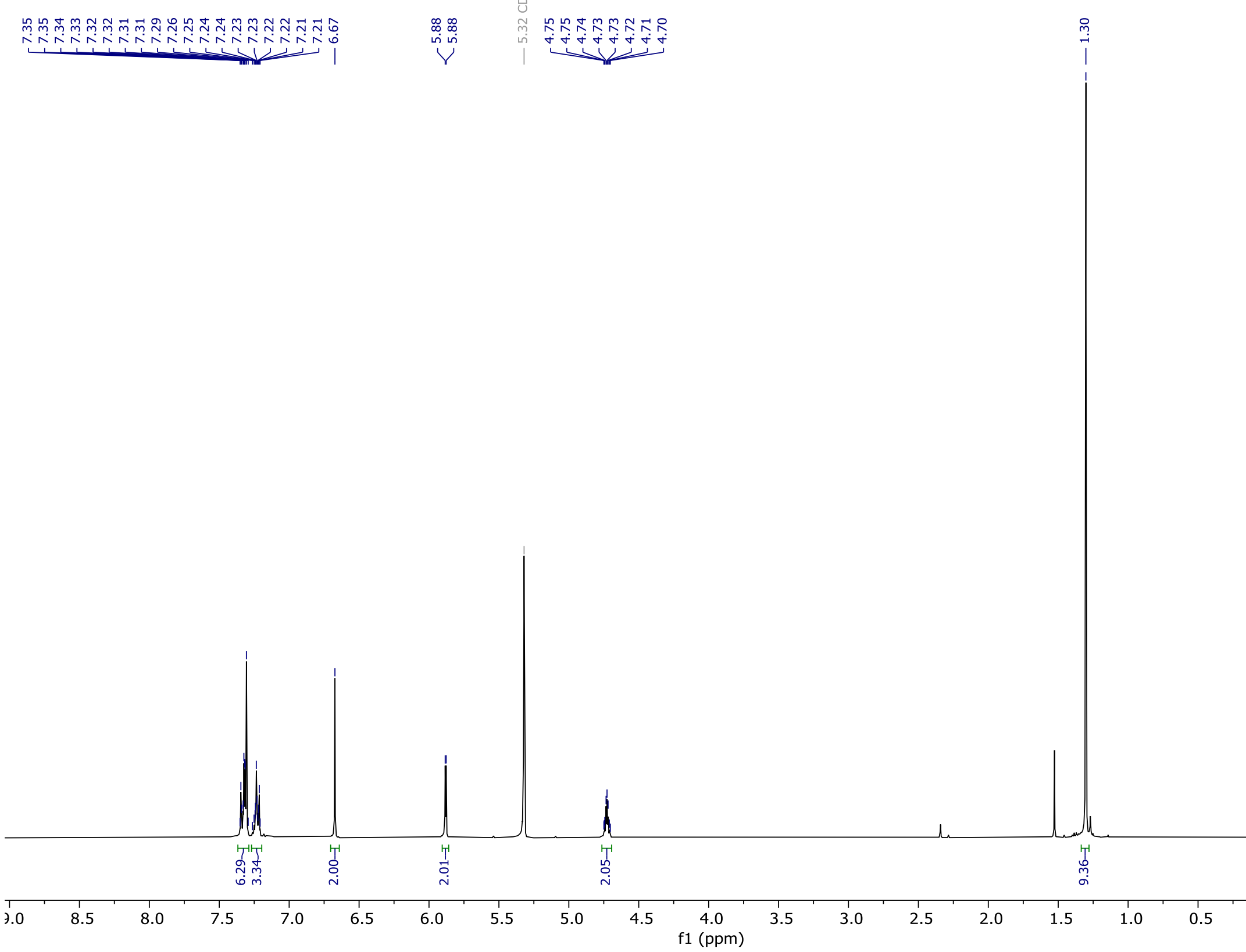

^{1}H NMR spectrum (400 MHz, CD_2Cl_2) of **4g**.

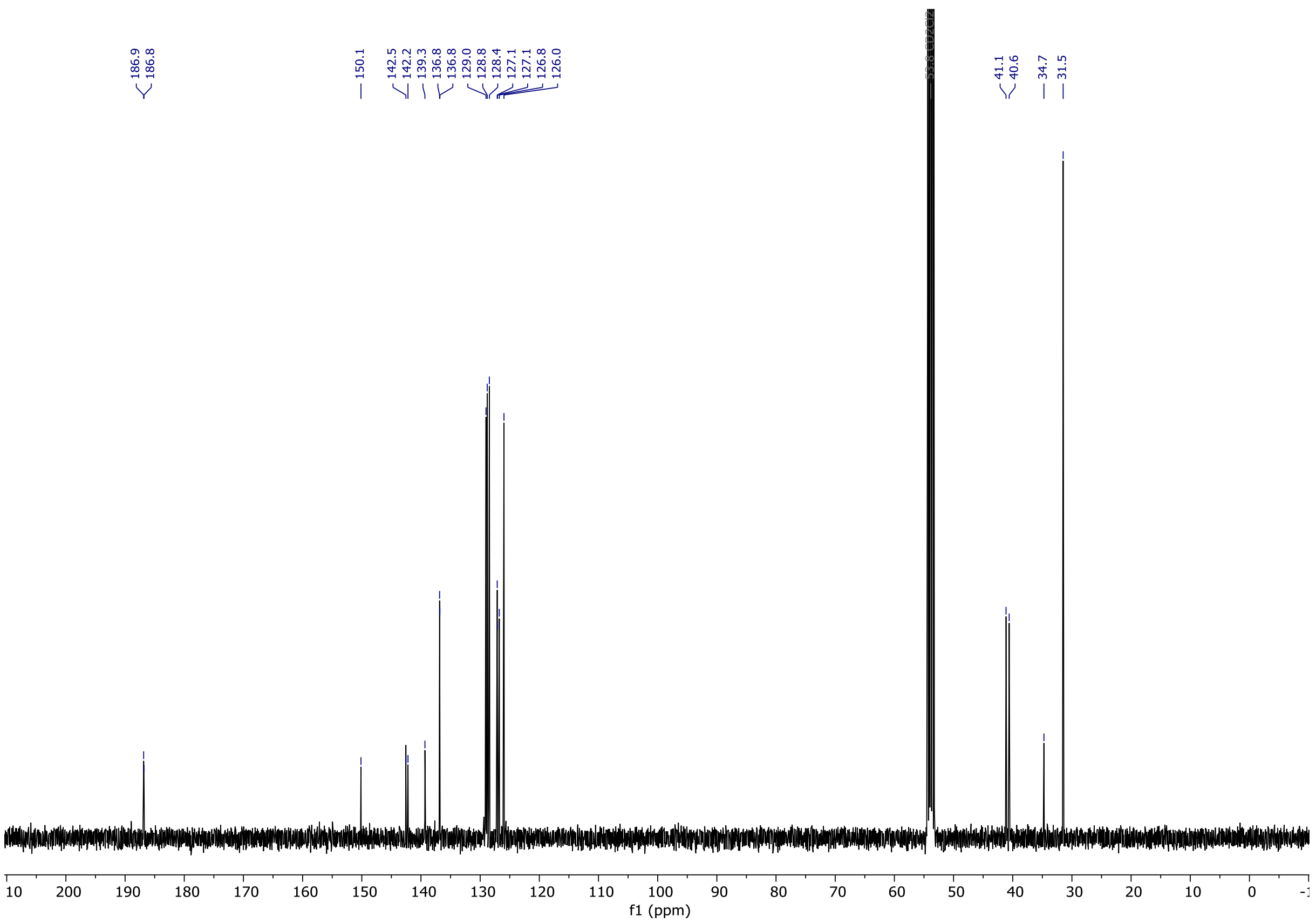

^{13}C NMR spectrum (101 MHz, CD_2Cl_2) of **4g**.

5,8-Dihydro-5-(2-methoxyphenyl)-8-phenylnaphthalene-1,4-dione (**4h**)

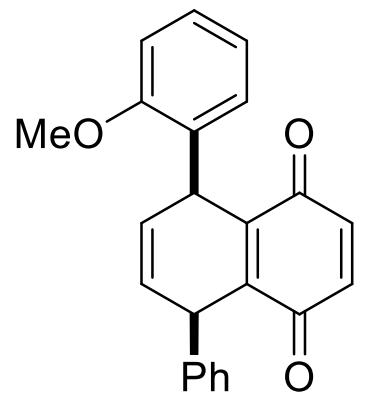

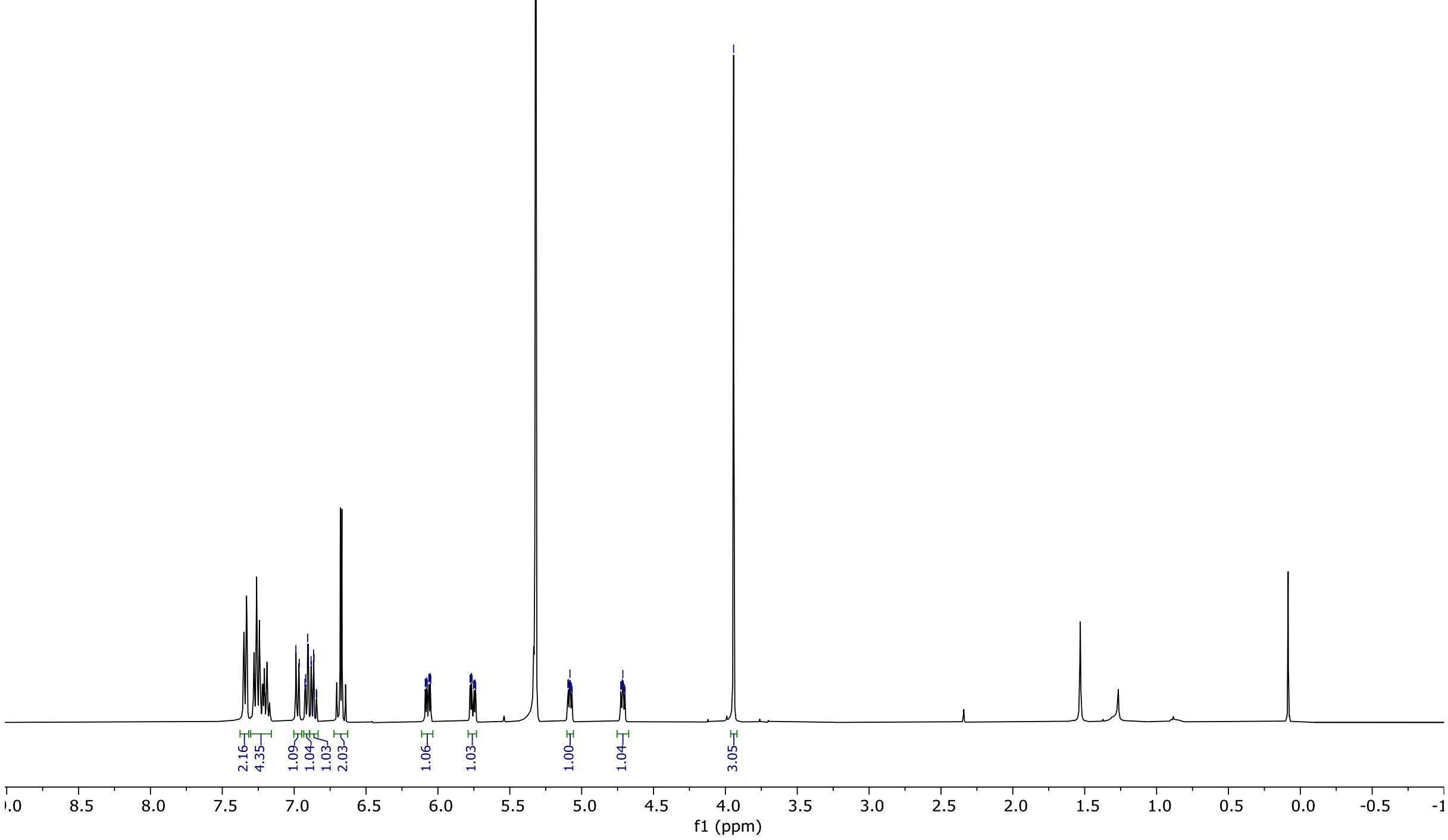

^{1}H NMR spectrum (400 MHz, CD_2Cl_2) of **4h**.

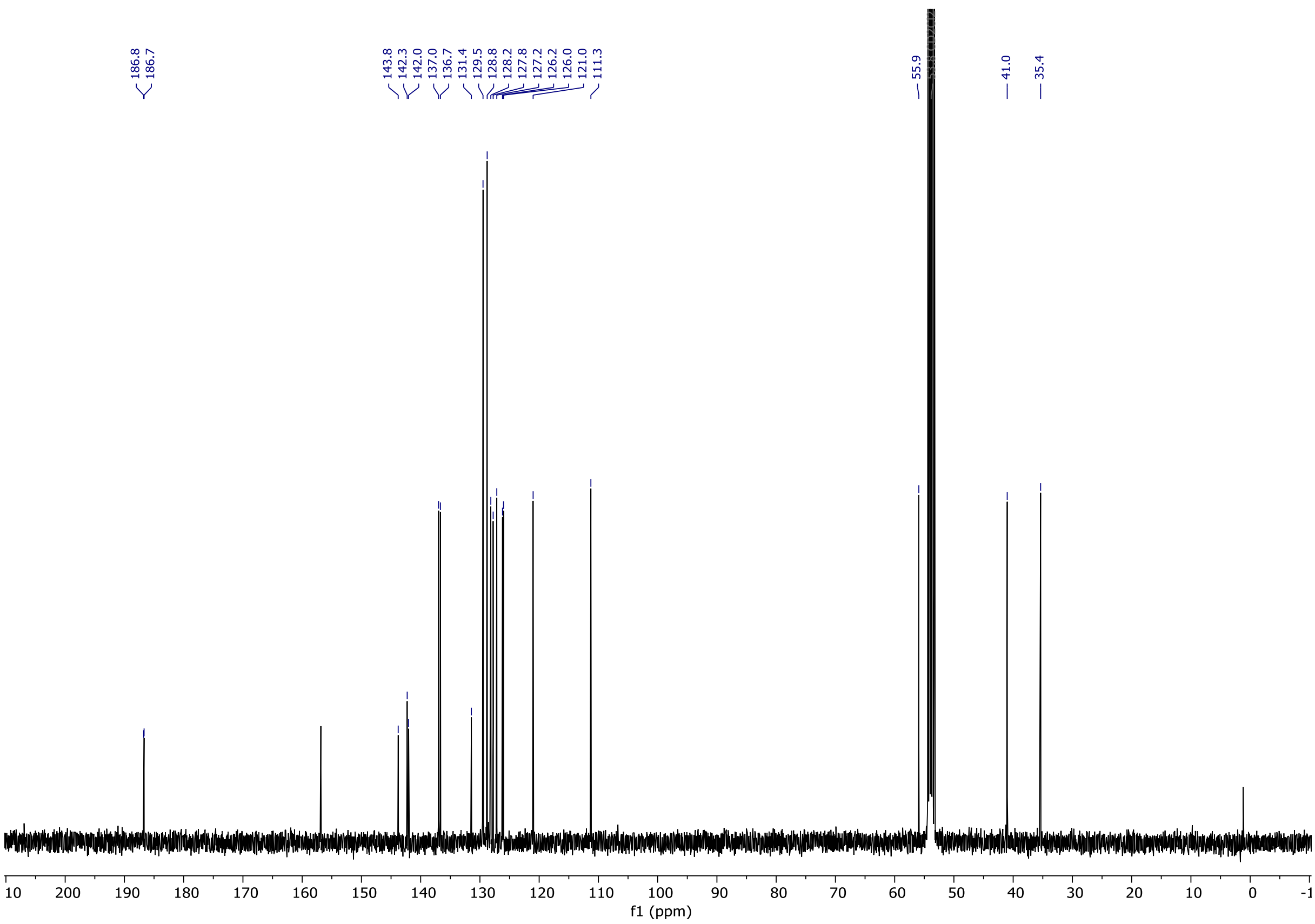

^{13}C NMR spectrum (101 MHz, CD_2Cl_2) of **4h**.

5,8-Dihydro-5-(3-methoxyphenyl)-8-phenylnaphthalene-1,4-dione (**4i**)

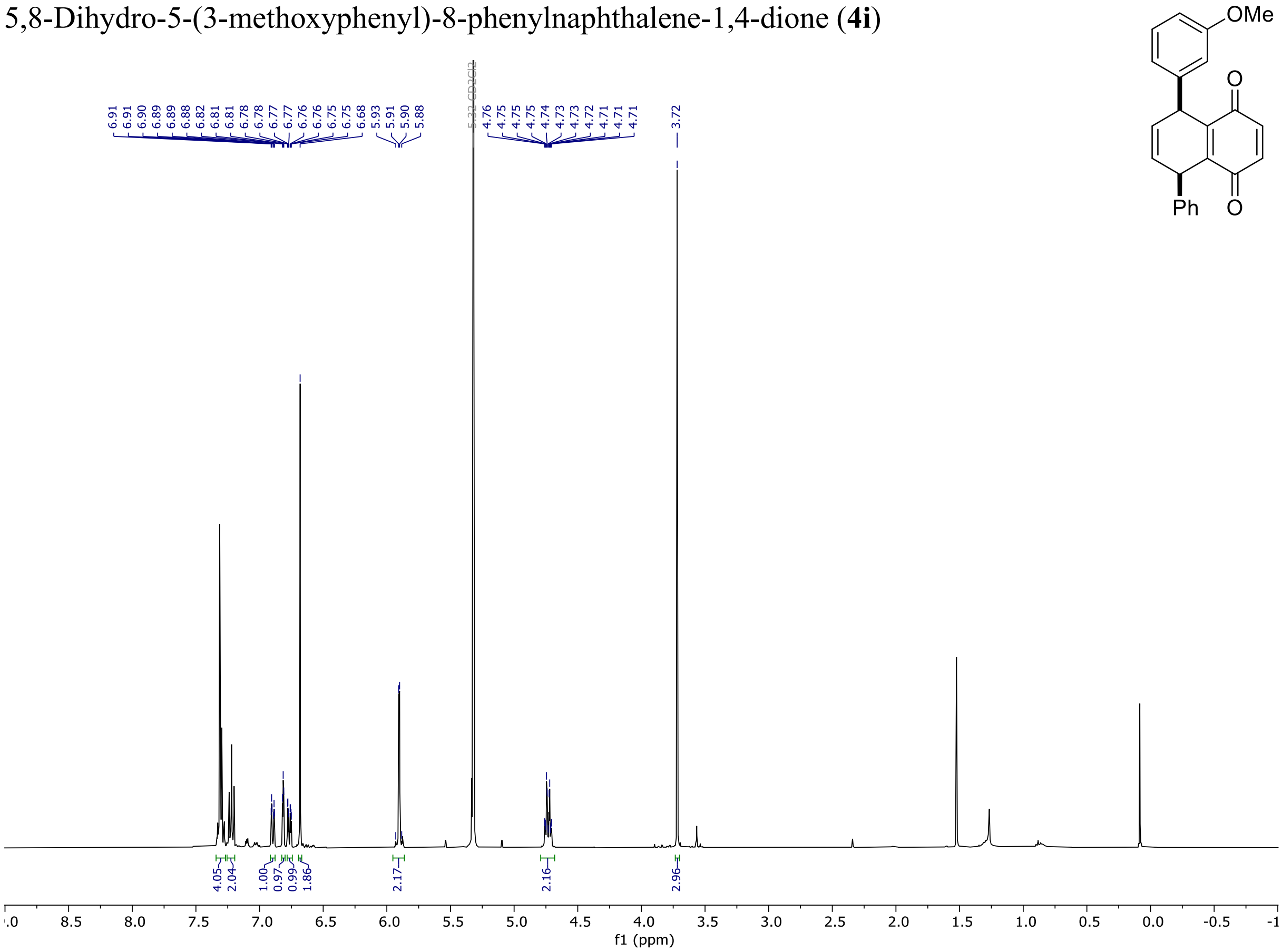

^{1}H NMR spectrum (400 MHz, CD_2Cl_2) of **4i**.

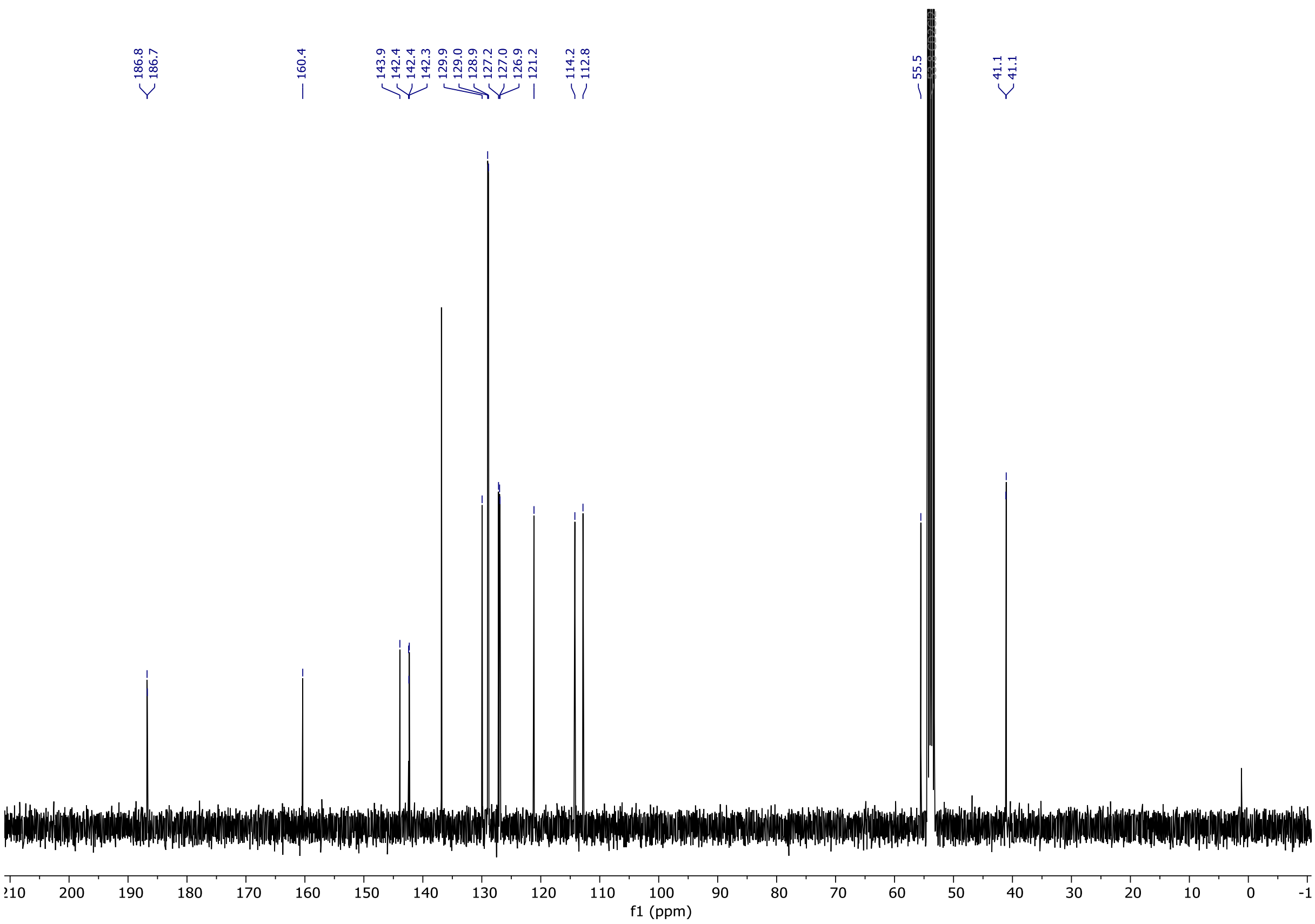

^{13}C NMR spectrum (101 MHz, CD_2Cl_2) of **4i**.

5,8-Dihydro-5-(4-methoxyphenyl)-8-phenylnaphthalene-1,4-dione (**4j**)

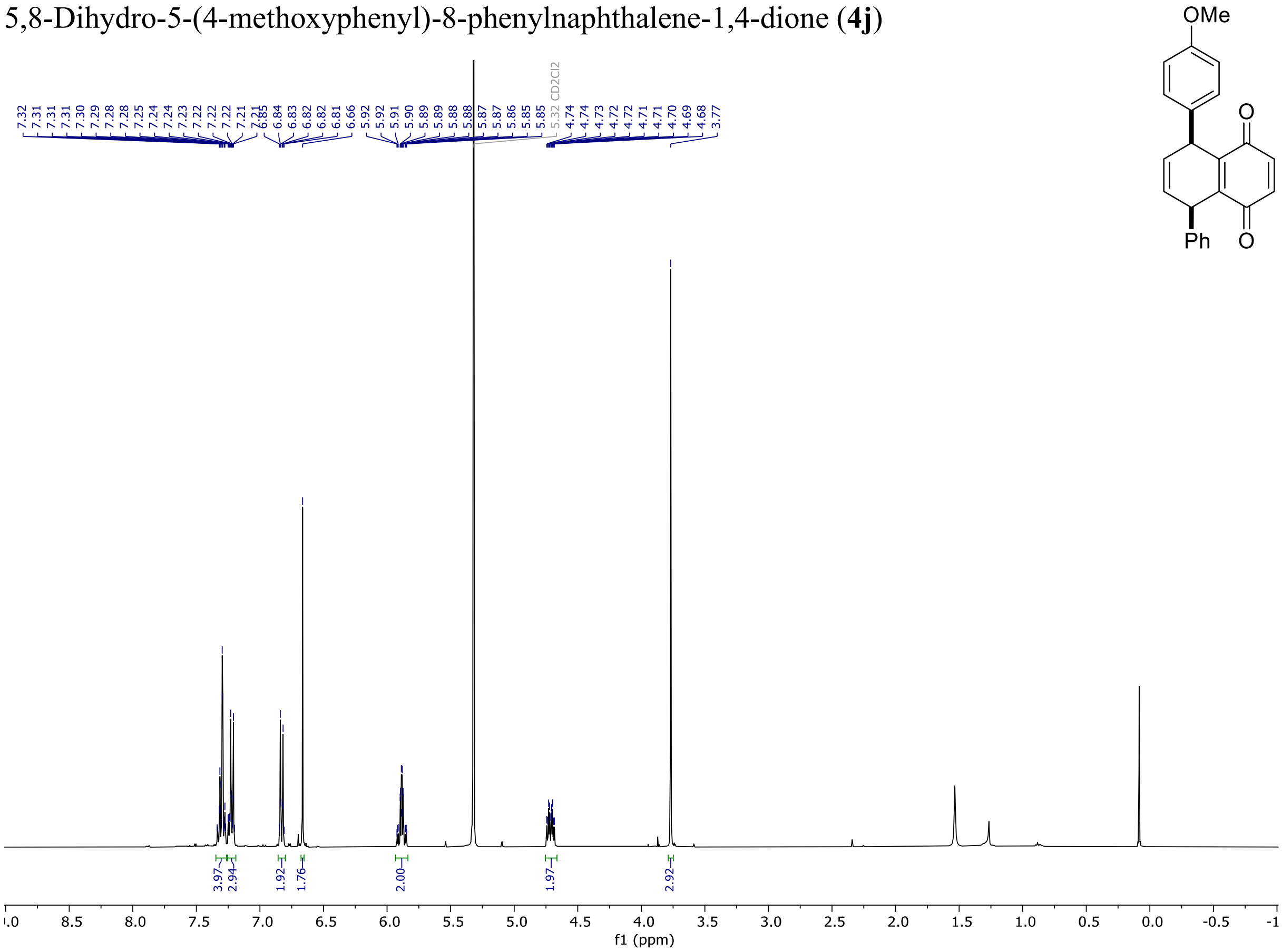

^{1}H NMR spectrum (400 MHz, CD_2Cl_2) of **4j**.

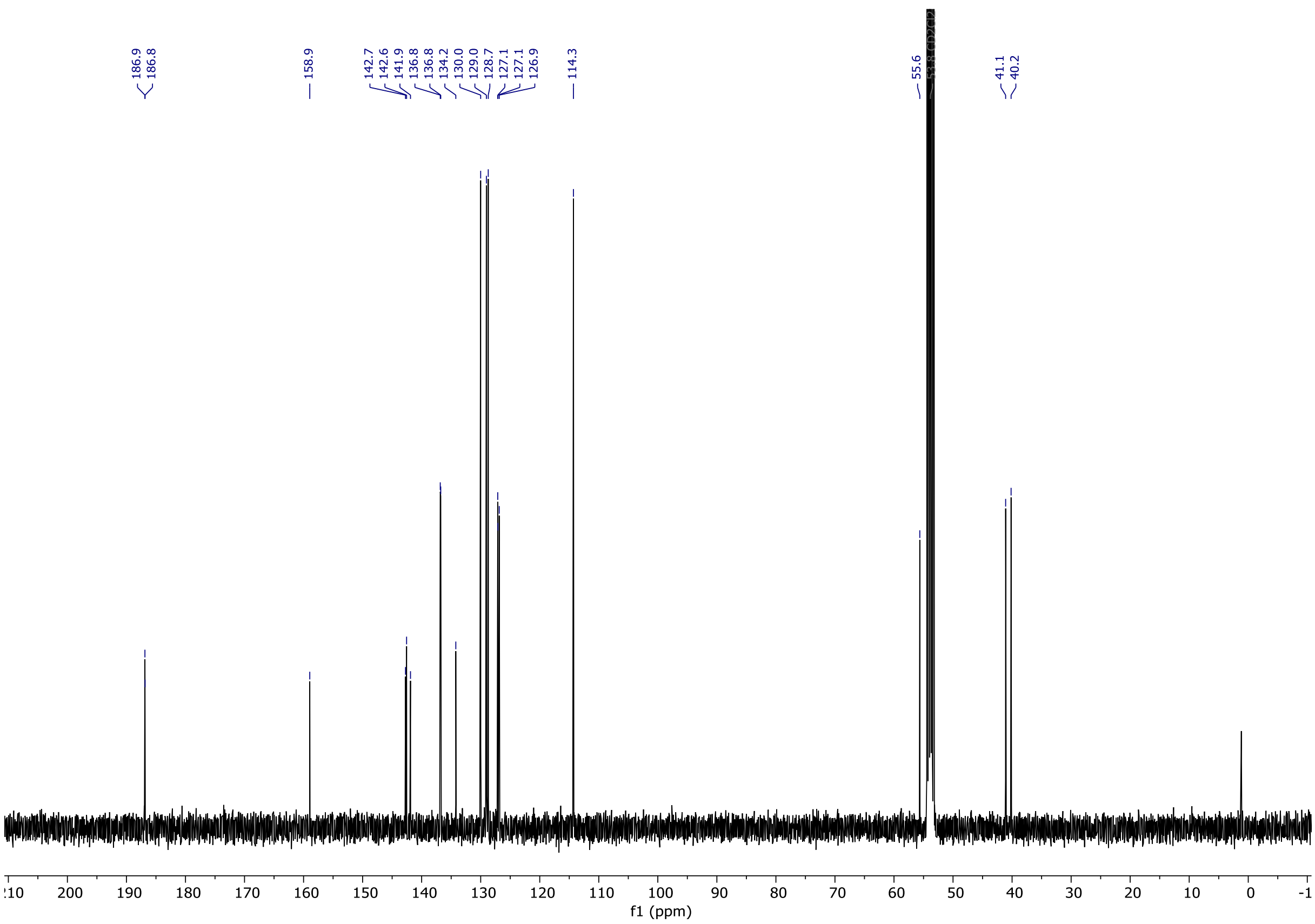

^{13}C NMR spectrum (101 MHz, CD_2Cl_2) of **4j**.

5,8-Dihydro-5-(1-naphthyl)-8-phenylnaphthalene-1,4-dione (**4k**)

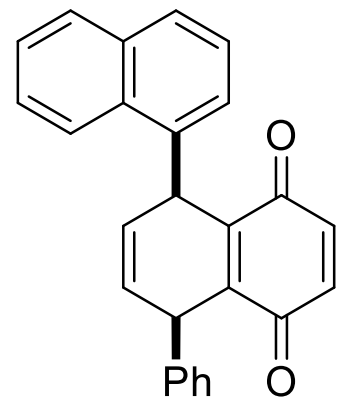

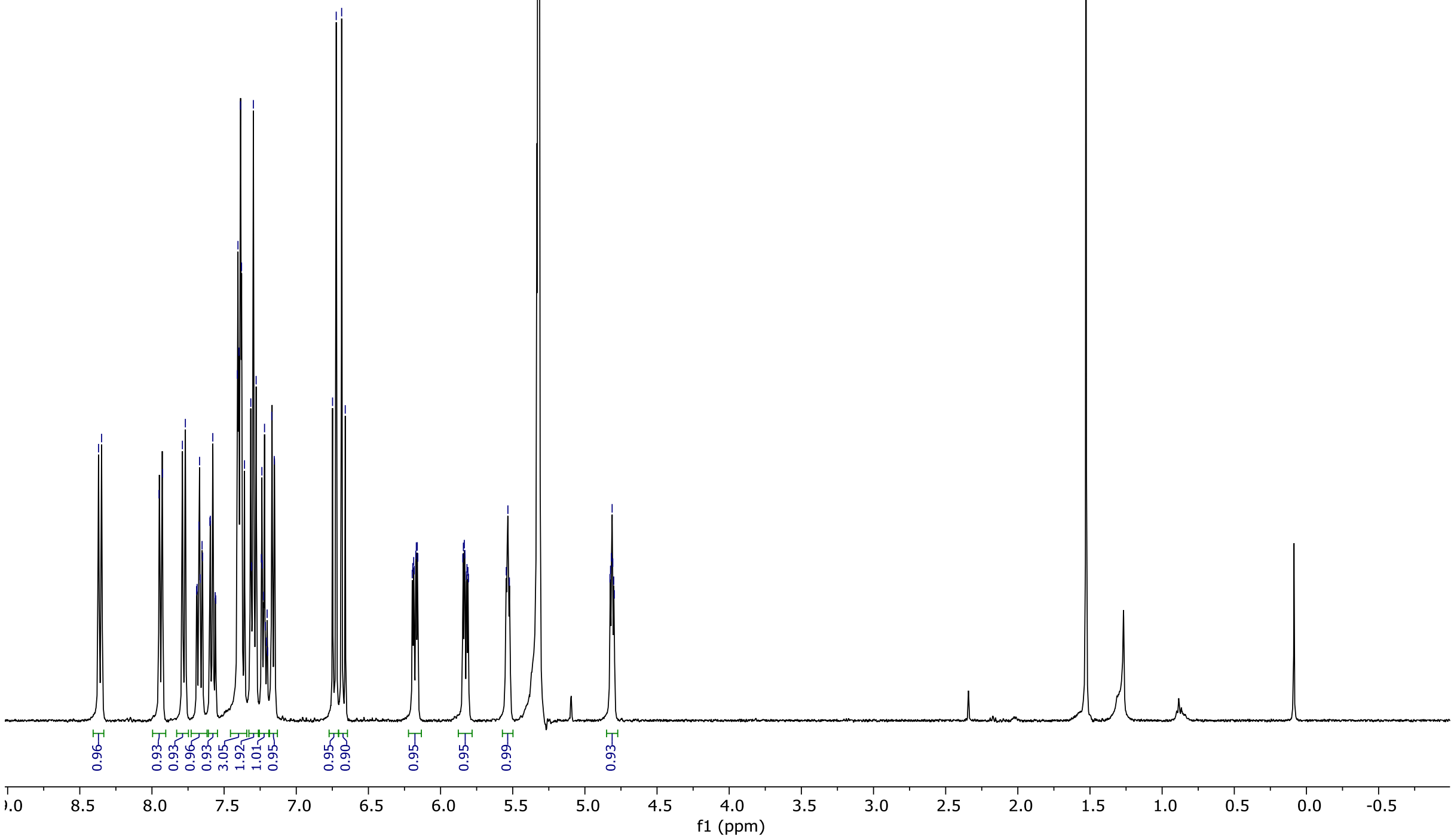

^{1}H NMR spectrum (400 MHz, CD_2Cl_2) of **4k**.

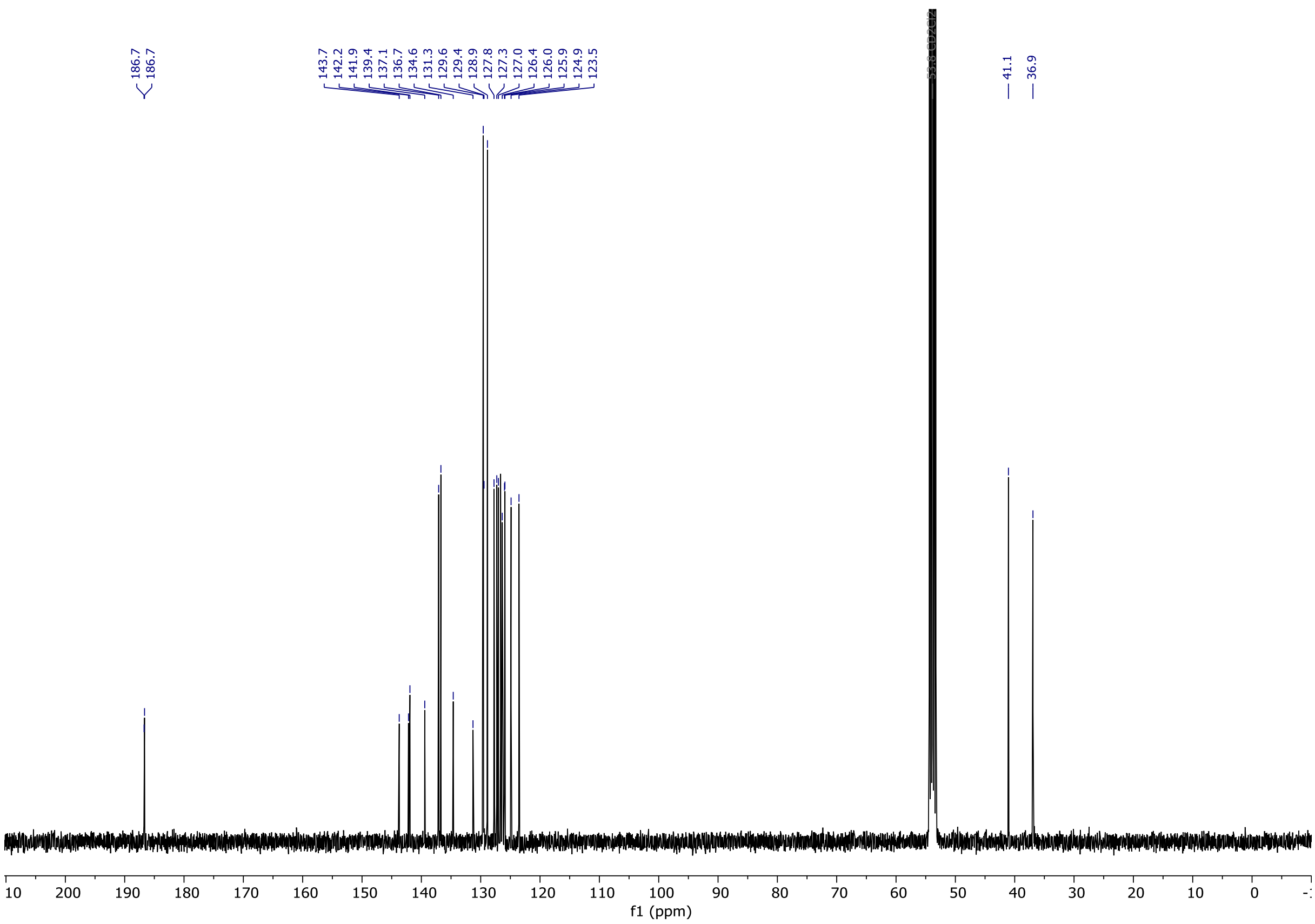

^{13}C NMR spectrum (101 MHz, CD_2Cl_2) of **4k**.

5,8-Dihydro-5-(2-naphthyl)-8-phenylnaphthalene-1,4-dione (**4l**)

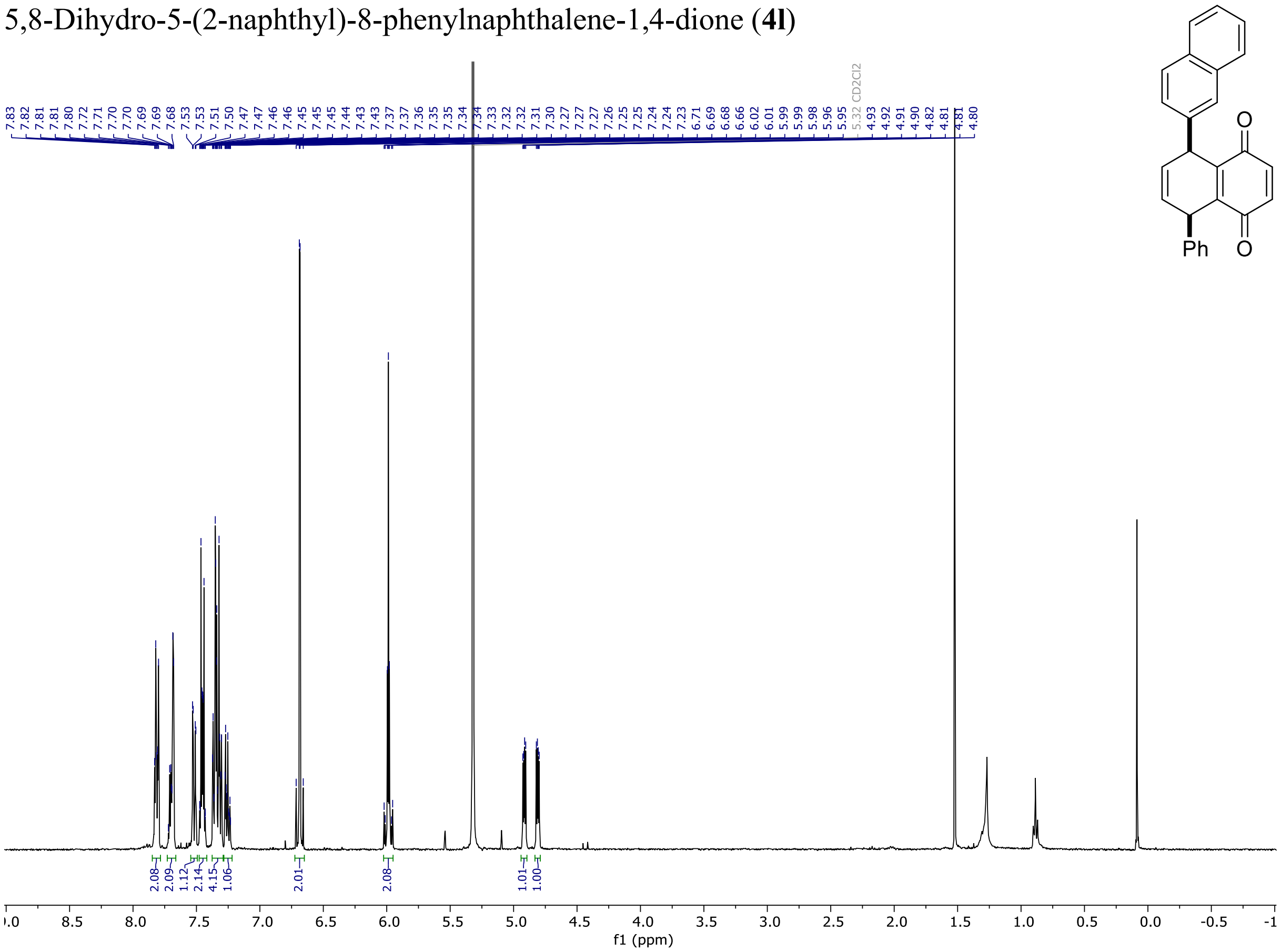

^{1}H NMR spectrum (400 MHz, CD_2Cl_2) of **4l**.

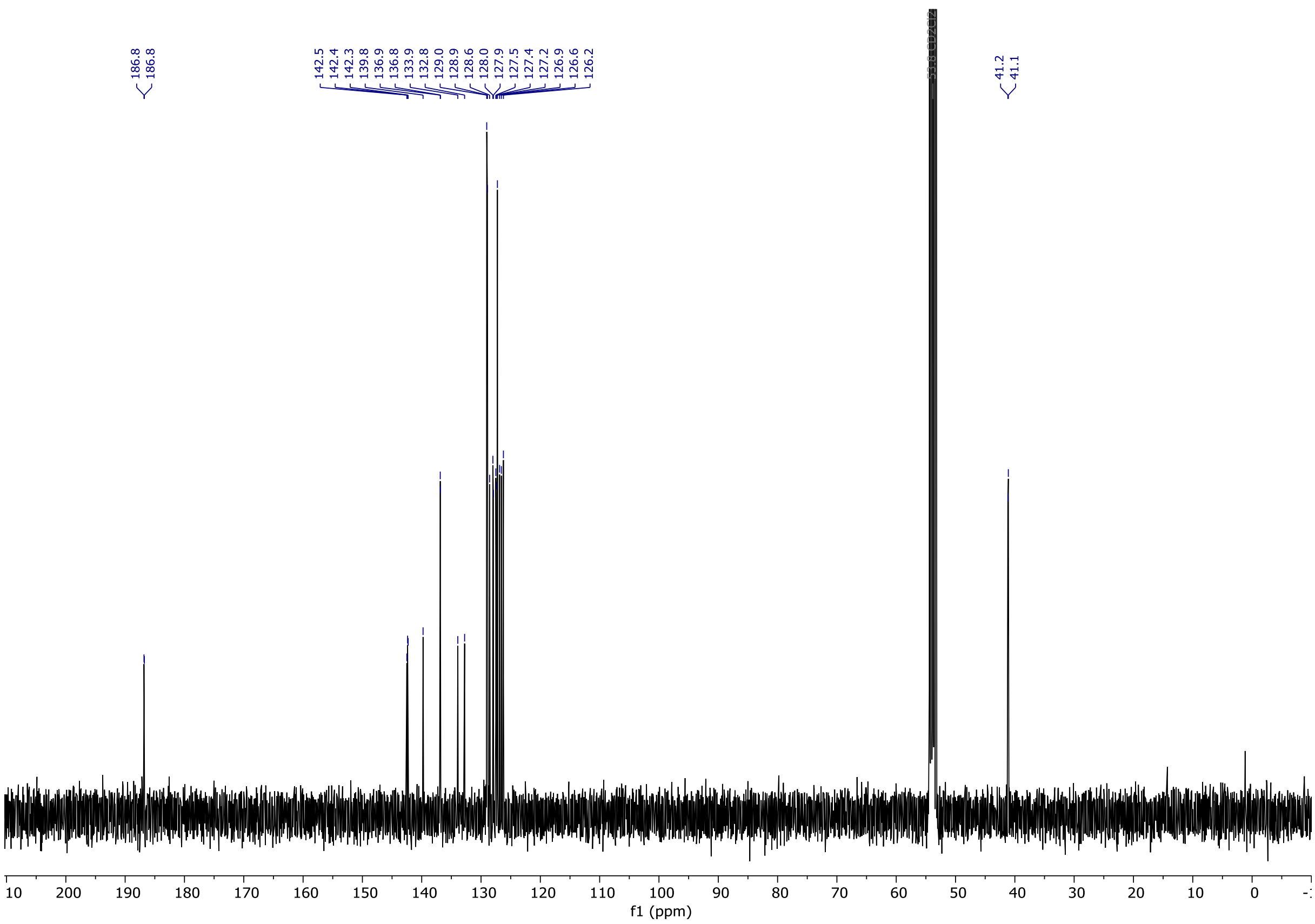

^{13}C NMR spectrum (101 MHz, CD_2Cl_2) of **4l**.

5,8-Dihydro-5-phenyl-8-(1-pyrenyl)naphthalene-1,4-dione (**4m**)

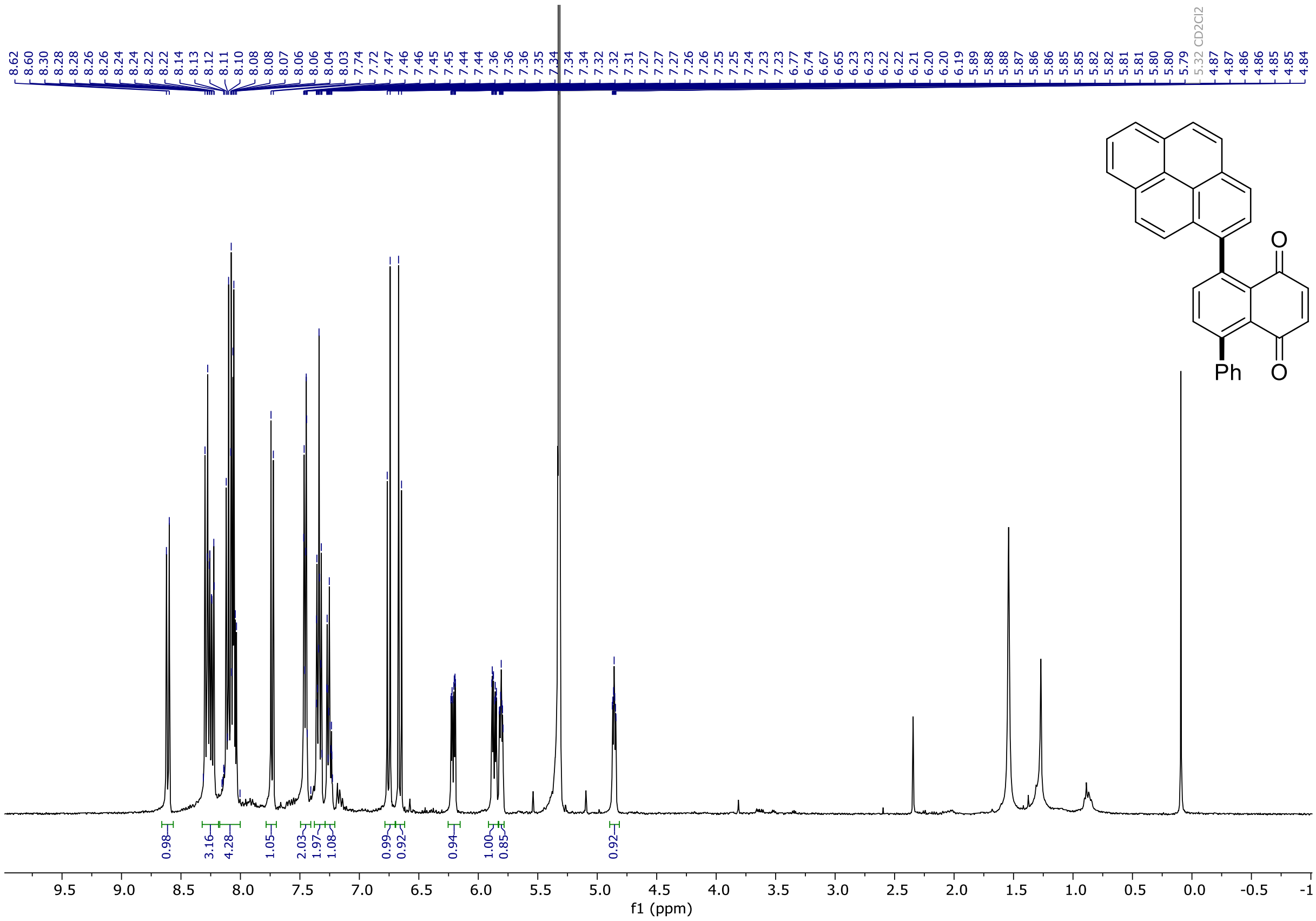

^{1}H NMR spectrum (400 MHz, CD_2Cl_2) of **4m**.

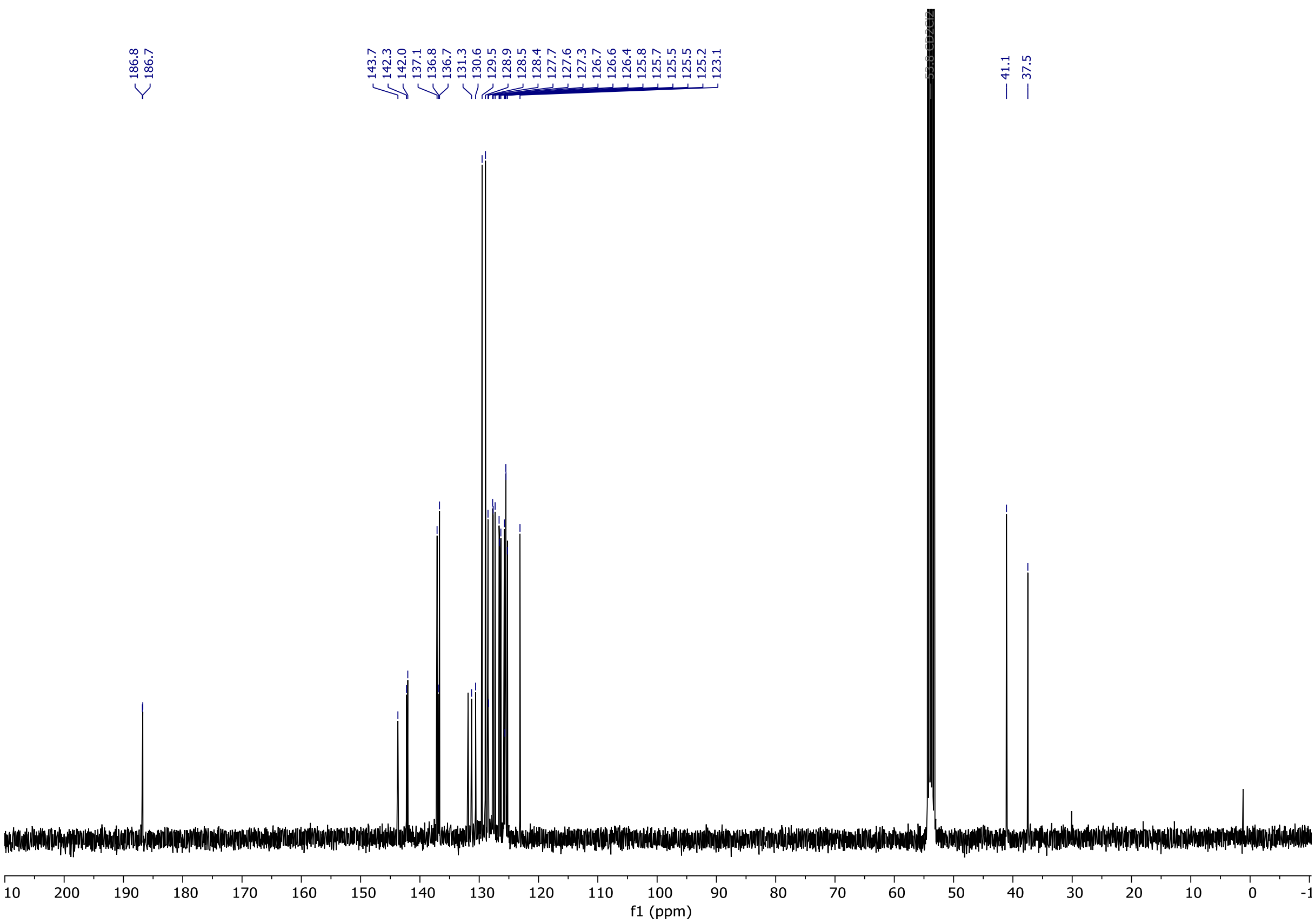

^{13}C NMR spectrum (101 MHz, CD_2Cl_2) of **4m**.

5,8-Dihydro-5-(2-chlorophenyl)-8-phenylnaphthalene-1,4-dione (**4n**)

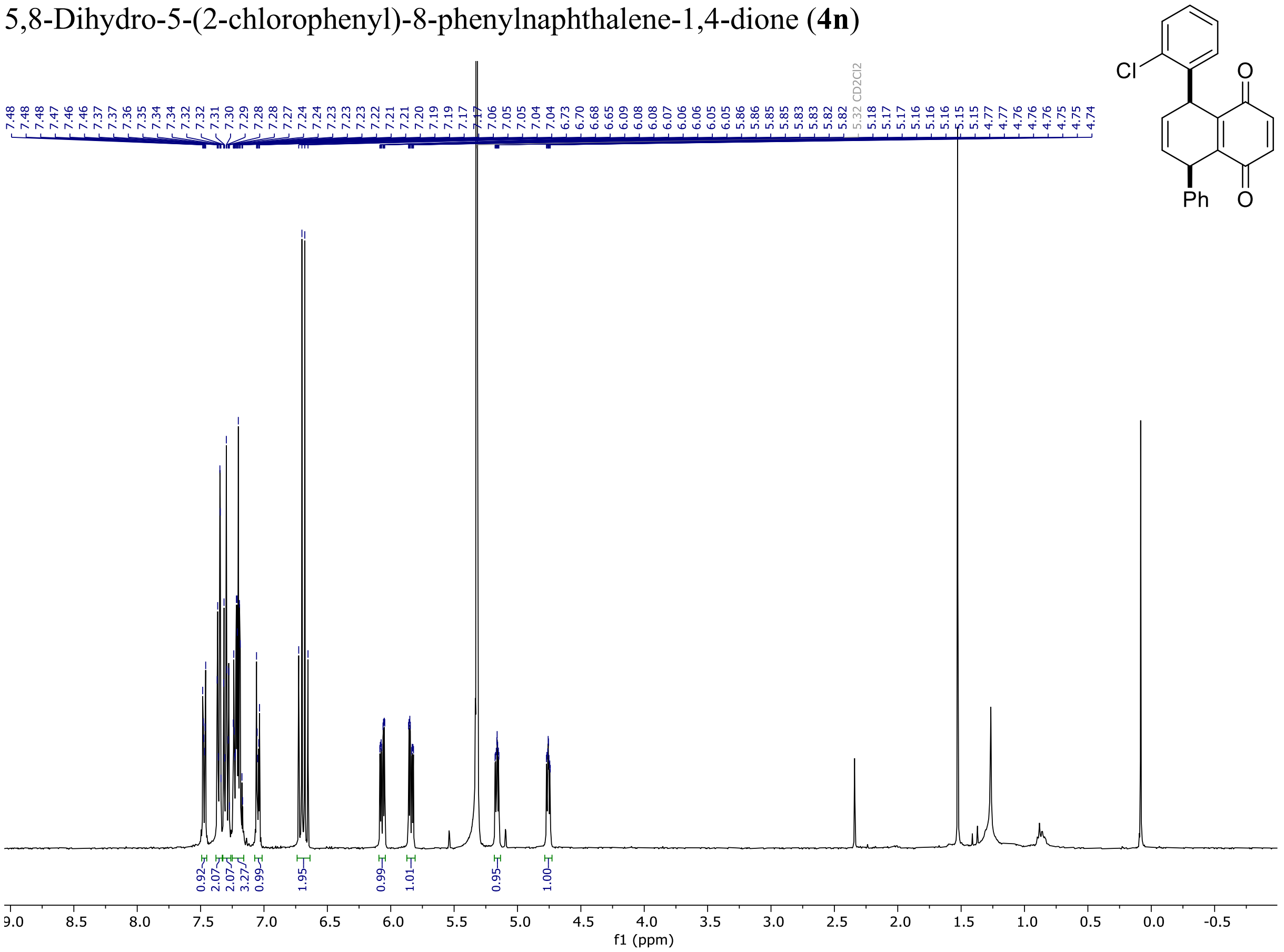

^{1}H NMR spectrum (400 MHz, CD_2Cl_2) of **4n**.

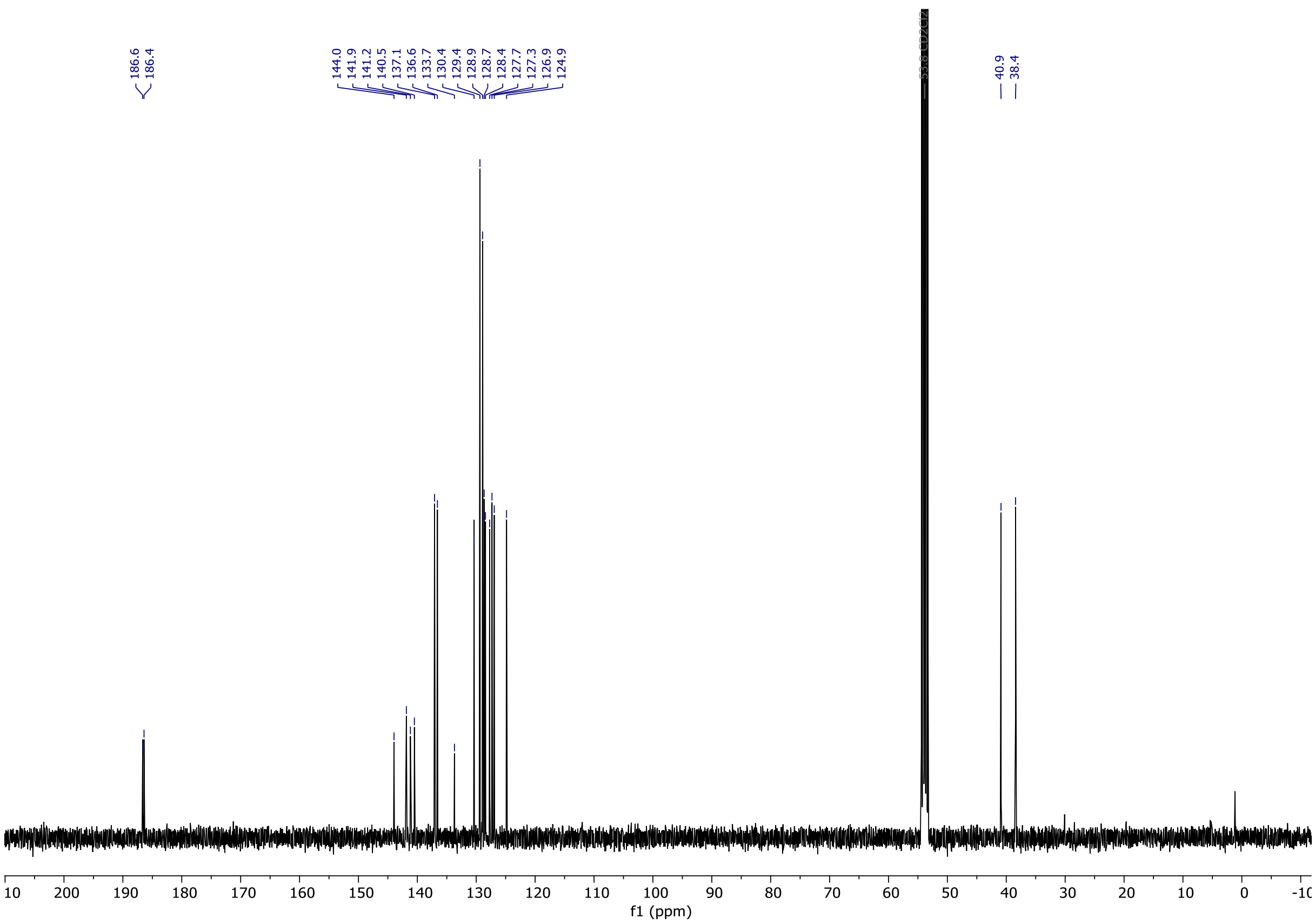

^{13}C NMR spectrum (101 MHz, CD_2Cl_2) of **4n**.

5,8-Dihydro-5-(4-chlorophenyl)-8-phenylnaphthalene-1,4-dione (**4o**)

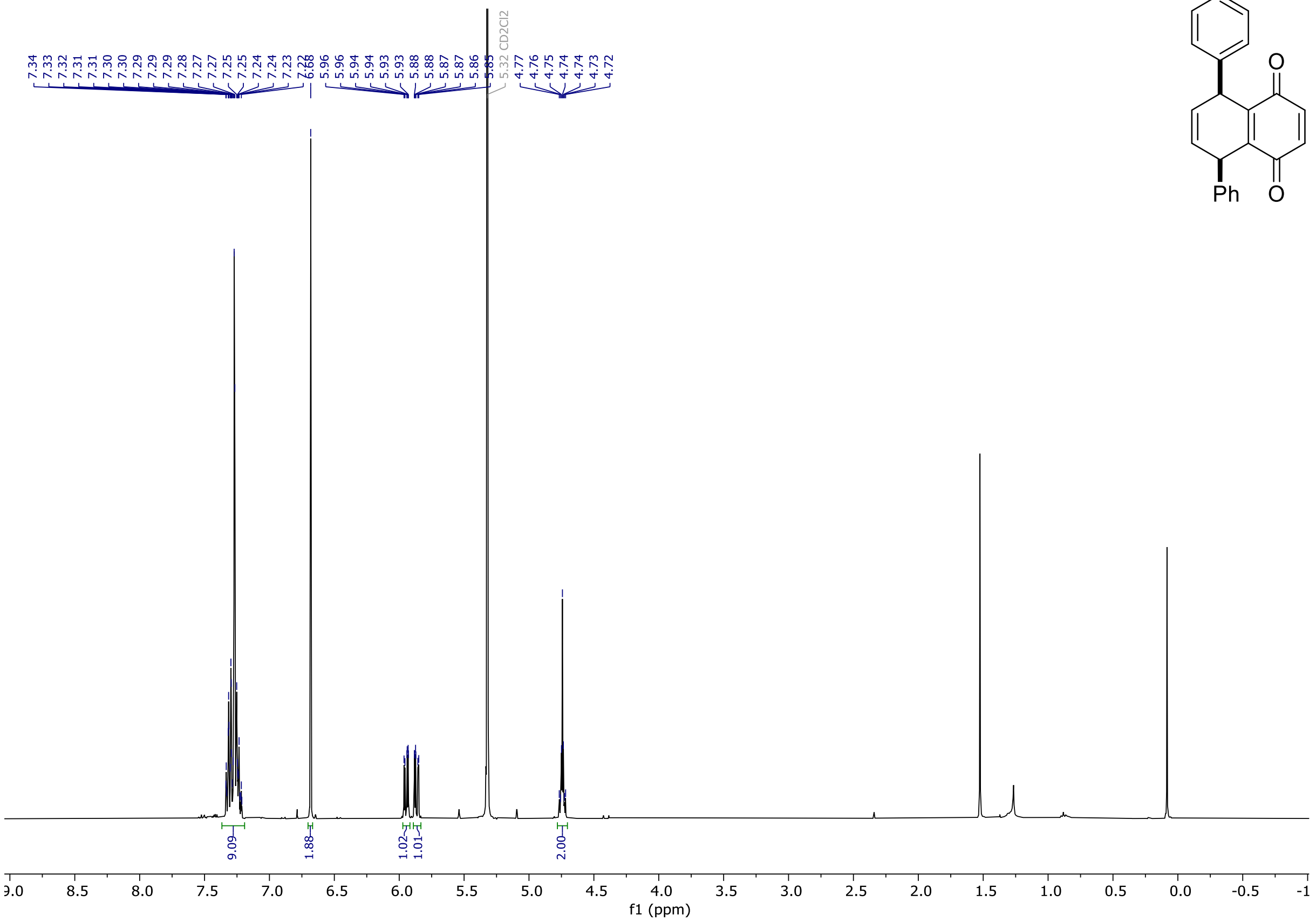

^{1}H NMR spectrum (400 MHz, CD_2Cl_2) of **4o**.

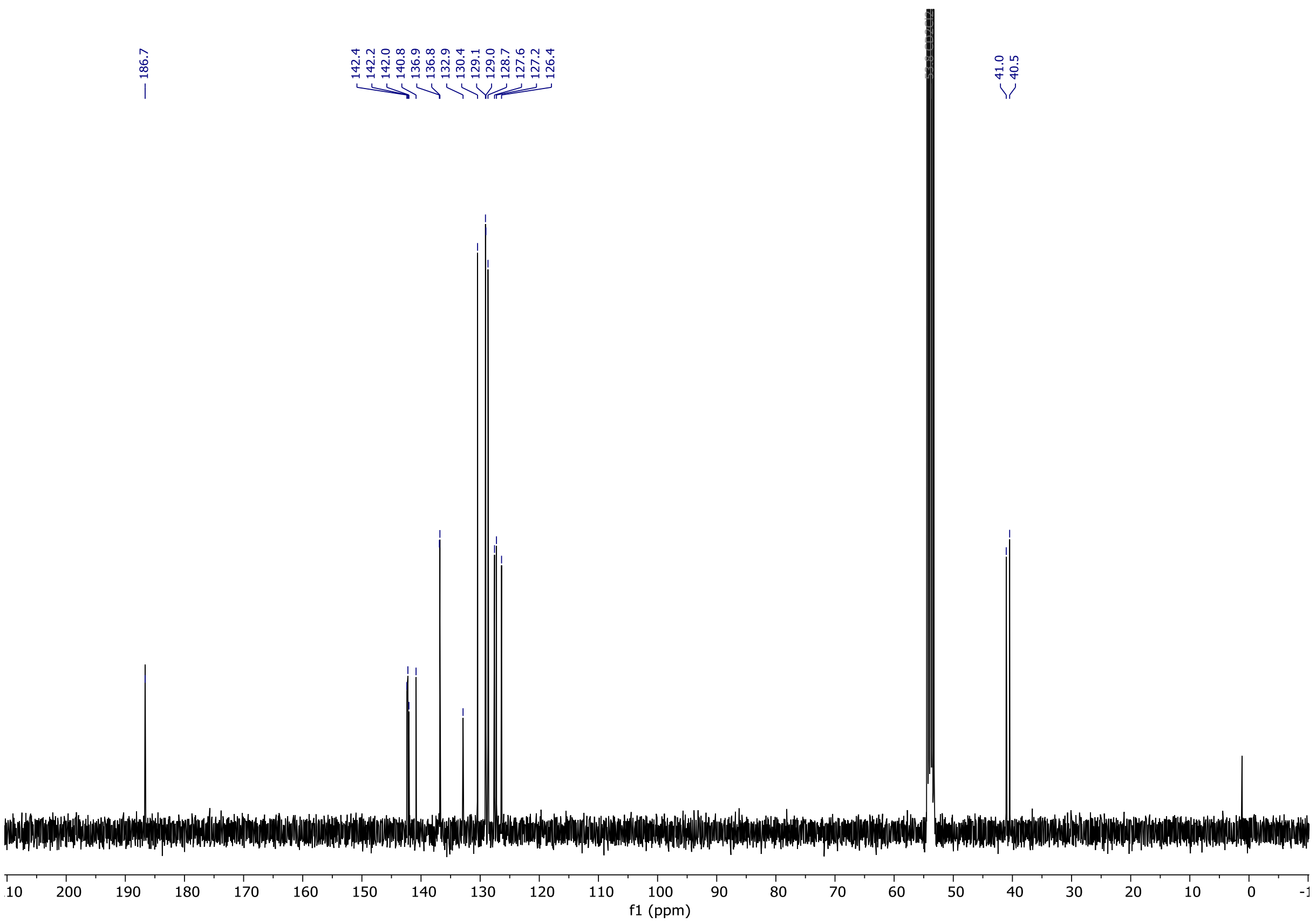

^{13}C NMR spectrum (101 MHz, CD_2Cl_2) of **4o**.

5,8-Dihydro-5-(2-bromophenyl)-8-phenylnaphthalene-1,4-dione (**4p**)

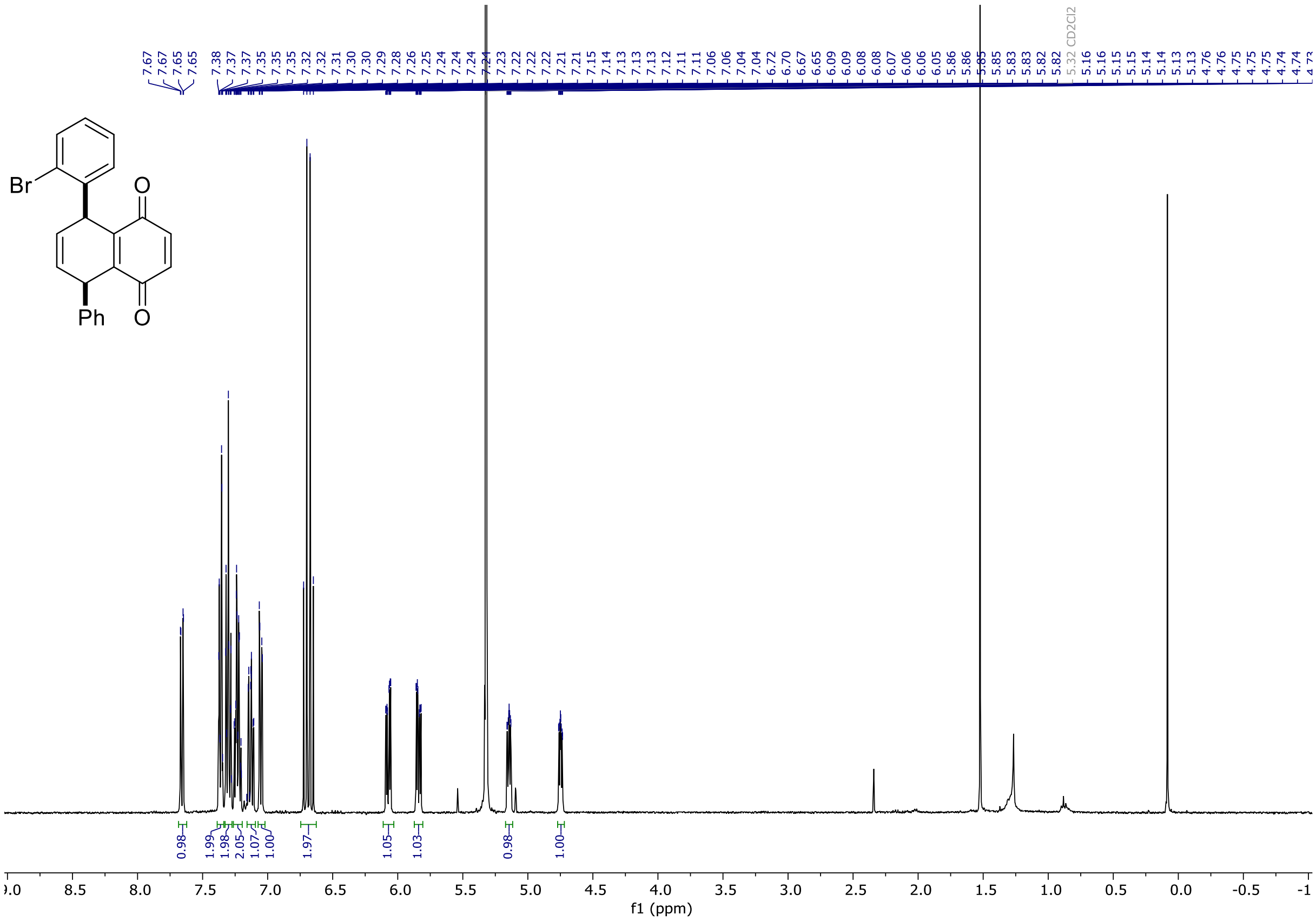

^{1}H NMR spectrum (400 MHz, CD_2Cl_2) of **4p**.

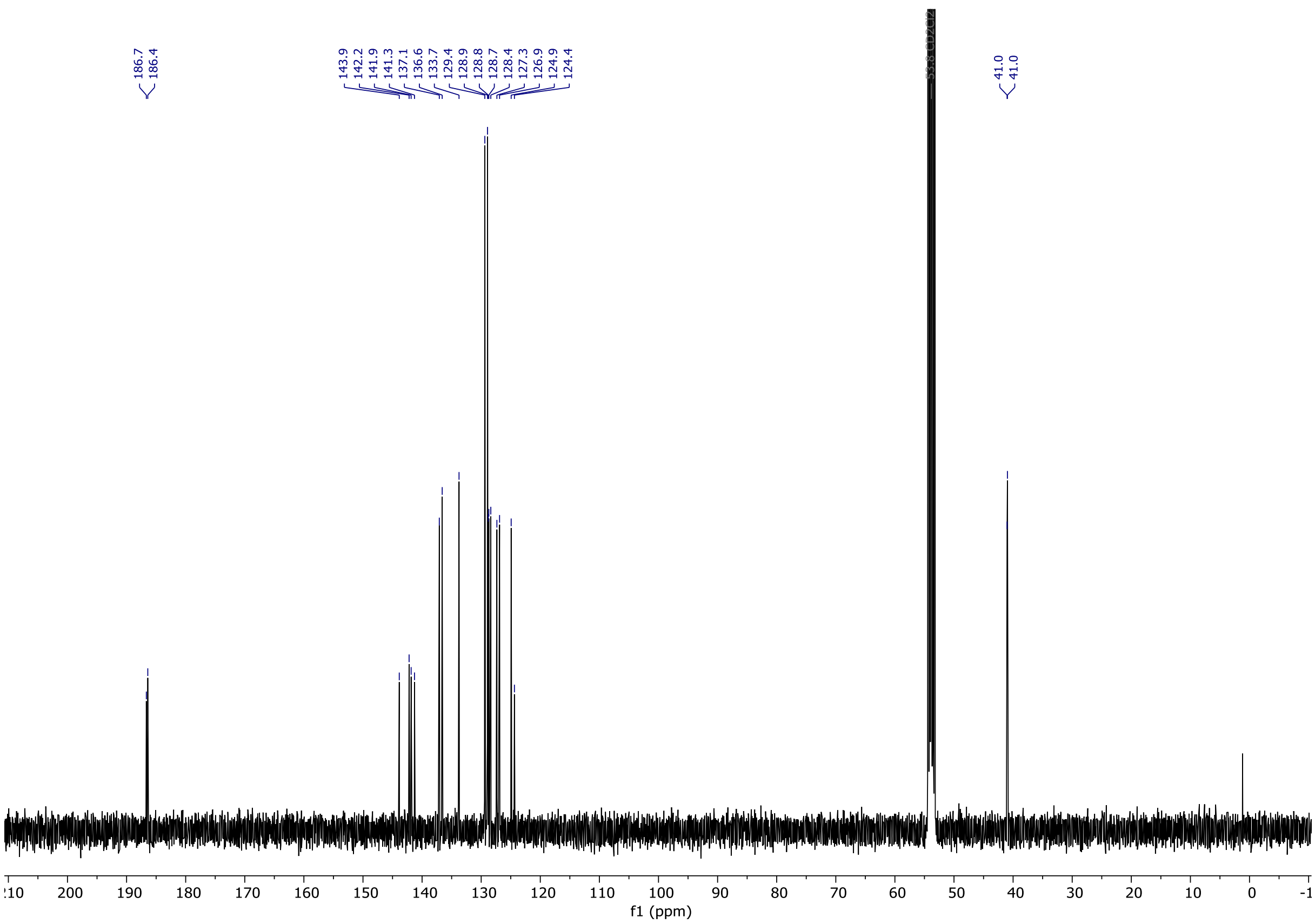

^{13}C NMR spectrum (101 MHz, CD_2Cl_2) of **4p**.

5,8-Dihydro-5-(3-bromophenyl)-8-phenylnaphthalene-1,4-dione (**4q**)

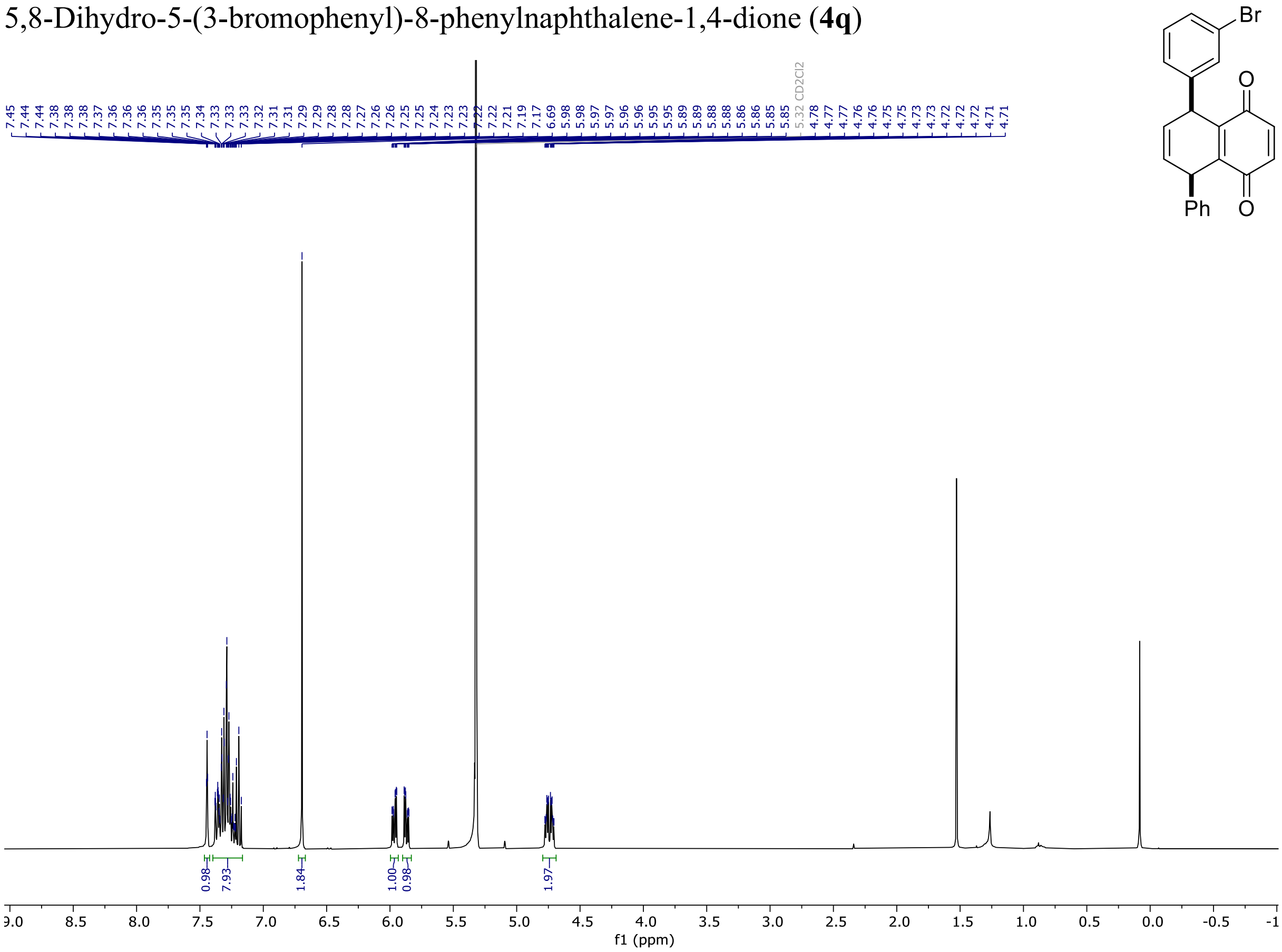

^{1}H NMR spectrum (400 MHz, CD_2Cl_2) of **4q**.

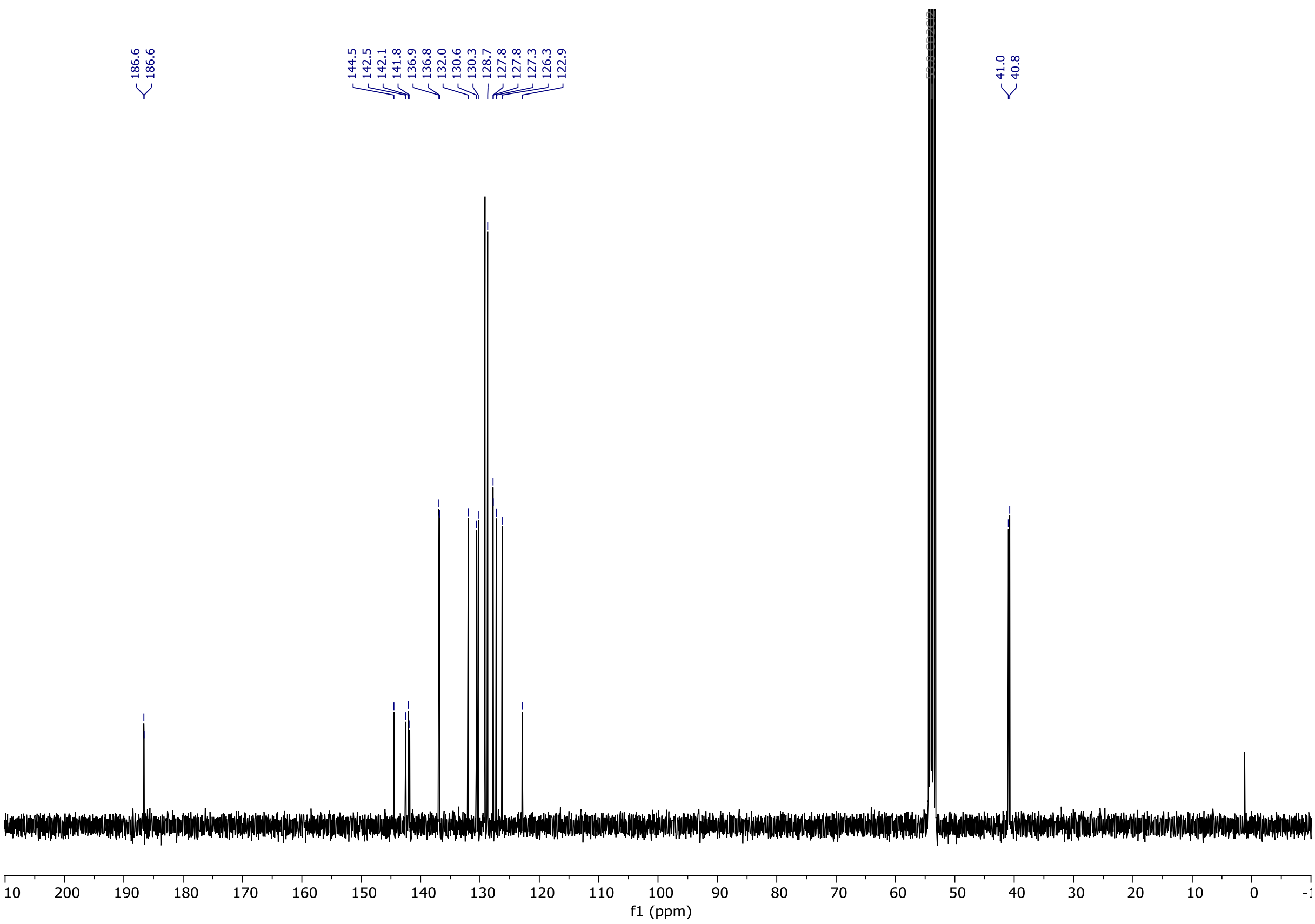

^{13}C NMR spectrum (101 MHz, CD_2Cl_2) of **4q**.

5,8-Dihydro-5-(4-bromophenyl)-8-phenylnaphthalene-1,4-dione (**4r**)

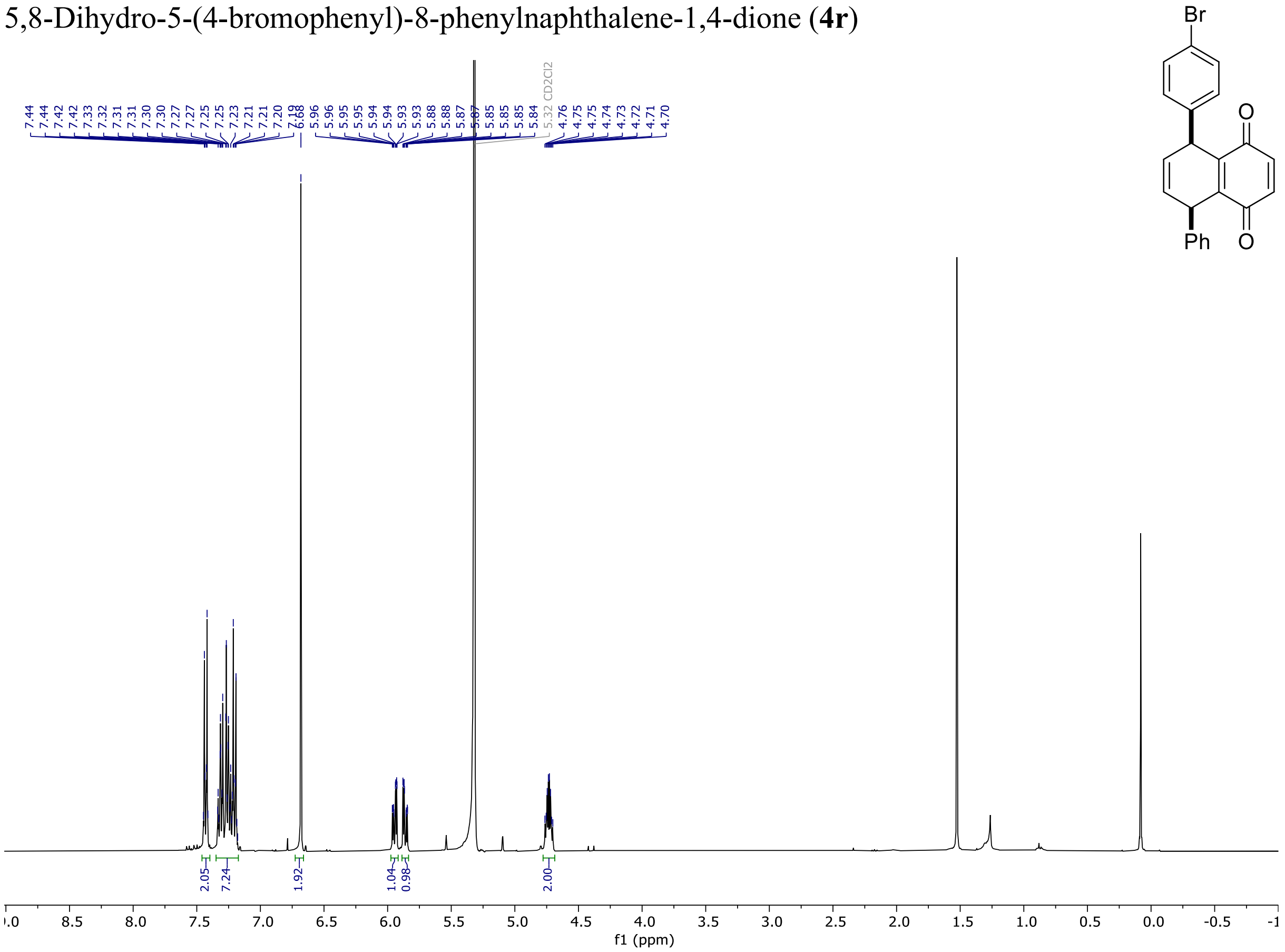

^{1}H NMR spectrum (400 MHz, CD_2Cl_2) of **4r**.

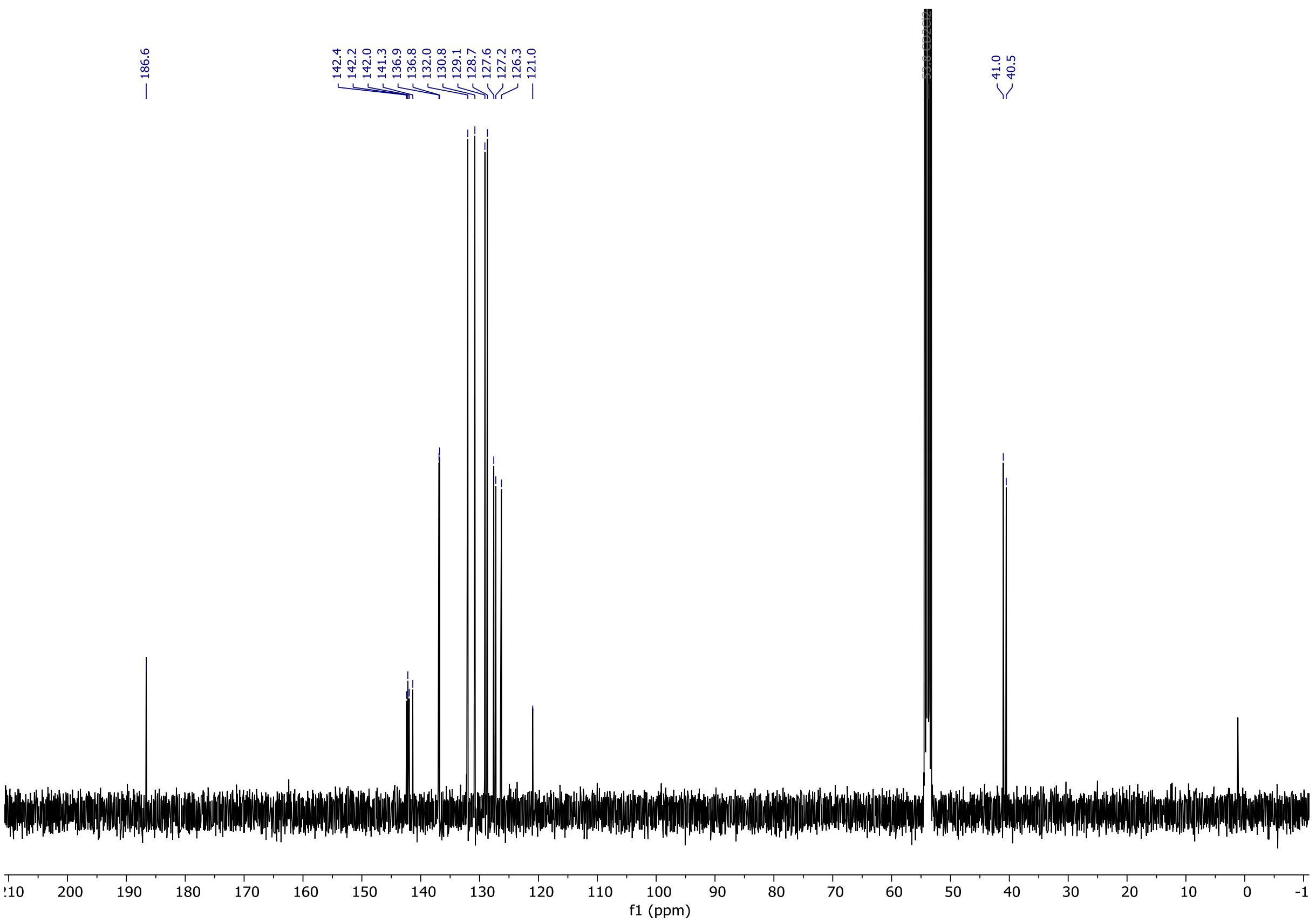

^{13}C NMR spectrum (101 MHz, CD_2Cl_2) of **4r**.

5,8-Dihydro-5-(4-iodophenyl)-8-phenylnaphthalene-1,4-dione (**4s**)

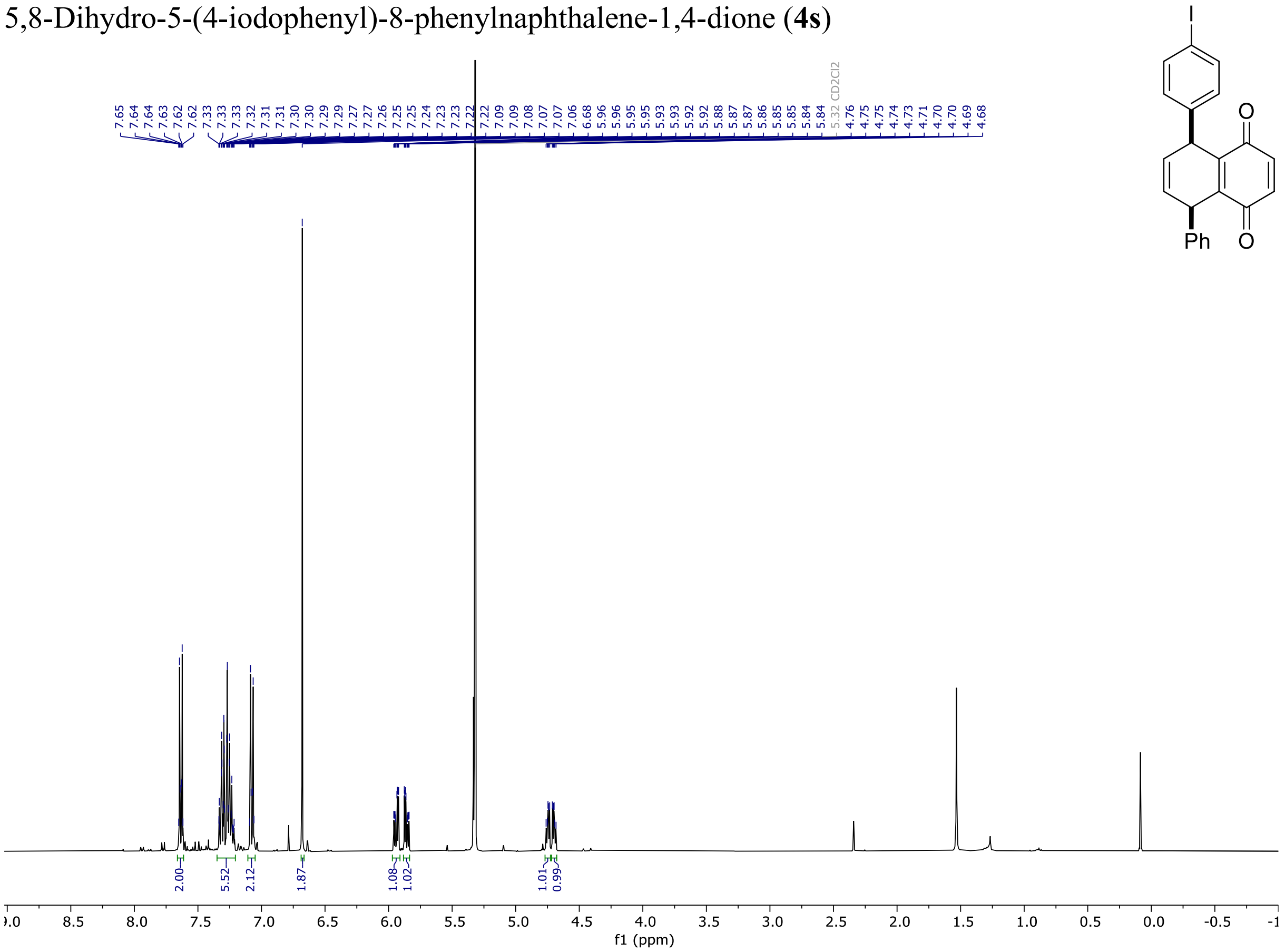

^{1}H NMR spectrum (400 MHz, CD_2Cl_2) of **4s**.

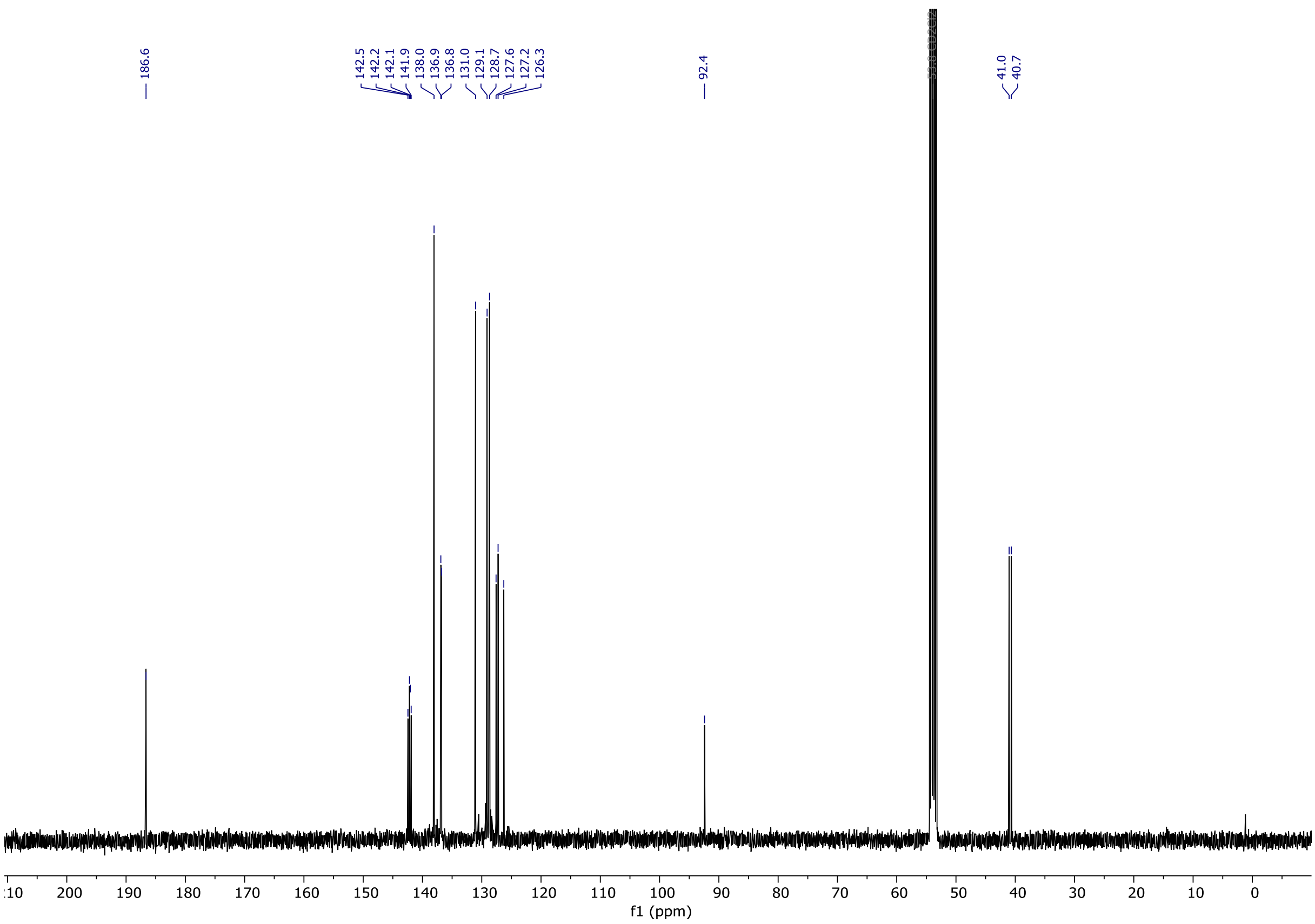

^{13}C NMR spectrum (101 MHz, CD_2Cl_2) of **4s**.

5,8-Dihydro-5-(4-trifluoromethylphenyl)-8-phenylnaphthalene-1,4-dione (**4t**)

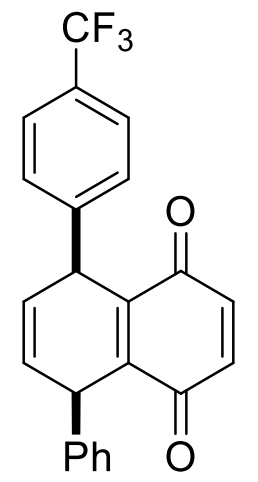

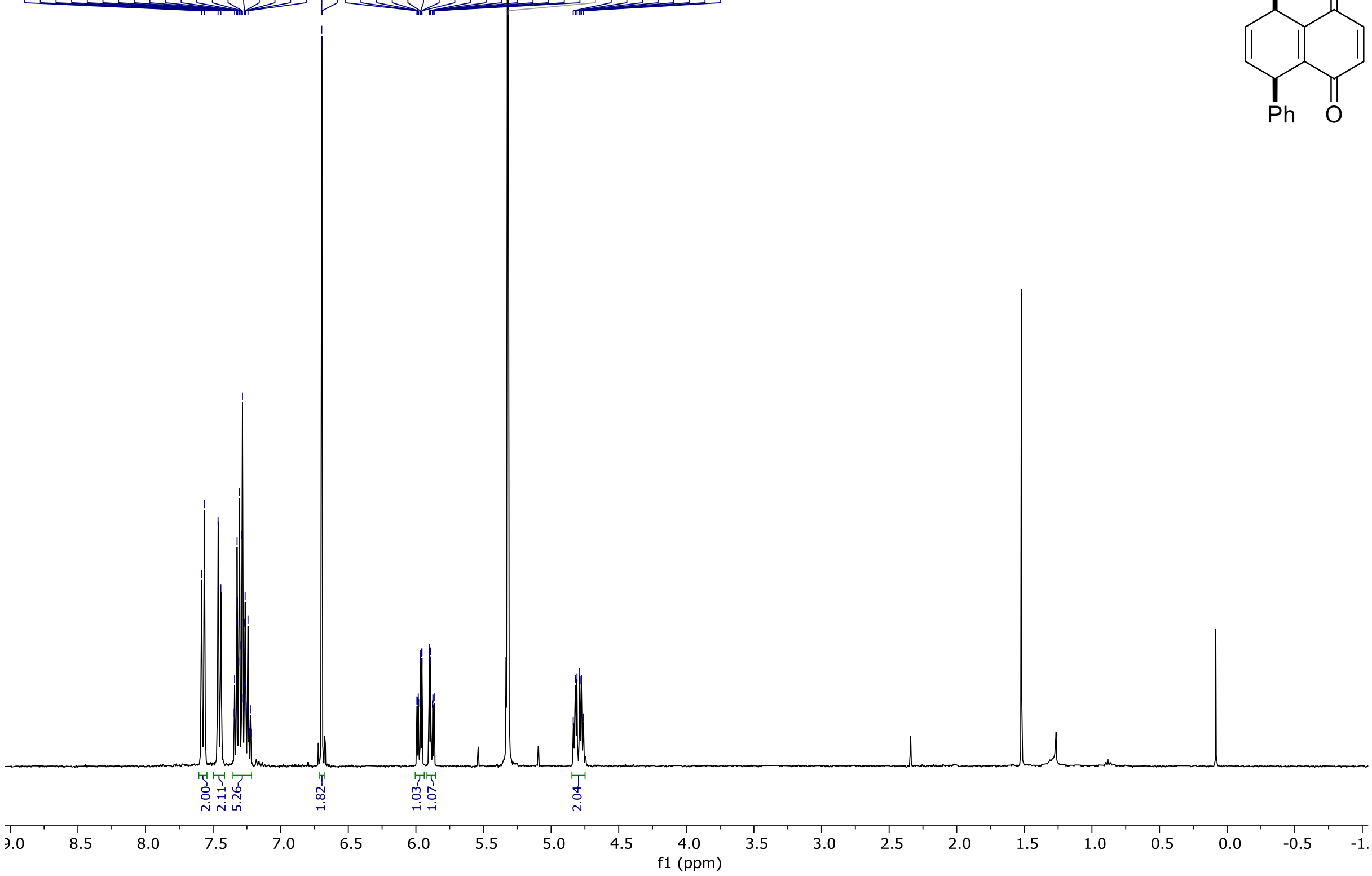

^{1}H NMR spectrum (400 MHz, CD_2Cl_2) of **4t**.

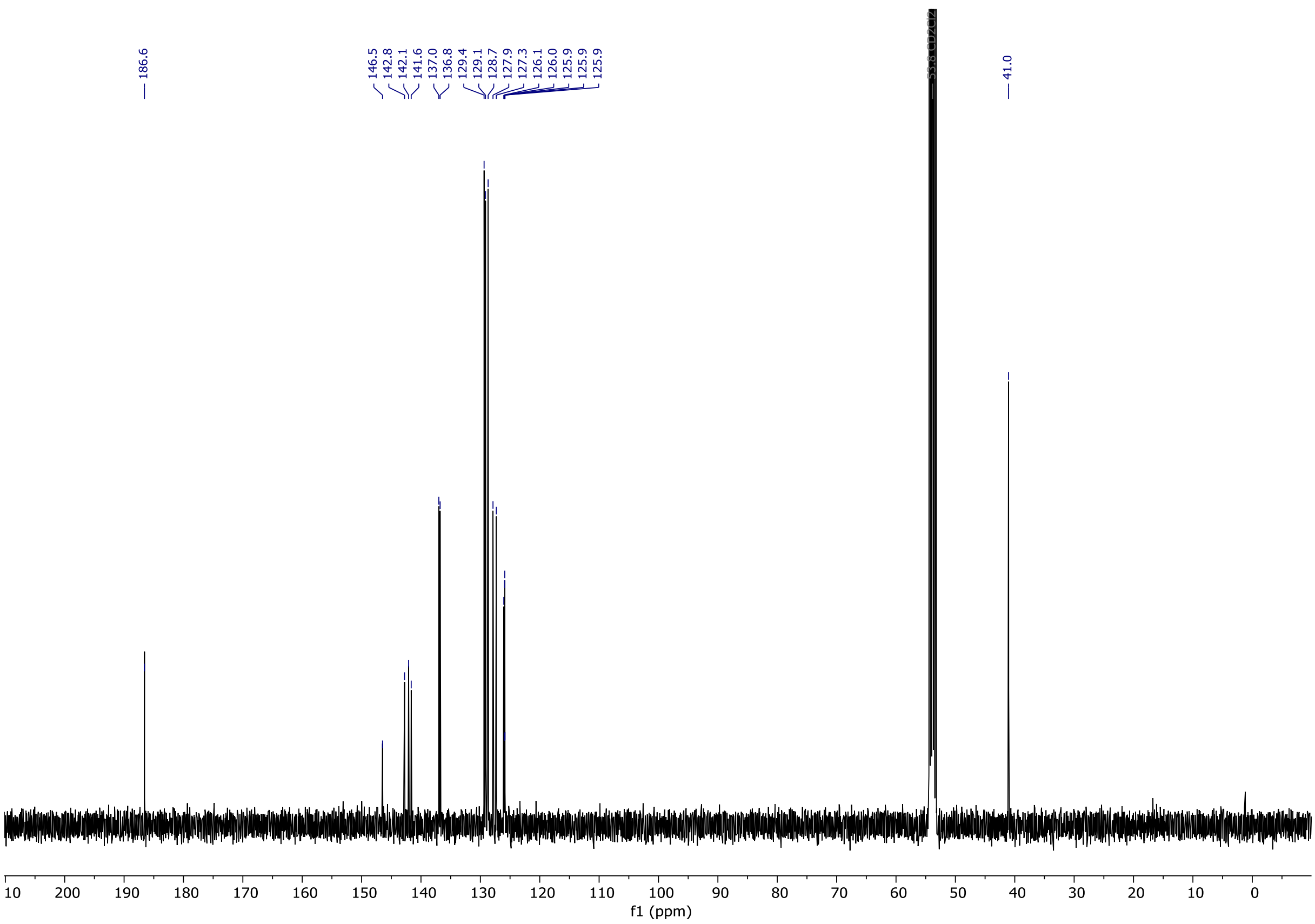

^{13}C NMR spectrum (101 MHz, CD_2Cl_2) of **4t**.

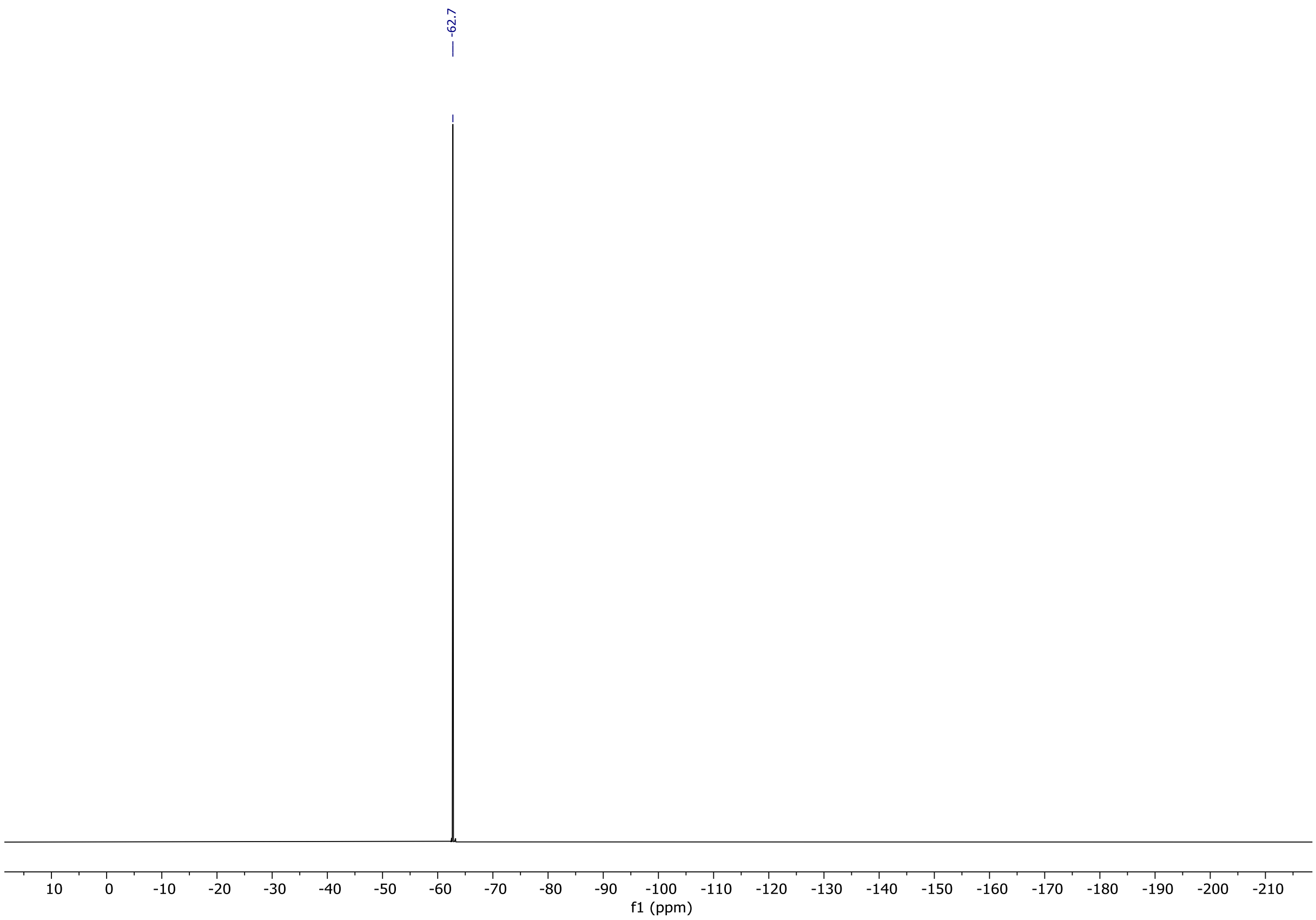

^{19}F NMR spectrum (344 MHz, CD_2Cl_2) of **4t**.

5,8-Dihydro-5-(4-methyl benzoate)-8-phenylnaphthalene-1,4-dione (**4u**)

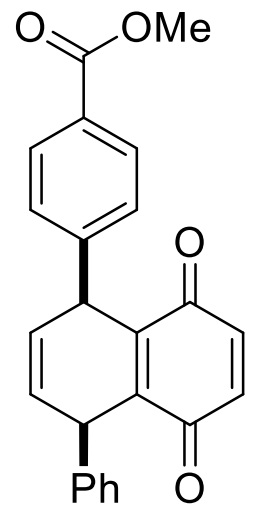

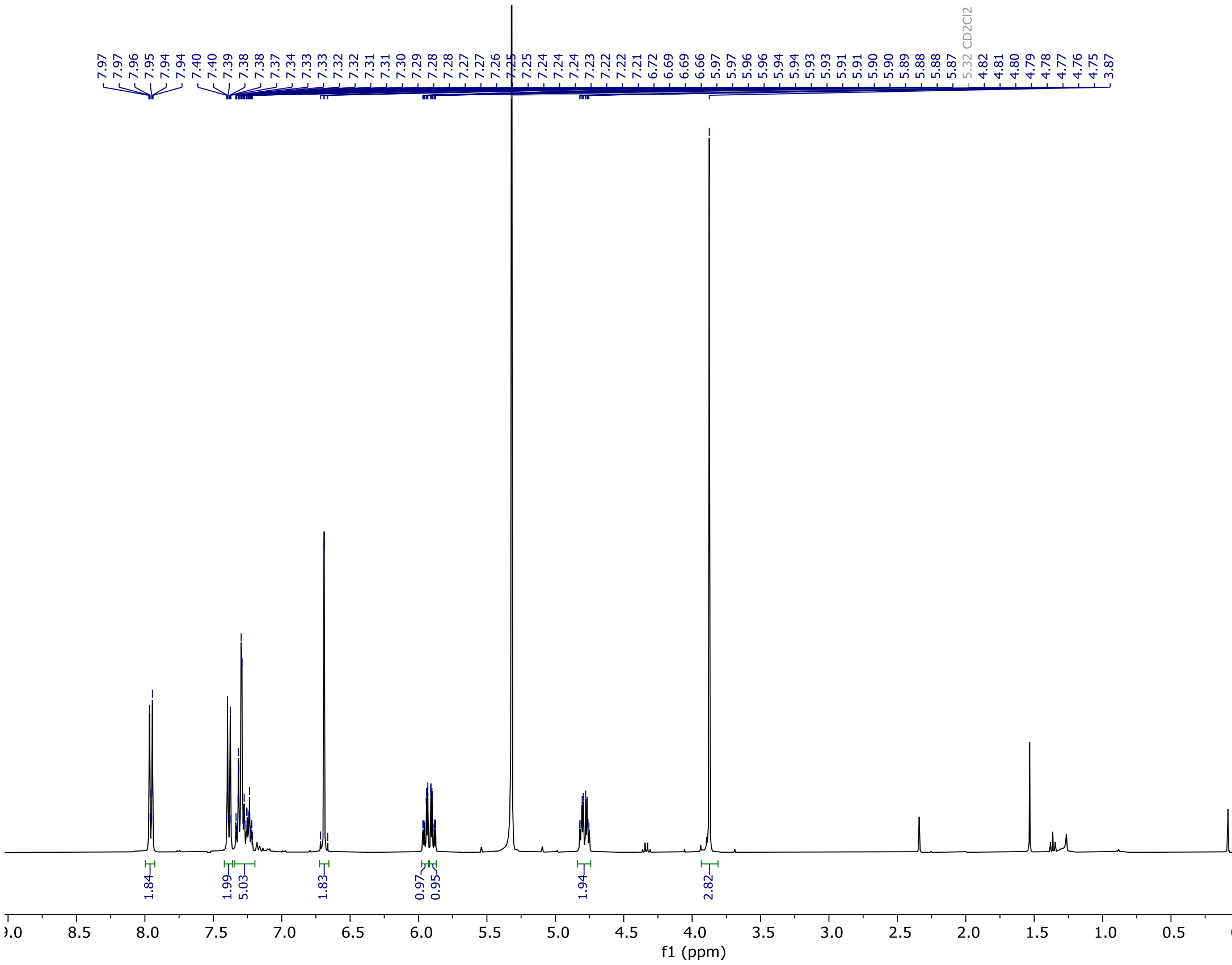

^{1}H NMR spectrum (400 MHz, CD_2Cl_2) of **4u**.

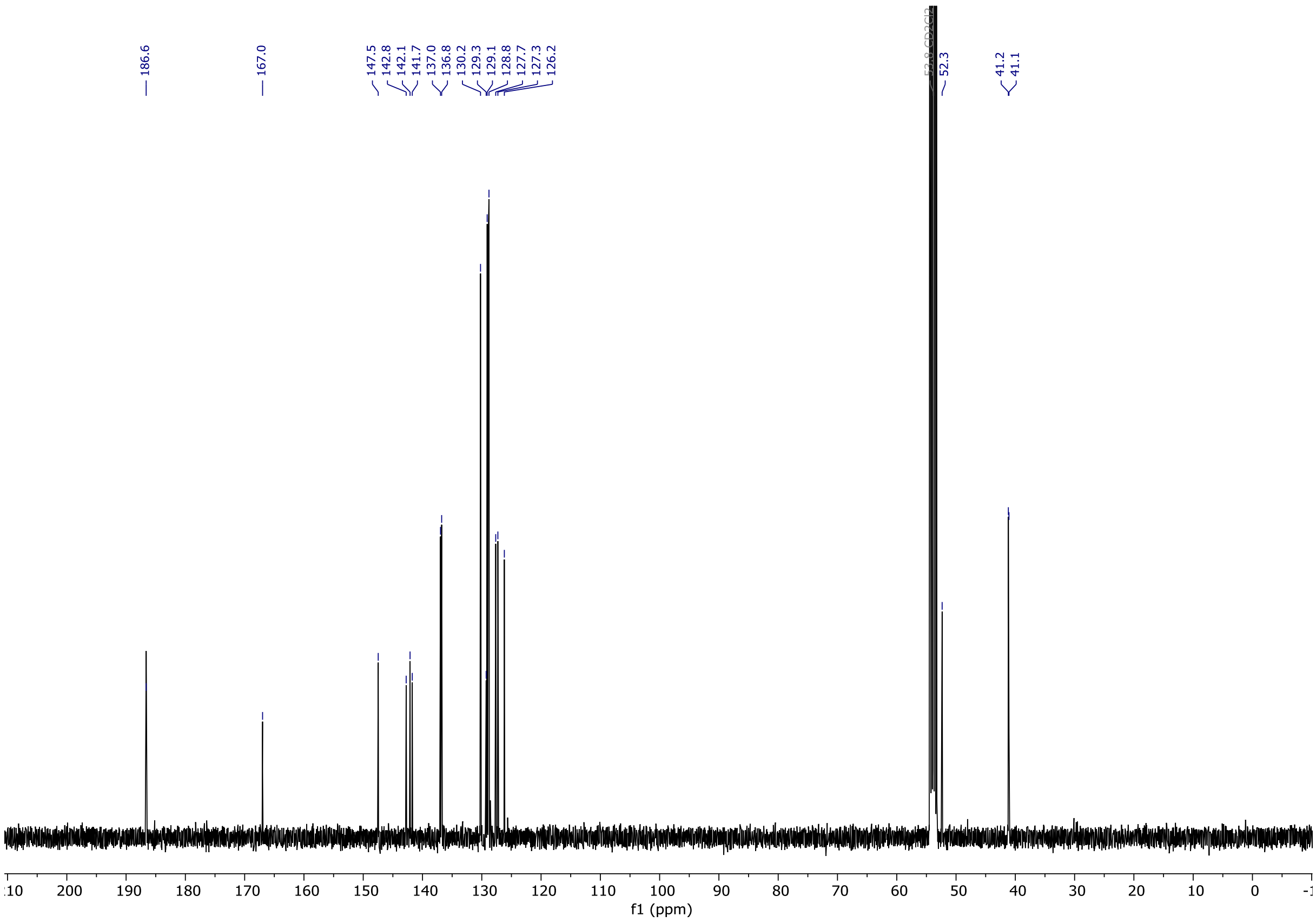

^{13}C NMR spectrum (101 MHz, CD_2Cl_2) of **4u**.

5,8-Dihydro-5-(4-cyanophenyl)-8-phenylnaphthalene-1,4-dione (**4v**)

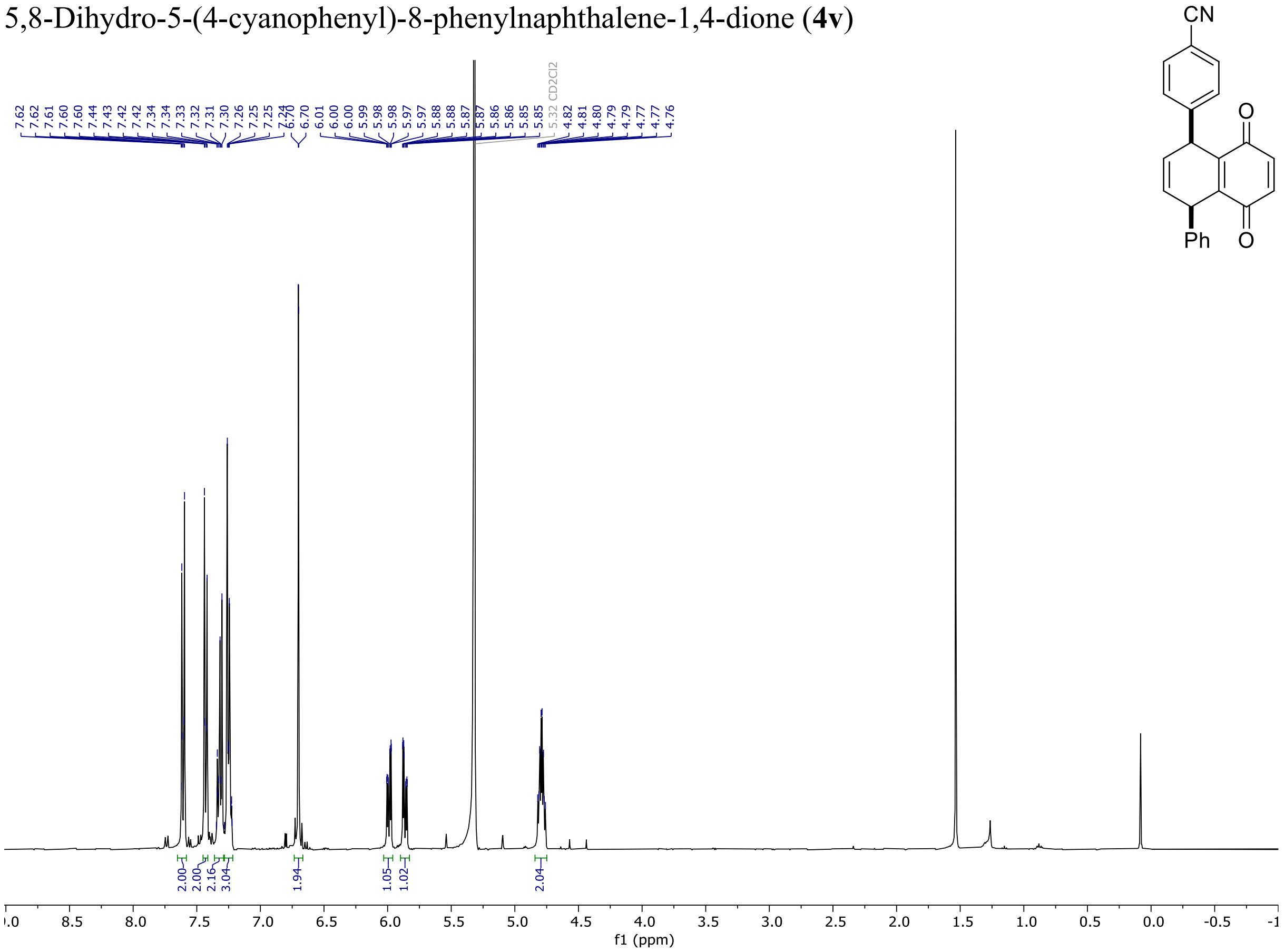

^{1}H NMR spectrum (400 MHz, CD_2Cl_2) of **4v**.

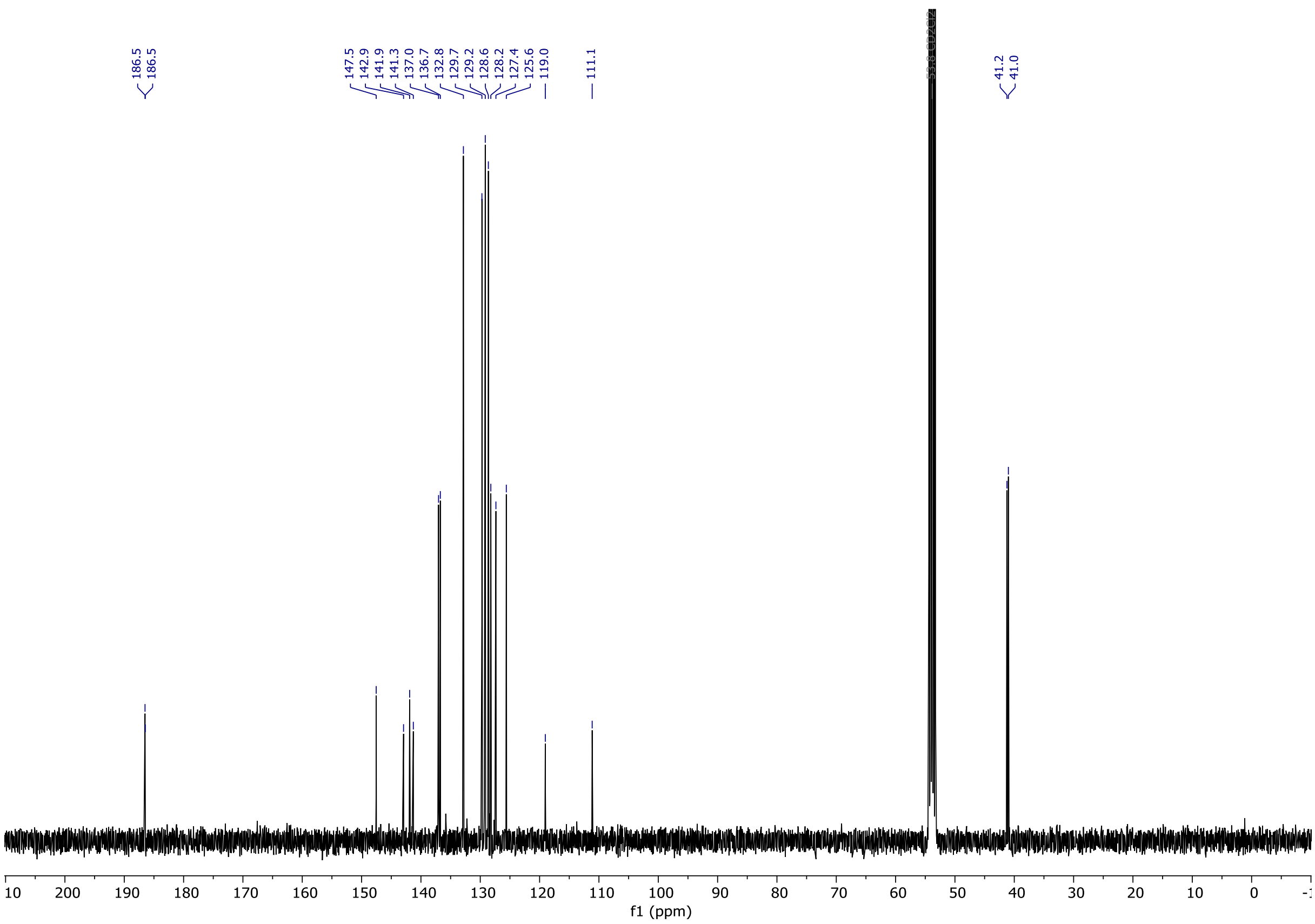

^{13}C NMR spectrum (101 MHz, CD_2Cl_2) of **4v**.

5,8-Dihydro-5-(4-nitrophenyl)-8-phenylnaphthalene-1,4-dione (**4w**)

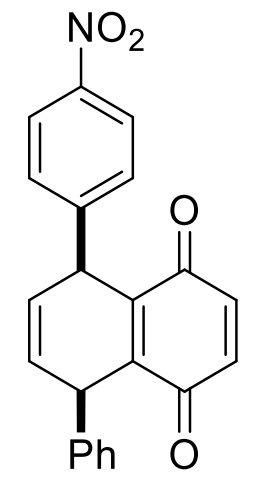

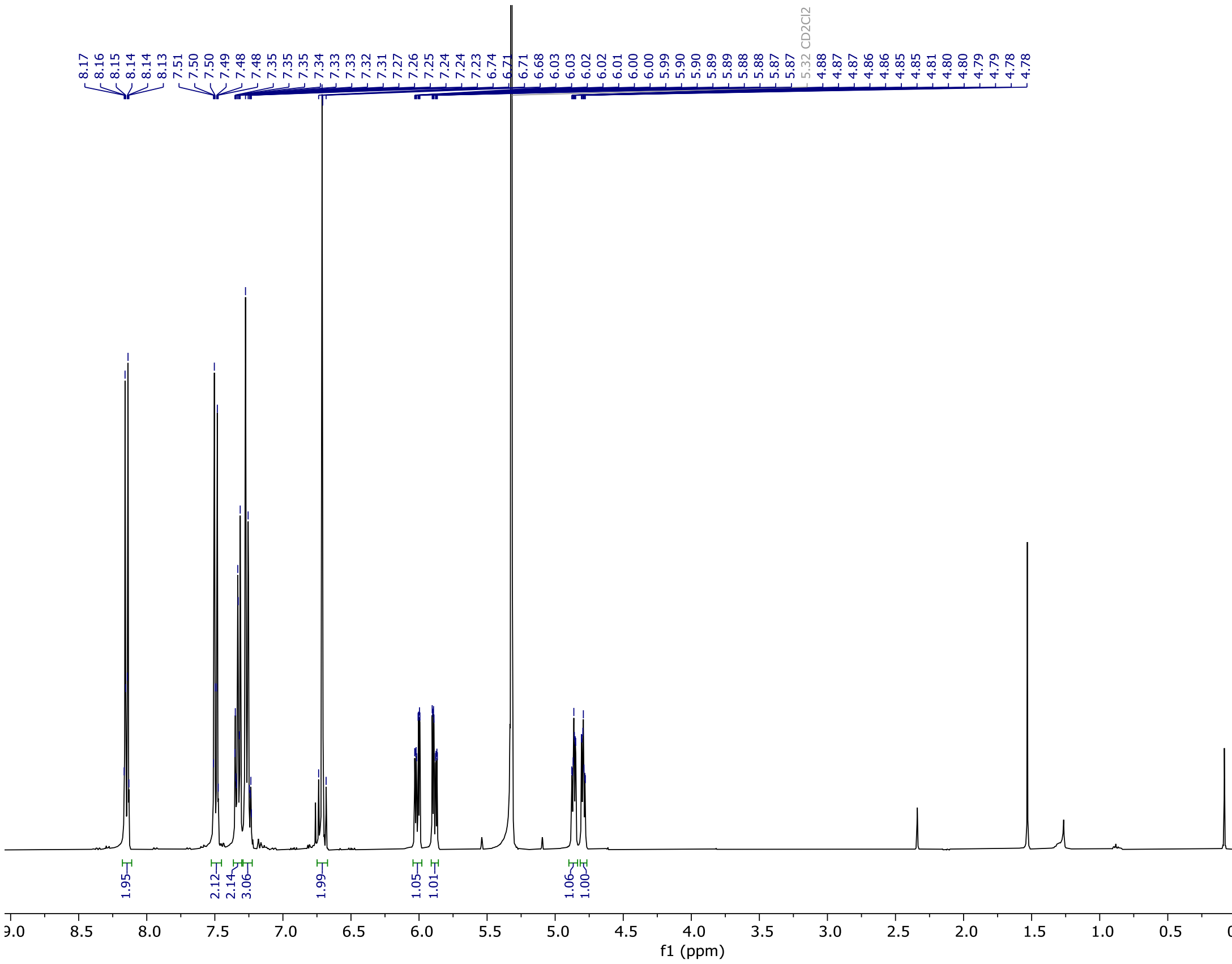

^{1}H NMR spectrum (400 MHz, CD_2Cl_2) of **4w**.

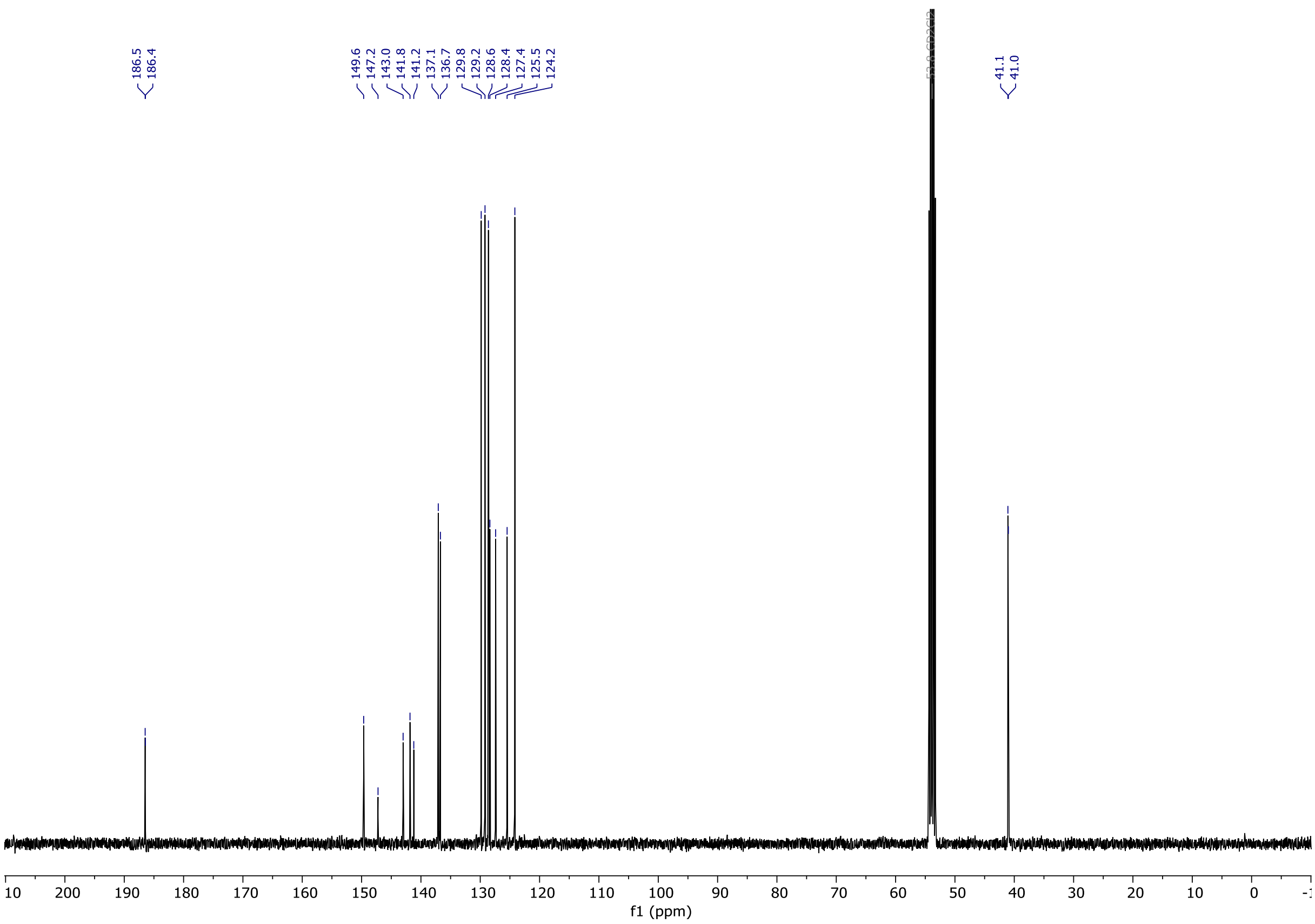

^{13}C NMR spectrum (101 MHz, CD_2Cl_2) of **4w**.

5,8-Dihydro-5-ethyl-8-phenylnaphthalene-1,4-dione (**4x**)

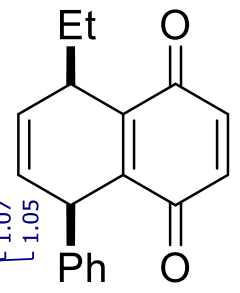

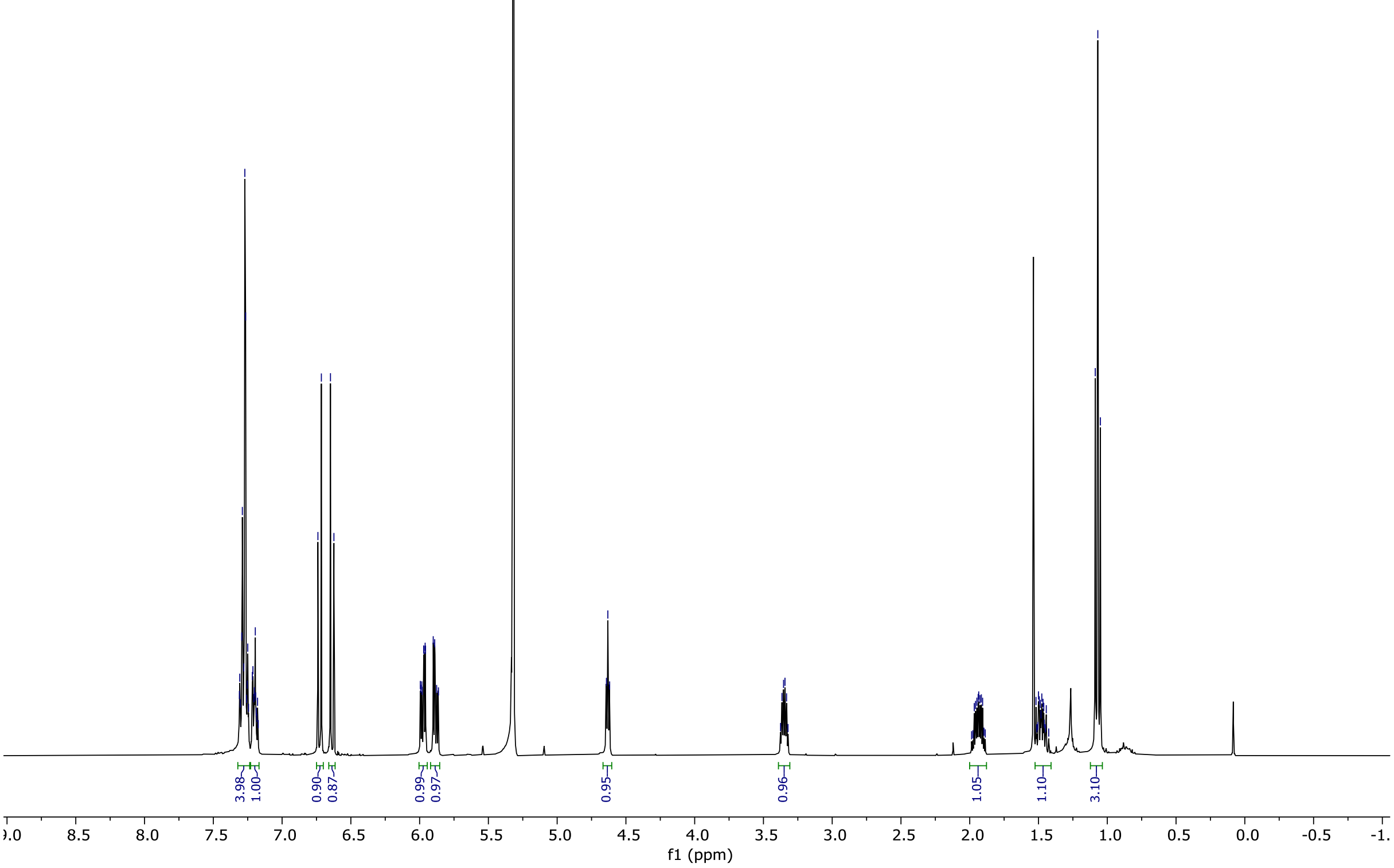

^{1}H NMR spectrum (400 MHz, CD_2Cl_2) of **4x**.

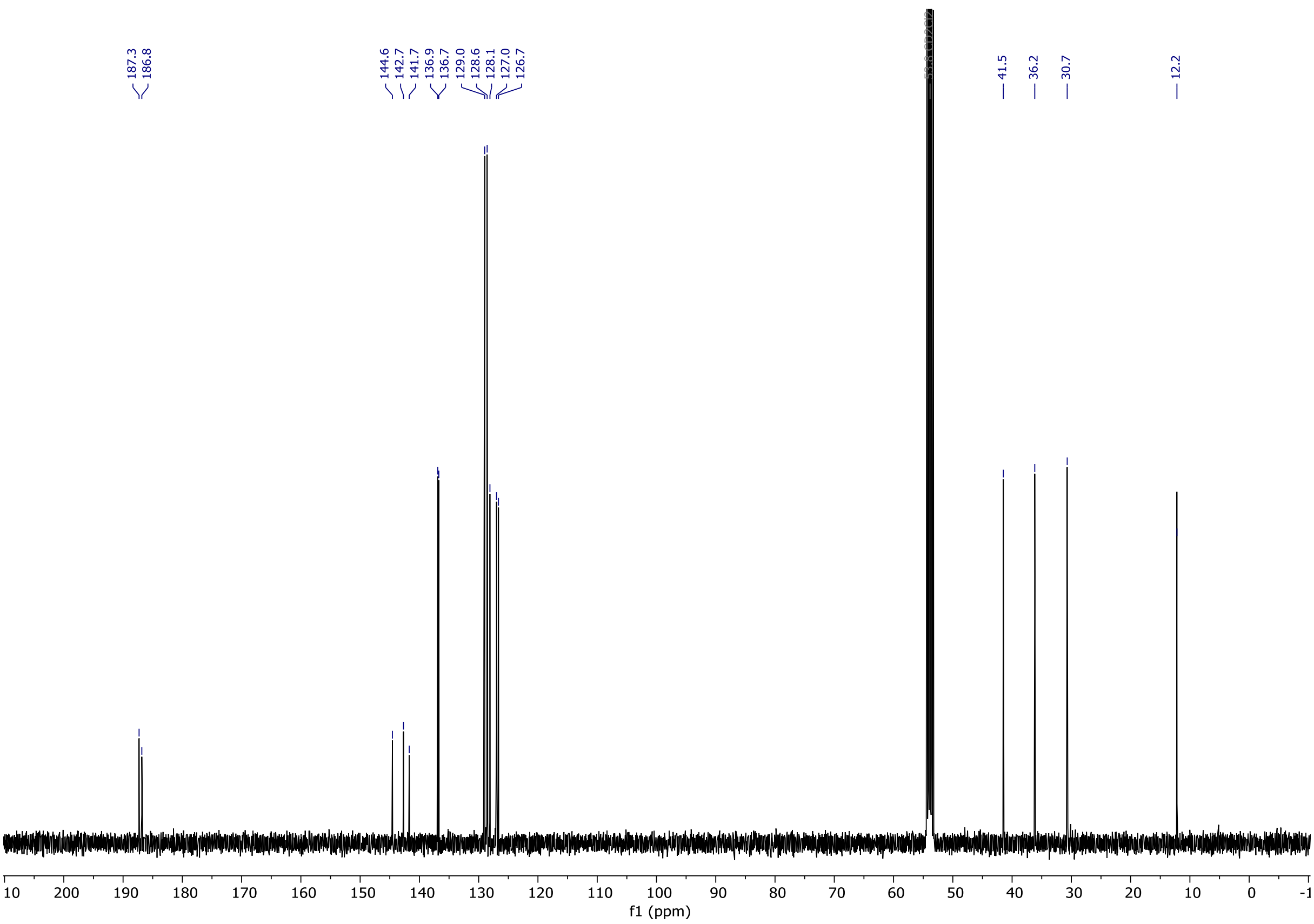

^{13}C NMR spectrum (101 MHz, CD_2Cl_2) of **4x**.

5,8-Dihydro-5-cyclohexyl-8-phenylnaphthalene-1,4-dione (**4y**)

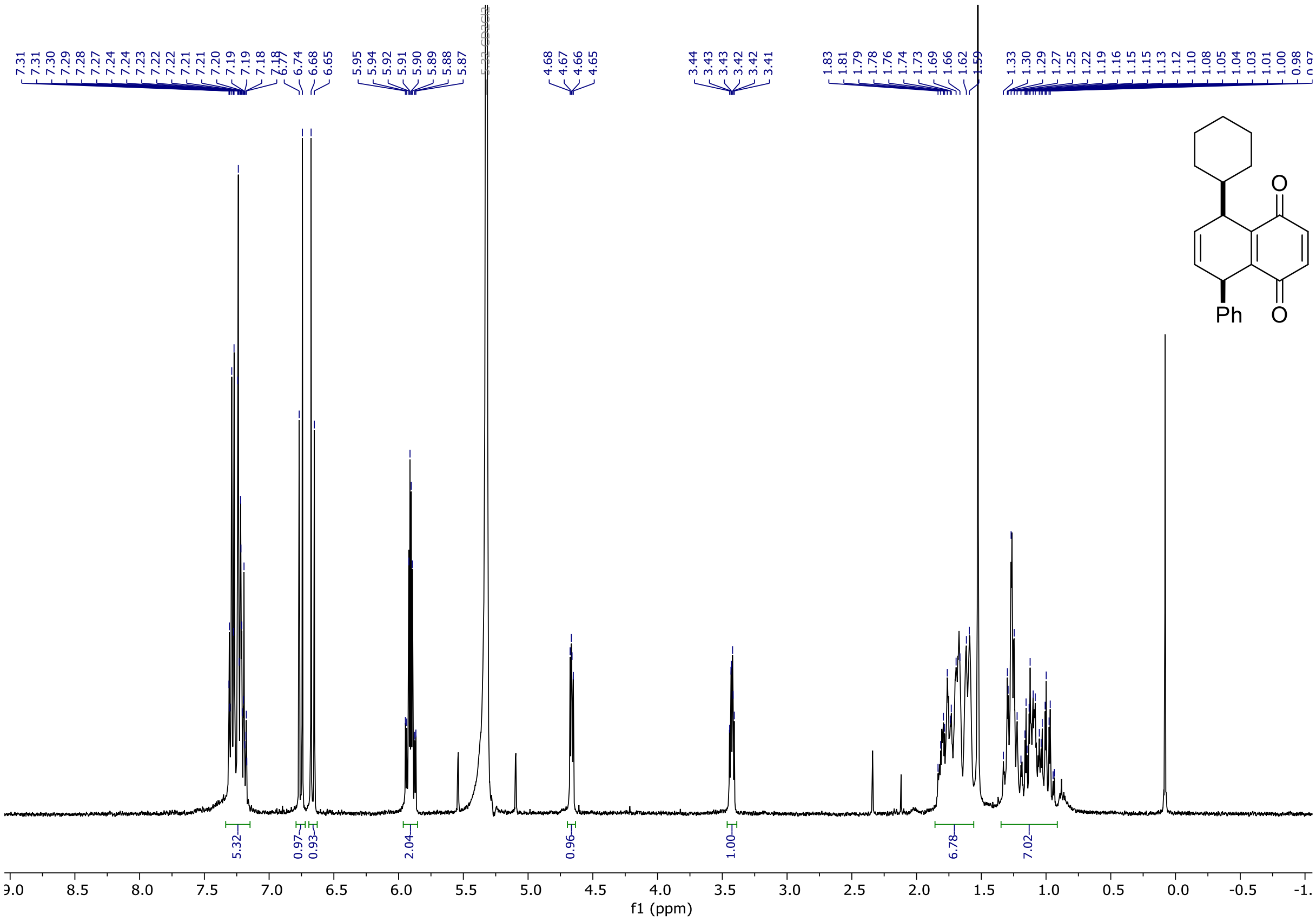

^{1}H NMR spectrum (400 MHz, CD_2Cl_2) of **4y**.

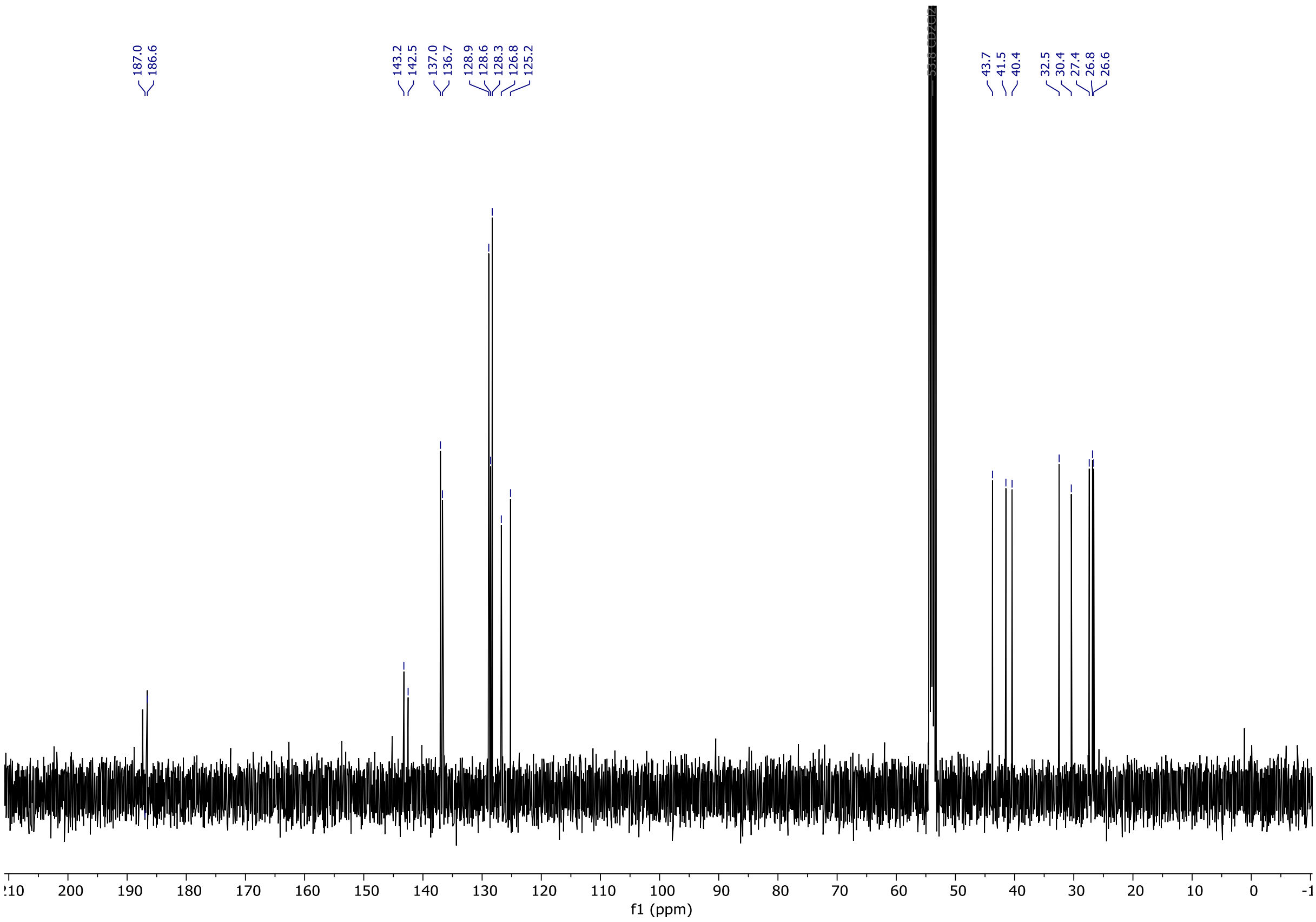

^{13}C NMR spectrum (101 MHz, CD_2Cl_2) of **4y**.

5,8-Dihydro-5,8-di(4-methylphenyl)naphthalene-1,4-dione (**4z**)

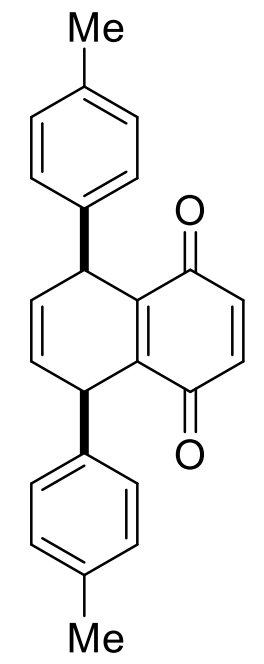

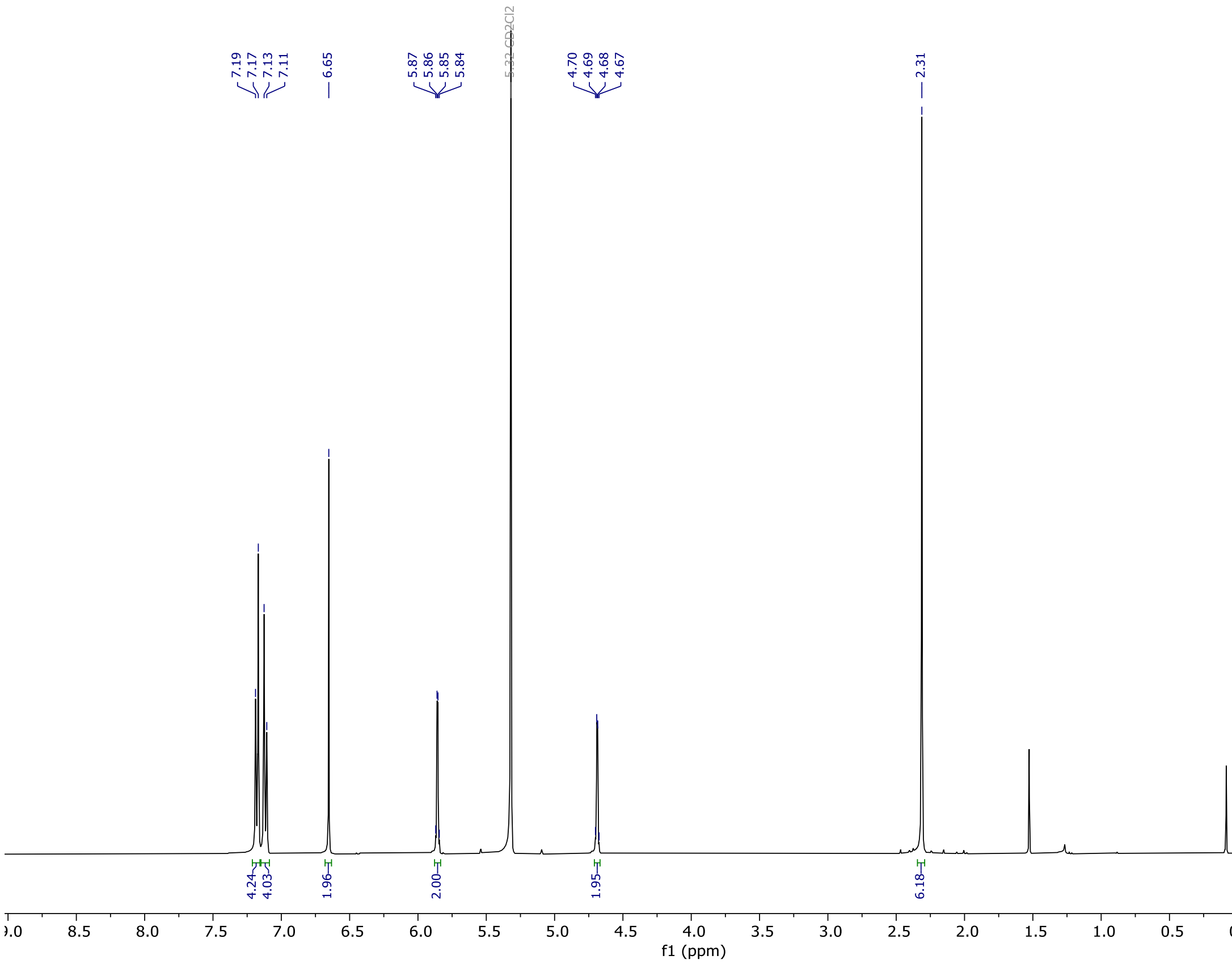

^{1}H NMR spectrum (400 MHz, CD_2Cl_2) of **4z**.

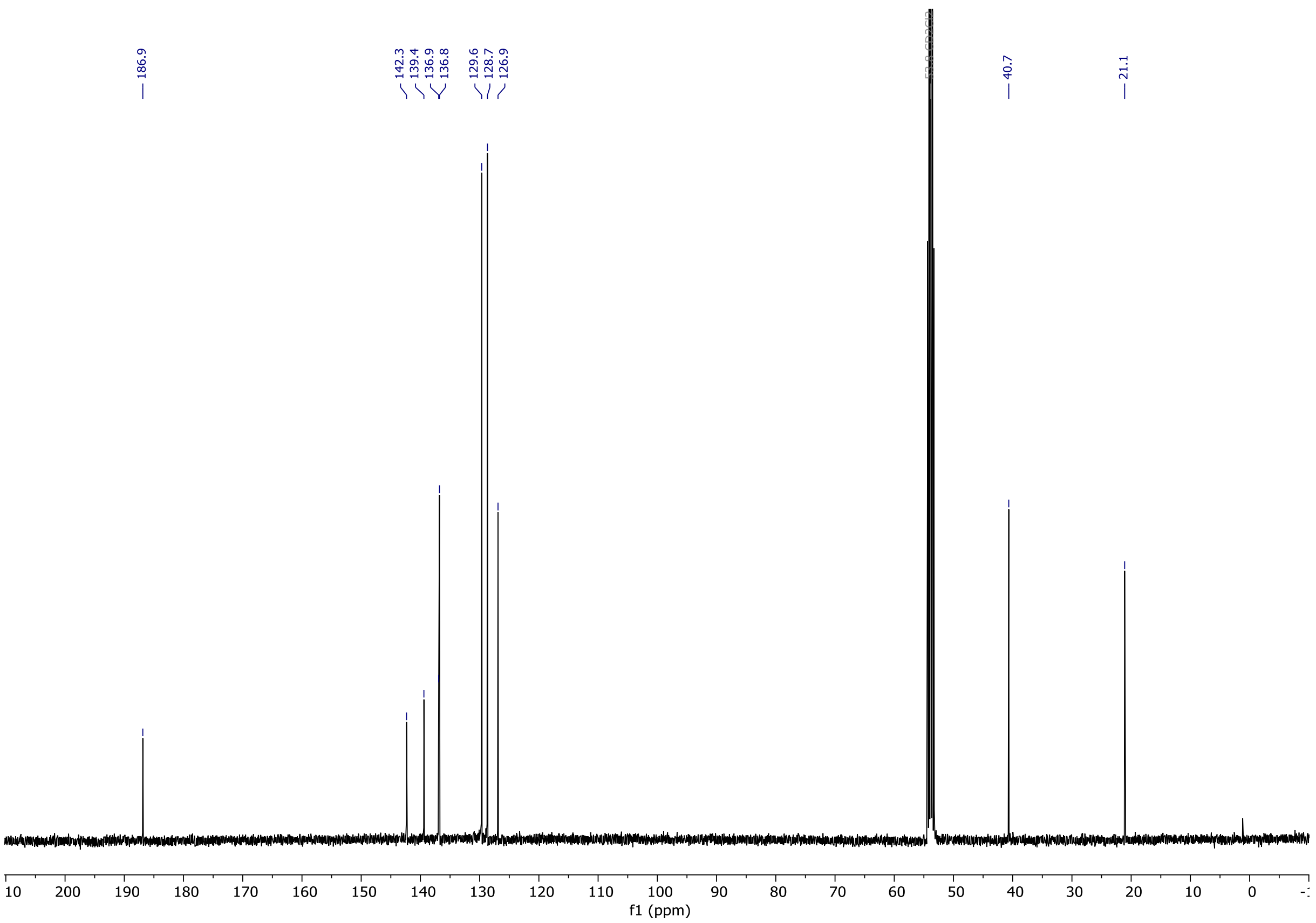

^{13}C NMR spectrum (101 MHz, CD_2Cl_2) of **4z**.

5,8-Dihydro-5,8-di(4-bromophenyl)naphthalene-1,4-dione (**4aa**)

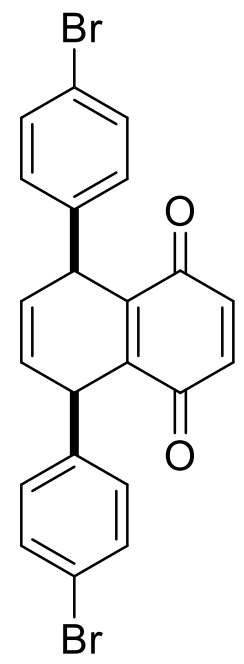

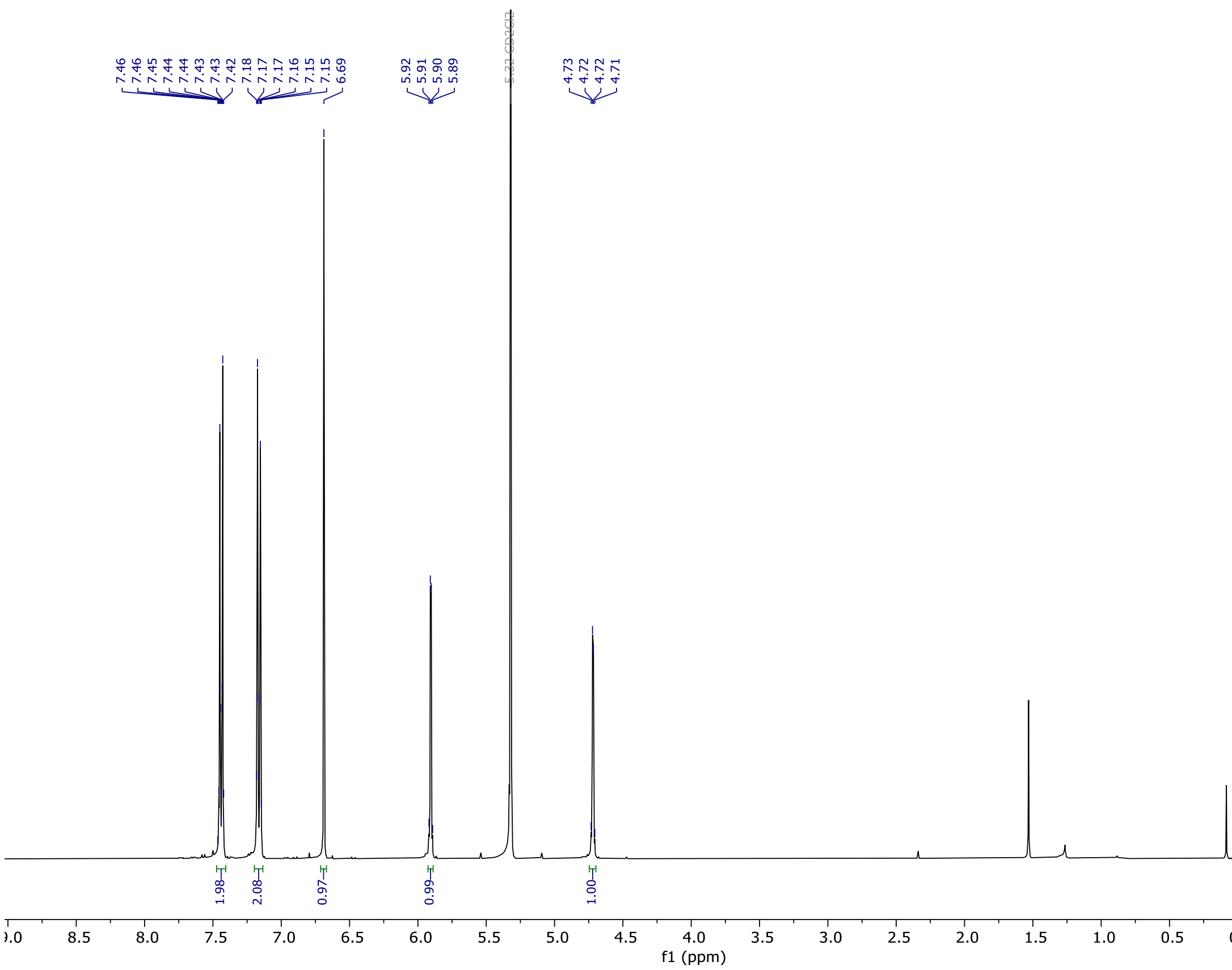

^{1}H NMR spectrum (400 MHz, CD_2Cl_2) of **4aa**.

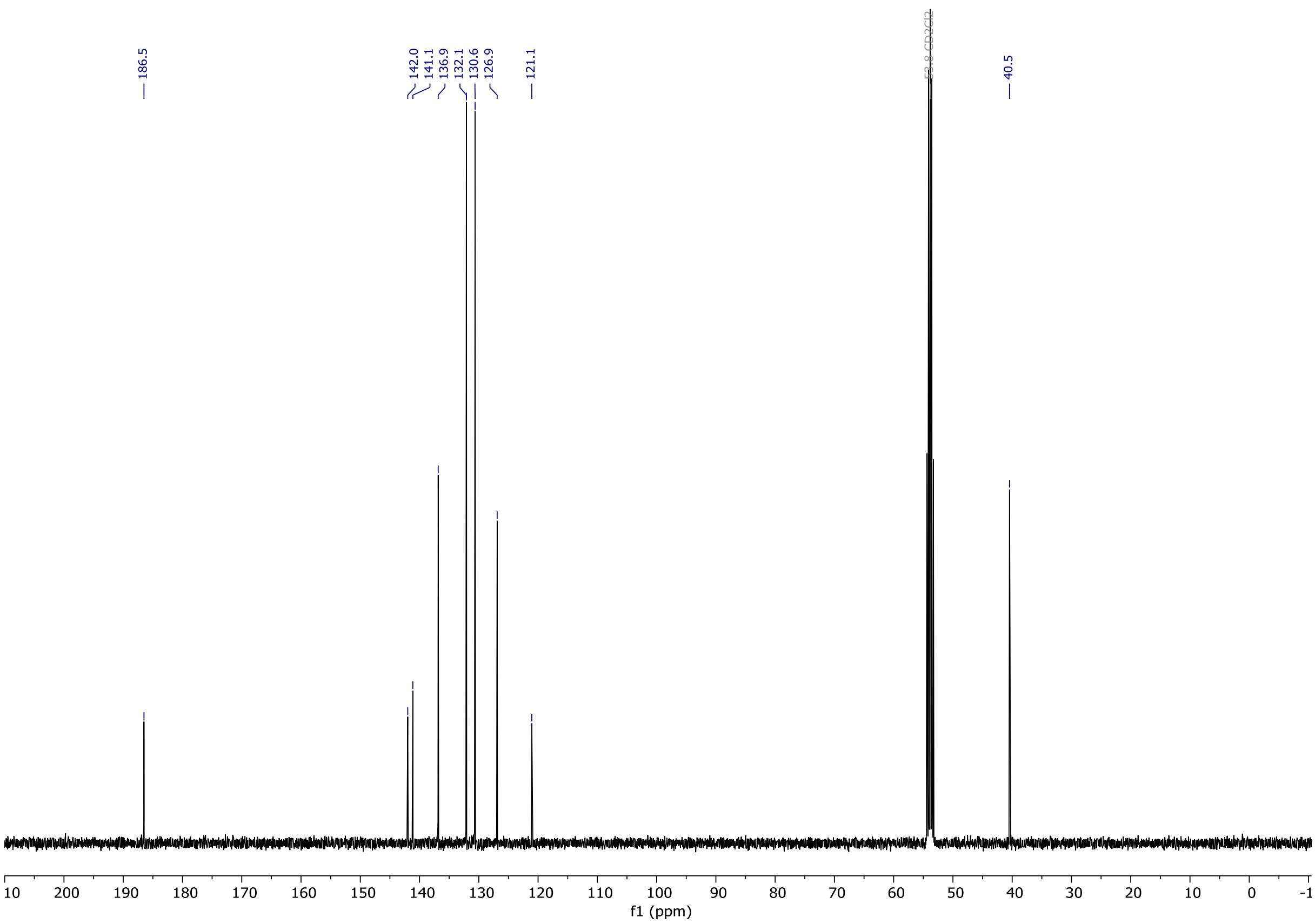

^{13}C NMR spectrum (101 MHz, CD_2Cl_2) of **4aa**.

5,8-Dihydro-5-ethoxycarbonyl-8-methylnaphthalene-1,4-dione (**4ab**)

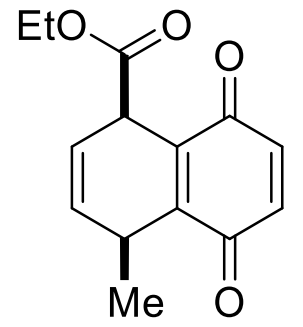

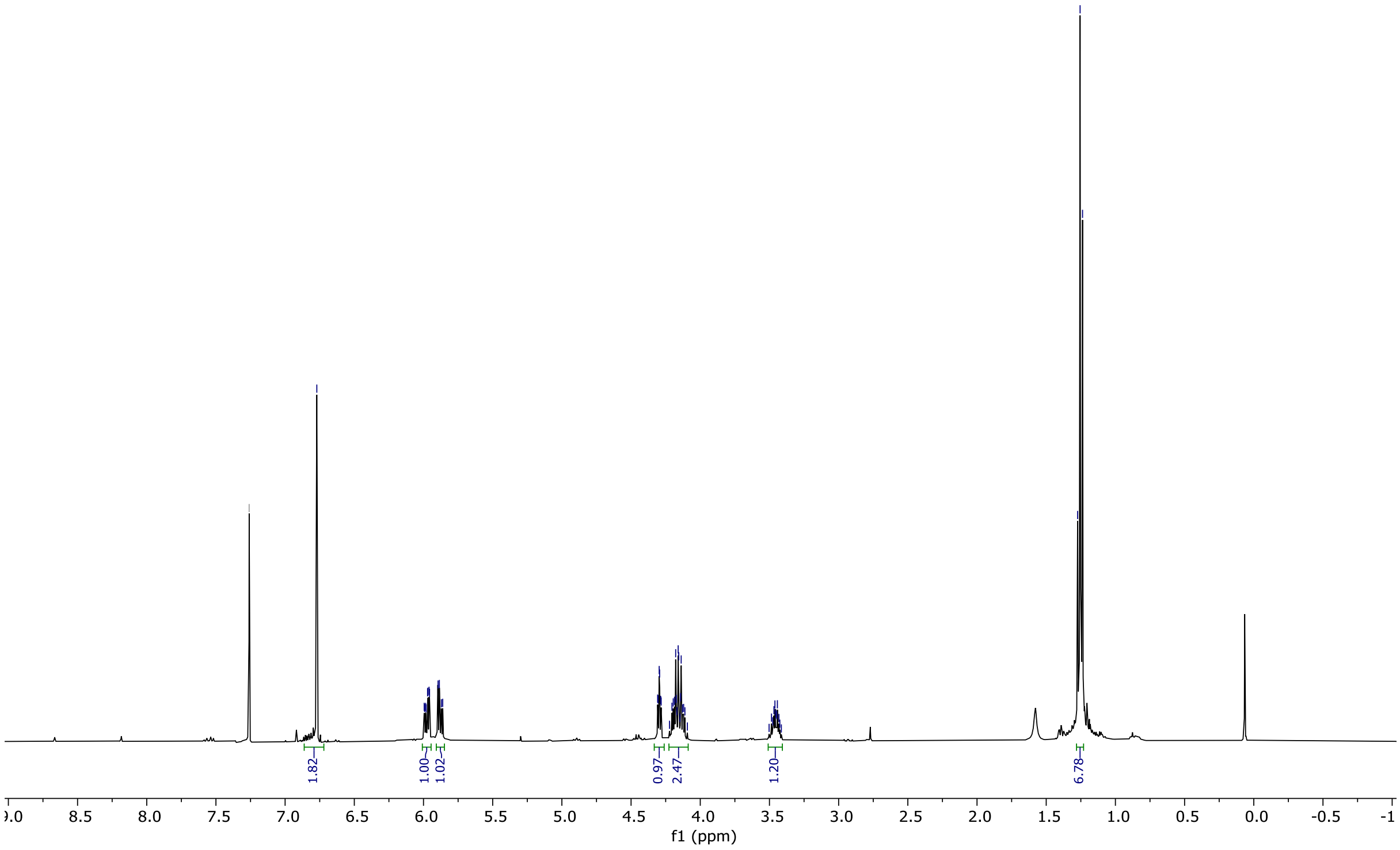

^{1}H NMR spectrum (400 MHz, CD_2Cl_2) of **4ab**.

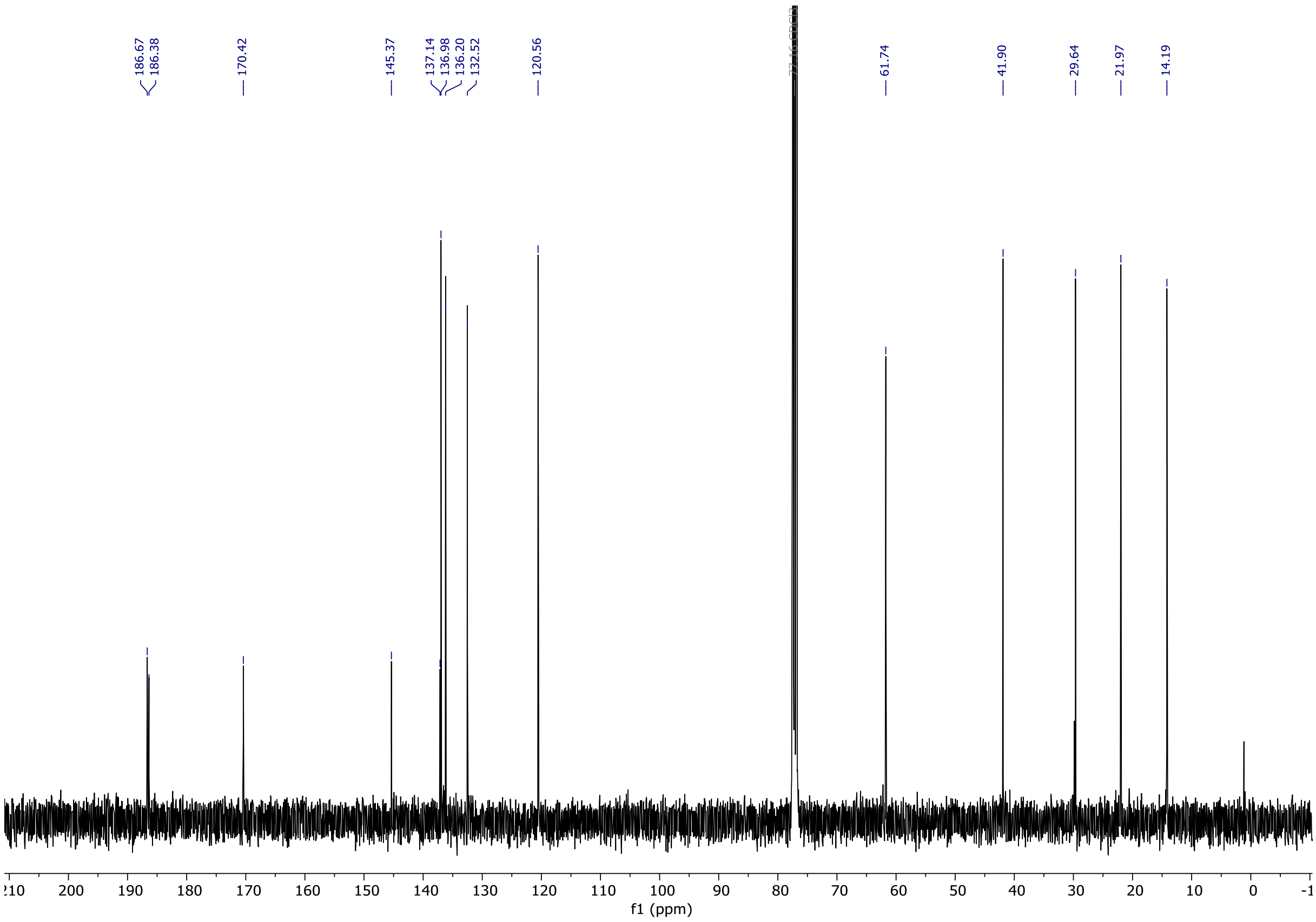

^{13}C NMR spectrum (101 MHz, CD_2Cl_2) of **4ab**.

5,8-Dihydro-5-ethoxycarbonyl-8-propylnaphthalene-1,4-dione (**4ac**)

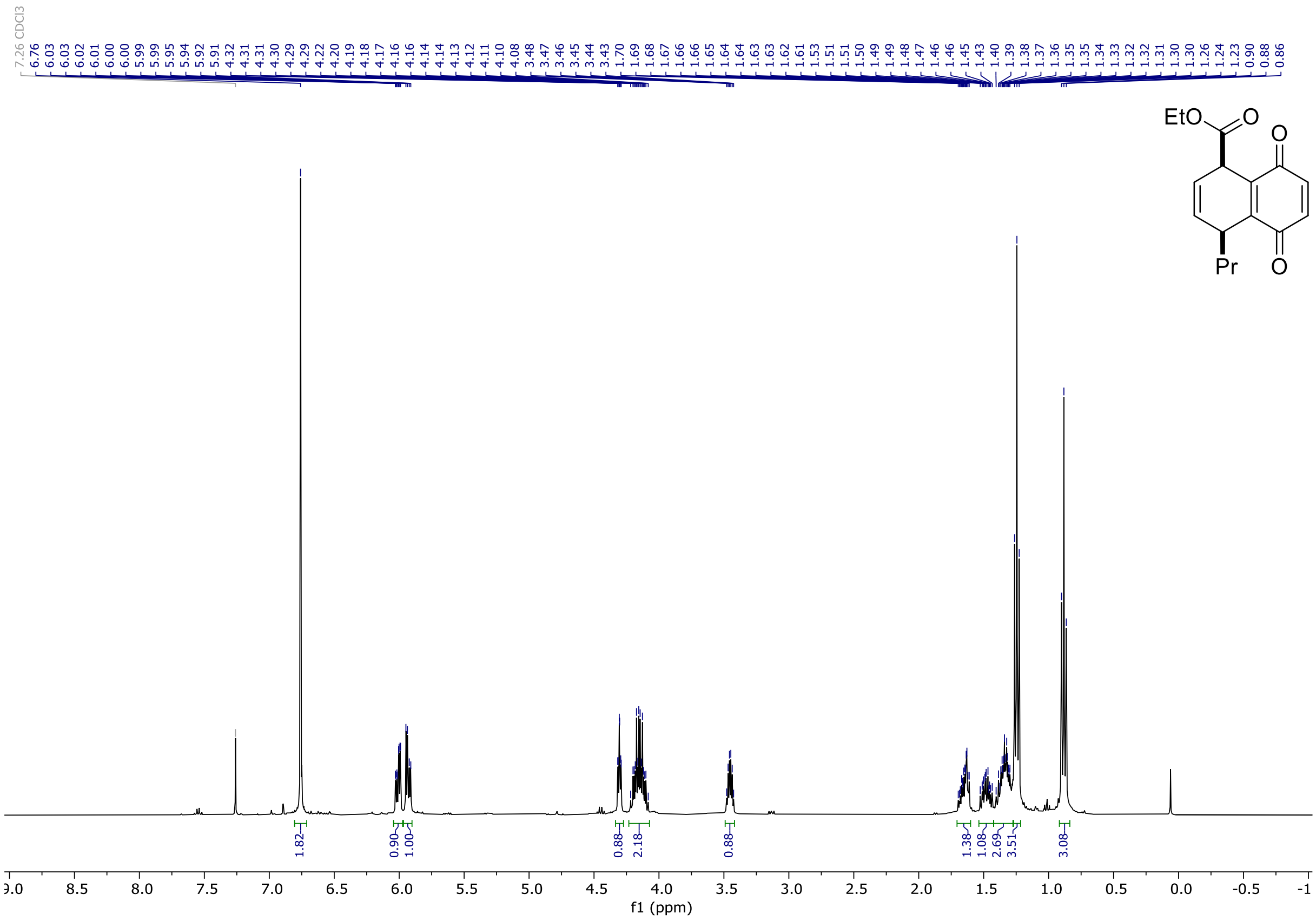

^{1}H NMR spectrum (400 MHz, CD_2Cl_2) of **4ac**.

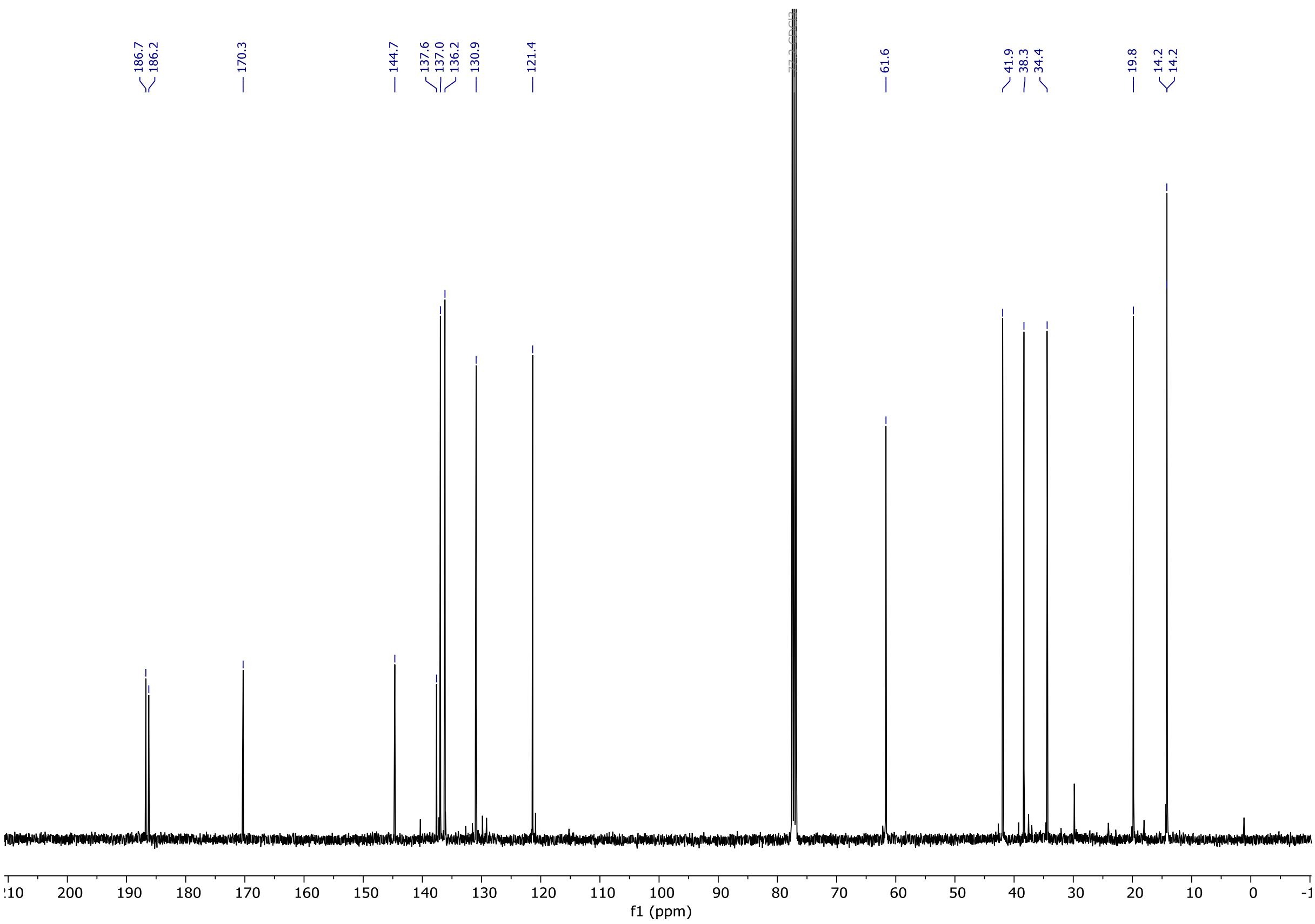

^{13}C NMR spectrum (101 MHz, CD_2Cl_2) of **4ac**.

9,10-Dihydro-9,10[1',2']-benzenoanthracene-1,4-dione (4**ag**)

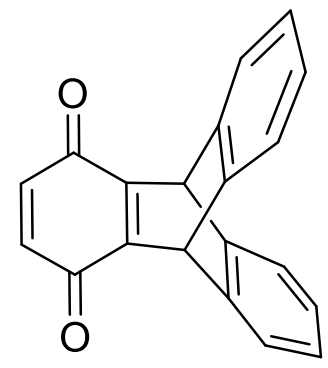

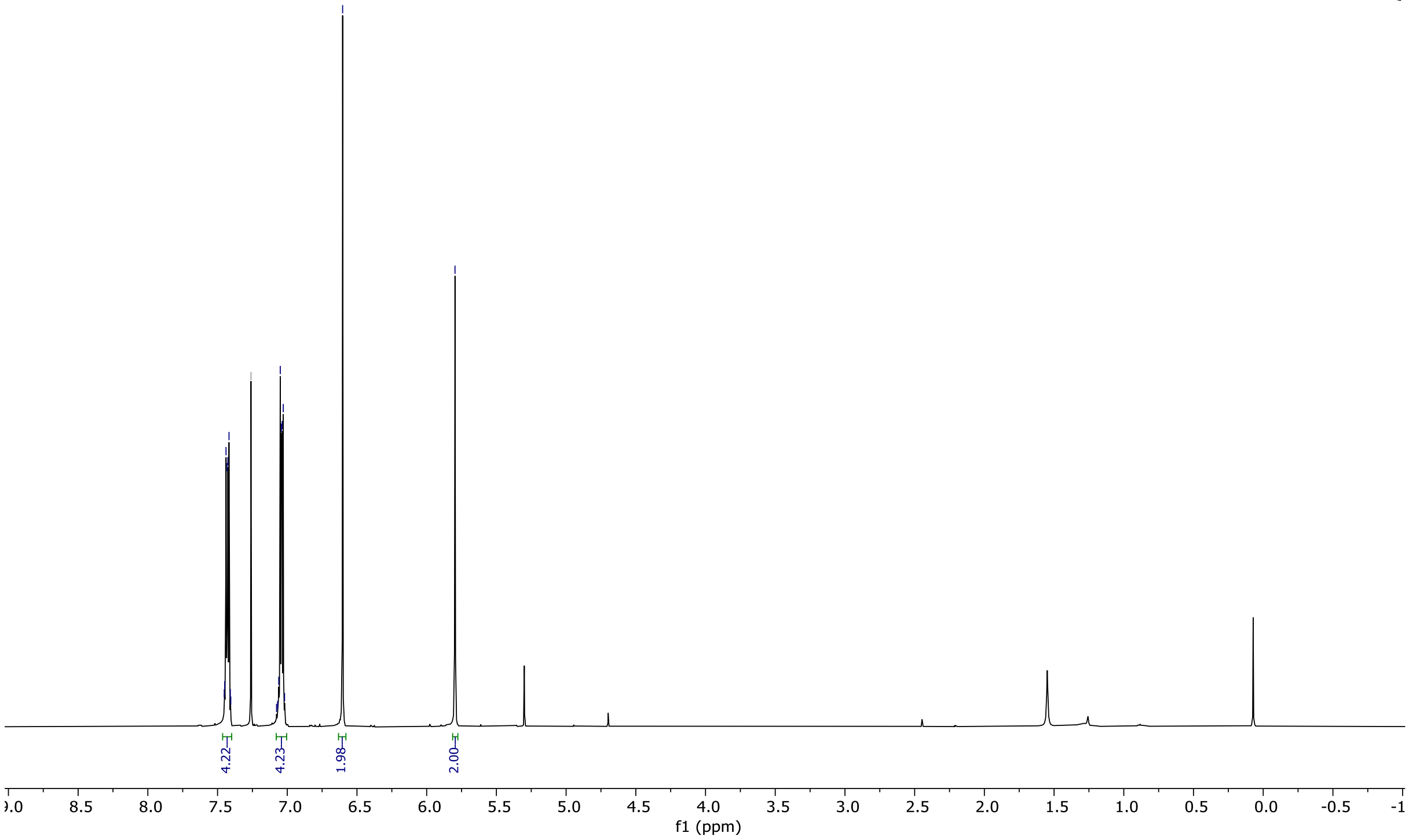

^{1}H NMR spectrum (400 MHz, CD_2Cl_2) of **4ag**.

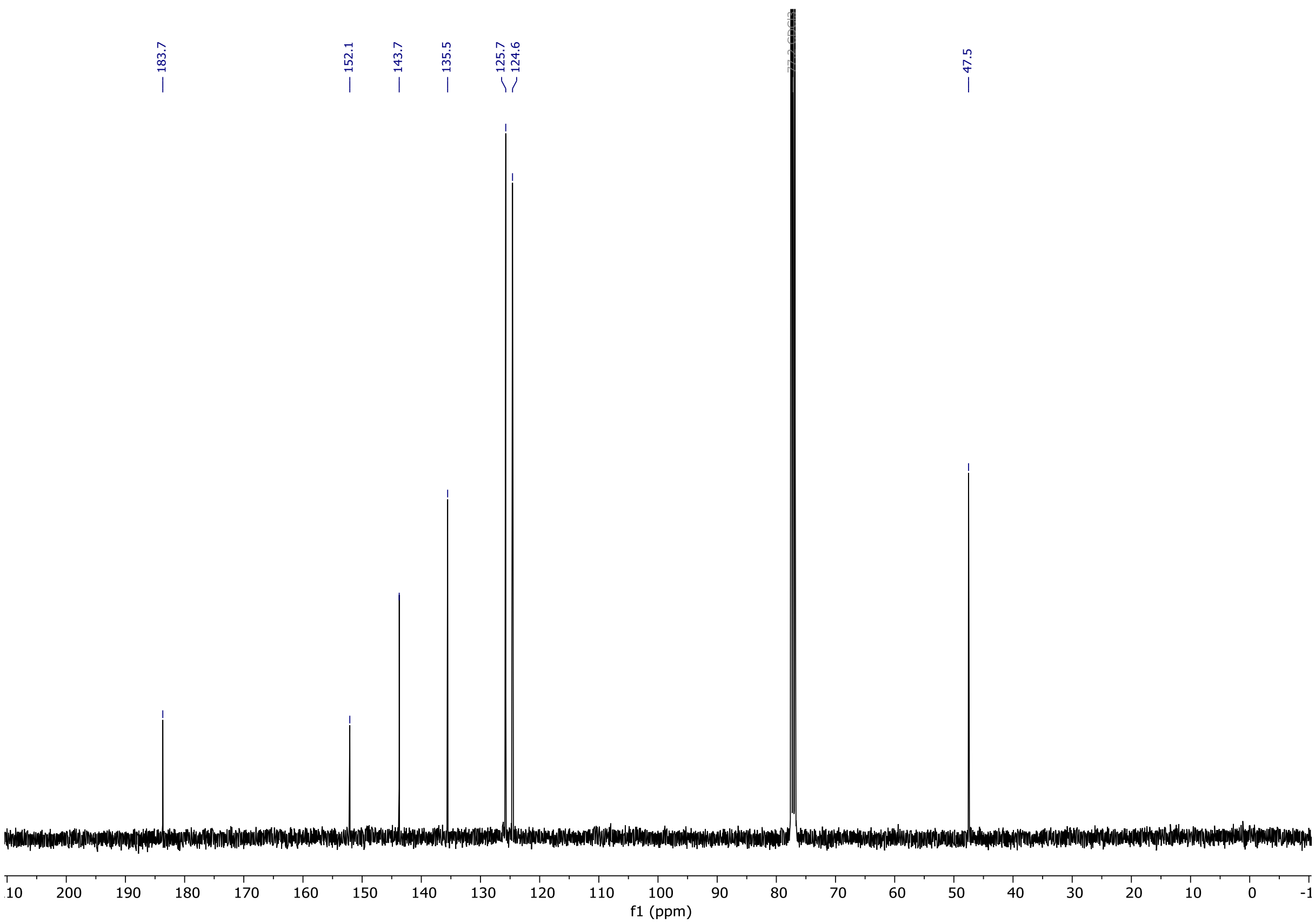

^{13}C NMR spectrum (101 MHz, CD_2Cl_2) of **4ag**.

5,8-Dihydro-2-methyl-5,8-diphenylnaphthalene-1,4-dione (**4ah**)

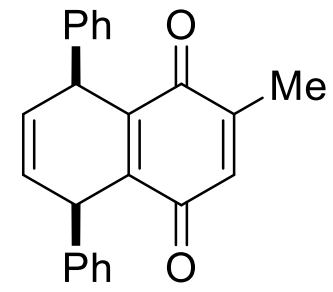

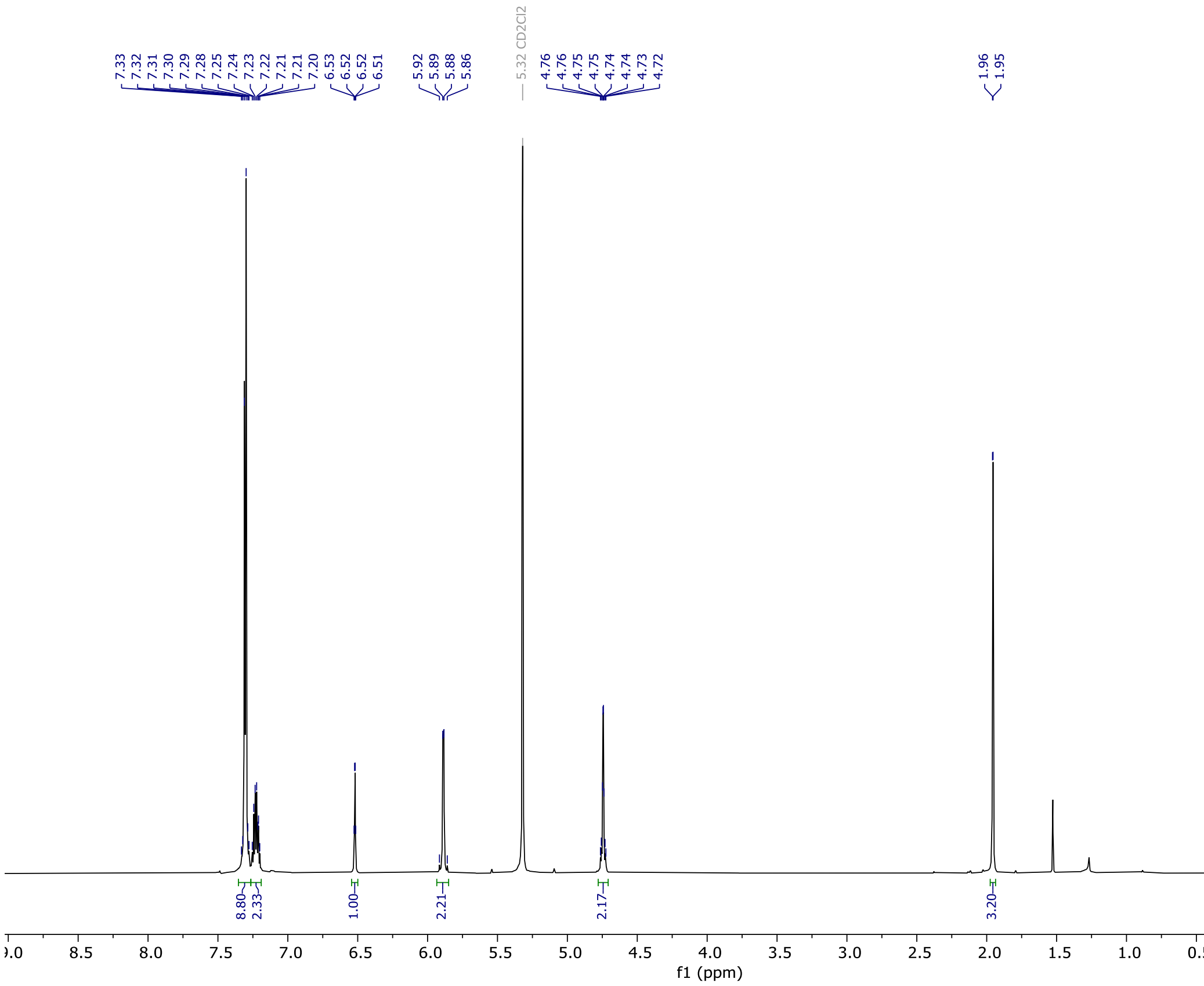

^{1}H NMR spectrum (400 MHz, CD_2Cl_2) of **4ah**.

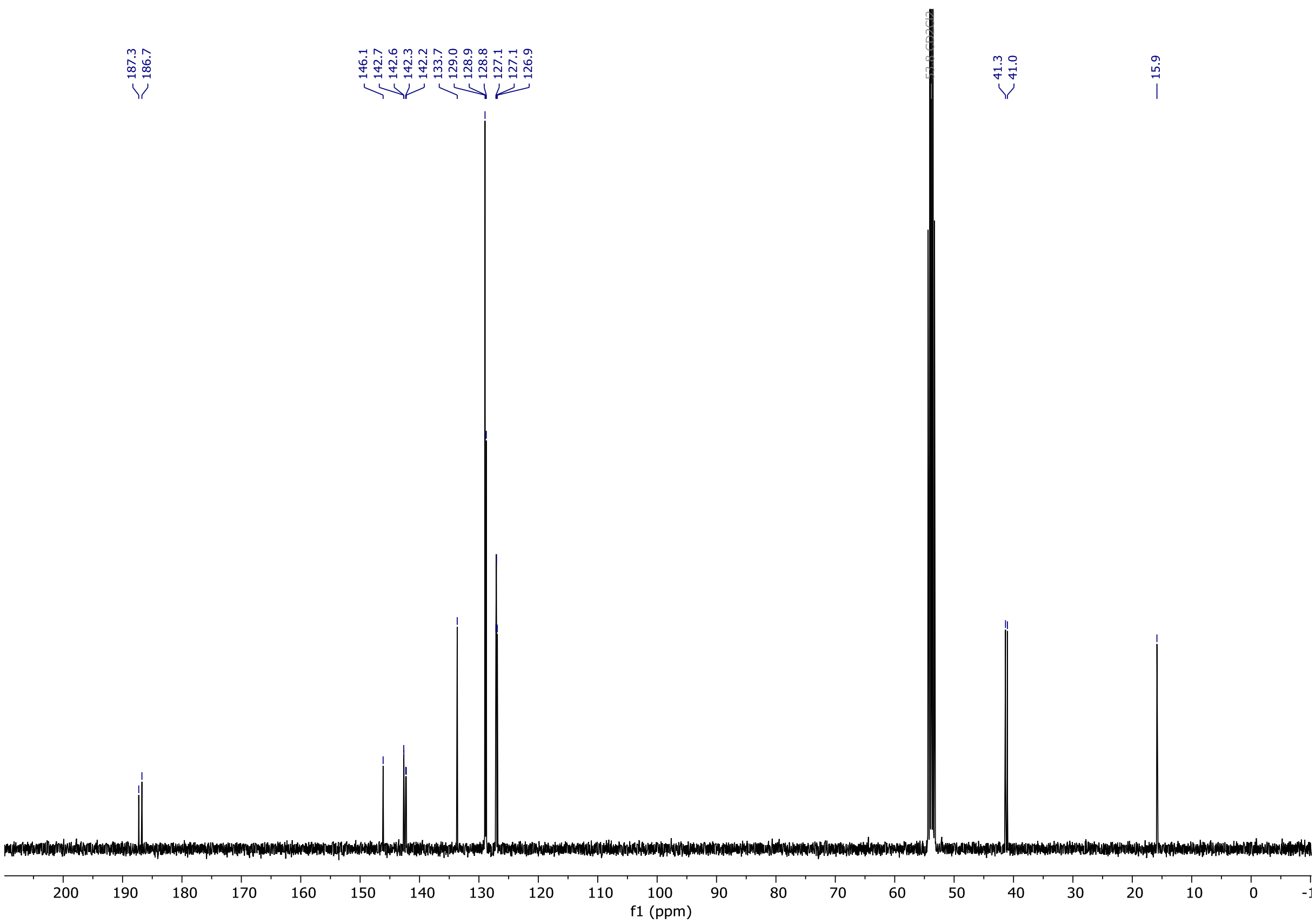

^{13}C NMR spectrum (101 MHz, CD_2Cl_2) of **4ah**.

5,8-Dihydro-2,3-dimethyl-5,8-diphenylnaphthalene-1,4-dione (**4ai**)

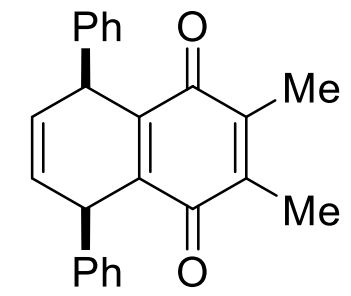

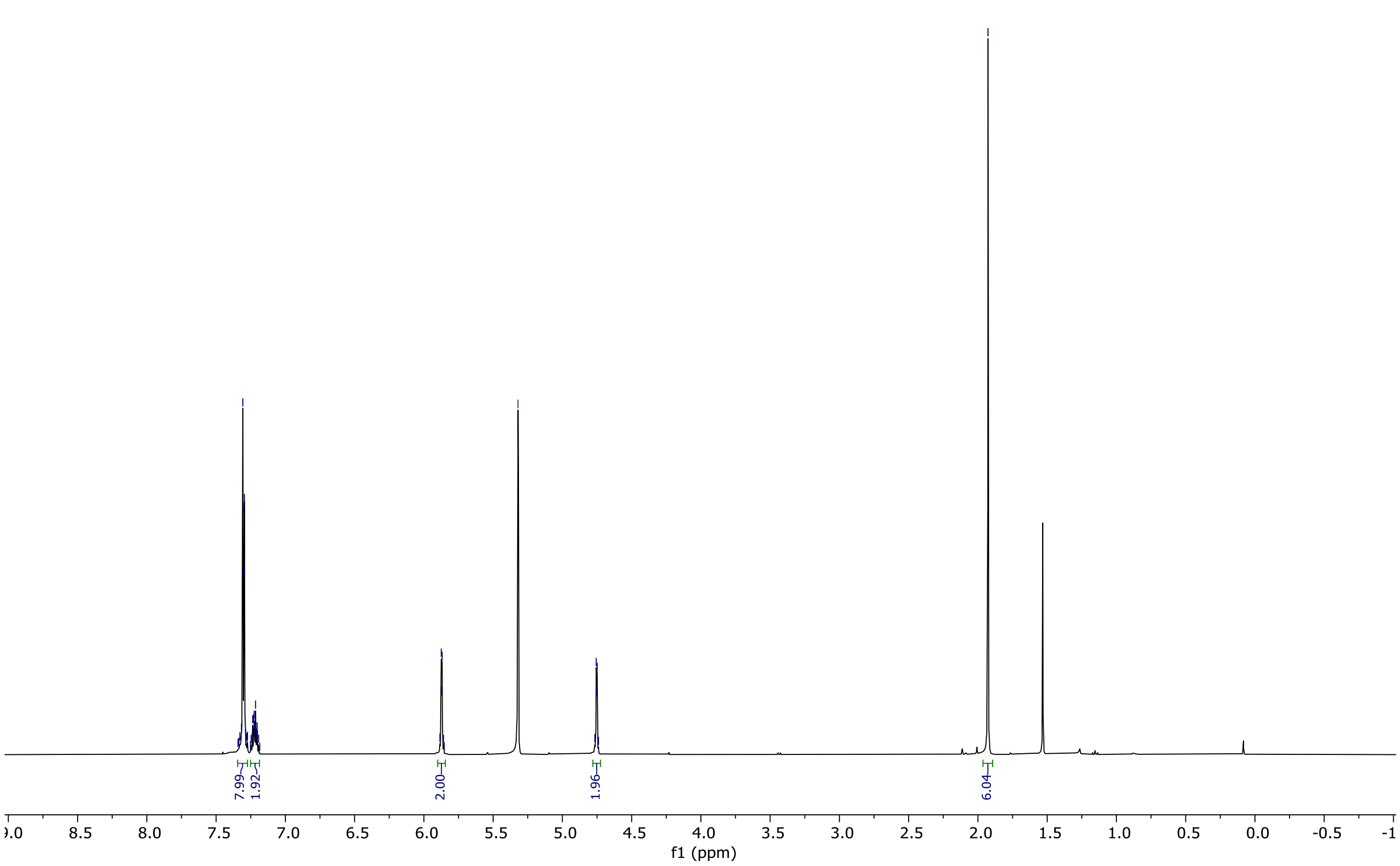

^{1}H NMR spectrum (400 MHz, CD_2Cl_2) of **4ai**.

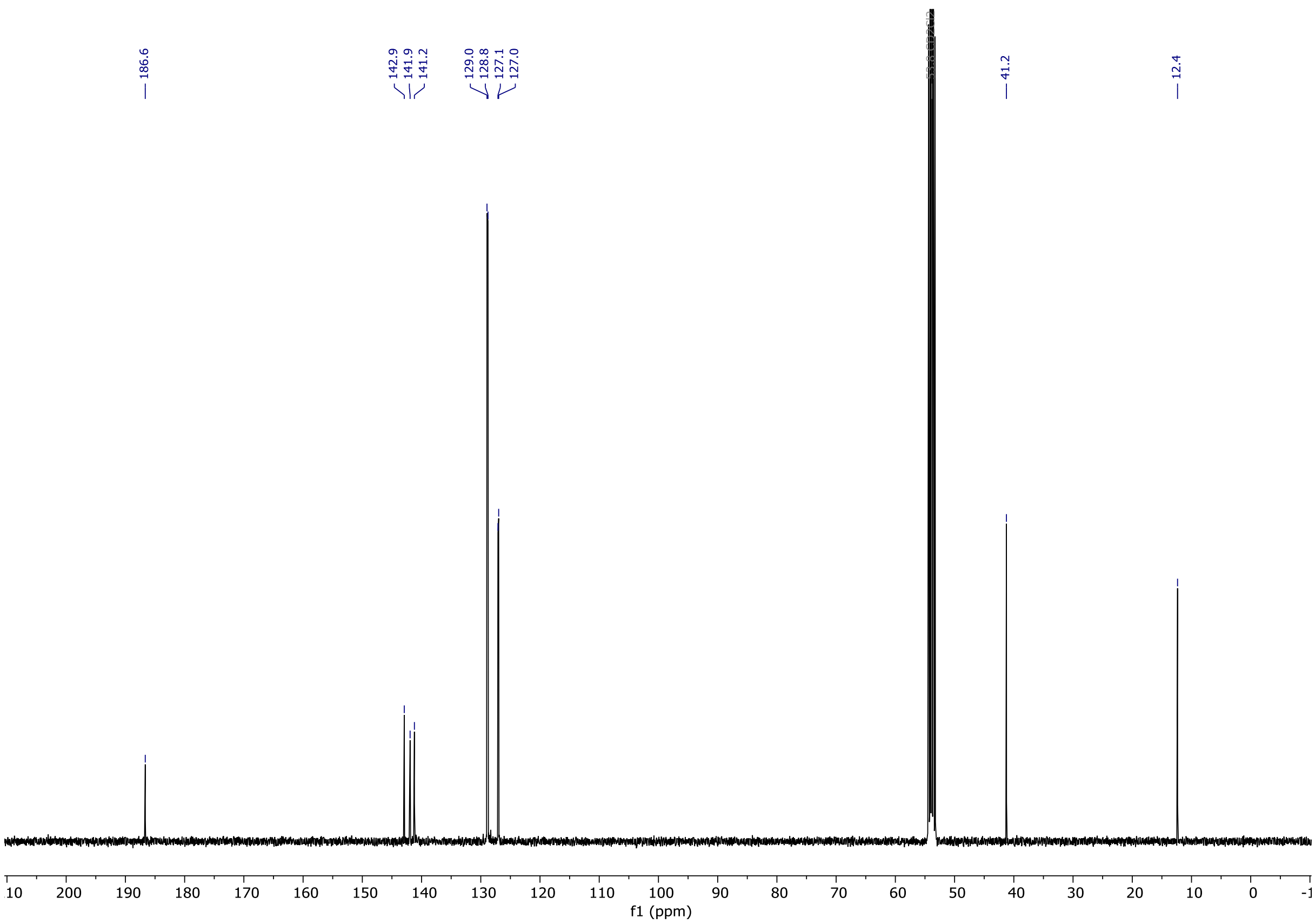

^{13}C NMR spectrum (101 MHz, CD_2Cl_2) of **4ai**.

5,8-Dihydro-2-cyclopropyl-5,8-diphenylnaphthalene-1,4-dione (**4aj**)

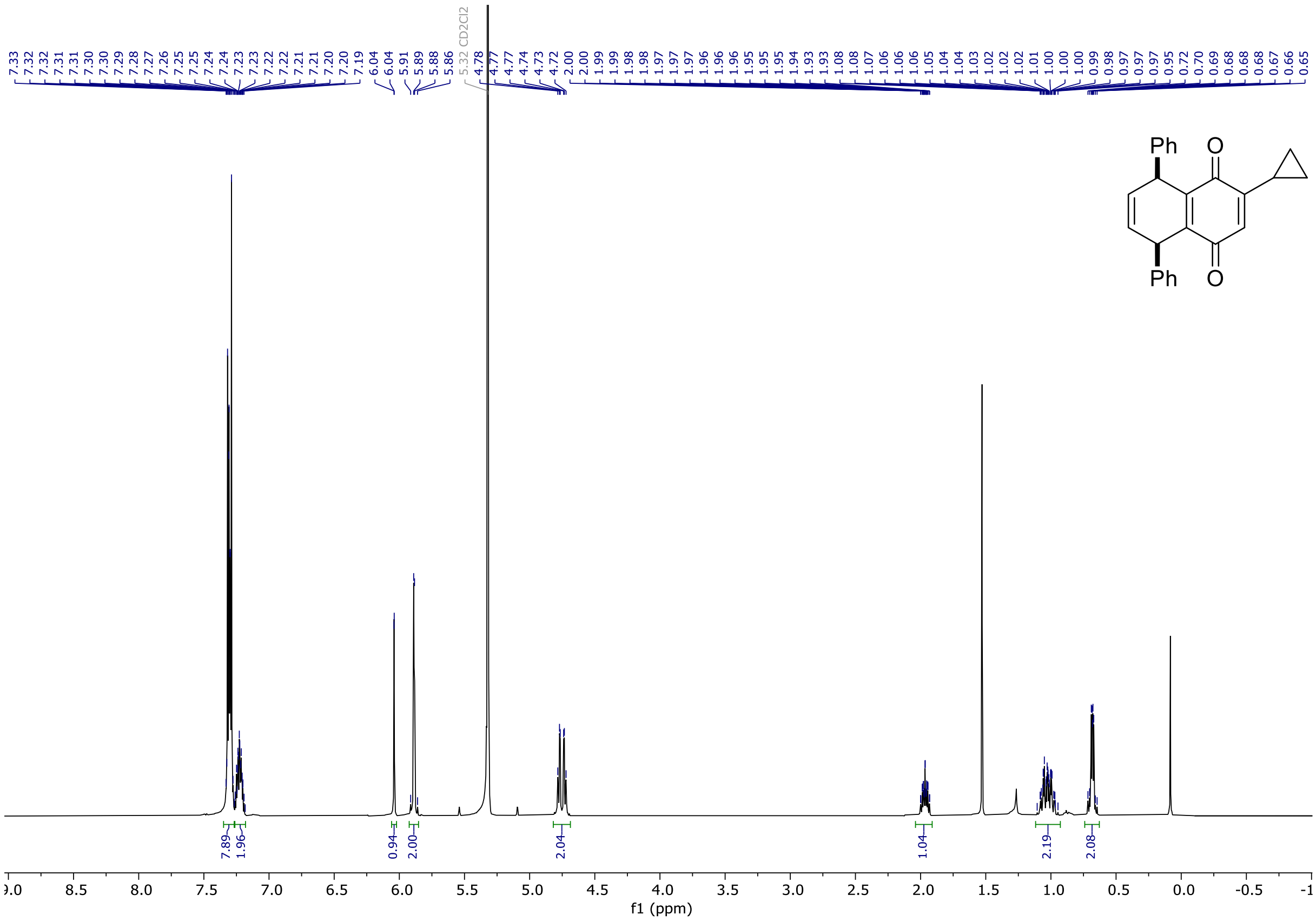

^{1}H NMR spectrum (400 MHz, CD_2Cl_2) of **4aj**.

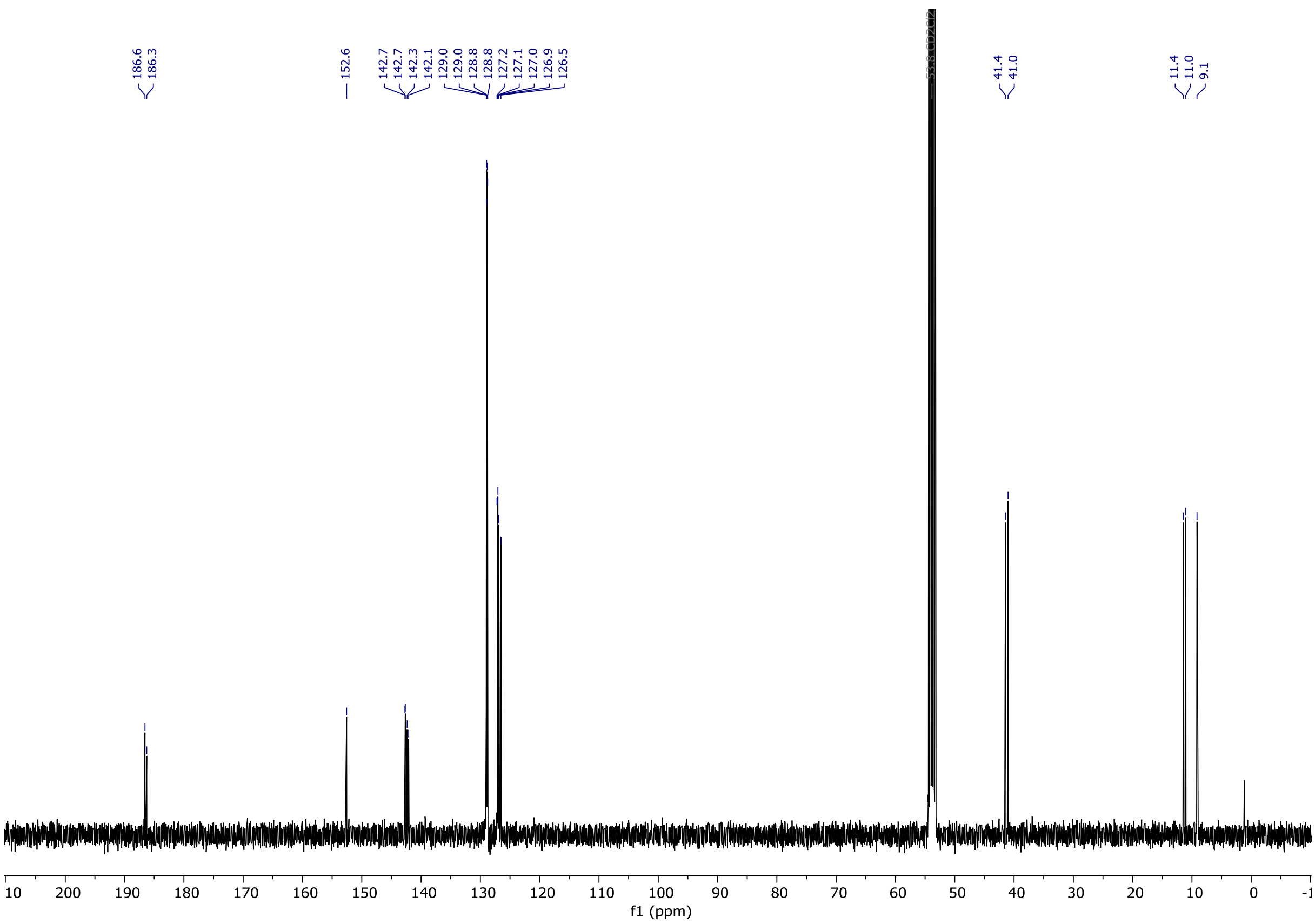

^{13}C NMR spectrum (101 MHz, CD_2Cl_2) of **4aj**.

5,8-Dihydro-2-cyclohexyl-5,8-diphenylnaphthalene-1,4-dione (**4ak**)

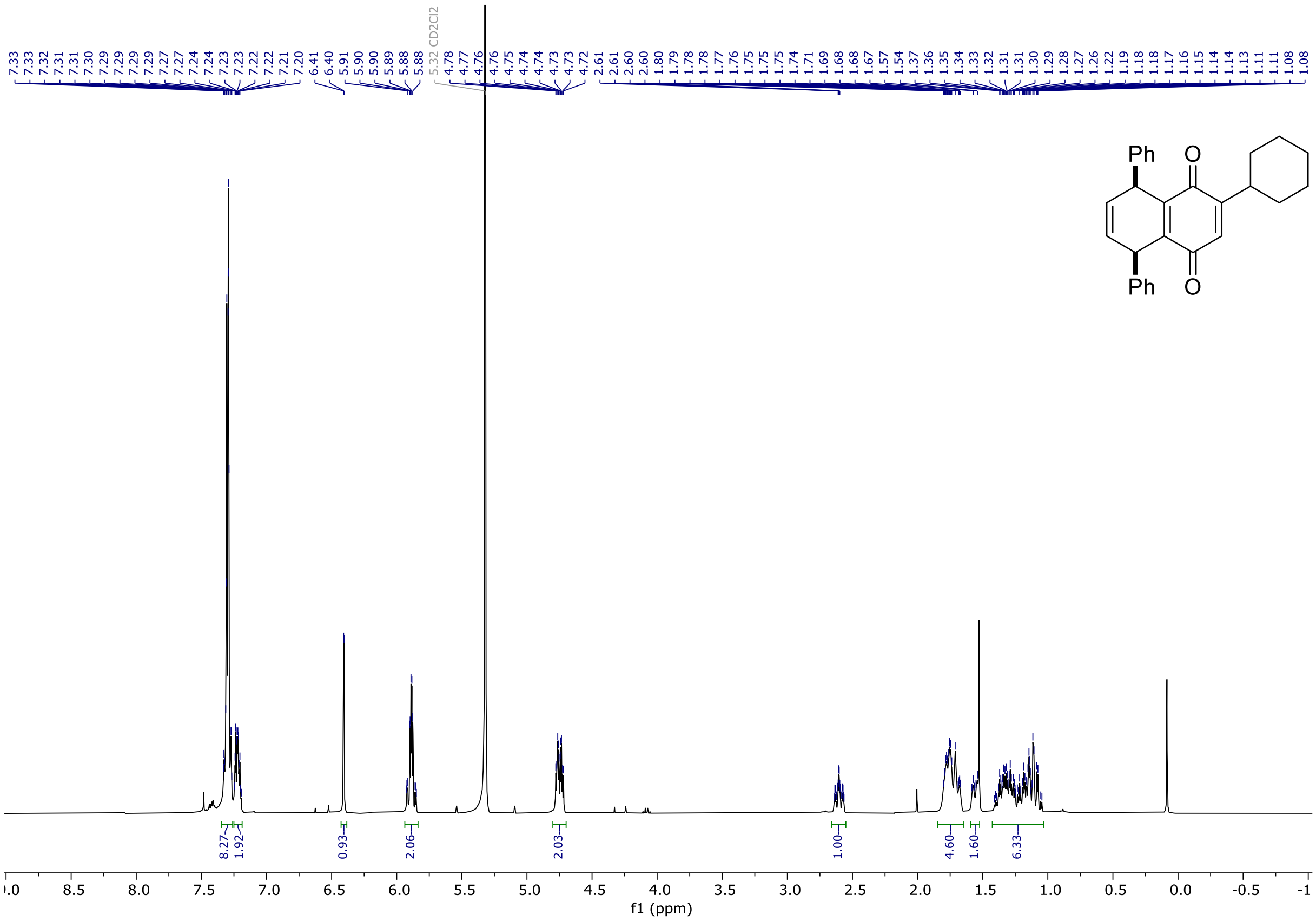

^{1}H NMR spectrum (400 MHz, CD_2Cl_2) of **4ak**.

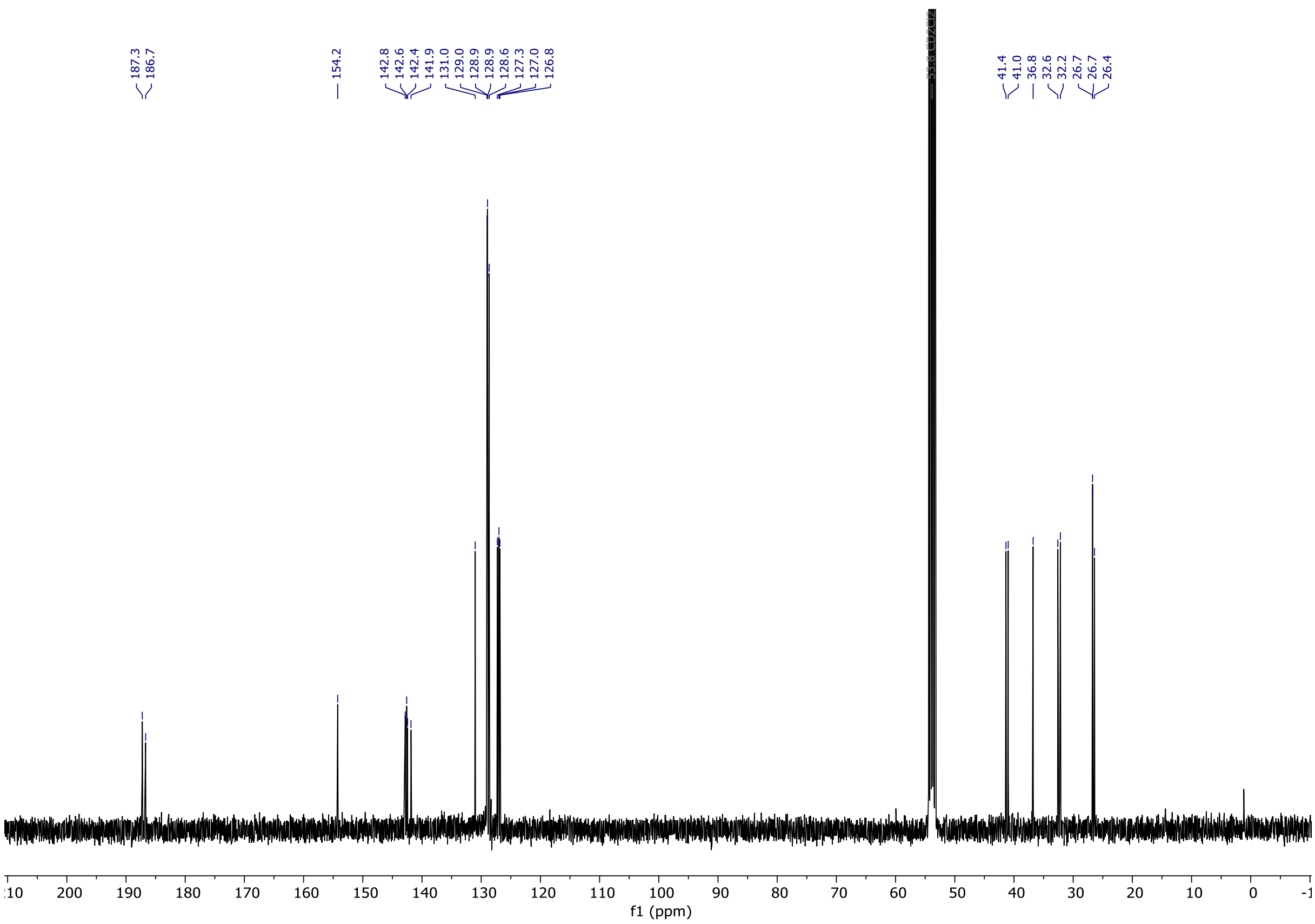

^{13}C NMR spectrum (101 MHz, CD_2Cl_2) of **4ak**.

5,8-Dihydro-2,5,8-triphenylnaphthalene-1,4-dione (**4al**)

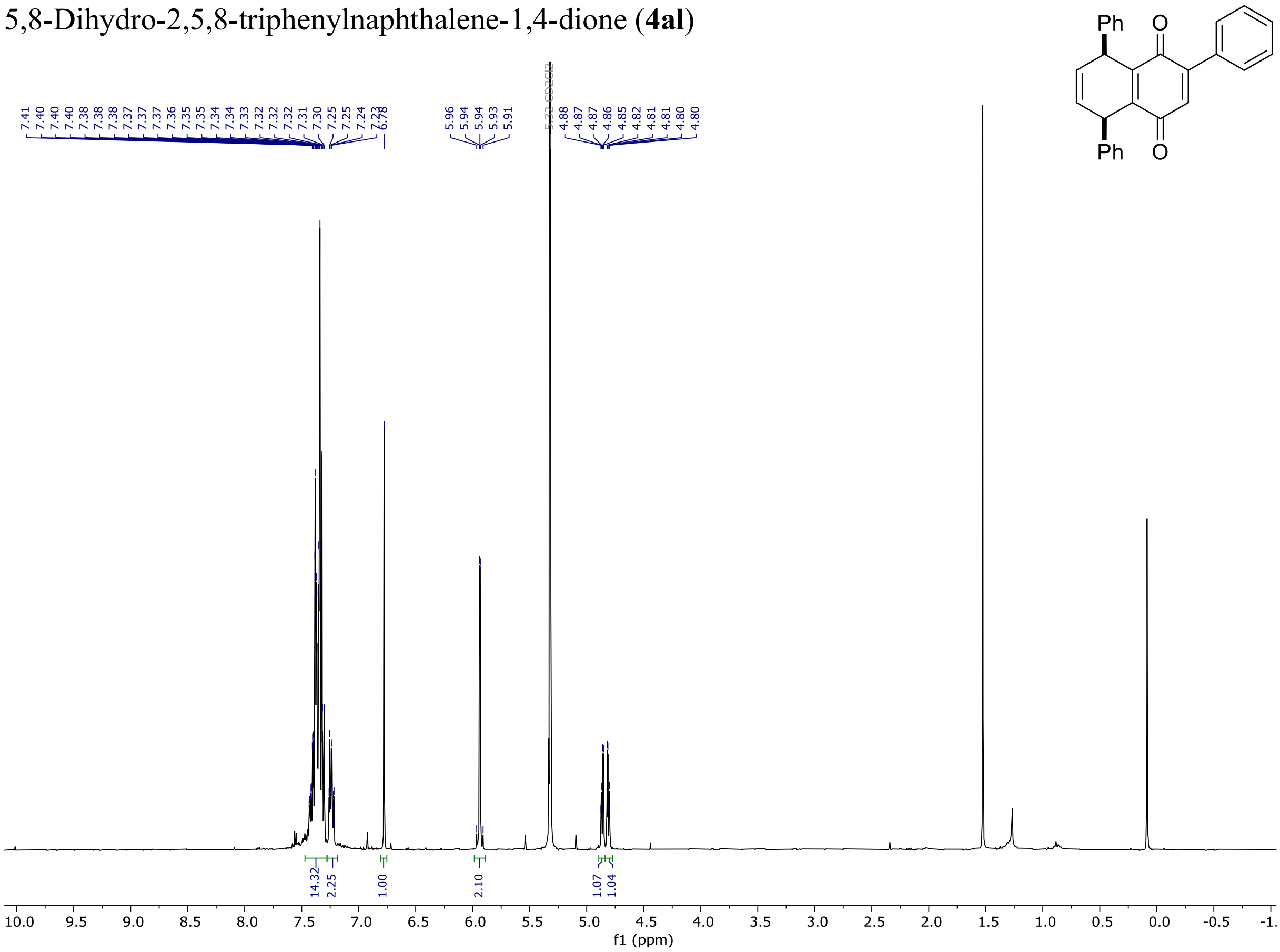

^{1}H NMR spectrum (400 MHz, CD_2Cl_2) of **4al**.

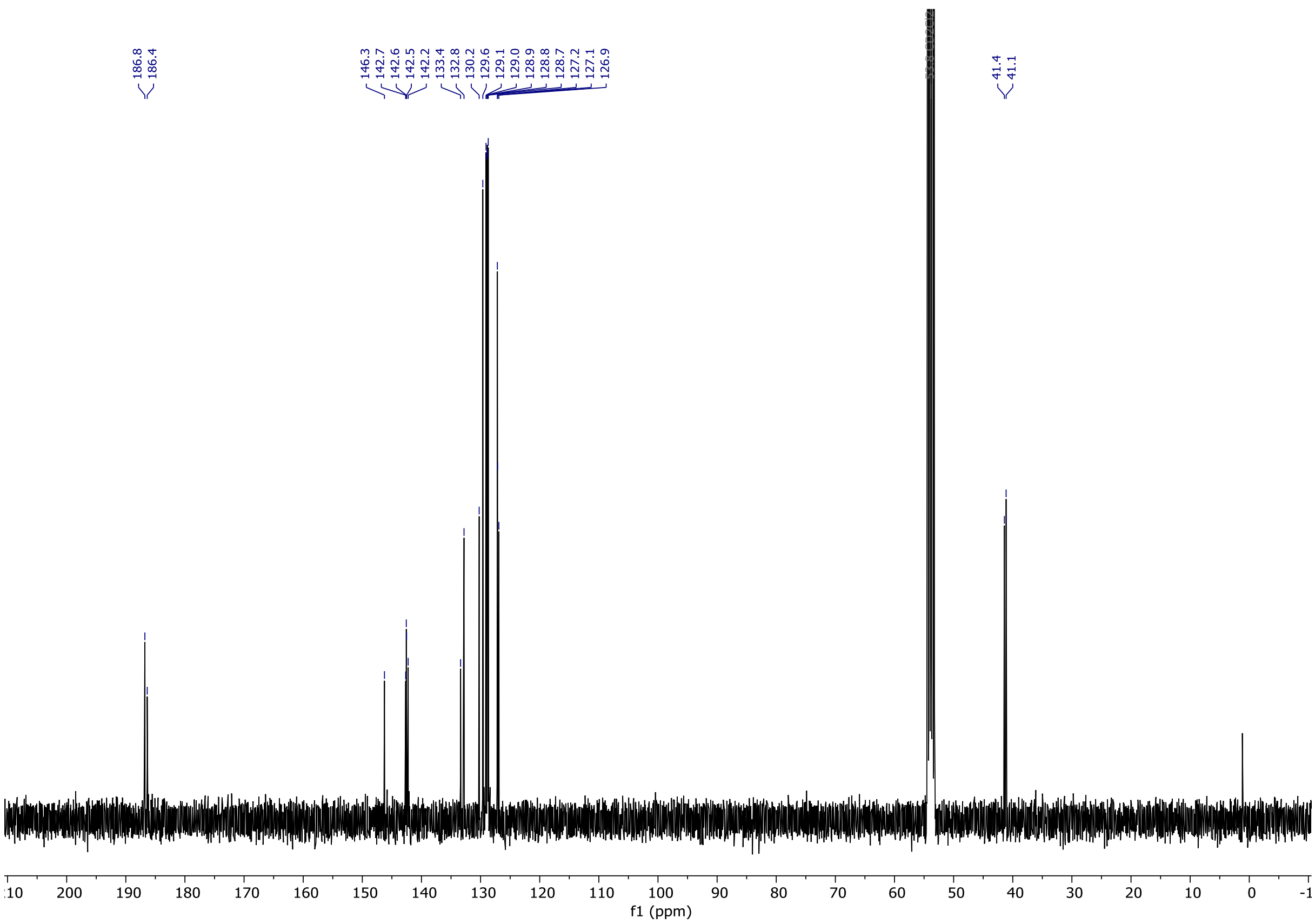

^{13}C NMR spectrum (101 MHz, CD_2Cl_2) of **4al**.

5,7,10,12-Tetrahydro-7,10-diphenyl-5,12[1',2']benzenonaphthacene-6,11-dione (**4am**)

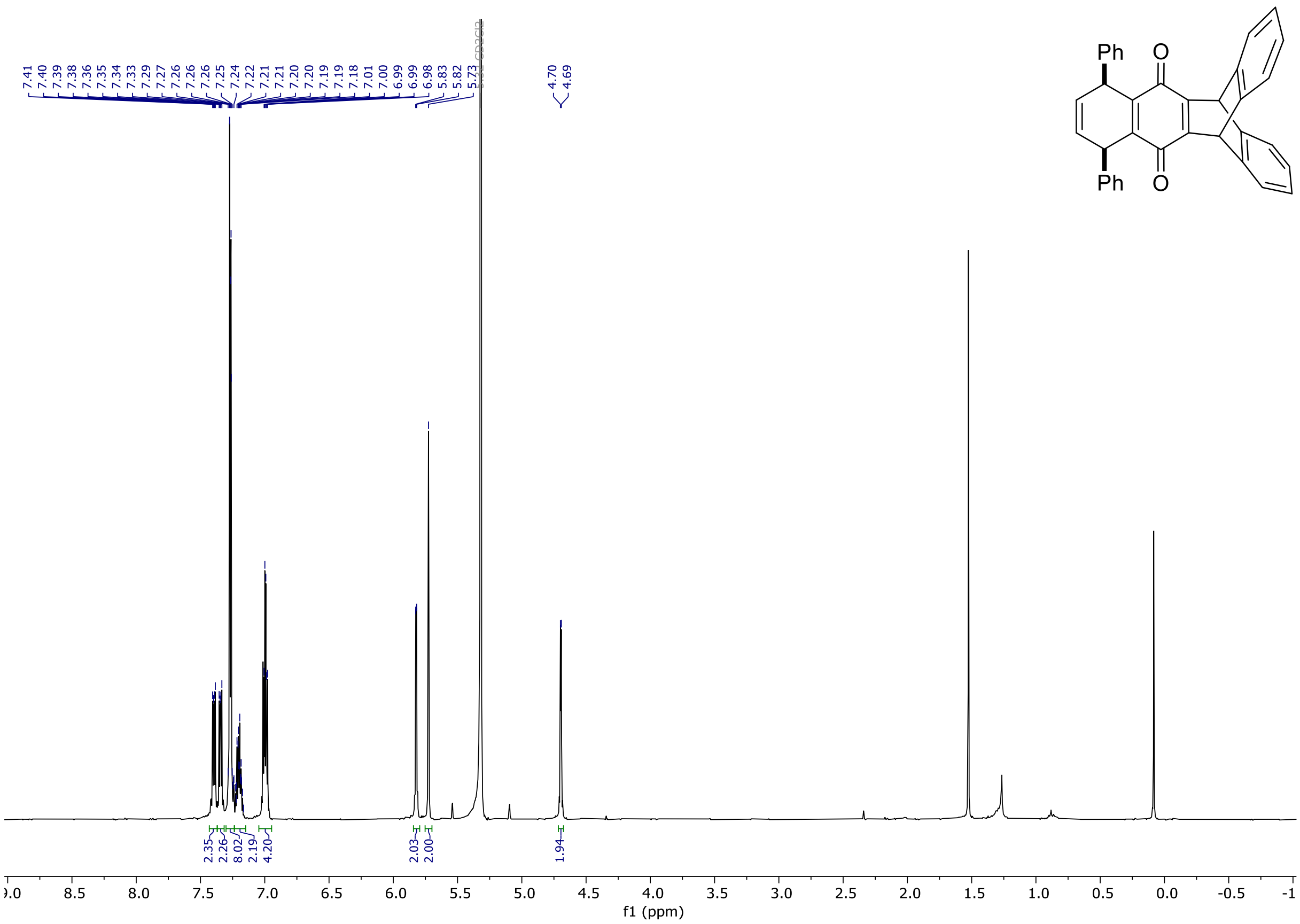

^{1}H NMR spectrum (400 MHz, CD_2Cl_2) of **4am**.

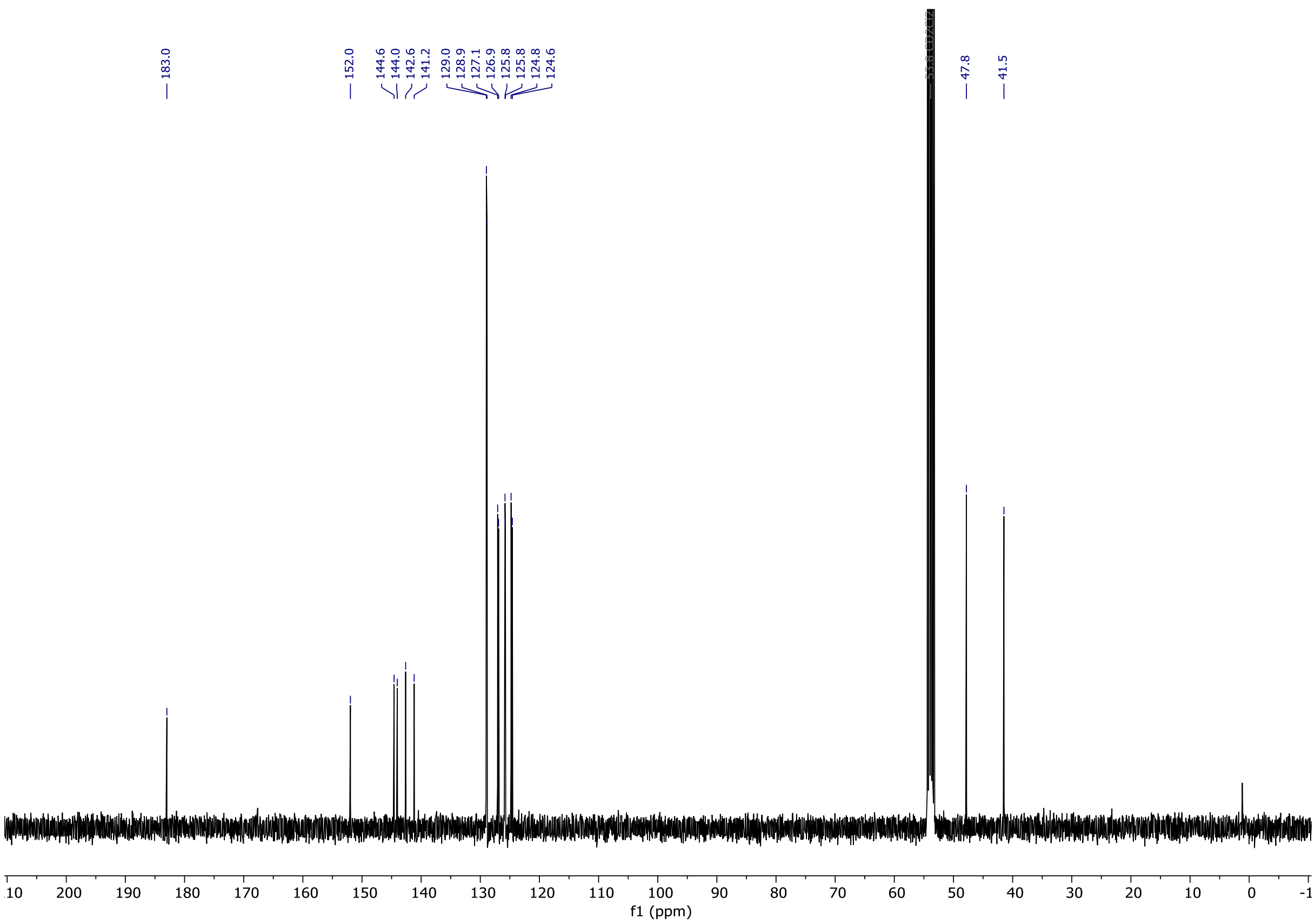

^{13}C NMR spectrum (101 MHz, CD_2Cl_2) of **4am**.

5,8-Dihydro-2-chloro-5,8-diphenylnaphthalene-1,4-dione (**4an**)

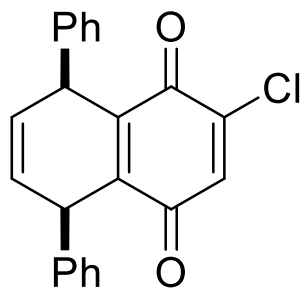

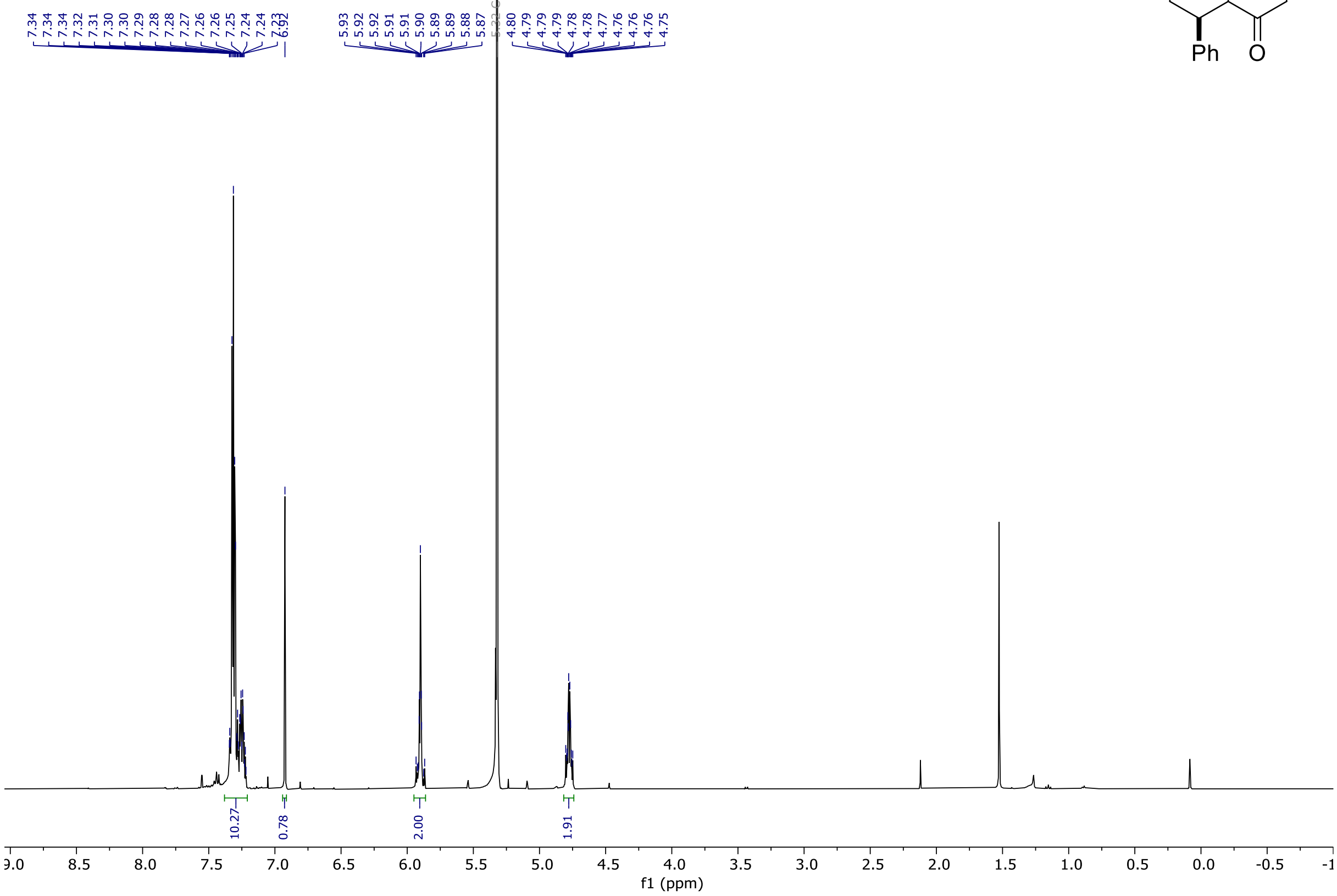

^{1}H NMR spectrum (400 MHz, CD_2Cl_2) of **4an**.

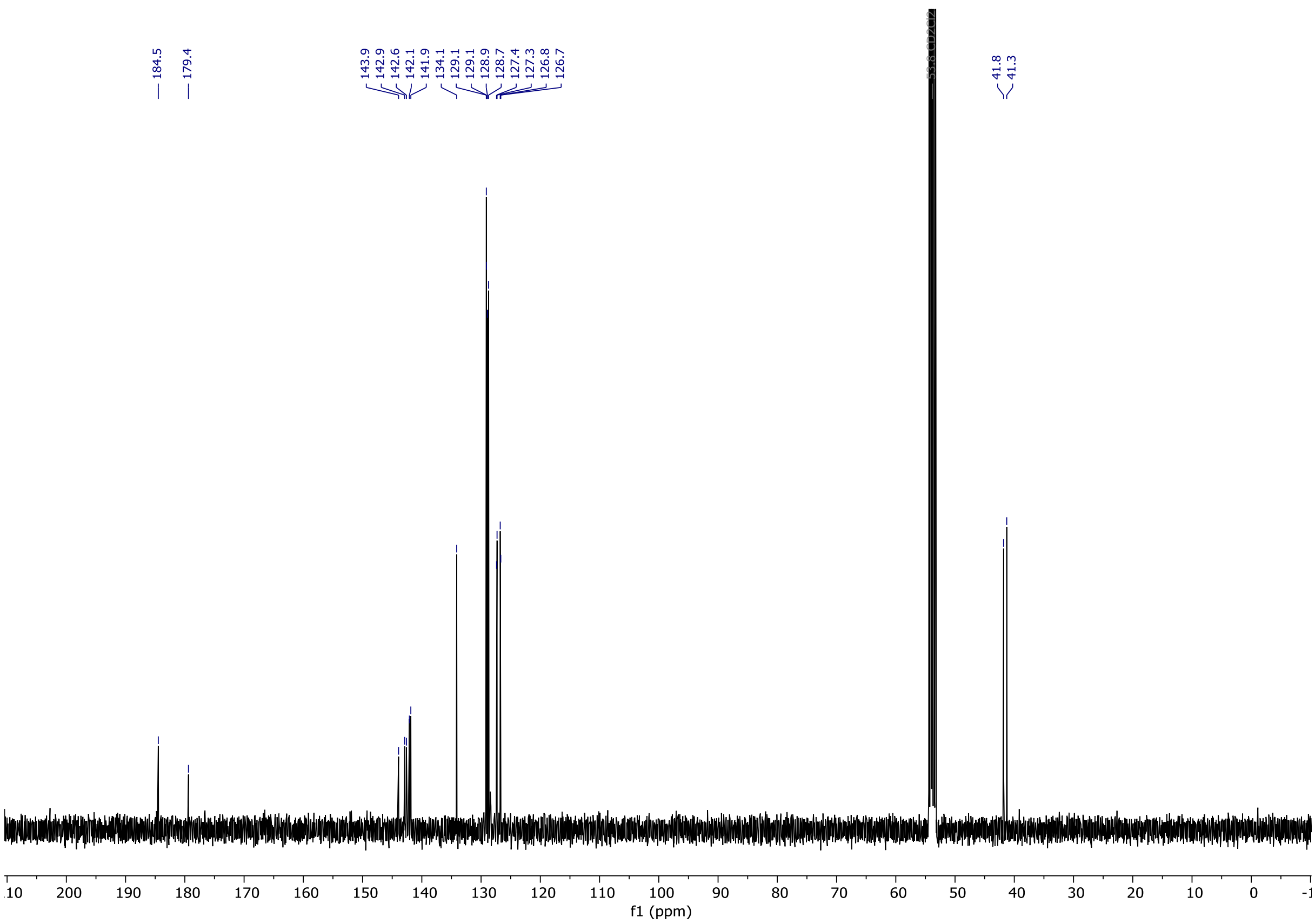

^{13}C NMR spectrum (101 MHz, CD_2Cl_2) of **4an**.

5,8-Dihydro-2-acetamido-5,8-diphenylnaphthalene-1,4-dione (**4ao**)

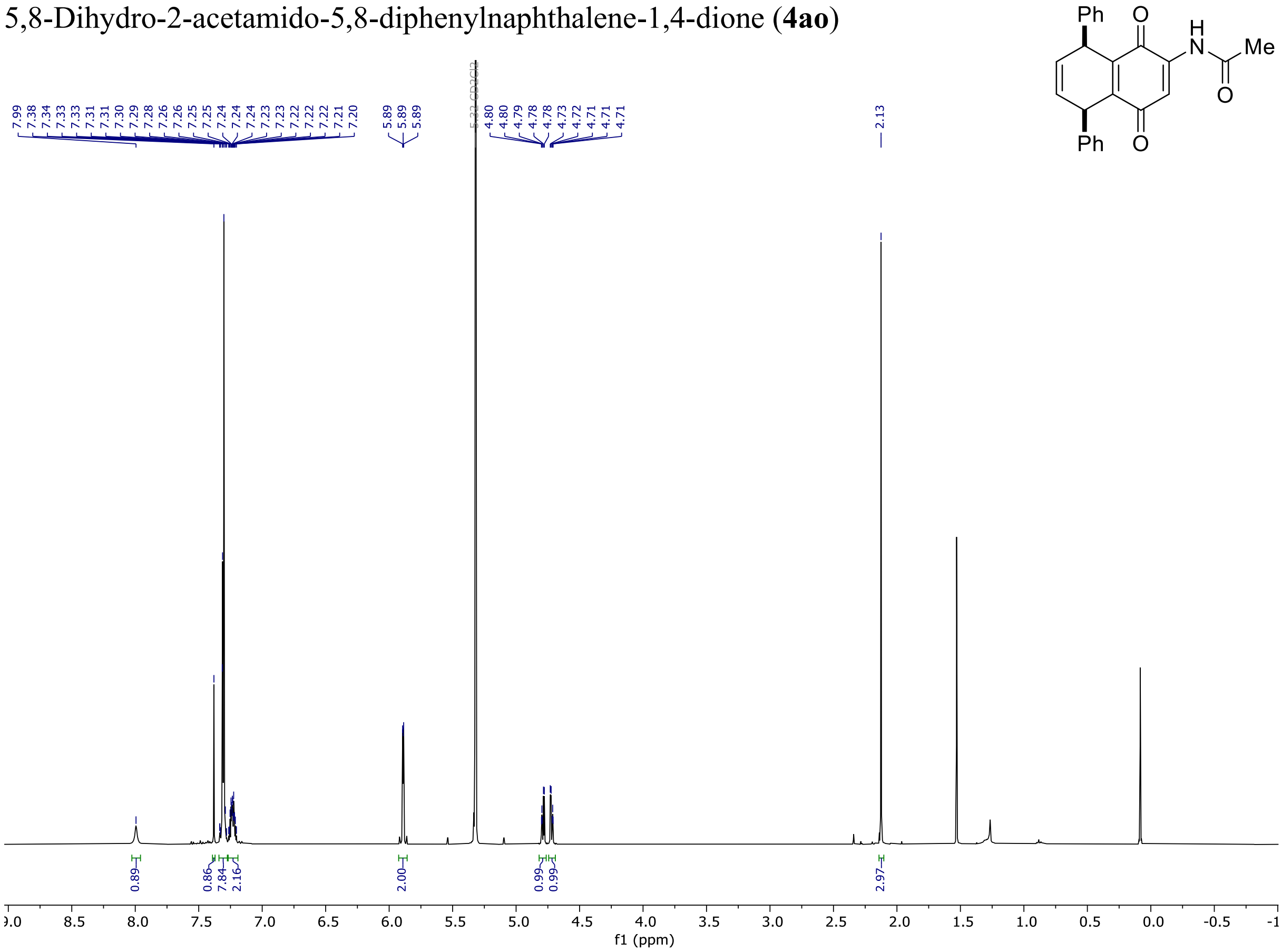

^{1}H NMR spectrum (400 MHz, CD_2Cl_2) of **4ao**.

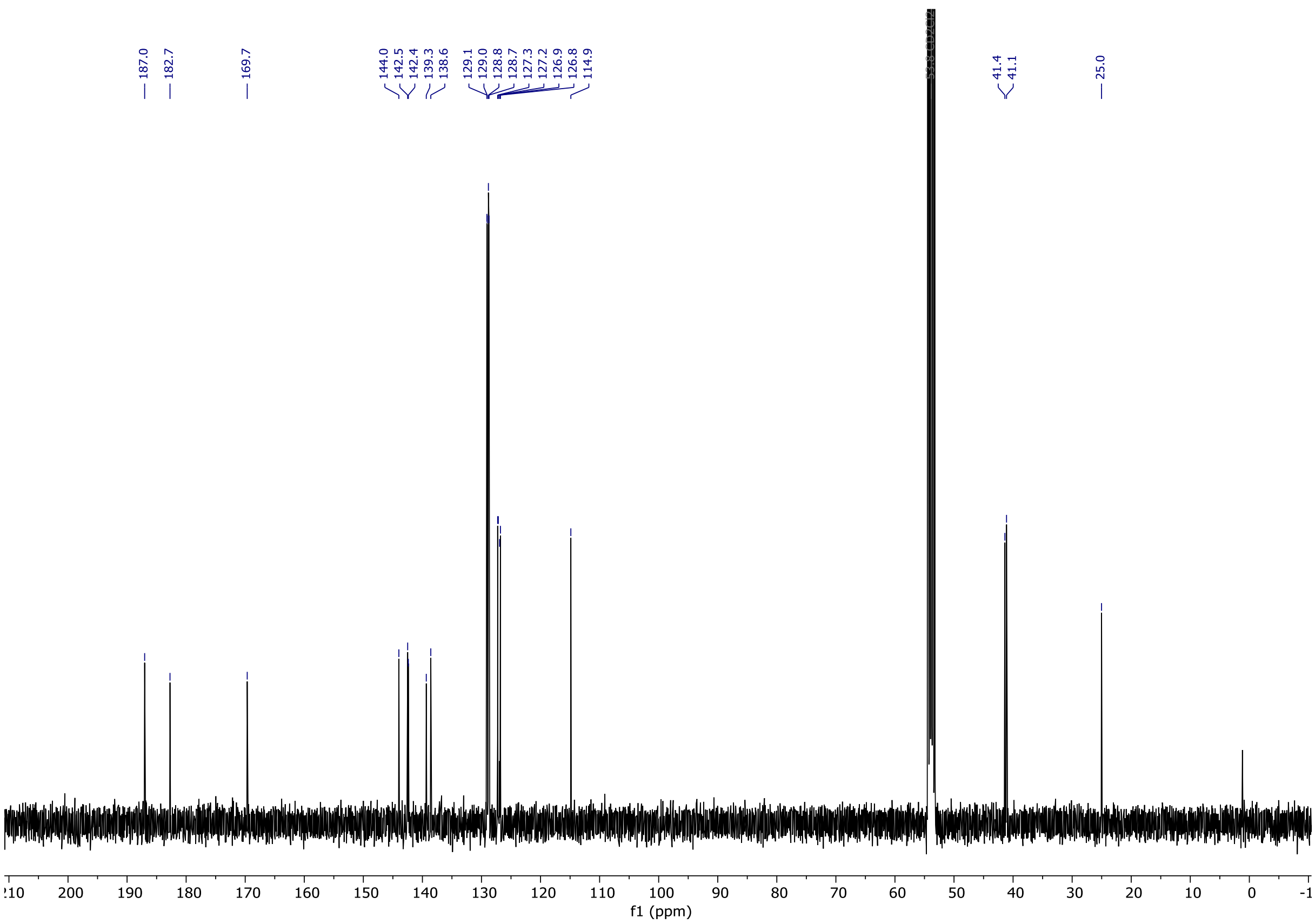

^{13}C NMR spectrum (101 MHz, CD_2Cl_2) of **4ao**.

1,4-Dihydro-1,4-diphenylanthracene-9,10-dione (**4ap**)

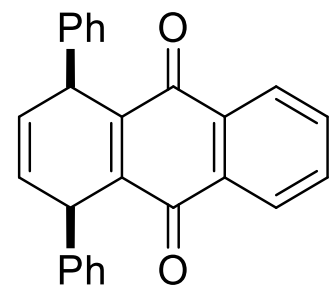

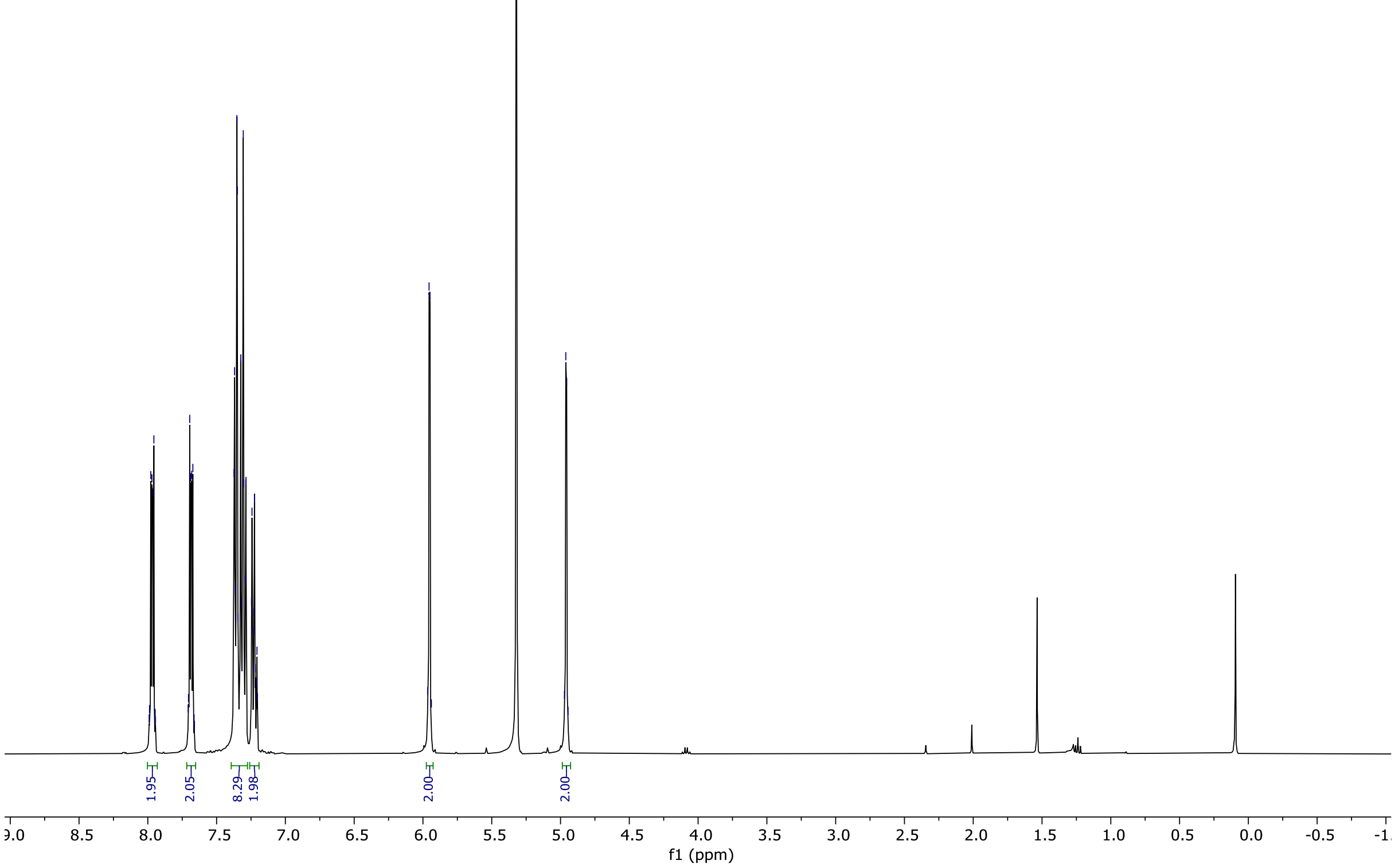

^{1}H NMR spectrum (400 MHz, CD_2Cl_2) of **4ap**.

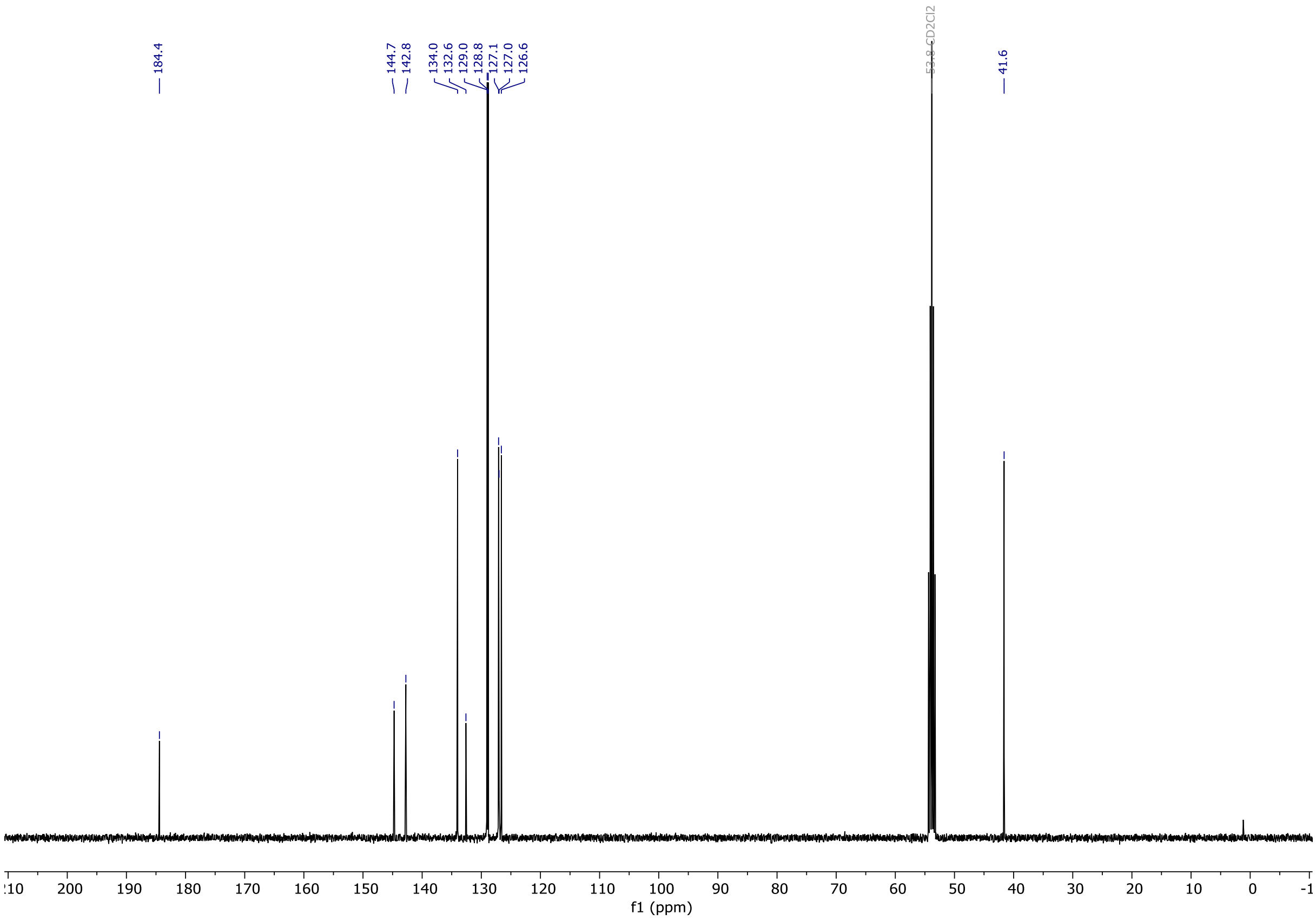

^{13}C NMR spectrum (101 MHz, CD_2Cl_2) of **4ap**.

5,8-Diphenylnaphthalene-1,4-dione (**5a**)

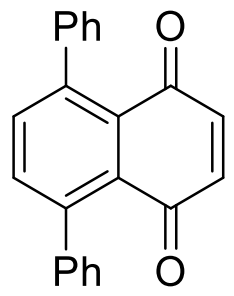

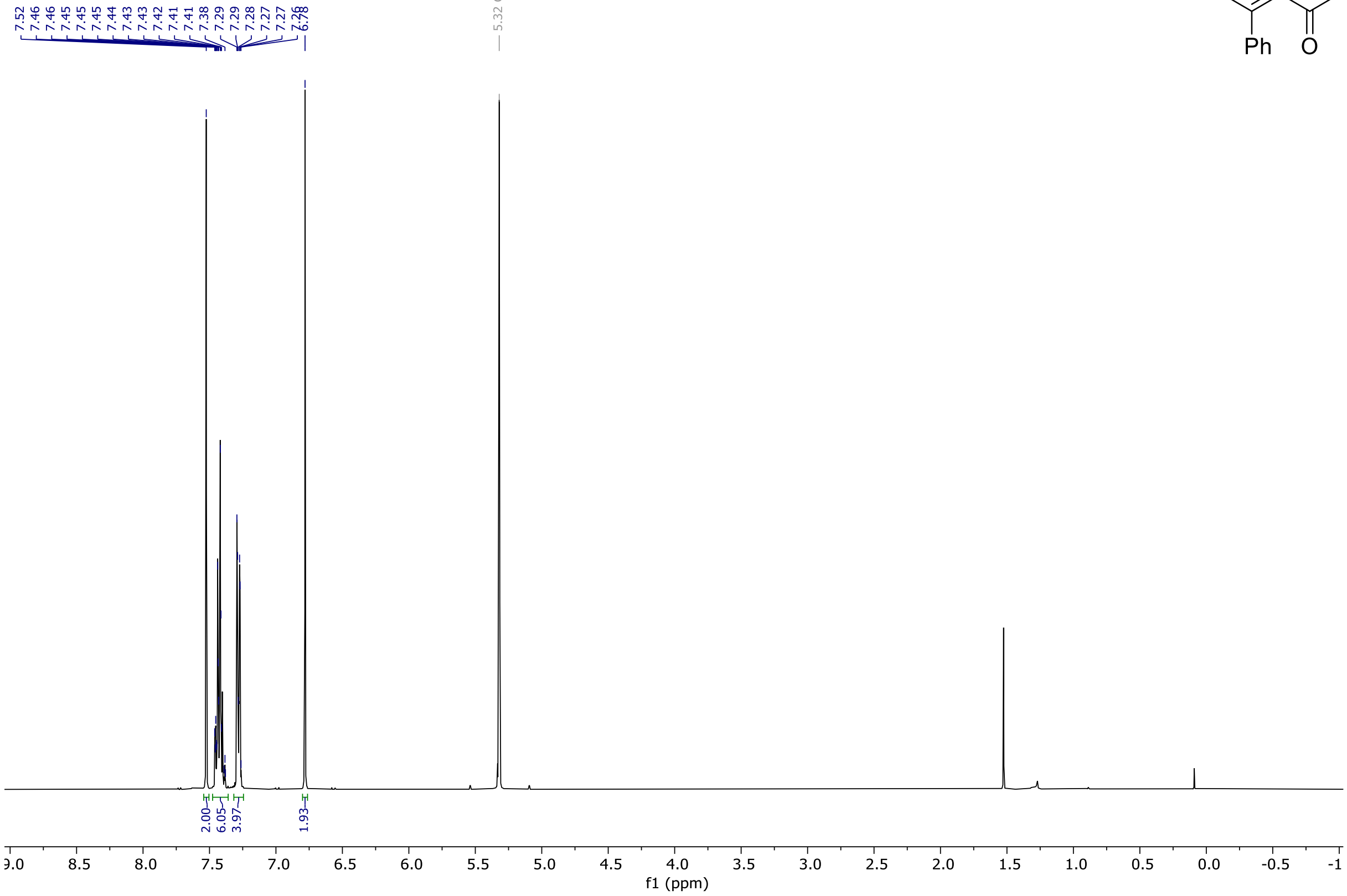

^1H NMR spectrum (400 MHz, CD_2Cl_2) of **5a**.

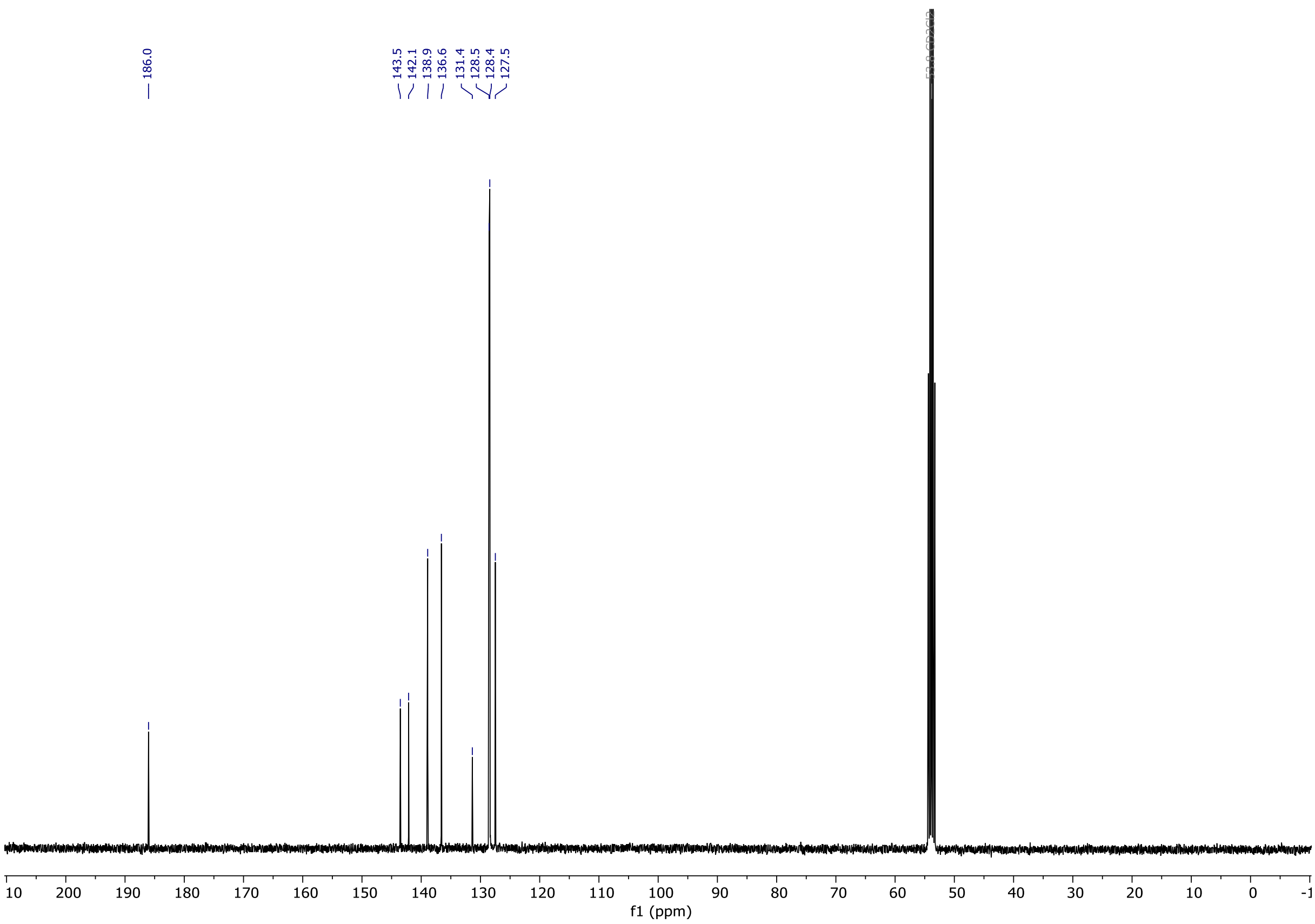

^{13}C NMR spectrum (101 MHz, CD_2Cl_2) of **5a**.

5-(4-Biphenyl)-8-phenylnaphthalene-1,4-dione (**5b**)

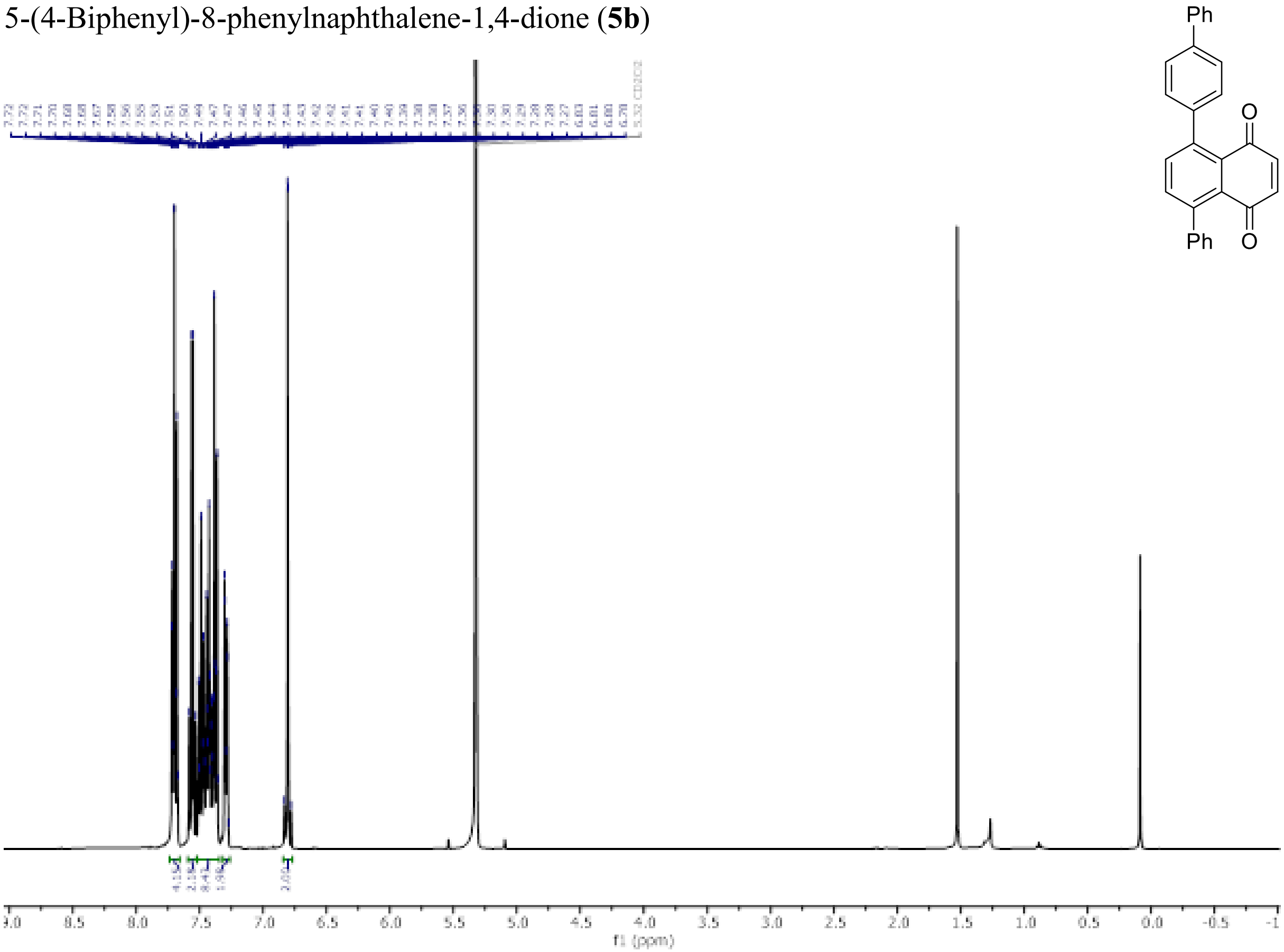

^{1}H NMR spectrum (400 MHz, CD_2Cl_2) of **5b**.

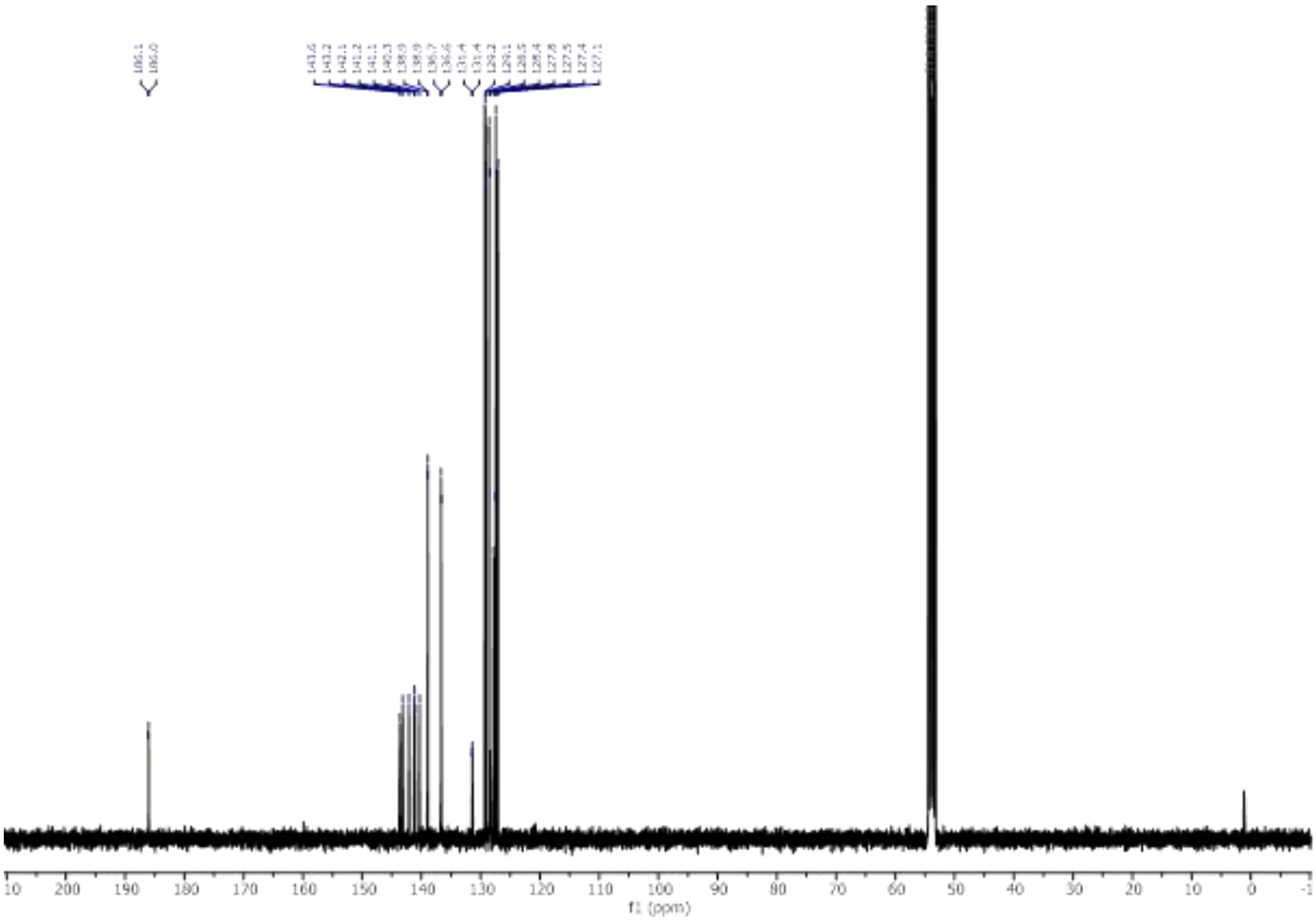

^{13}C NMR spectrum (101 MHz, CD_2Cl_2) of **5b**.

5-(2-Methylphenyl)-8-phenylnaphthalene-1,4-dione (**5c**)

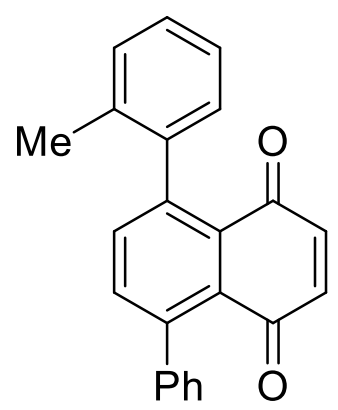

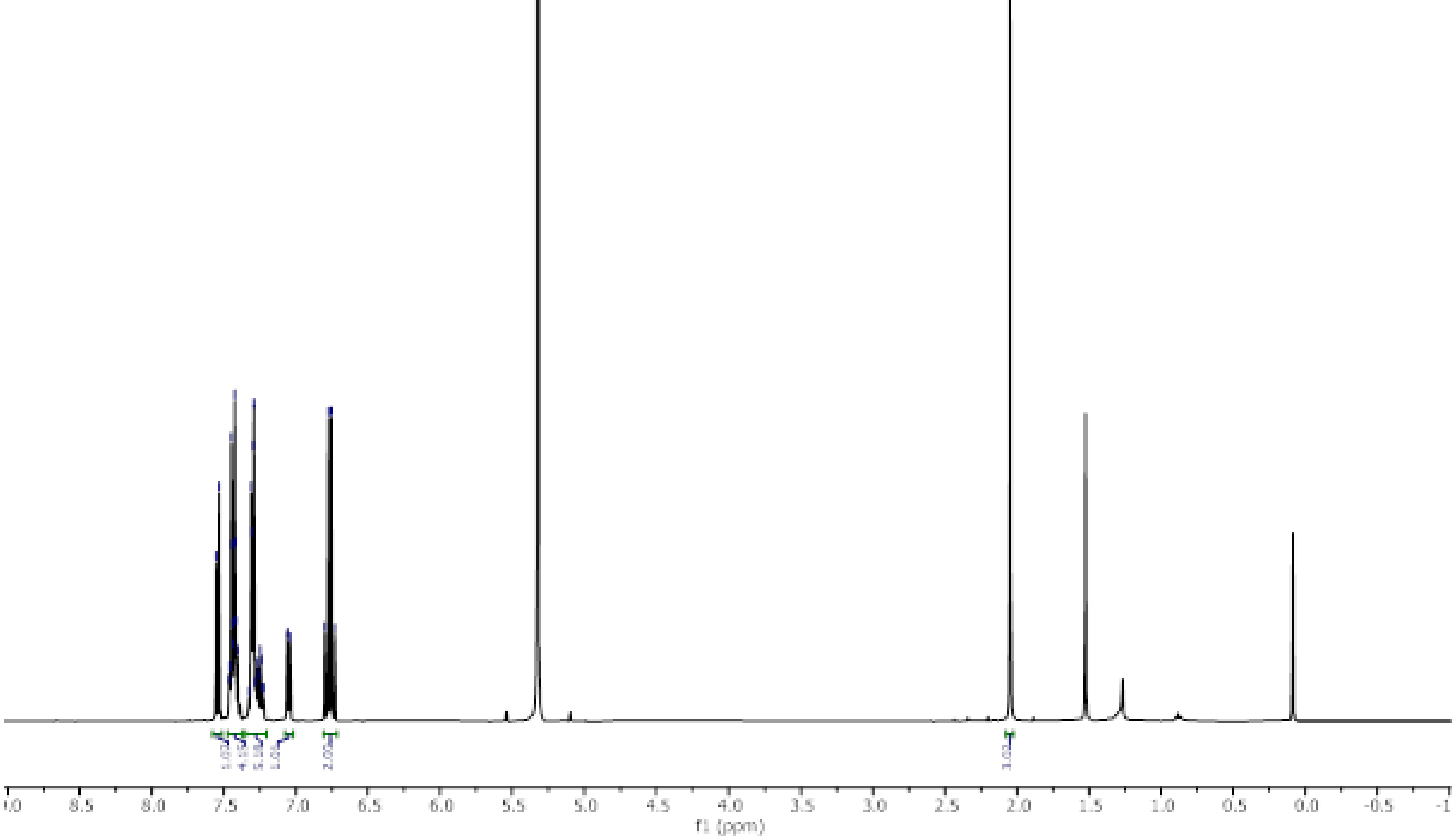

^{1}H NMR spectrum (400 MHz, CD_2Cl_2) of **5c**.

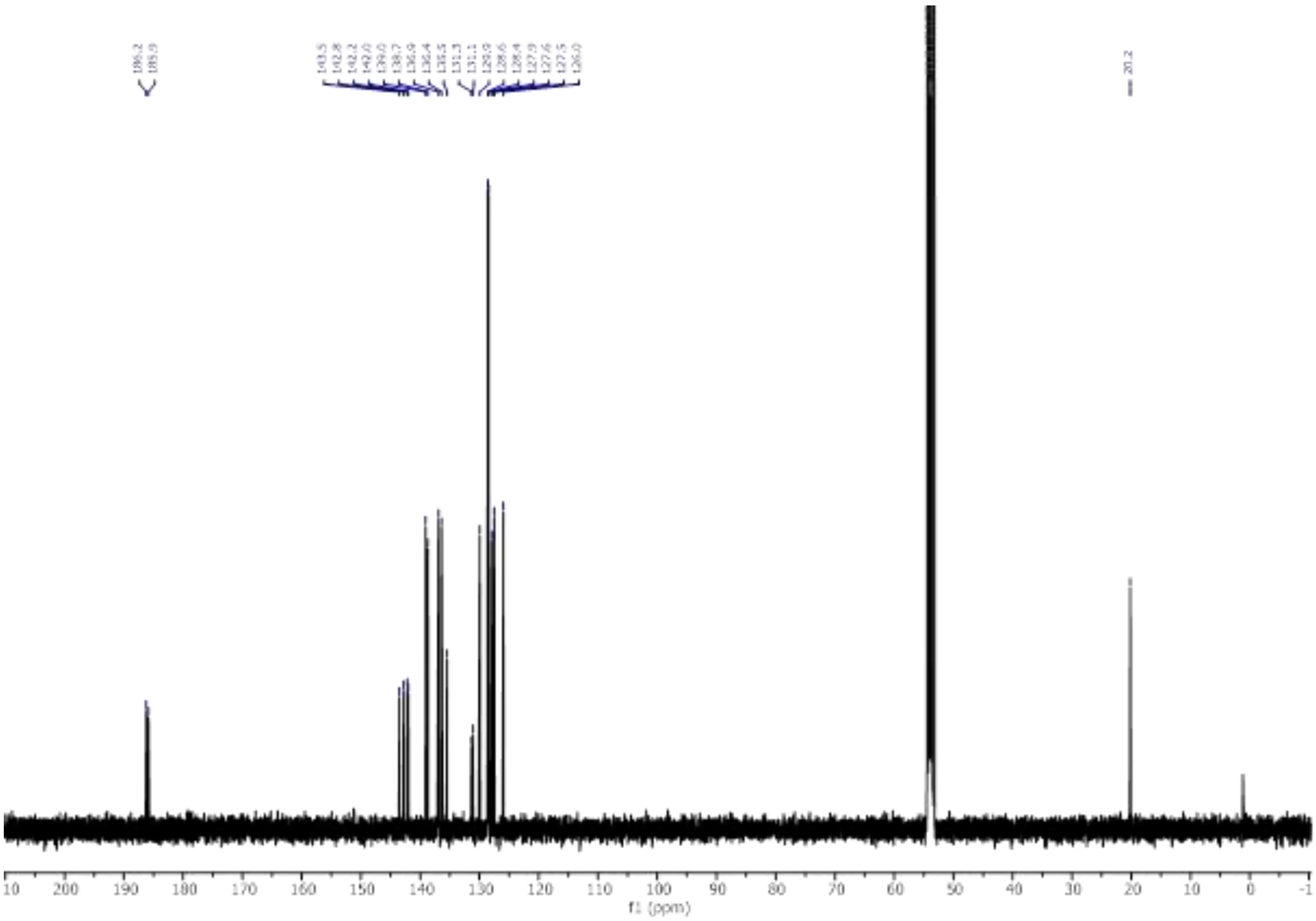

^{13}C NMR spectrum (101 MHz, CD_2Cl_2) of **5c**.

5-(3-Methylphenyl)-8-phenylnaphthalene-1,4-dione (**5d**)

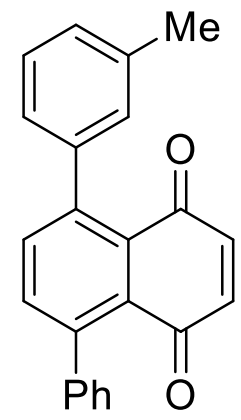

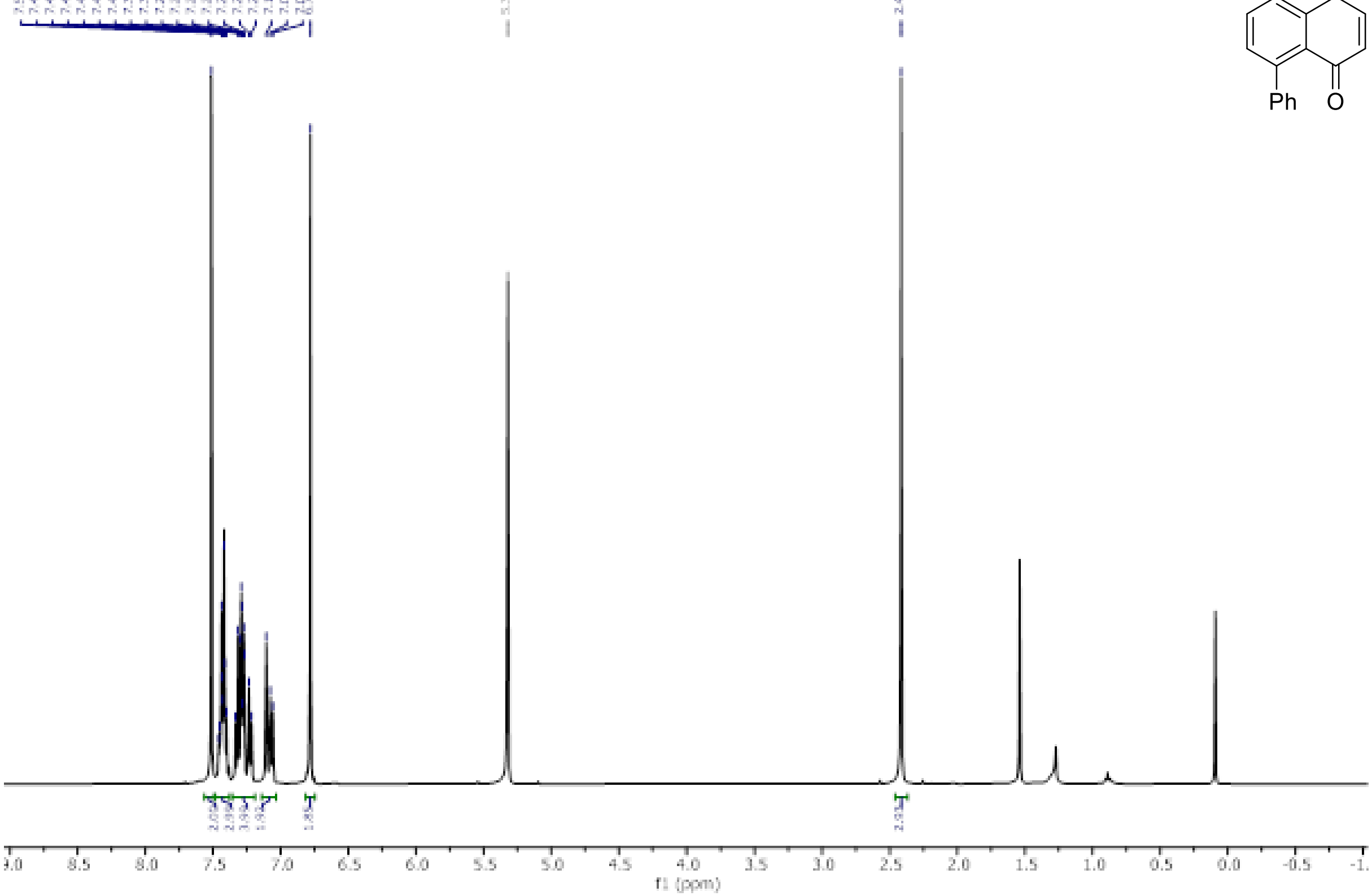

^{1}H NMR spectrum (400 MHz, CD_2Cl_2) of **5d**.

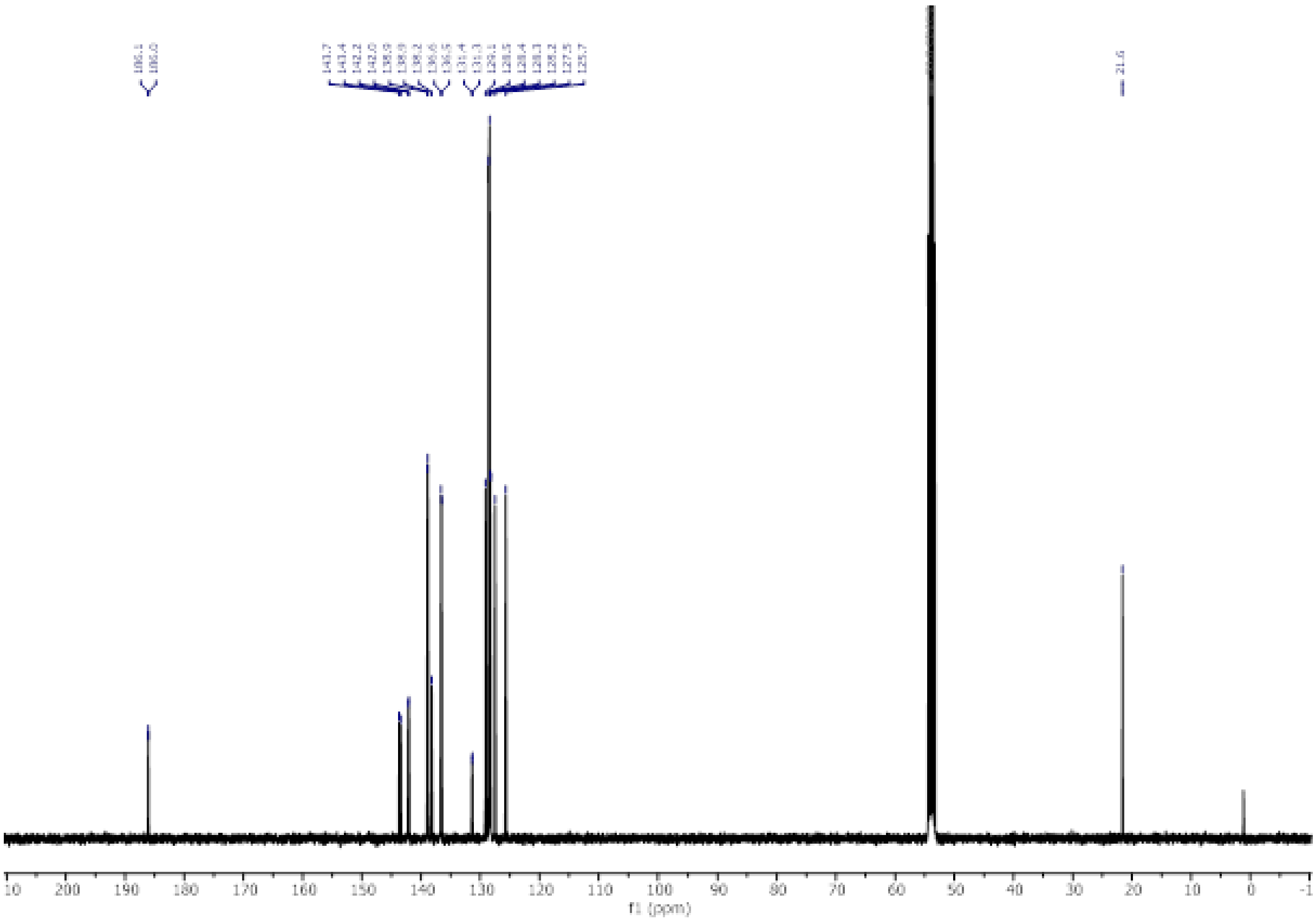

^{13}C NMR spectrum (101 MHz, CD_2Cl_2) of **5d**.

5-(4-Methylphenyl)-8-phenylnaphthalene-1,4-dione (**5e**)

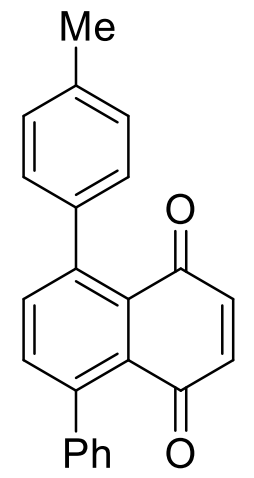

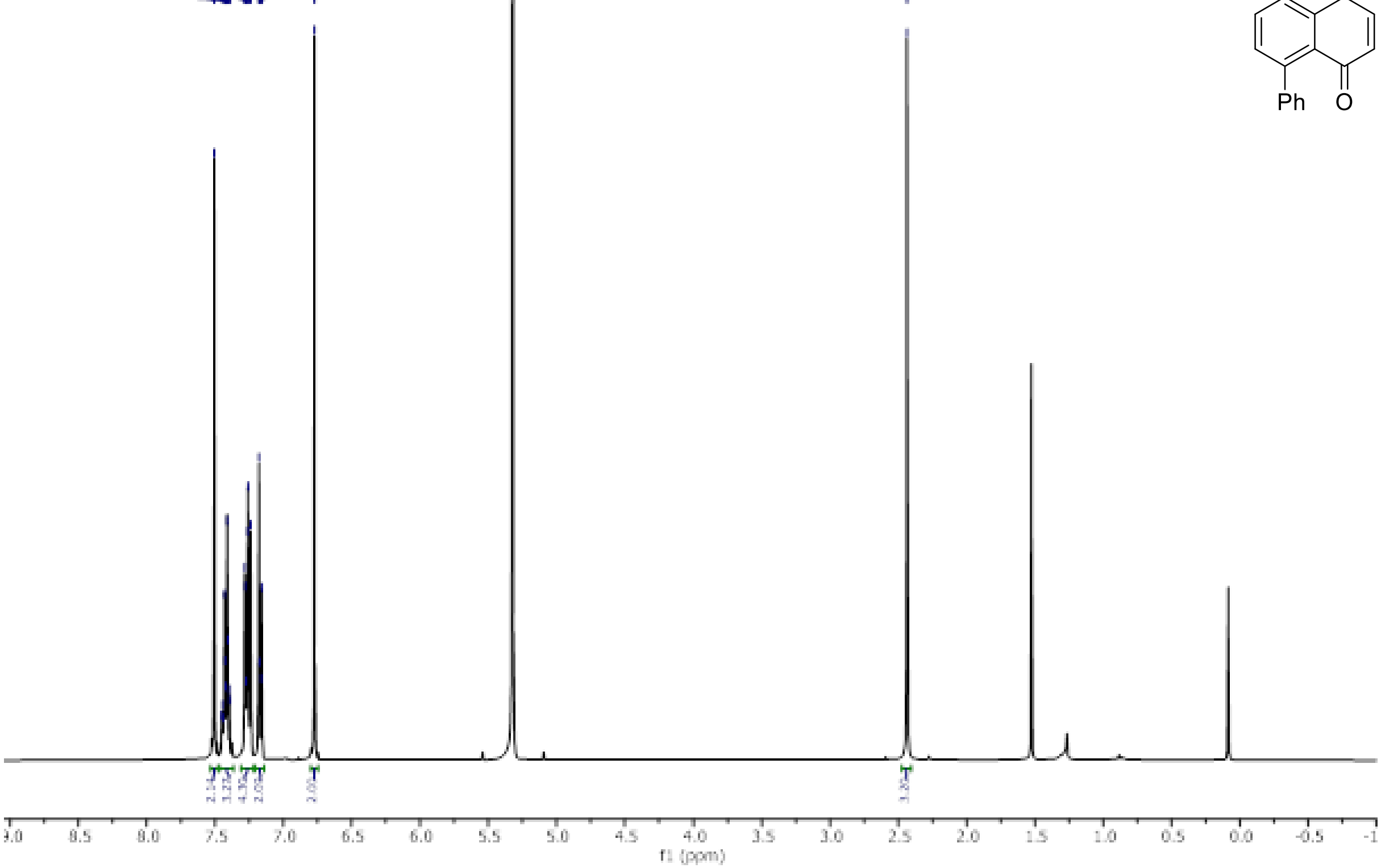

^{1}H NMR spectrum (400 MHz, CD_2Cl_2) of **5e**.

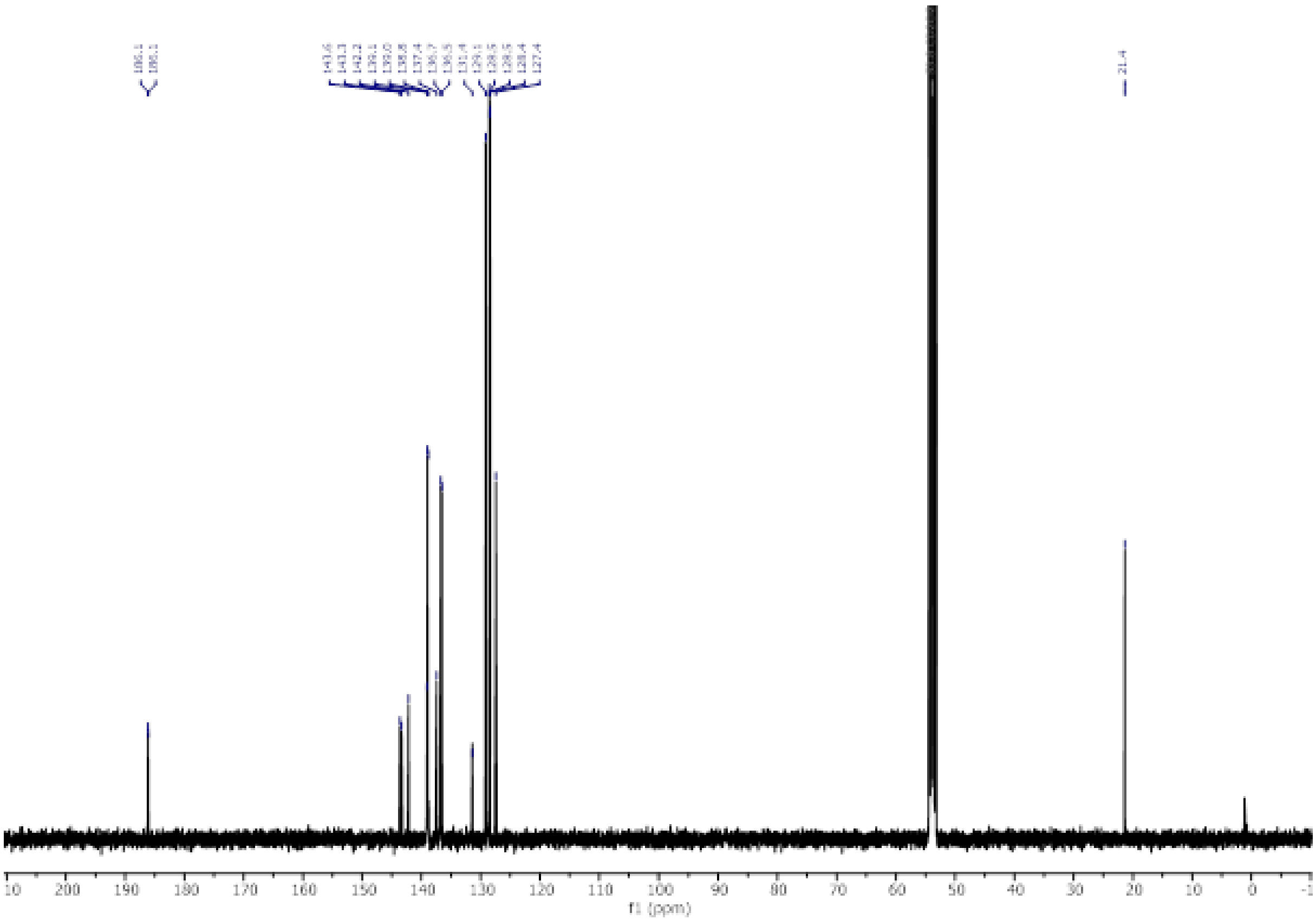

^{13}C NMR spectrum (101 MHz, CD_2Cl_2) of **5e**.

5-(4-Butylphenyl)-8-phenylnaphthalene-1,4-dione (**5f**)

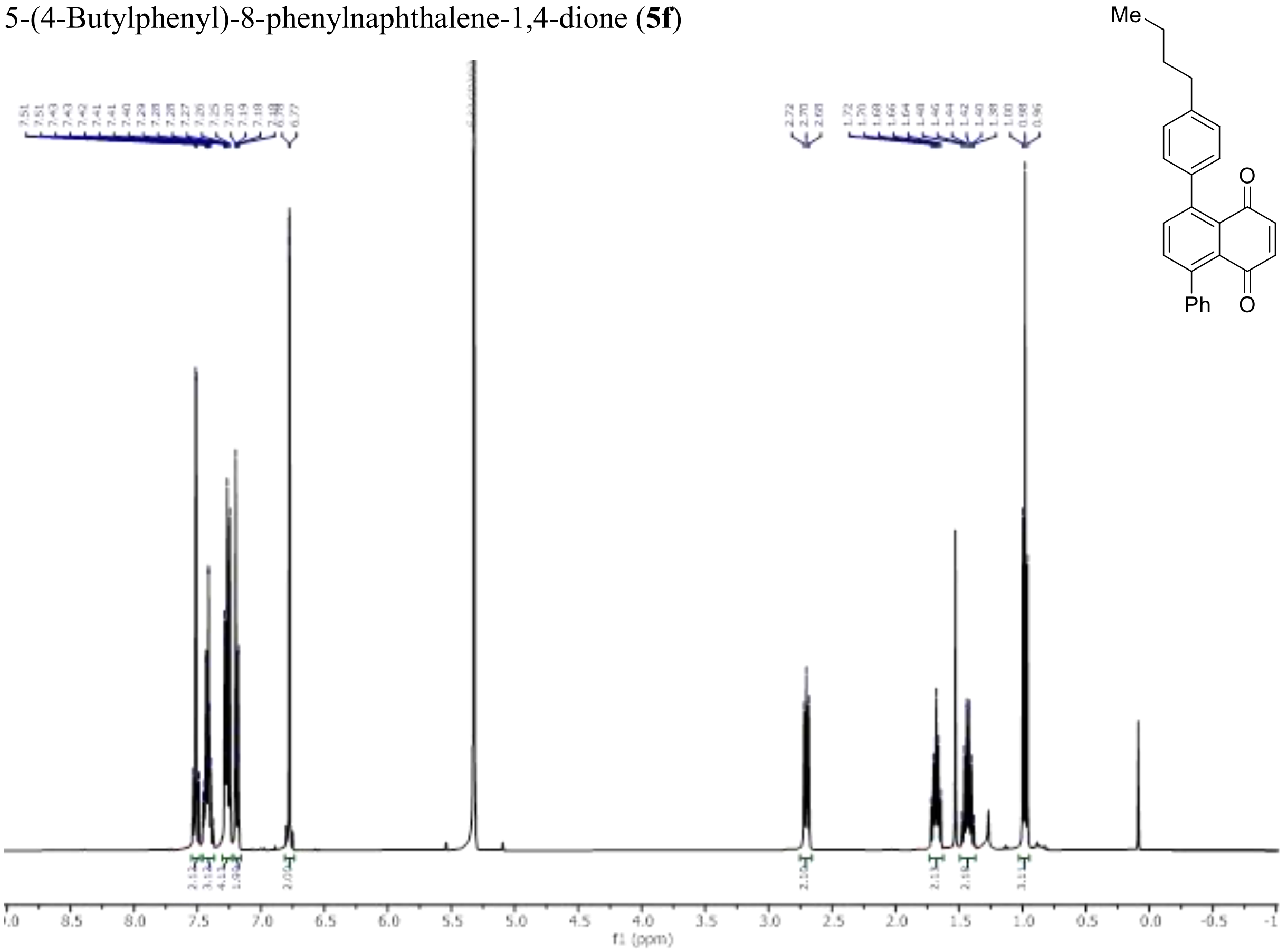

^{1}H NMR spectrum (400 MHz, CD_2Cl_2) of **5f**.

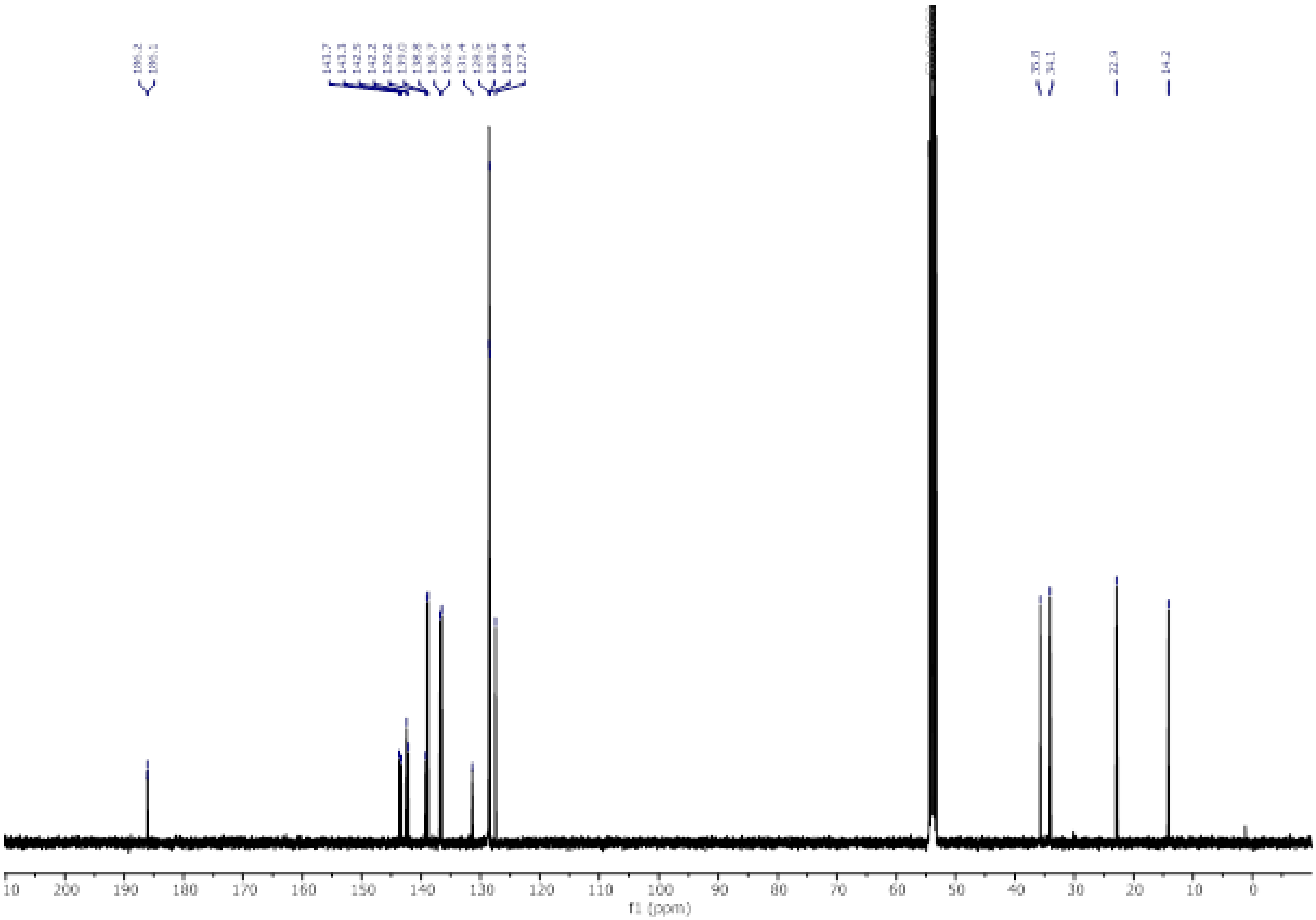

^{13}C NMR spectrum (101 MHz, CD_2Cl_2) of **5f**.

5-(4-tert-Butylphenyl)-8-phenylnaphthalene-1,4-dione (**5g**)

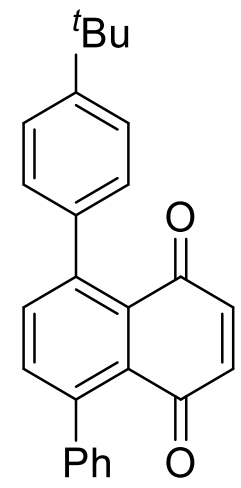

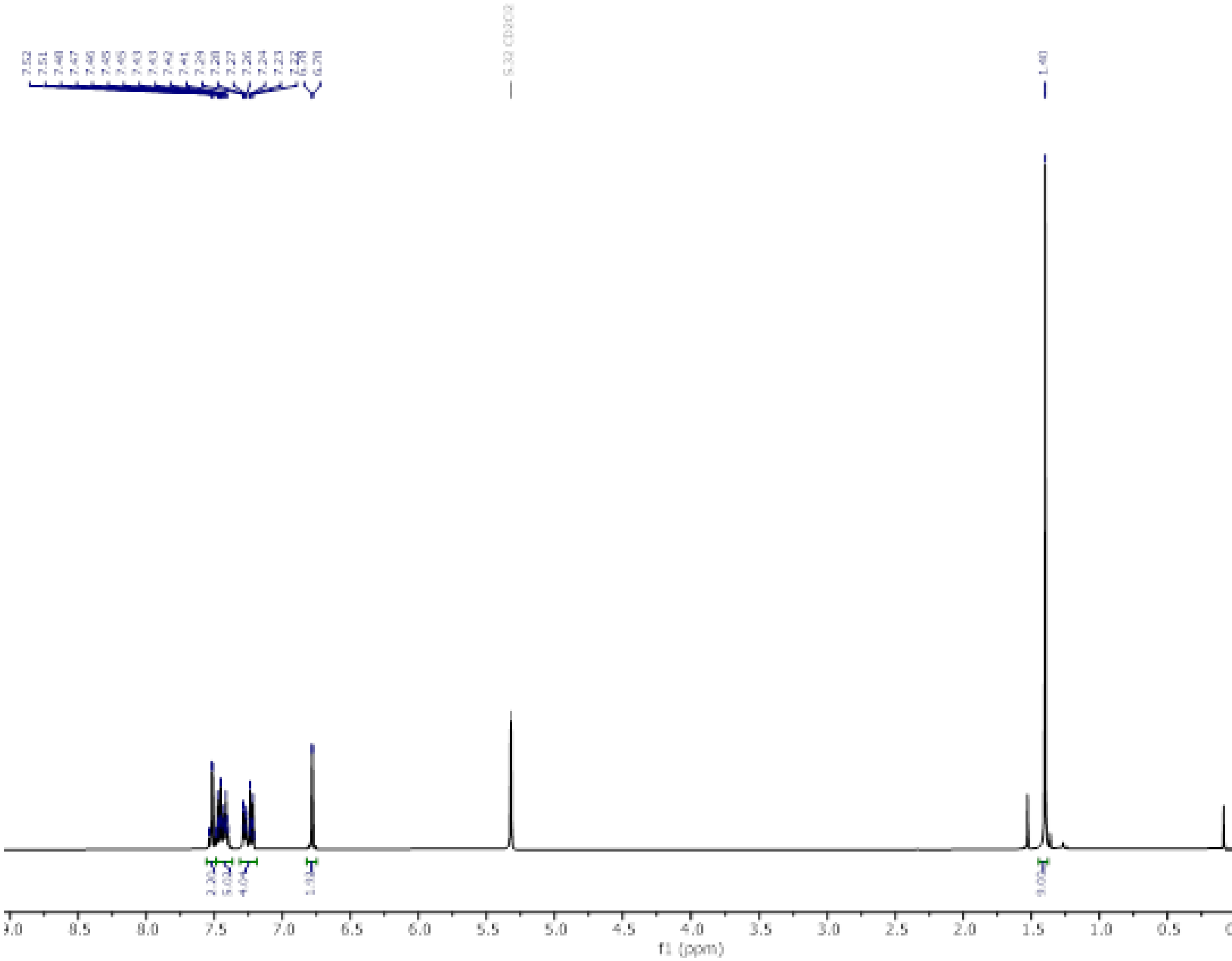

^{1}H NMR spectrum (400 MHz, CD_2Cl_2) of **5g**.

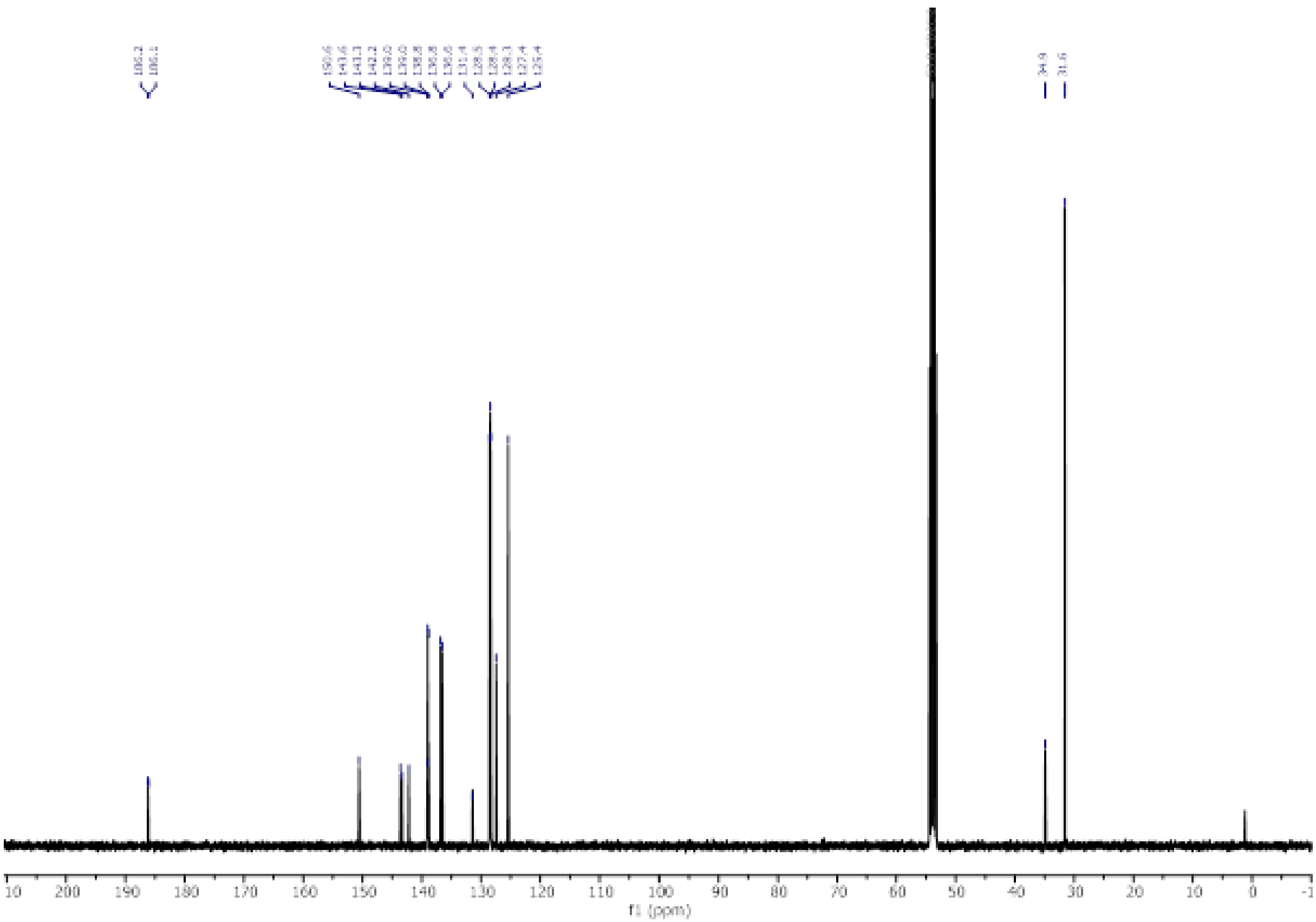

^{13}C NMR spectrum (101 MHz, CD_2Cl_2) of **5g**.

5-(2-Methoxyphenyl)-8-phenylnaphthalene-1,4-dione (**5h**)

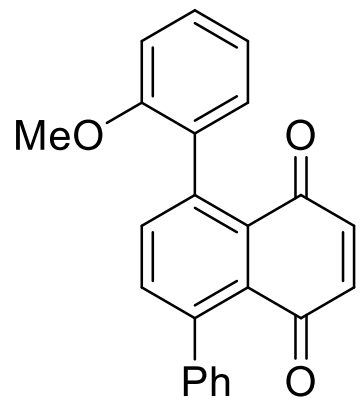

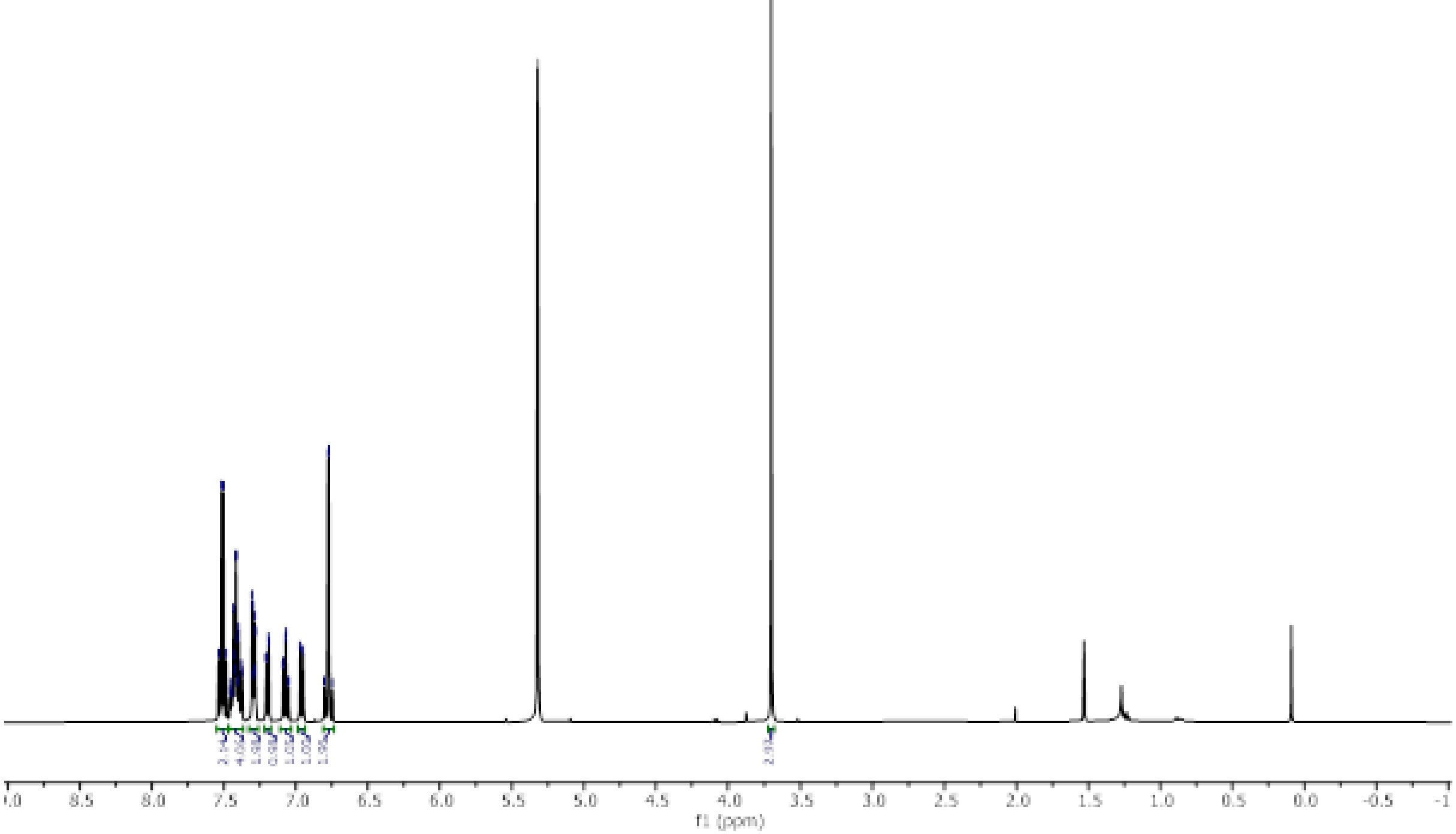

^{1}H NMR spectrum (400 MHz, CD_2Cl_2) of **5h**.

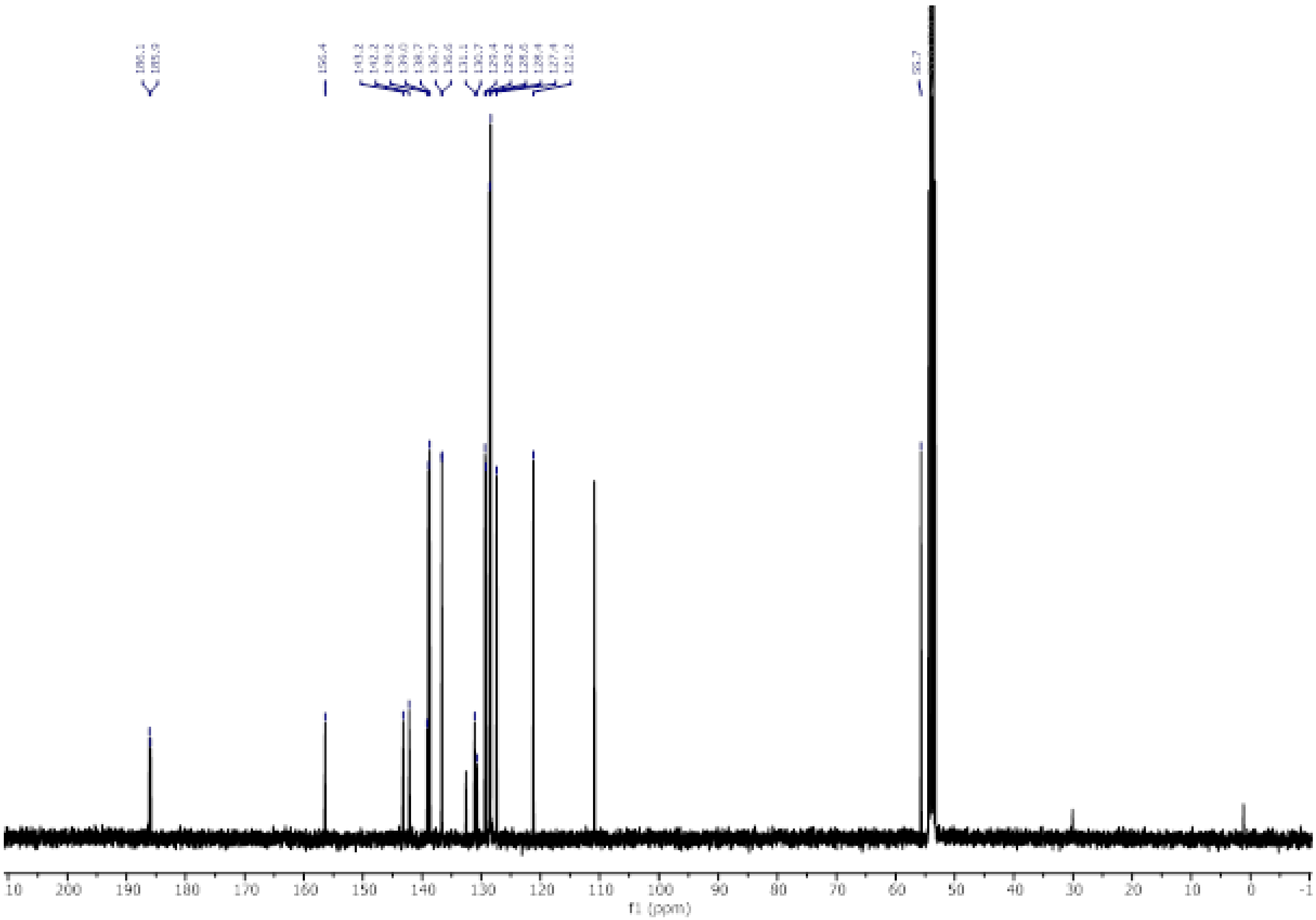

^{13}C NMR spectrum (101 MHz, CD_2Cl_2) of **5h**.

5-(3-Methoxyphenyl)-8-phenylnaphthalene-1,4-dione (**5i**)

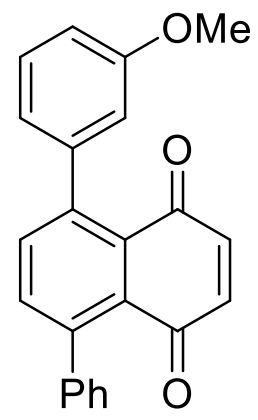

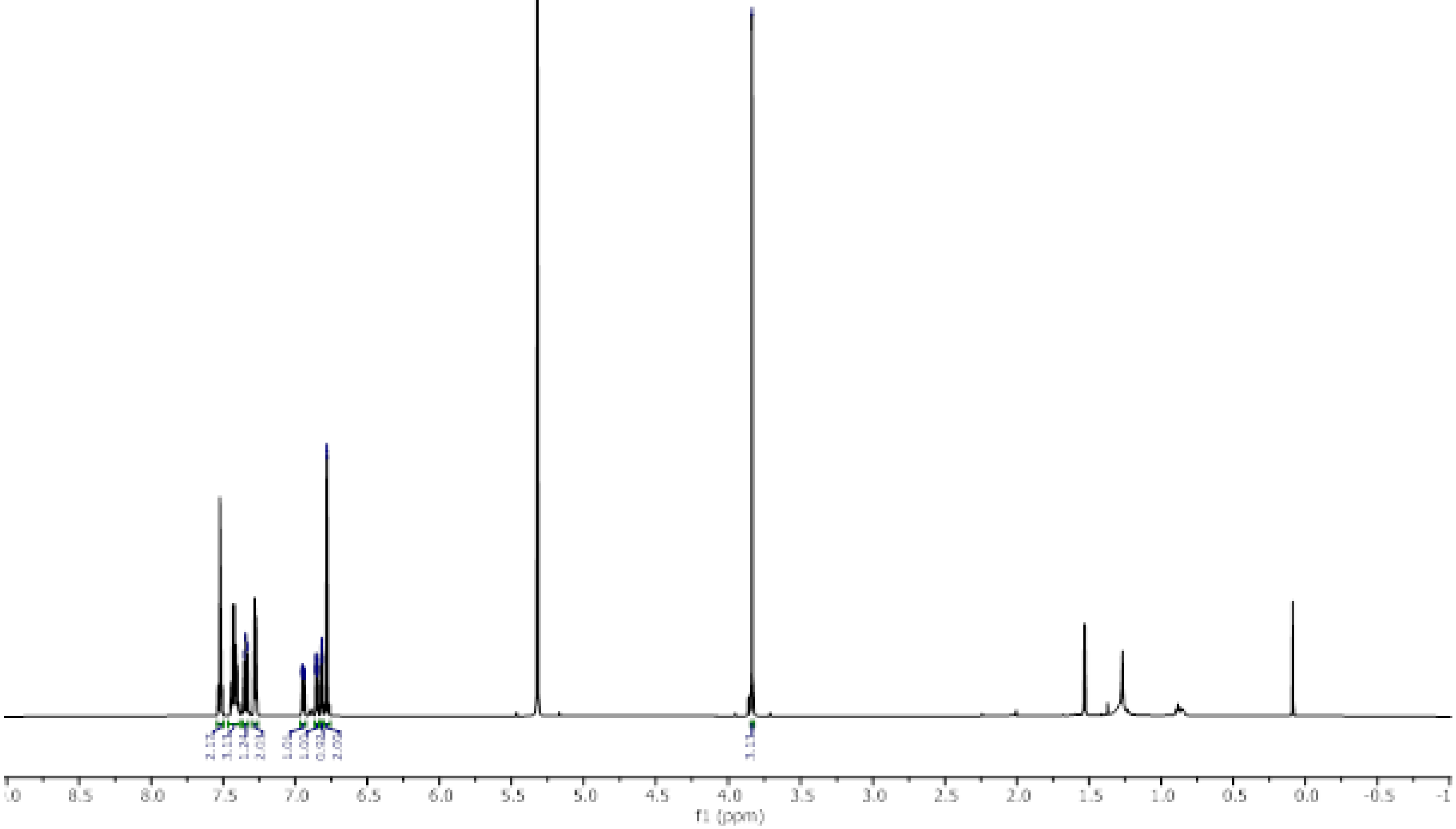

^{1}H NMR spectrum (600 MHz, CD_2Cl_2) of **5i**.

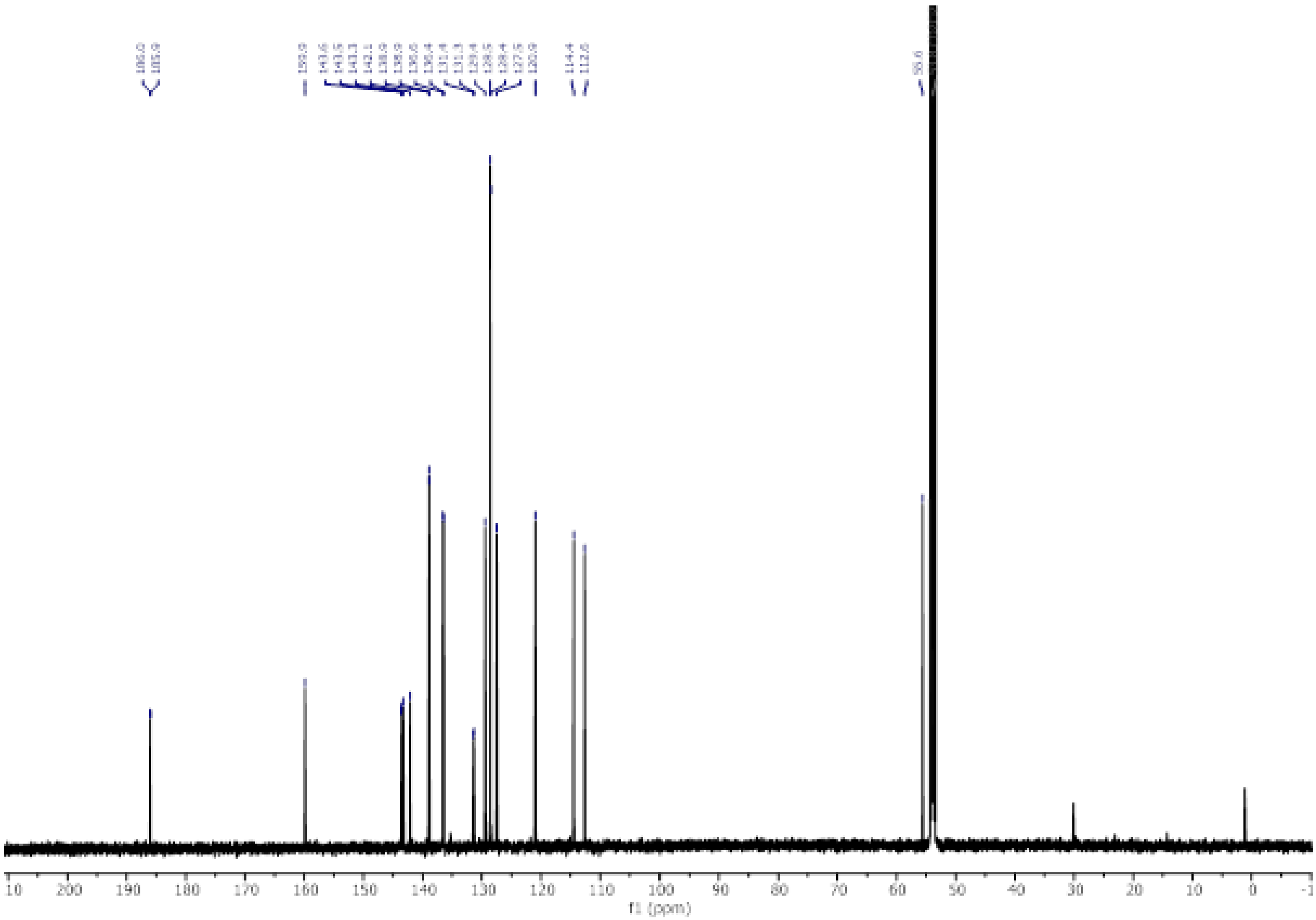

^{13}C NMR spectrum (151 MHz, CD_2Cl_2) of **5i**.

5-(4-Methoxyphenyl)-8-phenylnaphthalene-1,4-dione (**5j**)

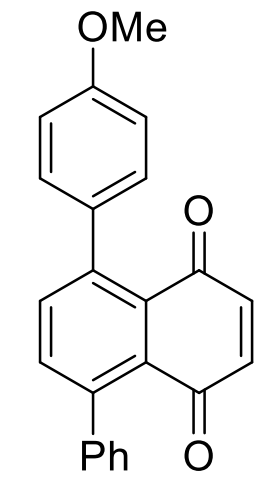

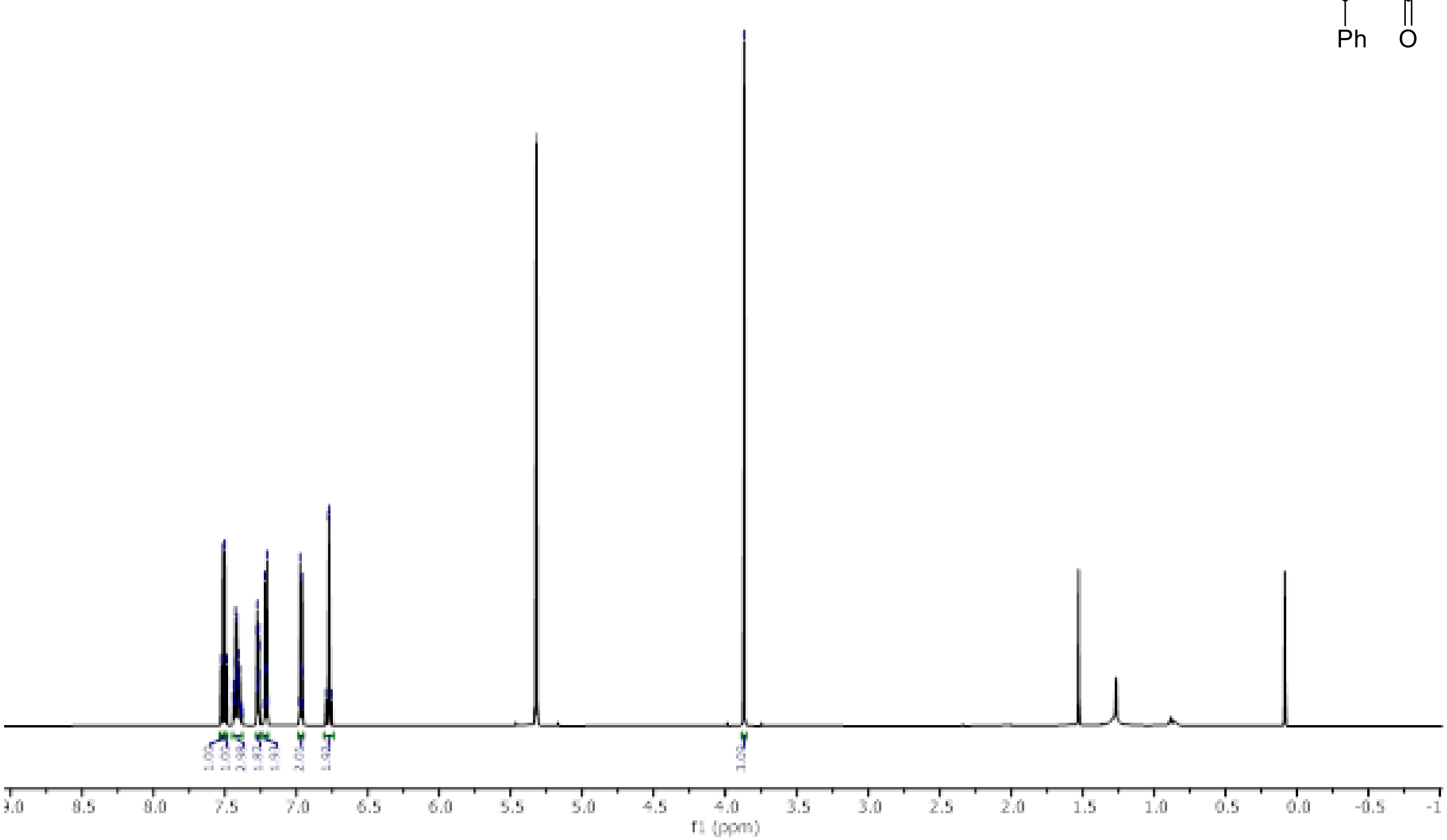

^{1}H NMR spectrum (400 MHz, CD_2Cl_2) of **5j**.

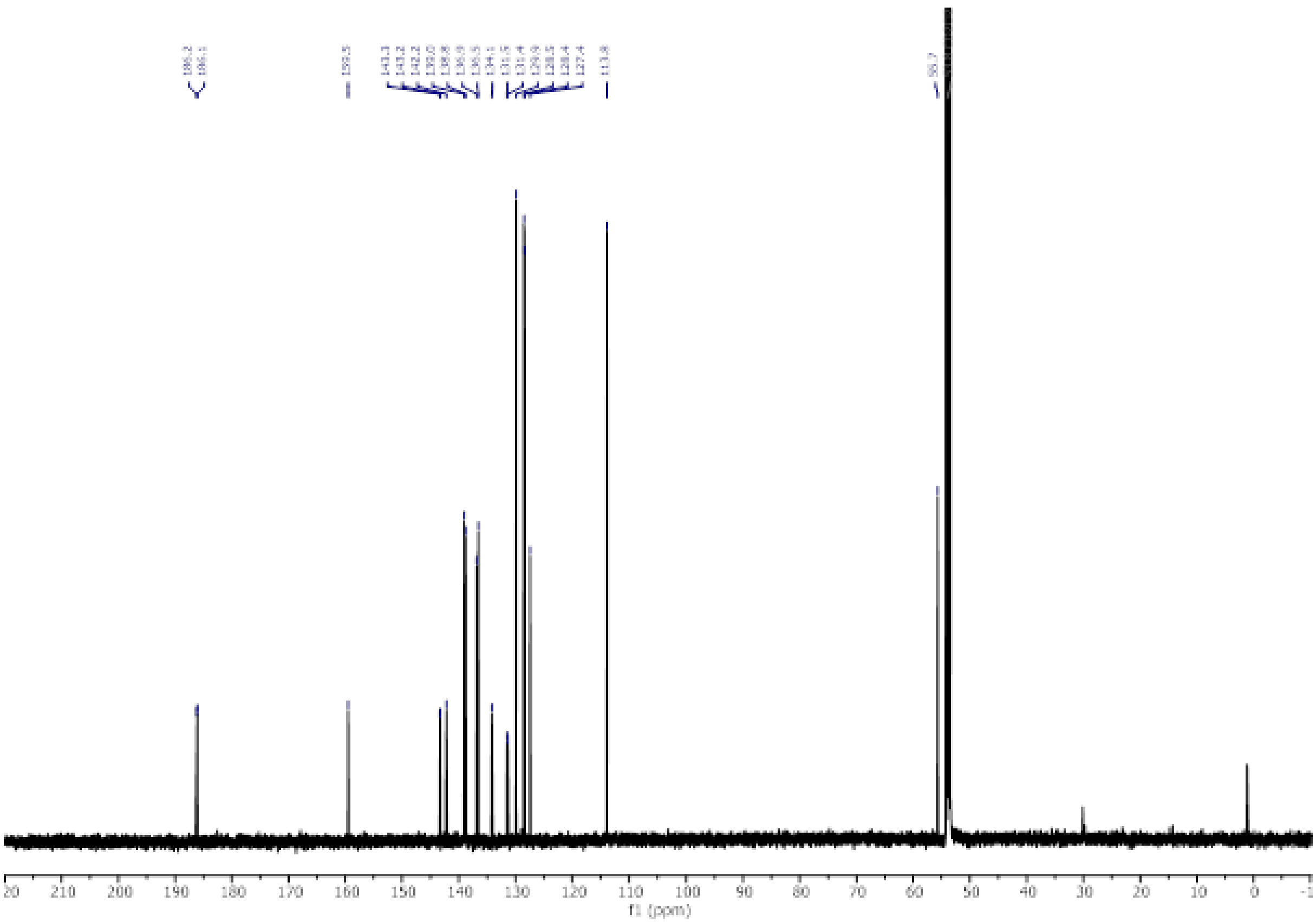

^{13}C NMR spectrum (101 MHz, CD_2Cl_2) of **5j**.

5-(1-Naphthyl)-8-phenylnaphthalene-1,4-dione (**5k**)

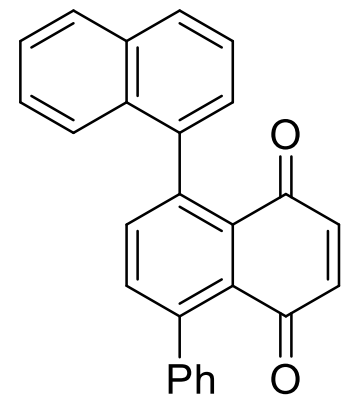

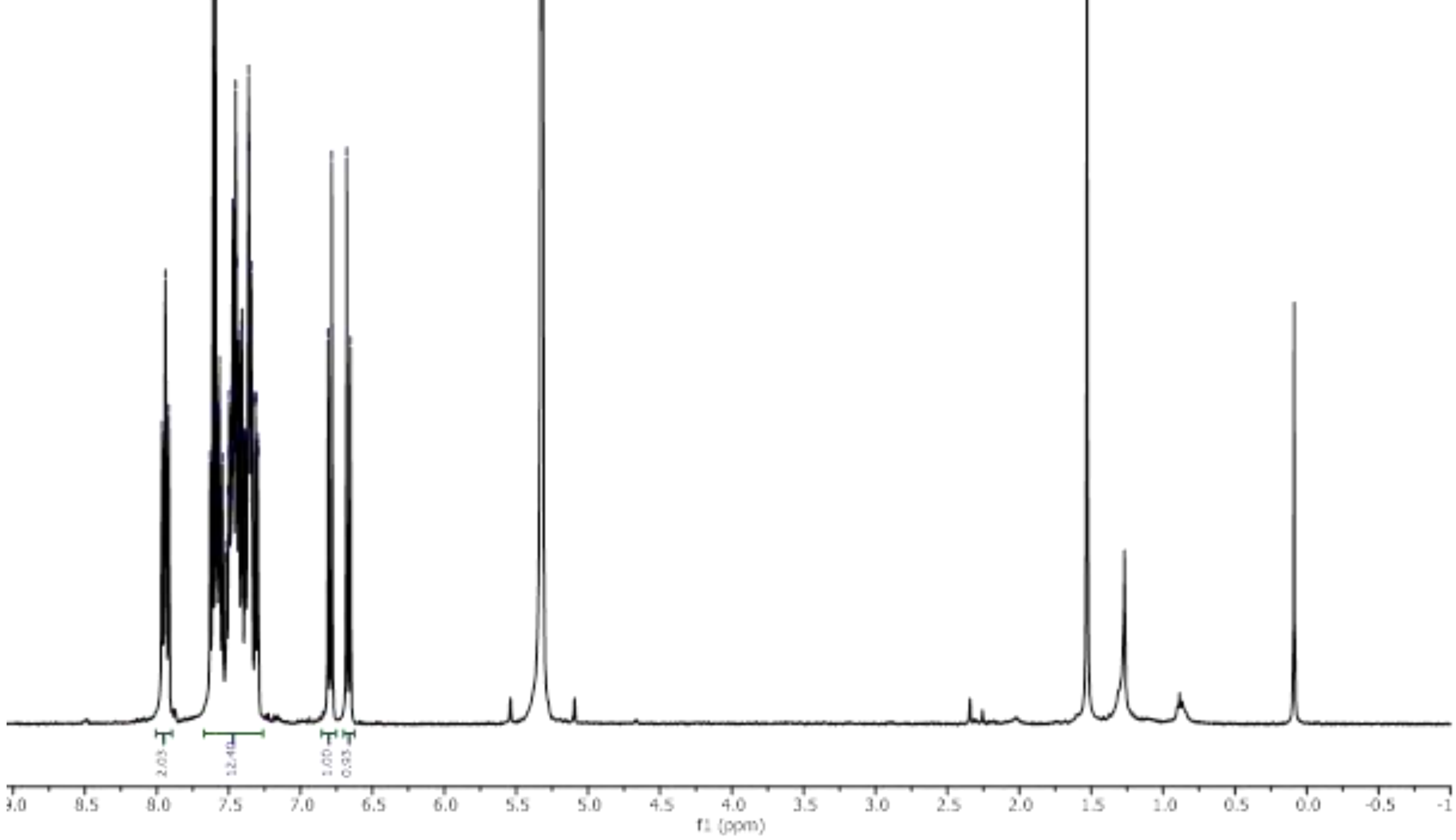

^{1}H NMR spectrum (400 MHz, CD_2Cl_2) of **5k**.

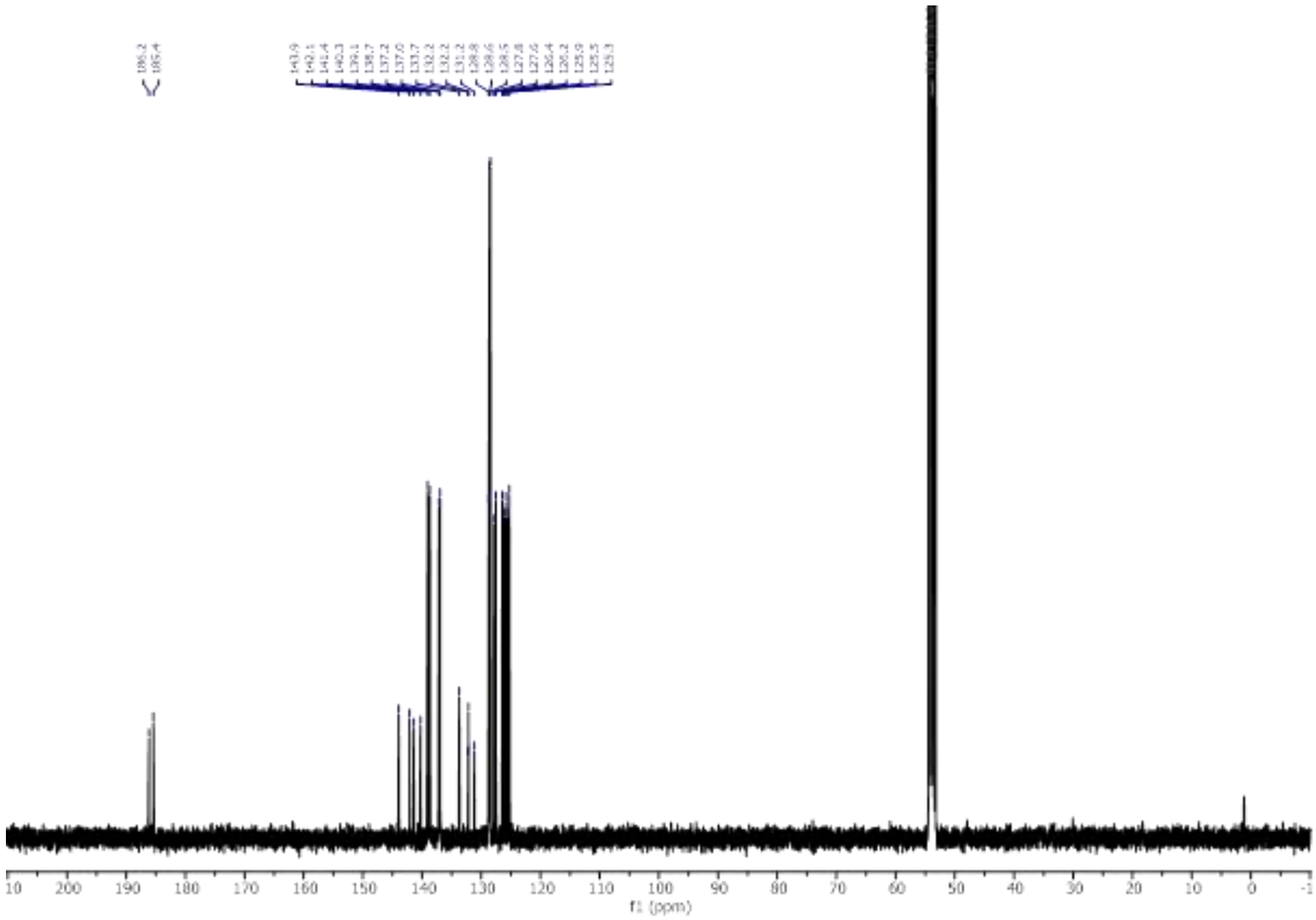

^{13}C NMR spectrum (101 MHz, CD_2Cl_2) of **5k**.

5-(2-Naphthyl)-8-phenylnaphthalene-1,4-dione (**5l**)

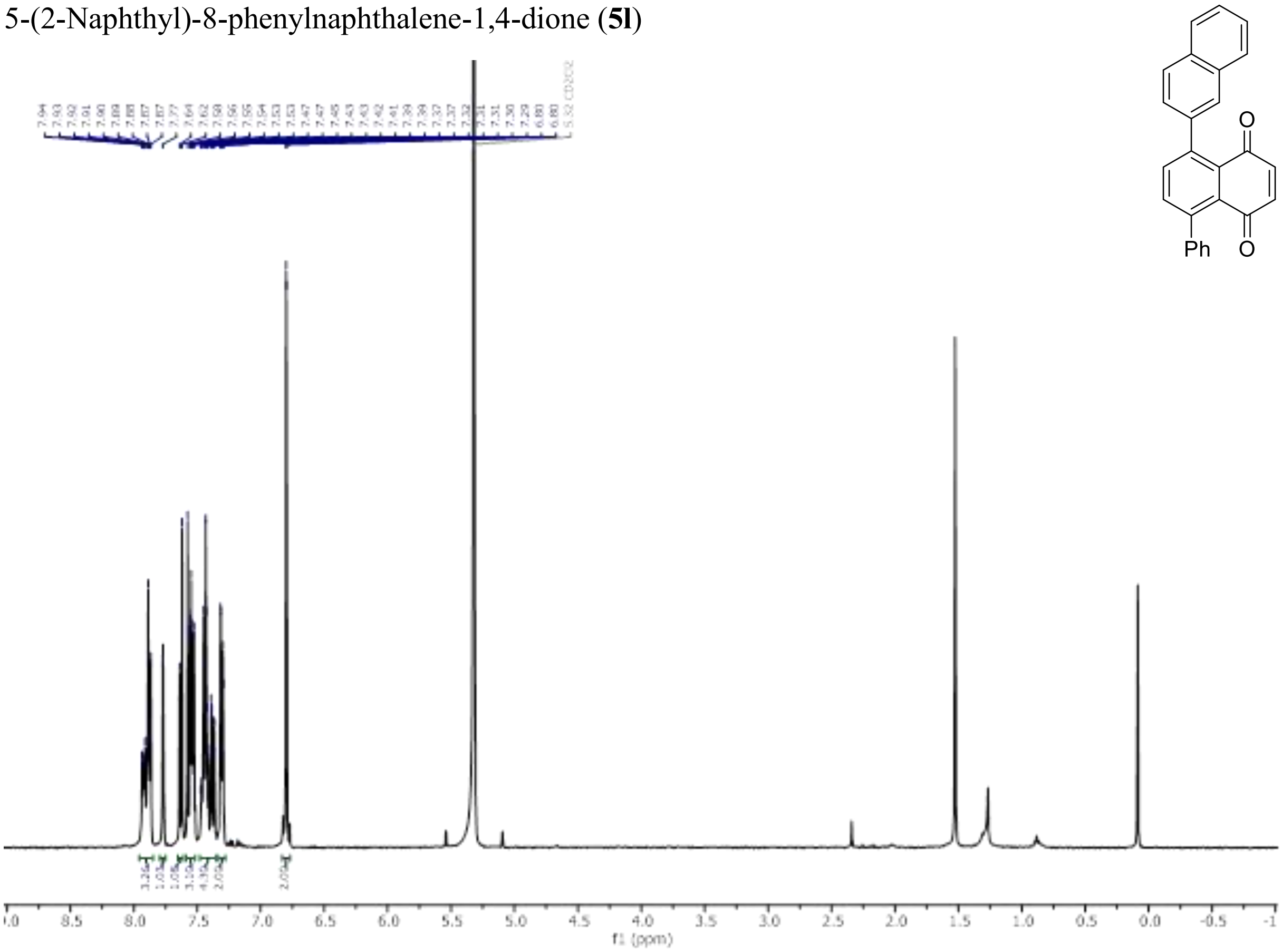

^{1}H NMR spectrum (400 MHz, CD_2Cl_2) of **5l**.

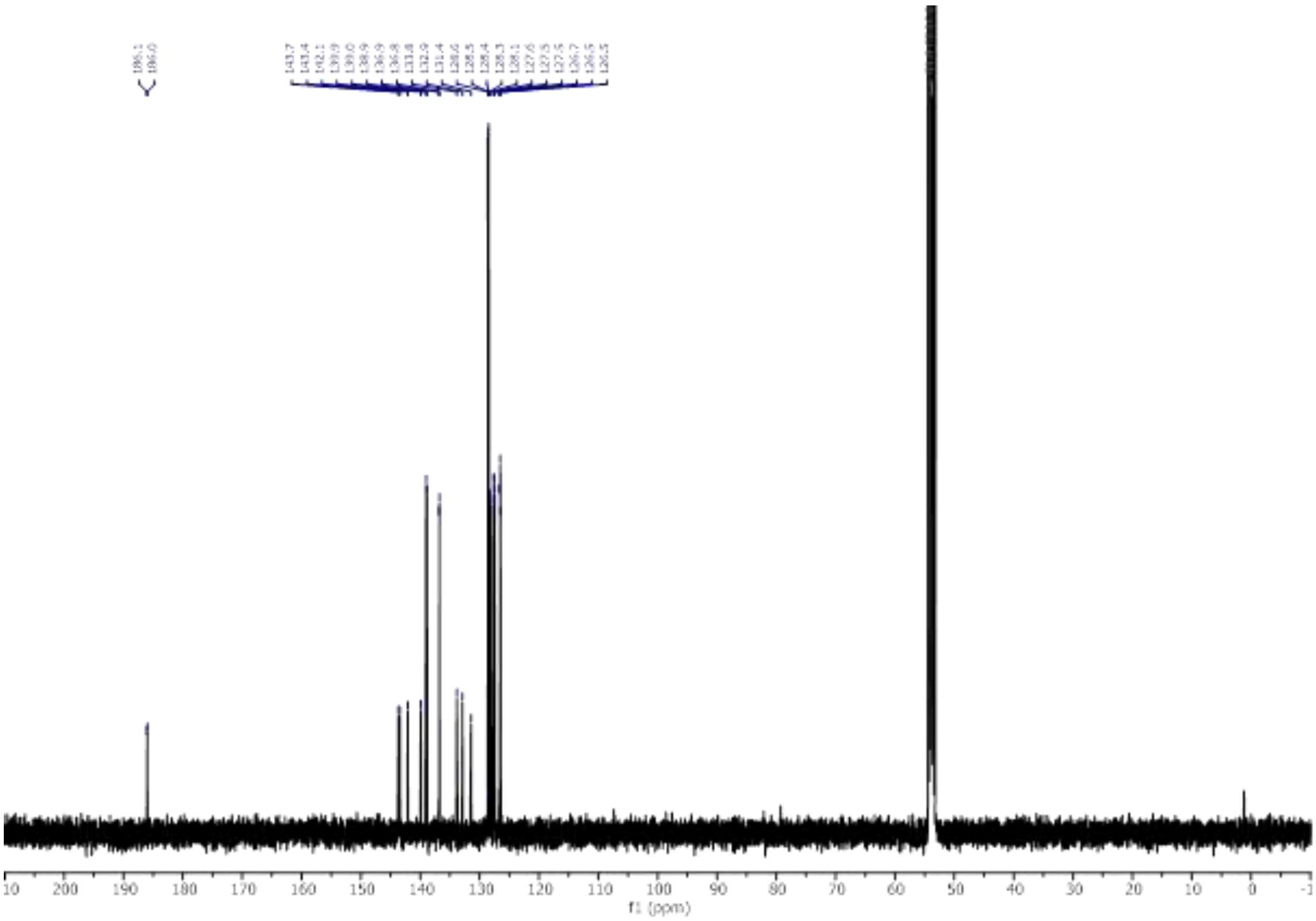

^{13}C NMR spectrum (101 MHz, CD_2Cl_2) of **5l**.

5-(2-Chlorophenyl)-8-phenylnaphthalene-1,4-dione (**5n**)

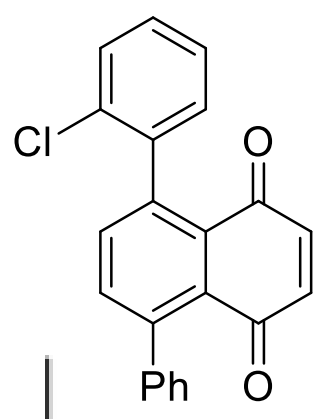

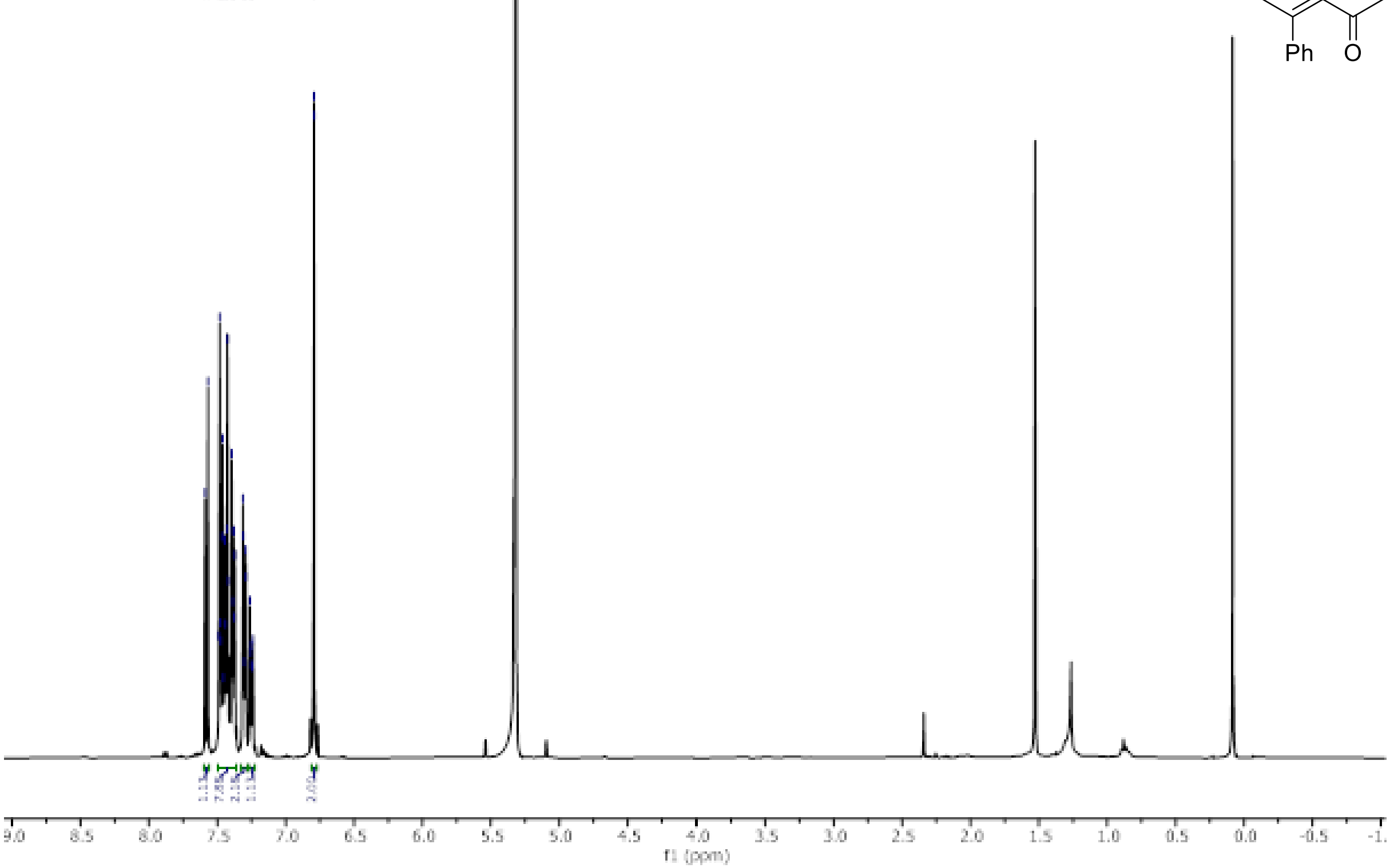

^{1}H NMR spectrum (400 MHz, CD_2Cl_2) of **5n**.

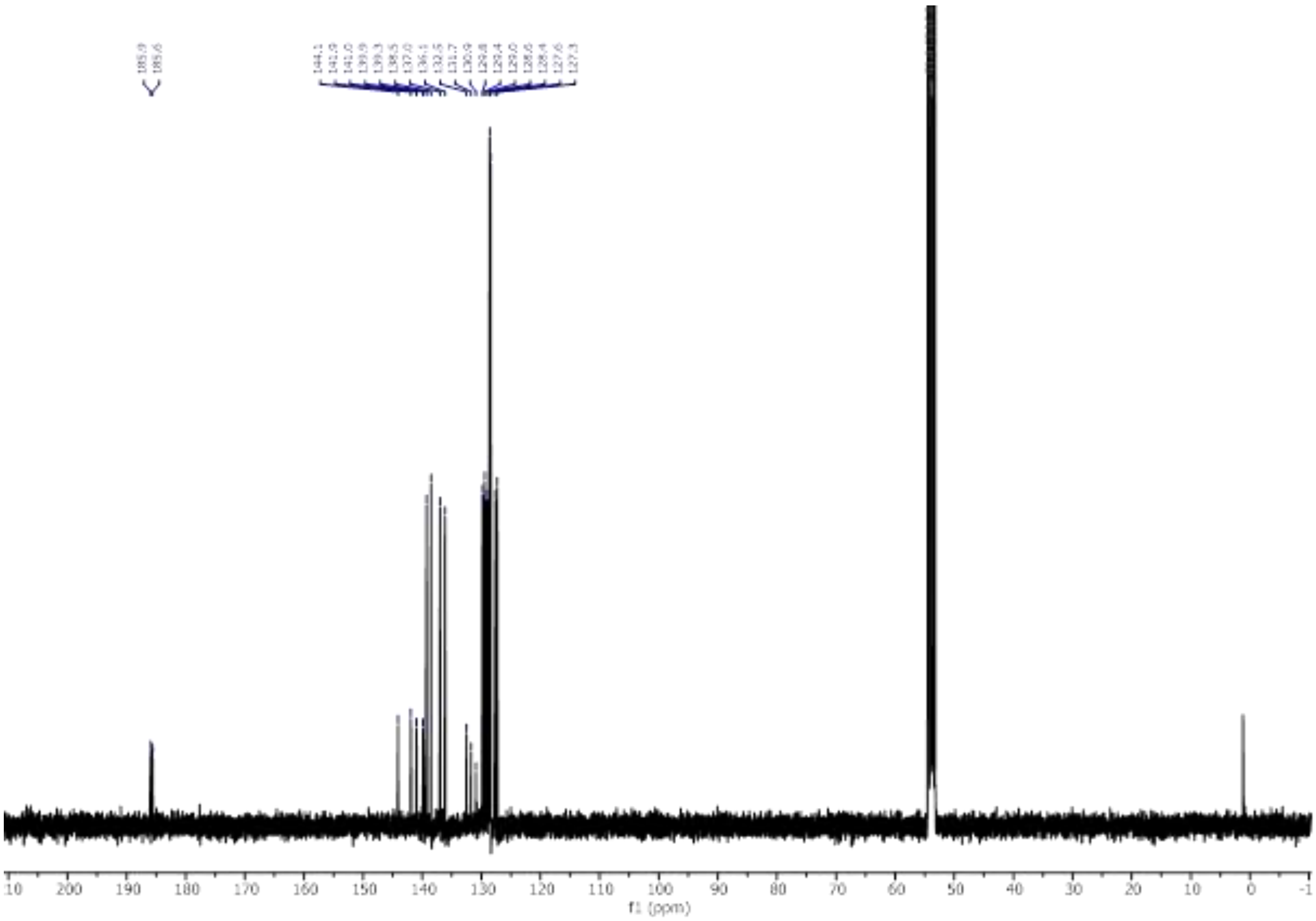

^{13}C NMR spectrum (101 MHz, CD_2Cl_2) of **5n**.

5-(4-Chlorophenyl)-8-phenylnaphthalene-1,4-dione (**5o**)

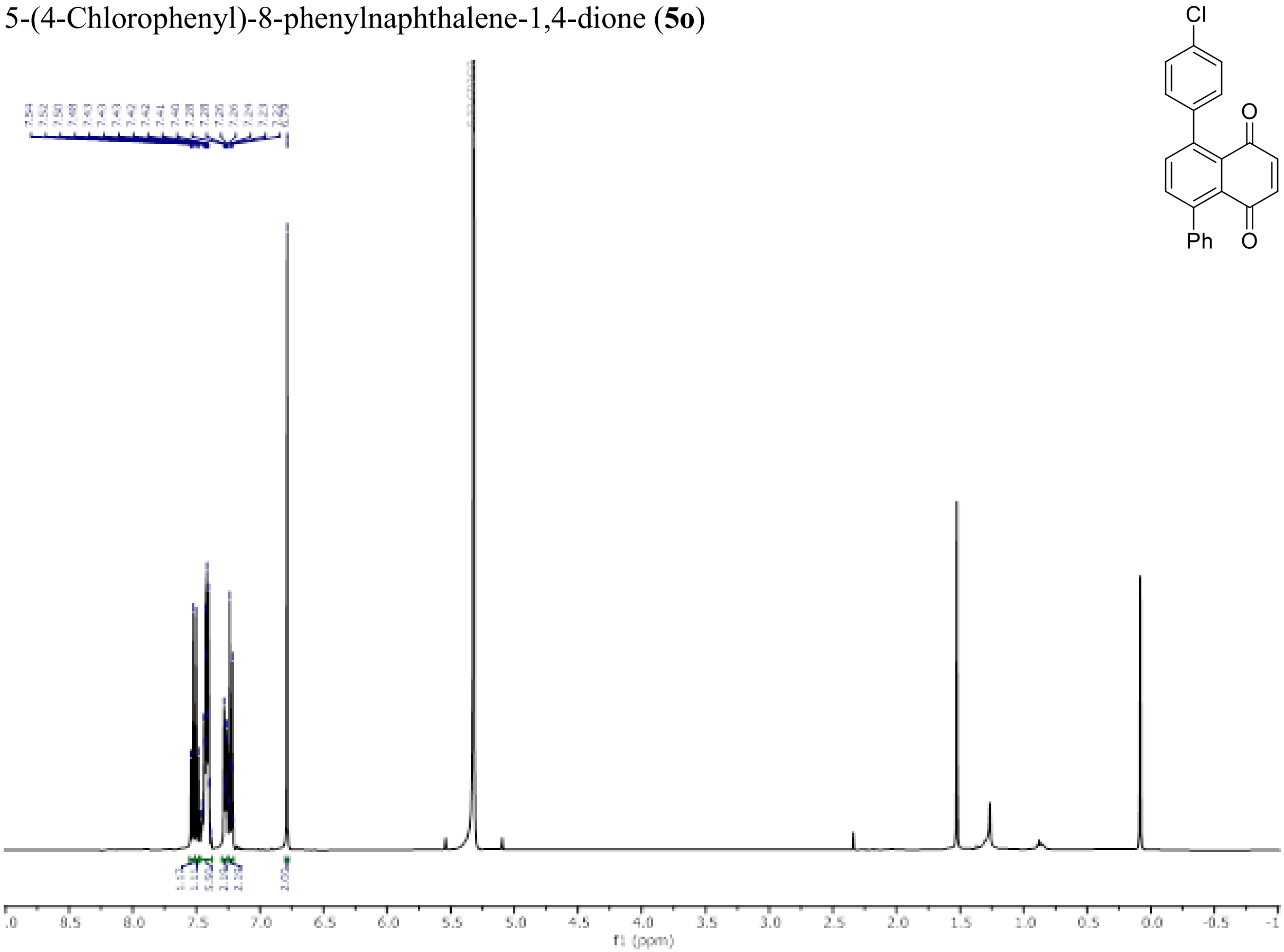

^{1}H NMR spectrum (400 MHz, CD_2Cl_2) of **5o**.

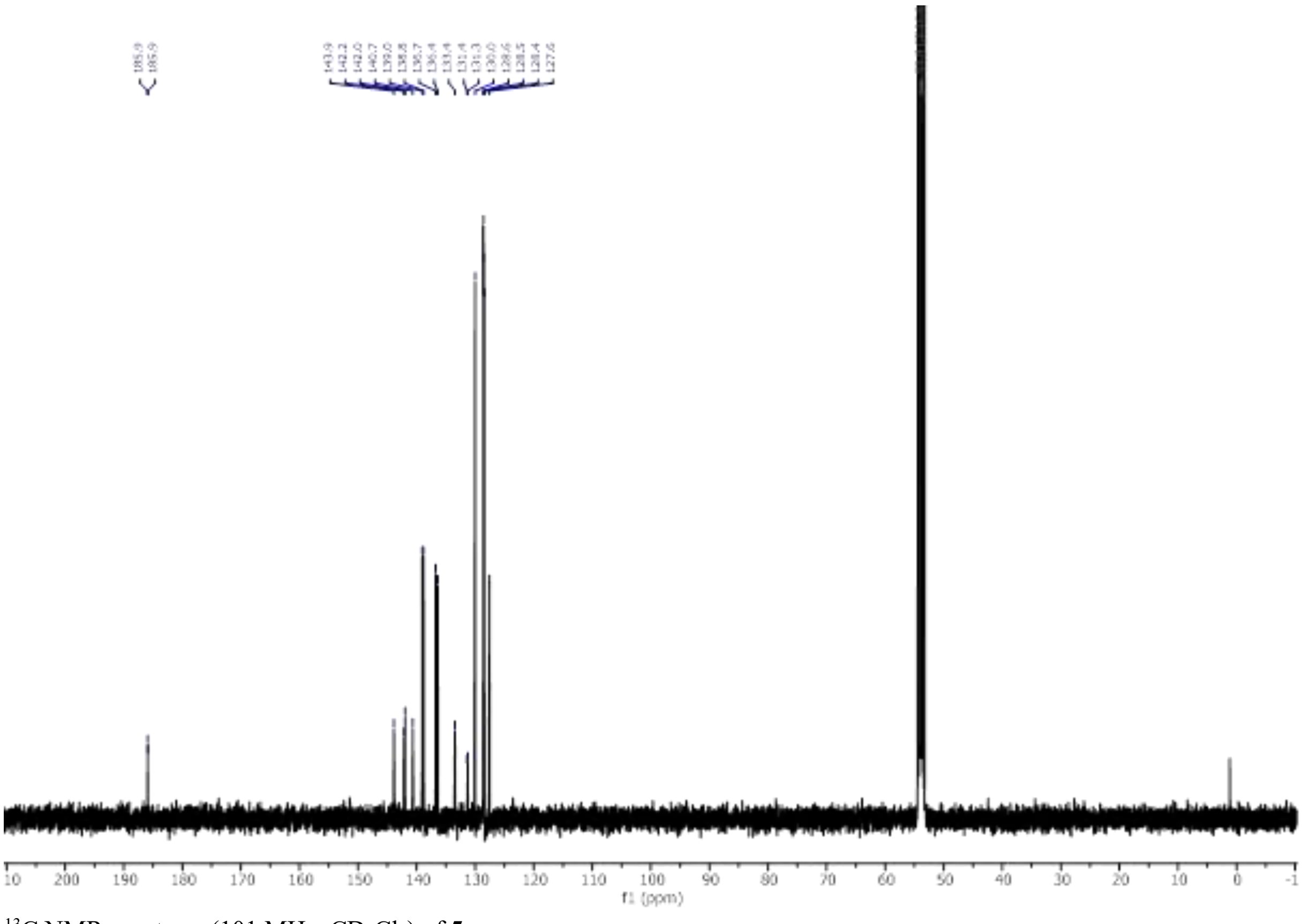

^{13}C NMR spectrum (101 MHz, CD_2Cl_2) of **5o**.

5-(2-Bromophenyl)-8-phenylnaphthalene-1,4-dione (**5p**)

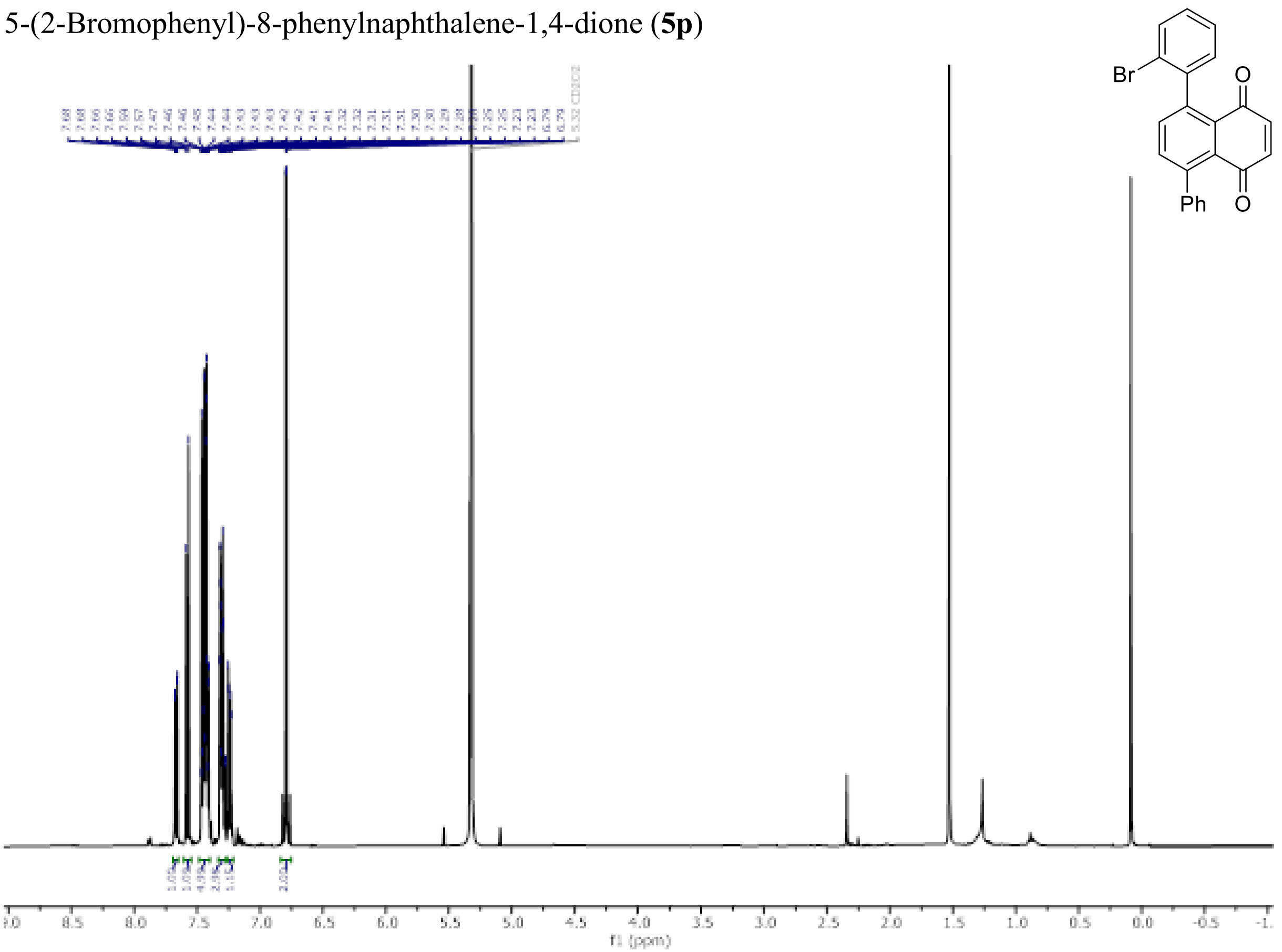

^{1}H NMR spectrum (400 MHz, CD_2Cl_2) of **5p**.

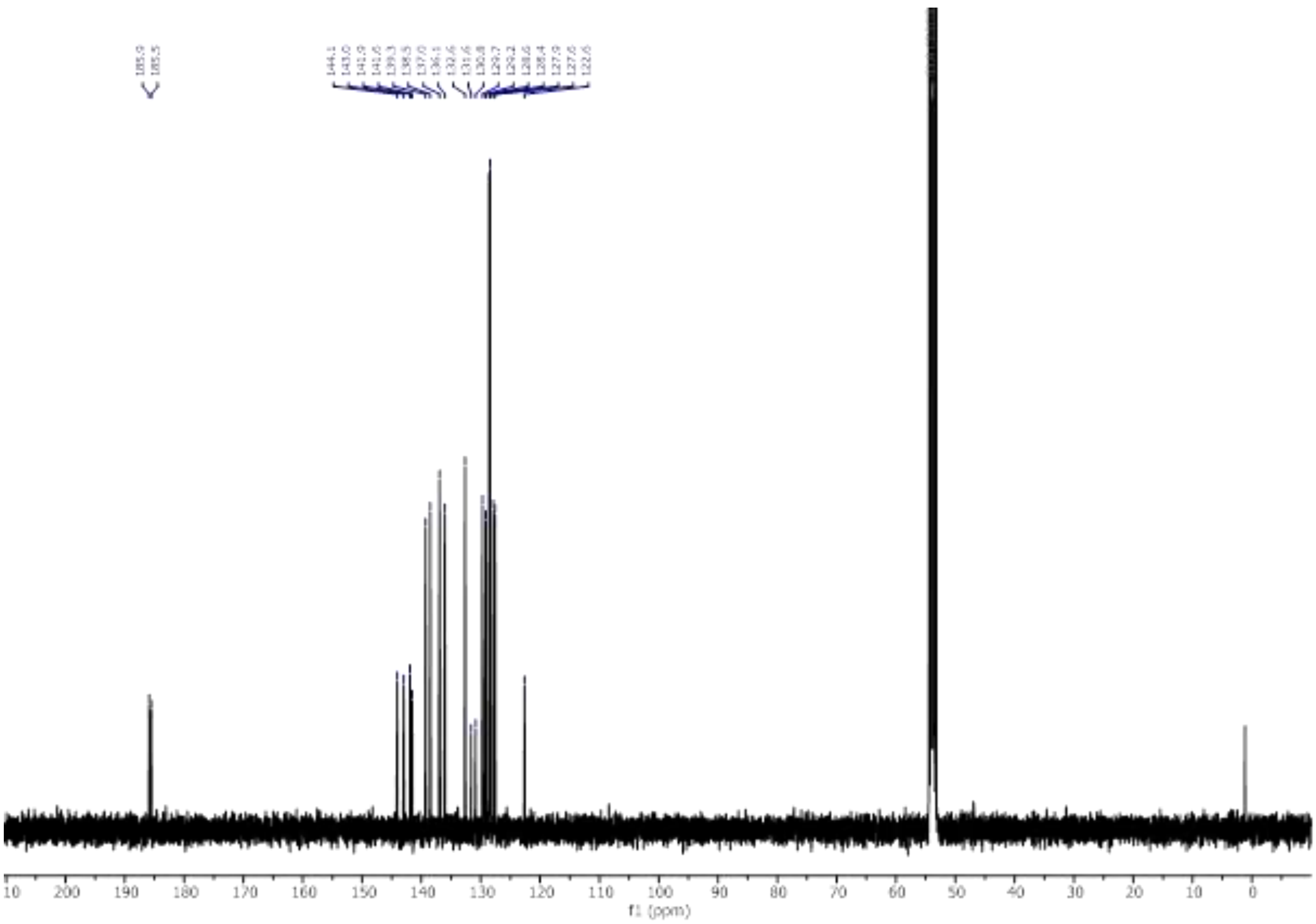

^{13}C NMR spectrum (101 MHz, CD_2Cl_2) of **5p**.

5-(3-Bromophenyl)-8-phenylnaphthalene-1,4-dione (**5q**)

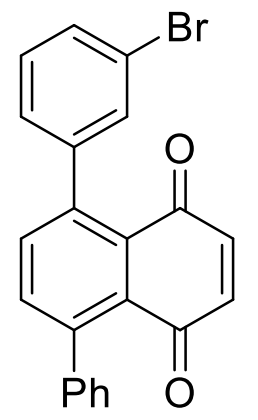

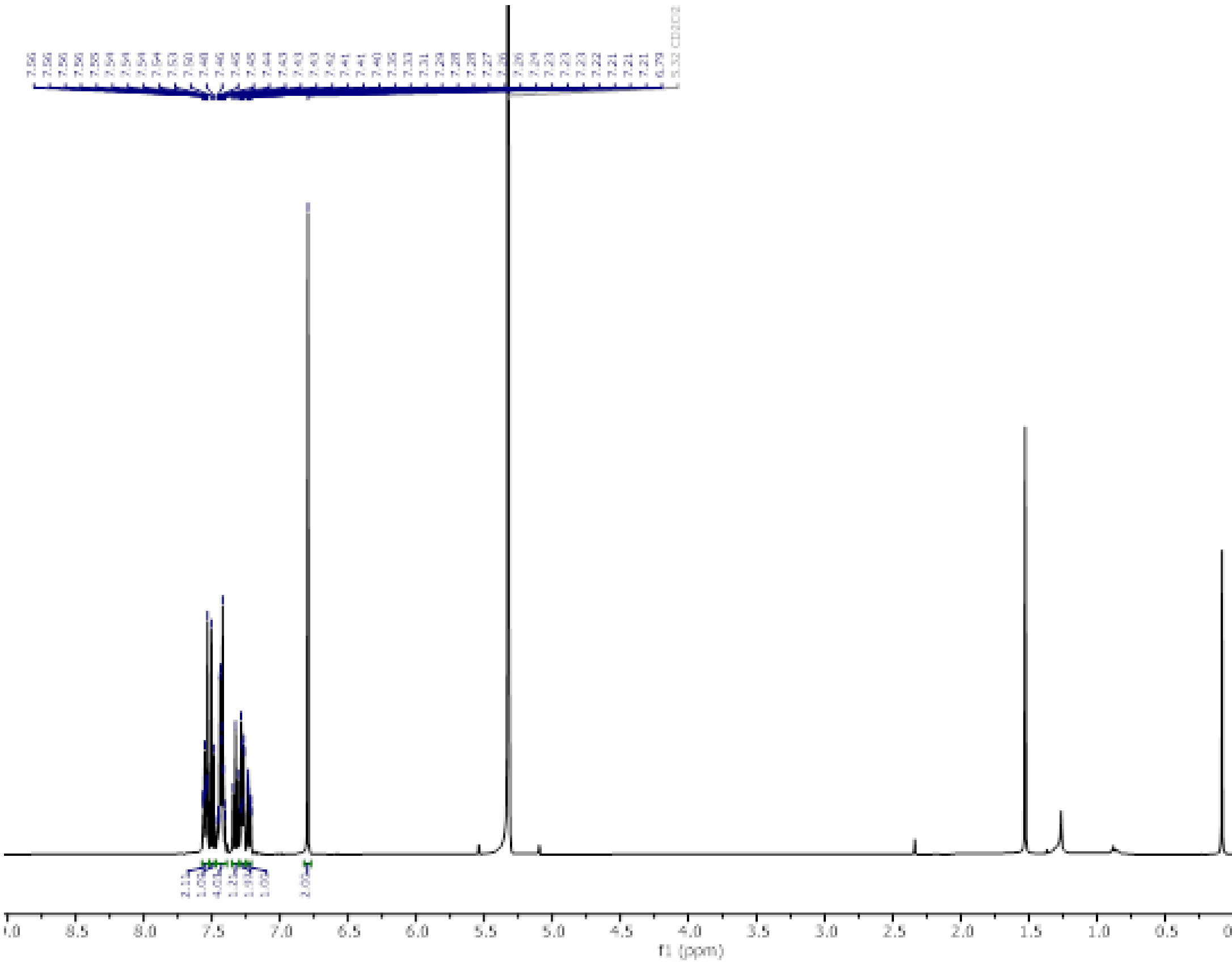

^{1}H NMR spectrum (400 MHz, CD_2Cl_2) of **5q**.

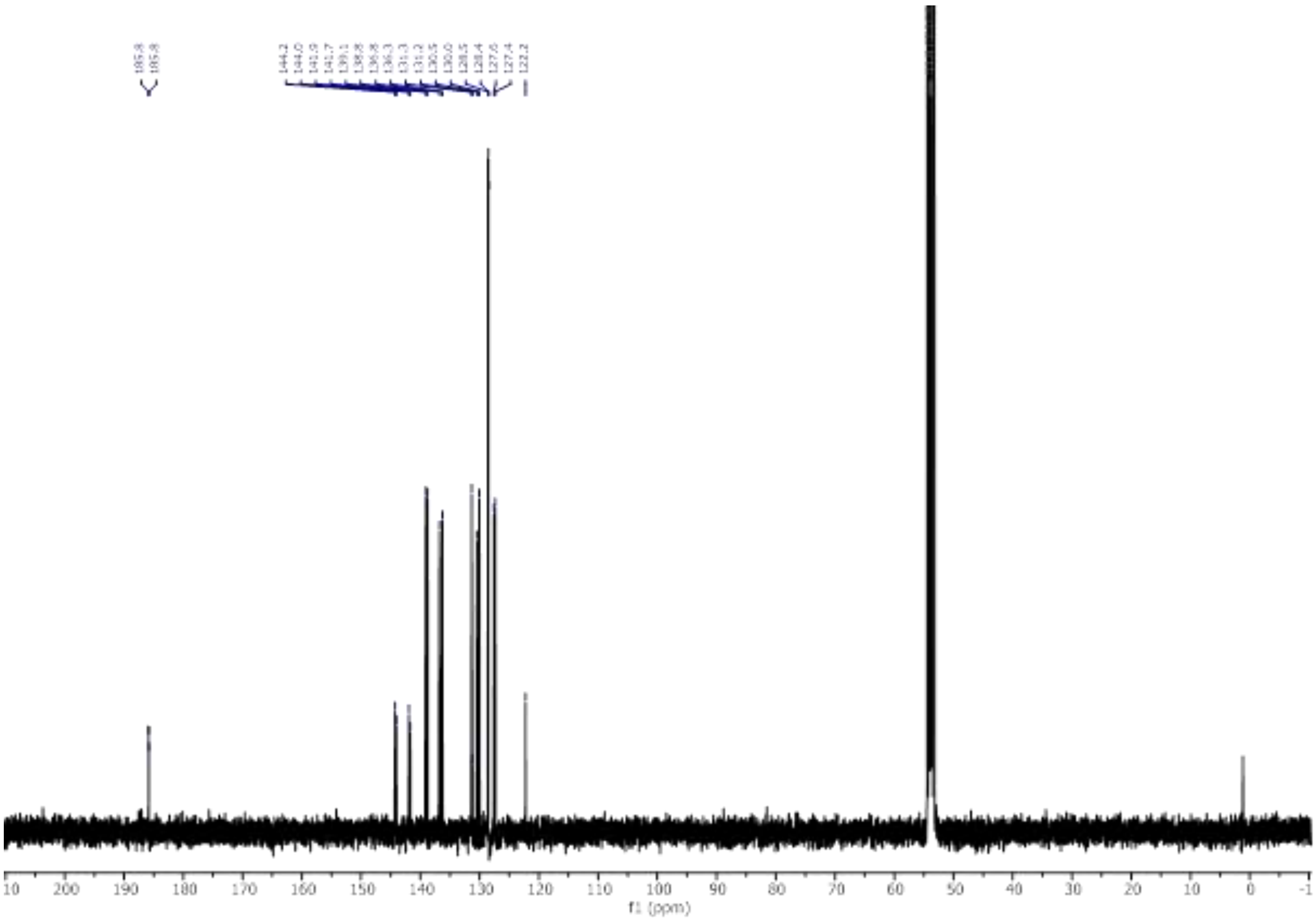

^{13}C NMR spectrum (101 MHz, CD_2Cl_2) of **5q**.

5-(4-Bromophenyl)-8-phenylnaphthalene-1,4-dione (**5r**)

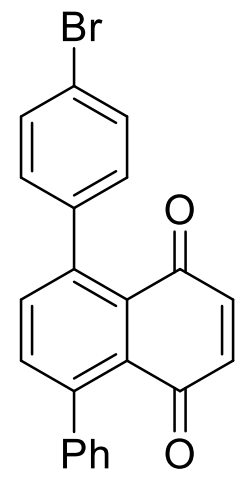

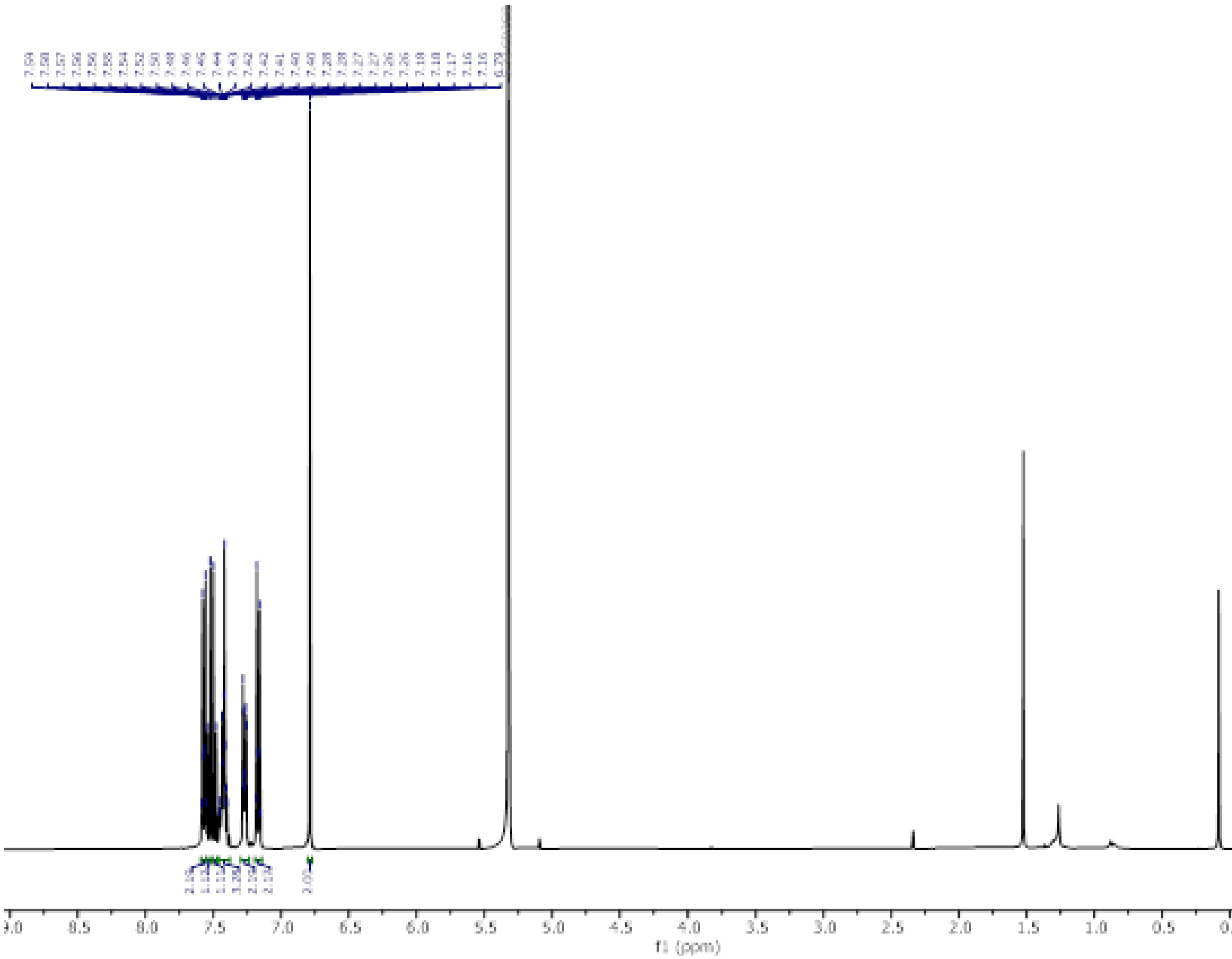

^{1}H NMR spectrum (400 MHz, CD_2Cl_2) of **5r**.

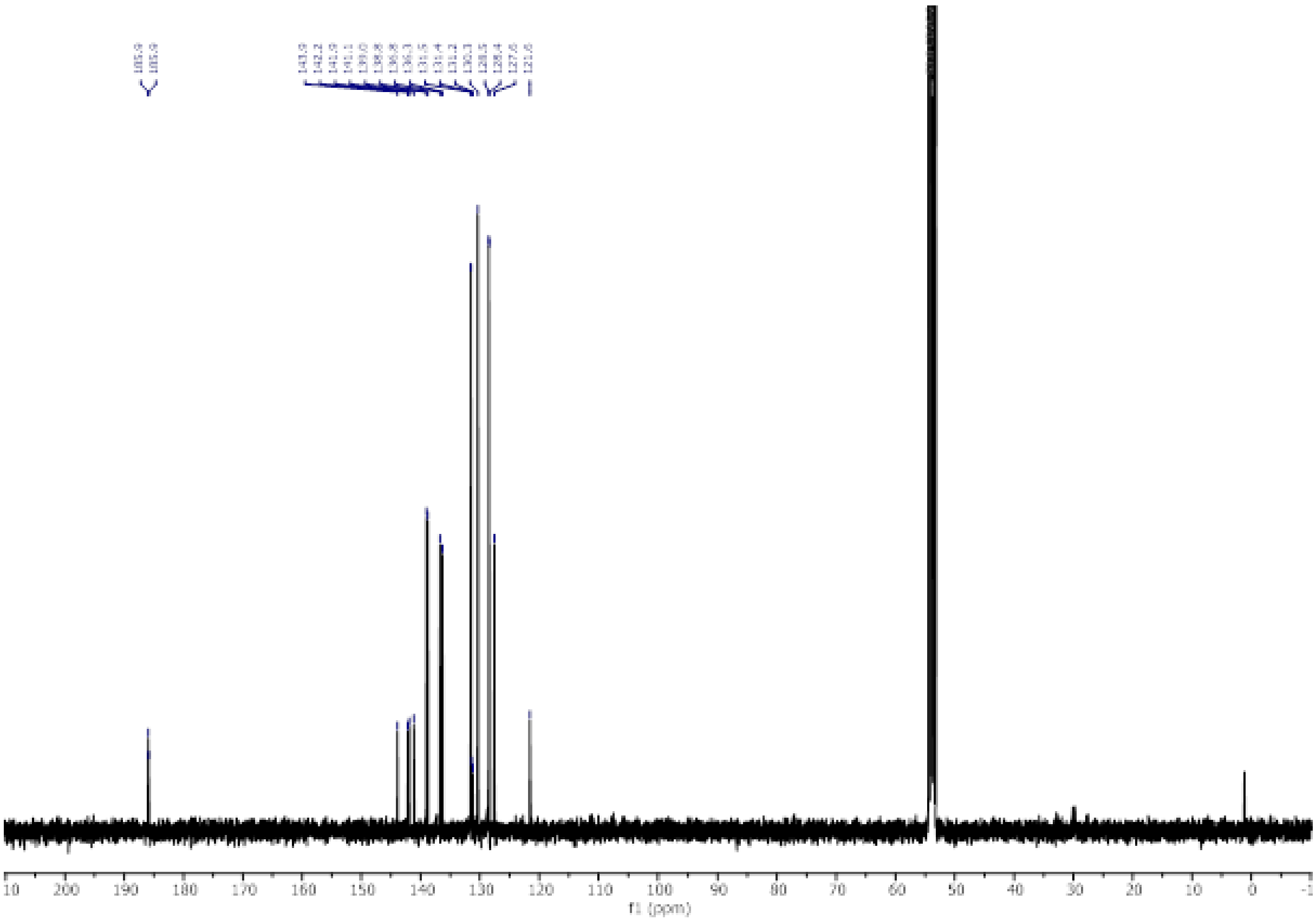

^{13}C NMR spectrum (101 MHz, CD_2Cl_2) of **5r**.

5-(4-Iodophenyl)-8-phenylnaphthalene-1,4-dione (**5s**)

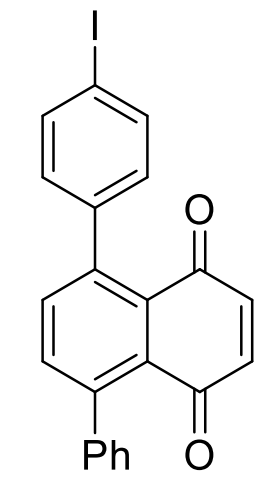

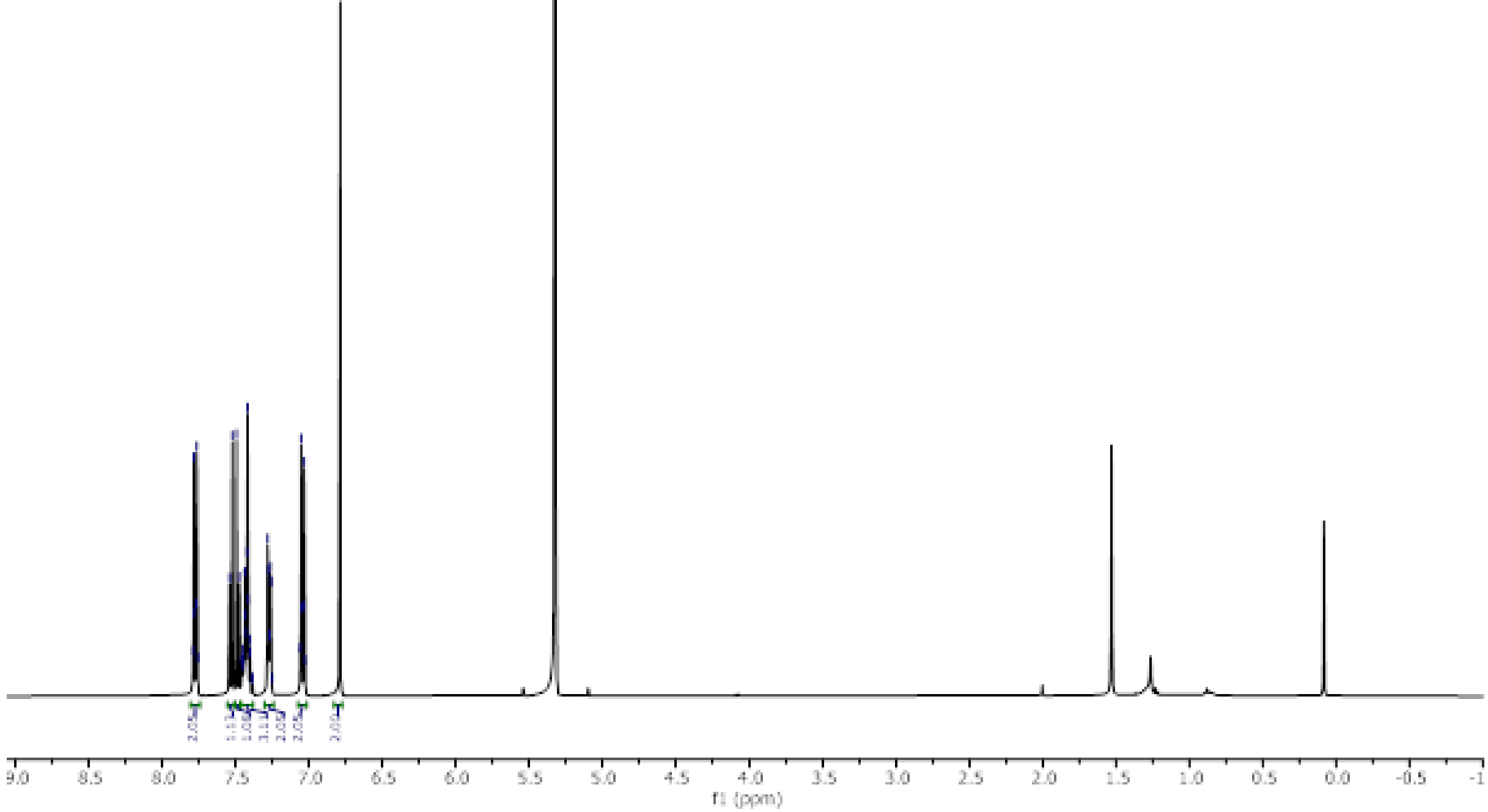

^{1}H NMR spectrum (400 MHz, CD_2Cl_2) of **5s**.

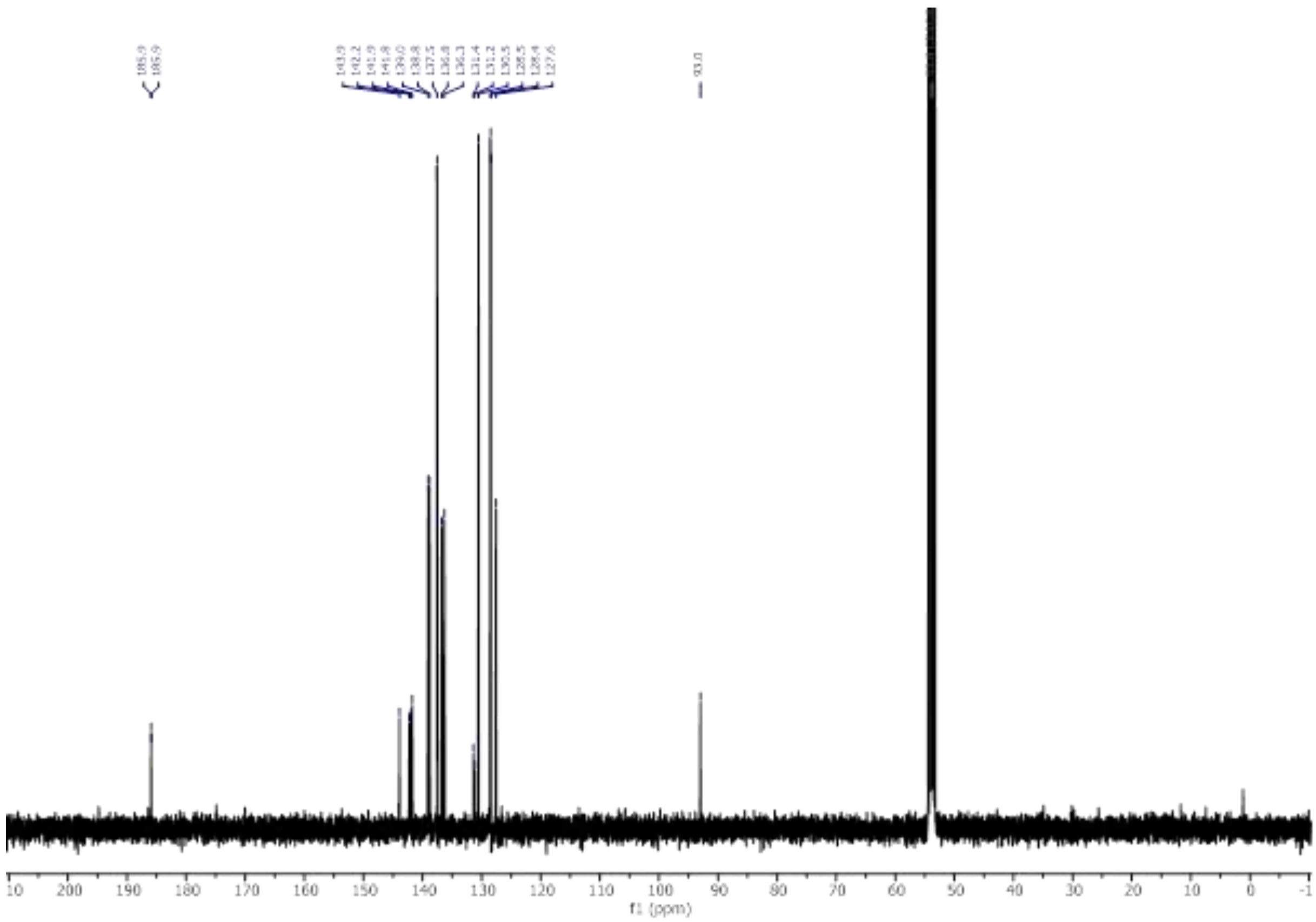

^{13}C NMR spectrum (101 MHz, CD_2Cl_2) of **5s**.

5-(4-Trifluoromethylphenyl)-8-phenylnaphthalene-1,4-dione (**5t**)

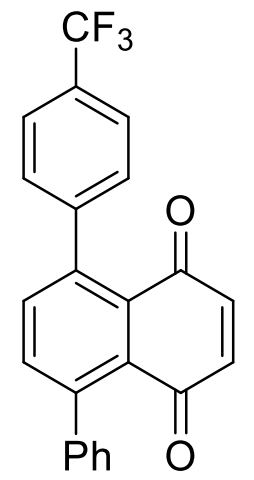

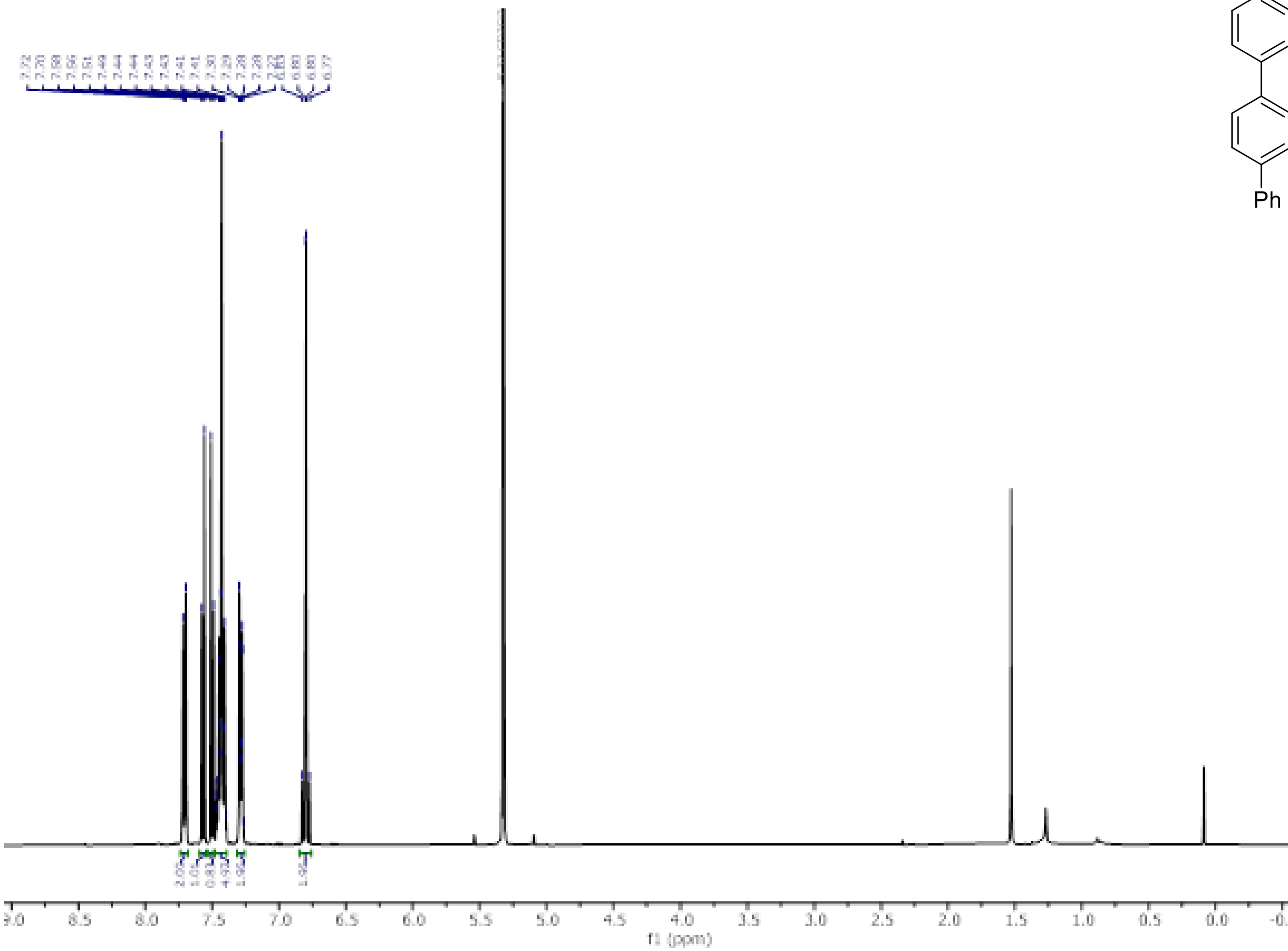

^{1}H NMR spectrum (400 MHz, CD_2Cl_2) of **5t**.

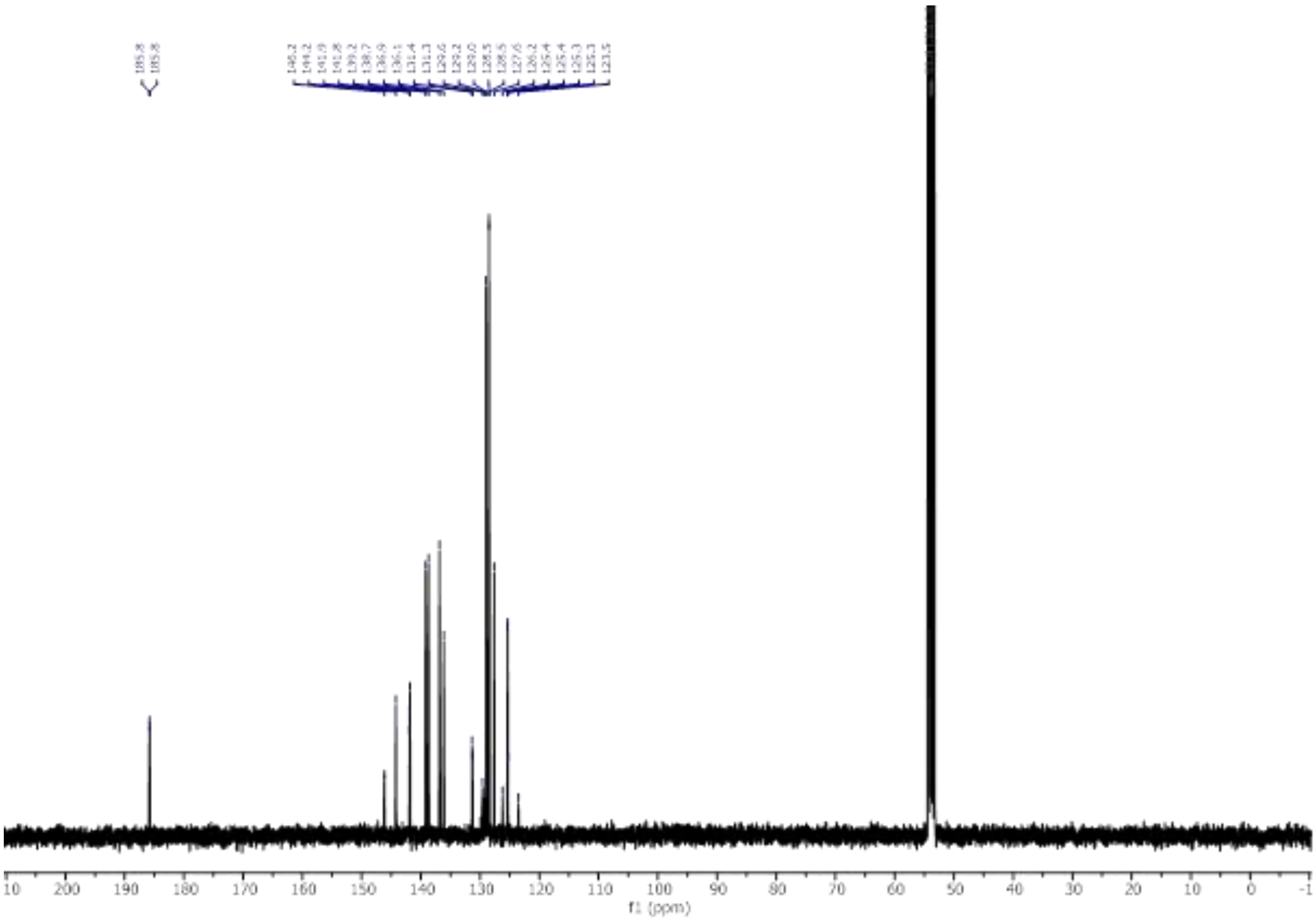

^{13}C NMR spectrum (101 MHz, CD_2Cl_2) of **5t**.

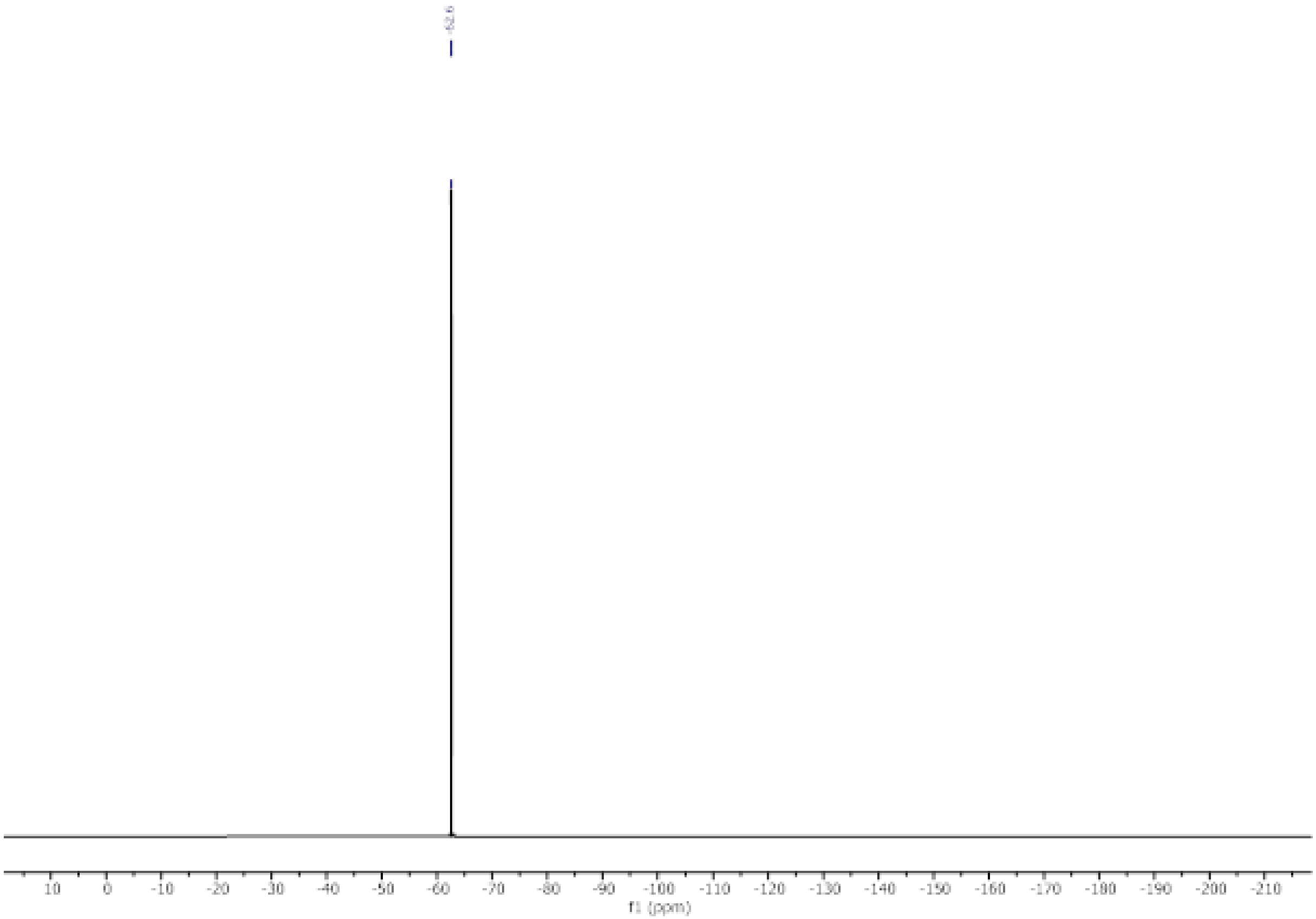

^{19}F NMR spectrum (344 MHz, CD_2Cl_2) of **5t**.

5-(4-Methyl benzoate)-8-phenylnaphthalene-1,4-dione (**5u**)

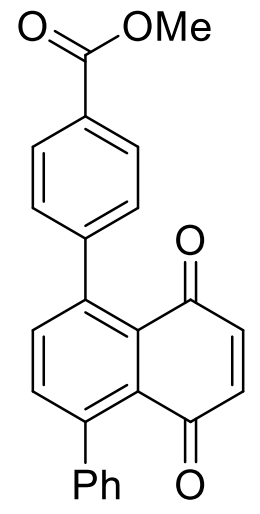

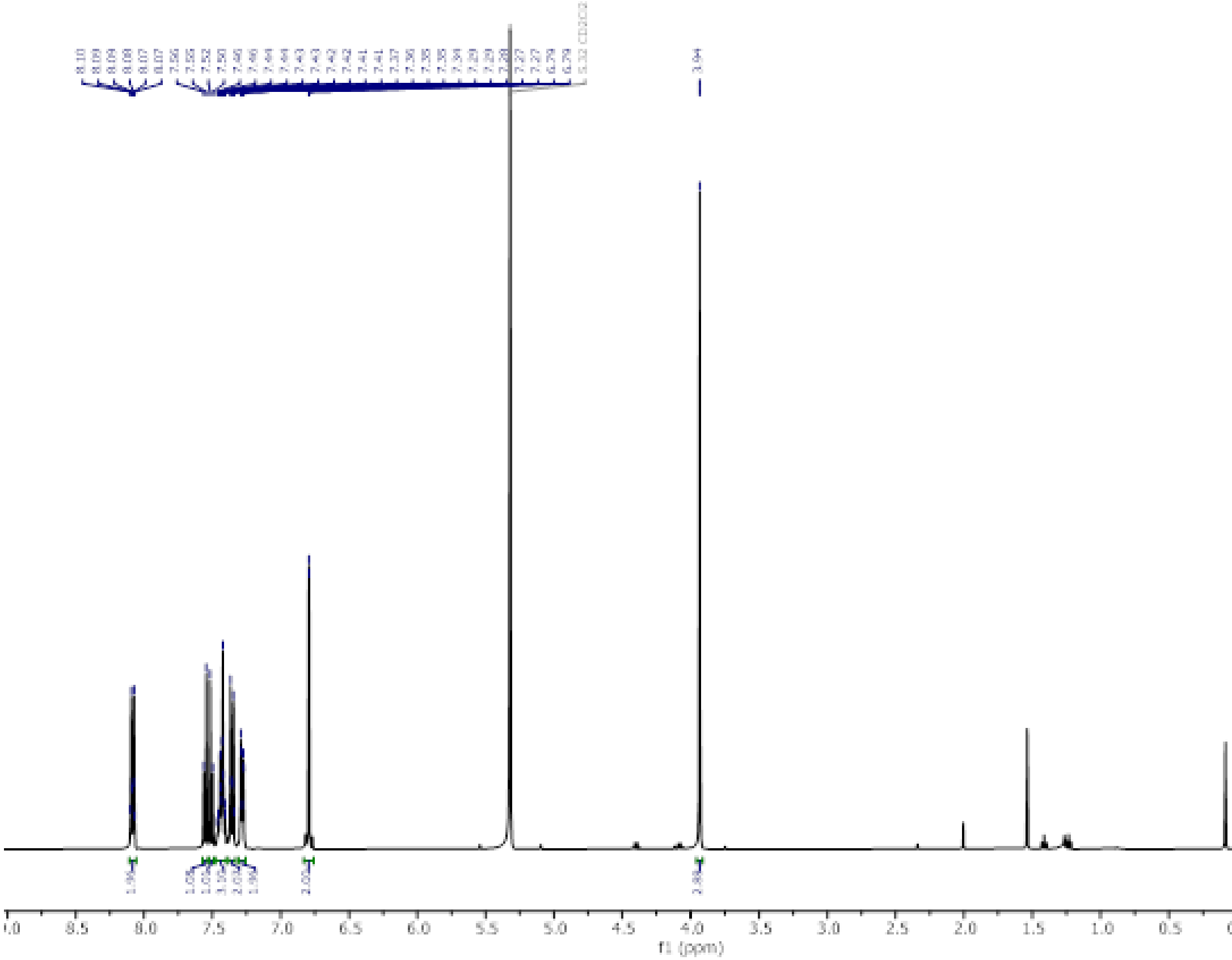

^{1}H NMR spectrum (400 MHz, CD_2Cl_2) of **5u**.

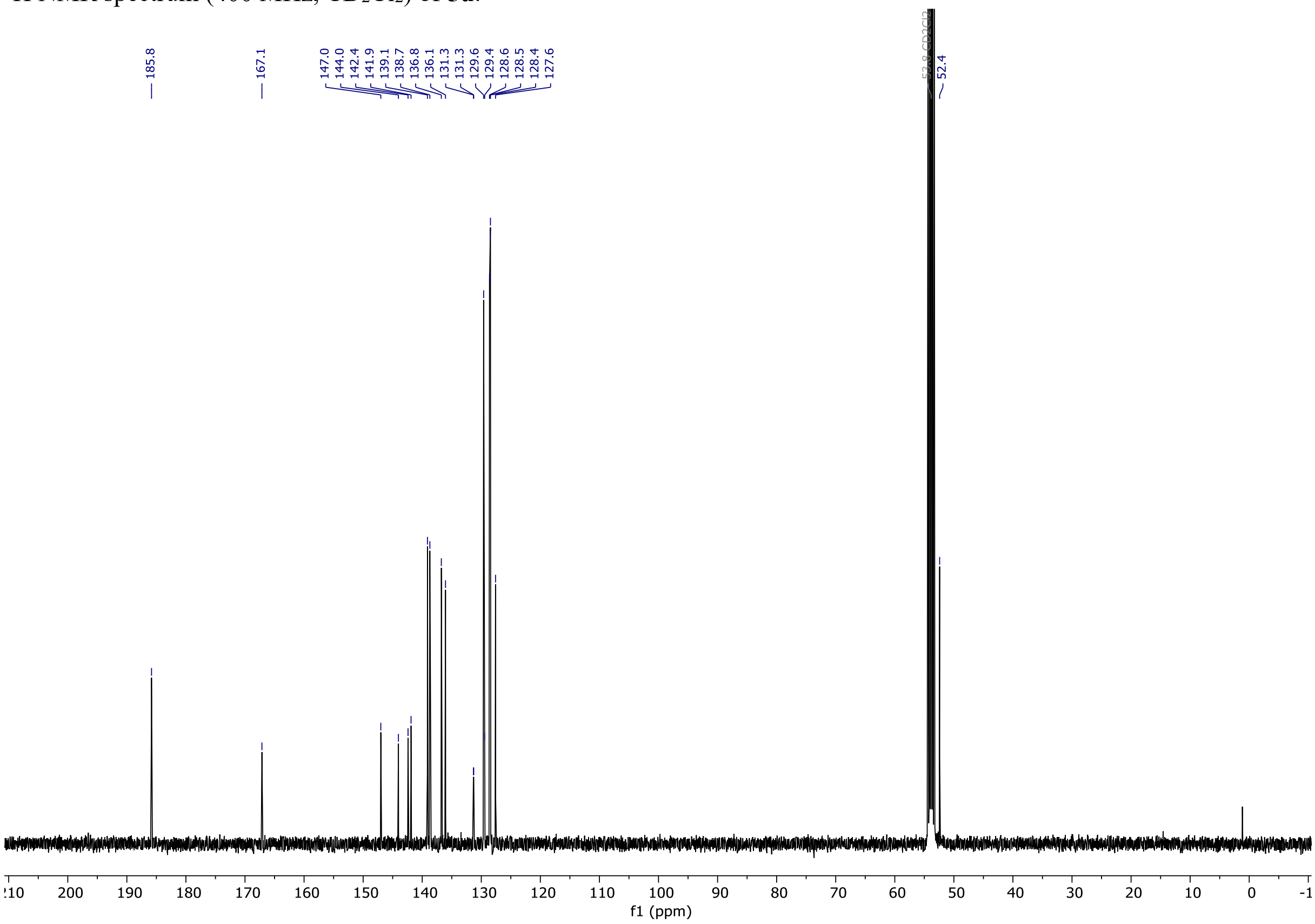

^{13}C NMR spectrum (101 MHz, CD_2Cl_2) of **5u**.

5-(4-Nitrophenyl)-8-phenylnaphthalene-1,4-dione (**5w**)

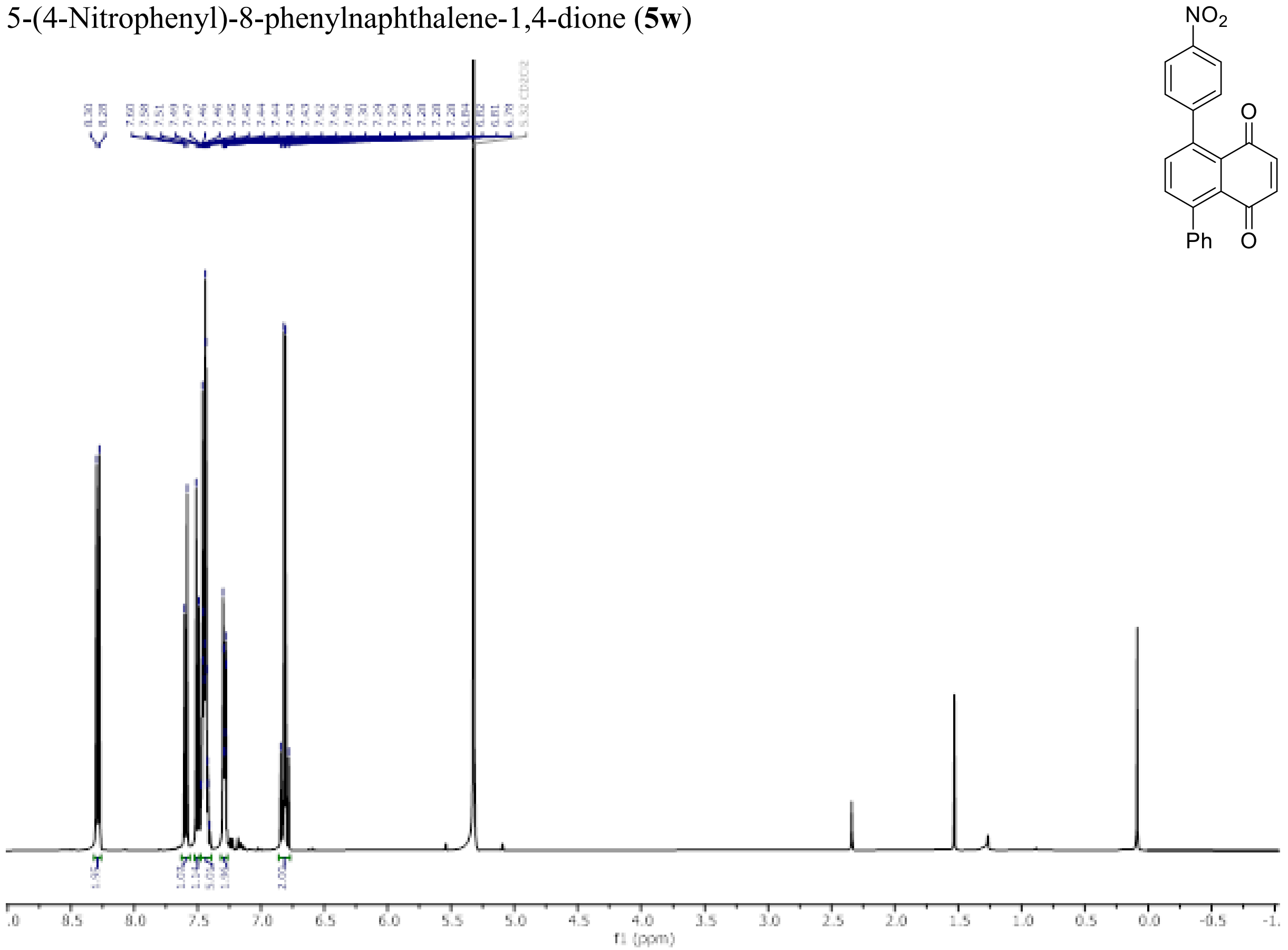

^{1}H NMR spectrum (400 MHz, CD_2Cl_2) of **5w**.

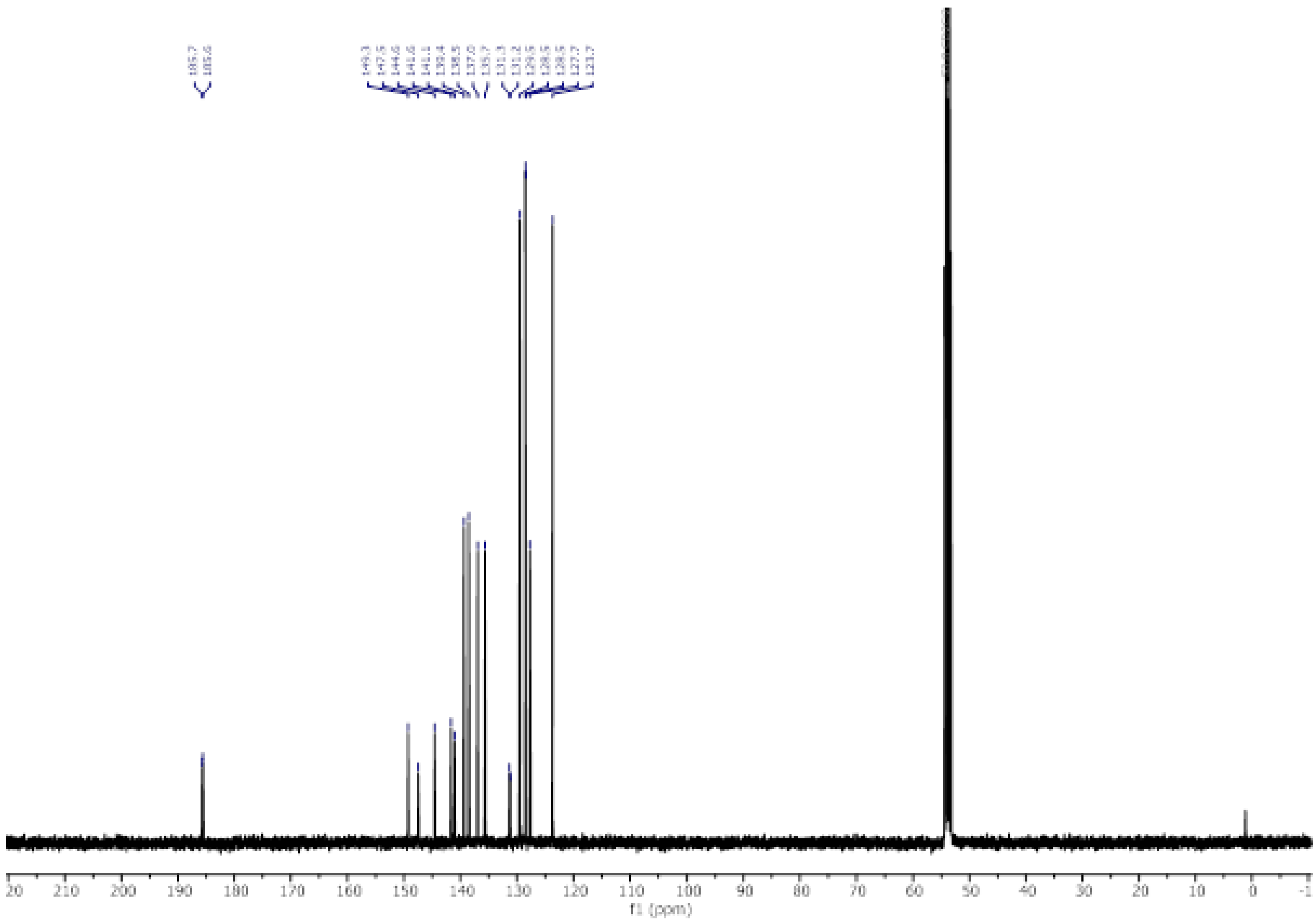

^{13}C NMR spectrum (101 MHz, CD_2Cl_2) of **5w**.

5-Cyclohexyl-8-phenylnaphthalene-1,4-dione (**5y**)

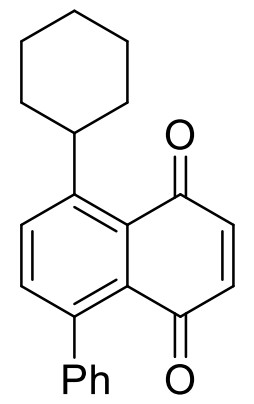

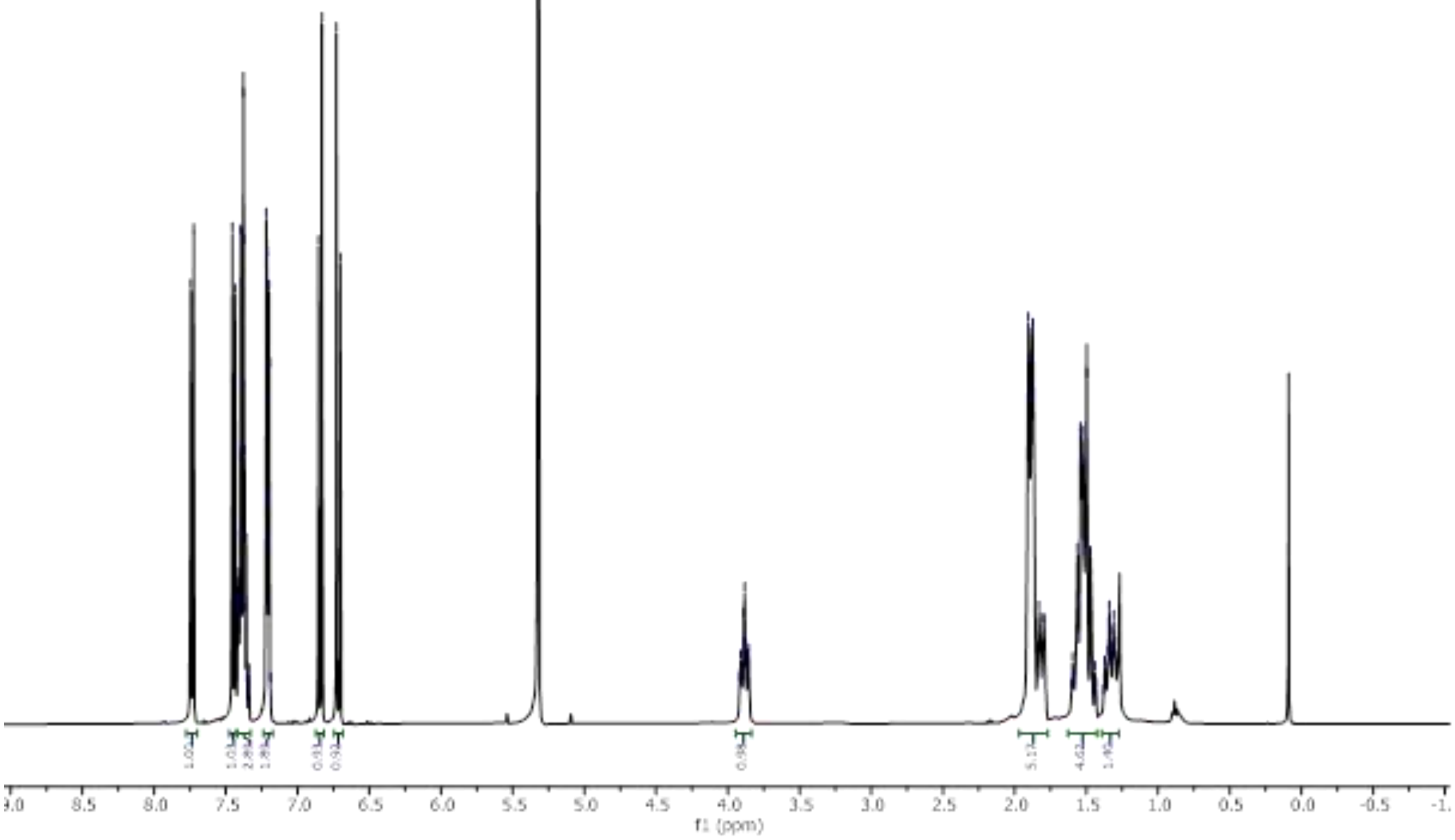

^{1}H NMR spectrum (400 MHz, CD_2Cl_2) of **5y**.

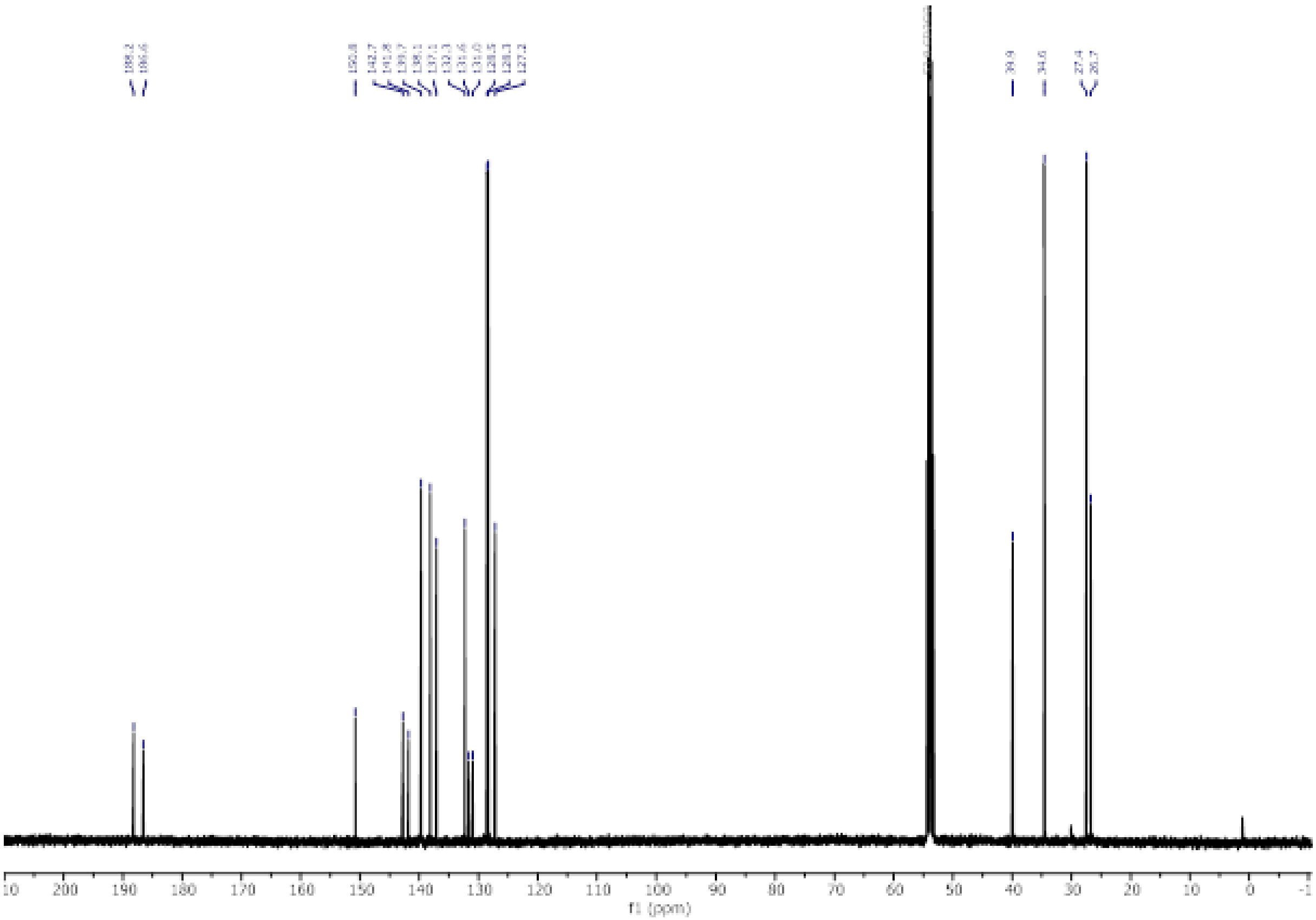

^{13}C NMR spectrum (101 MHz, CD_2Cl_2) of **1y**.

5,8-Di(4-methylphenyl)naphthalene-1,4-dione (**5z**)

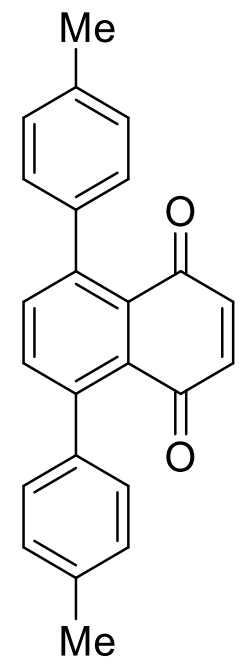

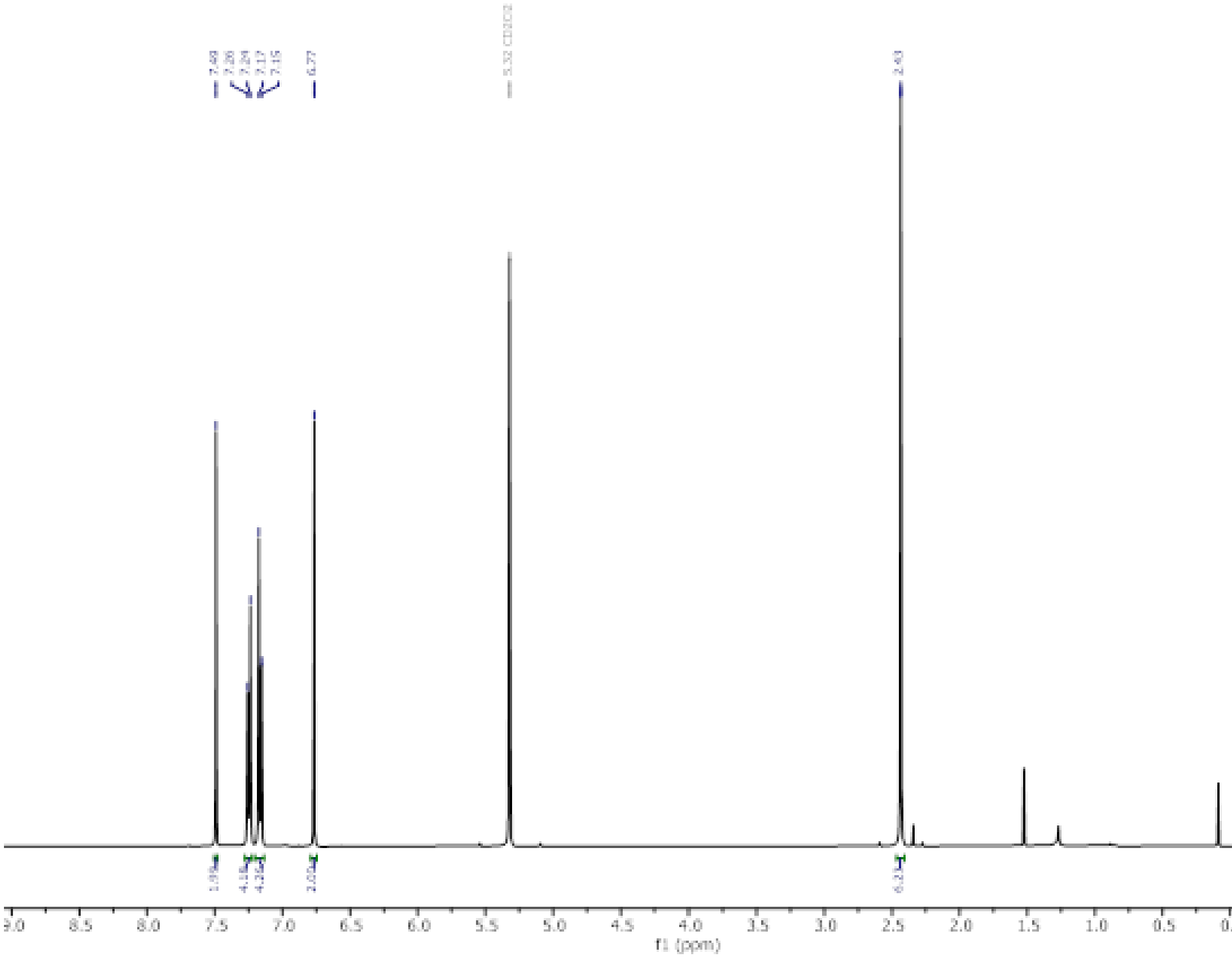

^{1}H NMR spectrum (400 MHz, CD_2Cl_2) of **5z**.

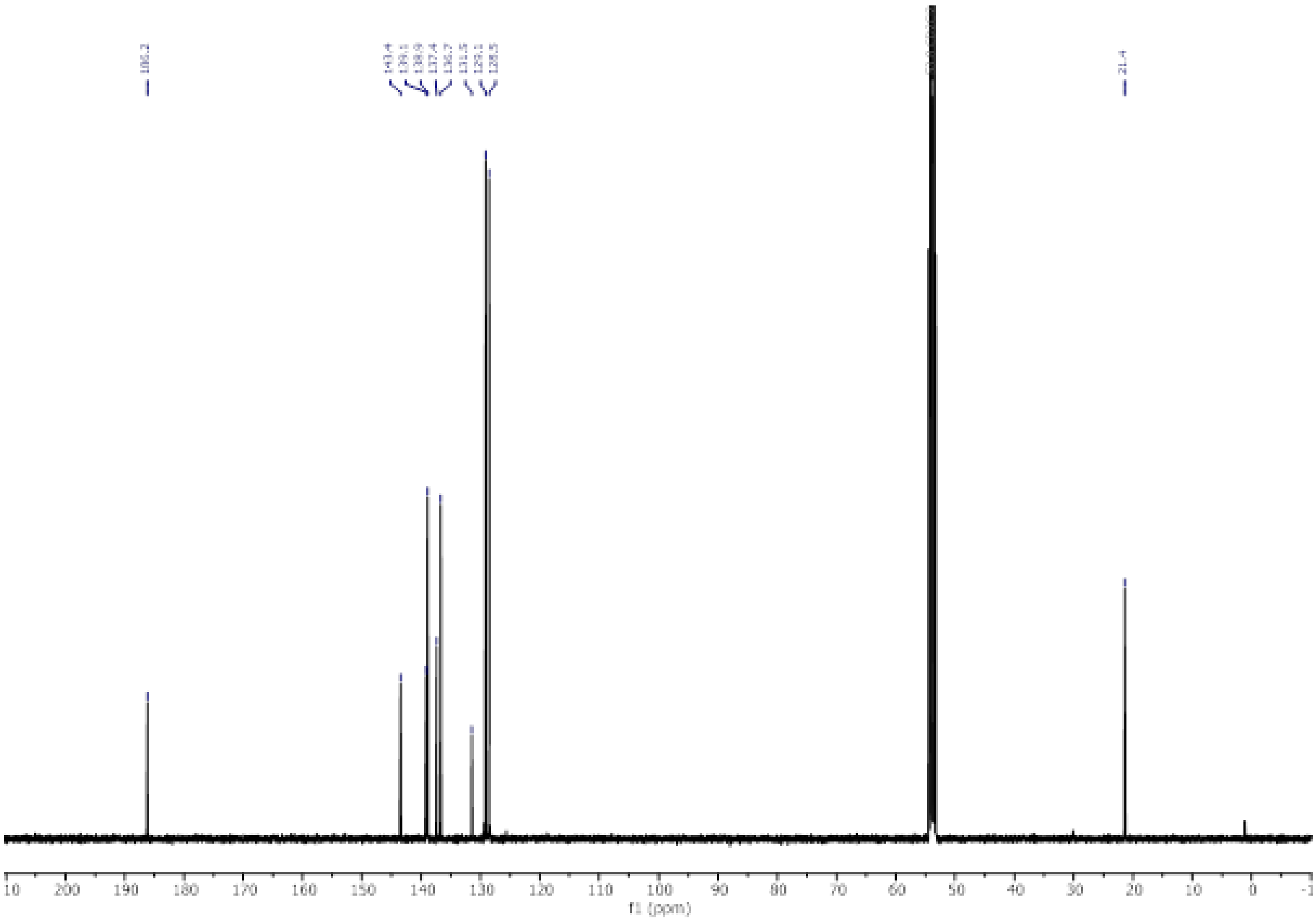

^{13}C NMR spectrum (101 MHz, CD_2Cl_2) of **5z**.

5,8-Di(4-bromophenyl)naphthalene-1,4-dione (**5aa**)

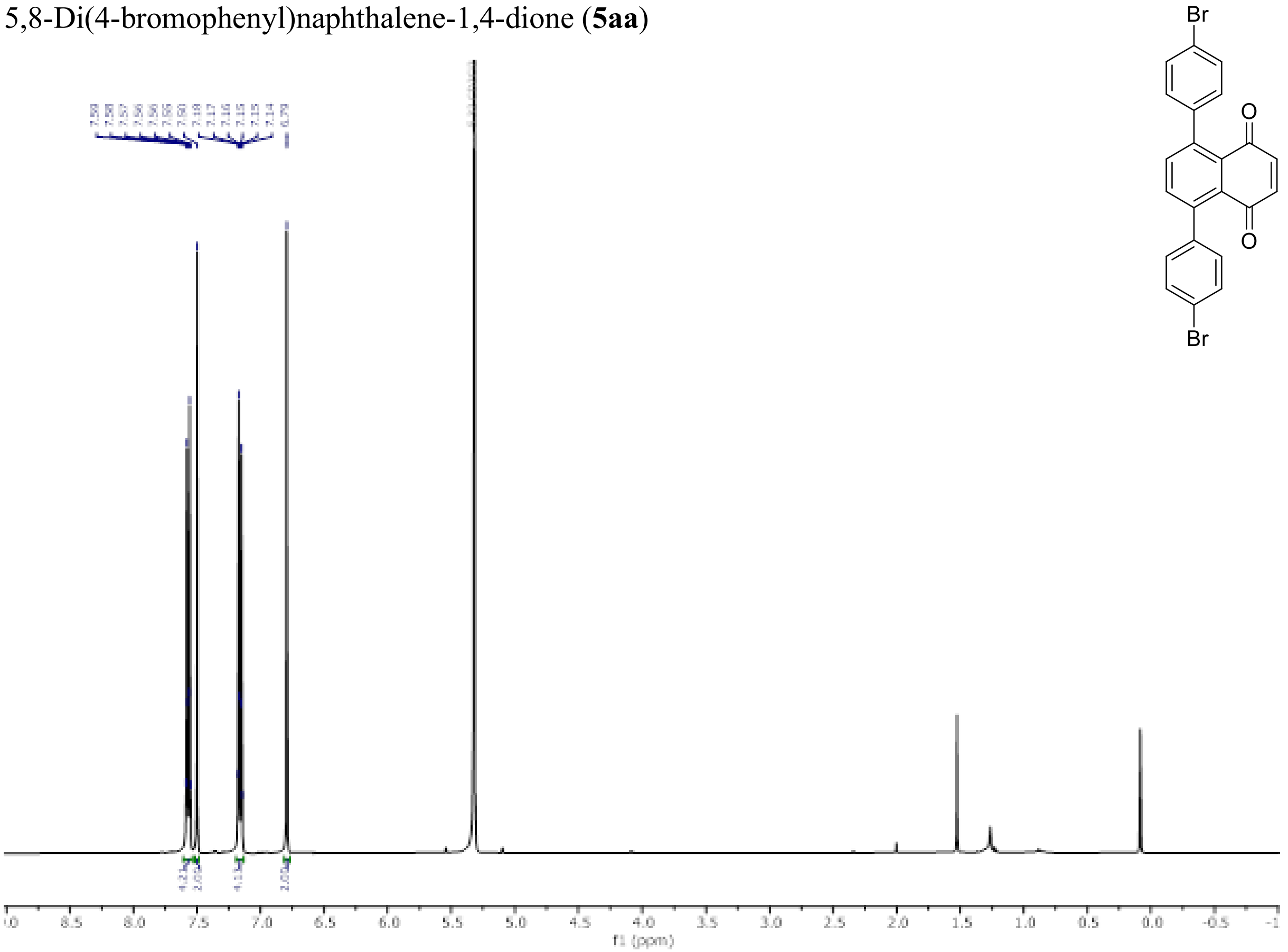

^{1}H NMR spectrum (400 MHz, CD_2Cl_2) of **5aa**.

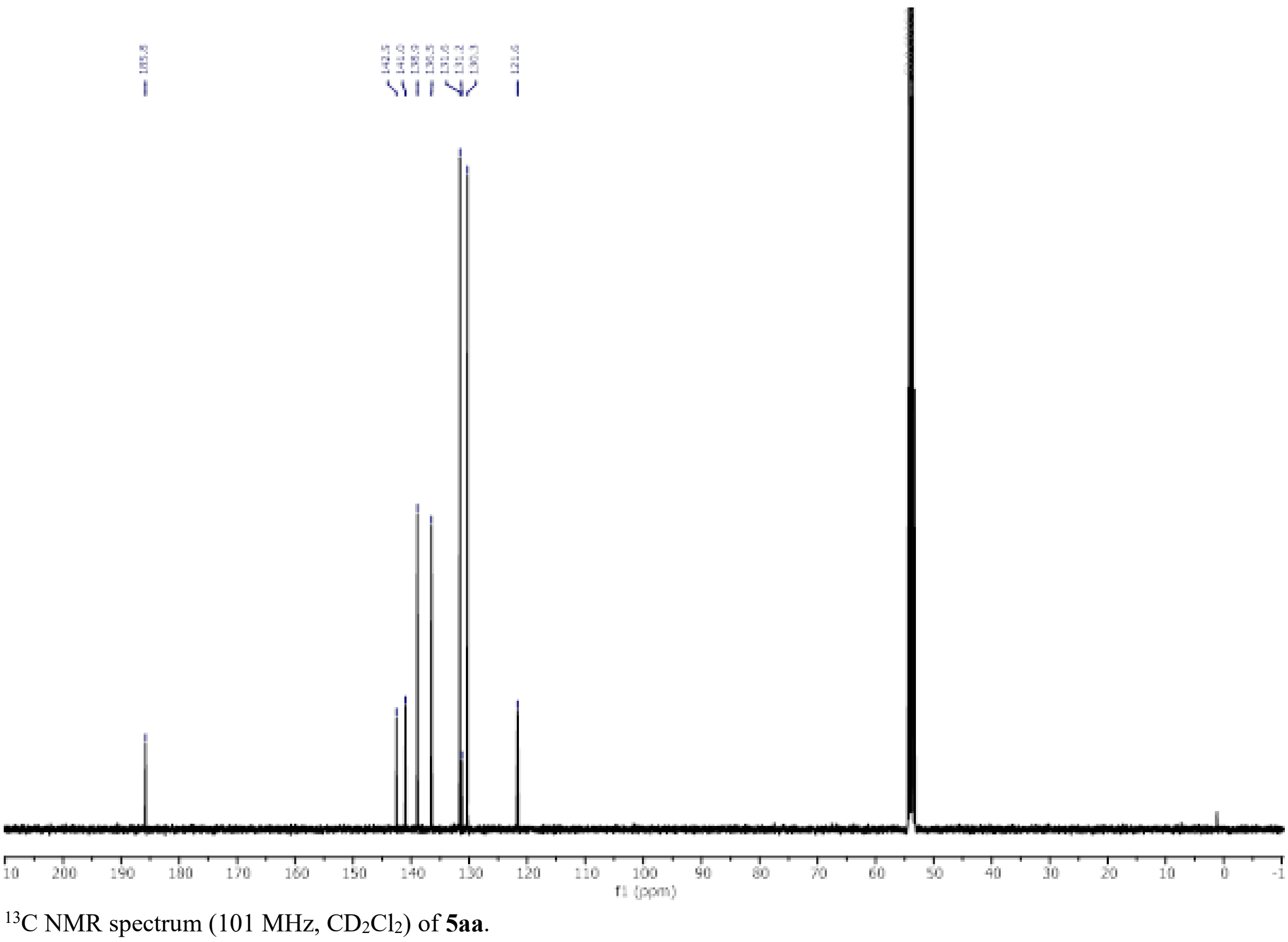

^{13}C NMR spectrum (101 MHz, CD_2Cl_2) of **5aa**.

5-Ethoxycarbonyl-8-phenylnaphthalene-1,4-dione (**5ad**)

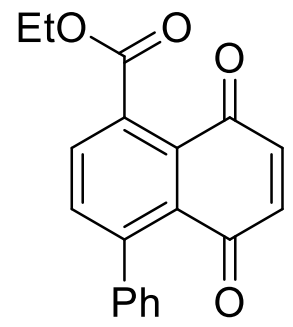

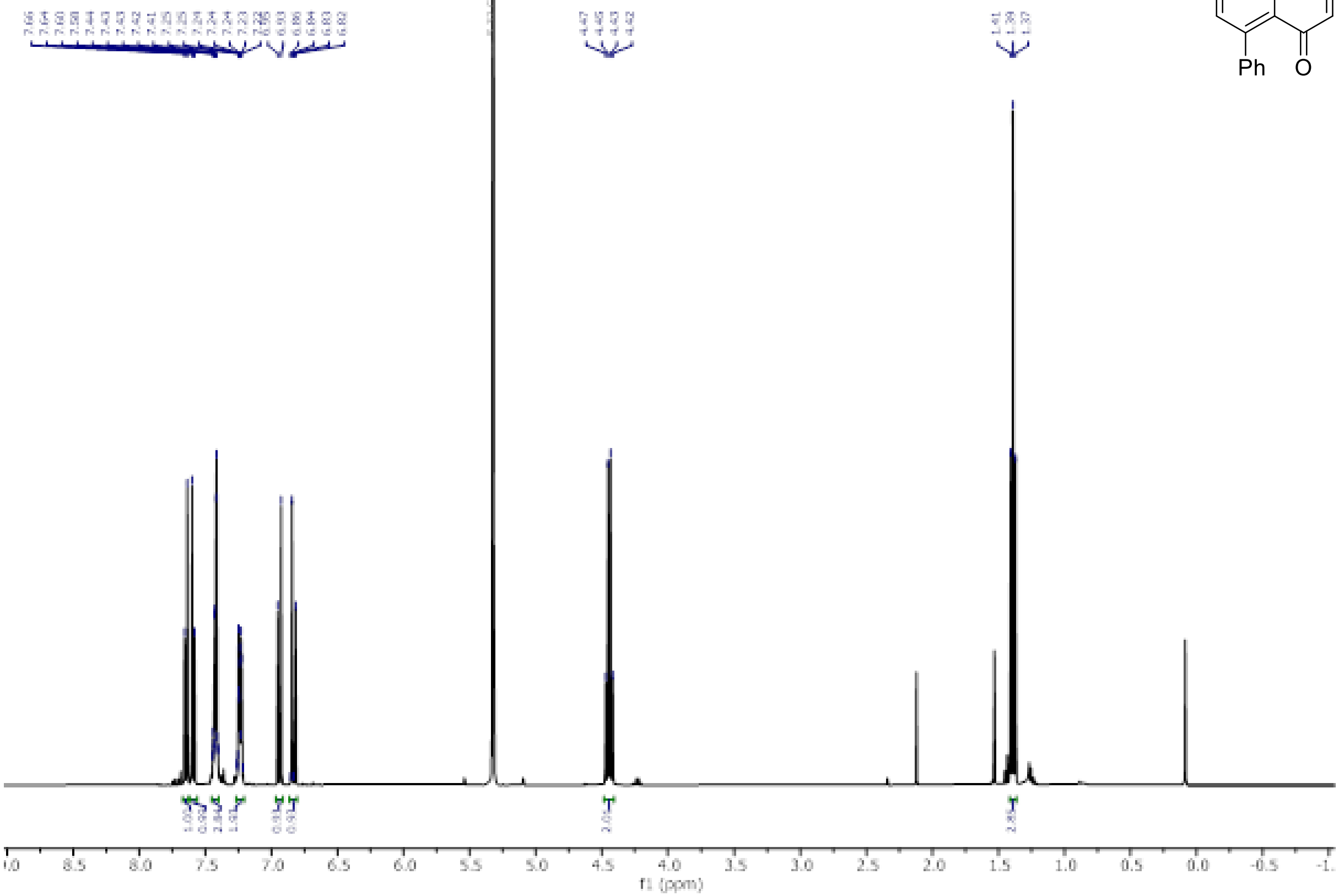

^{1}H NMR spectrum (400 MHz, CD_2Cl_2) of **5ad**.

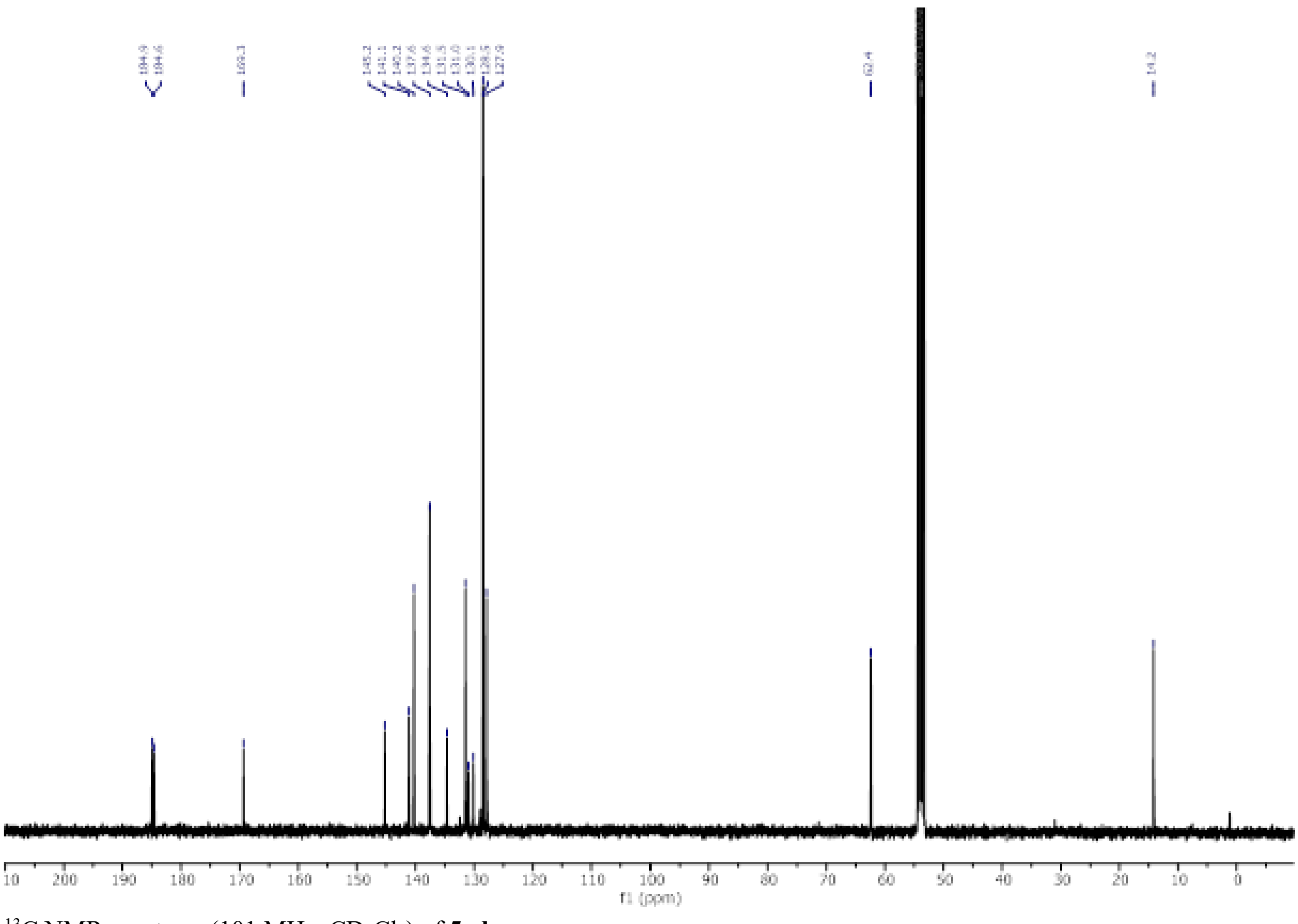

^{13}C NMR spectrum (101 MHz, CD_2Cl_2) of **5ad**.

5-Biphenylnaphthalene-1,4-dione (**5ae**)

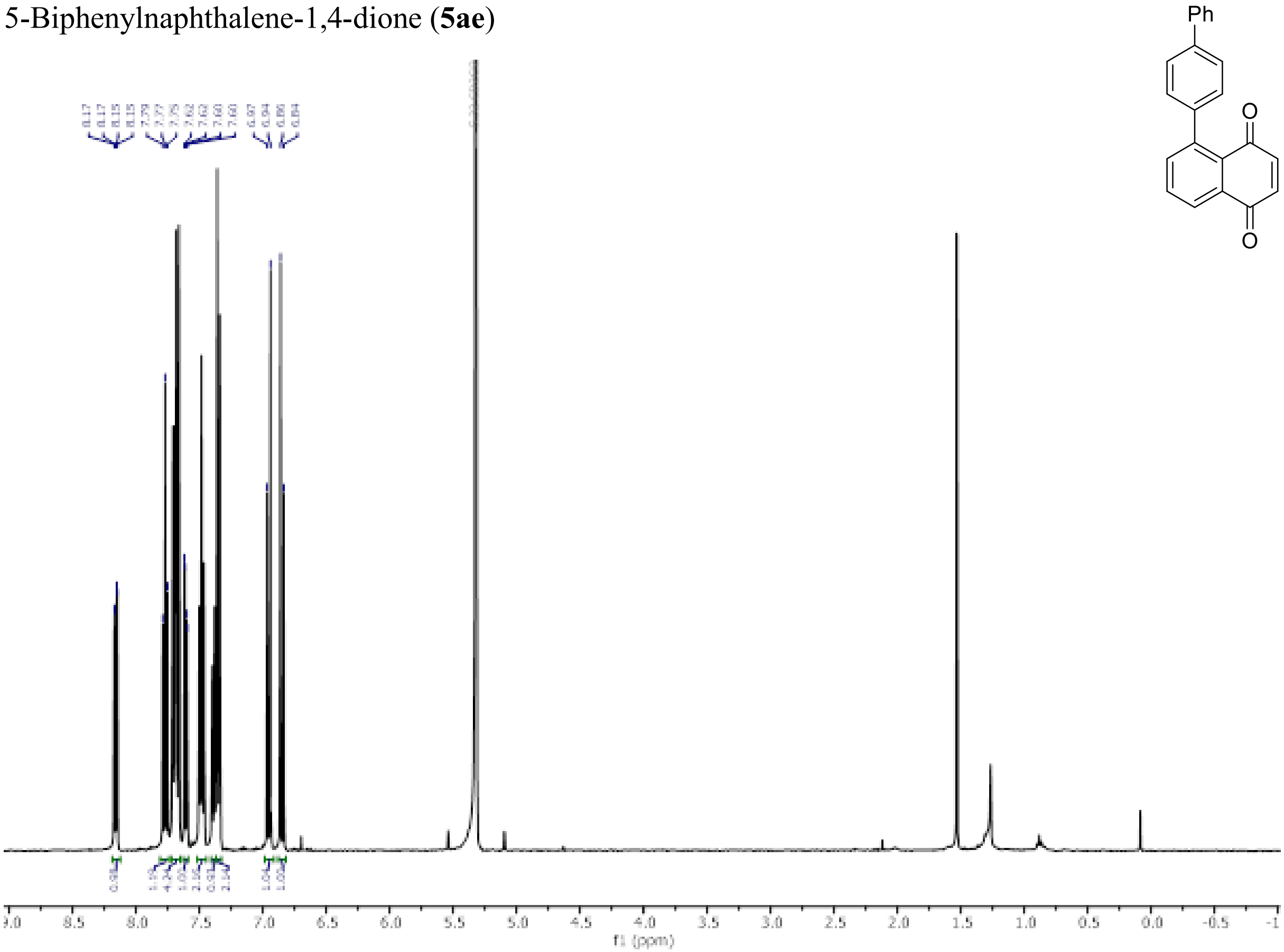

^{1}H NMR spectrum (400 MHz, CD_2Cl_2) of **5ae**.

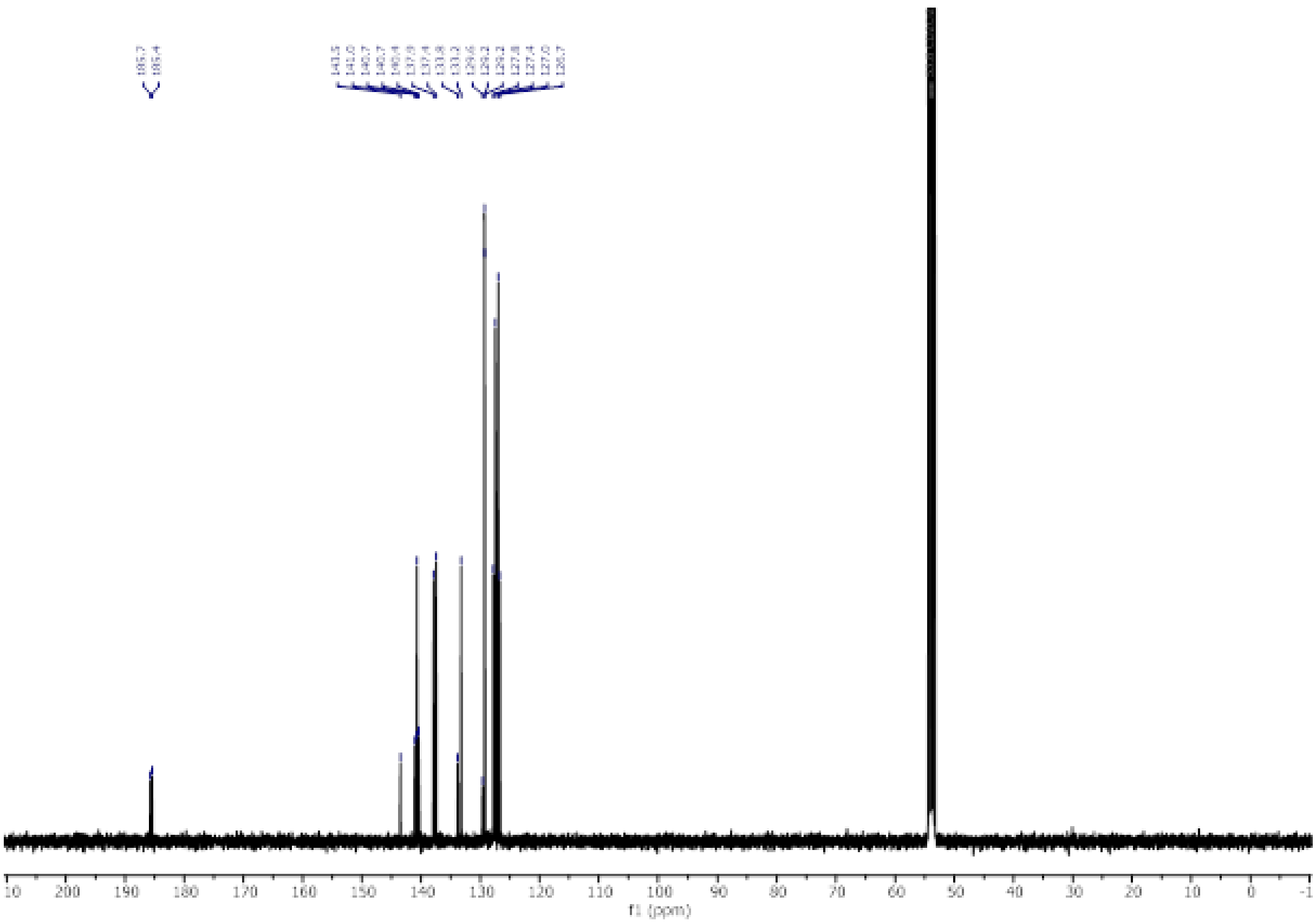

^{13}C NMR spectrum (101 MHz, CD_2Cl_2) of **5ae**.

5-(4-Methylphenyl)naphthalene-1,4-dione (**5af**)

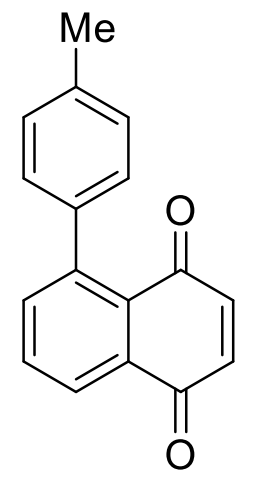

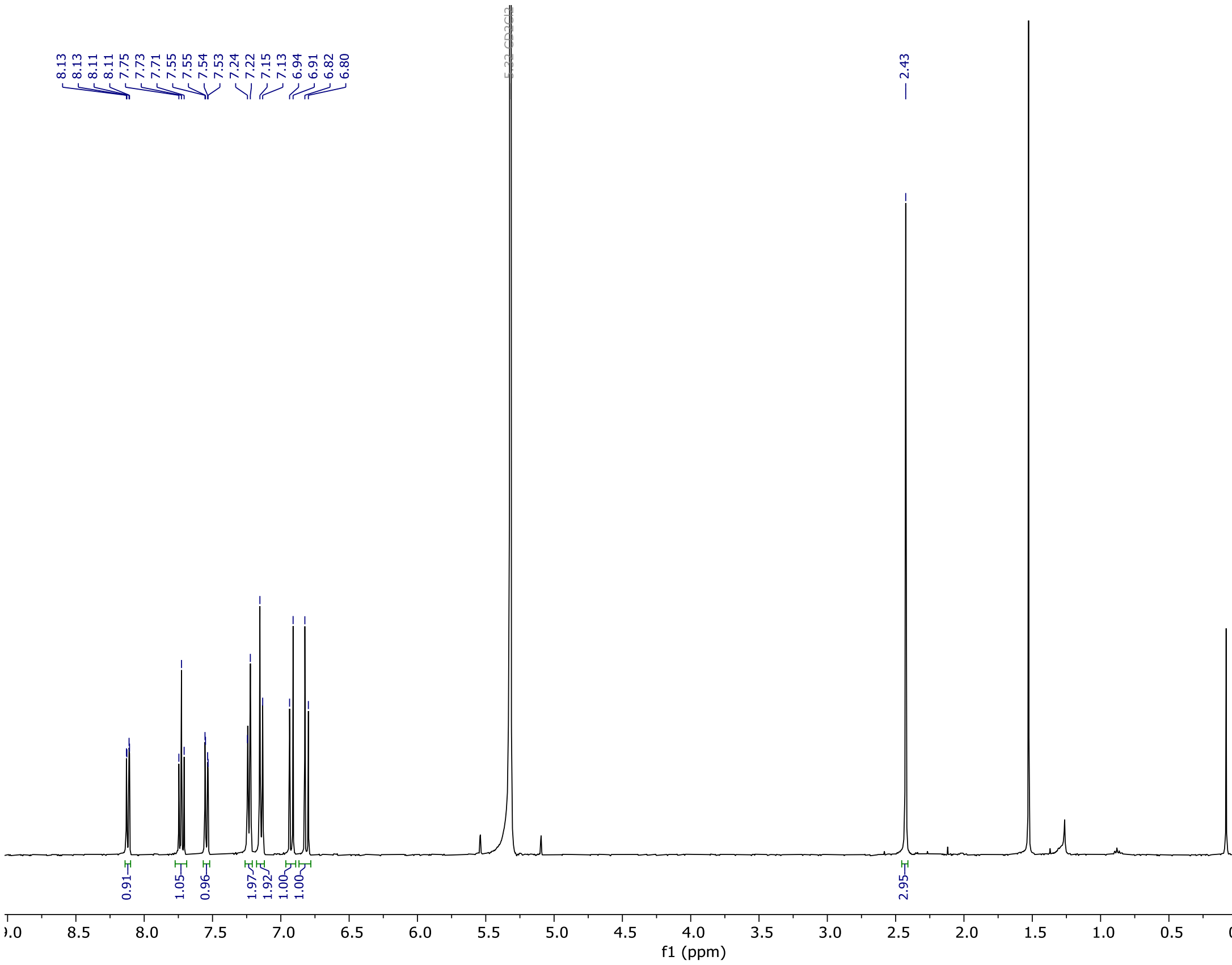

^{1}H NMR spectrum (400 MHz, CD_2Cl_2) of **5af**.

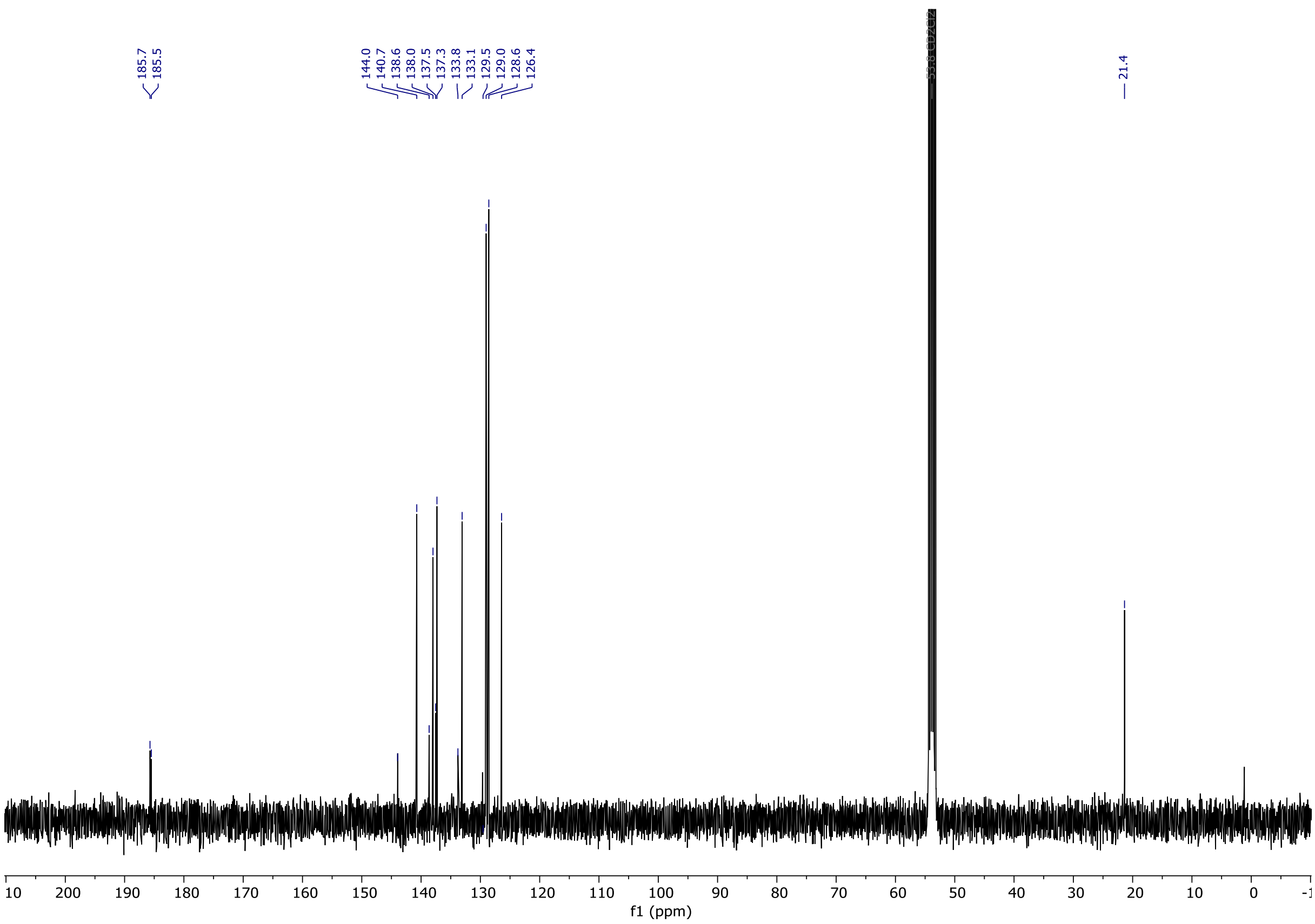

^{13}C NMR spectrum (101 MHz, CD_2Cl_2) of **5af**.

2-Methyl-5,8-diphenylnaphthalene-1,4-dione (**5ah**)

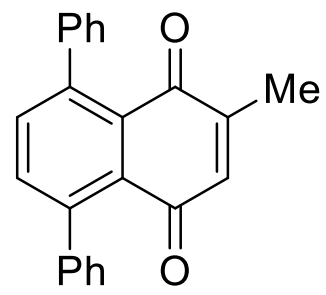

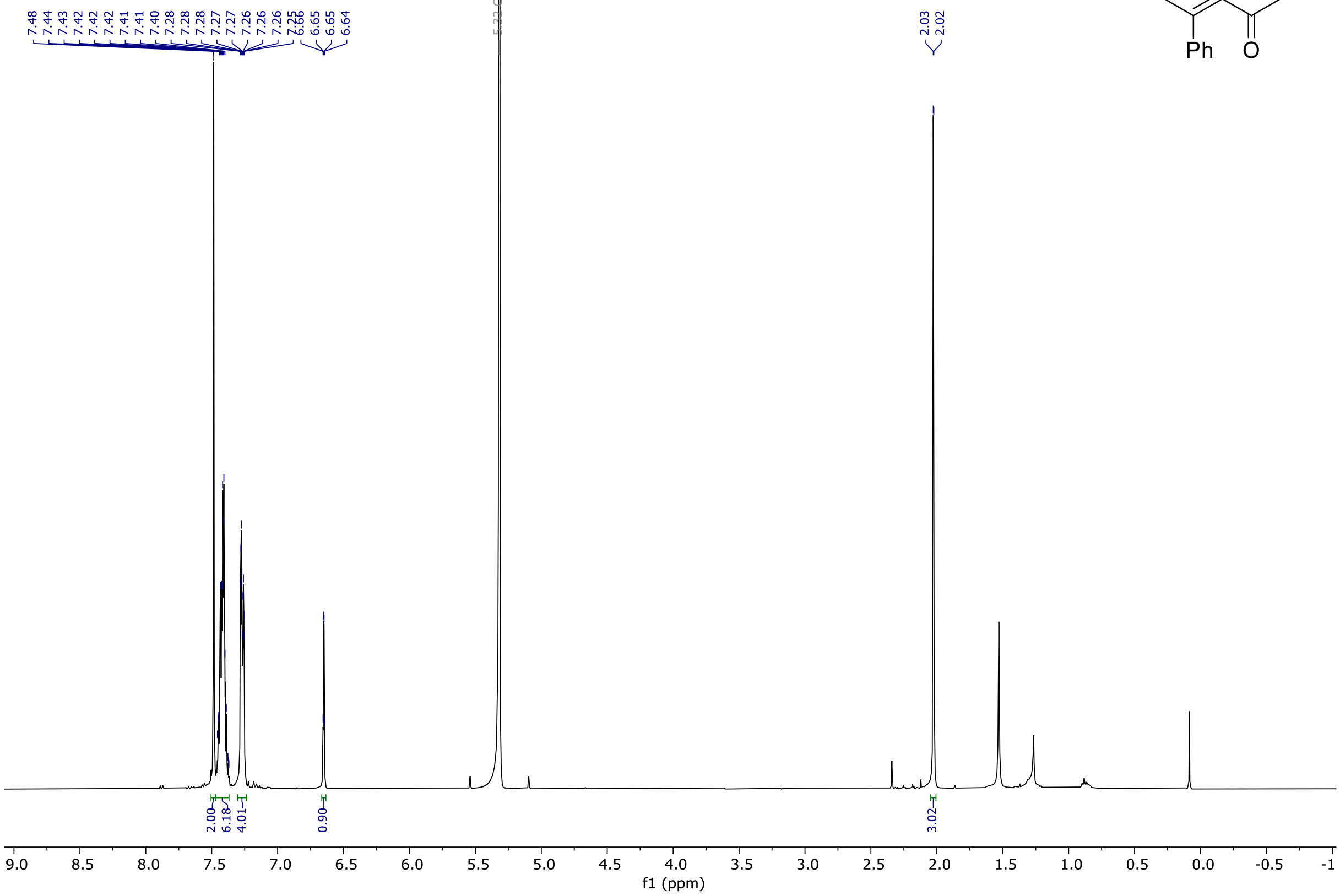

^{1}H NMR spectrum (400 MHz, CD_2Cl_2) of **5ah**.

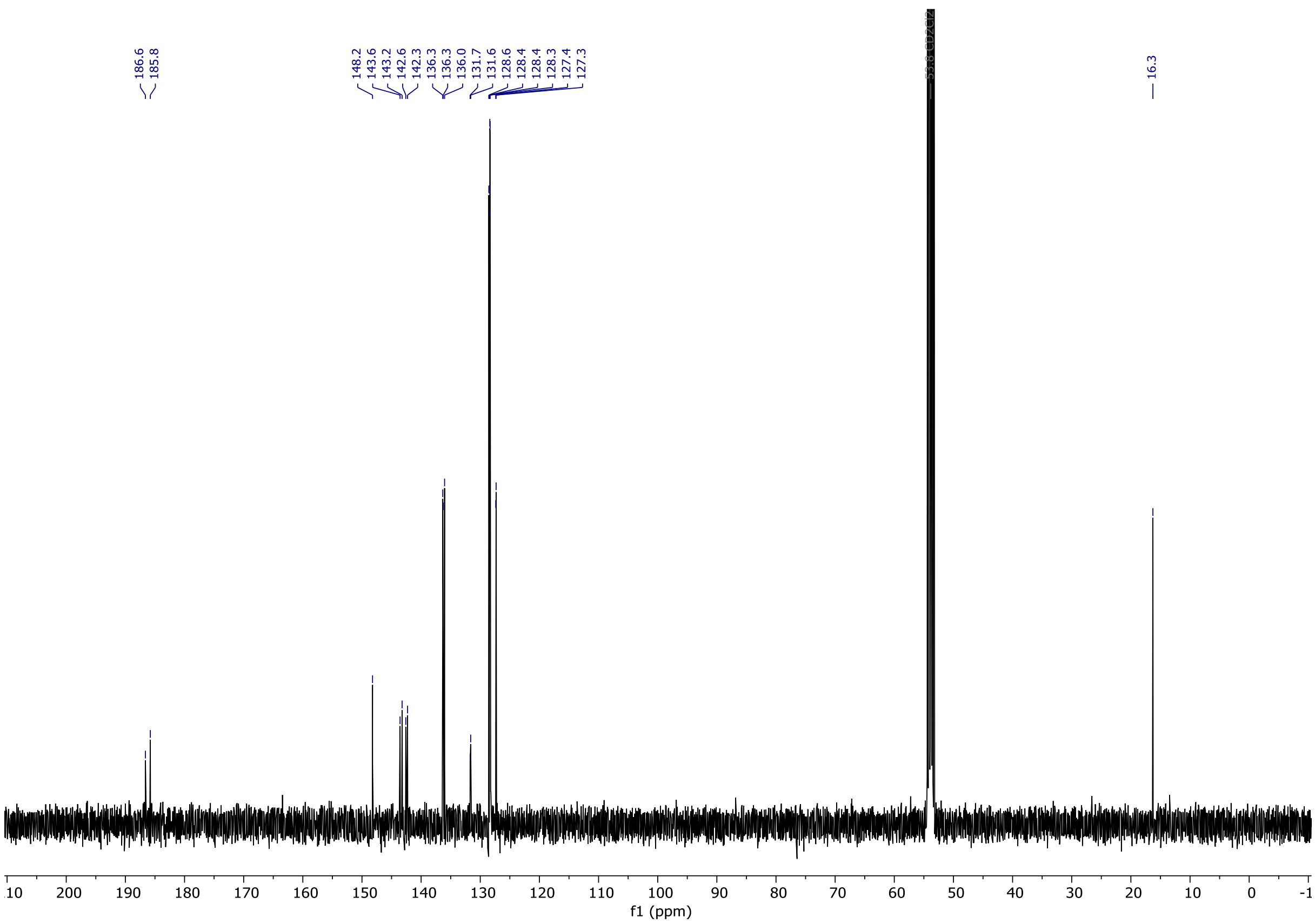

^{13}C NMR spectrum (101 MHz, CD_2Cl_2) of **5ah**.

2,3-Dimethyl-5,8-diphenylnaphthalene-1,4-dione (**5ai**)

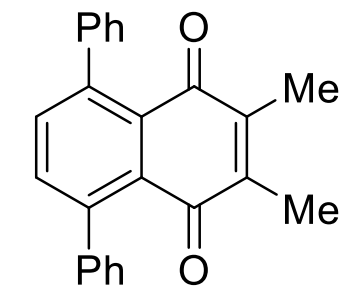

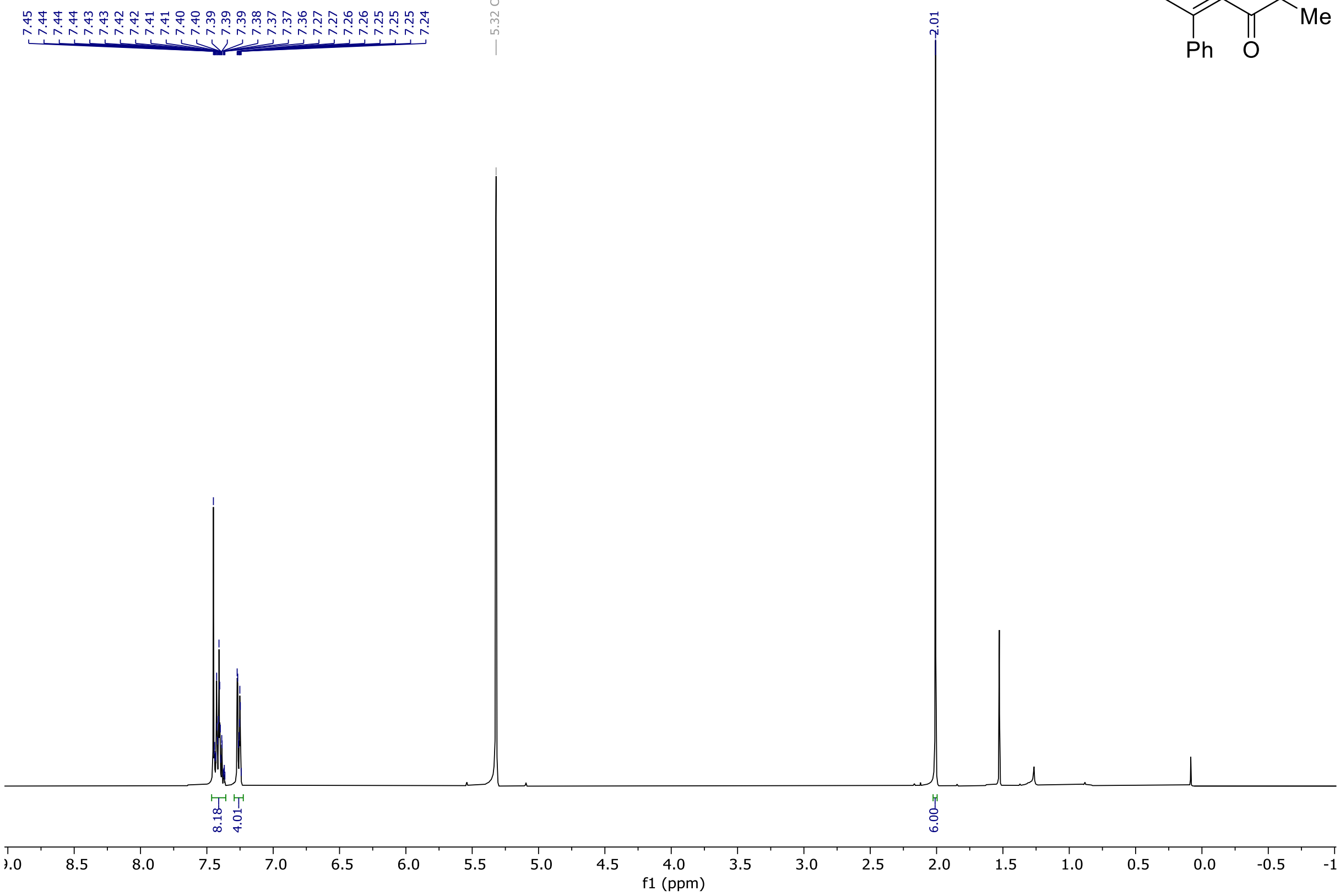

^{1}H NMR spectrum (400 MHz, CD_2Cl_2) of **5ai**.

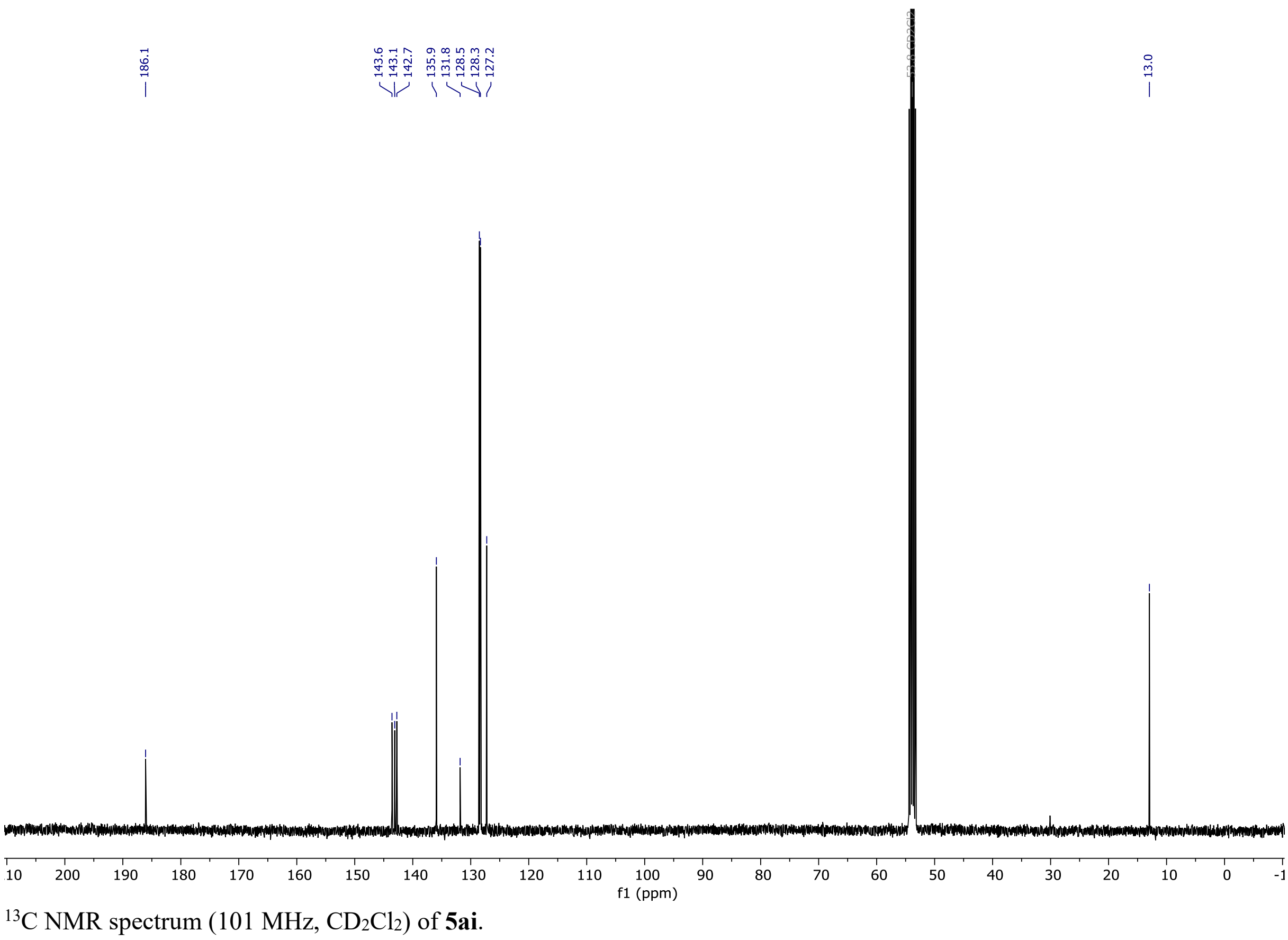

^{13}C NMR spectrum (101 MHz, CD_2Cl_2) of **5ai**.

2-Cyclopropyl-5,8-diphenylnaphthalene-1,4-dione (**5aj**)

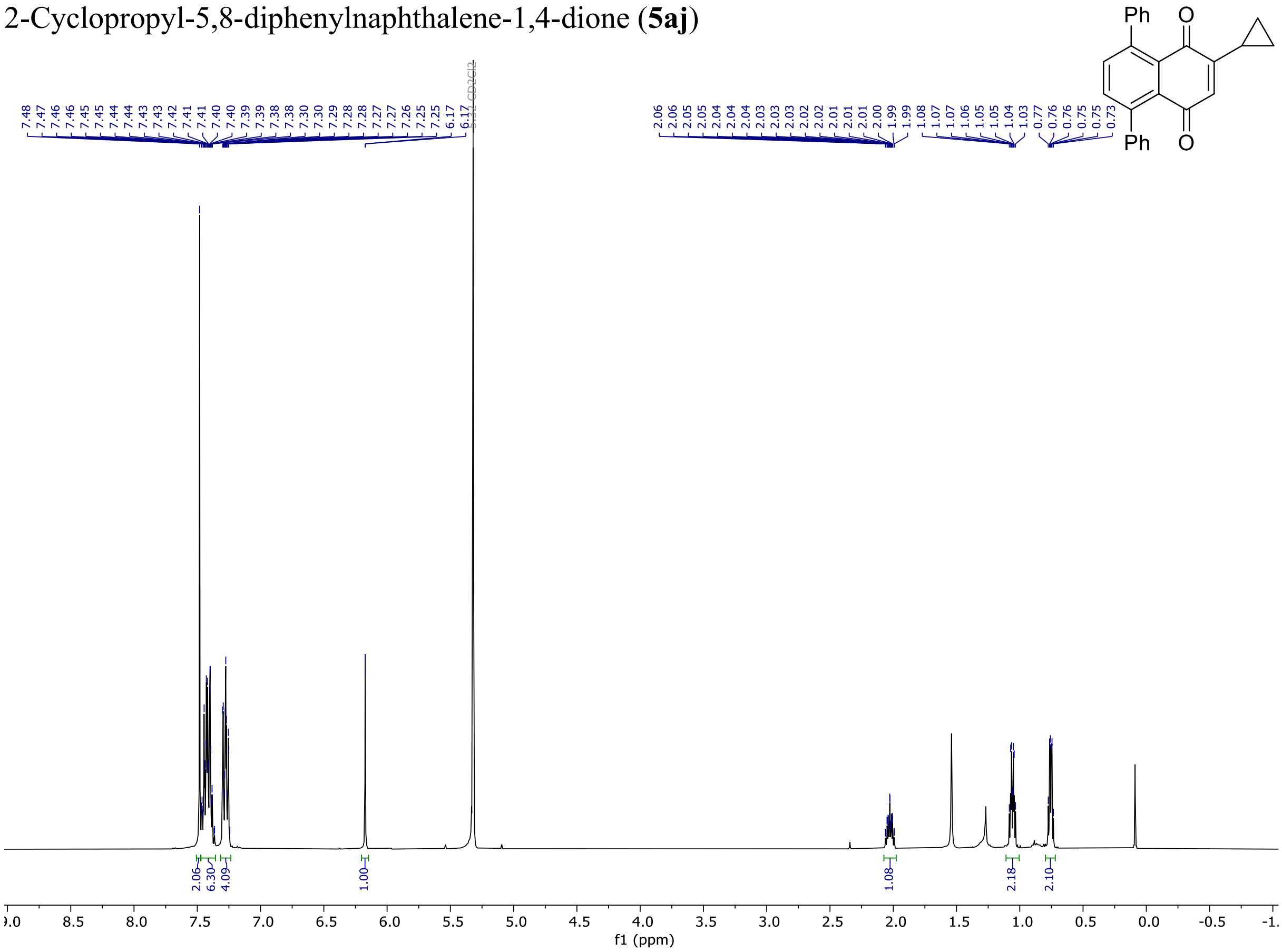

^{1}H NMR spectrum (400 MHz, CD_2Cl_2) of **5aj**.

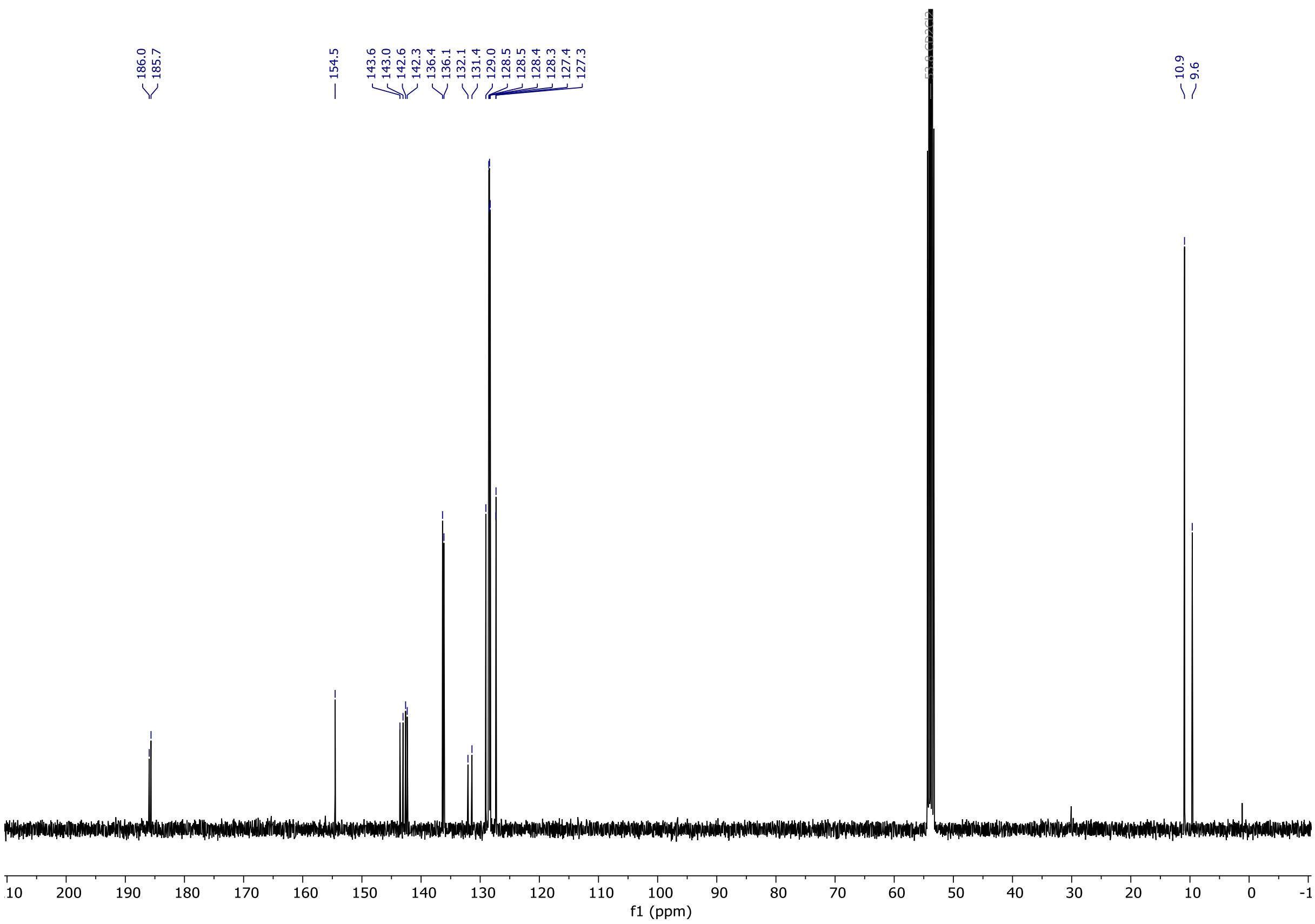

^{13}C NMR spectrum (101 MHz, CD_2Cl_2) of **5aj**.

2-Cyclohexyl-5,8-diphenylnaphthalene-1,4-dione (**5ak**)

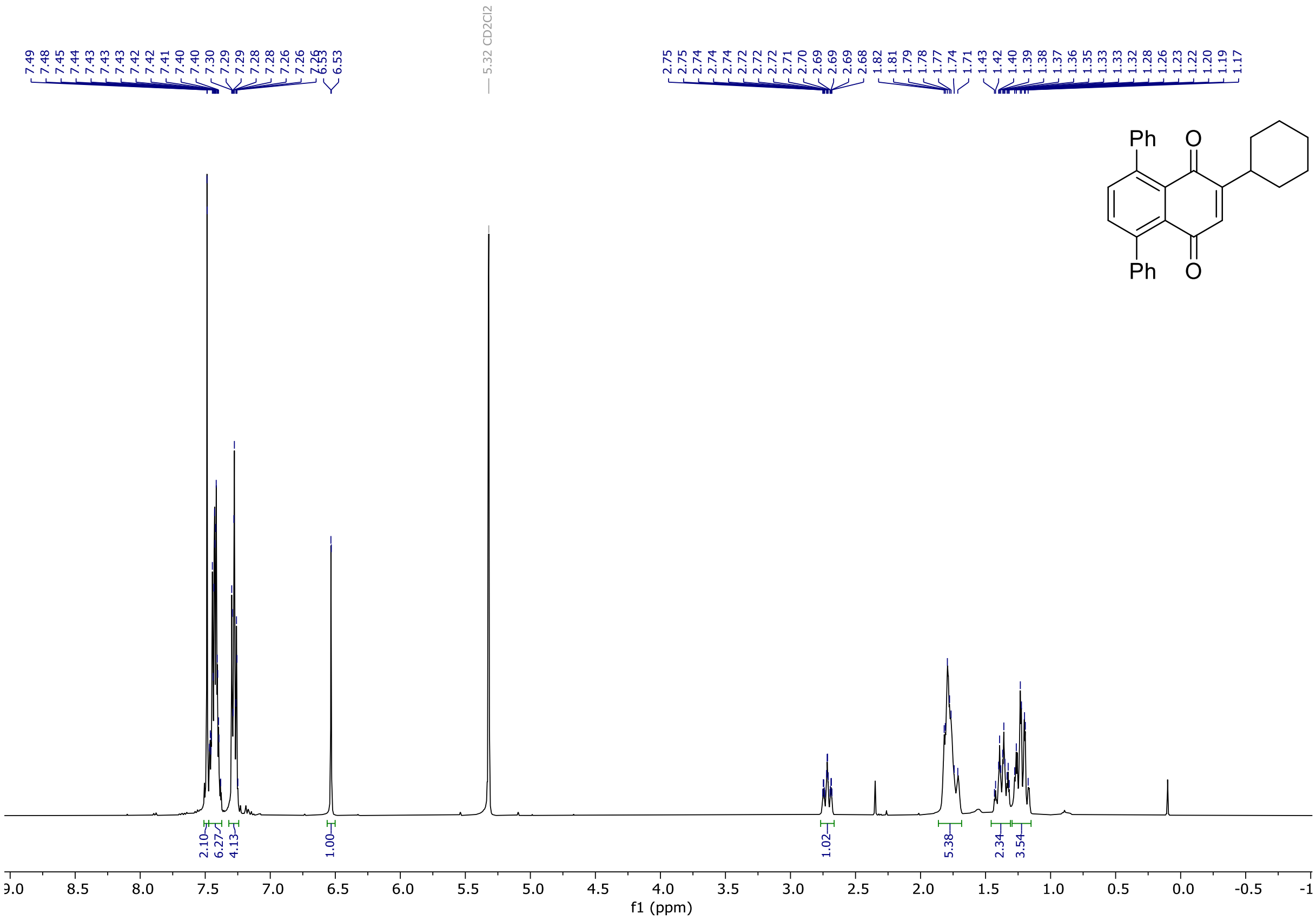

^{1}H NMR spectrum (400 MHz, CD_2Cl_2) of **5ak**.

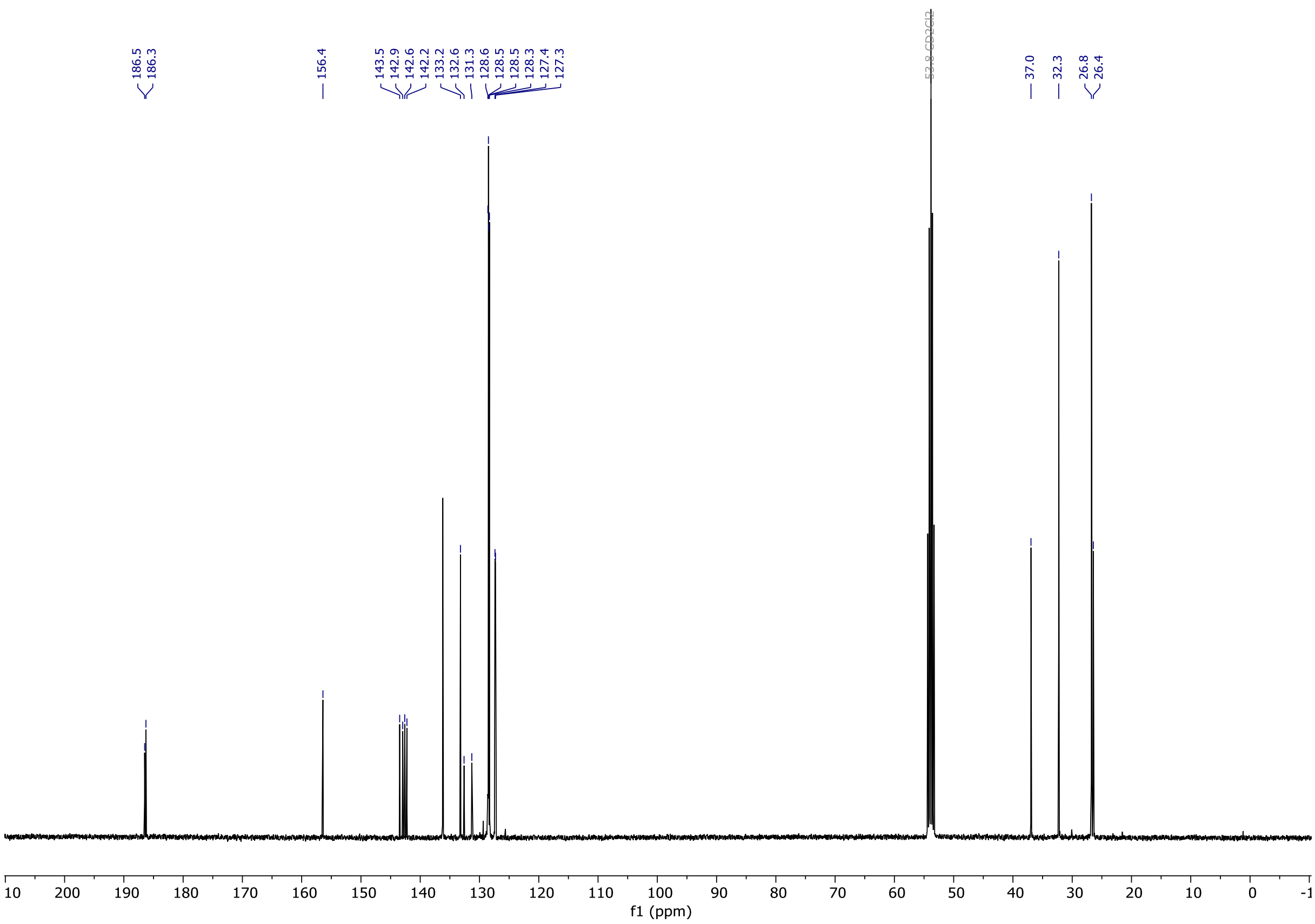

^{13}C NMR spectrum (101 MHz, CD_2Cl_2) of **5ak**.

2,5,8-Triphenylnaphthalene-1,4-dione (**5al**)

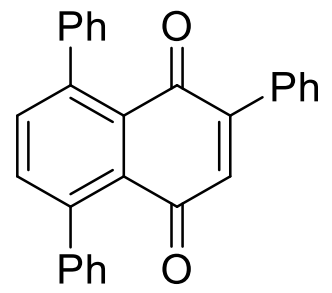

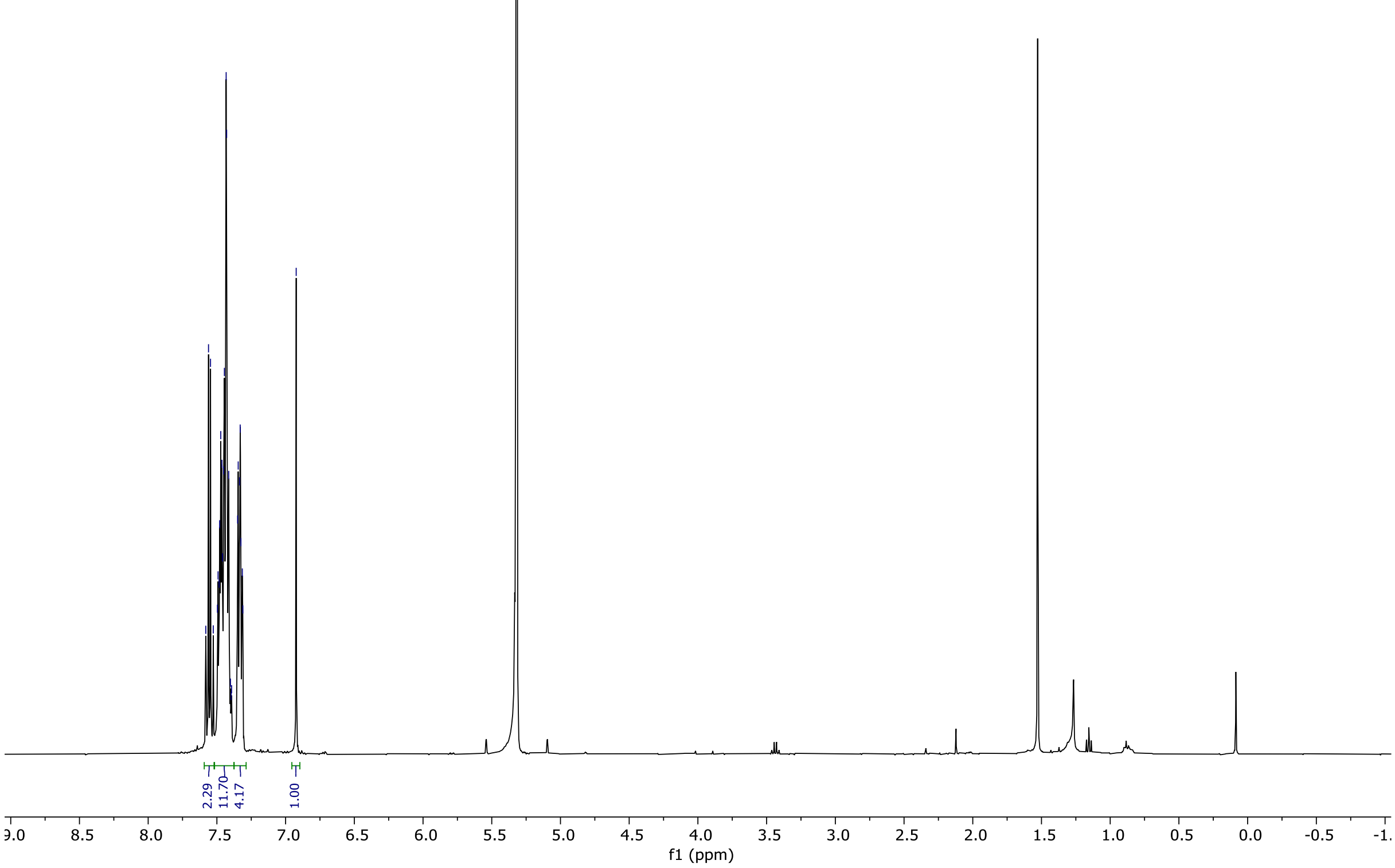

^{1}H NMR spectrum (400 MHz, CD_2Cl_2) of **5al**.

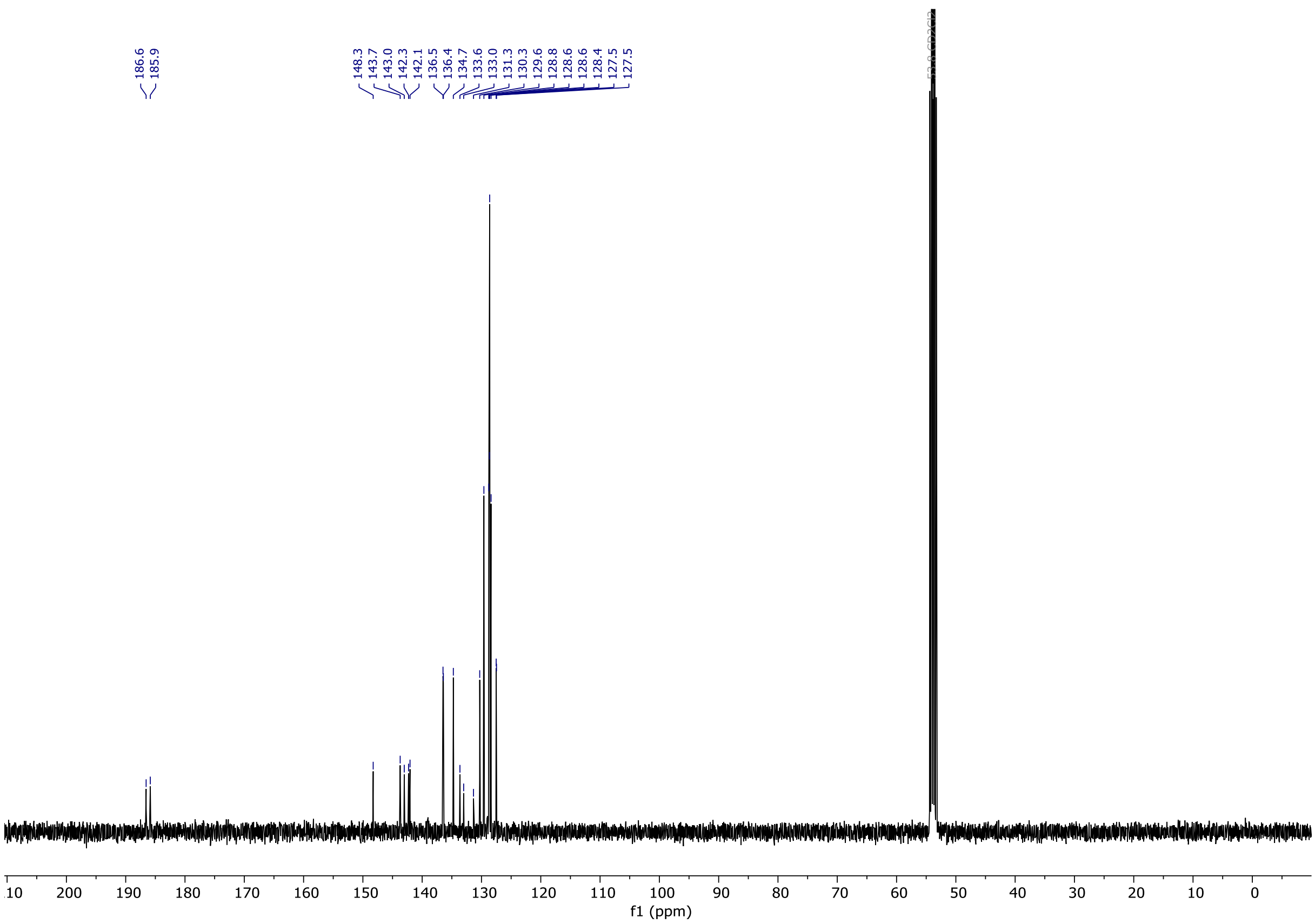

^{13}C NMR spectrum (101 MHz, CD_2Cl_2) of **5al**.

5,12-Dihydro-7,10-diphenyl-5,12[1',2']benzenonaphthacene-6,11-dione (**5am**)

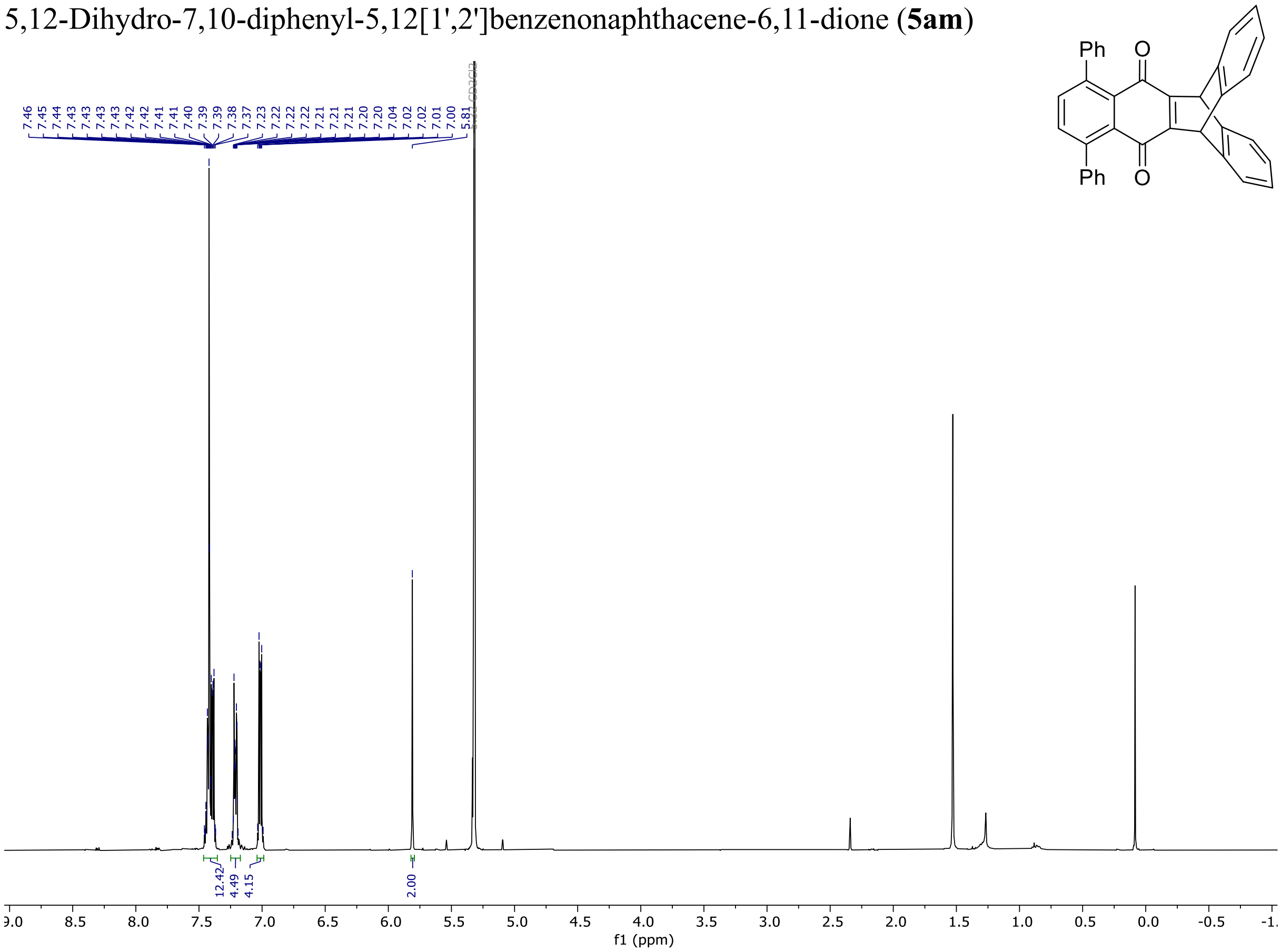

^{1}H NMR spectrum (400 MHz, CD_2Cl_2) of **5am**.

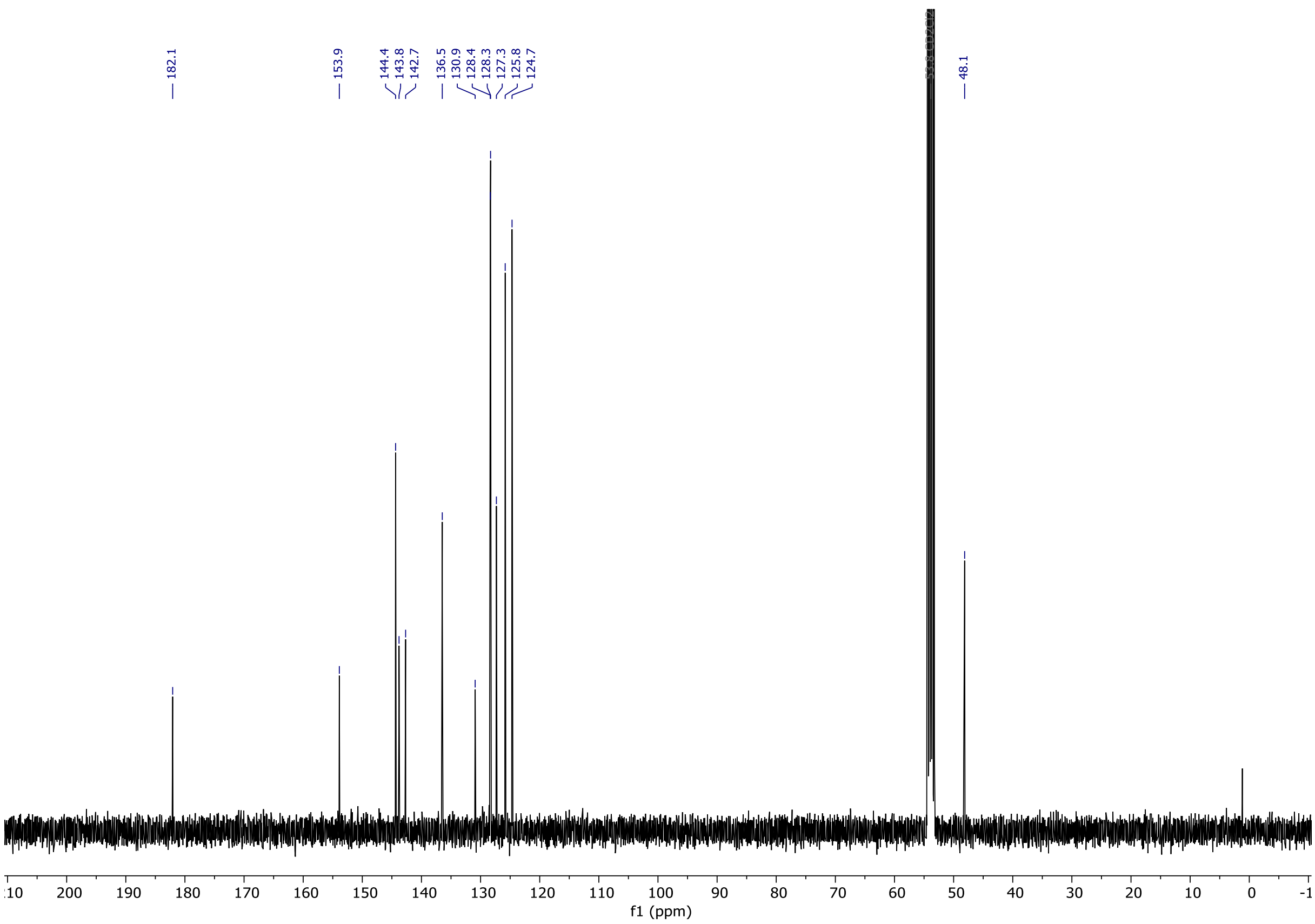

^{13}C NMR spectrum (101 MHz, CD_2Cl_2) of **5am**.

2-Chloro-5,8-diphenylnaphthalene-1,4-dione (**5an**)

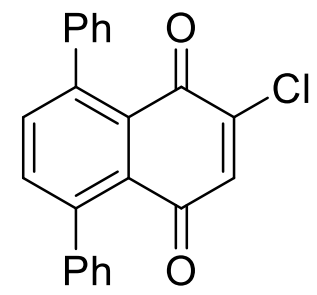

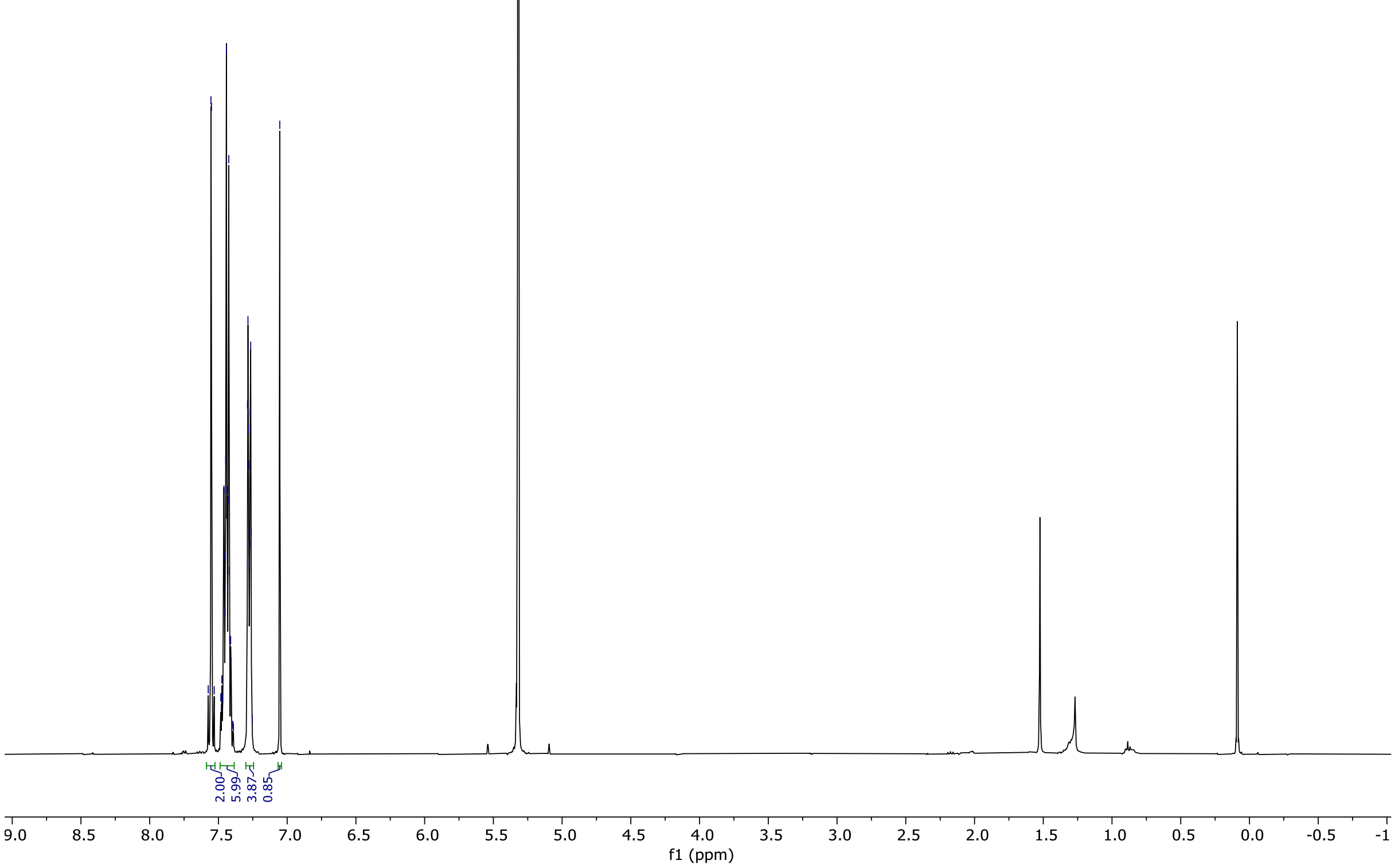

^{1}H NMR spectrum (400 MHz, CD_2Cl_2) of **5an**.

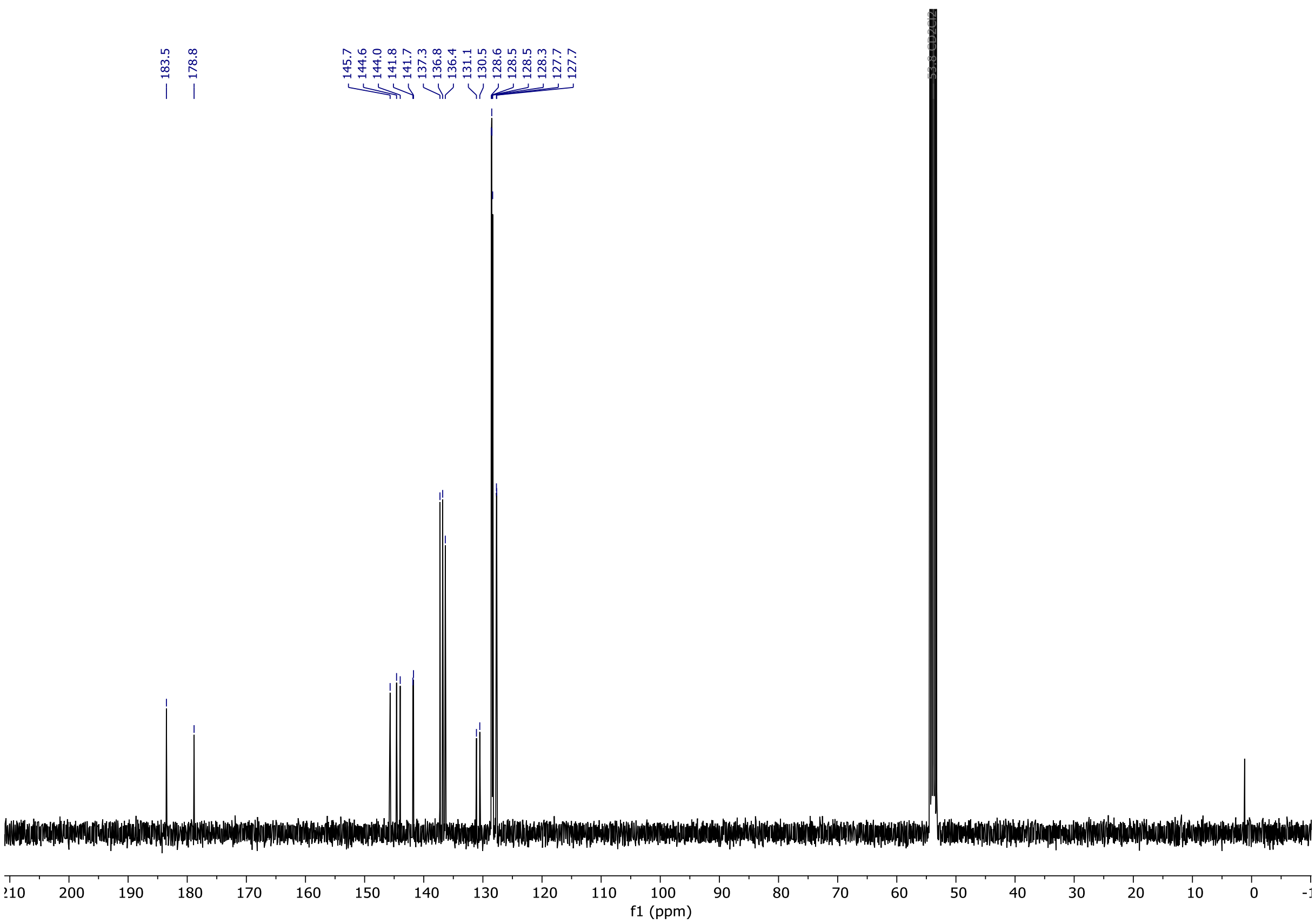

^{13}C NMR spectrum (101 MHz, CD_2Cl_2) of **5an**.

2-Acetamido-5,8-diphenylnaphthalene-1,4-dione (**5ao**)

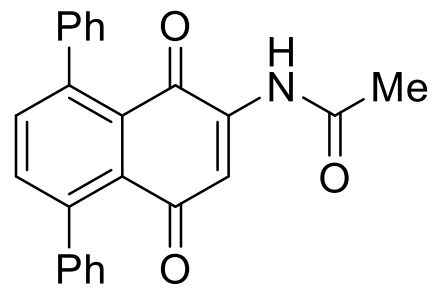

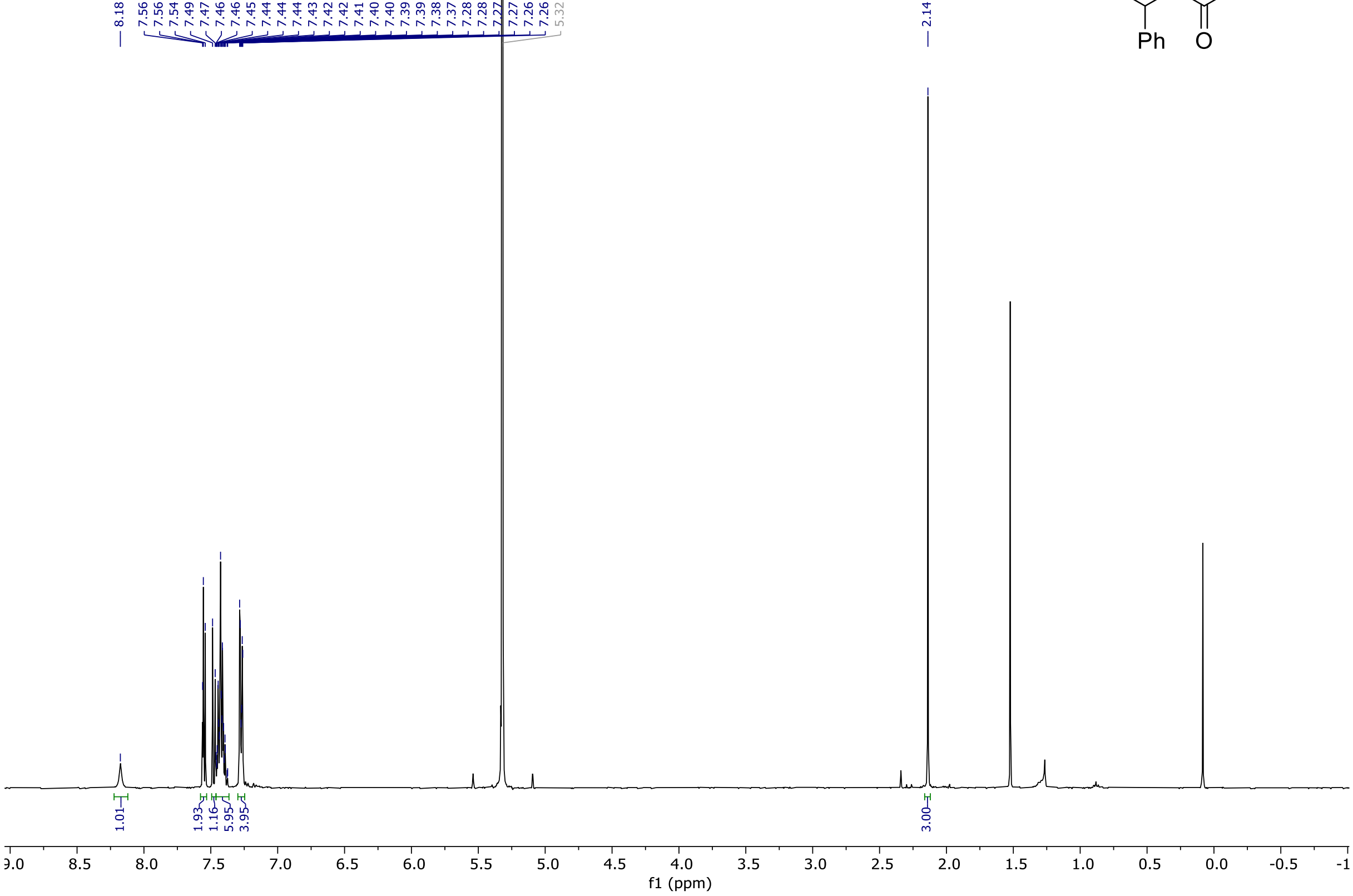

^{1}H NMR spectrum (400 MHz, CD_2Cl_2) of **5ao**.

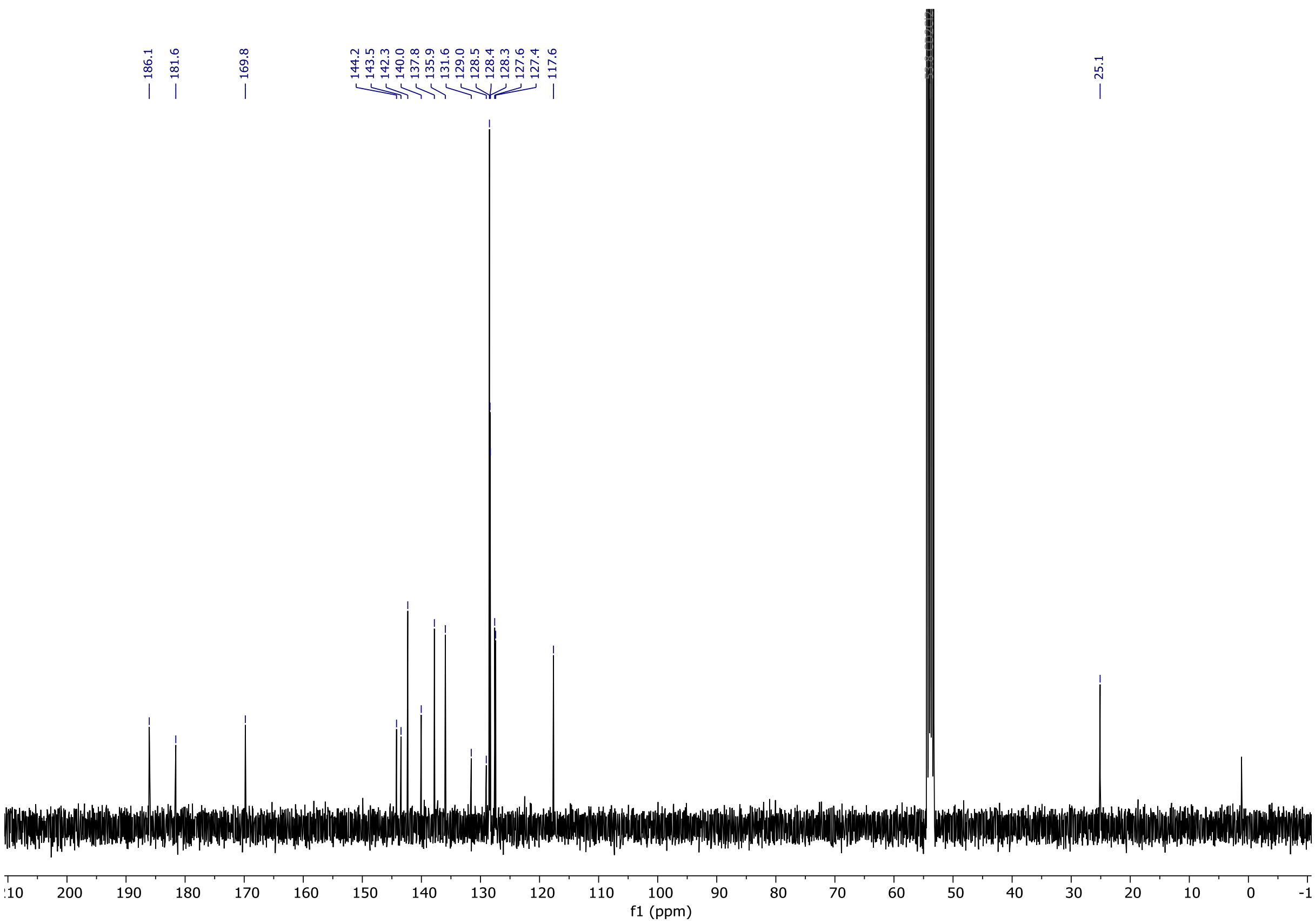

^{13}C NMR spectrum (101 MHz, CD_2Cl_2) of **5ao**.

1,4,4a,8a-Tetrahydro-1,4-ethanonaphthalene-5,8-dione (**6a**)

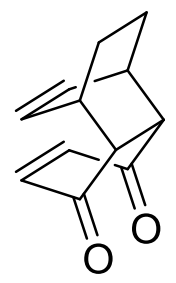

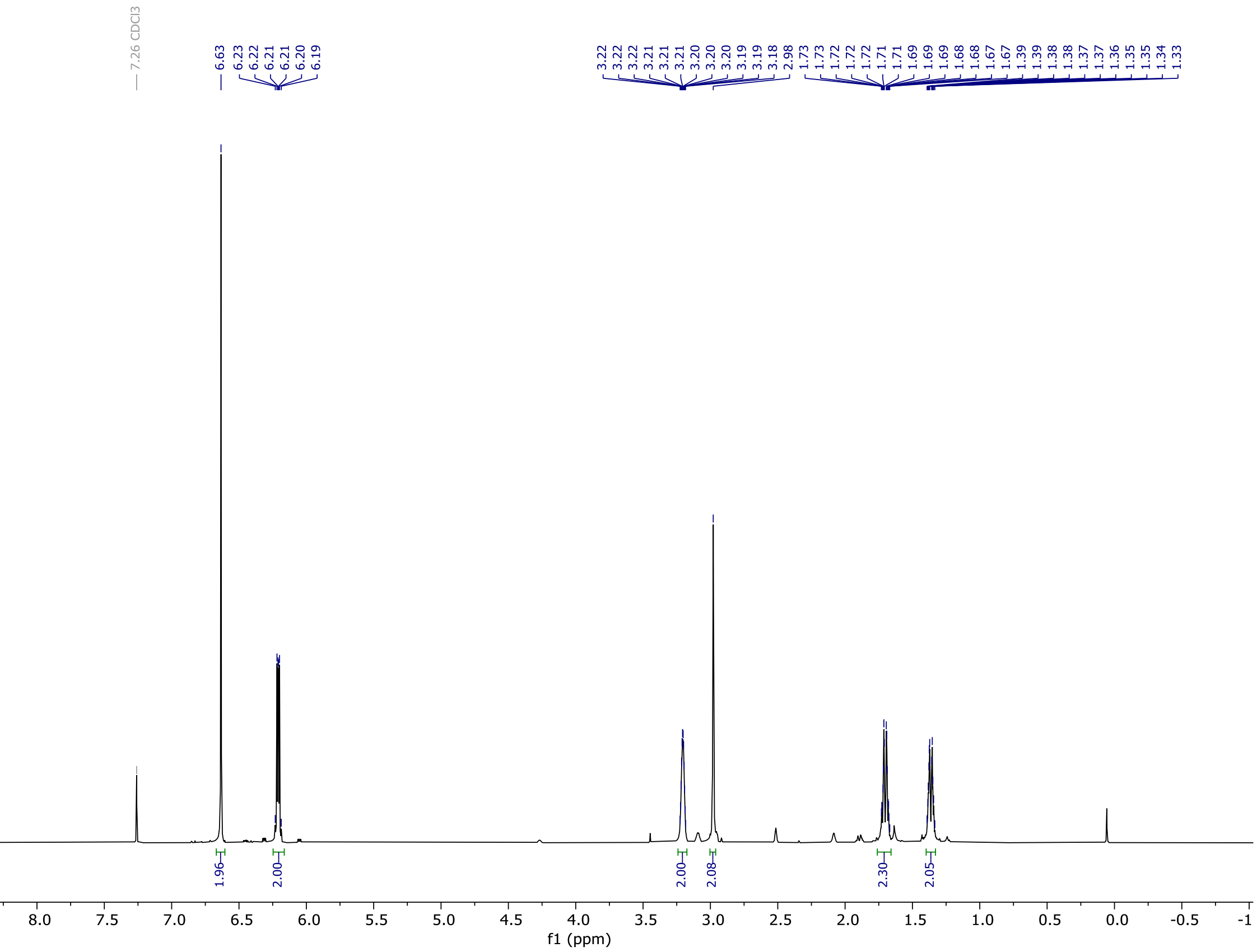

^{1}H NMR spectrum (400 MHz, CDCl3) of **6a**.

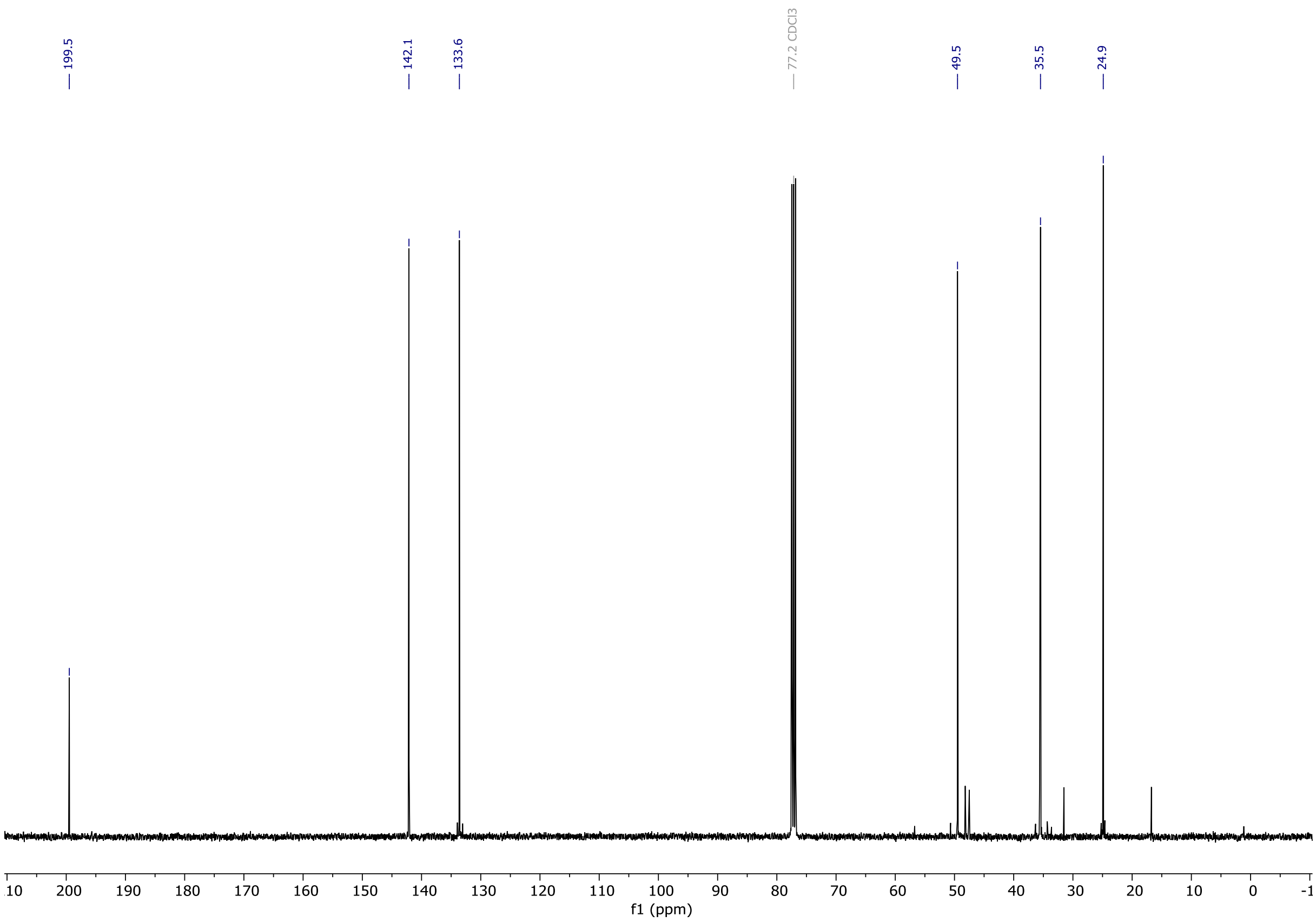

^{13}C NMR spectrum (101 MHz, CDCl3) of **6a**.

1,4,4a,8a-Tetrahydro-6-methyl-1,4-ethanonaphthalene-5,8-dione (**6b**)

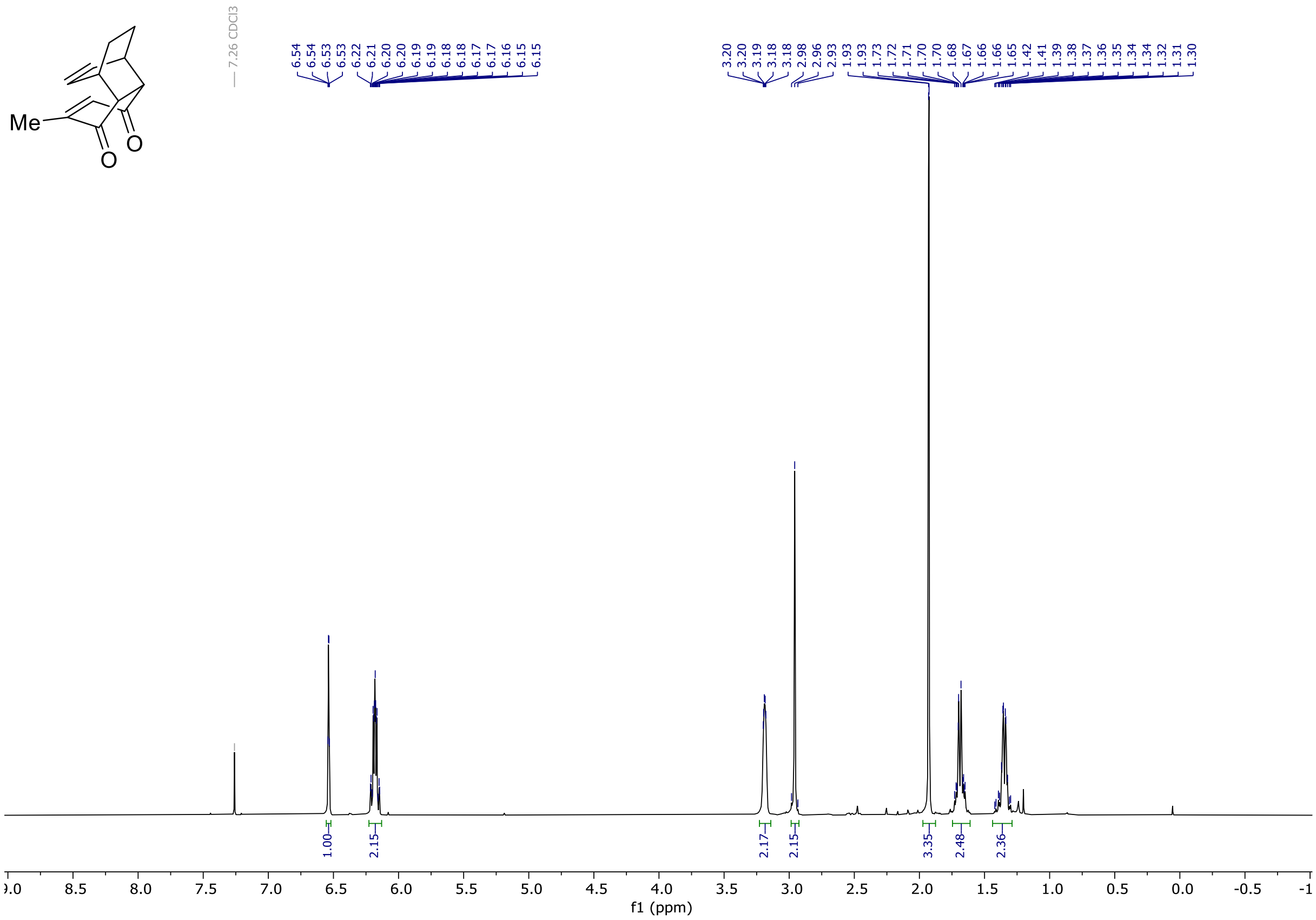

^{1}H NMR spectrum (400 MHz, $CDCl_3$) of **6b**.

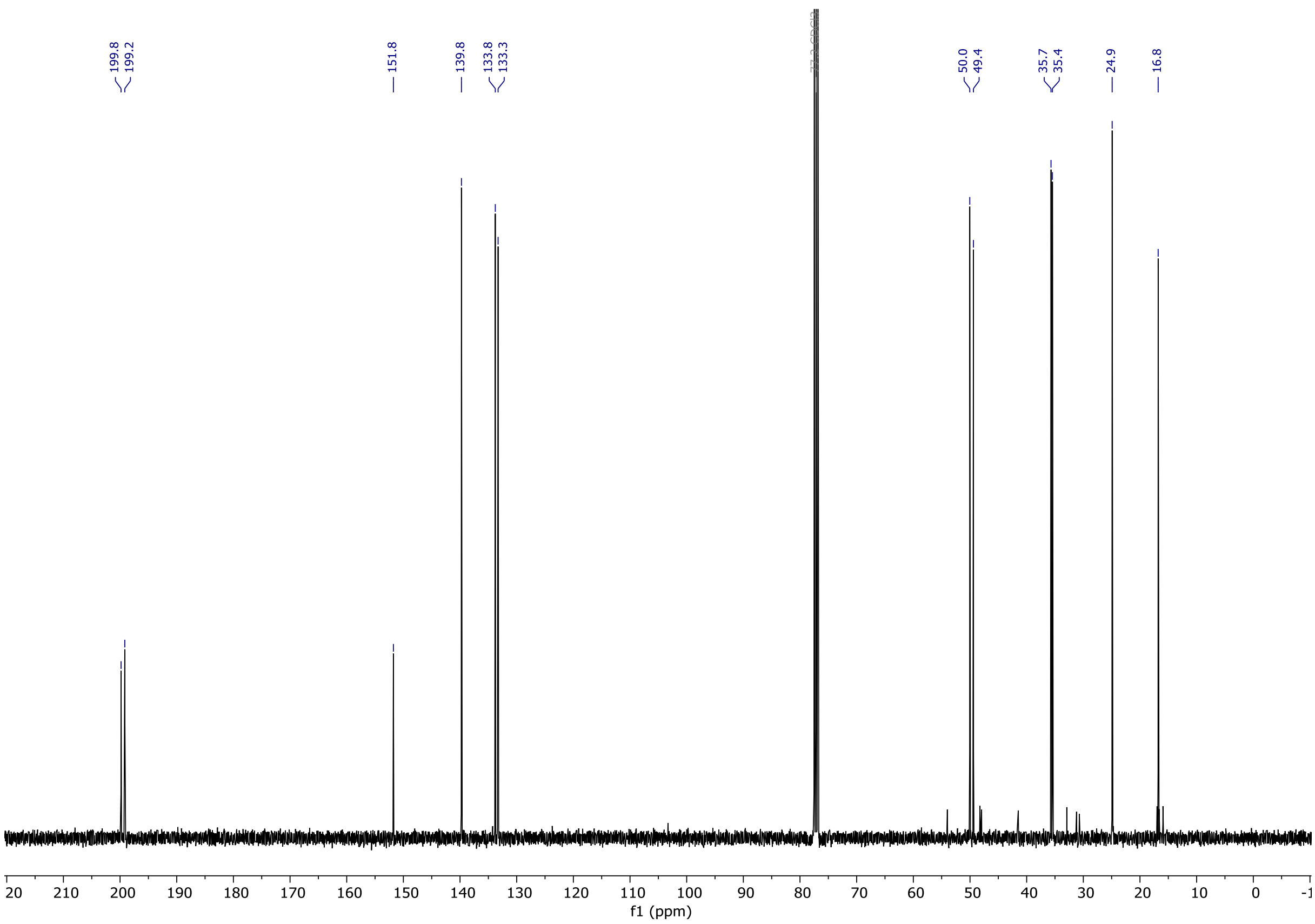

^{13}C NMR spectrum (101 MHz, $CDCl_3$) of **6b**.

1,4,4a,8a-Tetrahydro-4a,7-dimethyl-1,4-ethanonaphthalene-5,8-dione (**6c**)

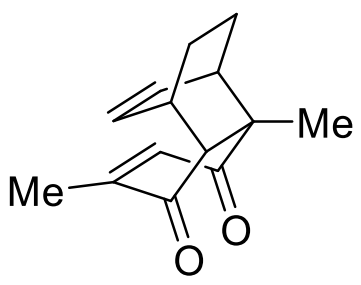

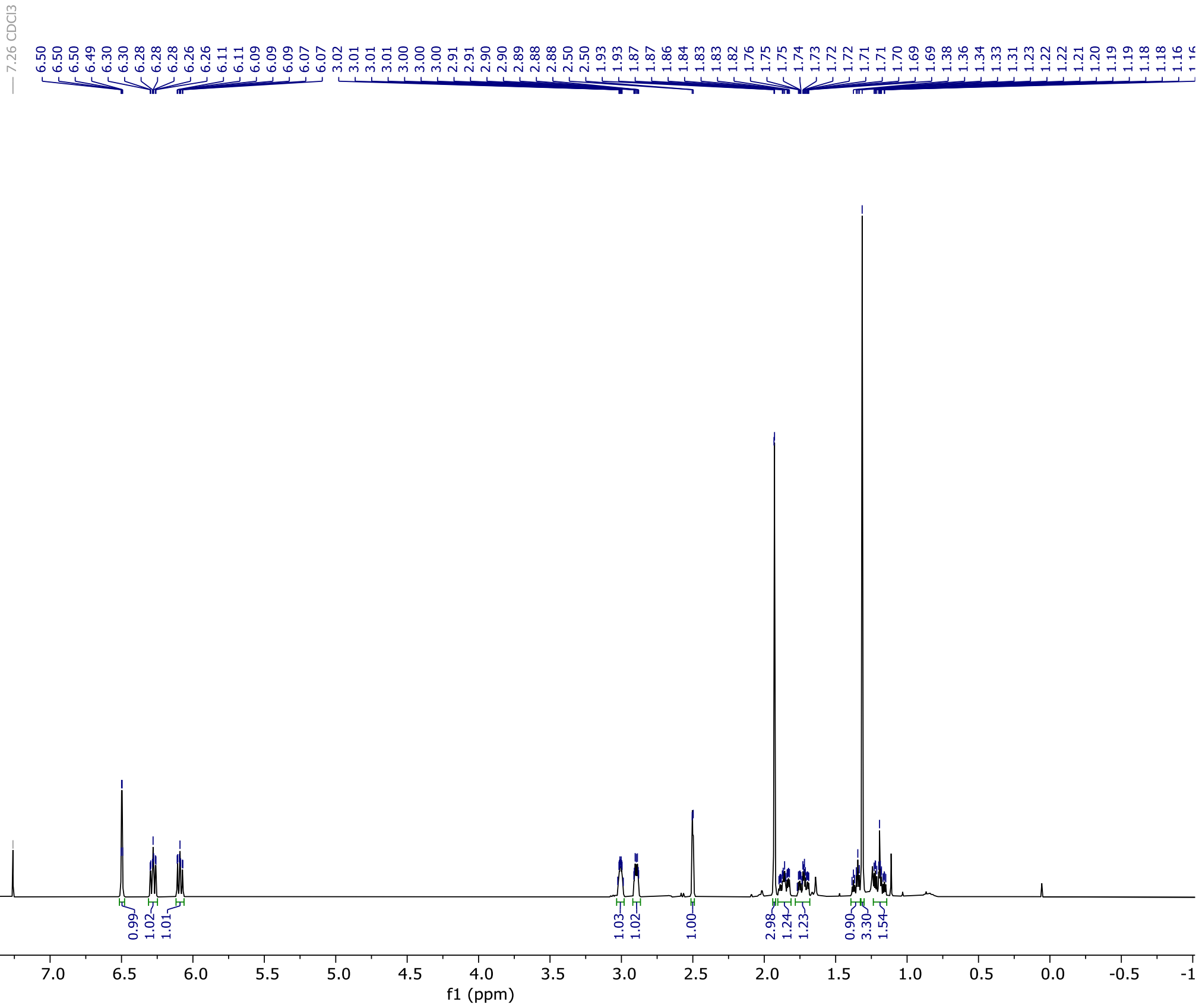

^{13}C NMR spectrum (101 MHz, $CDCl_3$) of **6c**.

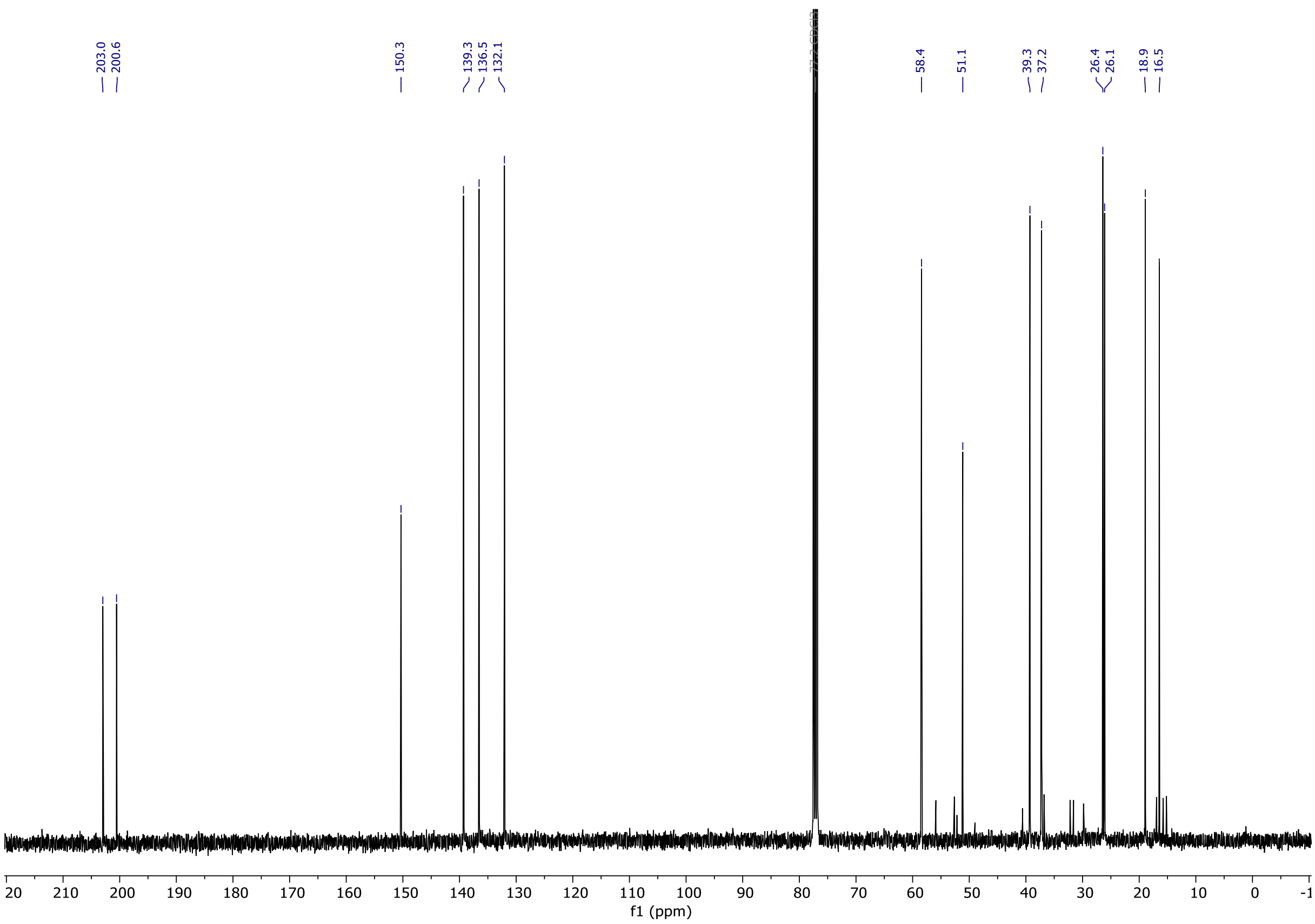

^{13}C NMR spectrum (101 MHz, $CDCl_3$) of **6c**.

1,4,4a,8a-Tetrahydro-6-acetamido-1,4-ethanonaphthalene-5,8-dione (**6d**)

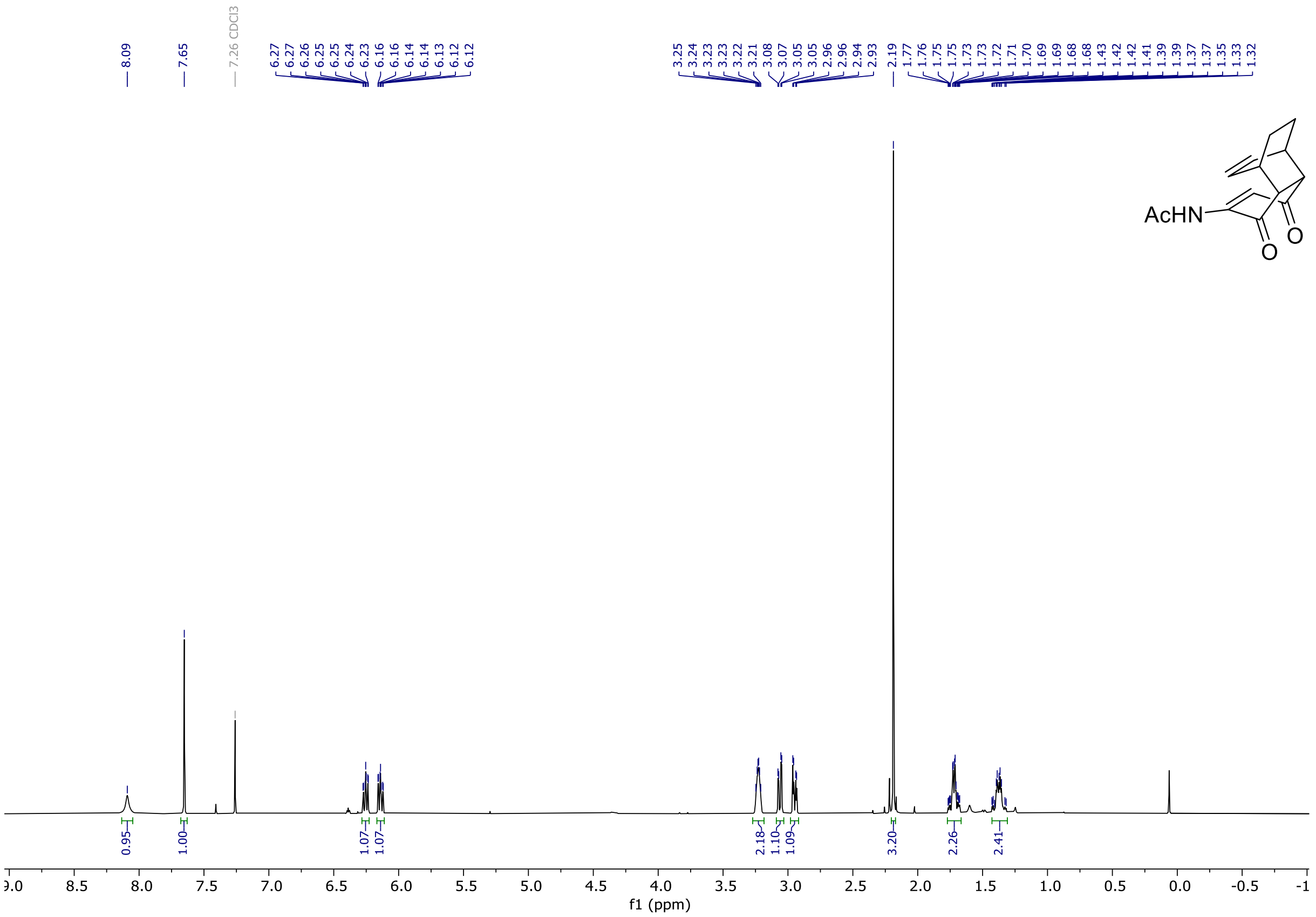

^{1}H NMR spectrum (400 MHz, $CDCl_3$) of **6d**.

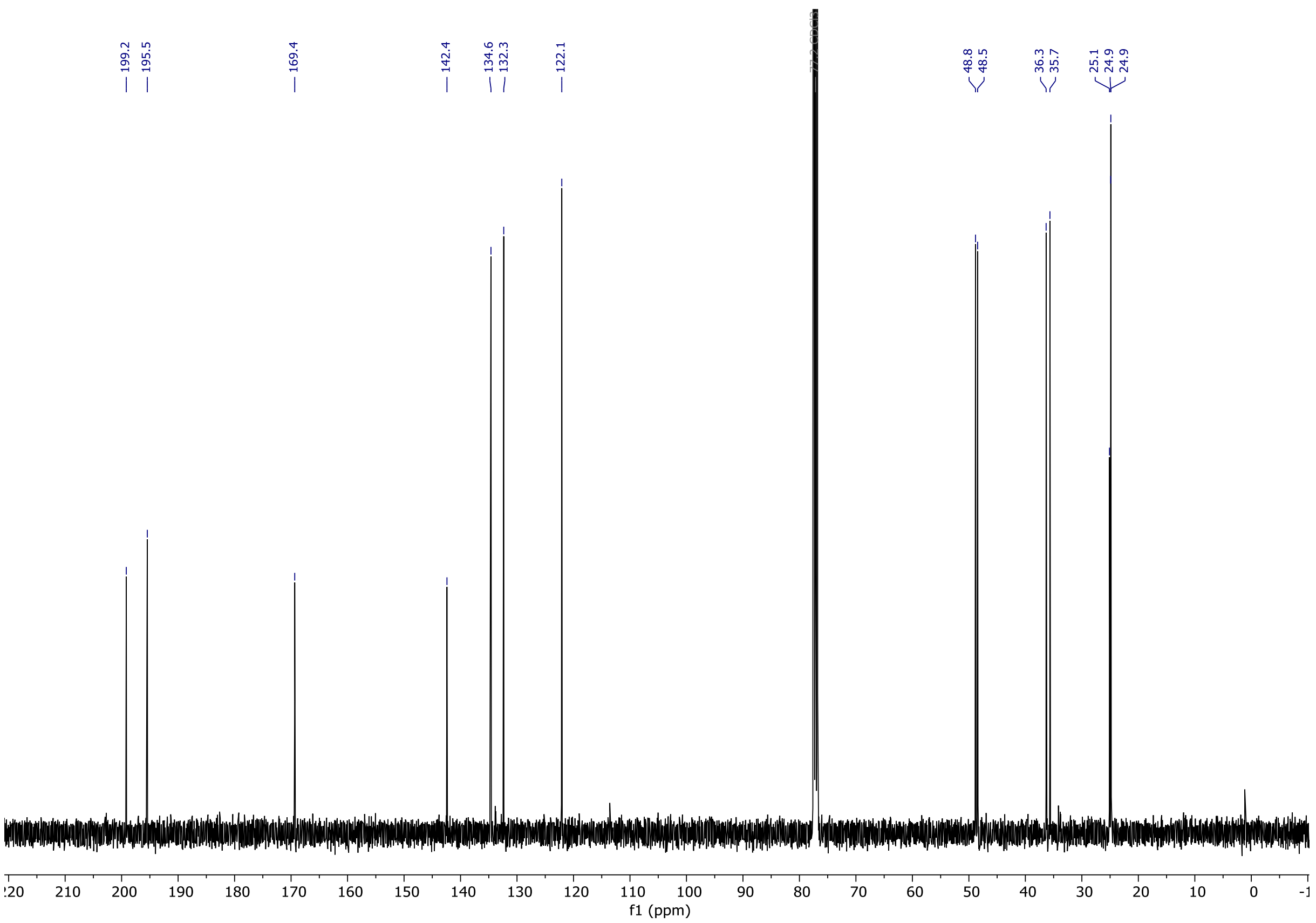

^{13}C NMR spectrum (101 MHz, $CDCl_3$) of **6d**.

1,4,4a,5,8,9a-Hexahydro-1,4-ethano-5,8-diphenylanthracene-9,10-dione (‘*bottom*’-*endo*-**6e**)

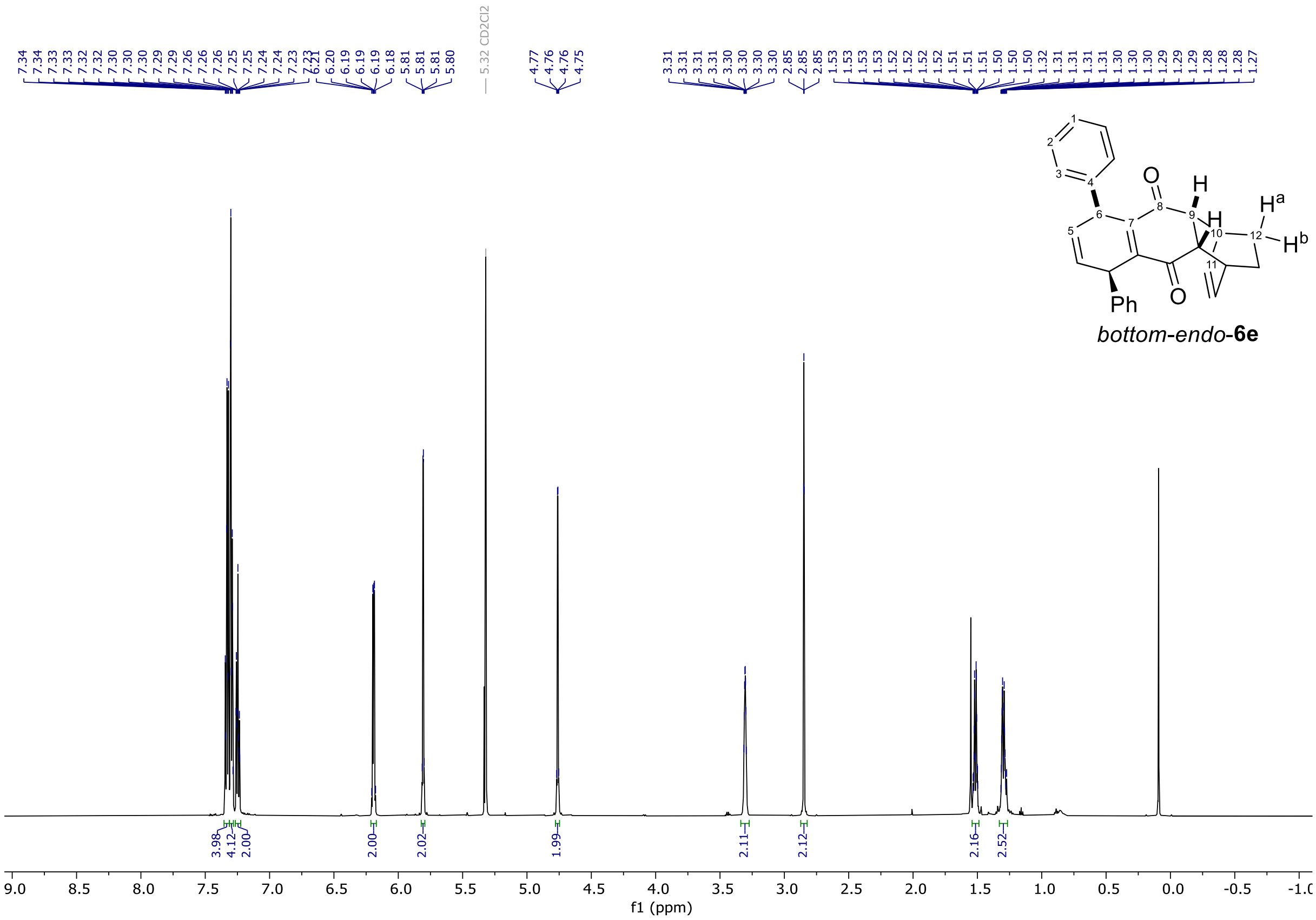

^{1}H NMR spectrum (600 MHz, CD_2Cl_2) of ‘*bottom*’-*endo*-**6e**.

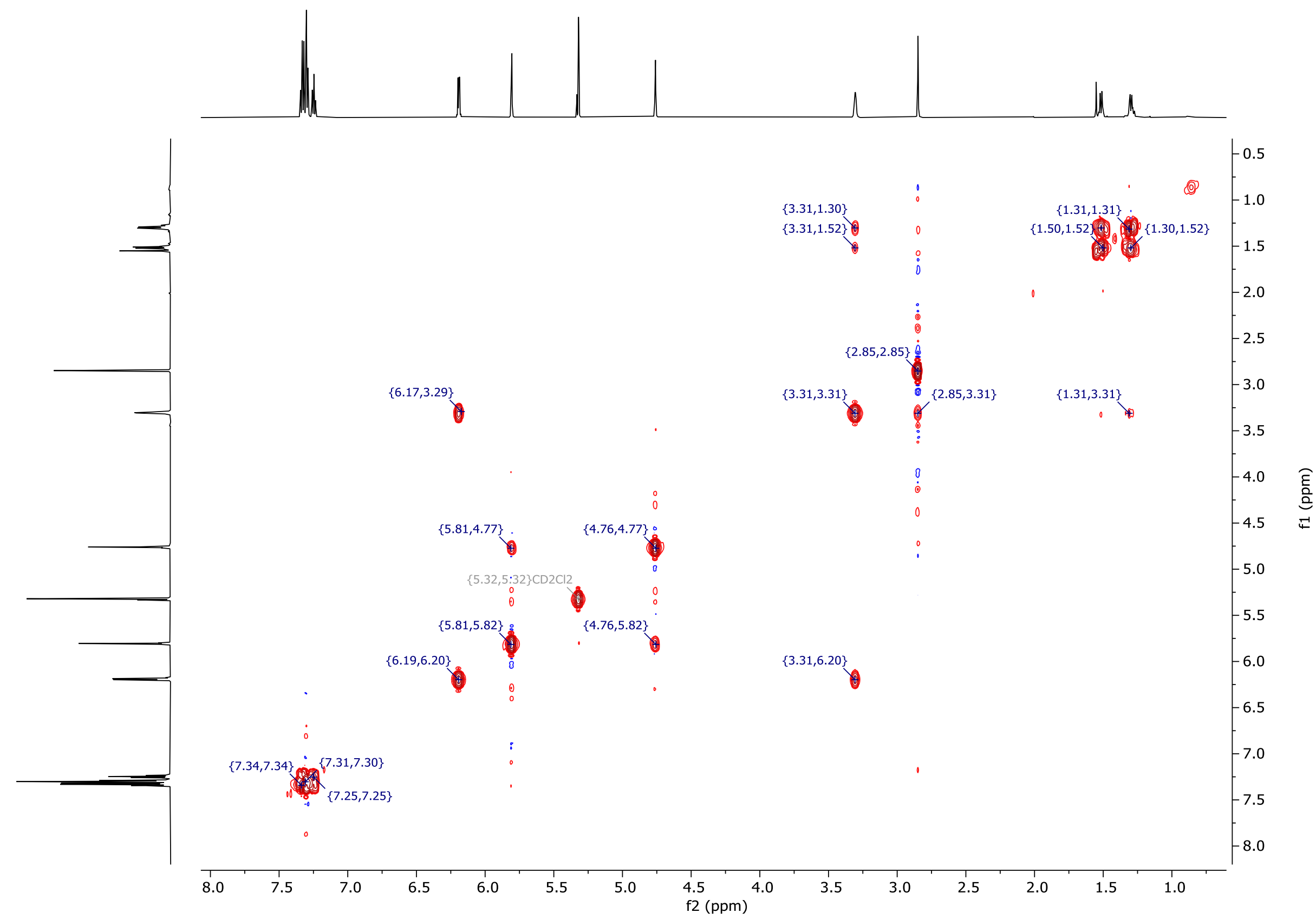

$^{1}H,^{1}H$ COSY NMR spectrum (600 MHz, CD_2Cl_2) of ‘*bottom*’-*endo*-**6e**.

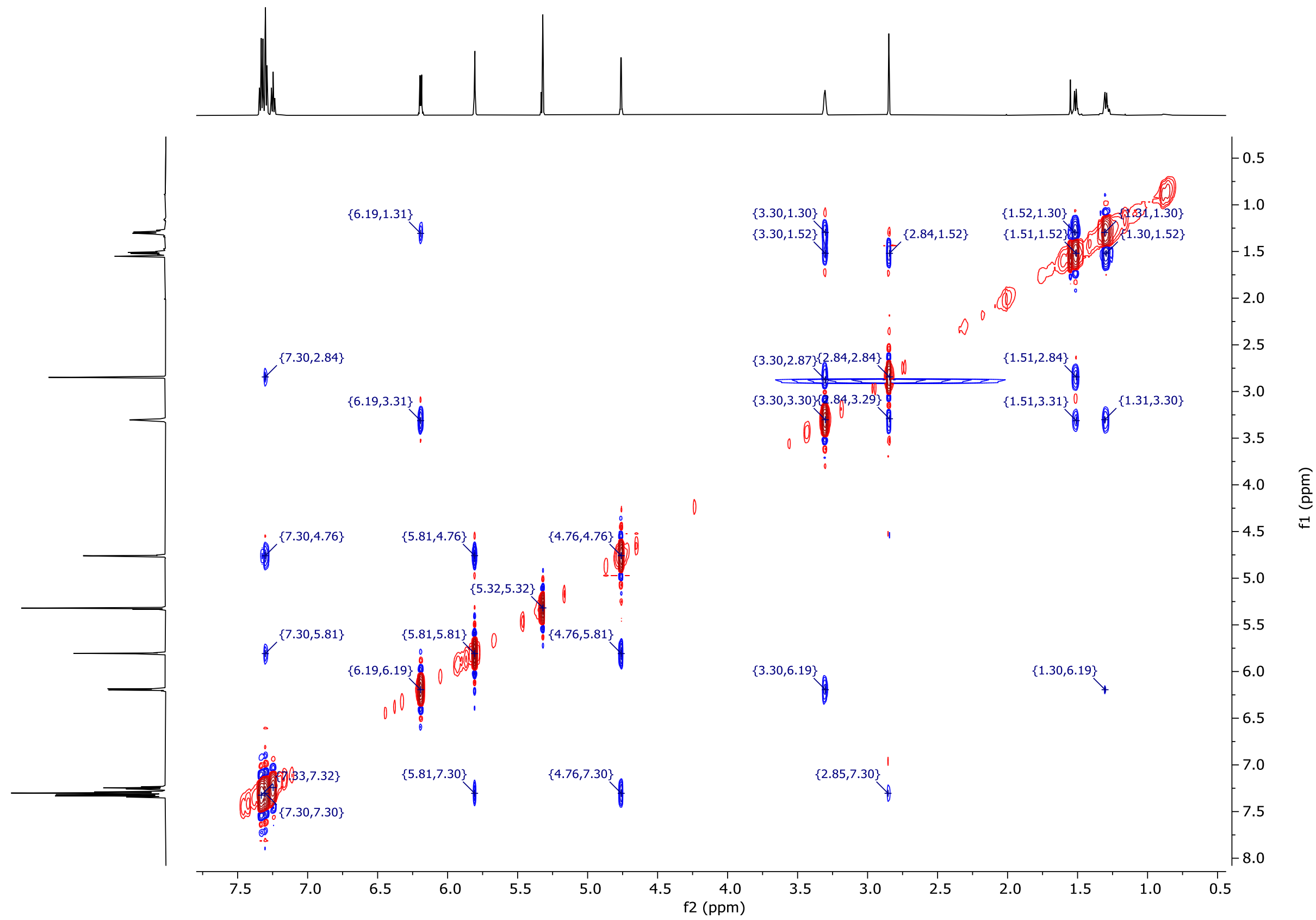

^{1}H,^{1}H NOESY NMR spectrum (600 MHz, CD_2Cl_2) of ‘*bottom*’-*endo*-**6e**.

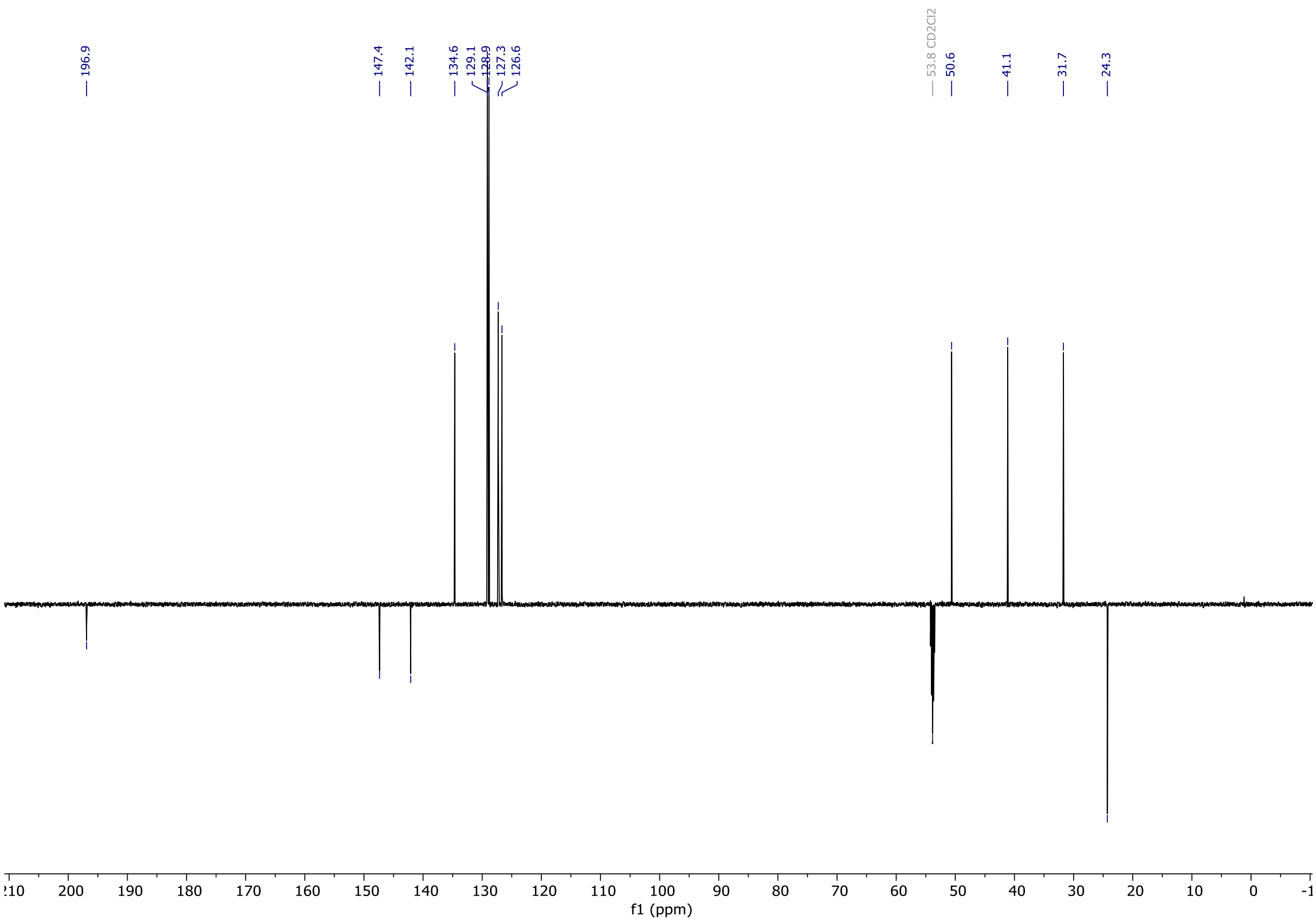

^{13}C APT NMR spectrum (151 MHz, CD_2Cl_2) of ‘*bottom*’-*endo*-**6e**.

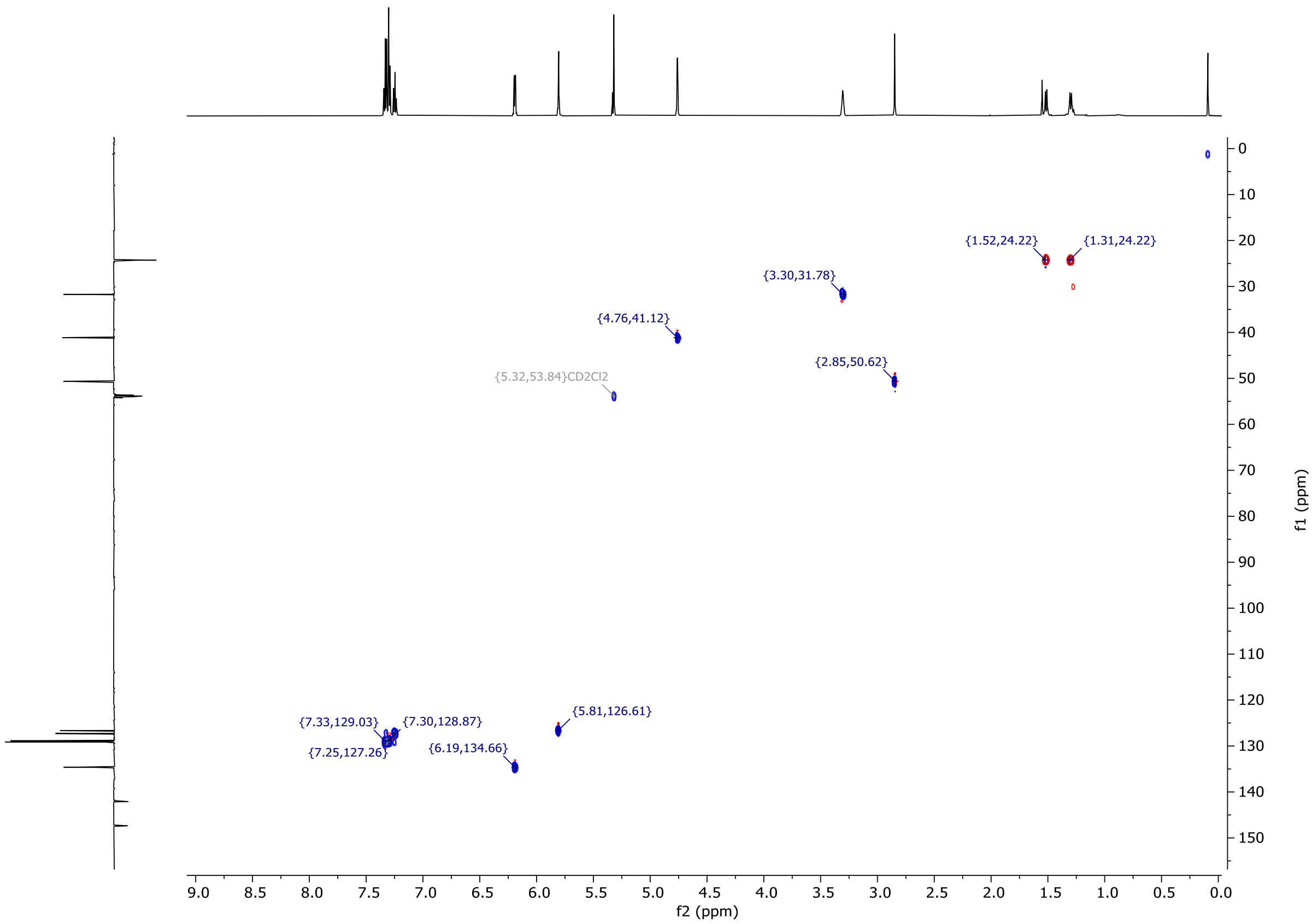

$^{1}H,^{13}C$ HSQC NMR spectrum (600 MHz/151 MHz, CD_2Cl_2) of '*bottom*'-*endo*-**6e**.

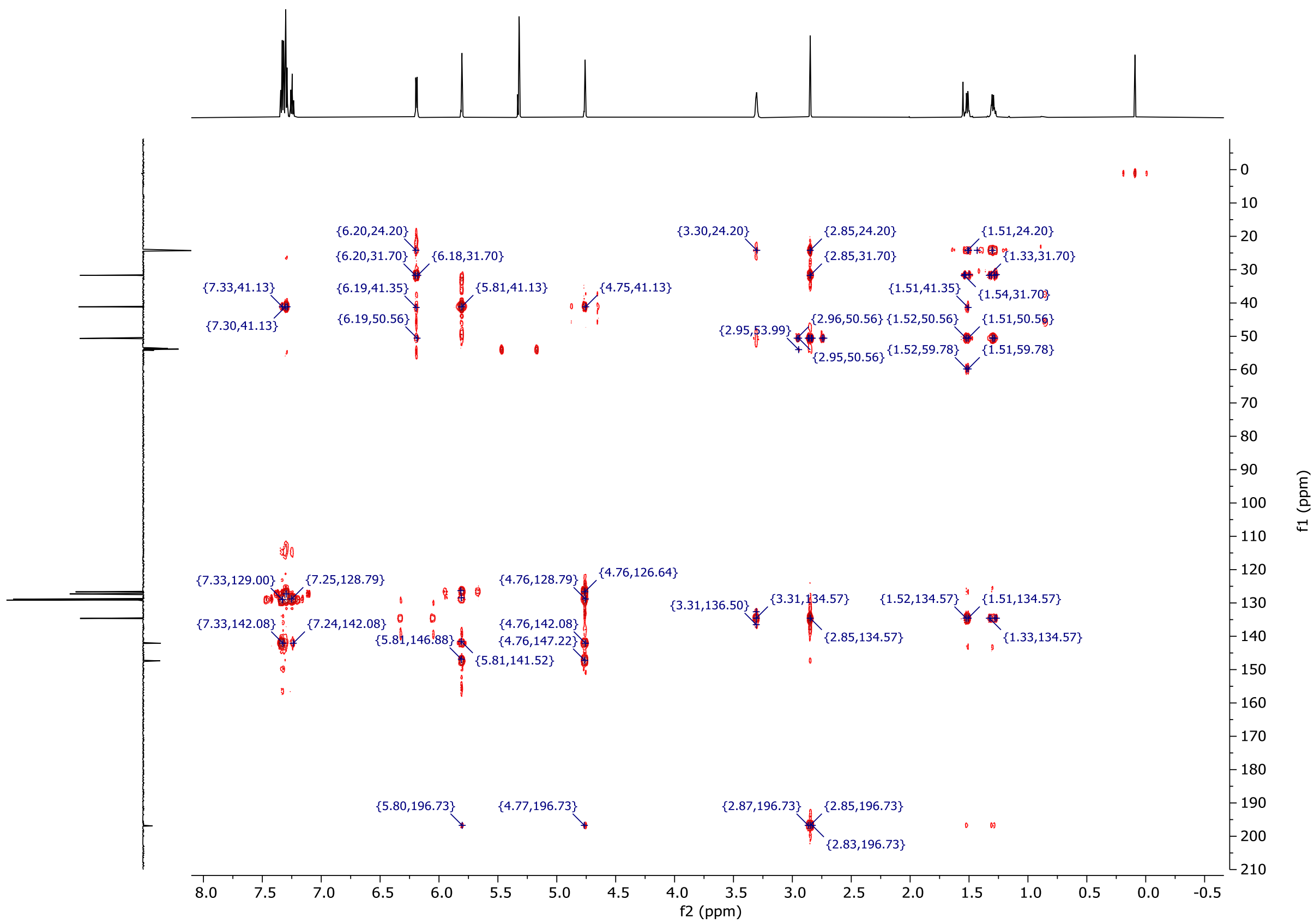

$^{1}H,^{13}C$ HMBC NMR spectrum (600MHz/151 MHz, CD_2Cl_2) of '*bottom*'-*endo*-**6e**.

- 1,4,4a,5,8,9a-hexahydro-1,4-ethano-5,8-diphenylanthracene-9,10-dione (‘*top’*-*endo*-**6e**)

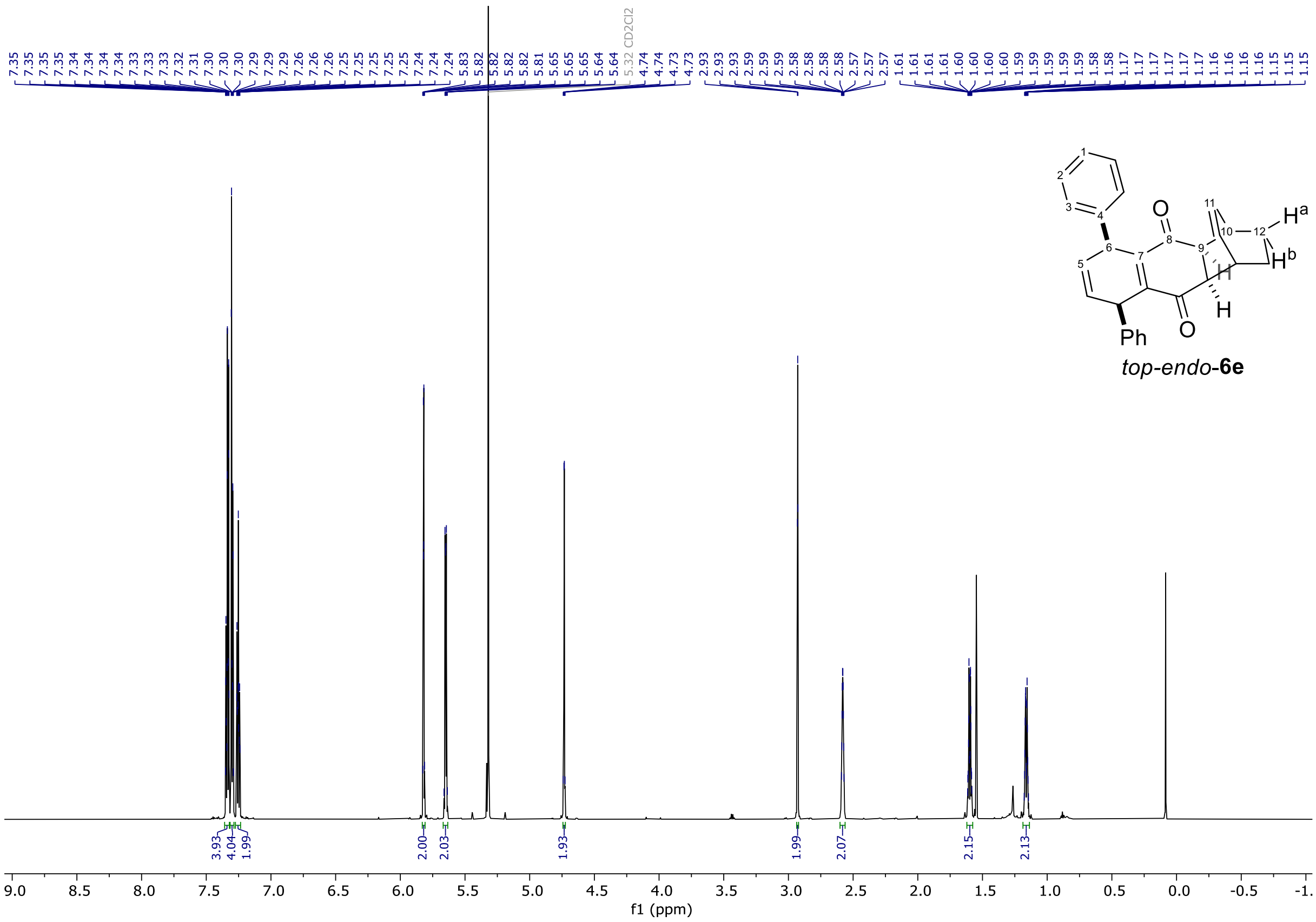

^{1}H NMR spectrum (700 MHz, CD_2Cl_2) of ‘*top*’-*endo*-**6e**.

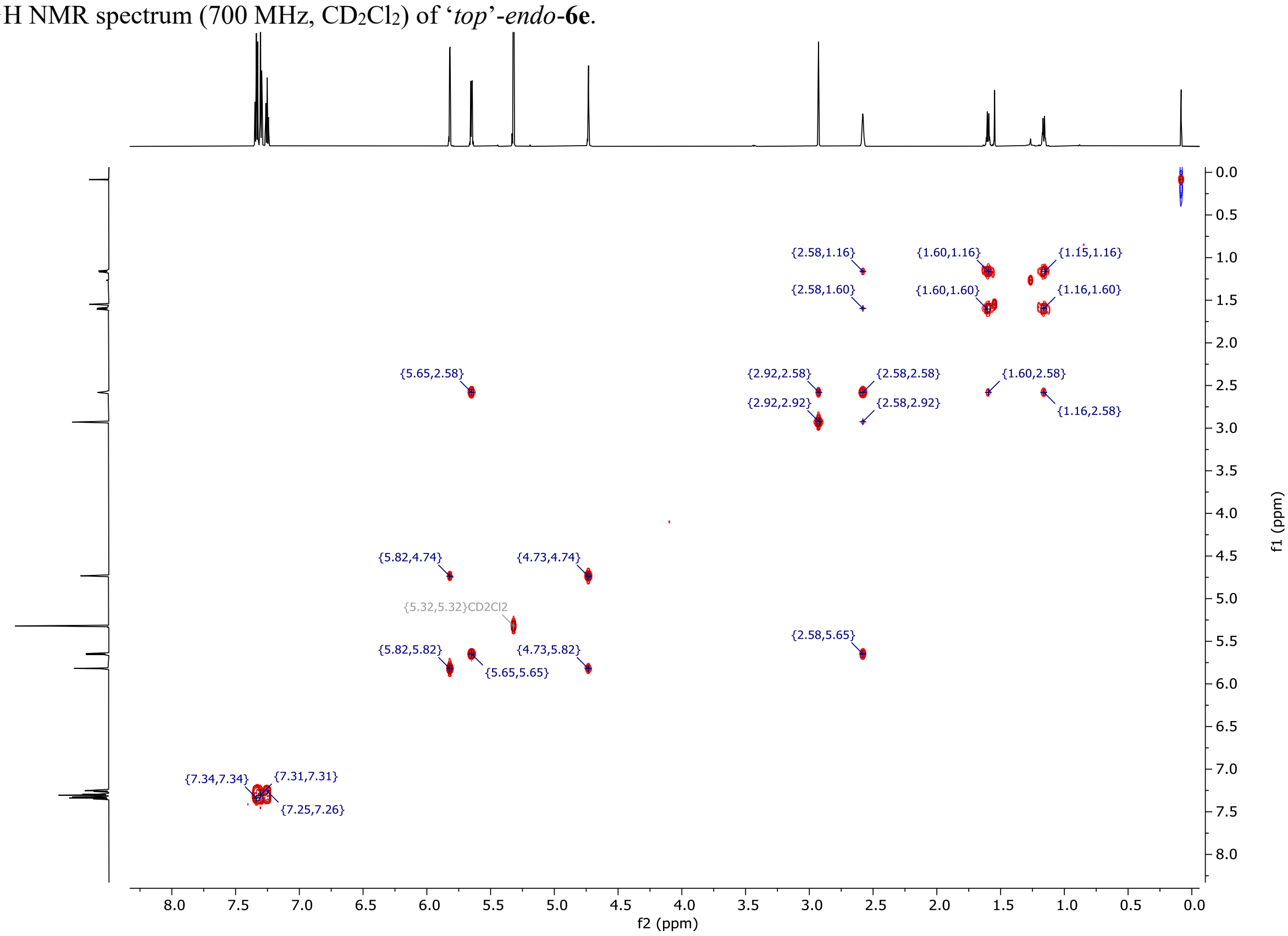

^{1}H,^{1}H COSY NMR spectrum (700 MHz, CD*2*Cl*2*) of ‘*top*’-*endo*-**6e**.

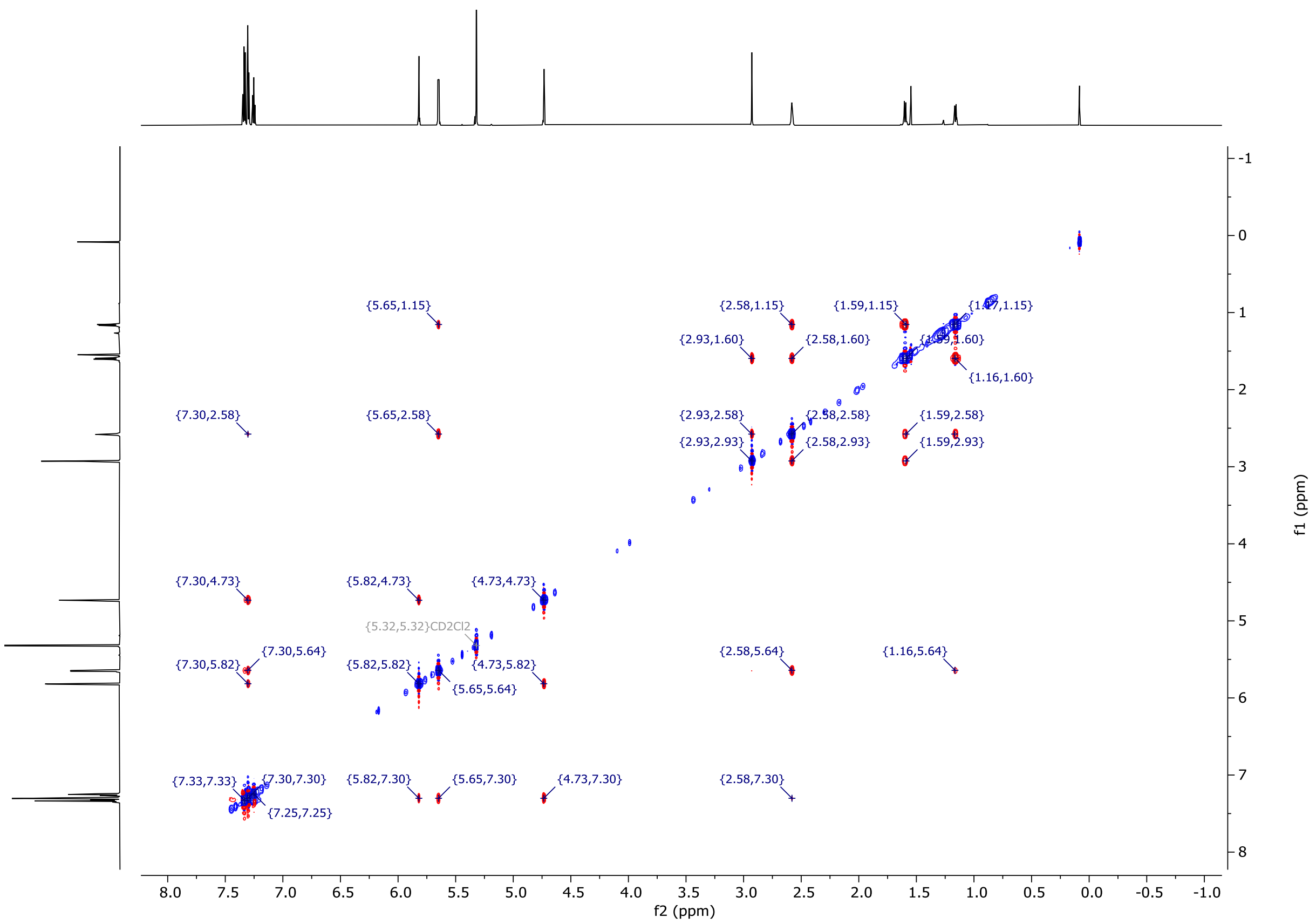

^{1}H,^{1}H NOESY NMR spectrum (700 MHz, CD_2Cl_2) of ‘*top*’-*endo*-**6e**

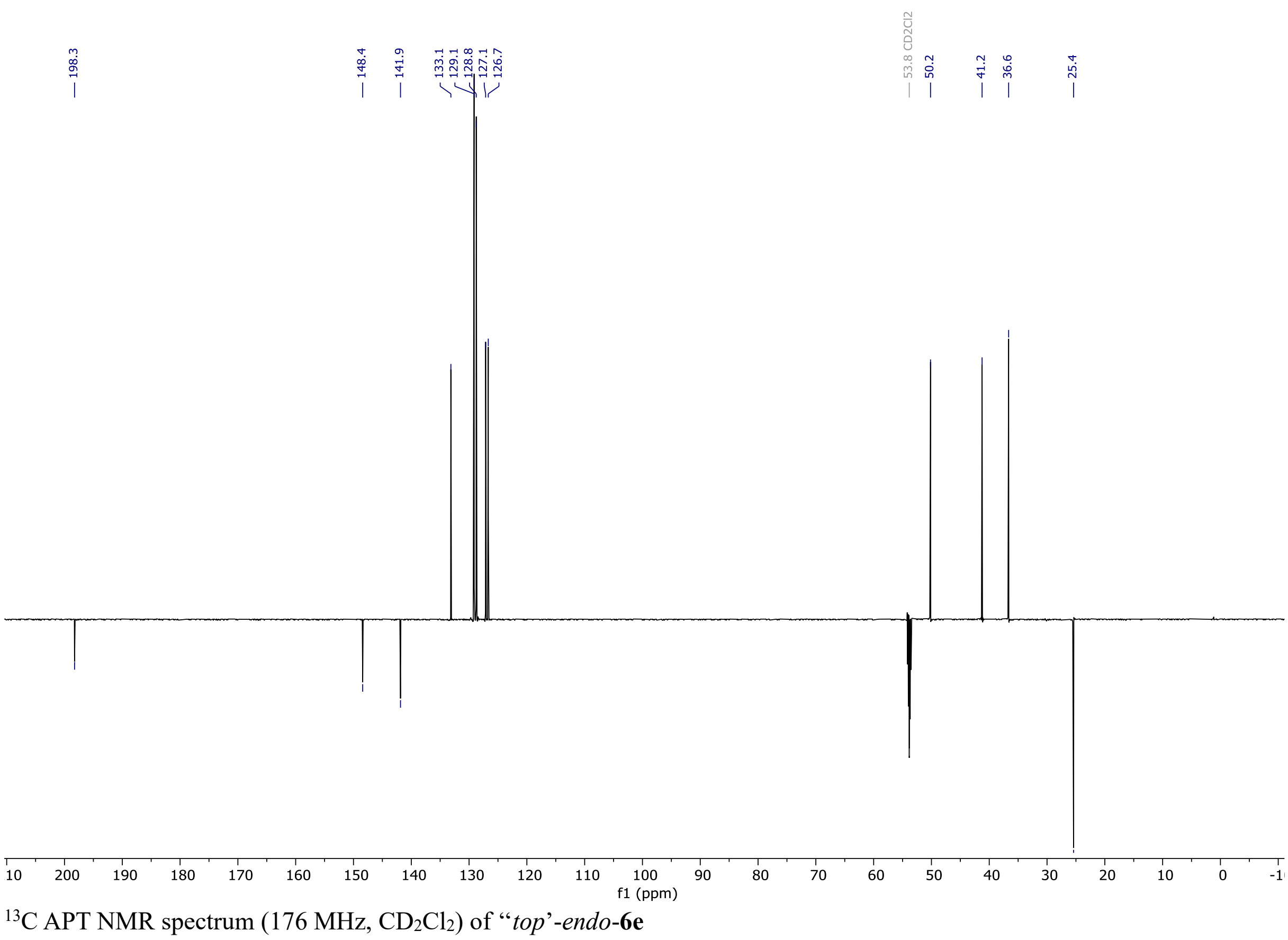

^{13}C APT NMR spectrum (176 MHz, CD_2Cl_2) of “*top*’-*endo*-**6e**

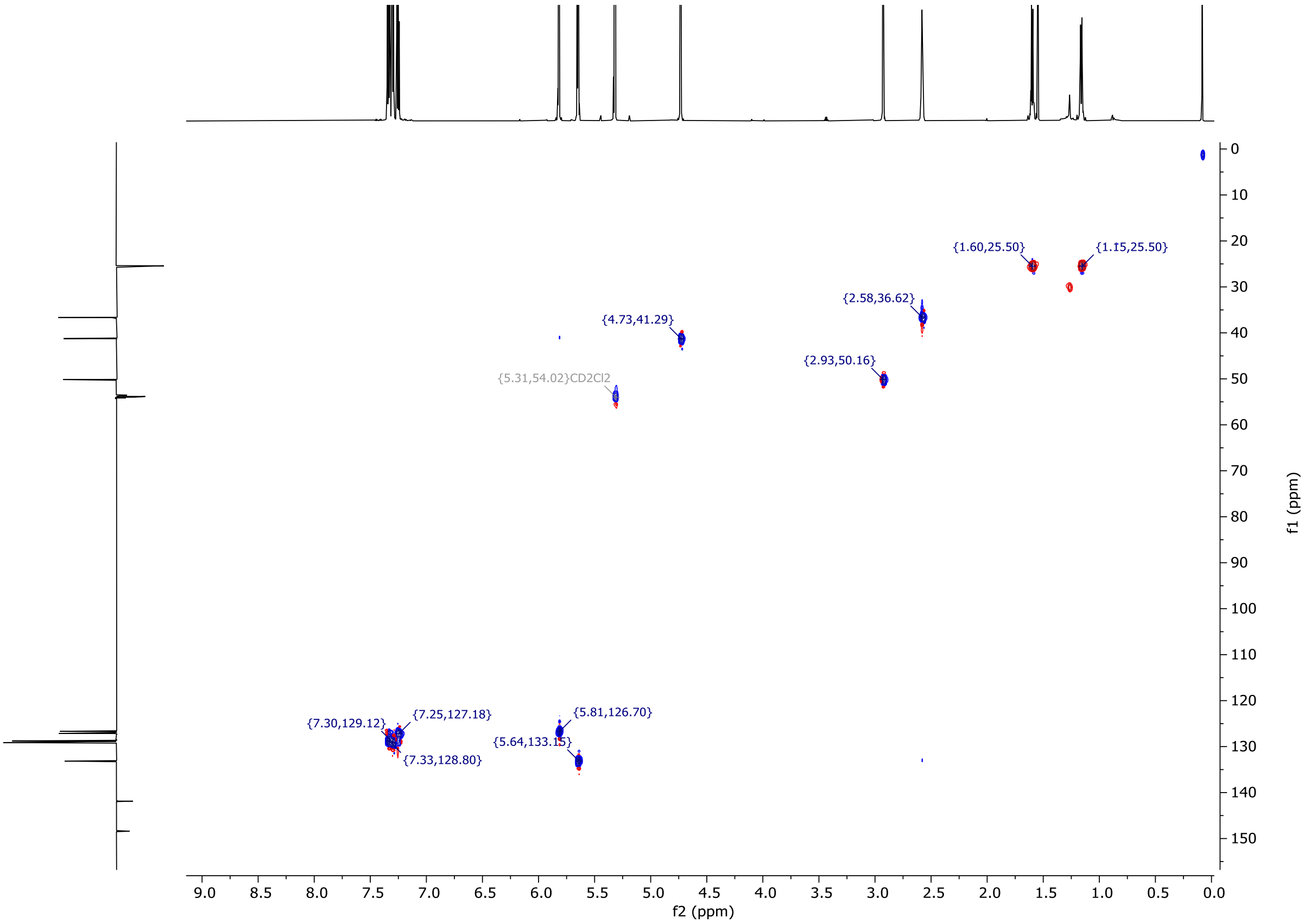

$^{1}H,^{13}C$ HSQC NMR spectrum (700 MHz/176 MHz, CD_2Cl_2) of ‘*top*’-*endo*-**6e**.

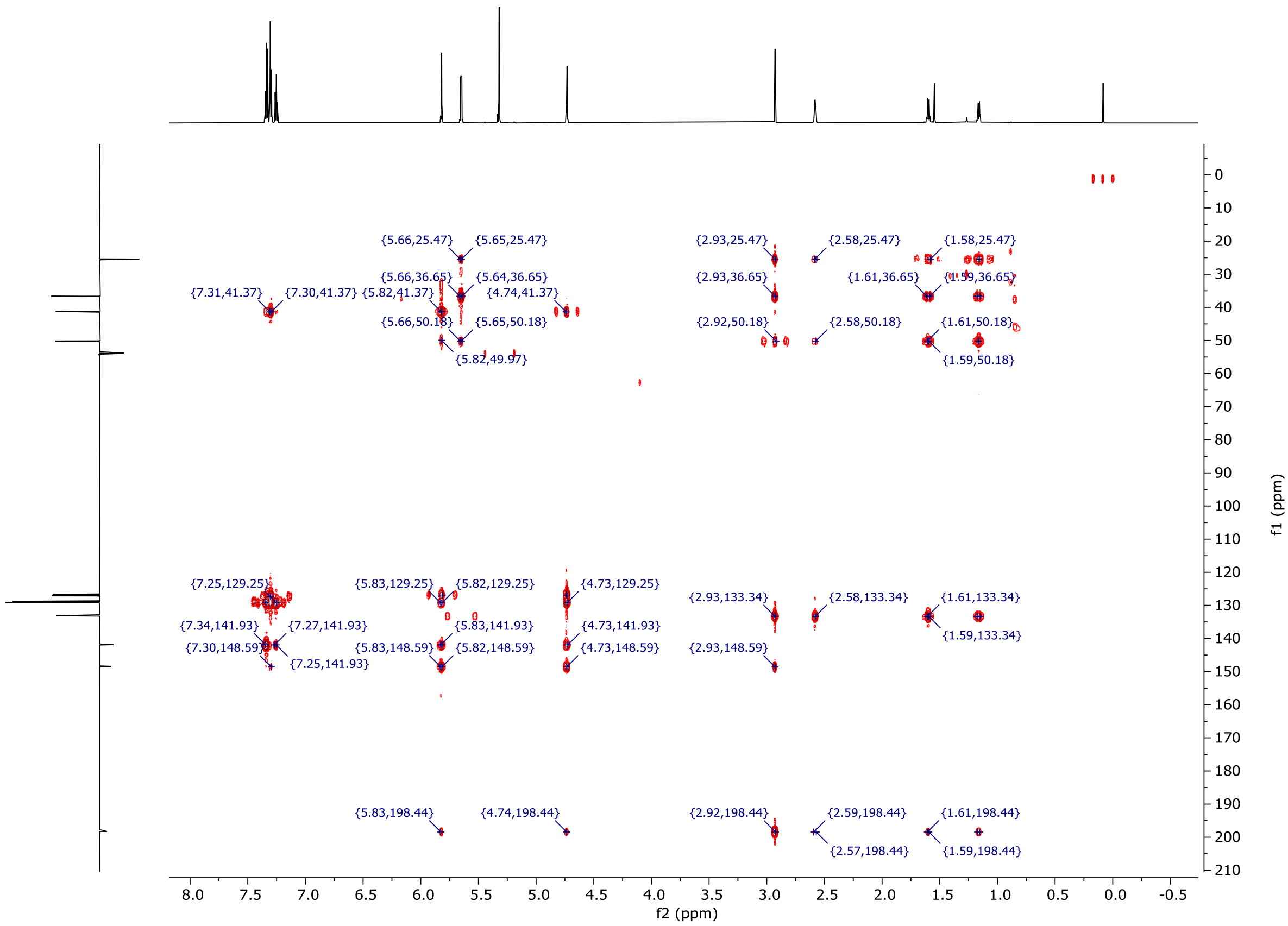

$^{1}H,^{13}C$ HMBC NMR spectrum (700 MHz/176 MHz, CD_2Cl_2) of ‘*top*’-*endo*-**6e**.

Pentacyclo[6.2.2.0^{2,7}.0^{4,10}.0^{5,9}]dodecane-3,6-dione (**7a**)

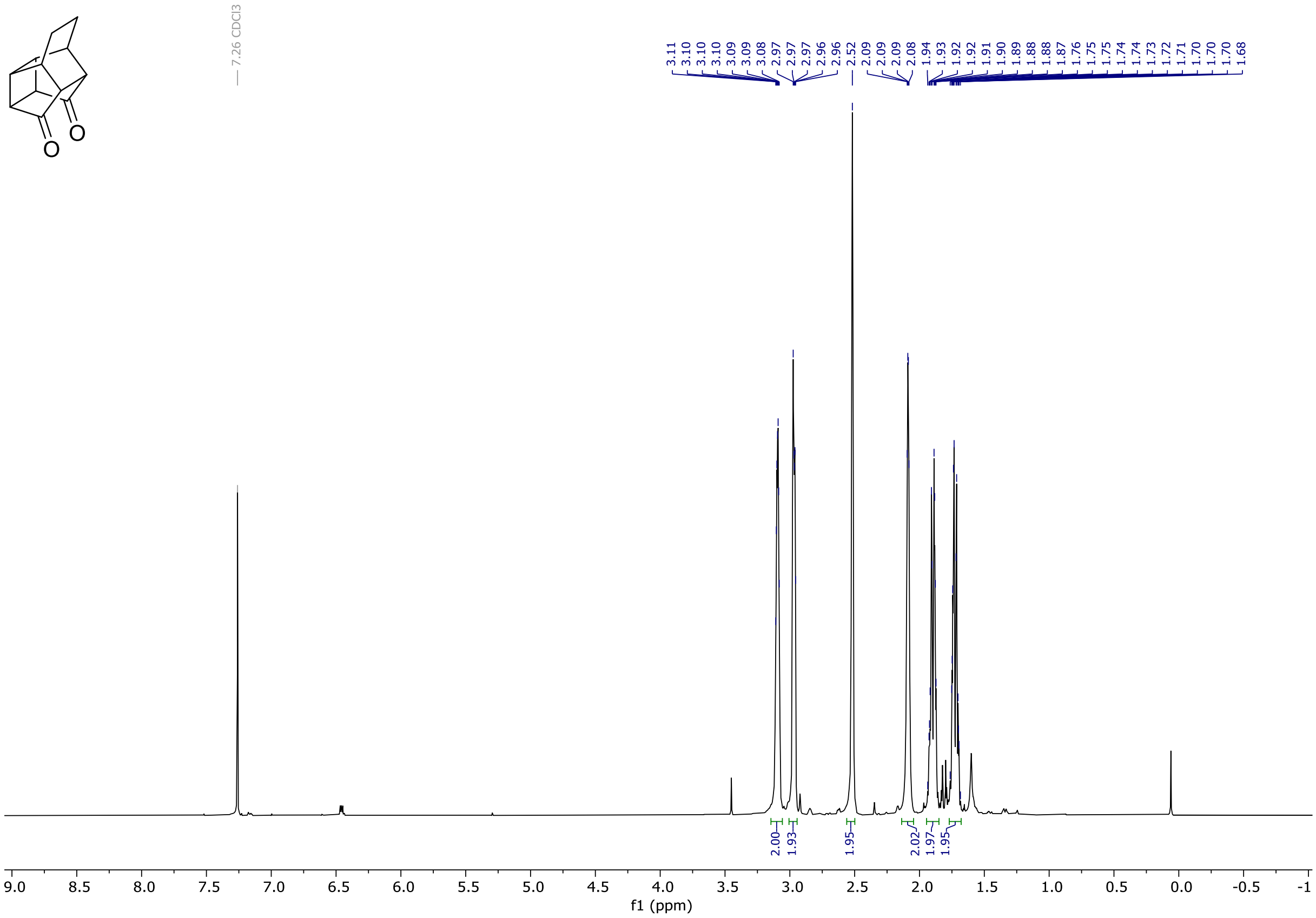

^{1}H NMR spectrum (400 MHz, $CDCl_3$) of **7a**.

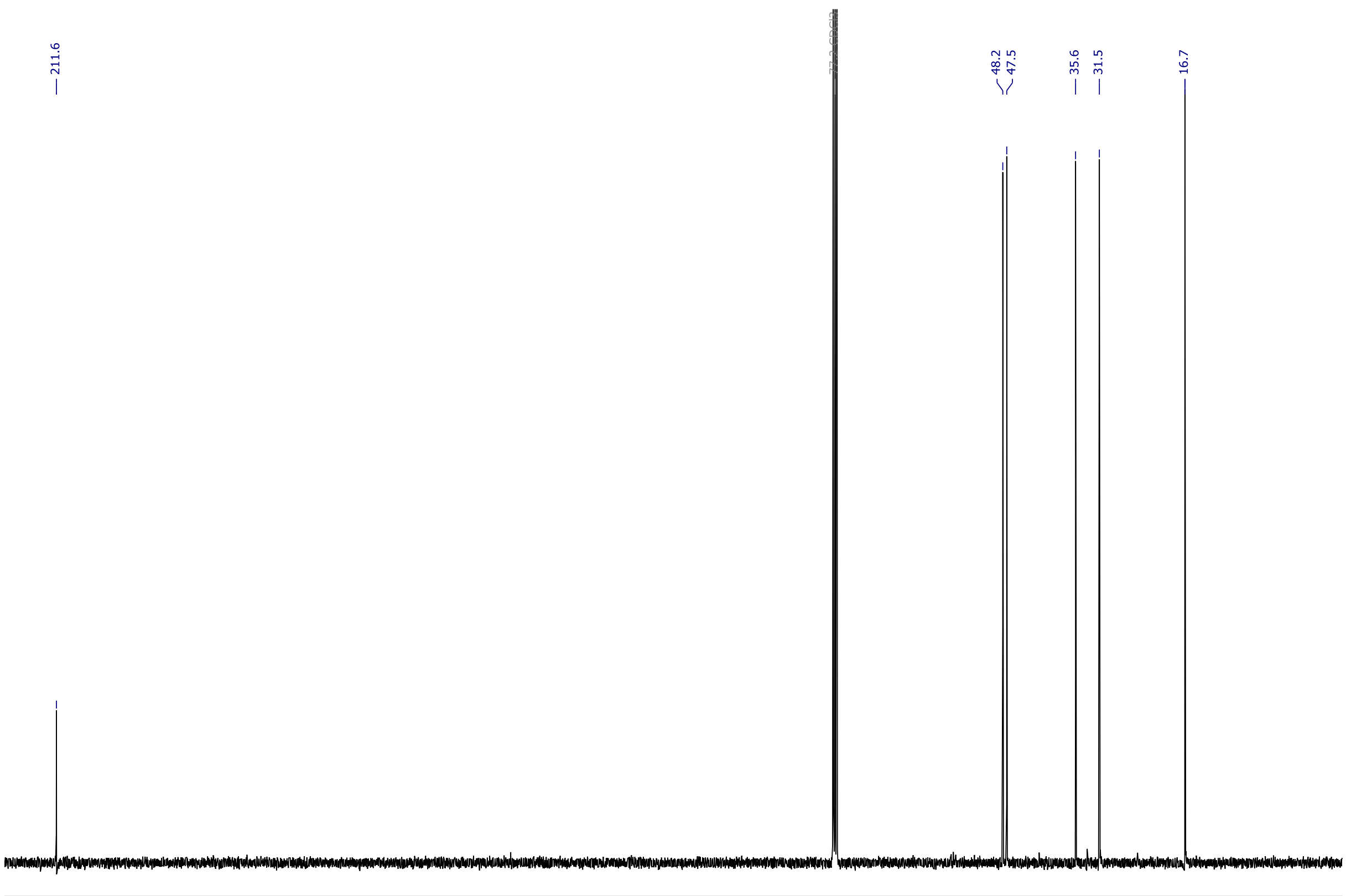

^{13}C NMR spectrum (101 MHz, $CDCl_3$) of **7a**.

2,5-Dimethylpentacyclo[6.2.2.0^{2,7}.0^{4,10}.0^{5,9}]dodecane-3,6-dione (**7b**)

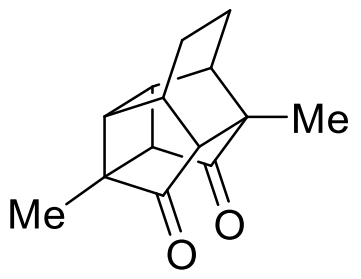

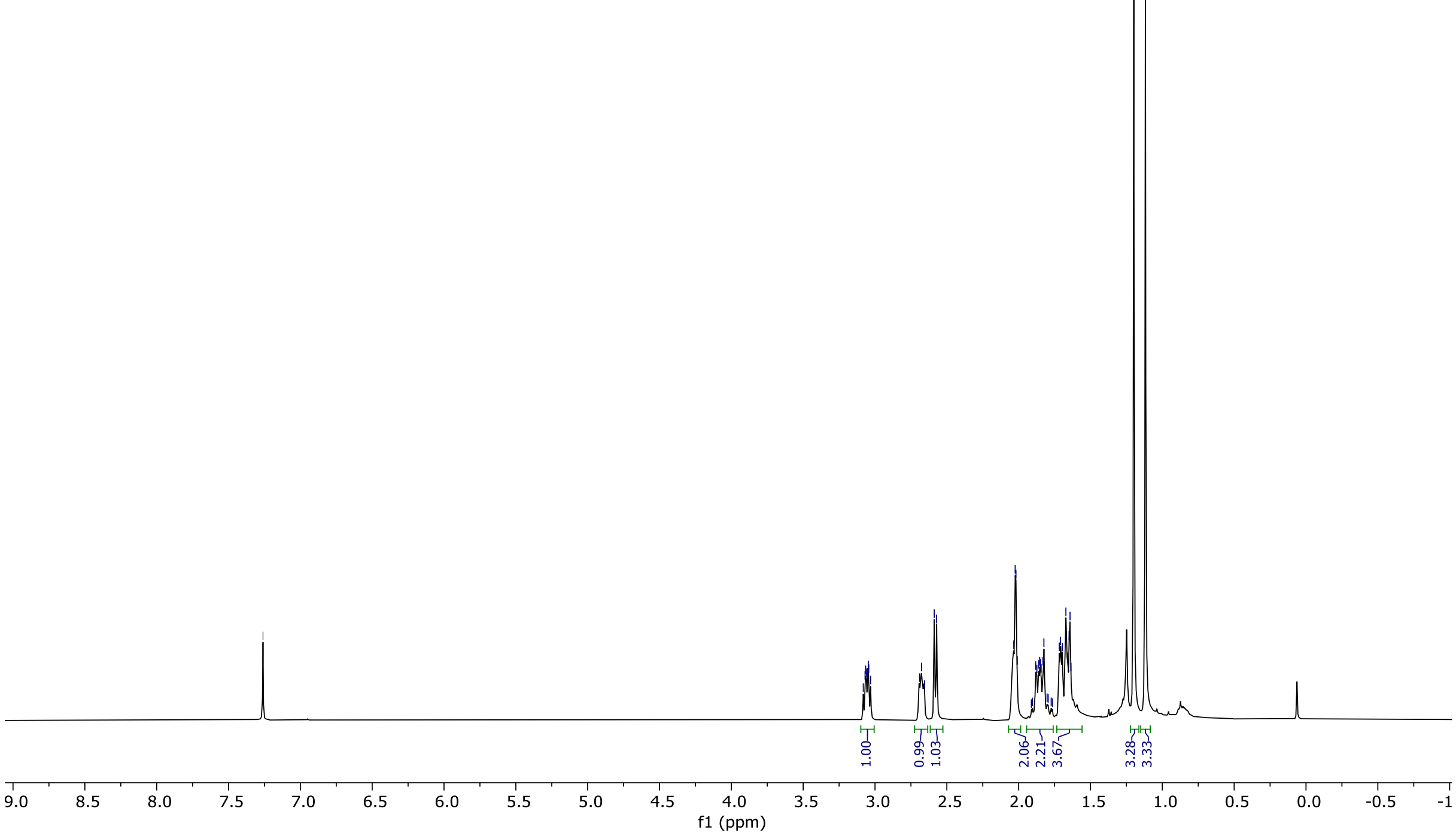

^{1}H NMR spectrum (400 MHz, CDCl$_3$) of **7b**.

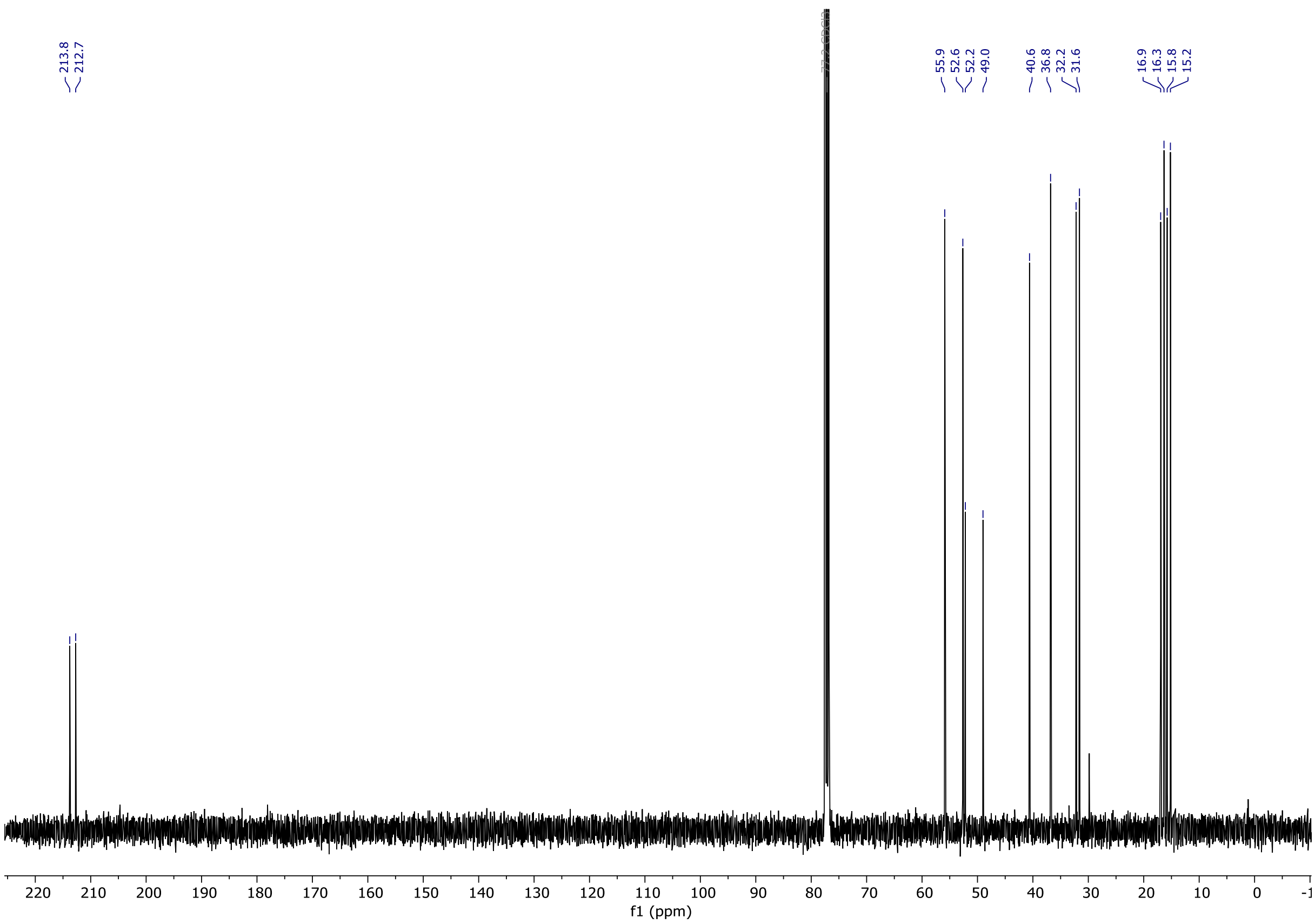

^{13}C NMR spectrum (101 MHz, CDCl$_3$) of **7b**.

5,8-Diphenylhexacyclo[10.2.2.0^{2,11}.0^{4,9}.0^{4,14}.0^{9,13}]hexadec-6,7-ene-3,10-dione (**7c**)

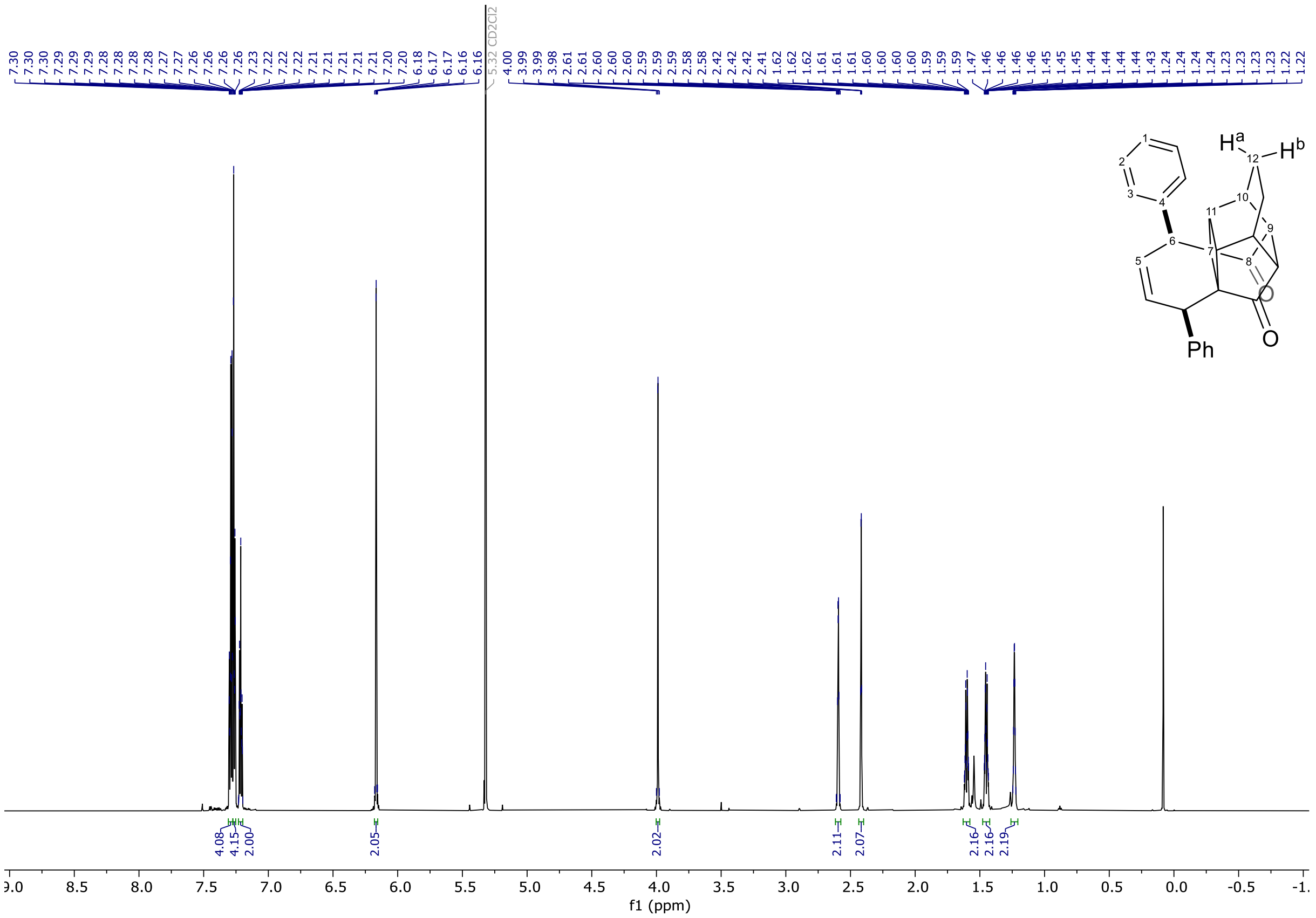

^{1}H NMR spectrum (700 MHz, $CDCl_3$) of **7c**.

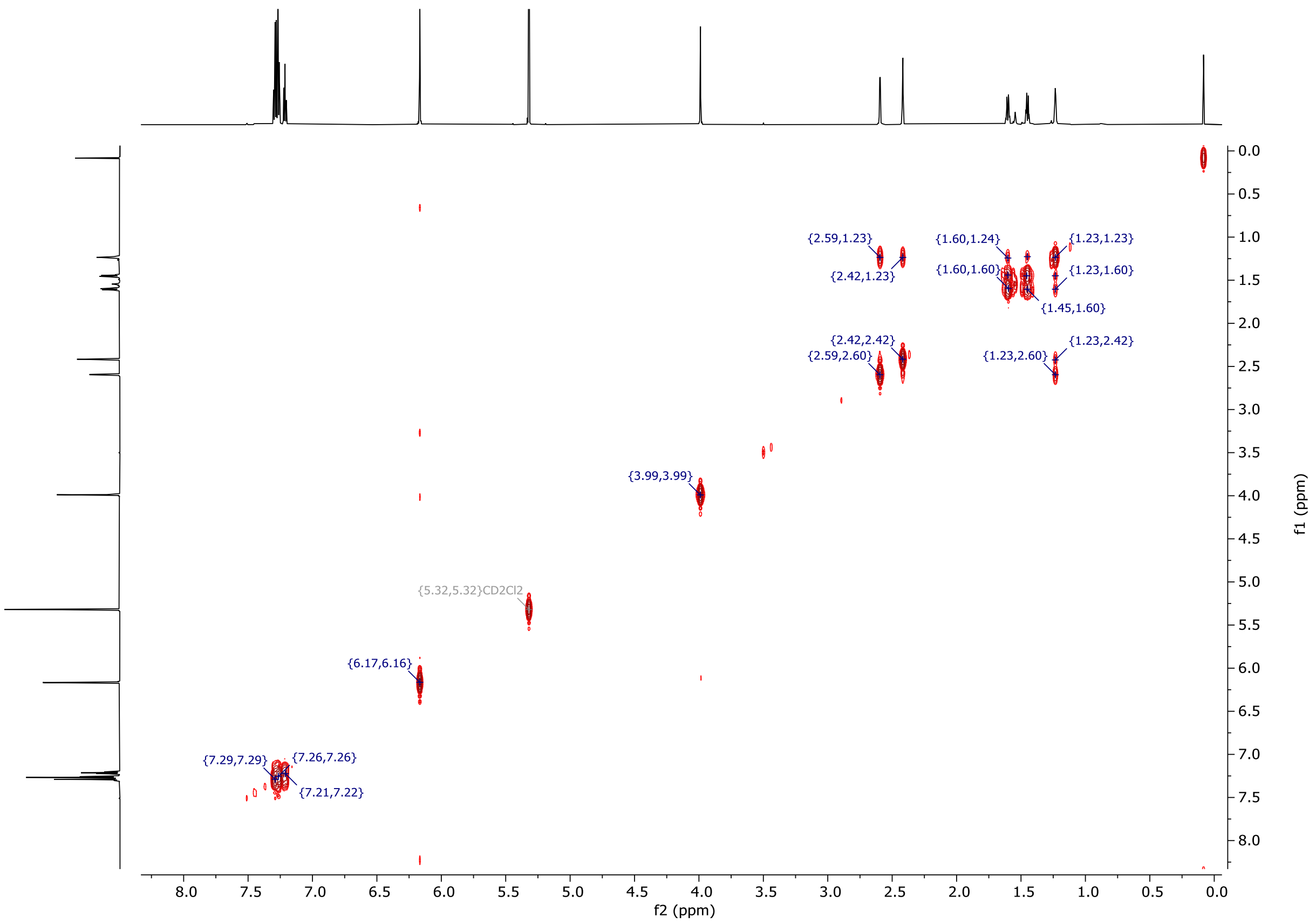

^{1}H,^{1}H COSY NMR spectrum (700 MHz, CD_2Cl_2) of **7c**.

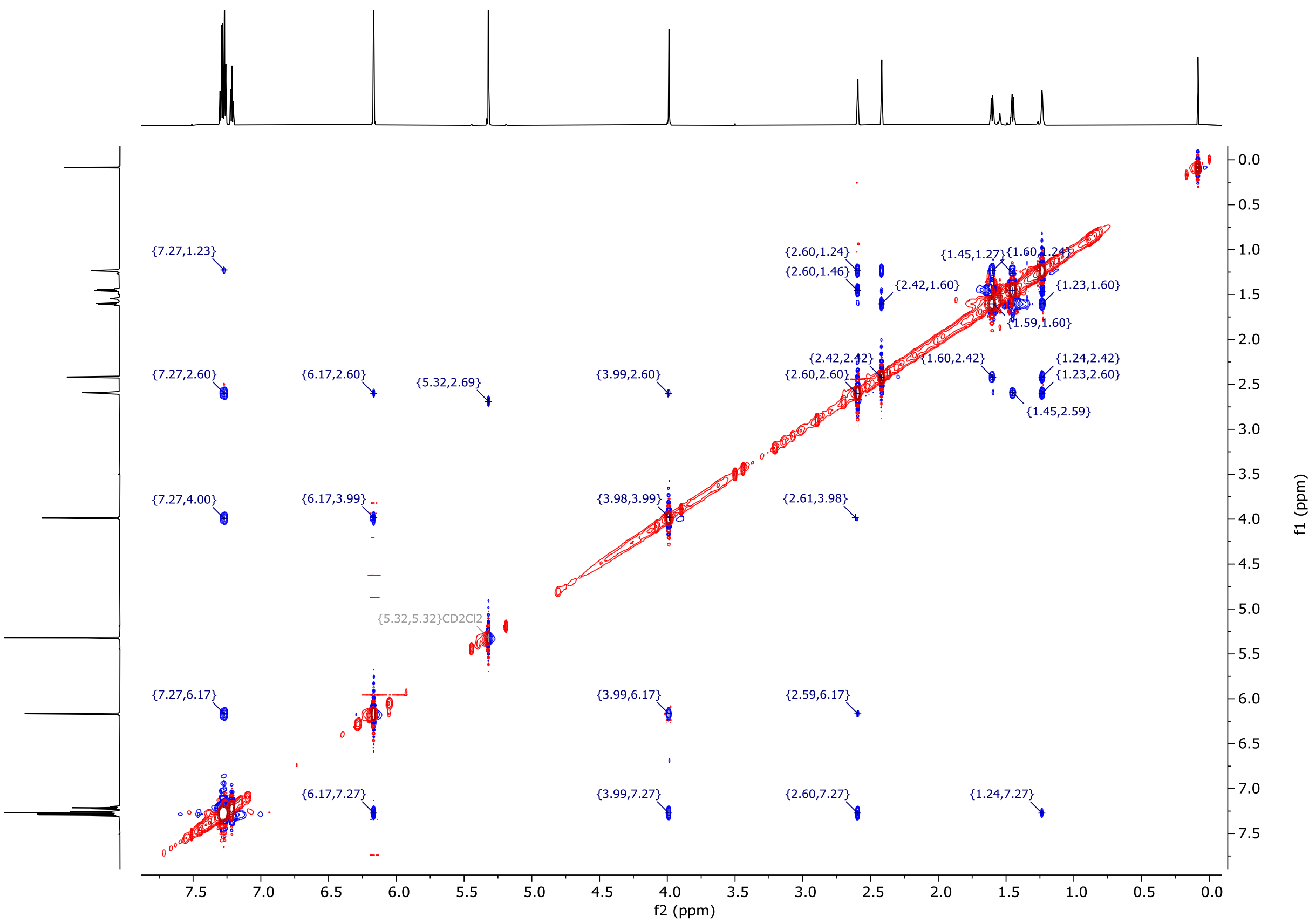

$^{1}H,^{1}H$ NOESY NMR spectrum (700 MHz, CD_2Cl_2) of **7c**.

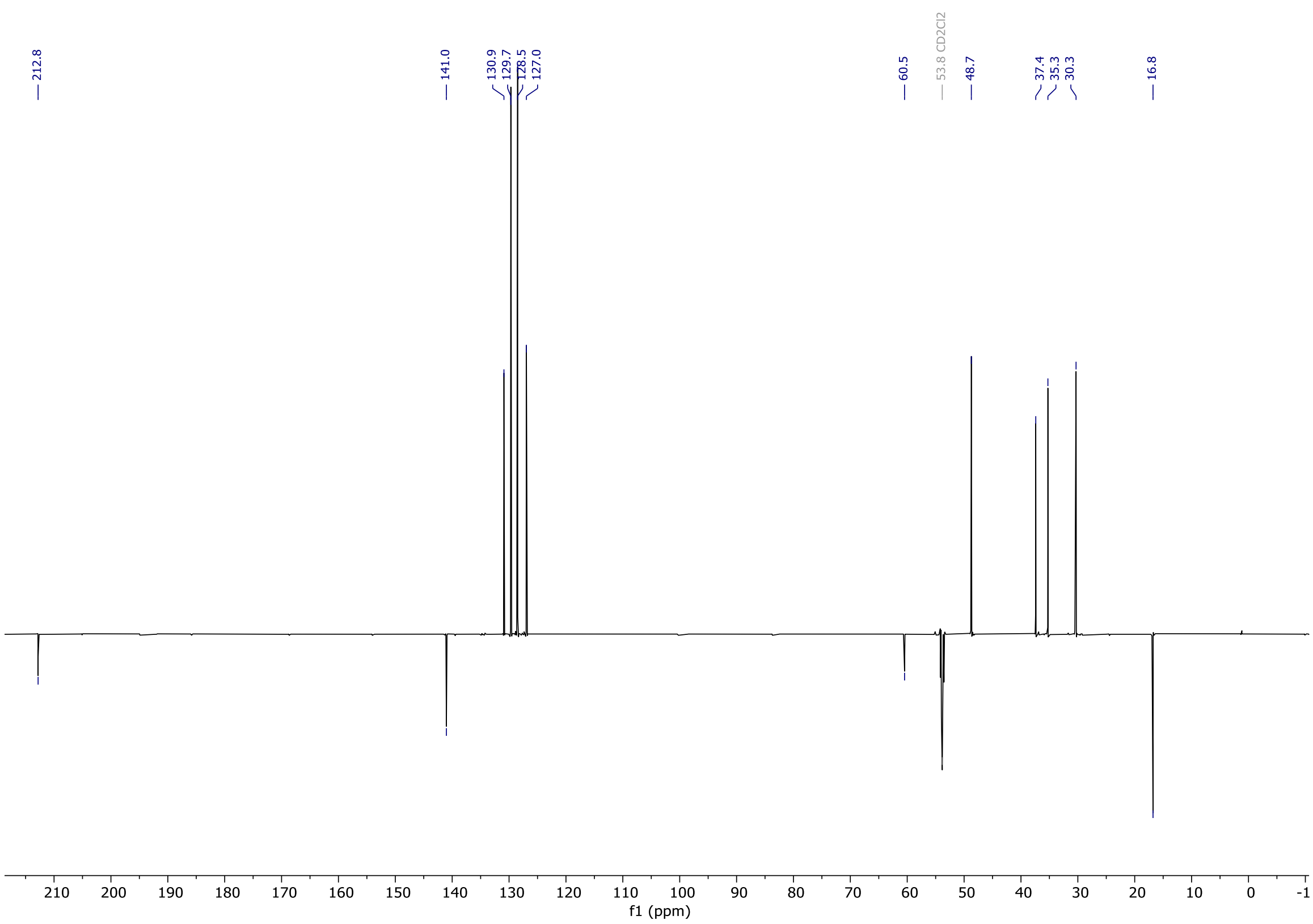

^{13}C APT NMR spectrum (176 MHz, $CDCl_3$) of **7c**.

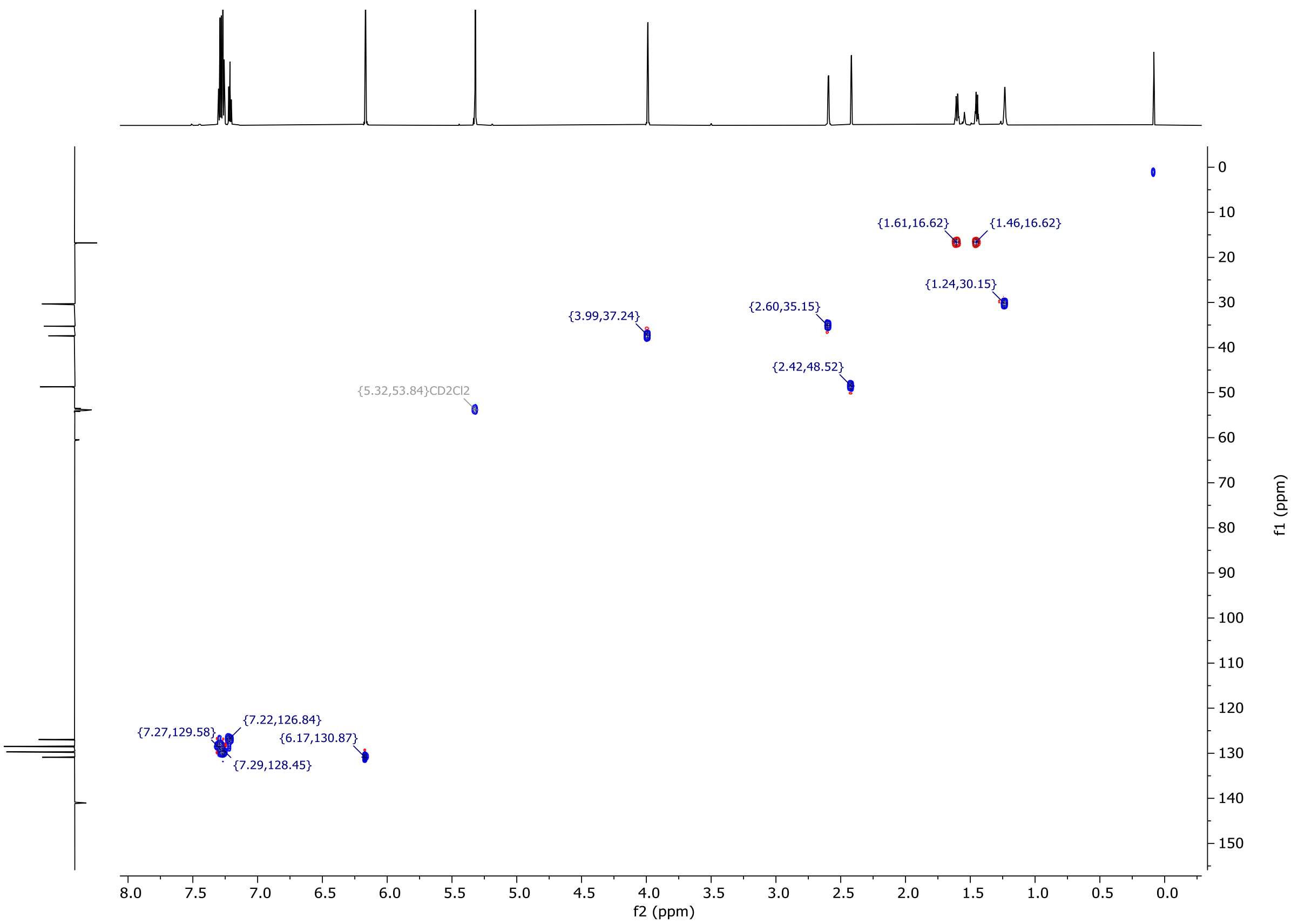

$^1H^{13}C$ HSQC APT NMR spectrum (700MHz/176 MHz, CD_2Cl_2) of **7c**.

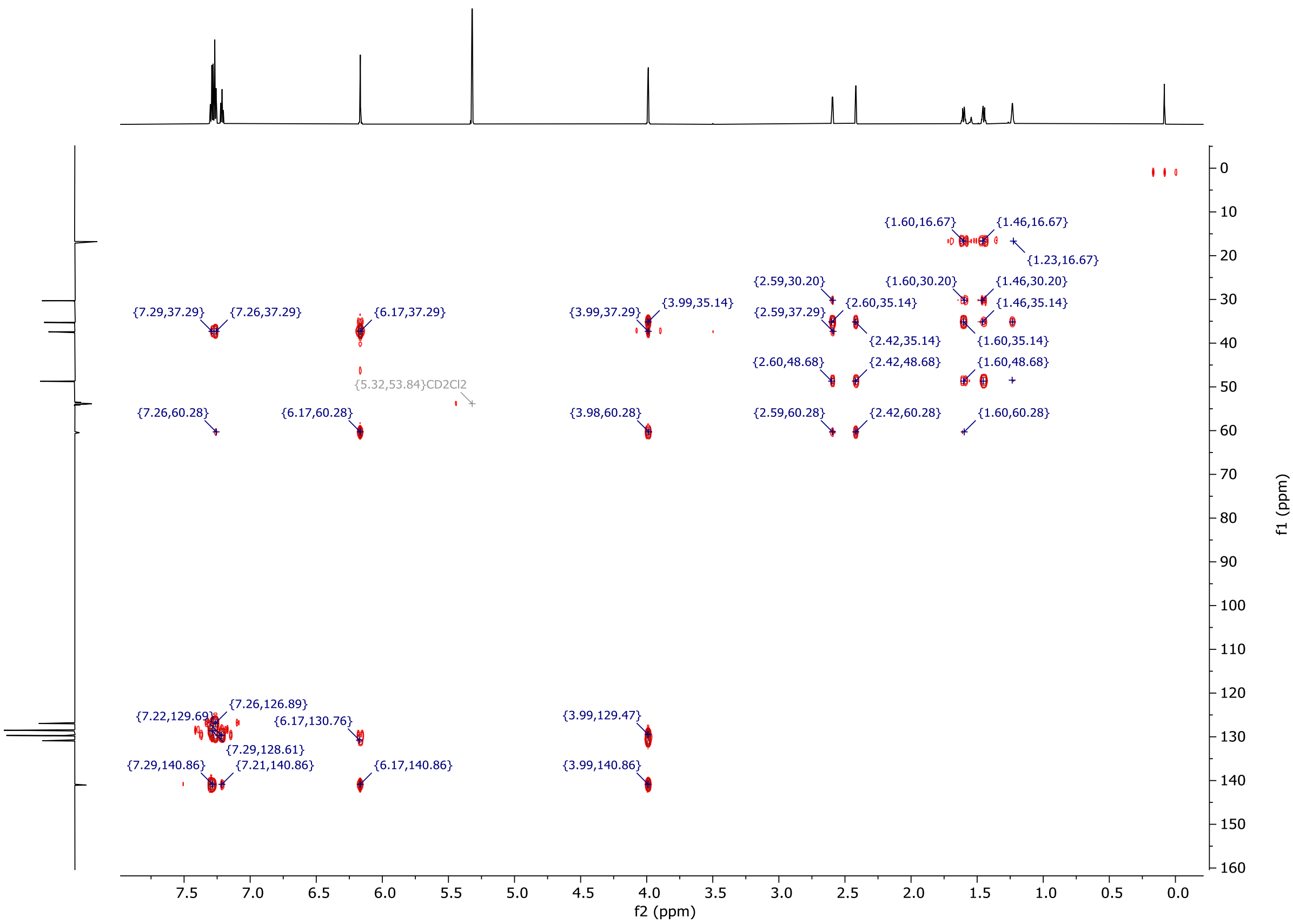

$^1H^{13}C$ HMBC NMR spectrum (700MHz/176 MHz, CD_2Cl_2) of **7c**.

1,4,5,8-Tetrahydro-1,4,5,8-tetraphenylanthracene-9,10-dione (**8a**)

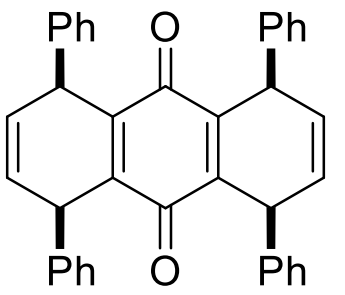

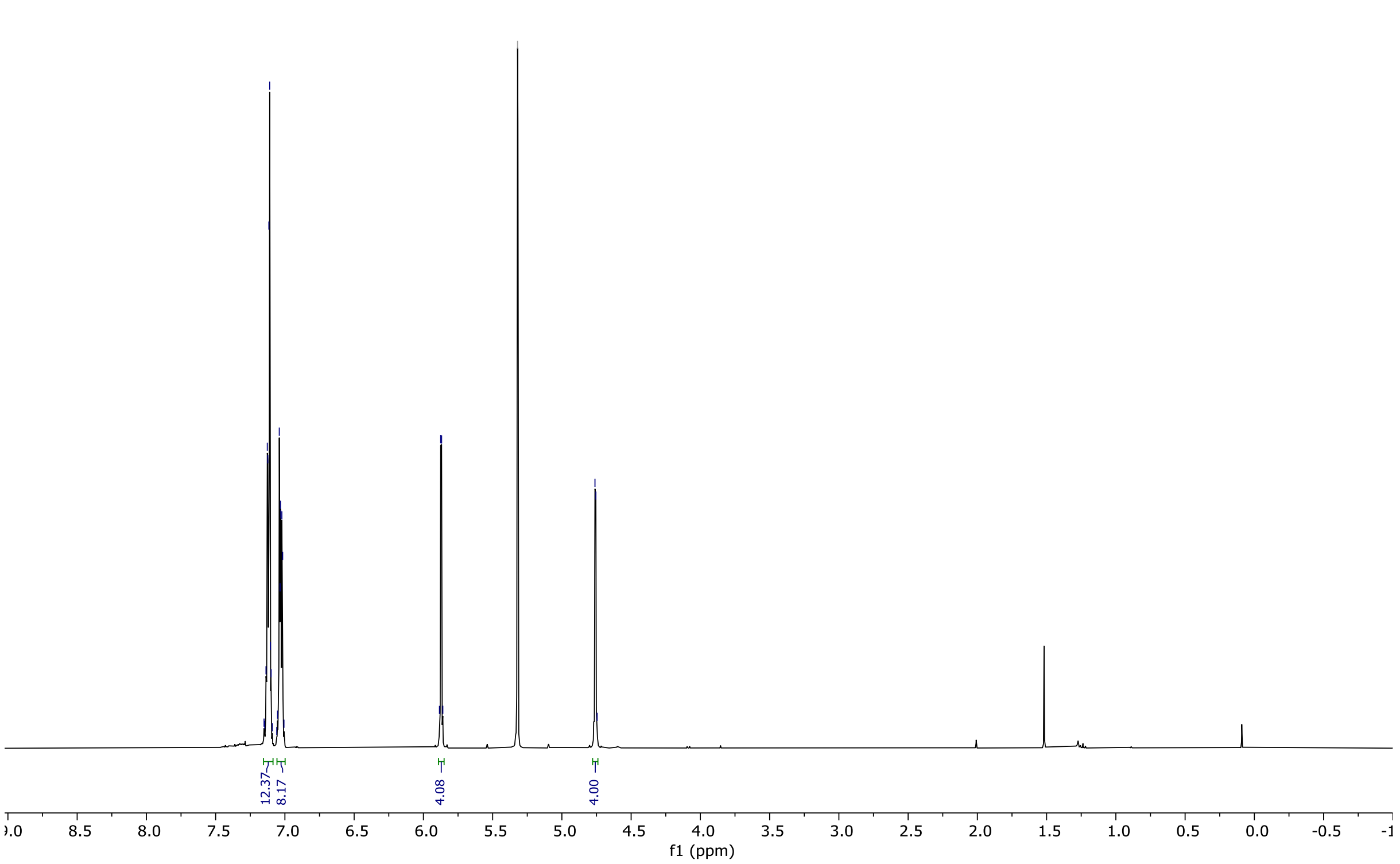

^{1}H NMR spectrum (400 MHz, CD_2Cl_2) of **8a**.

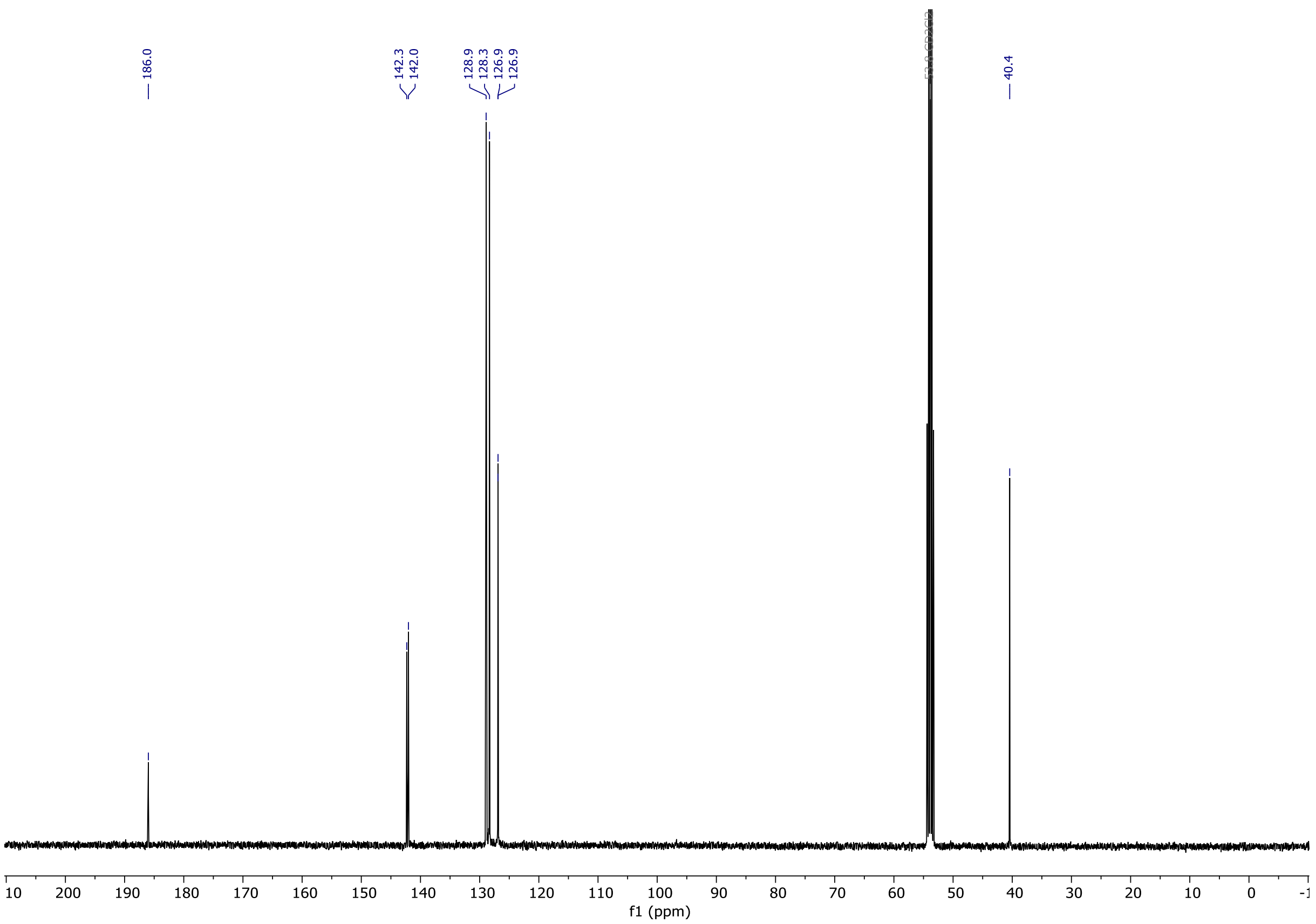

^{13}C NMR spectrum (101 MHz, CD_2Cl_2) of **8a**.

1,4,5,8-Tetrahydro-1,4-di(4-methylphenyl)-5,8-diphenylanthracene-9,10-dione (**8b**)

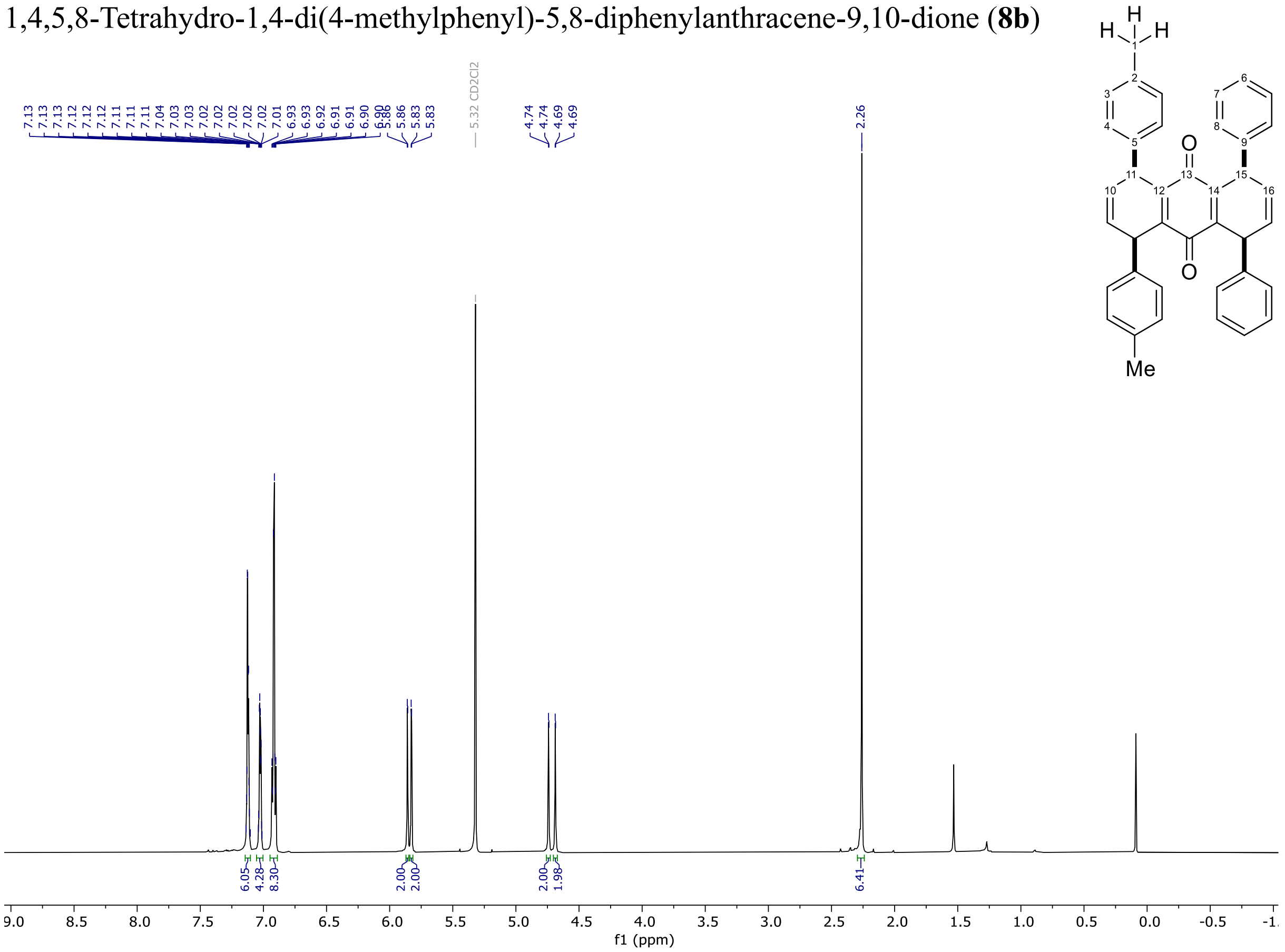

^{1}H NMR spectrum (700 MHz, CD_2Cl_2) of **8b**.

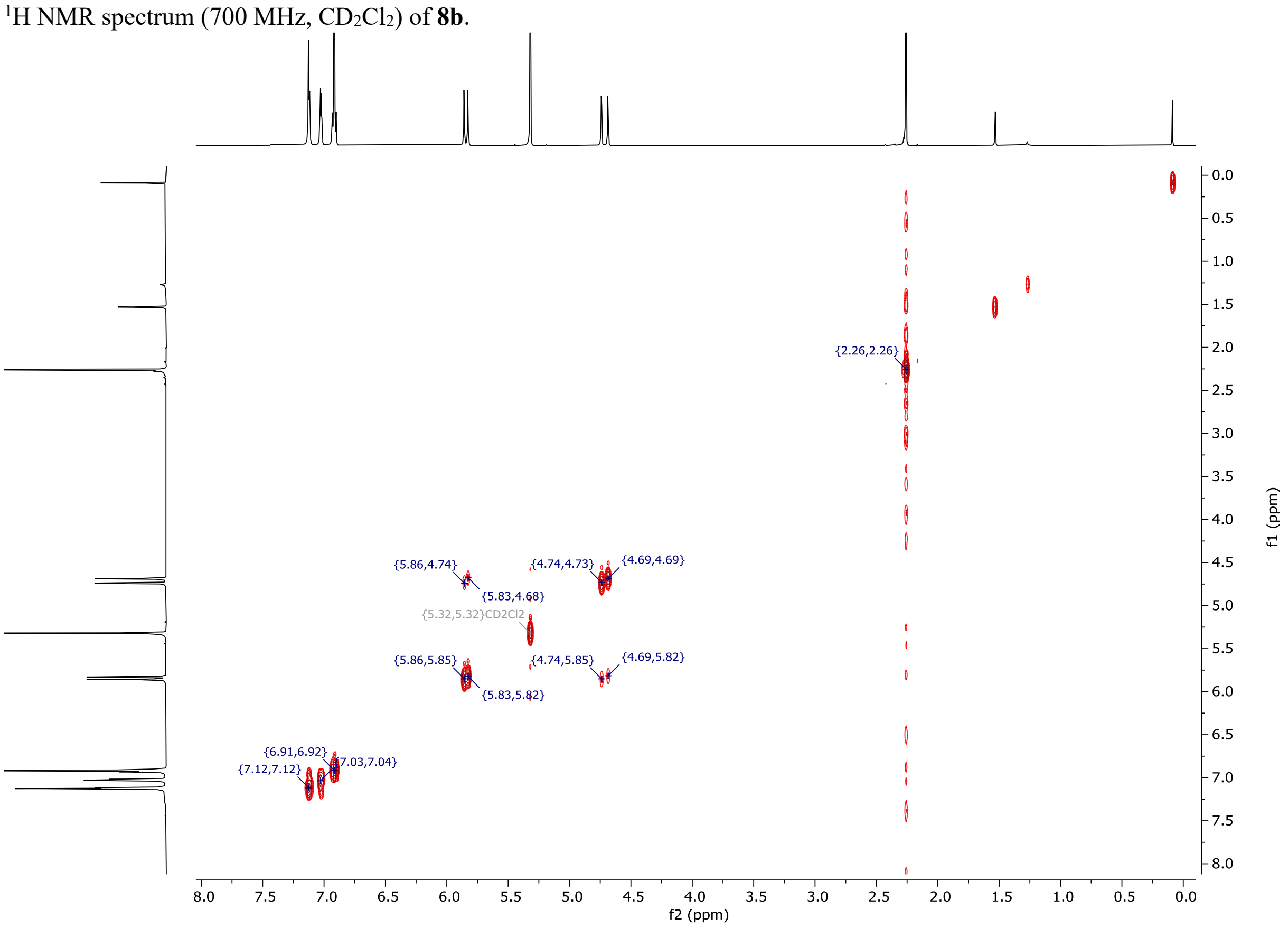

^{1}H,1H COSY NMR spectrum (700 MHz, CD_2Cl_2) of **8b**.

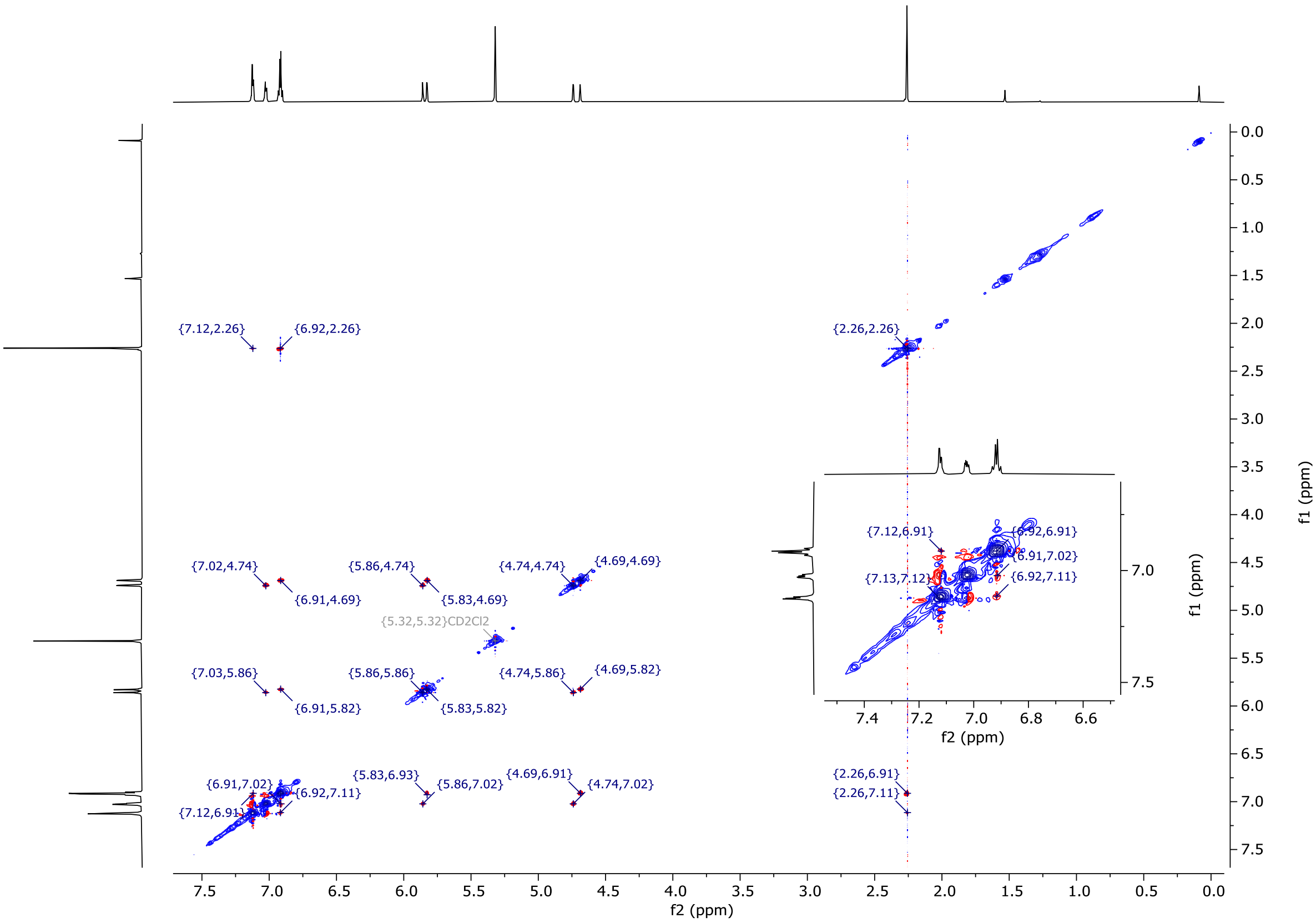

^{1}H,^{1}H NOESY NMR spectrum (700 MHz, CD_2Cl_2) of **8b**.

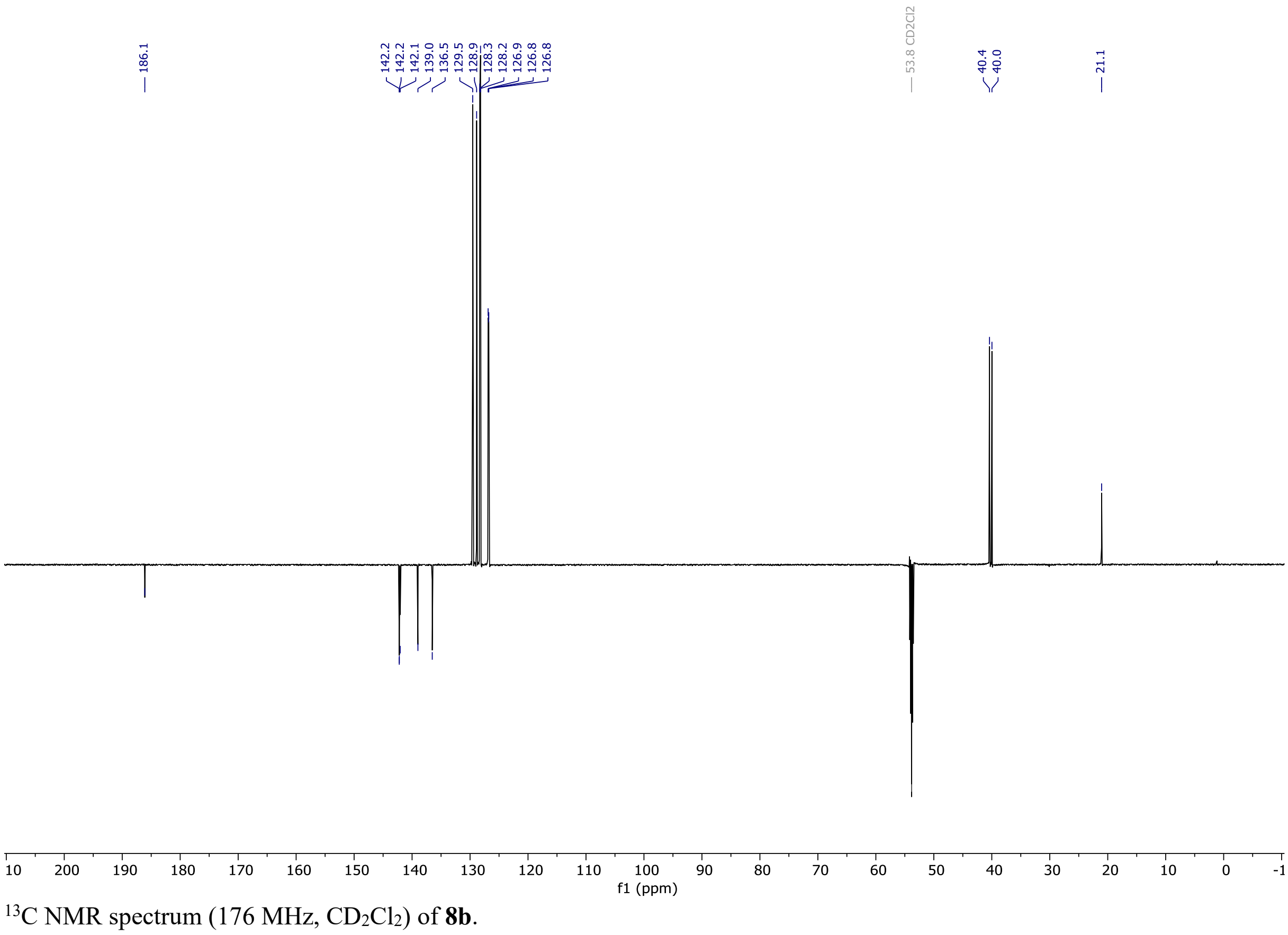

^{13}C NMR spectrum (176 MHz, CD_2Cl_2) of **8b**.

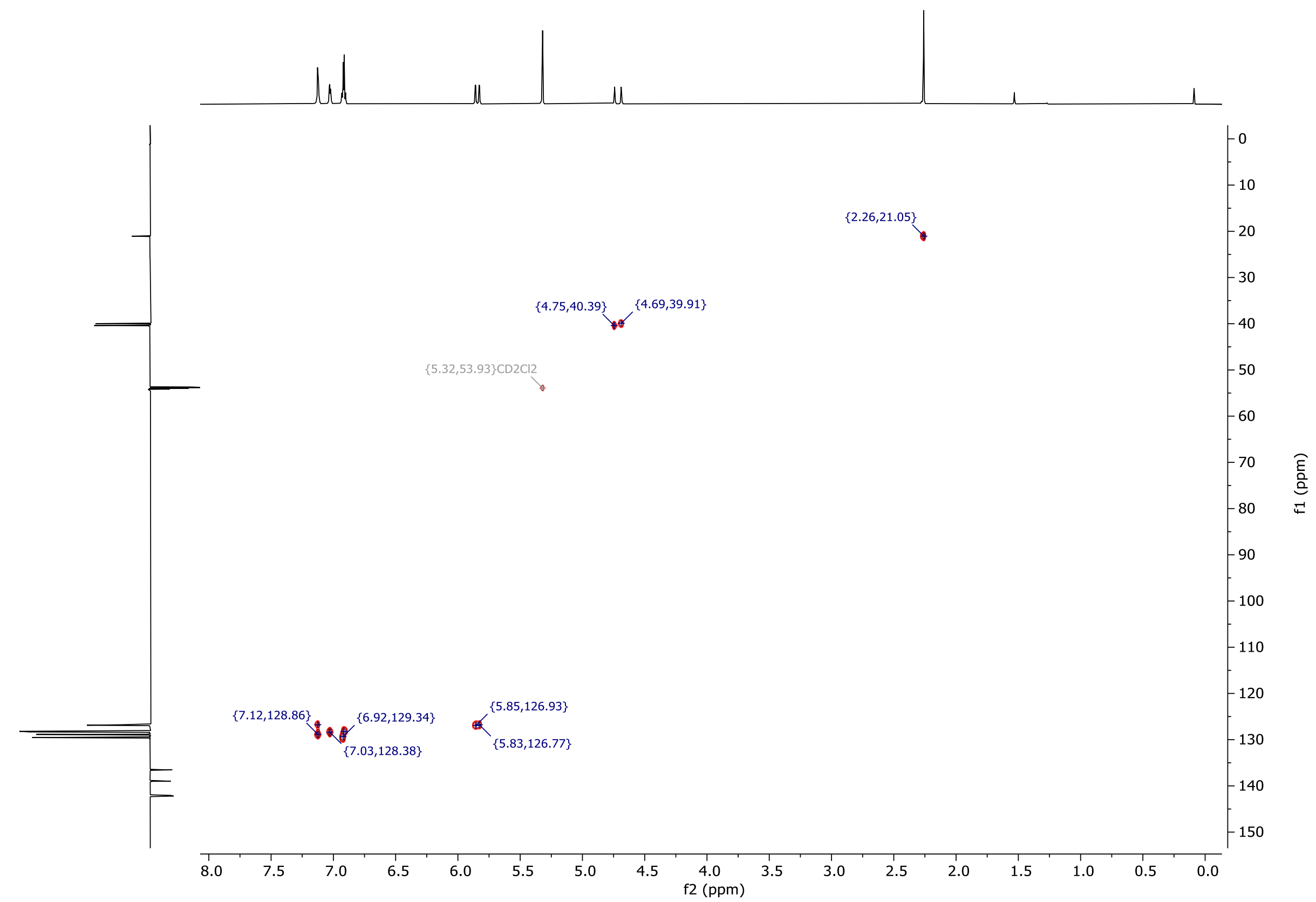

$^{1}H,^{13}C$ HSQC NMR spectrum (700 MHz/176 MHz, CD_2Cl_2) of **8b**.

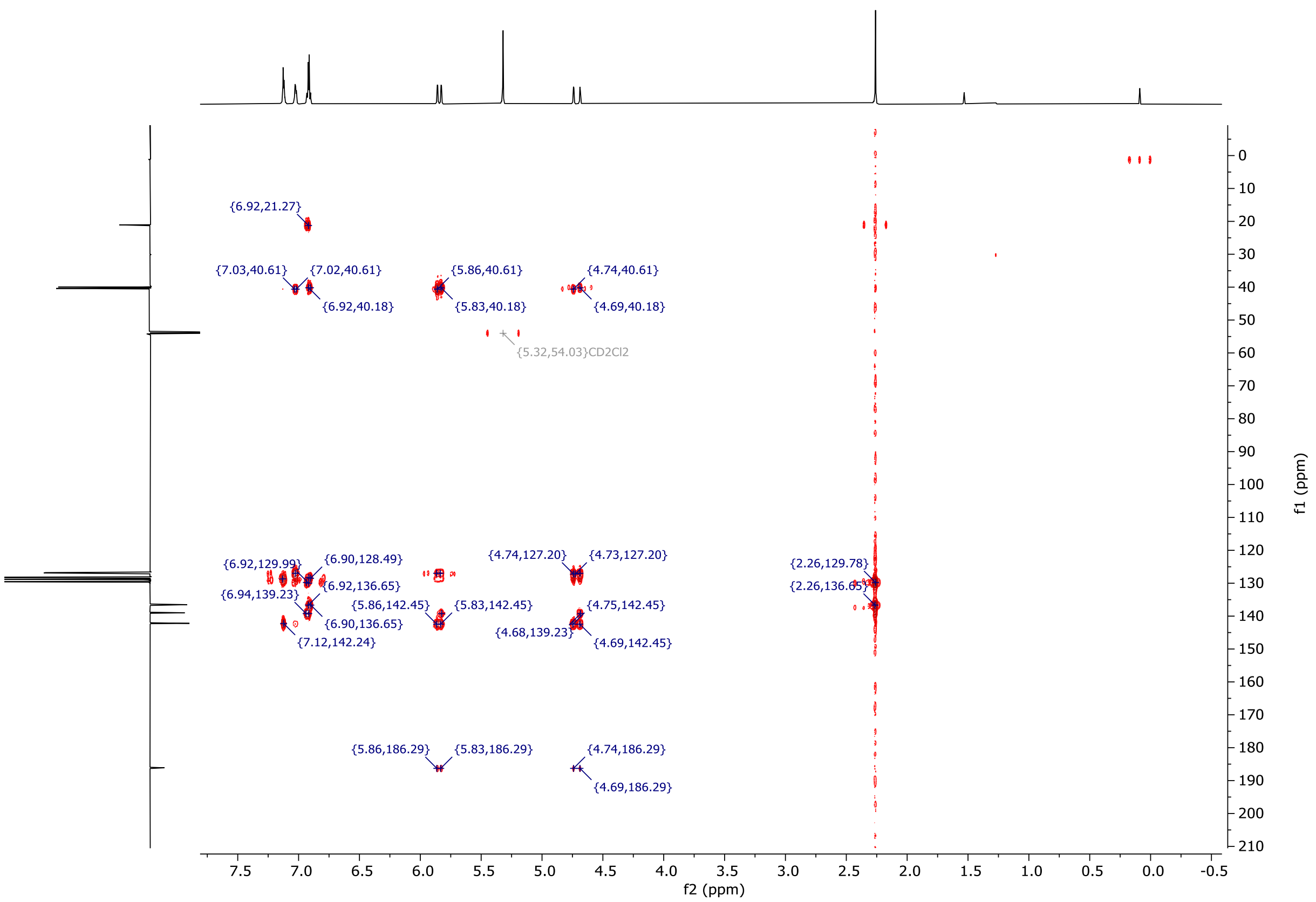

$^{1}H,^{13}C$ HMBC NMR spectrum (700 MHz/176 MHz, CD_2Cl_2) of **8b**.

1,4,5,8-Tetrahydro-1,4-di(4-bromophenyl)-5,8-diphenyl-9,10-anthracene-9,10-dione (**8c**)

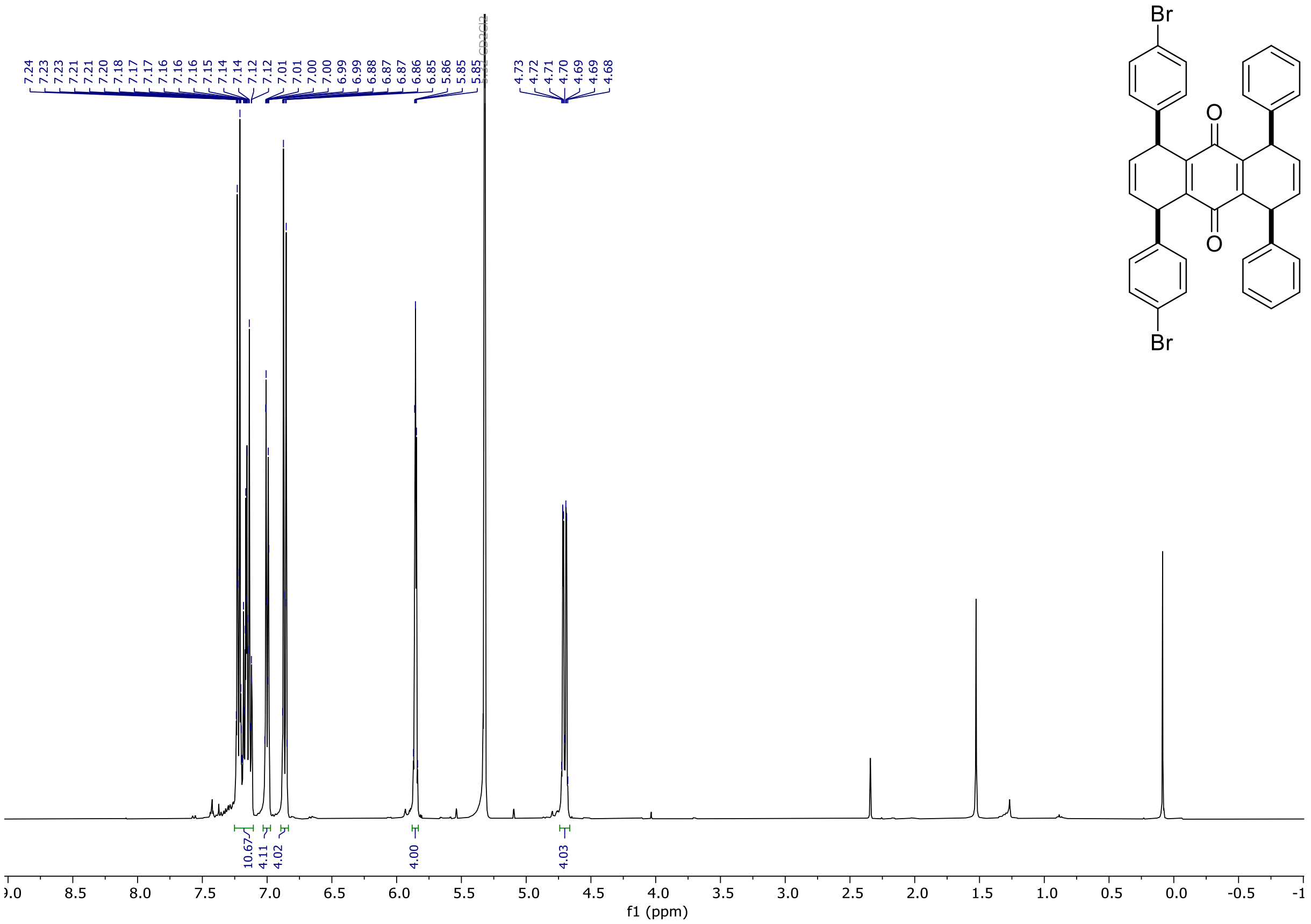

^{1}H NMR spectrum (400 MHz, CD_2Cl_2) of **8c**.

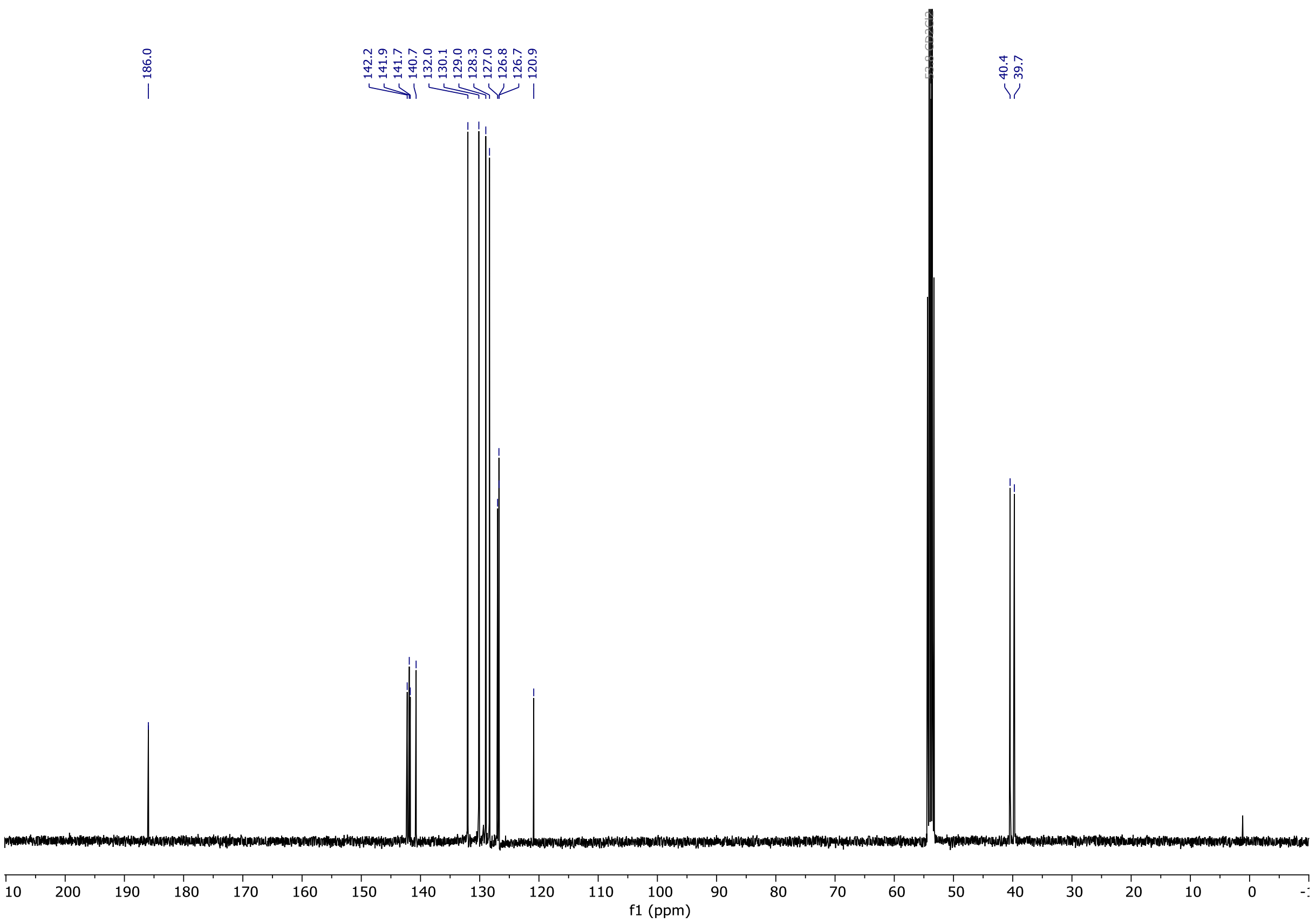

^{13}C NMR spectrum (101 MHz, CD_2Cl_2) of **8c**.

1,4,5,8,9,10-Hexaphenylanthracene (**9**)

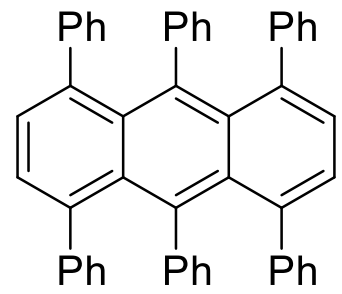

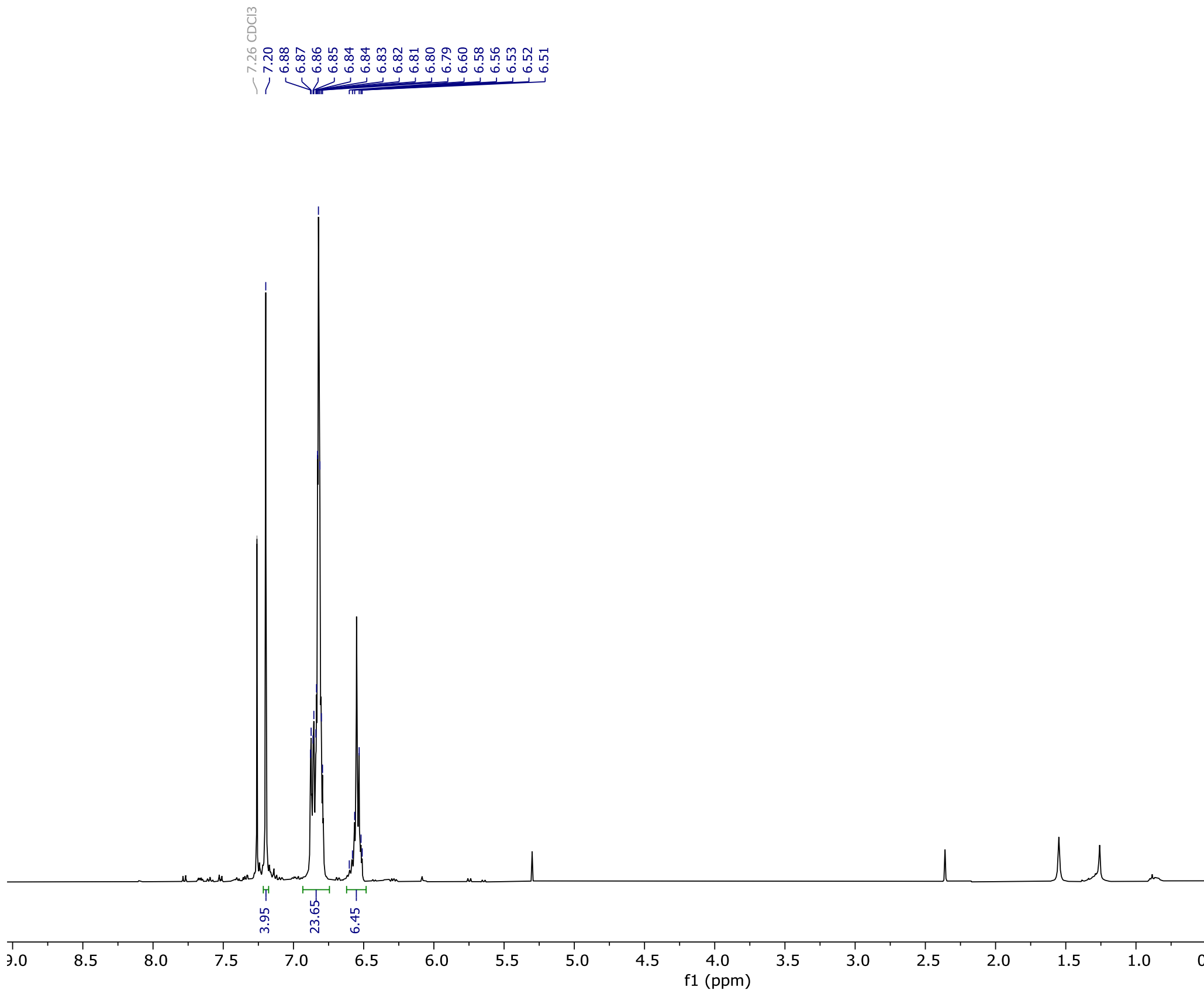

^{1}H NMR spectrum (400 MHz, $CDCl_3$) of **9**.

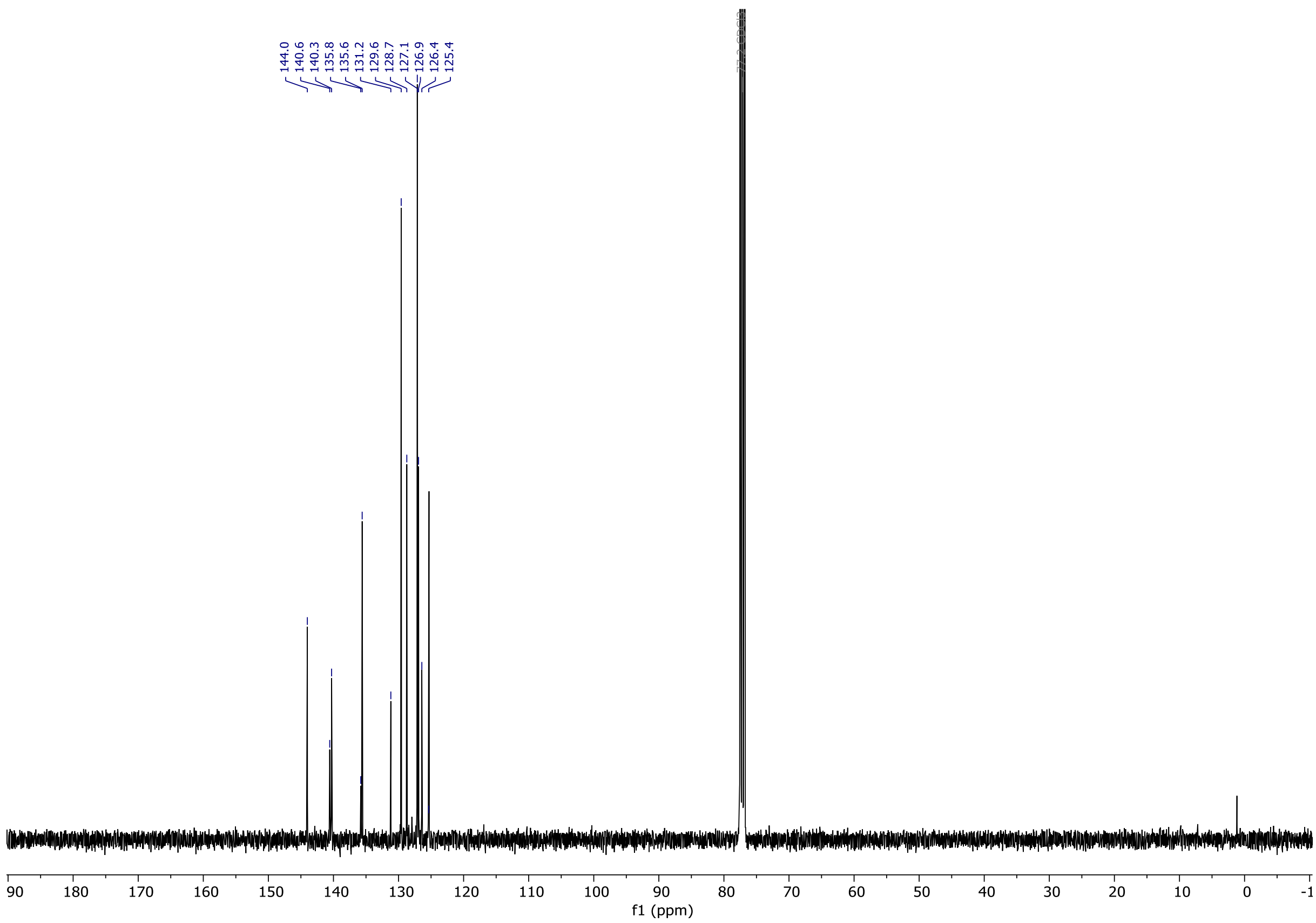

^{13}C NMR spectrum (101 MHz, $CDCl_3$) of **9**.

Methyl 1,4-Diphenyl-9,10-dioxoanthracene-6-carboxylate (**10**)

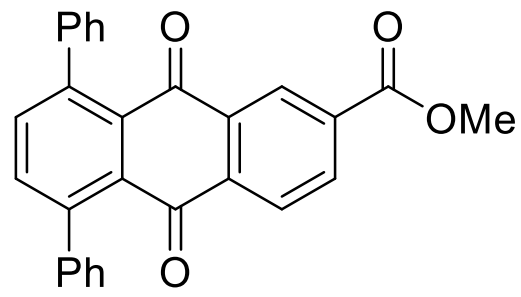

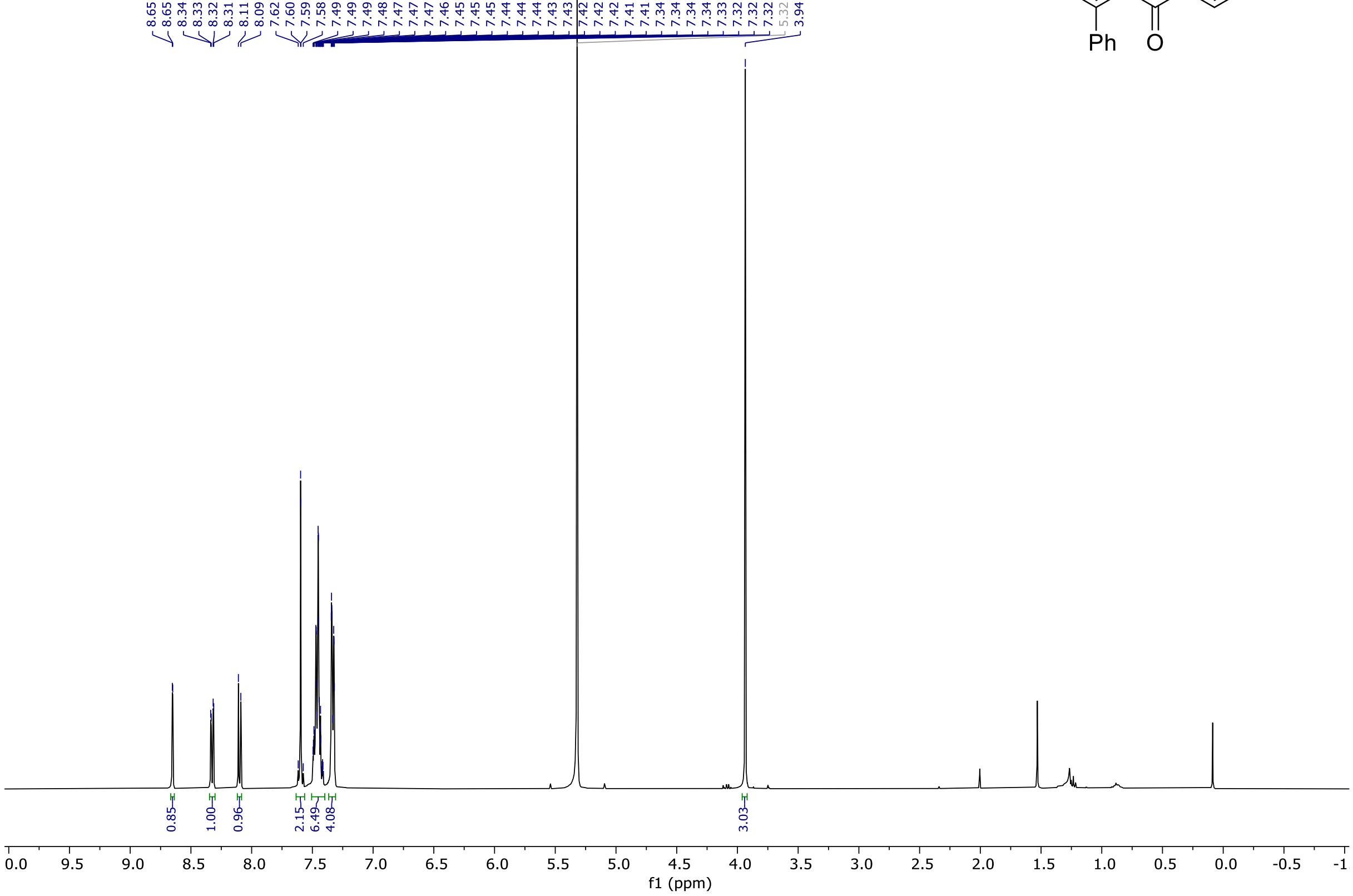

^{1}H NMR spectrum (400 MHz, CD_2Cl_2) of **10**.

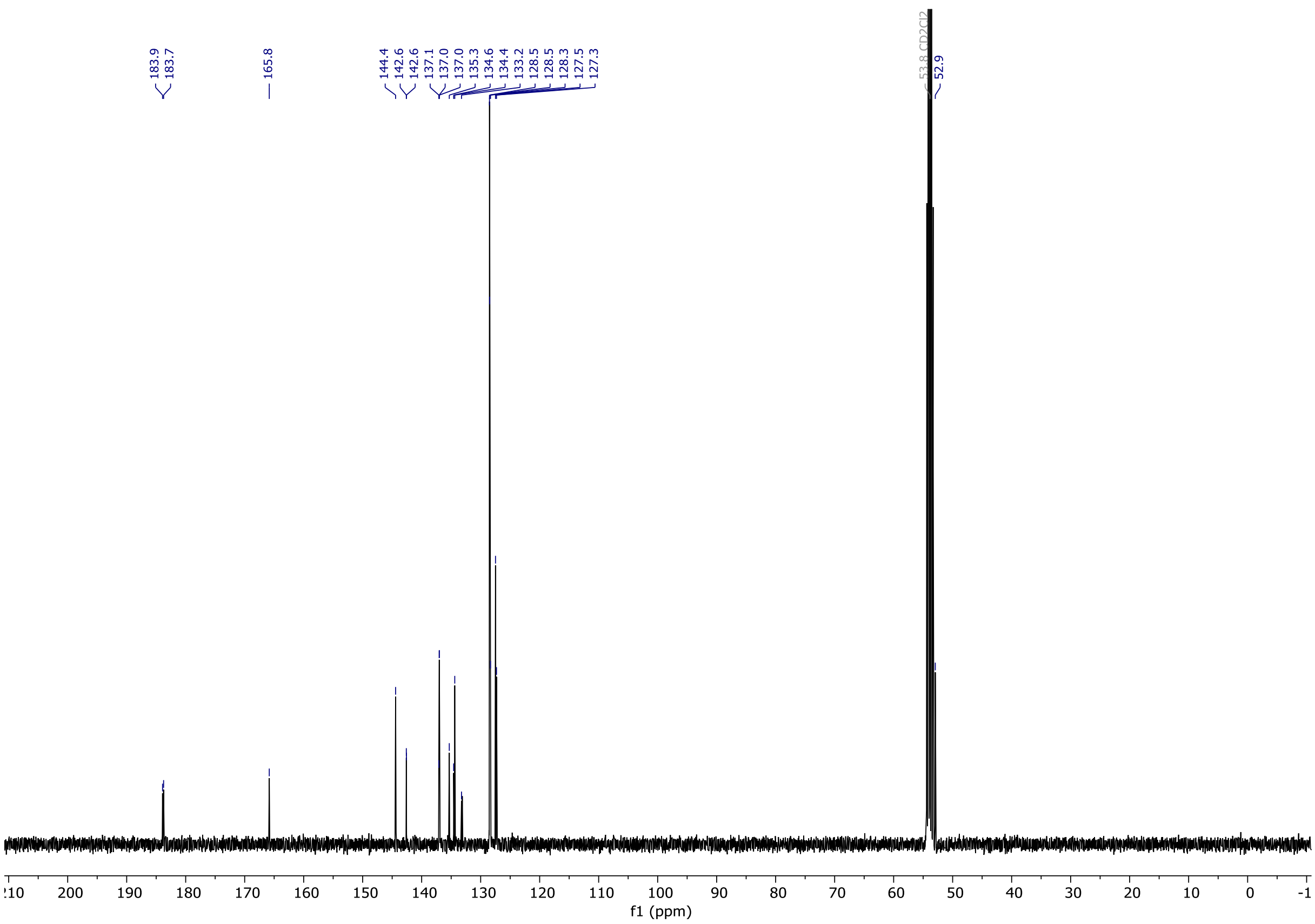

^{13}C NMR spectrum (101 MHz, CD_2Cl_2) of **10**.

3-Methyl-1,6,9-triphenylbenz[g]isoquinoline-5,10-dione (**11**)

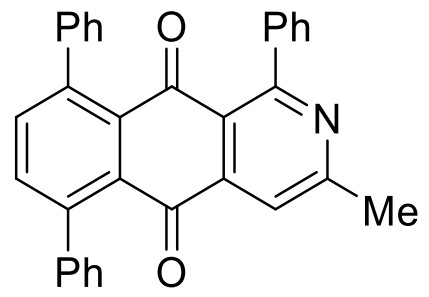

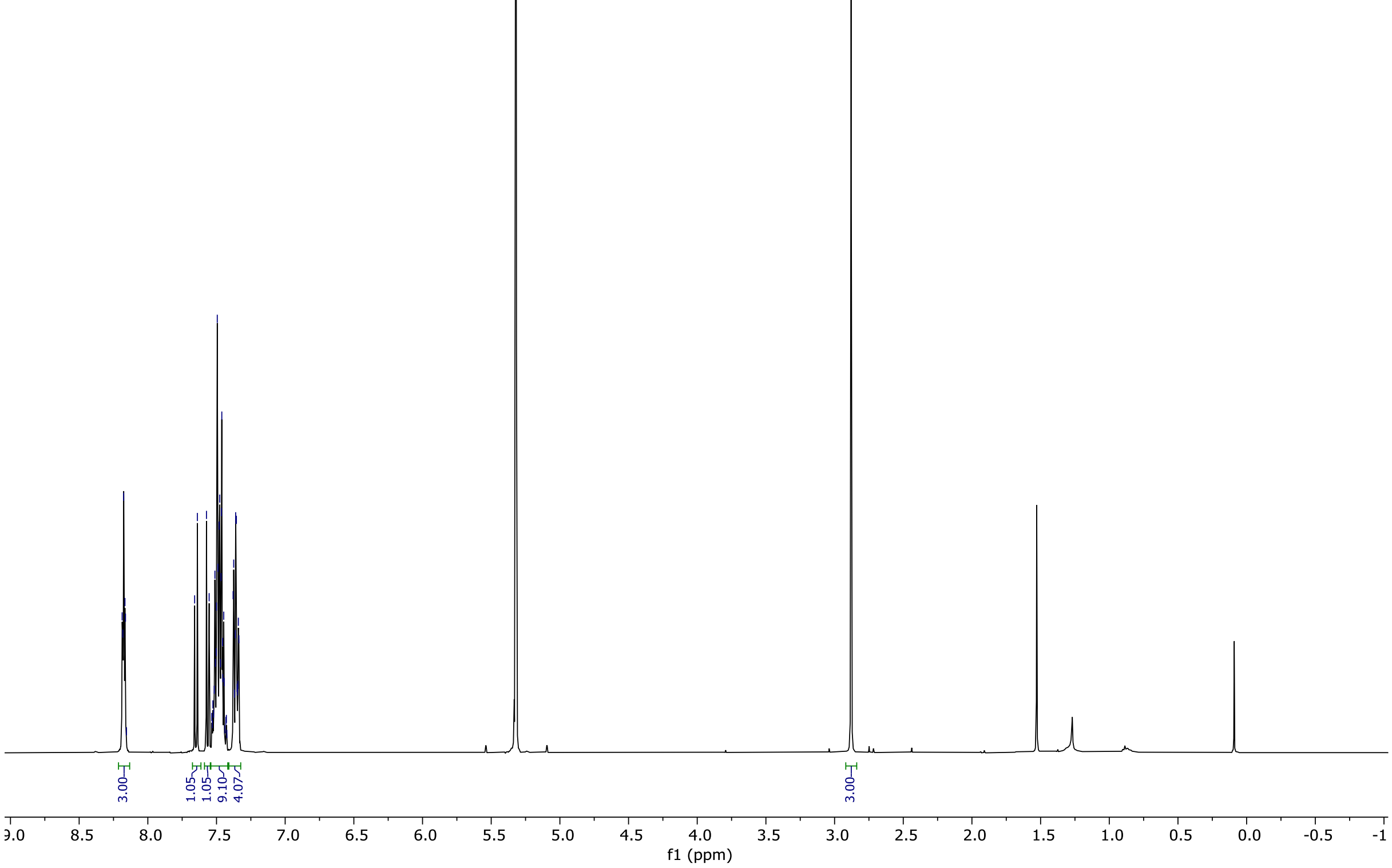

^{1}H NMR spectrum (400 MHz, CD_2Cl_2) of **11**.

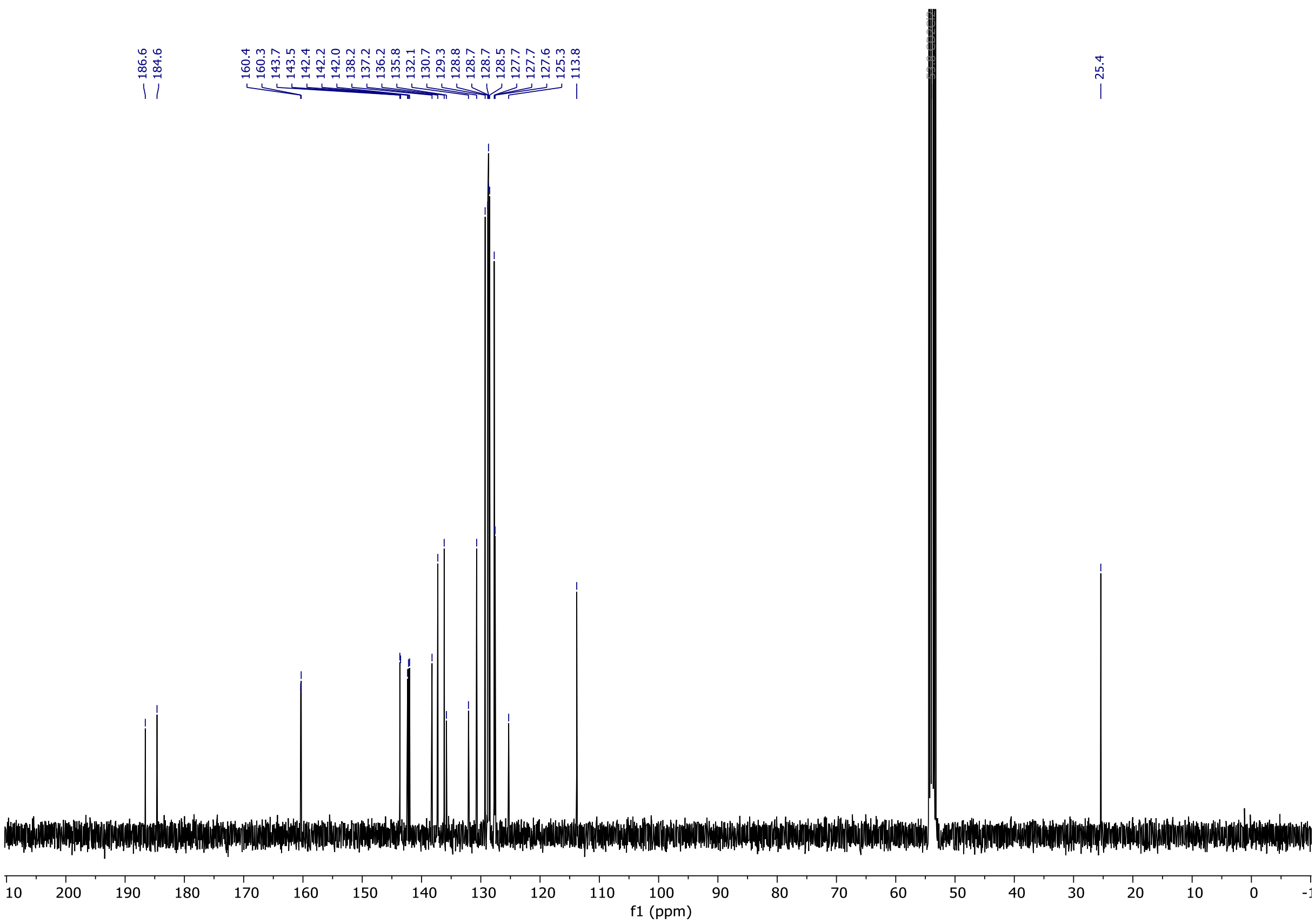

^{13}C NMR spectrum (101 MHz, CD_2Cl_2) of **11**.

1,4-Diphenylanthracene-9,10-dione (**12**)

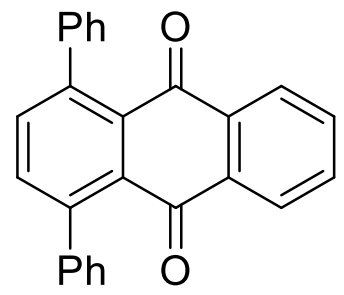

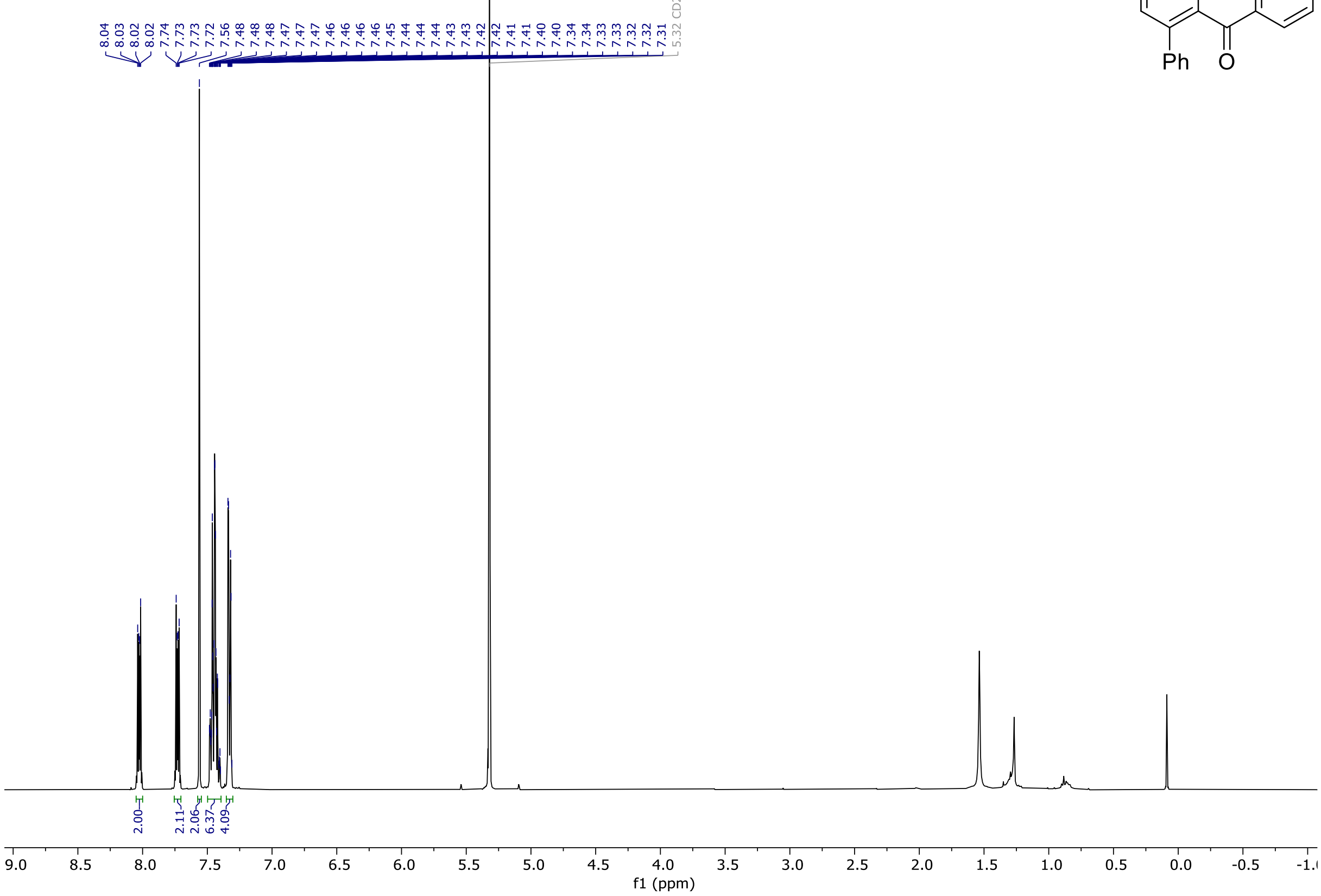

^{1}H NMR spectrum (400 MHz, CD_2Cl_2) of **12**.

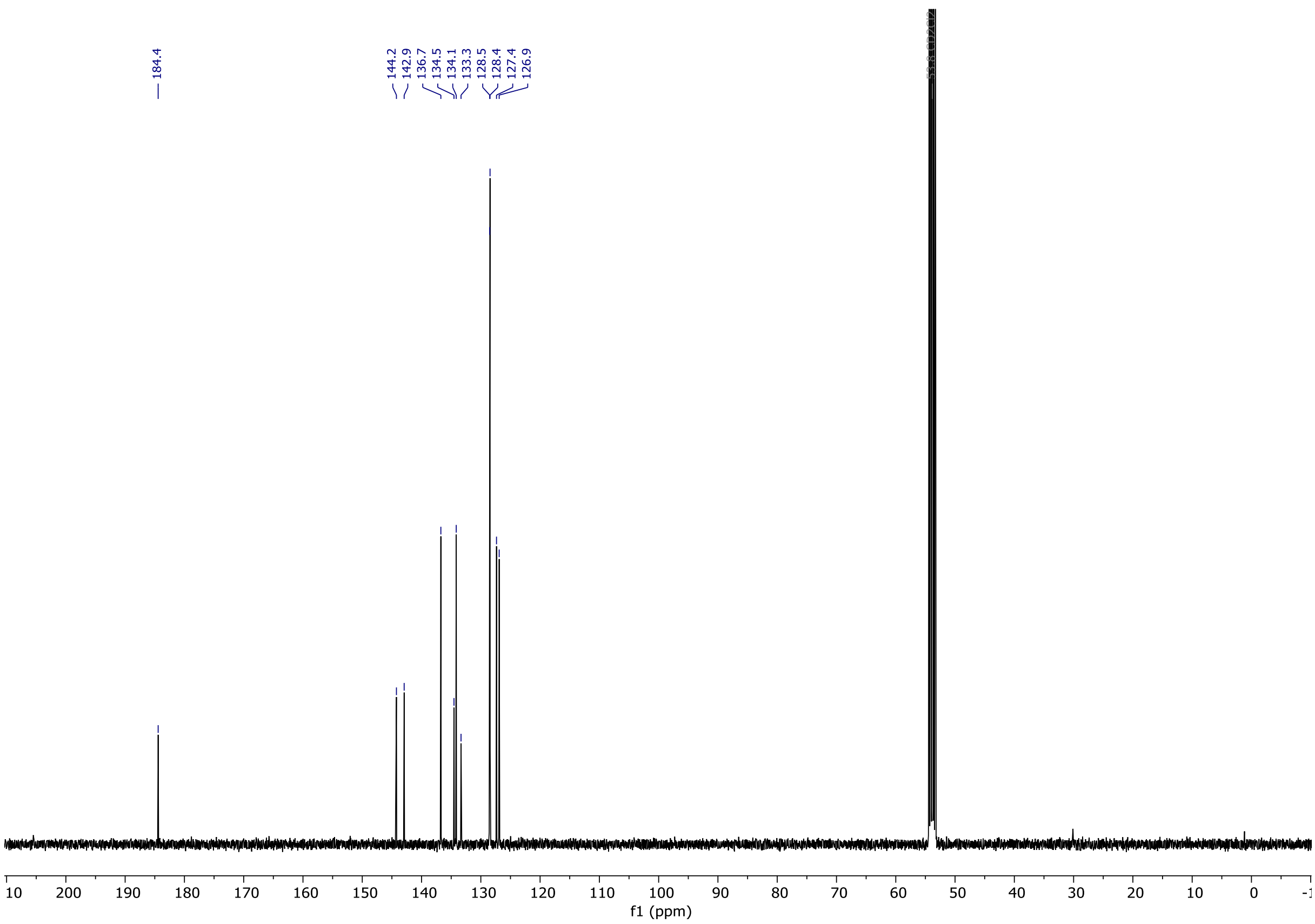

^{13}C NMR spectrum (101 MHz, CD_2Cl_2) of **12**.

1,4-Diphenylnaphthacene-5,12-dione (**13**)

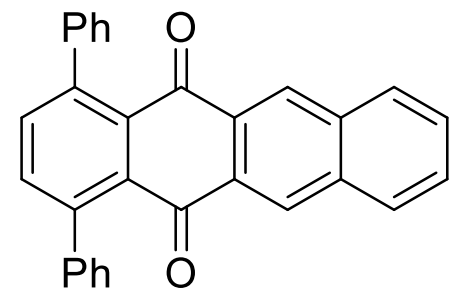

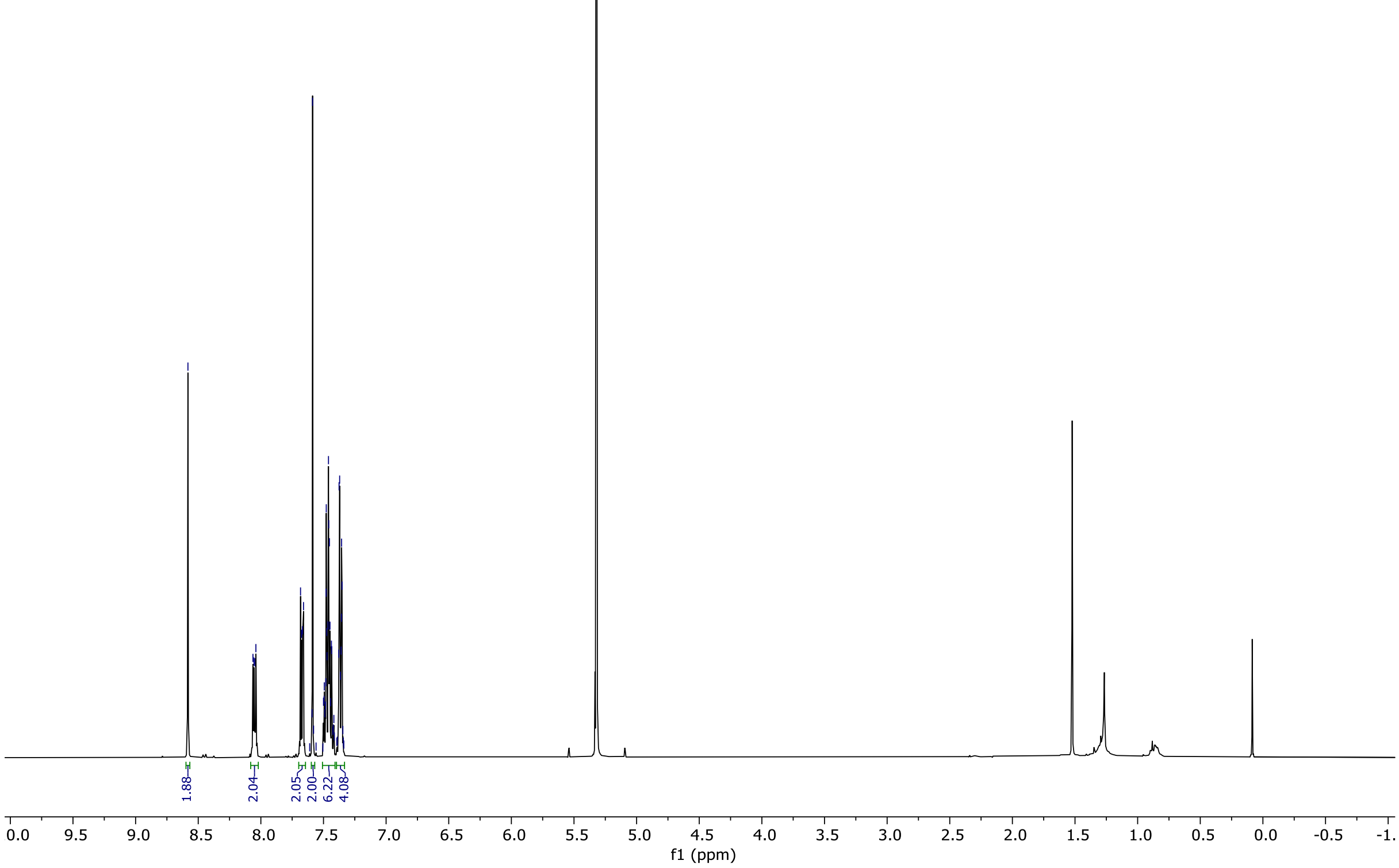

^{1}H NMR spectrum (400 MHz, CD_2Cl_2) of **13**

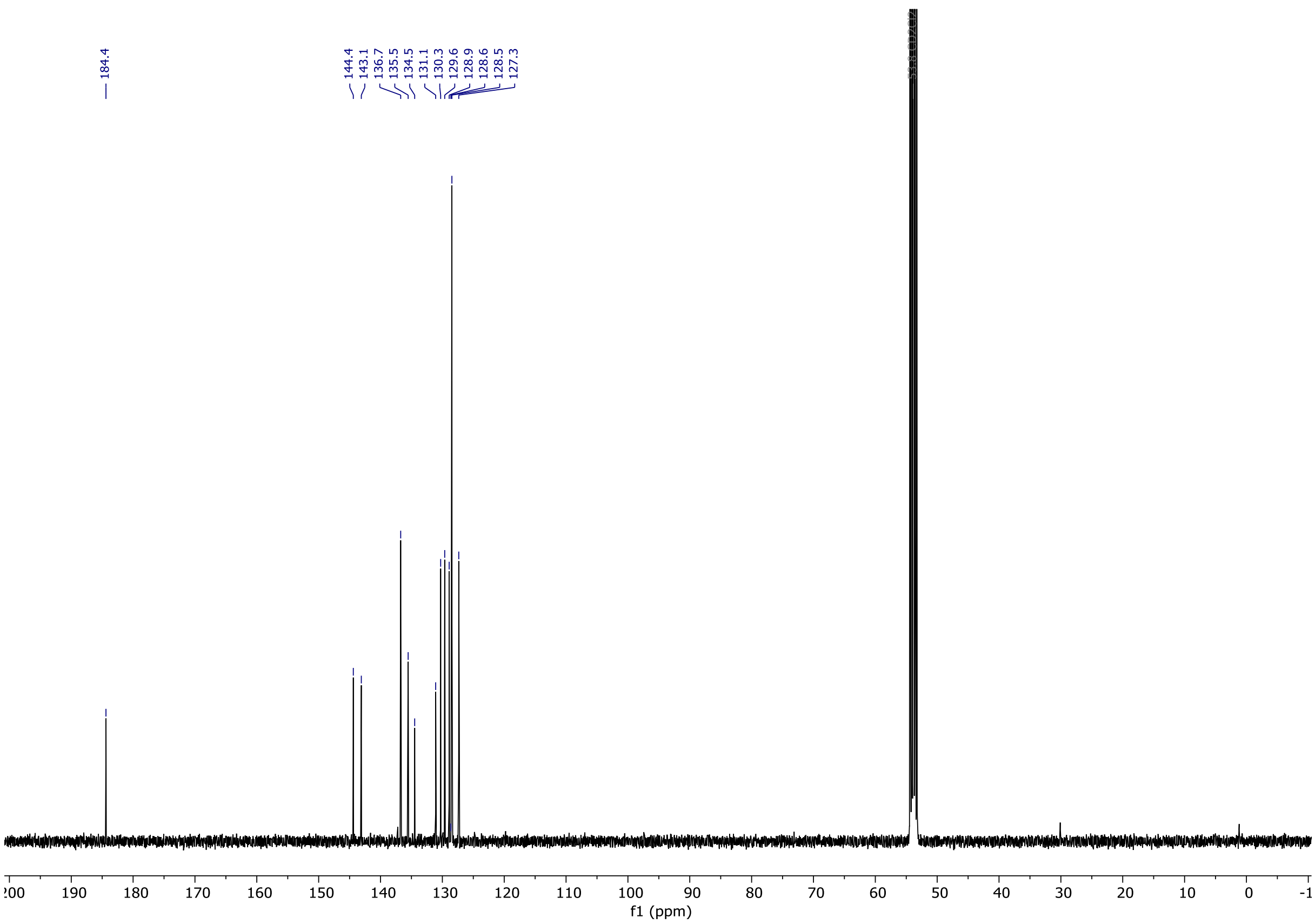

^{13}C NMR spectrum (101 MHz, CD_2Cl_2) of **13**.

1,4-Diphenylpentacene-5,14-dione (**14**)

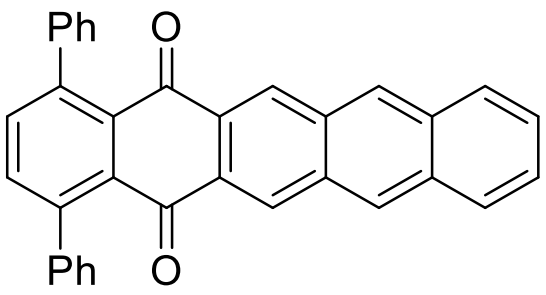

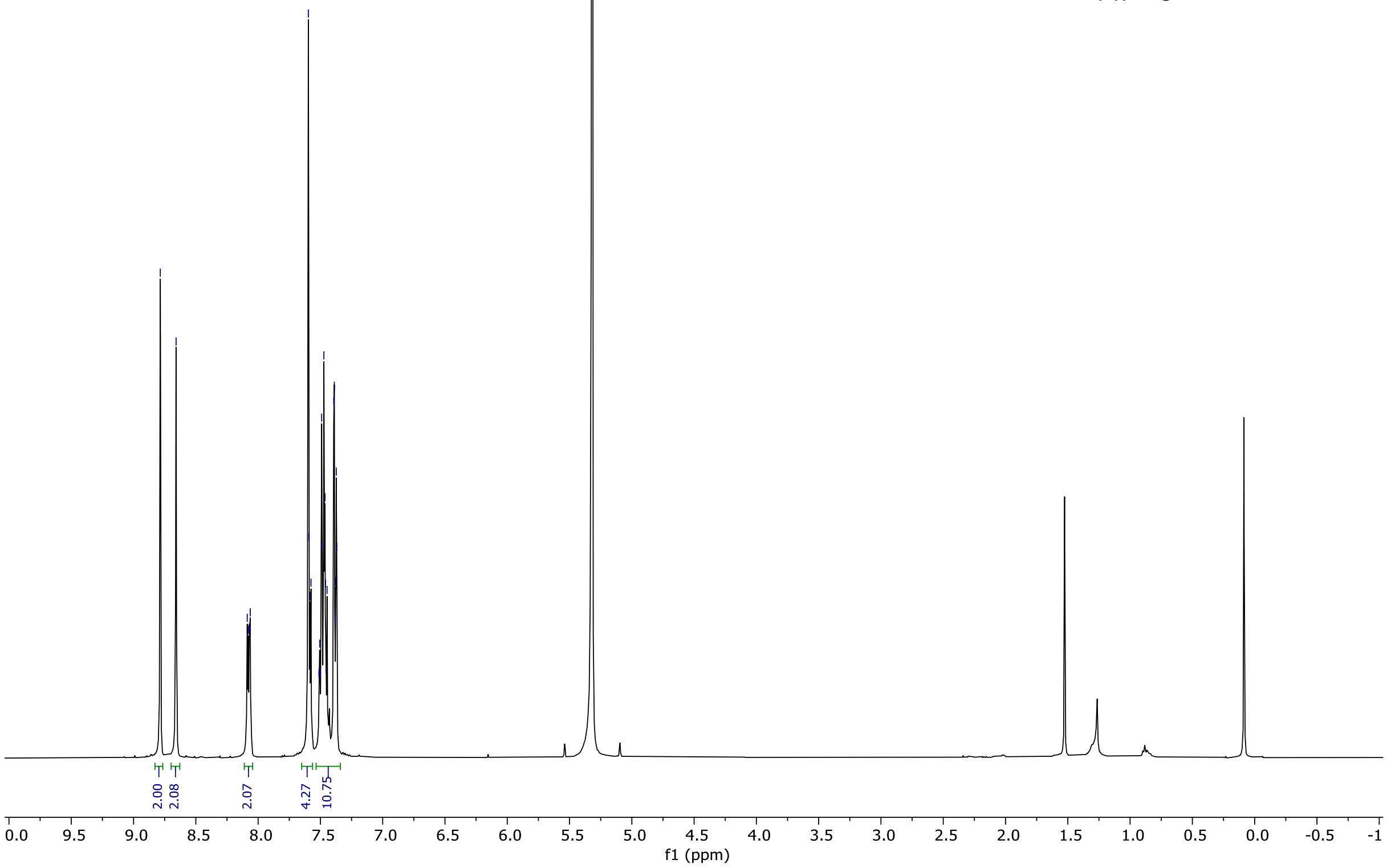

^{1}H NMR spectrum (400 MHz, CD_2Cl_2) of **14**.

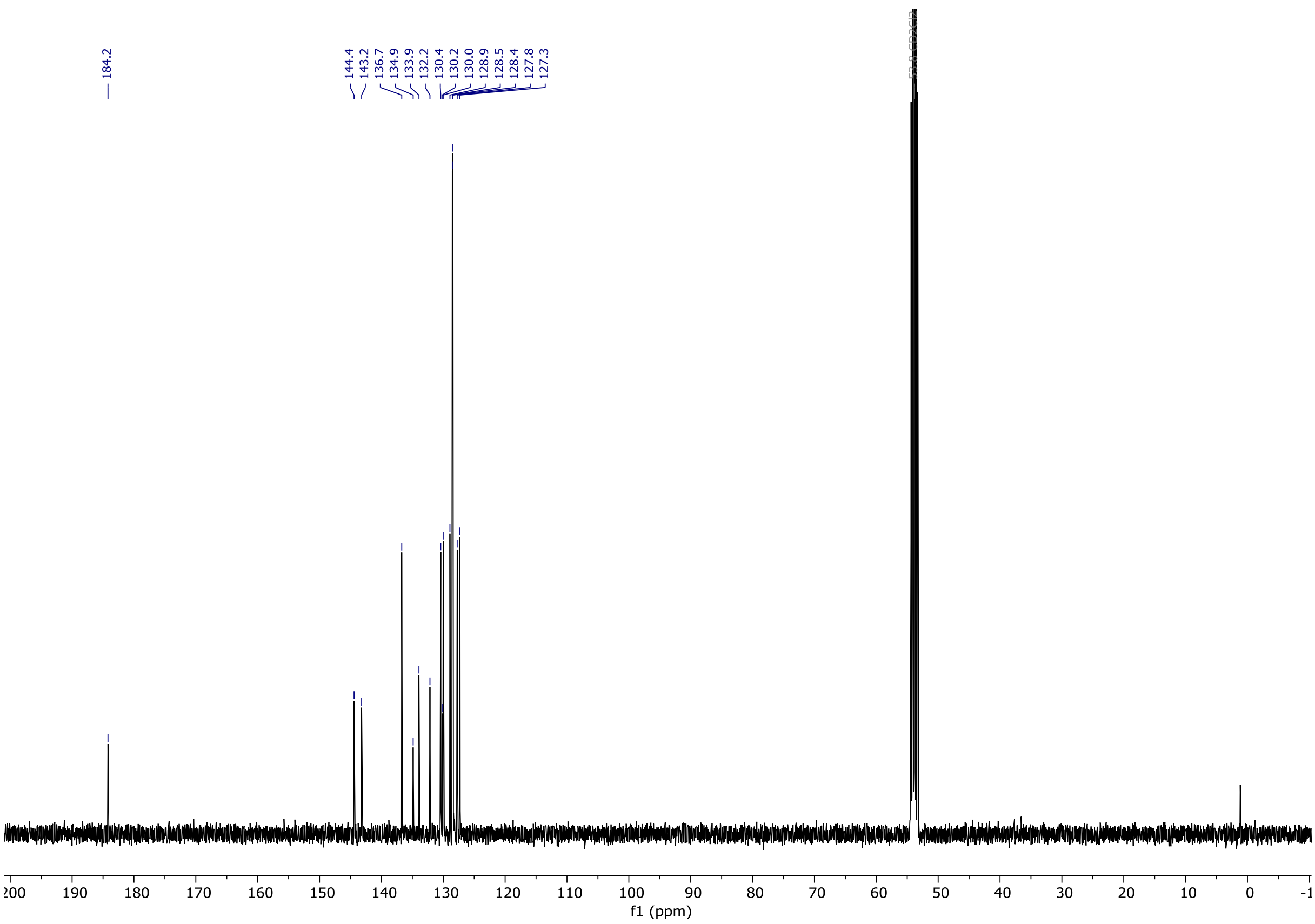

^{13}C NMR spectrum (101 MHz, CD_2Cl_2) of **14**.

3-Methyl-5-phenyl-2H-1,4-oxazin-2-one (**16**)

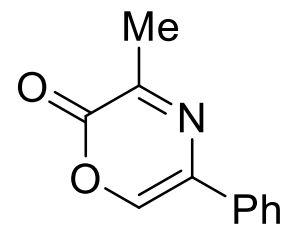

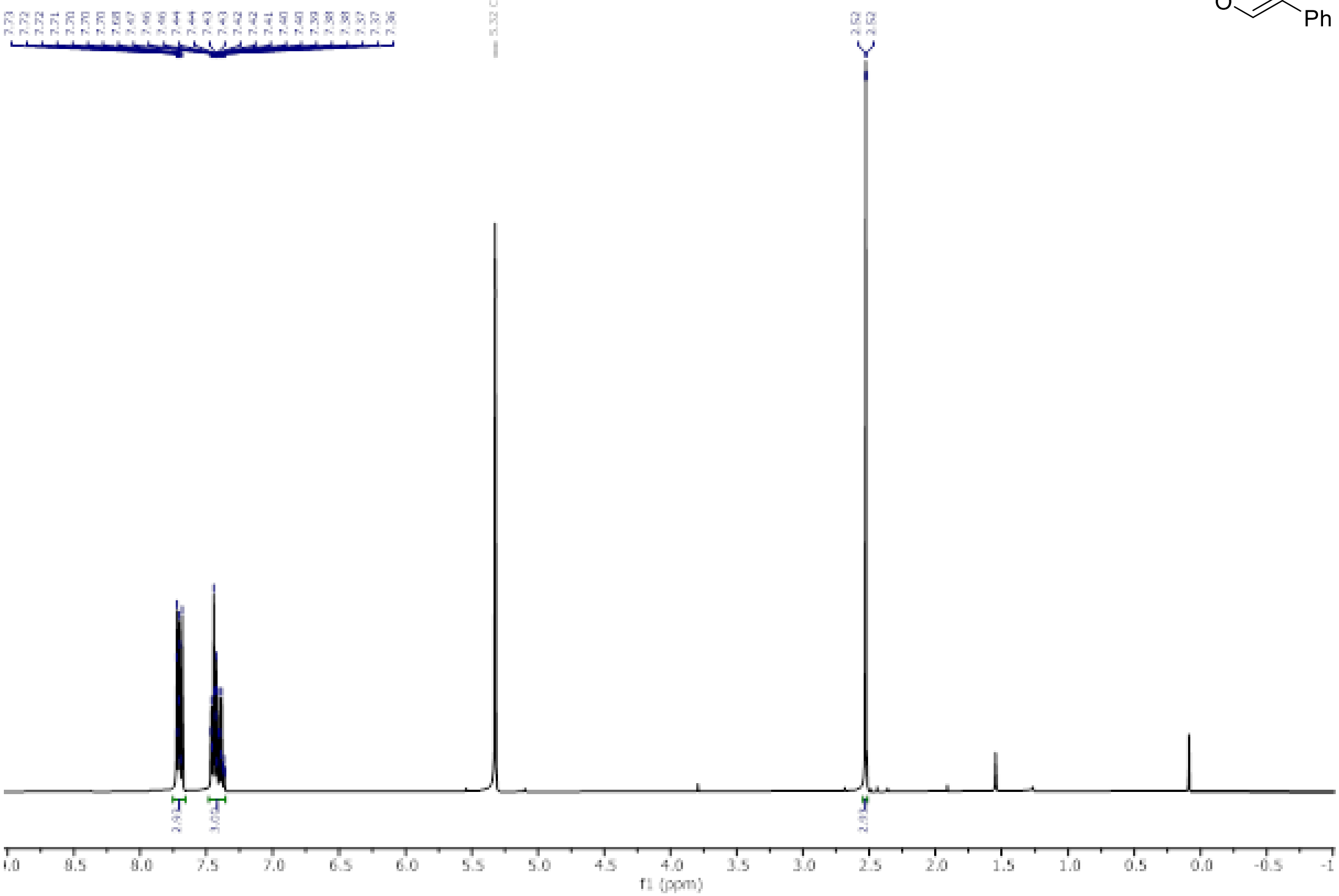

^{1}H NMR spectrum (400 MHz, CD_2Cl_2) of **16**.

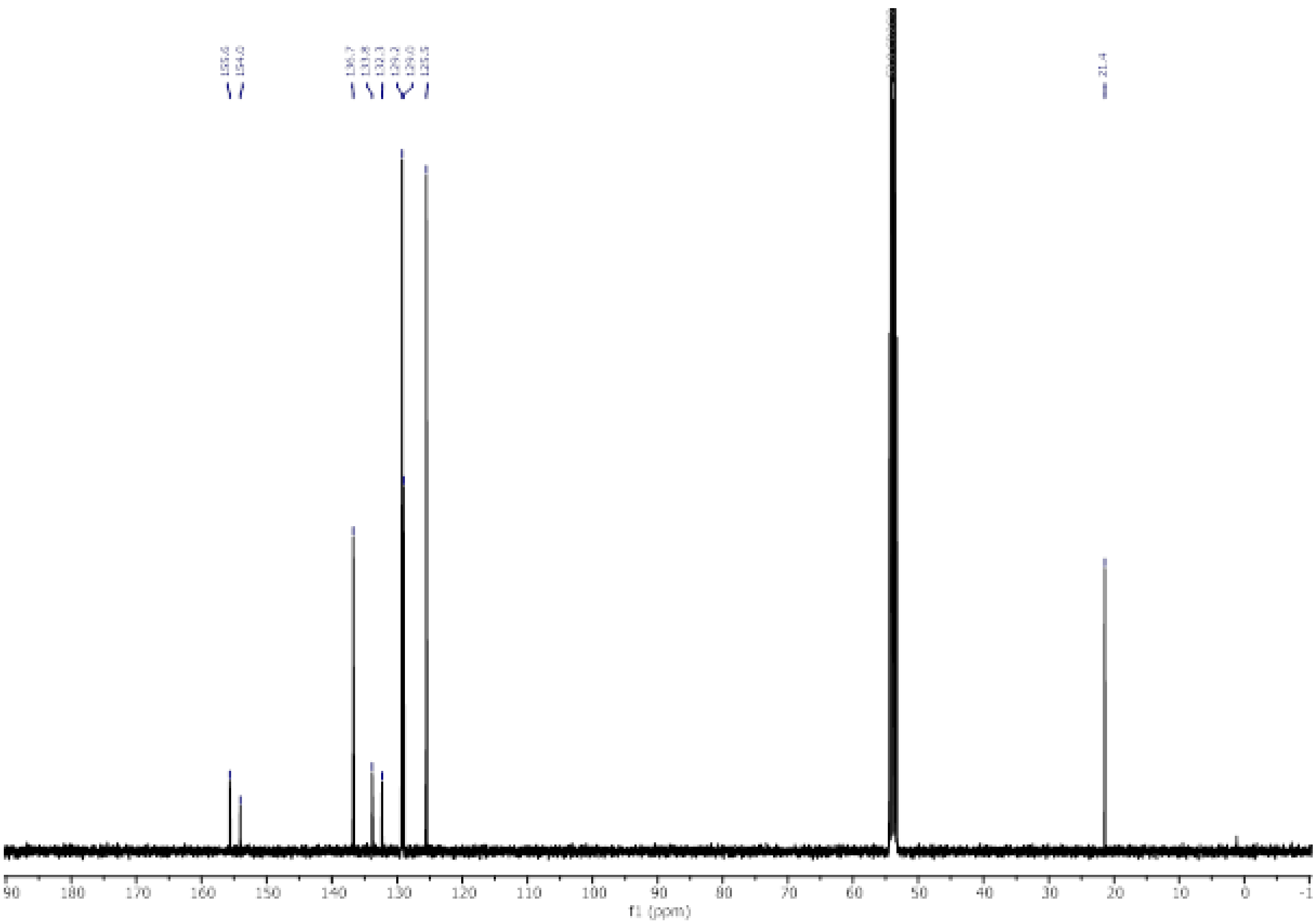

^{13}C NMR spectrum (101 MHz, CD_2Cl_2) of **16**.

Naphthalene-2,3-dicarboxaldeyhde (**18**)

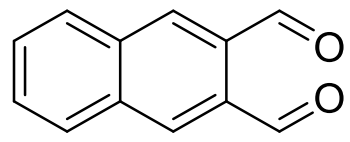

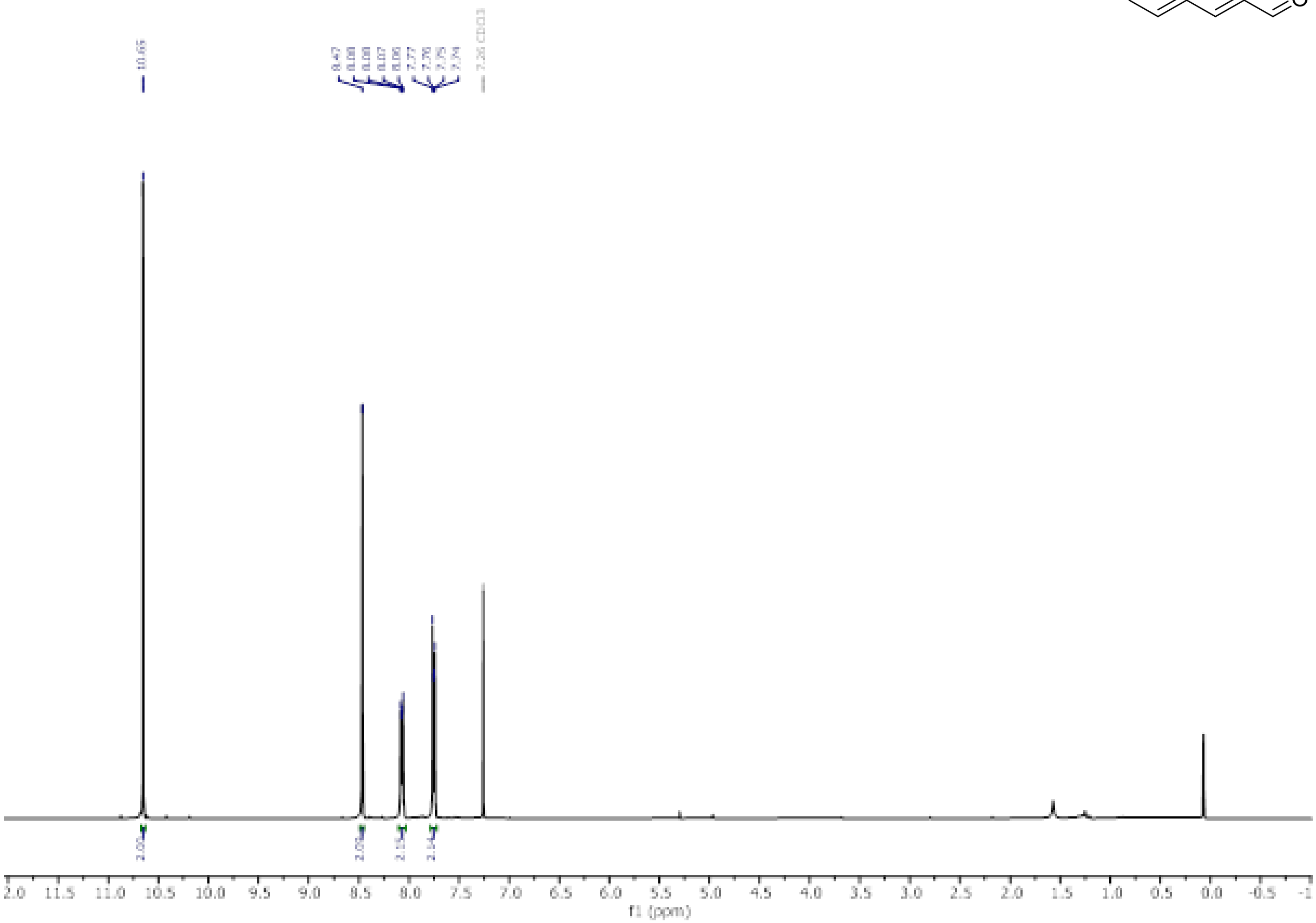

^{1}H NMR spectrum (400 MHz, CD_2Cl_2) of **18**.

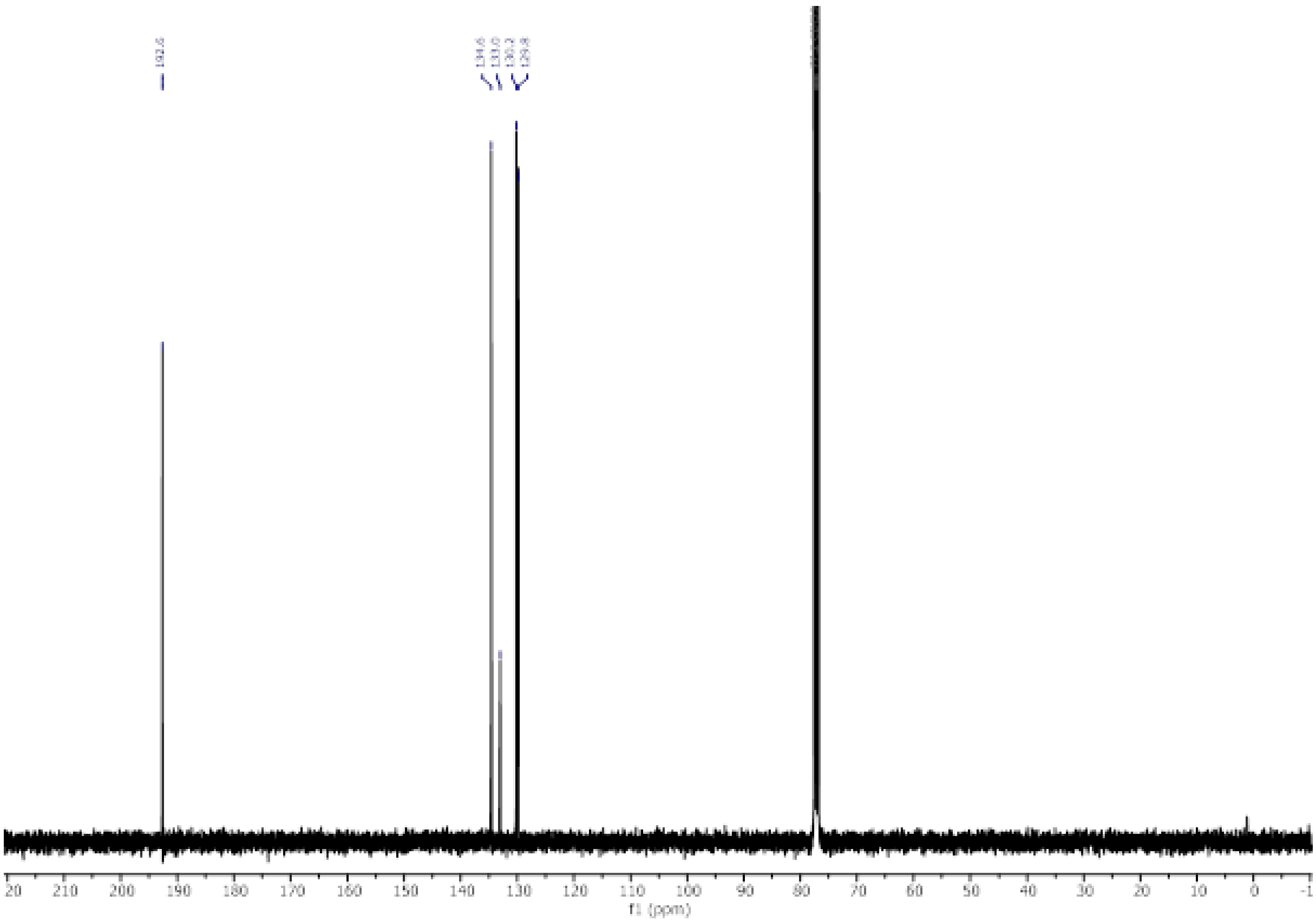

^{13}C NMR spectrum (101 MHz, CD_2Cl_2) of **18**.

2,3-Dihydro-2,3-epoxy-5,8-diphenylnaphthalene-1,4-dione (**19**)

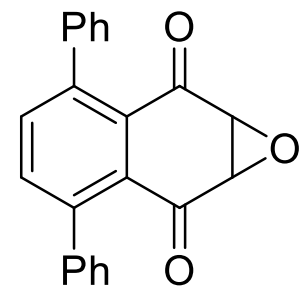

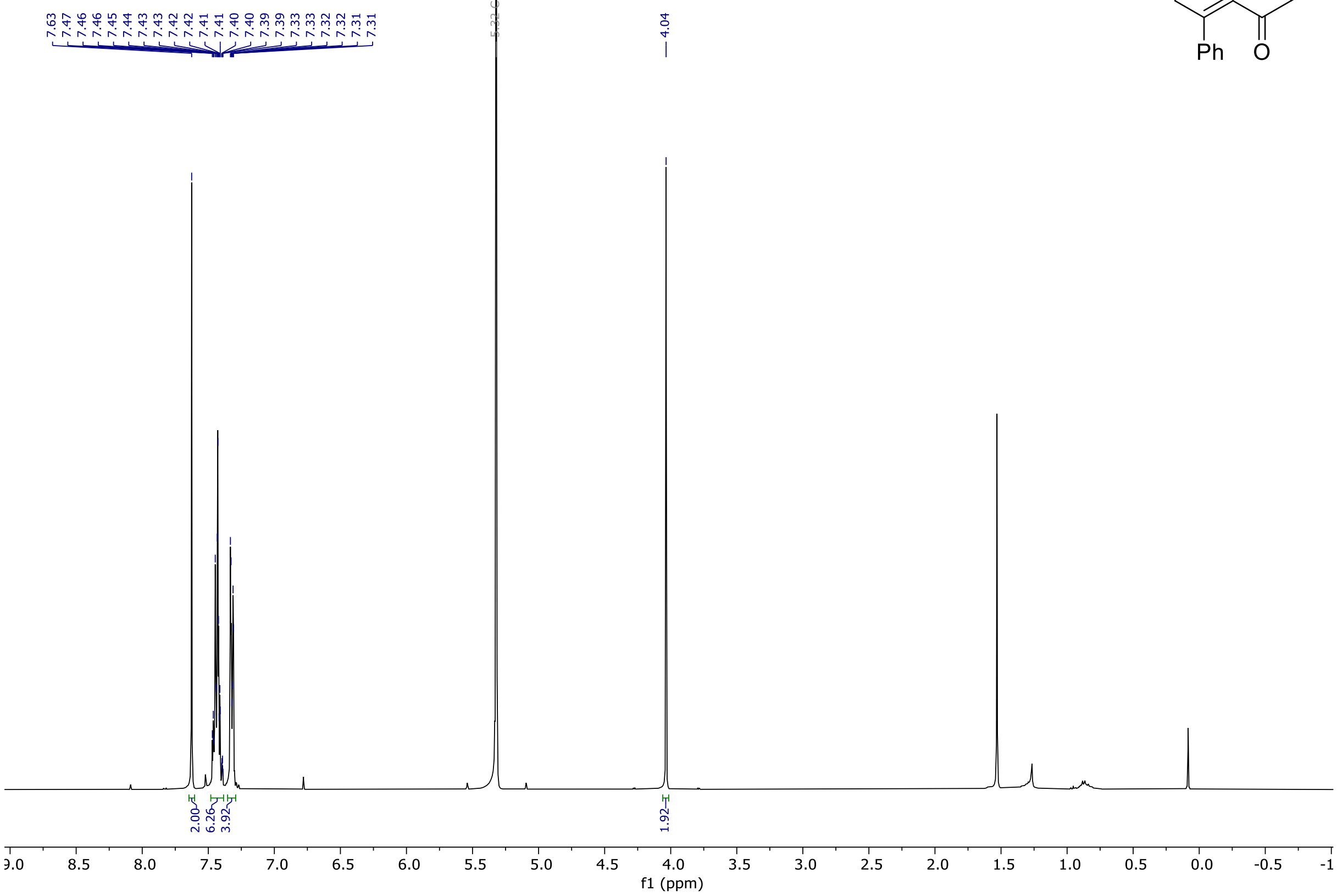

^1H NMR spectrum (400 MHz, CD_2Cl_2) of **19**.

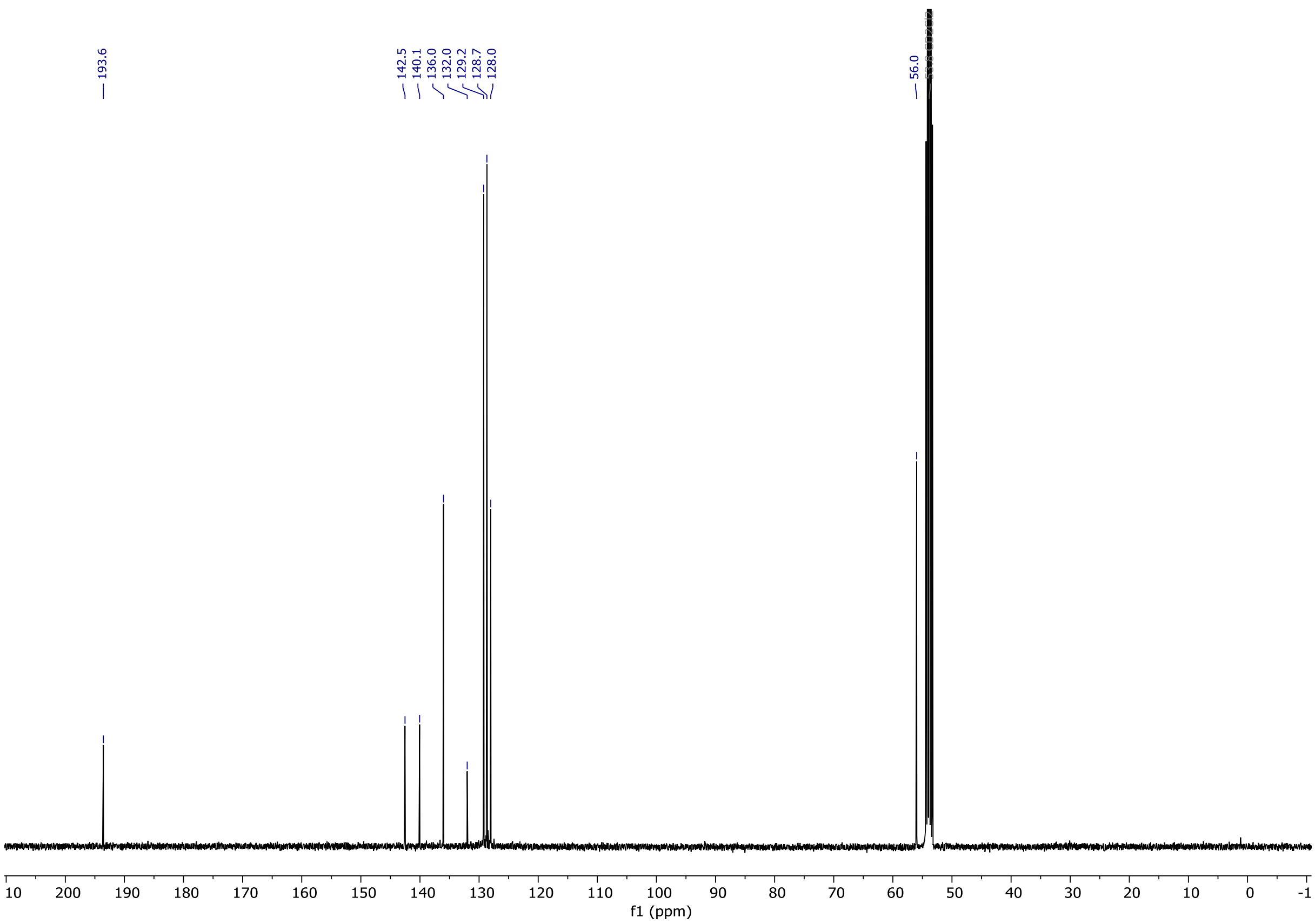

^{13}C NMR spectrum (101 MHz, CD_2Cl_2) of **19**.

2-(4-Methoxyphenyl)amino-5,8-diphenylnaphthalene-1,4-dione (**20**)

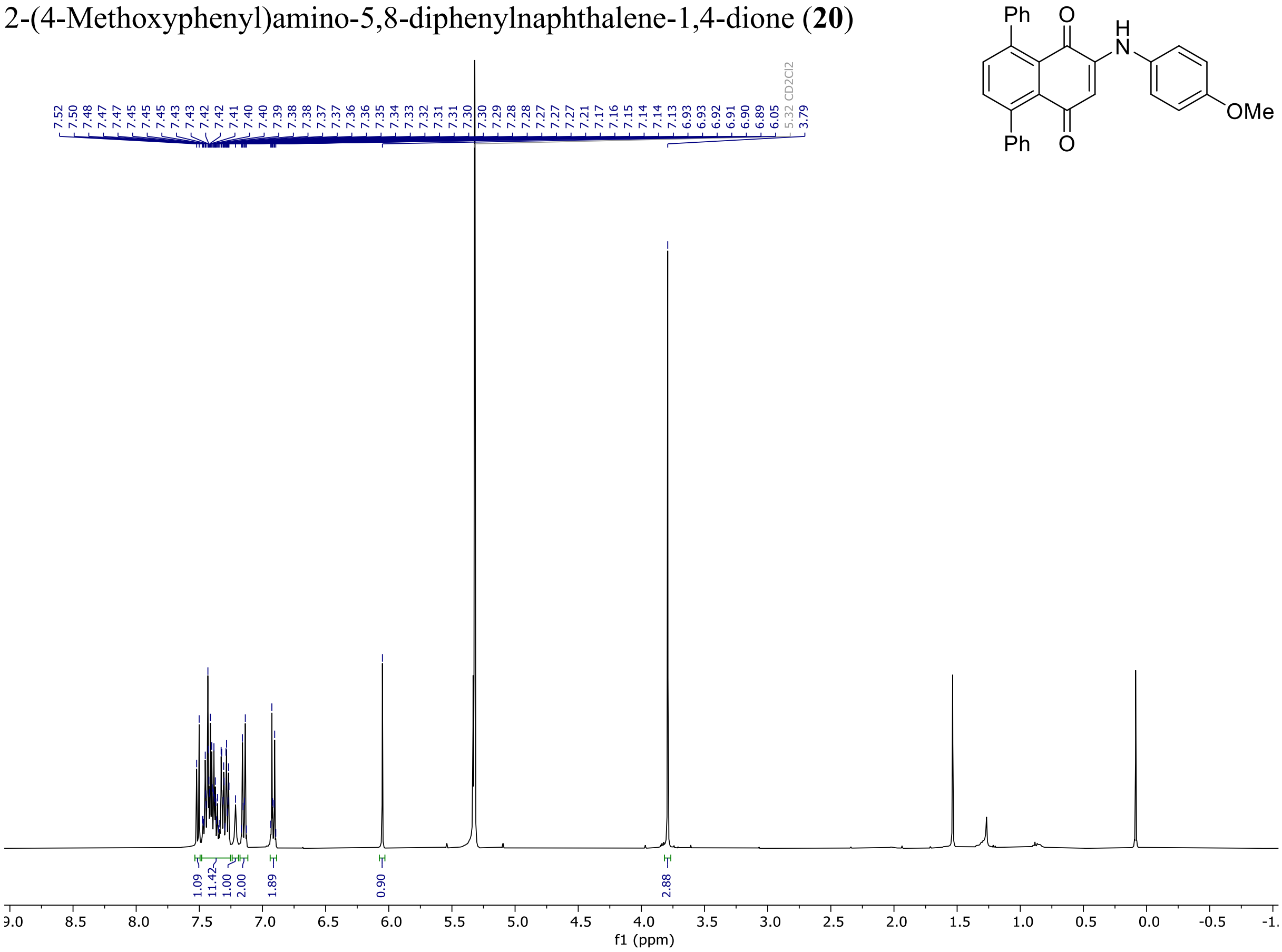

^{1}H NMR spectrum (400 MHz, CD_2Cl_2) of **20**.

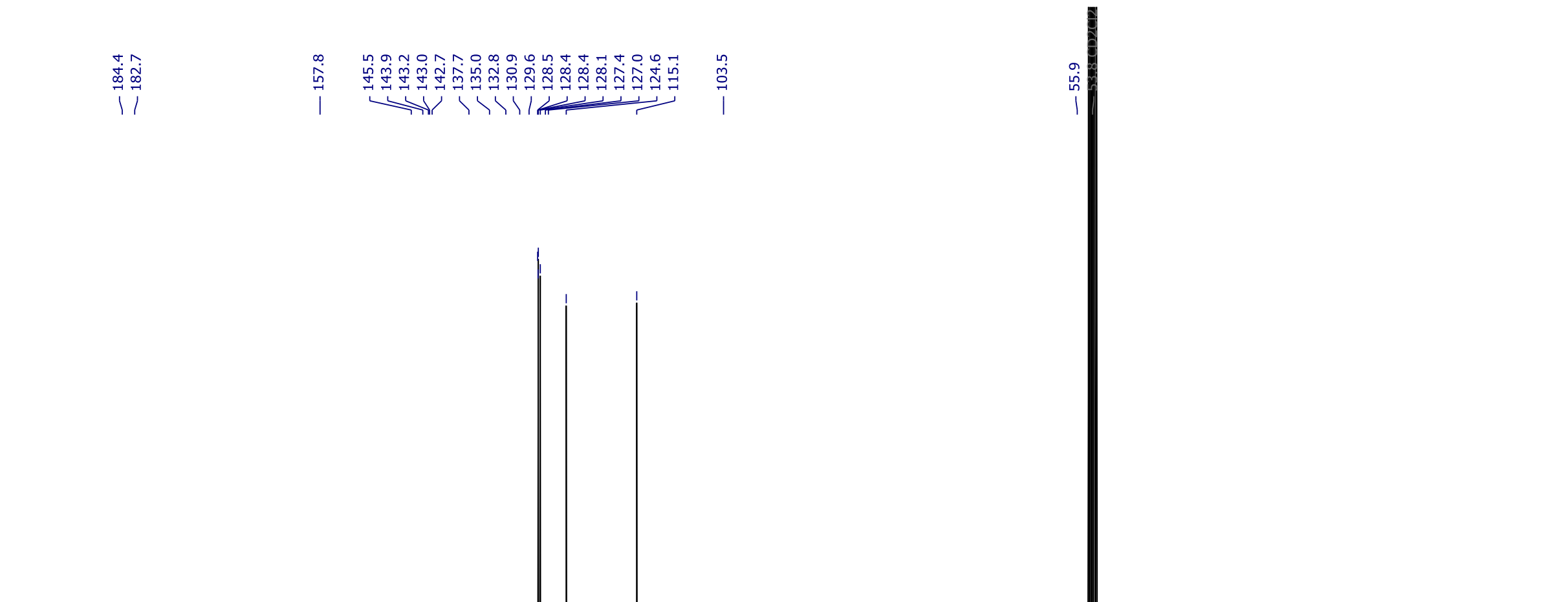

^{13}C NMR spectrum (101 MHz, CD_2Cl_2) of **20**.

1,4-Dimethoxy-5,8-diphenylnaphthalene (**21**)

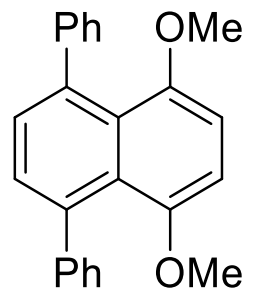

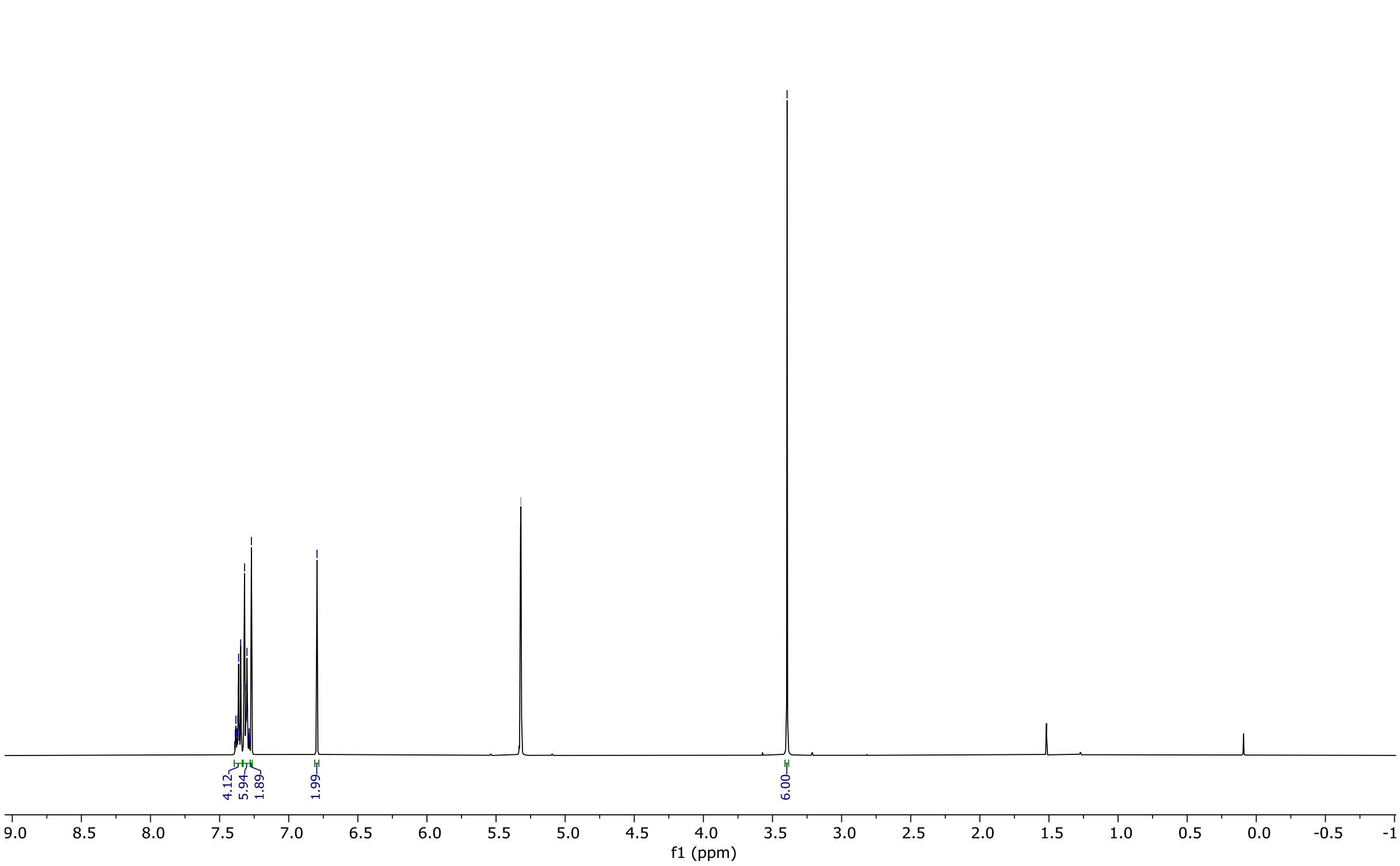

^{1}H NMR spectrum (400 MHz, CD_2Cl_2) of **21**

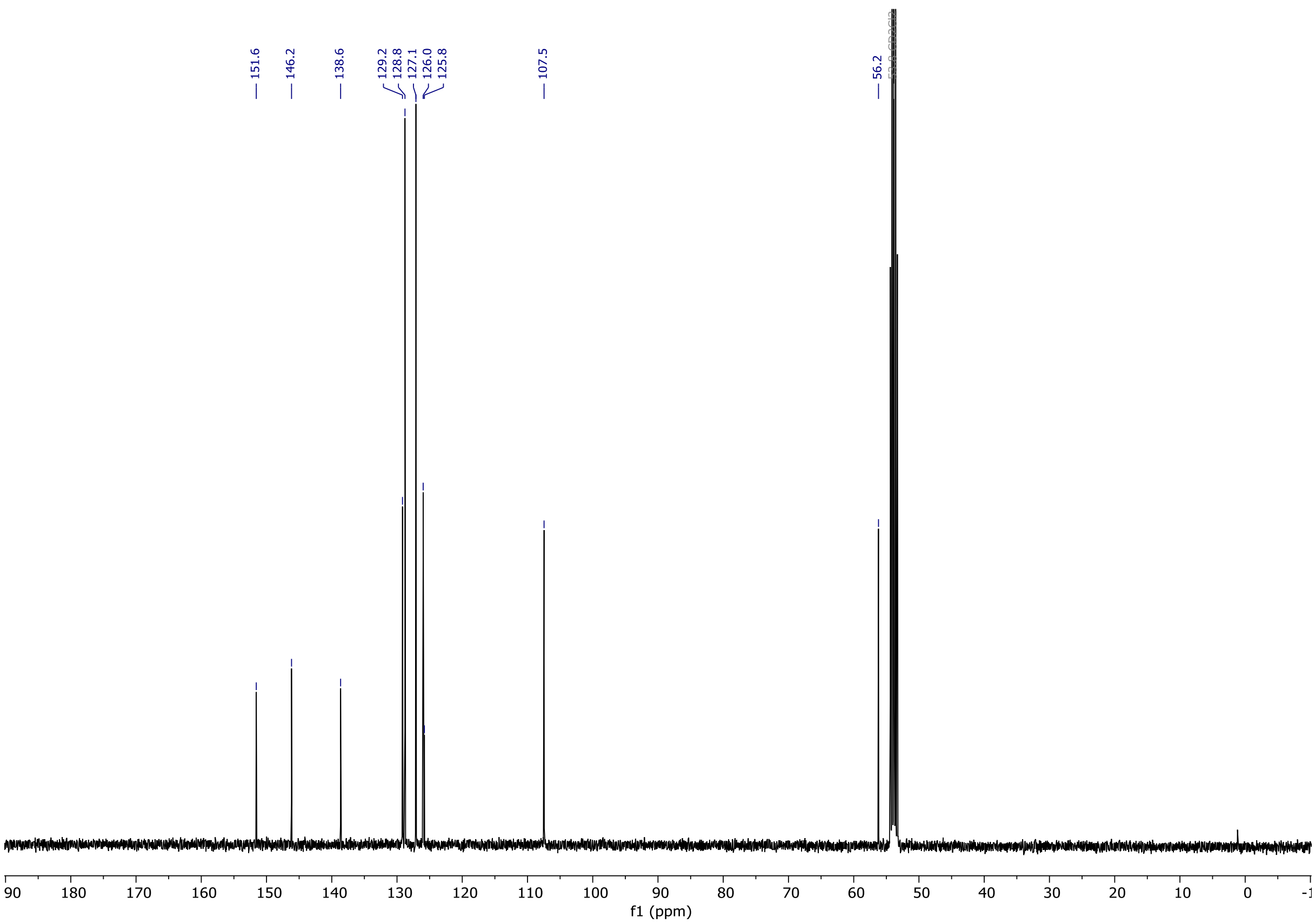

^{13}C NMR spectrum (101 MHz, CD_2Cl_2) of **21**.

1,4-Dimethoxy-5,8-diphenyl-2-napthalenecarboxaldehyde (**22**)

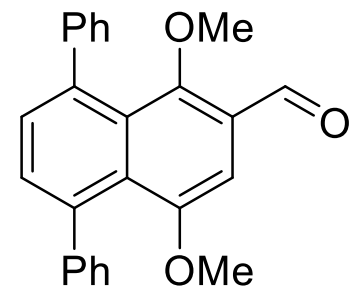

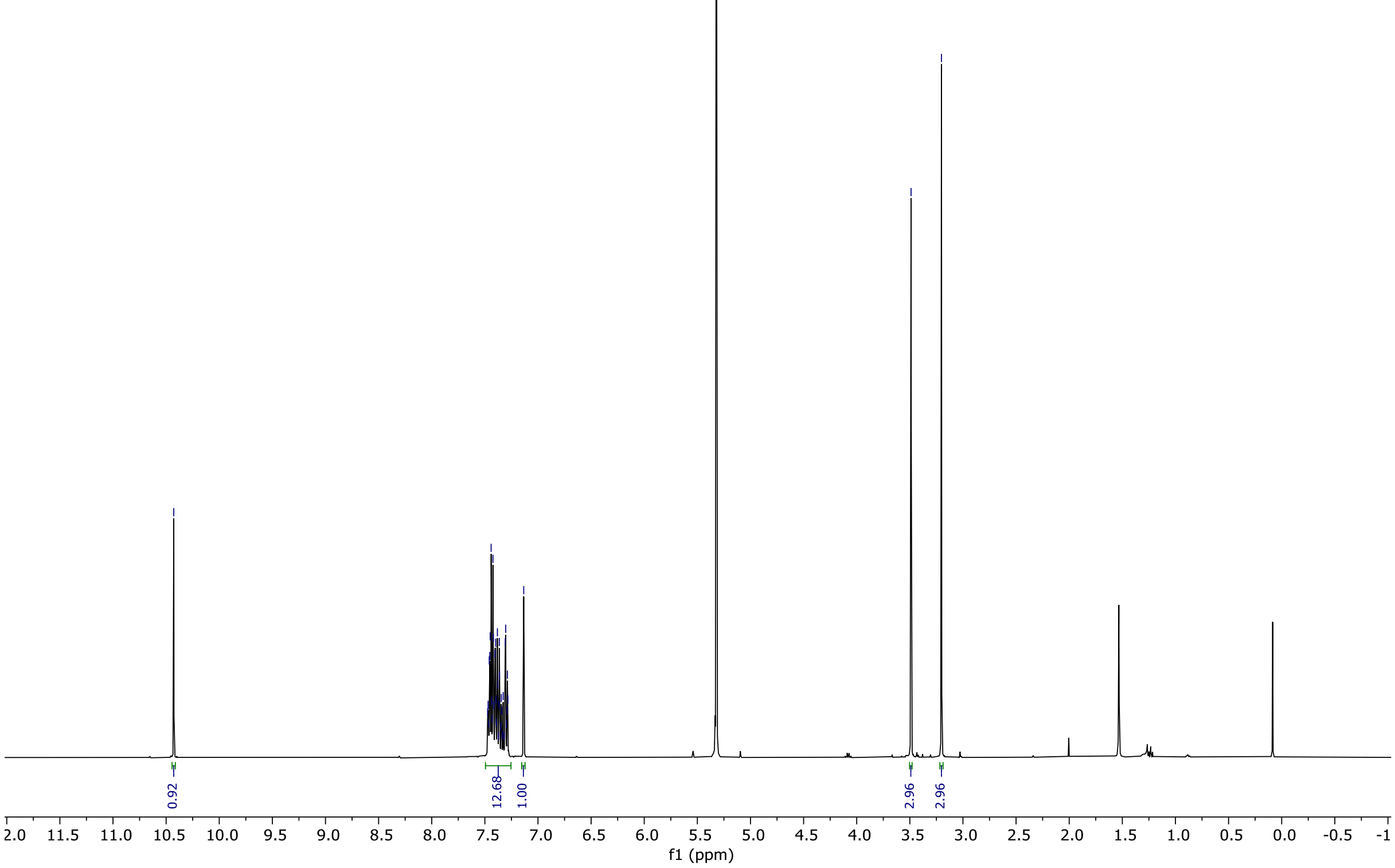

^{1}H NMR spectrum (400 MHz, CD_2Cl_2) of **22**

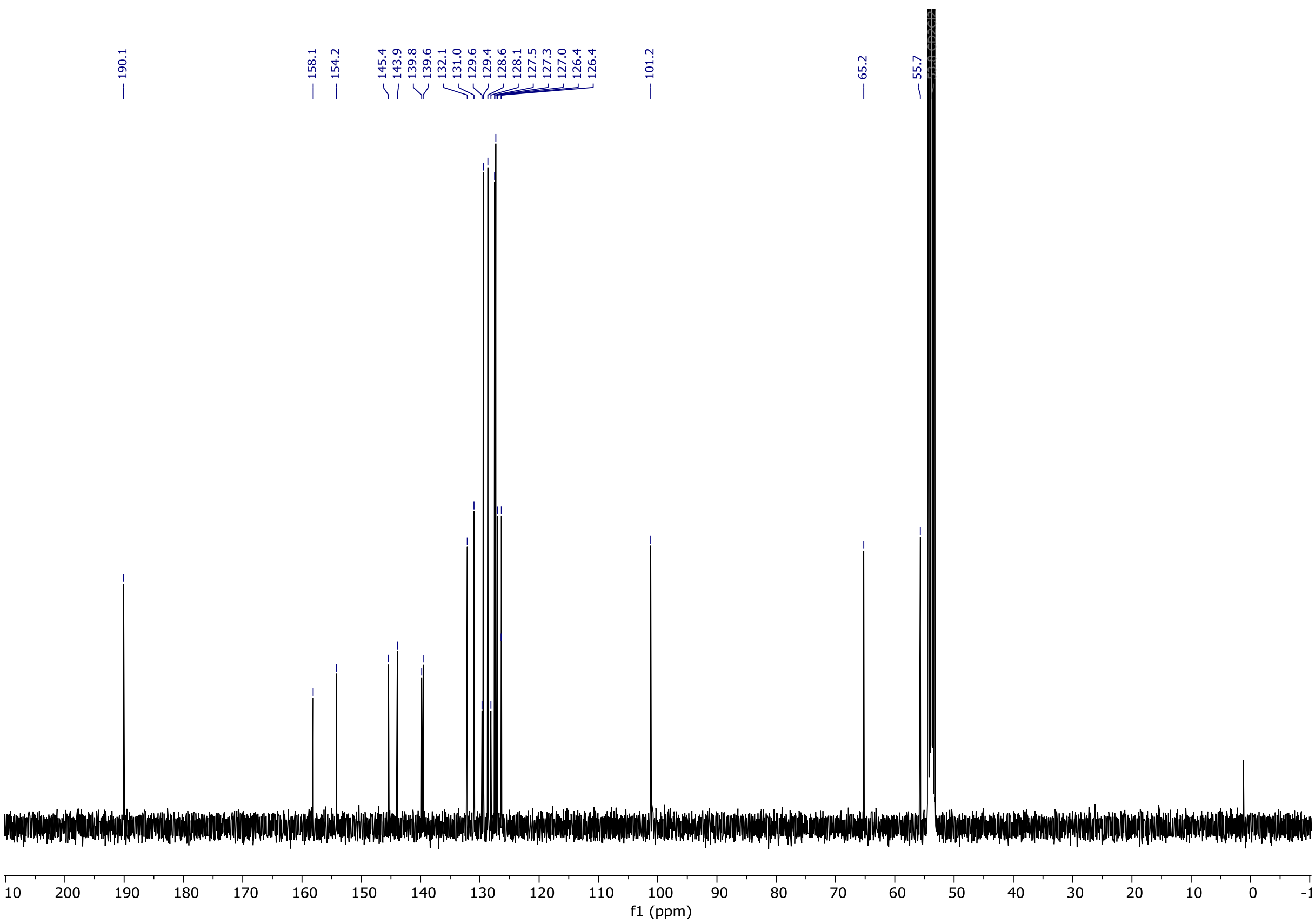

^{13}C NMR spectrum (101 MHz, CD_2Cl_2) of **22**.

5,8-Dihydro-5,8-diphenyl-1,4-naphthalenediol (**23**)

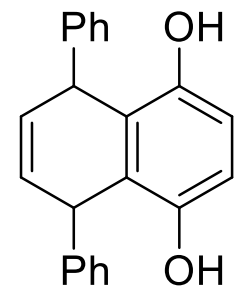

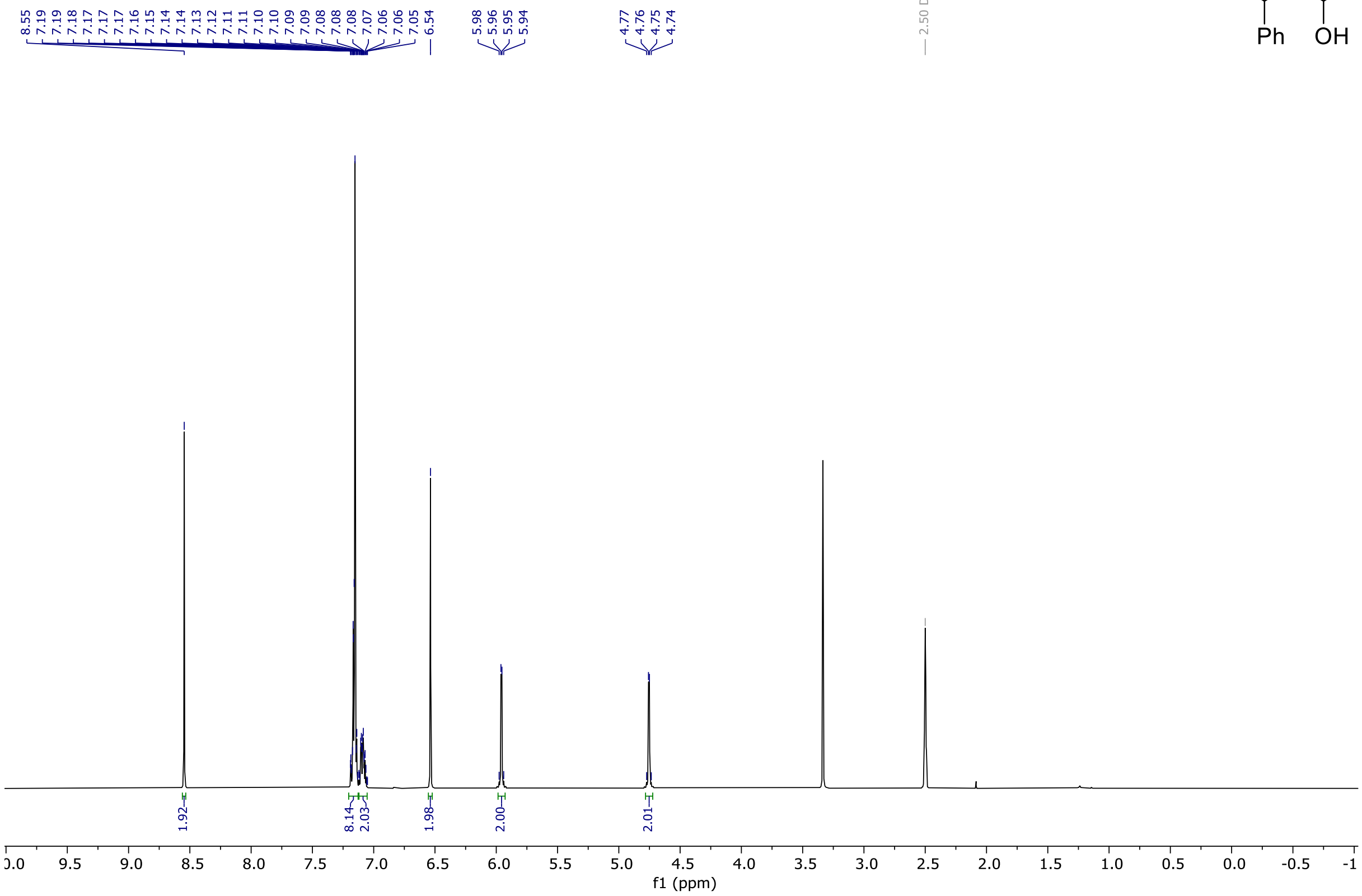

^{1}H NMR spectrum (400 MHz, SO(CD3)2) of **23**.

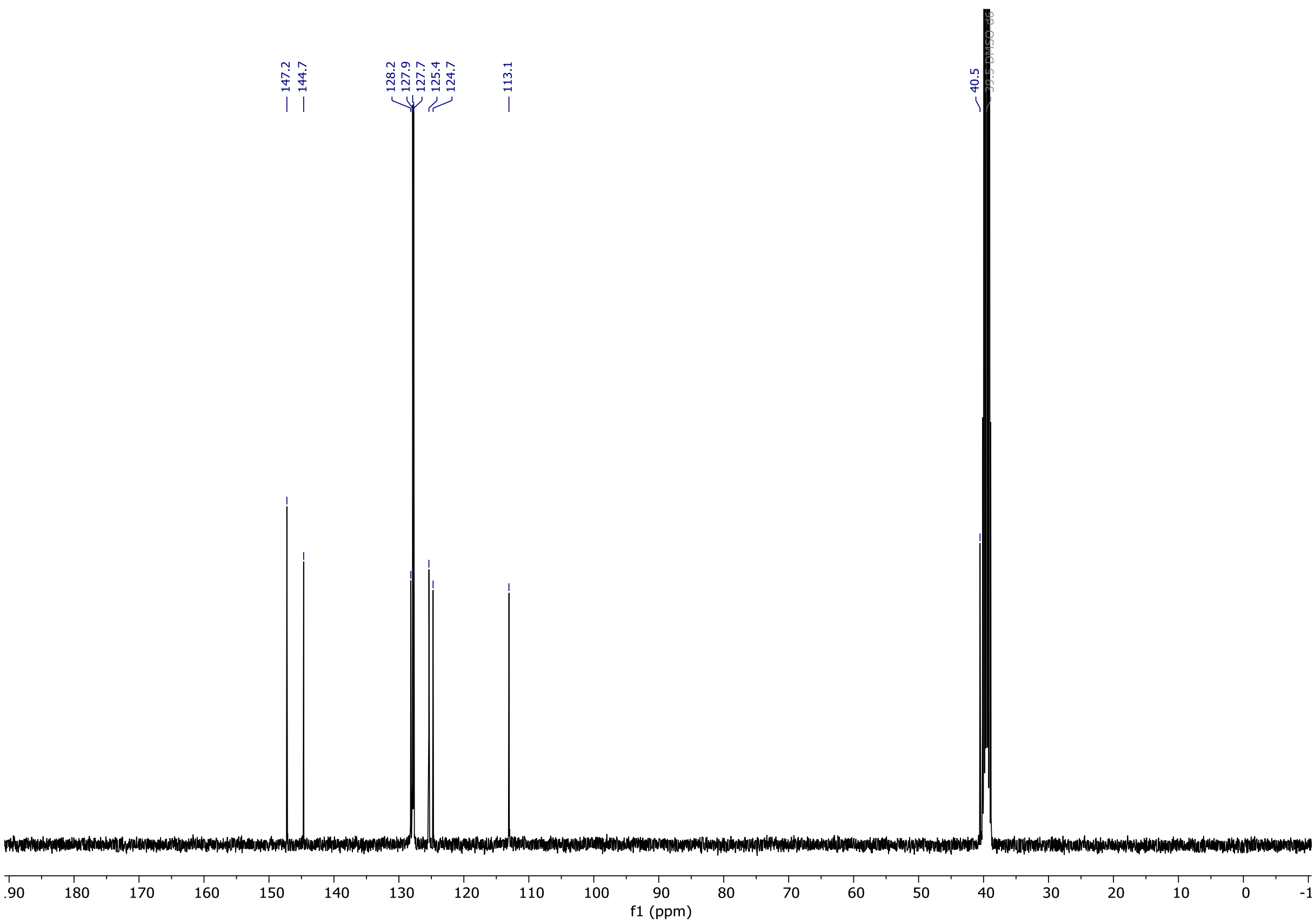

^{13}C NMR spectrum (101 MHz, SO(CD3)2) of **23**.

5,8-Dihydro-1,4-dimethoxy-5,8-diphenylnaphthalene (**24**)

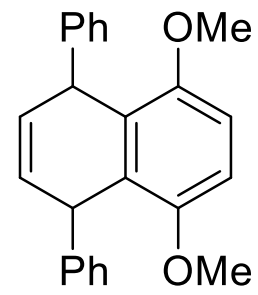

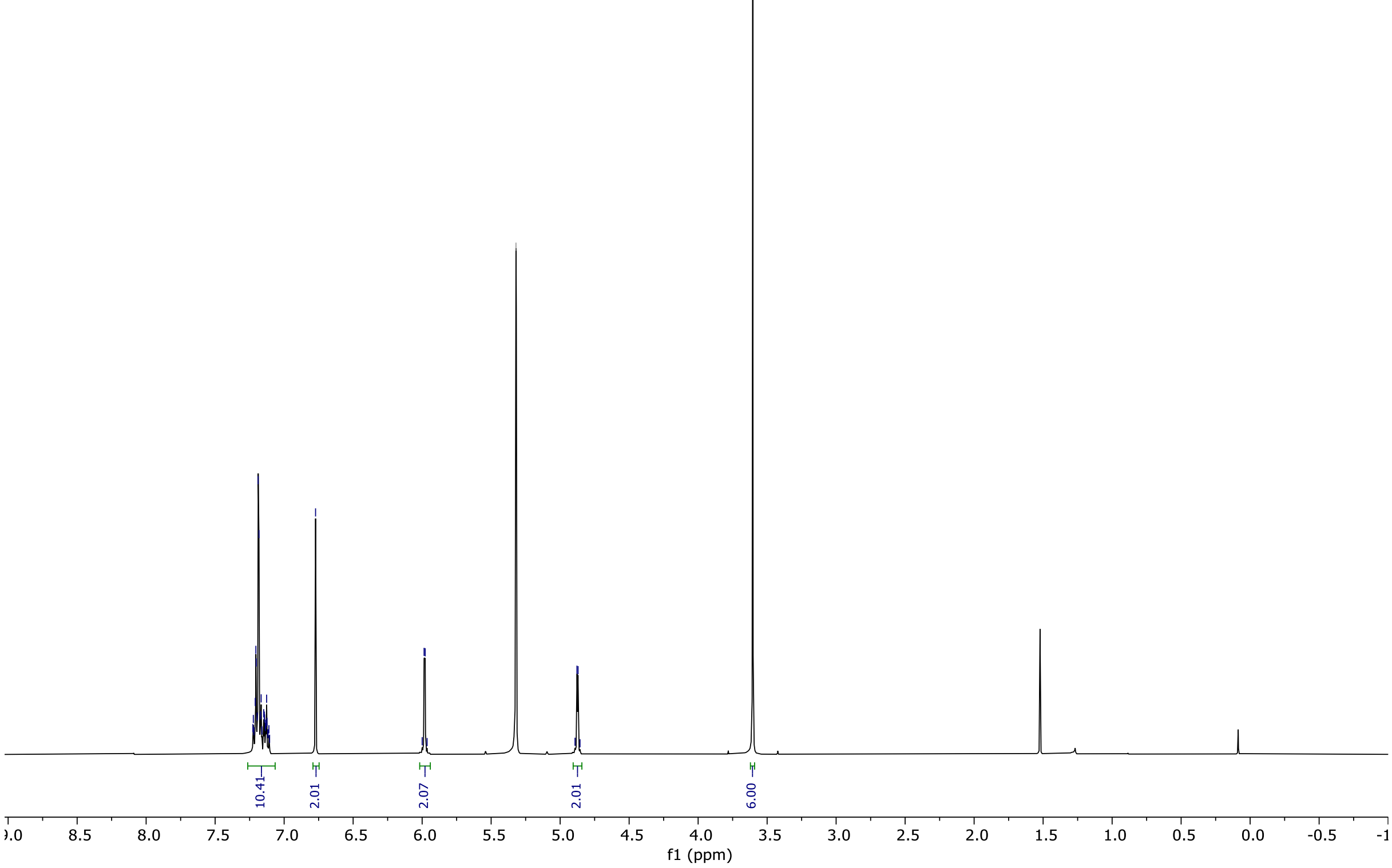

^{1}H NMR spectrum (400 MHz, CD_2Cl_2) of **24**.

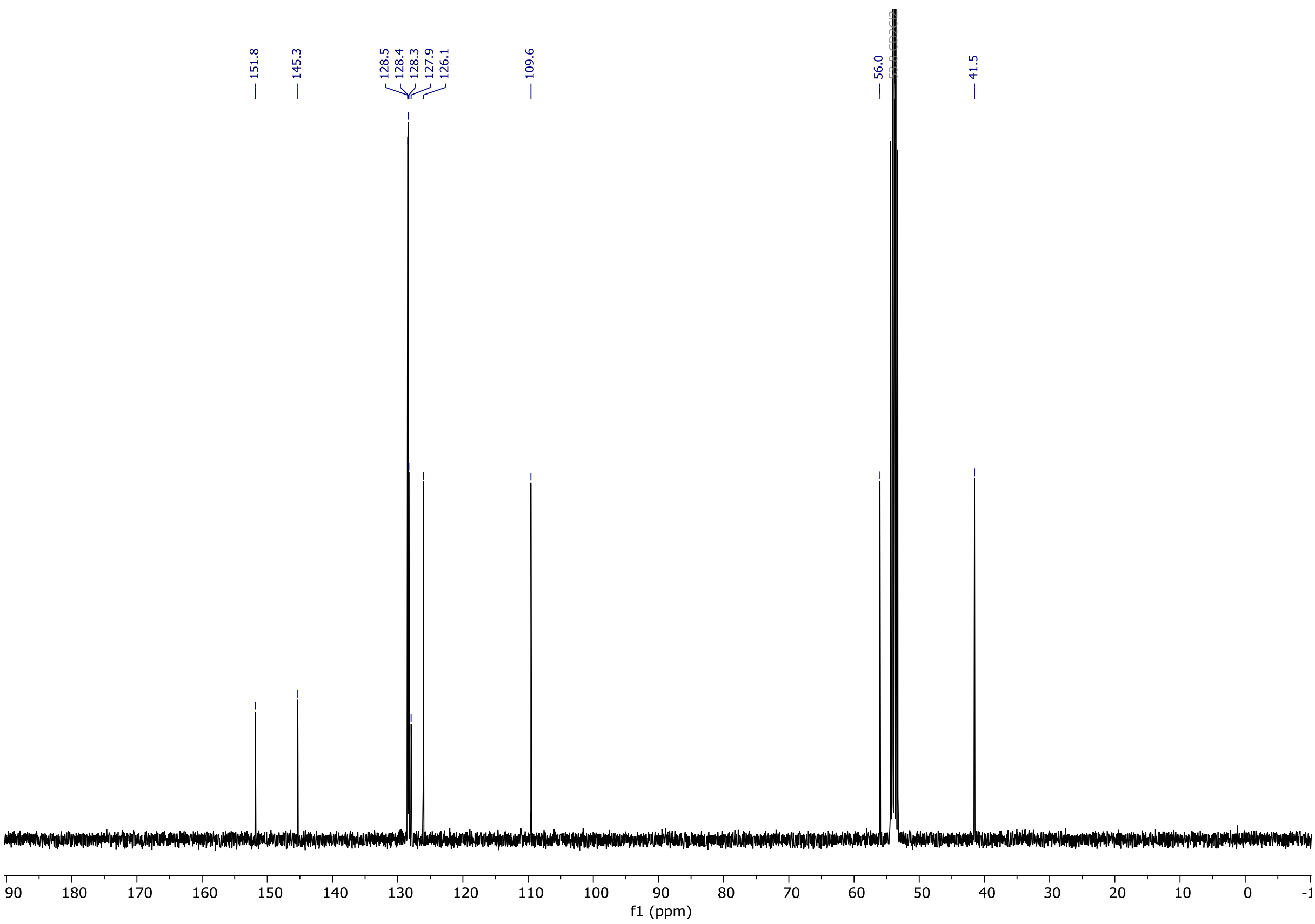

^{13}C NMR spectrum (101 MHz, CD_2Cl_2) of **24**.

1,2,3,4-Tetrahydro-2,3-epoxy-5,8-dimethoxy-1,4-diphenylnaphthalene (**25**)

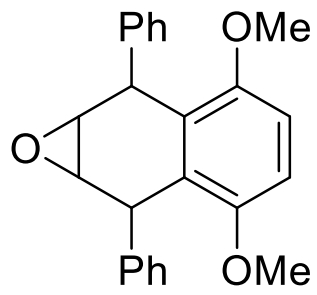

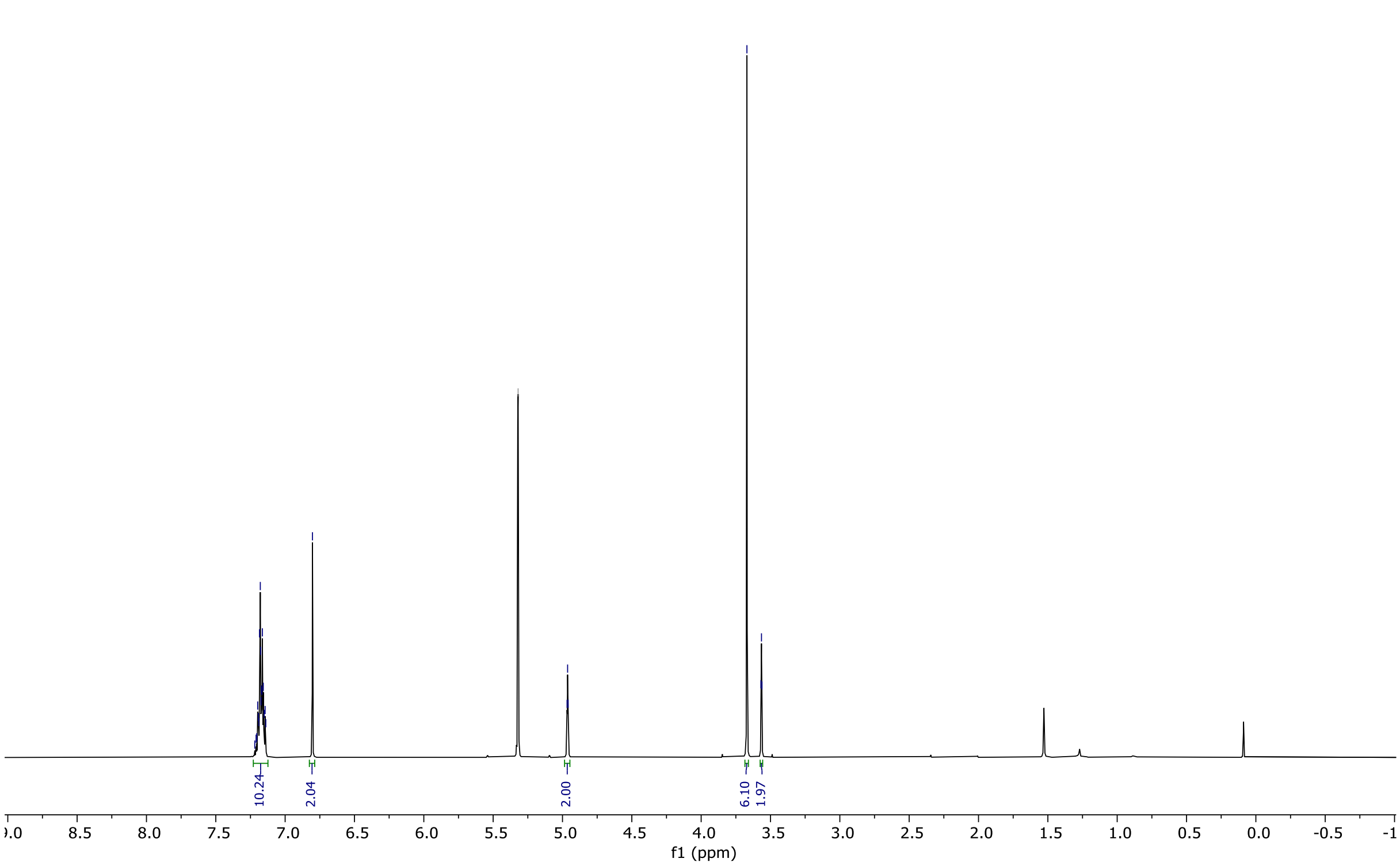

^{1}H NMR spectrum (400 MHz, CD_2Cl_2) of **25**.

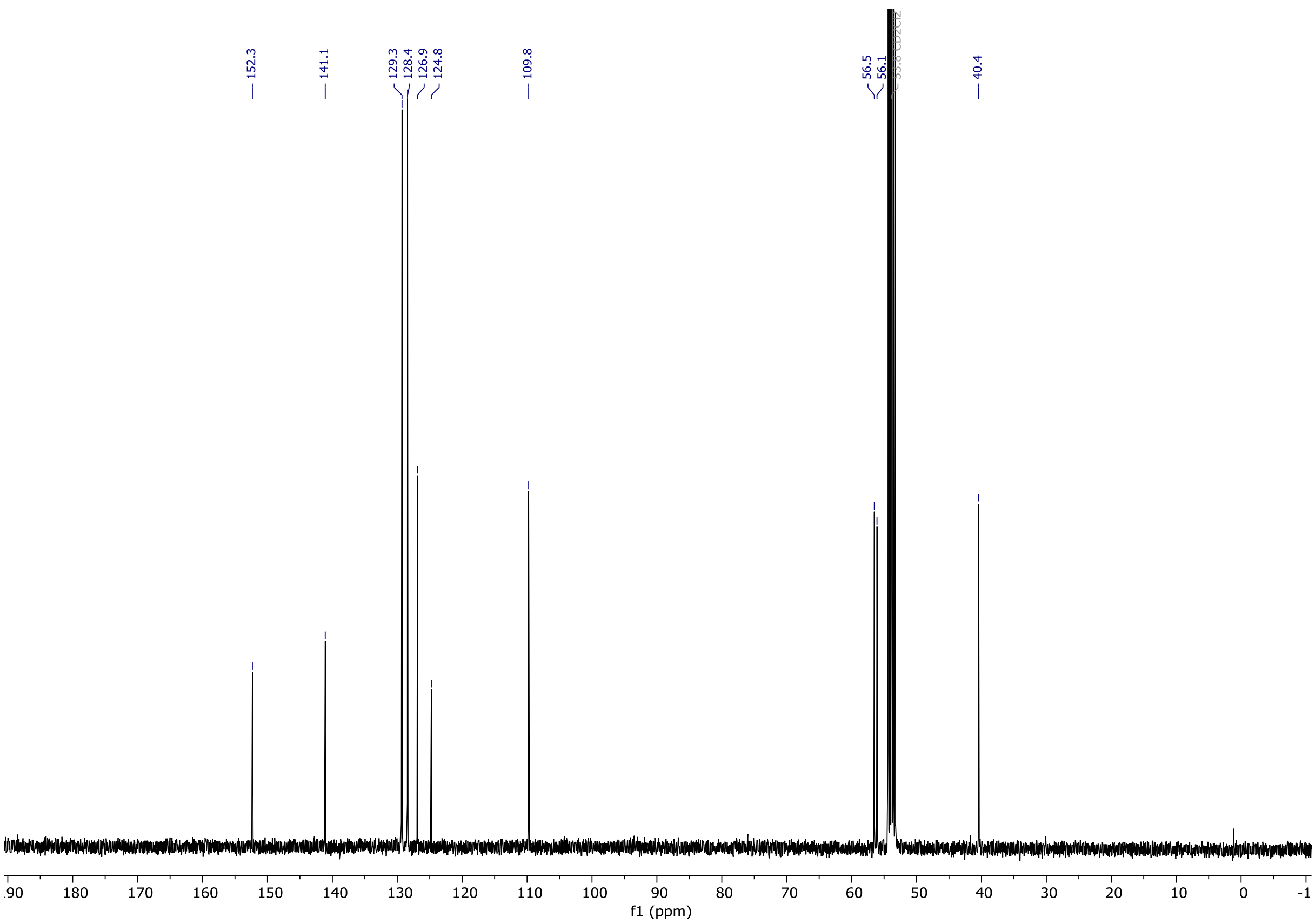

^{13}C NMR spectrum (101 MHz, CD_2Cl_2) of **25**.

P[(2*E*)-3-phenyl-2-propen-1-yl]phosphonate (**S5**)

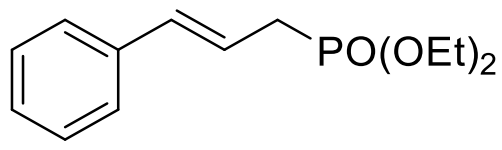

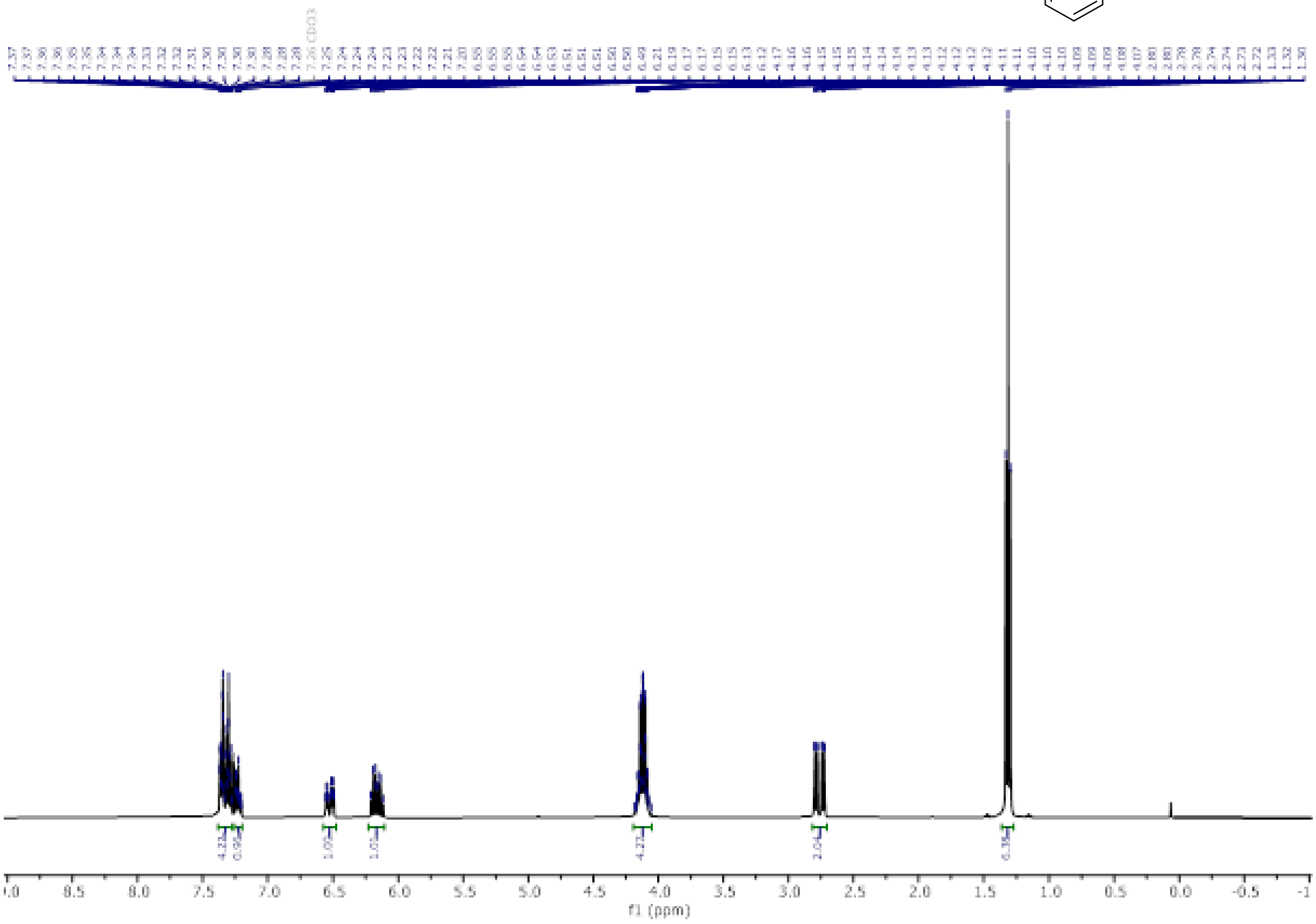

^{1}H NMR spectrum (401 MHz, $CDCl_3$) of **S5**.

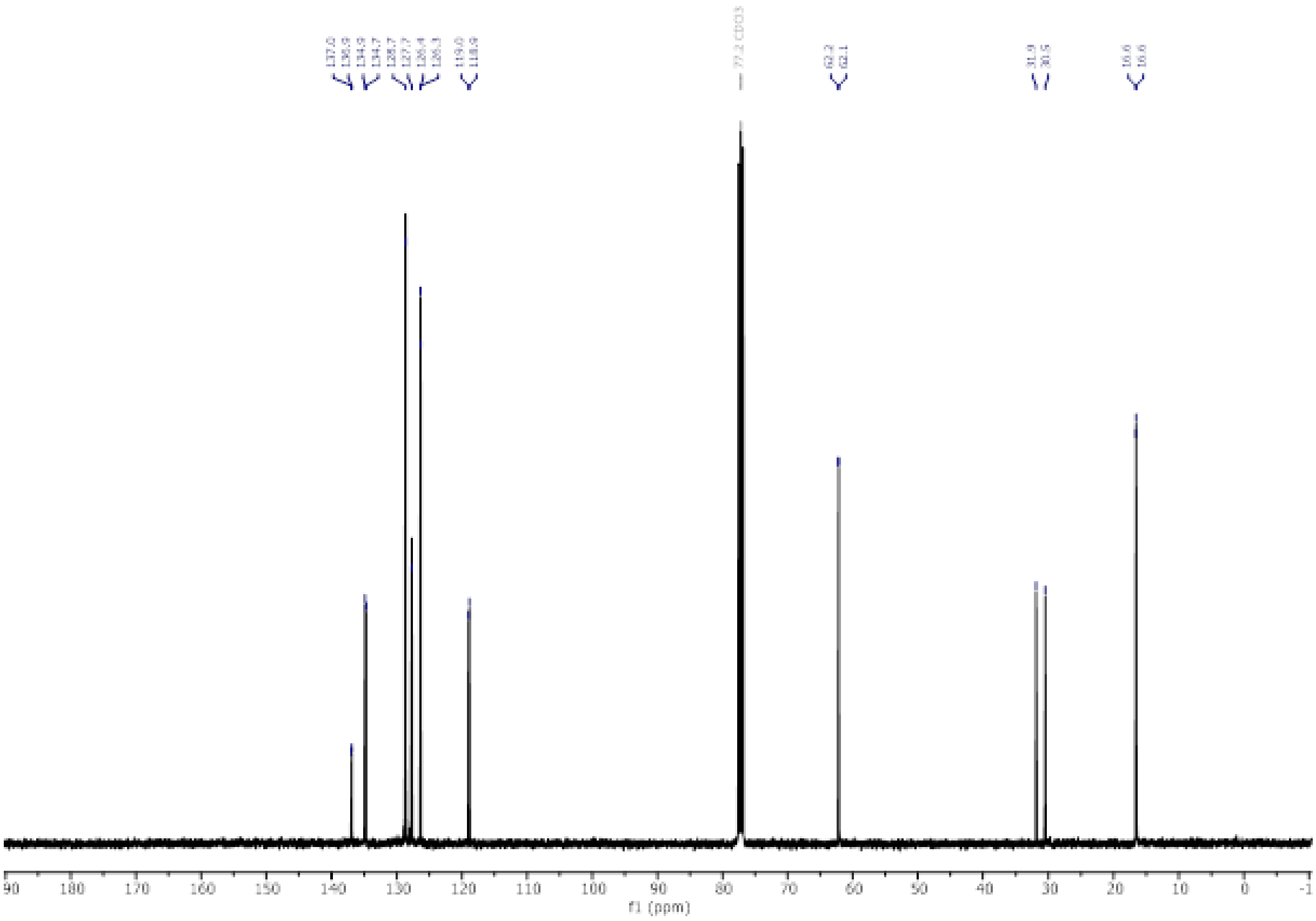

^{13}C NMR spectrum (101 MHz, $CDCl_3$) of **S5**.

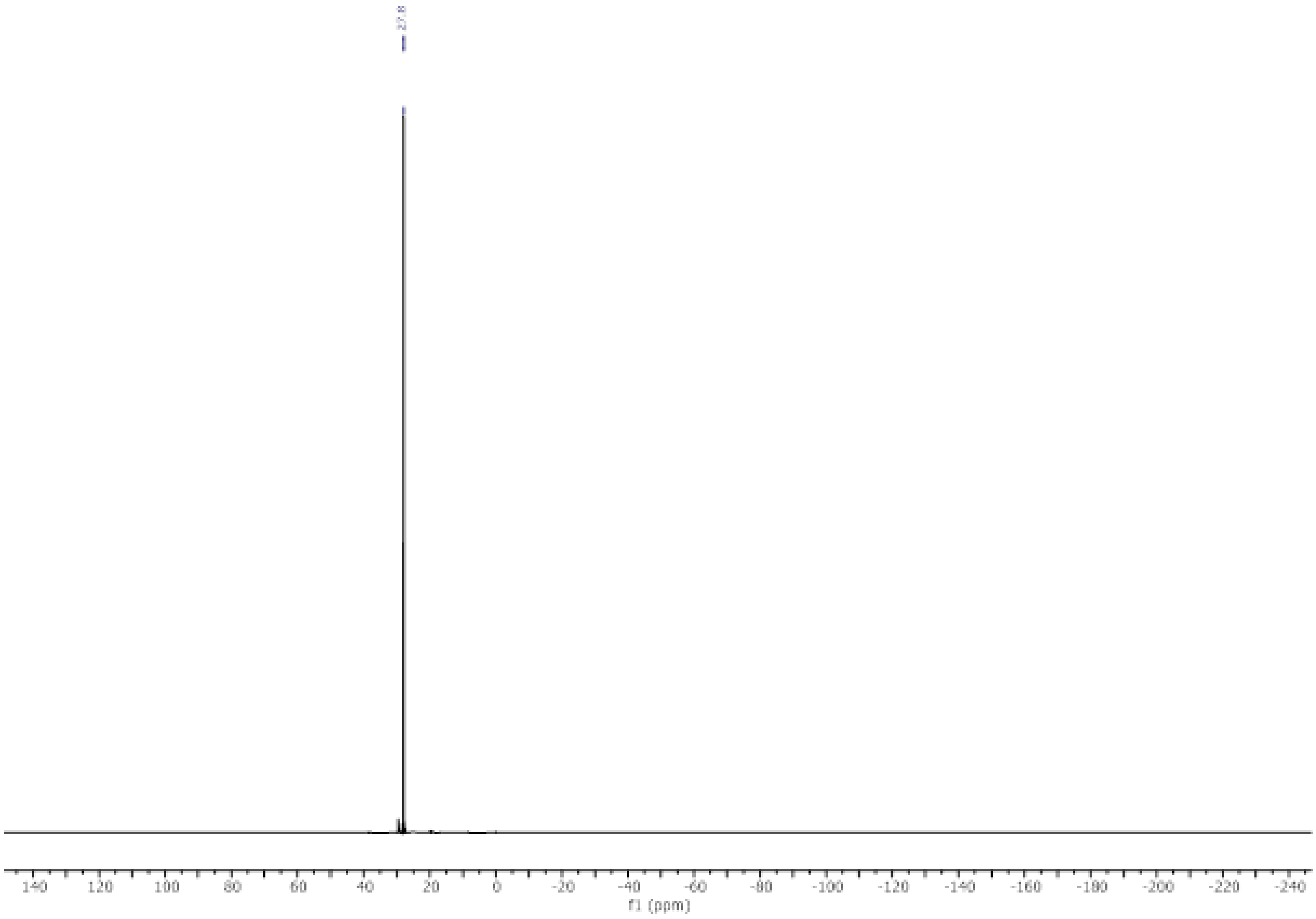

^{31}P NMR spectrum (162 MHz, $CDCl_3$) of **S5**.

Diethyl *P*[(2*E*)-3-(4-methylphenyl)-2-propenyl]phosphonate (**S9**)

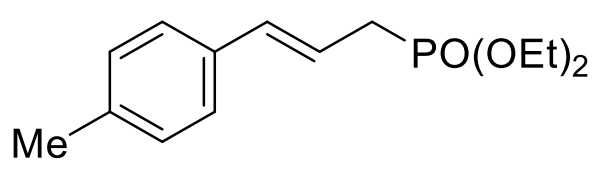

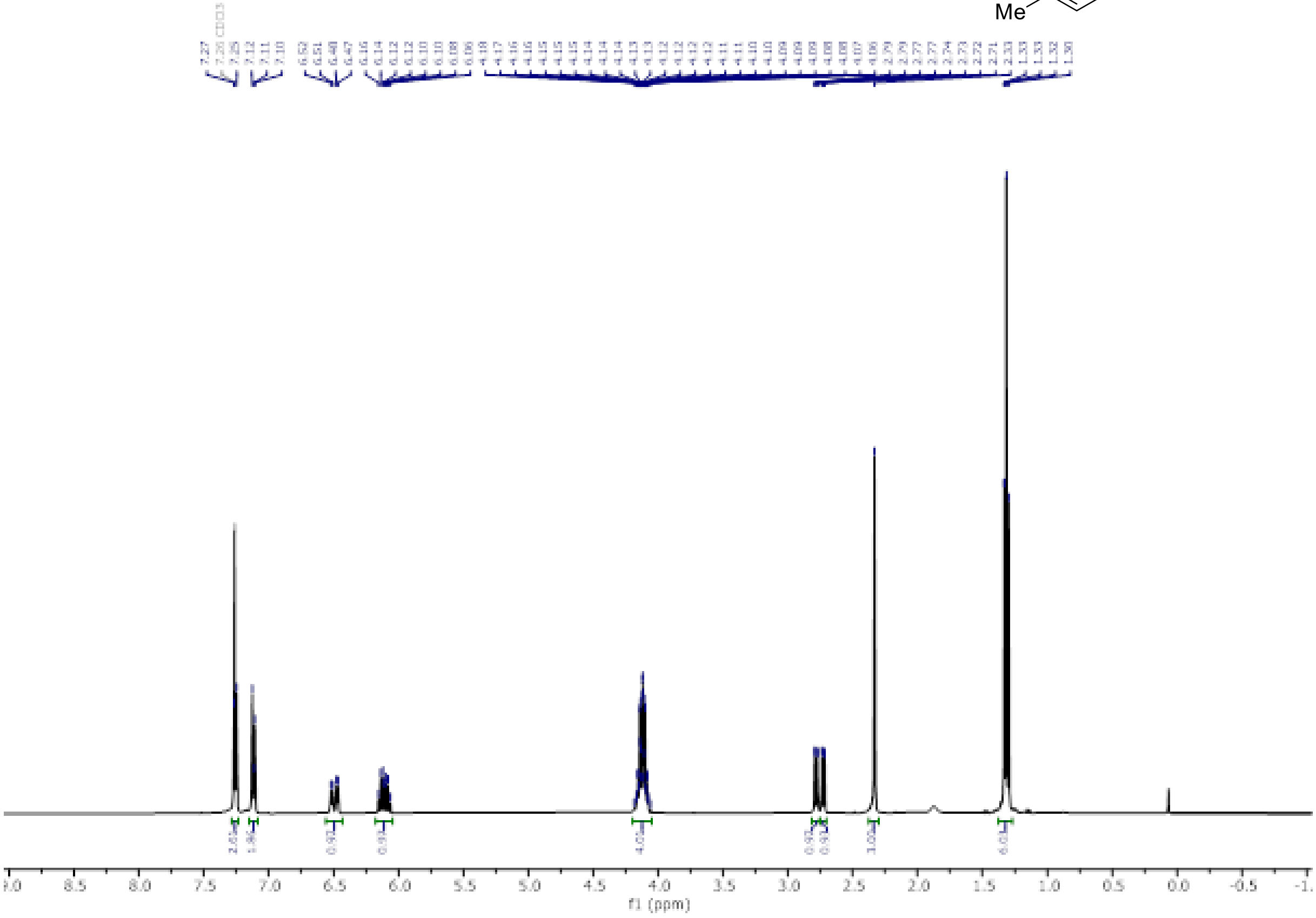

^{1}H NMR spectrum (400 MHz, $CDCl_3$) of **S9**.

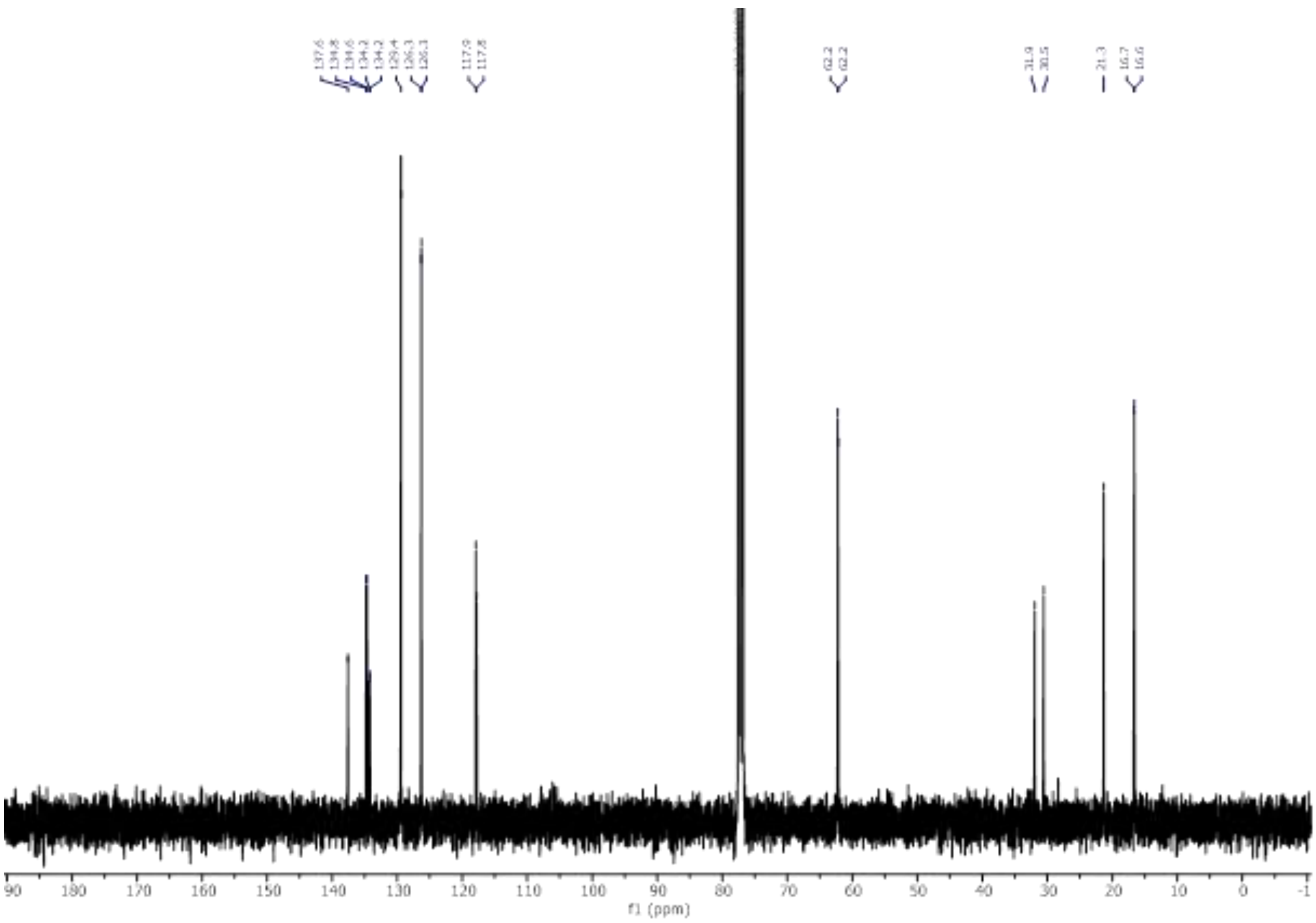

^{13}C NMR spectrum (101 MHz, $CDCl_3$) of **S9**.

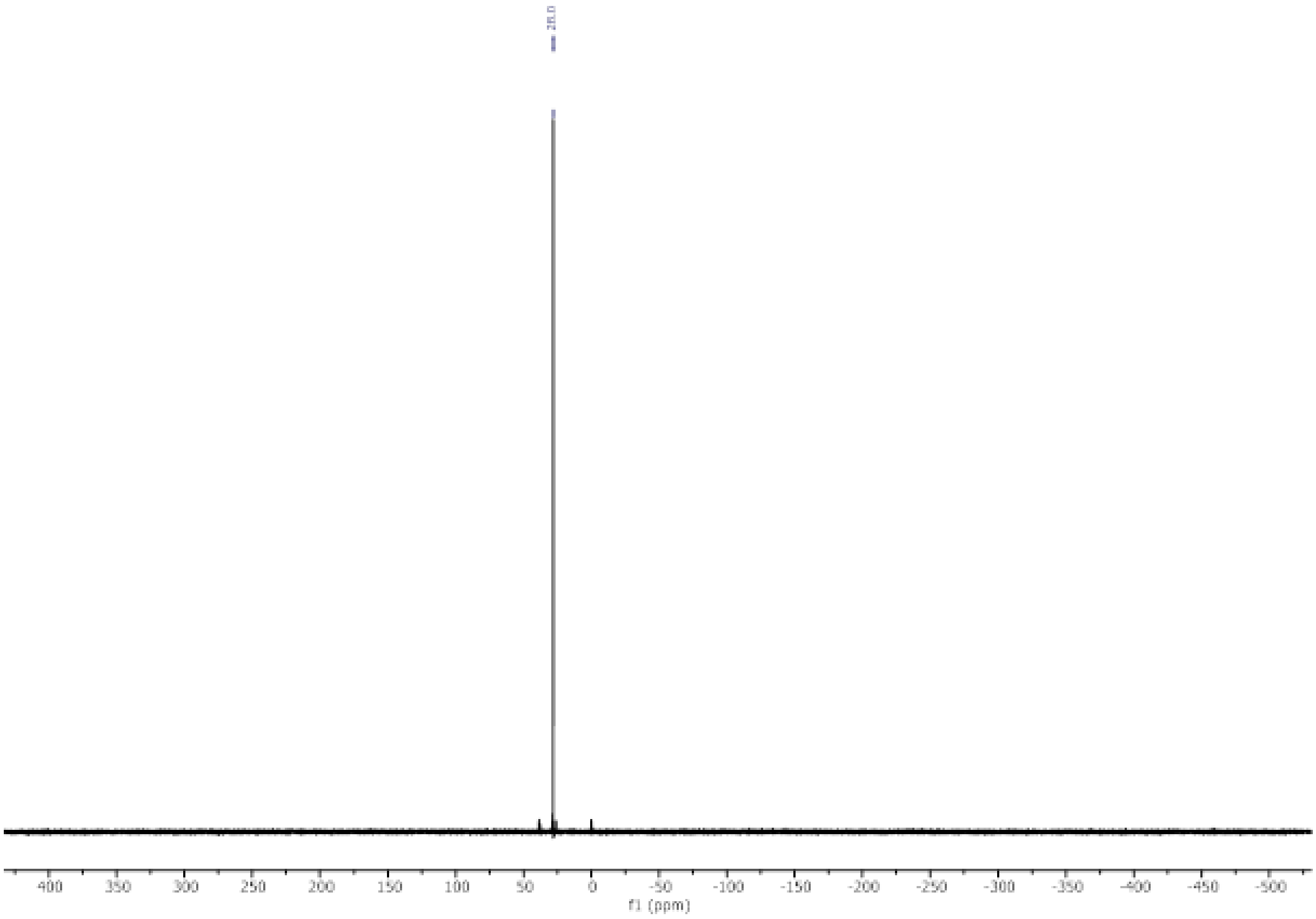

^{31}P NMR spectrum (162 MHz, $CDCl_3$) of **S9**.

Diethyl *P*[(2*E*)-3-(4-bromophenyl)-2-propenyl]phosphonate (**S13**)

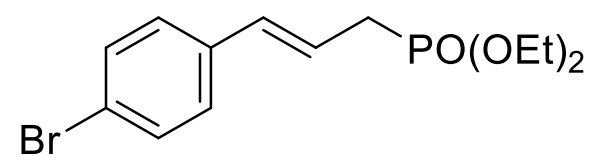

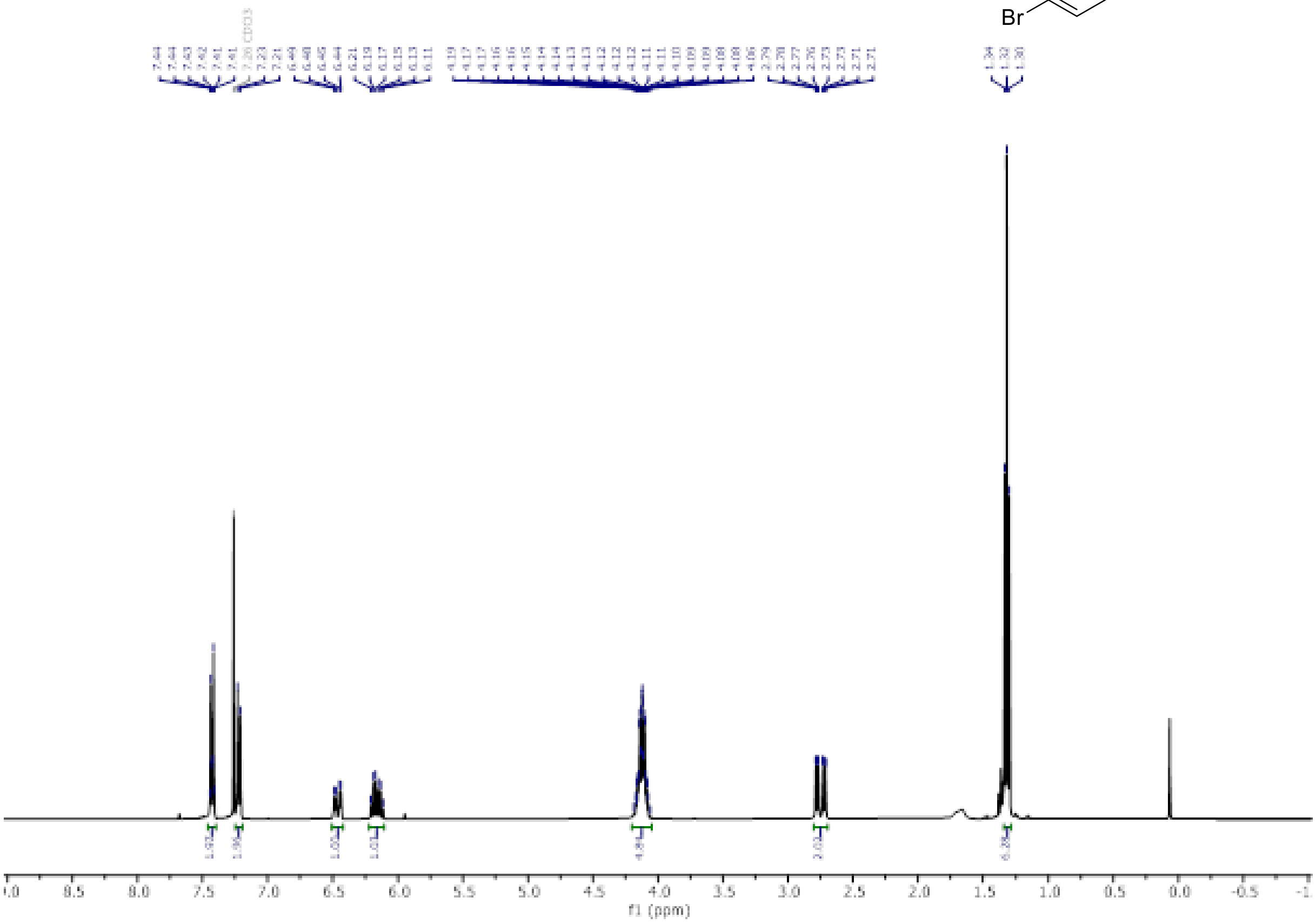

^{1}H NMR spectrum (400 MHz, $CDCl_3$) of **S13**.

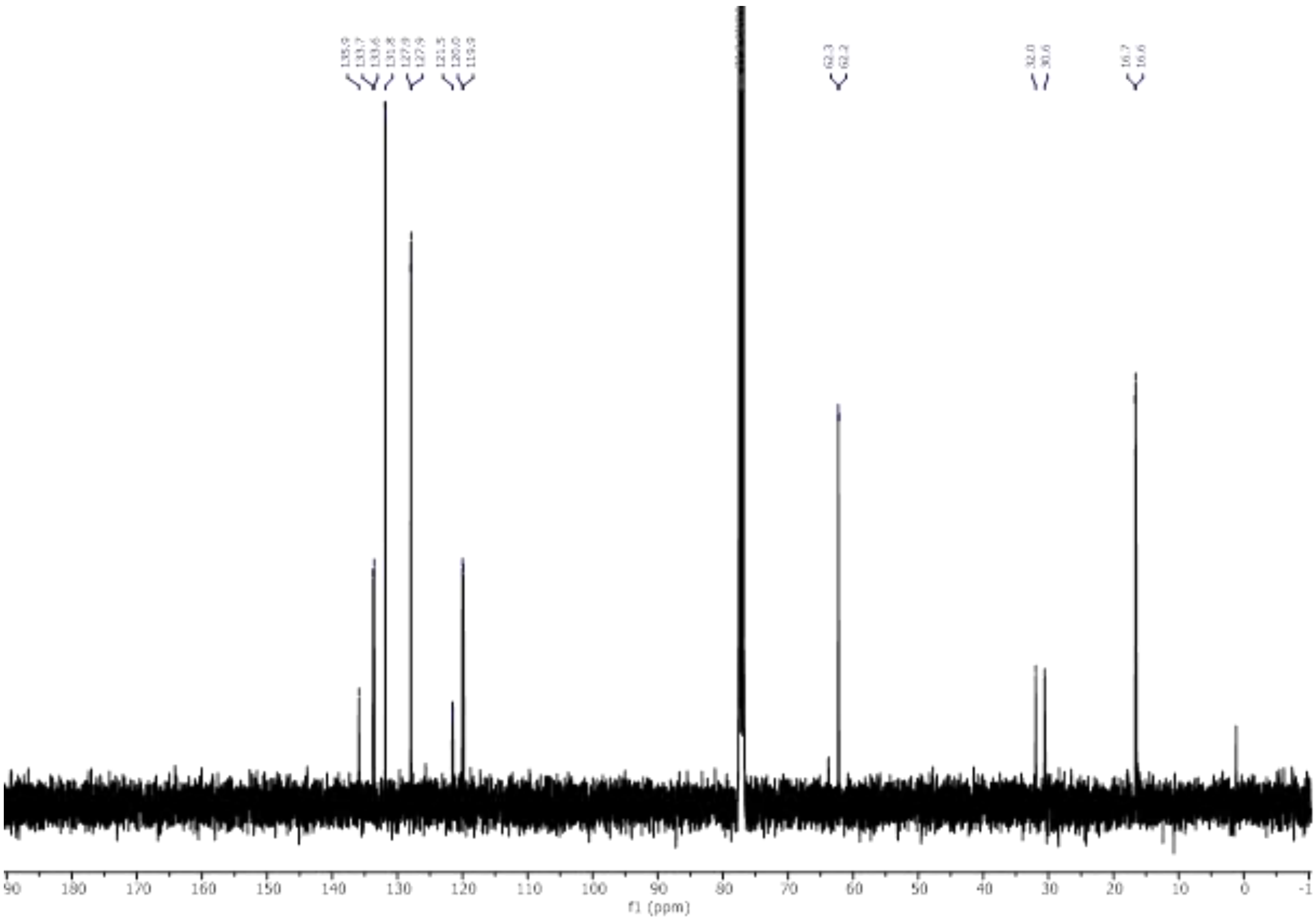

^{13}C NMR spectrum (101 MHz, $CDCl_3$) of **S13**.

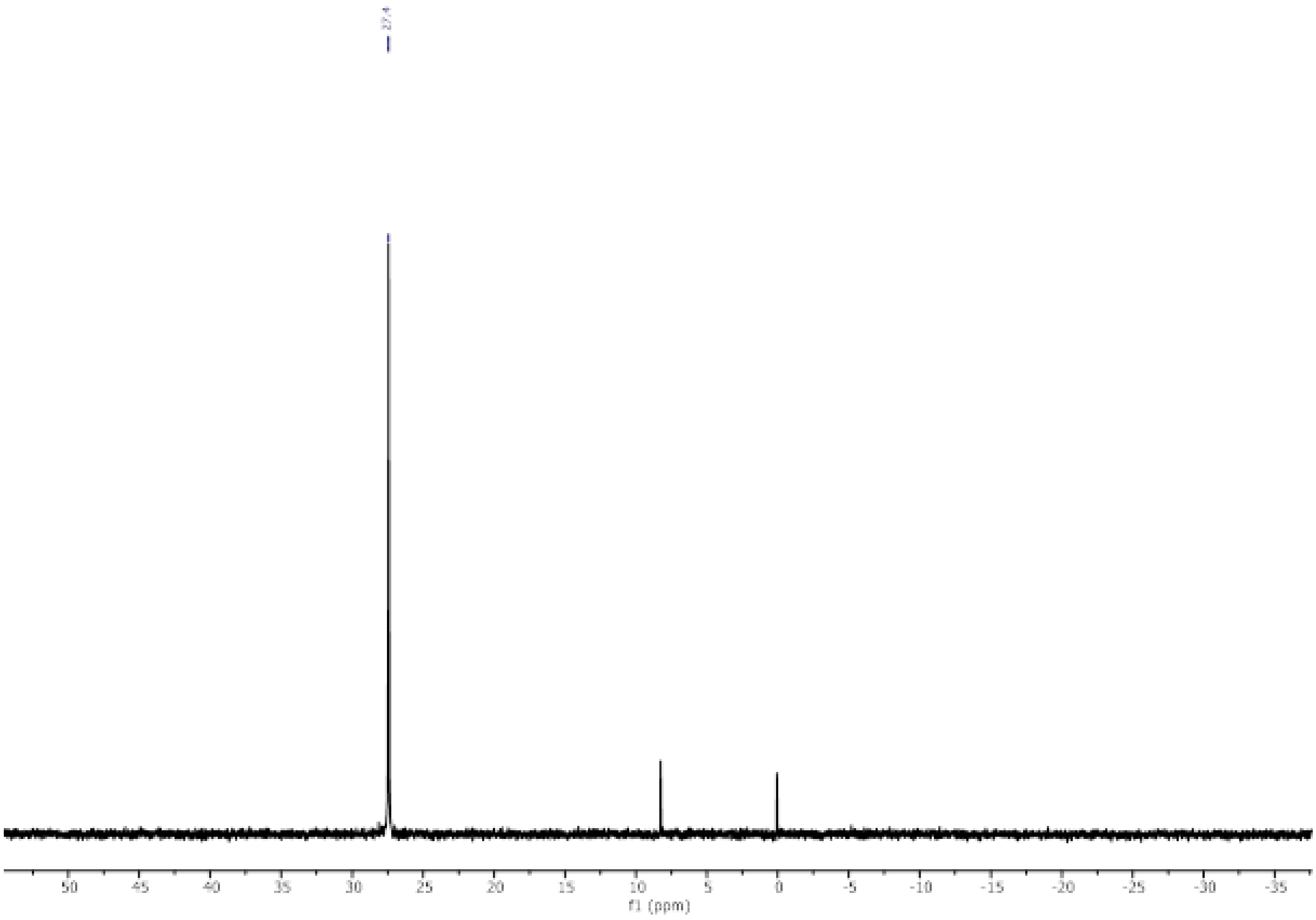

^{31}P NMR spectrum (162 MHz, $CDCl_3$) of **S13**.

Diethyl *P*-2-propen-1-ylphosphonate (**S14**)

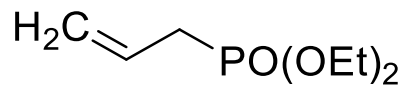

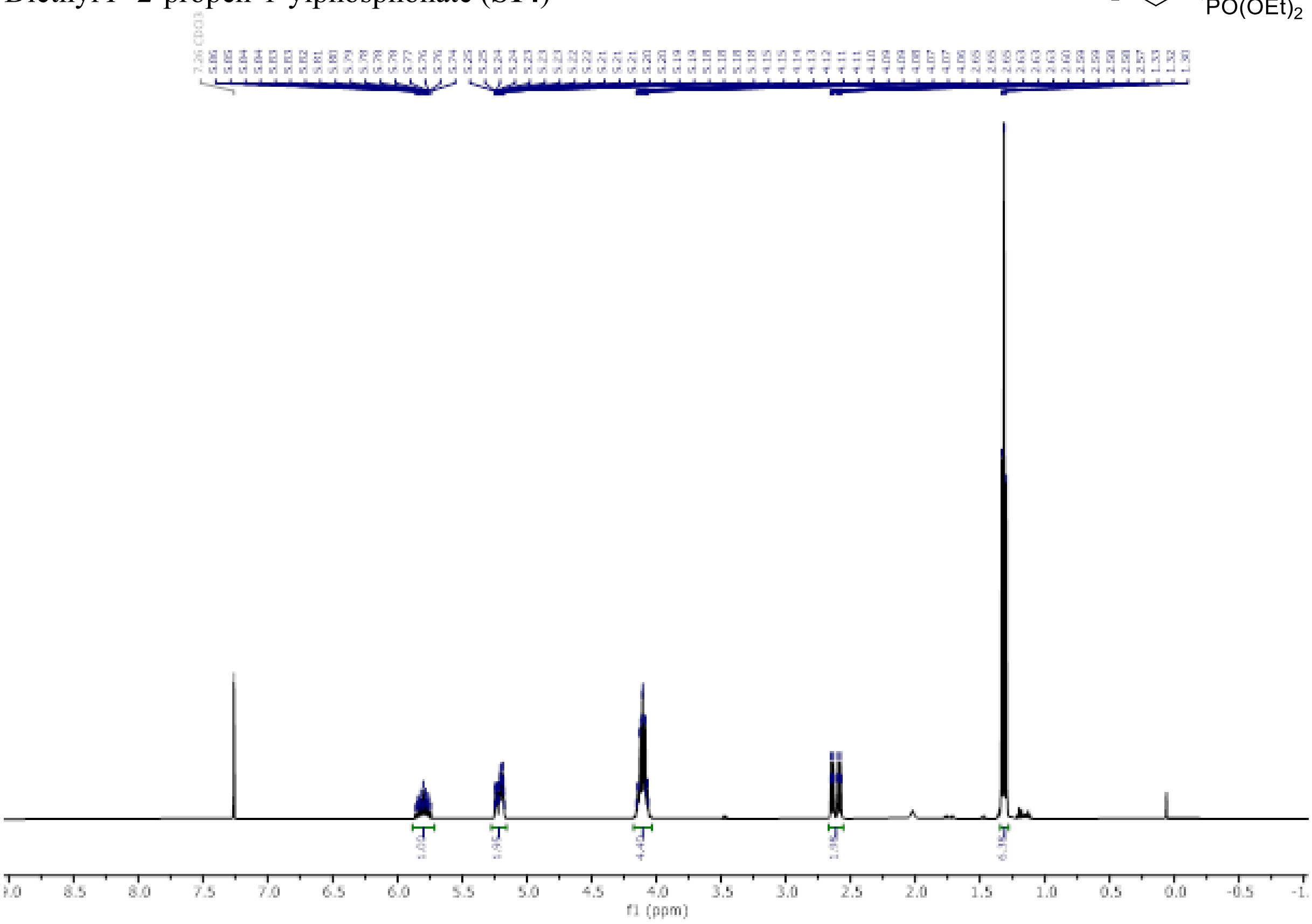

^{1}H NMR spectrum (400 MHz, $CDCl_3$) of **S14**.

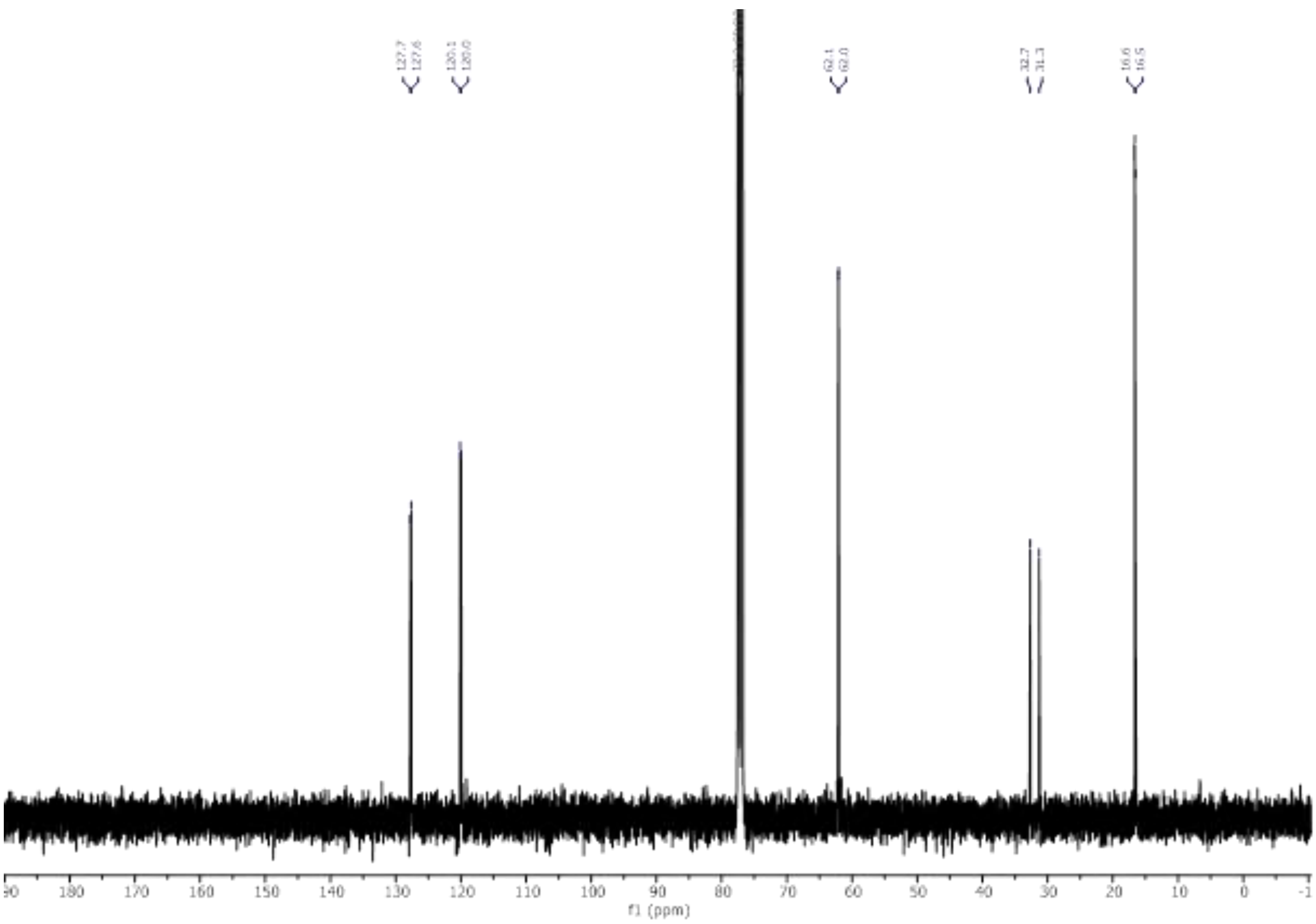

^{13}C NMR spectrum (101 MHz, $CDCl_3$) of **S14**.

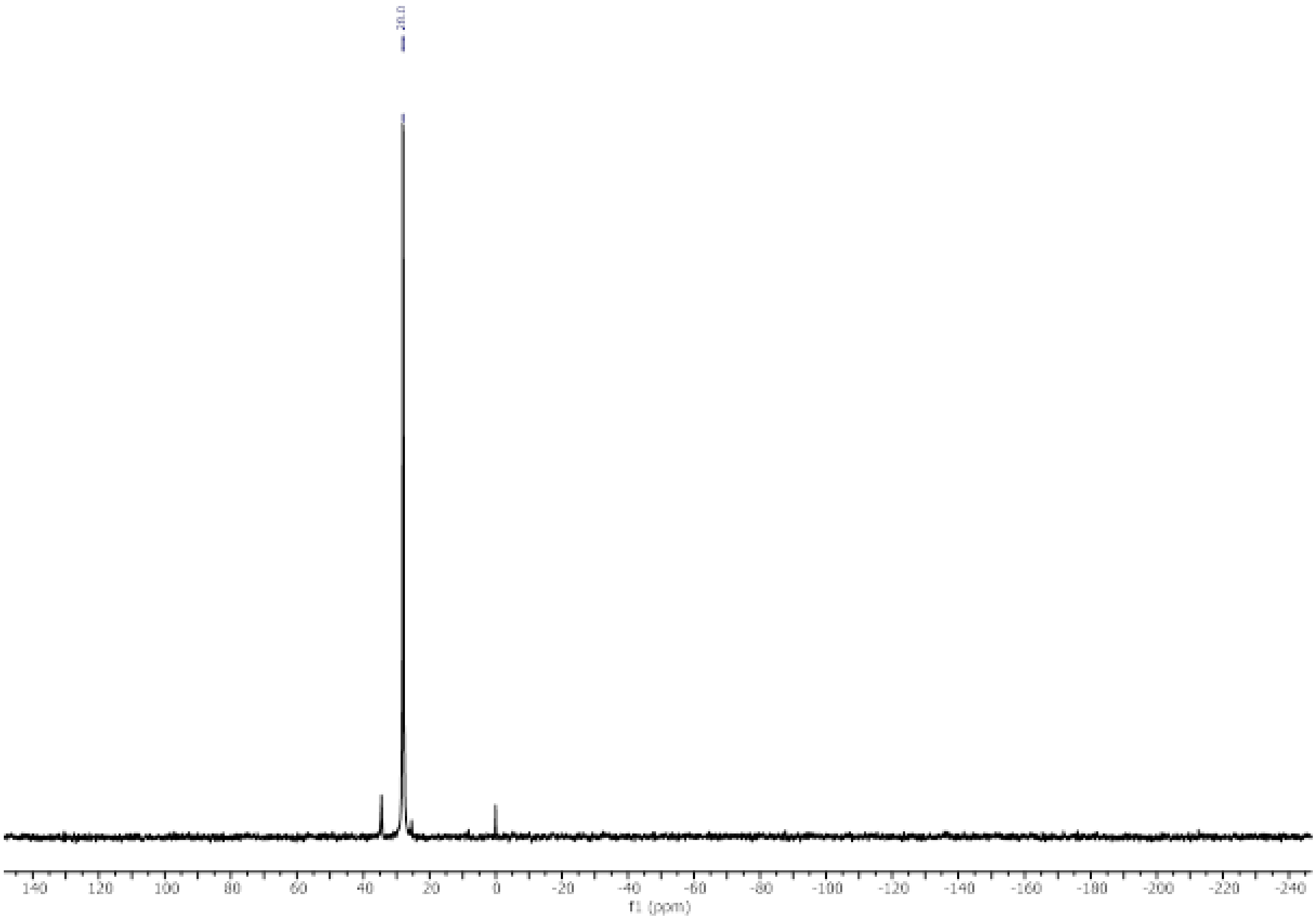

^{31}P NMR spectrum (162 MHz, $CDCl_3$) of **S14**.

5,8-Dihydro-5-ethoxycarbonyl-8-methylnaphthalene-1,4-diol (**S15**)

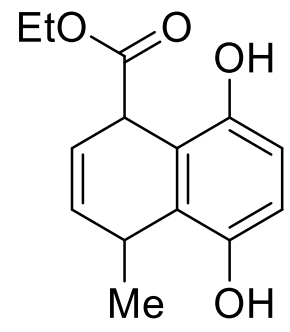

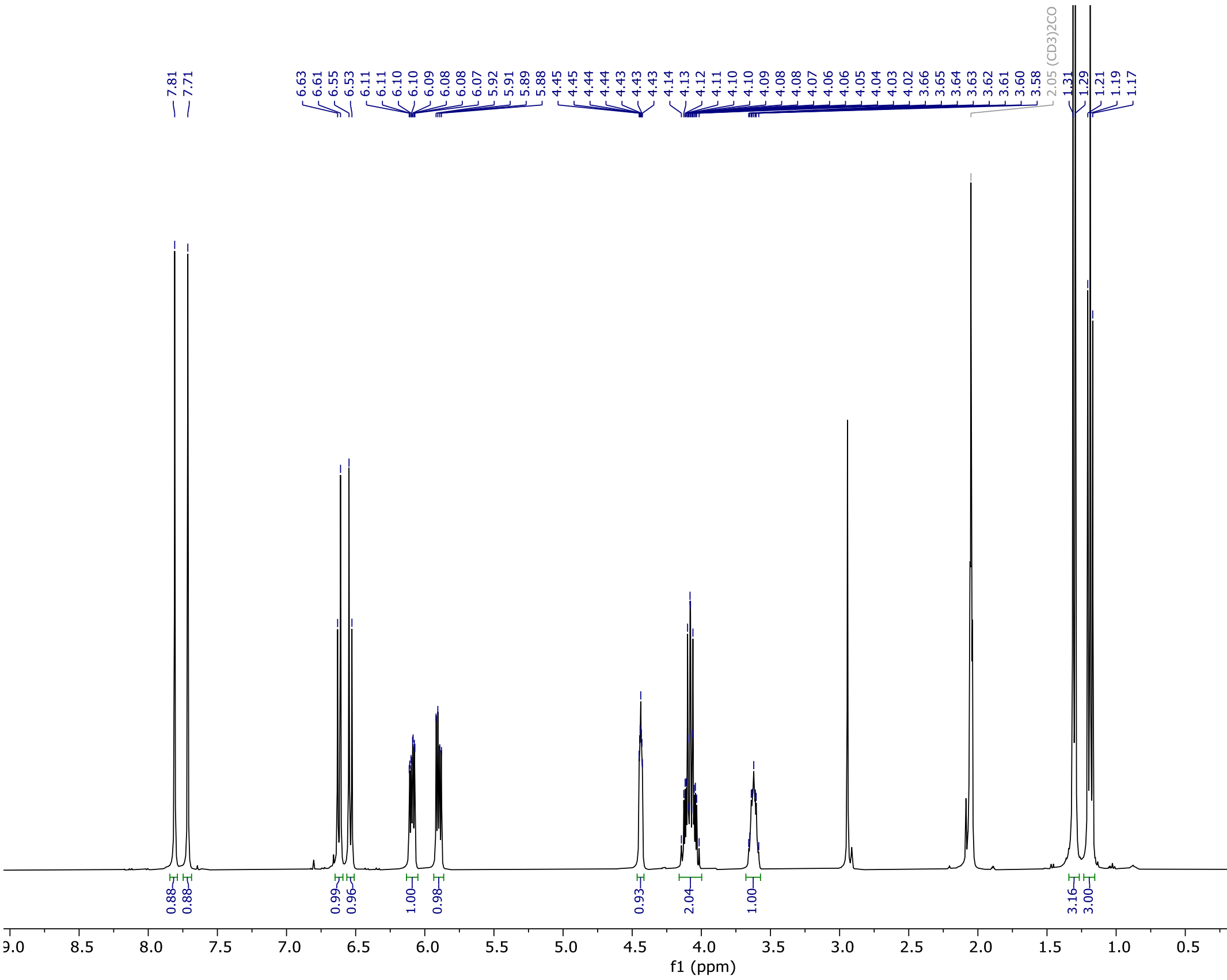

^{1}H NMR spectrum (400 MHz, CO(CH_3)$_2$) of **S15**.

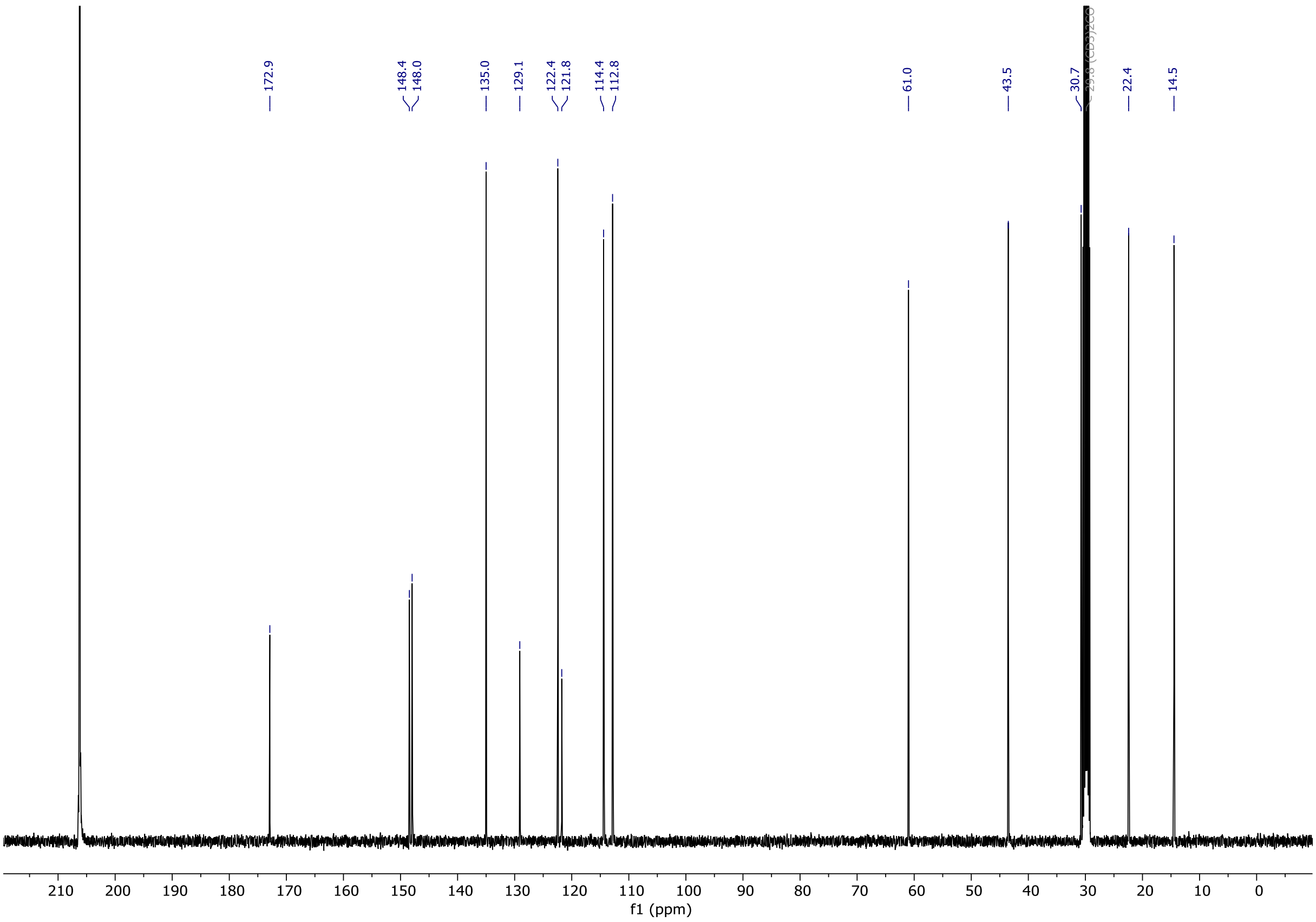

^{13}C NMR spectrum (101 MHz, CO(CH_3)$_2$) of **S15**.

5,8-Dihydro-5-ethoxycarbonyl-8-propylnaphthalene-1,4-diol (**S16**)

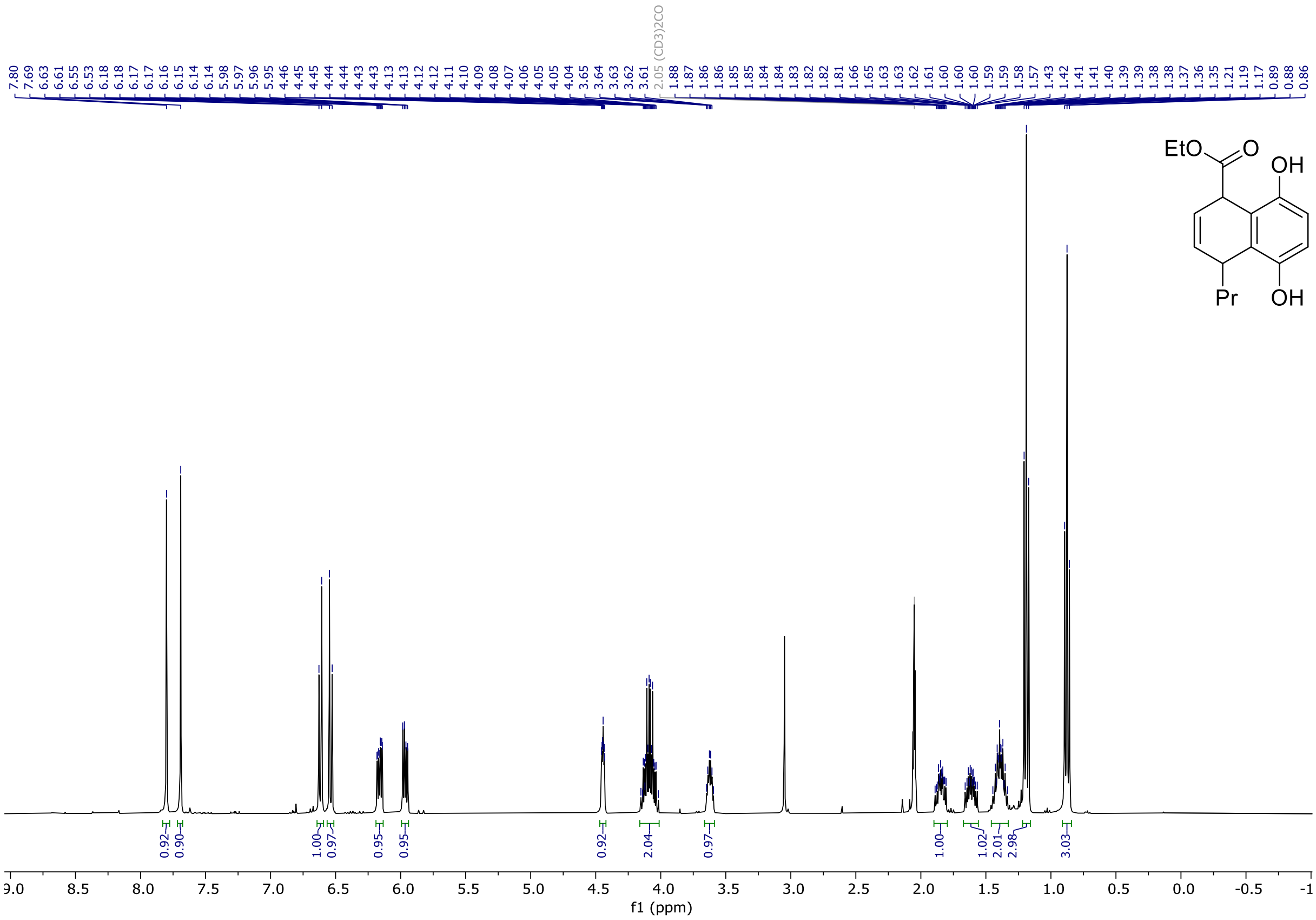

^{1}H NMR spectrum (400 MHz, $CO(CH_3)_2$) of **S16**.

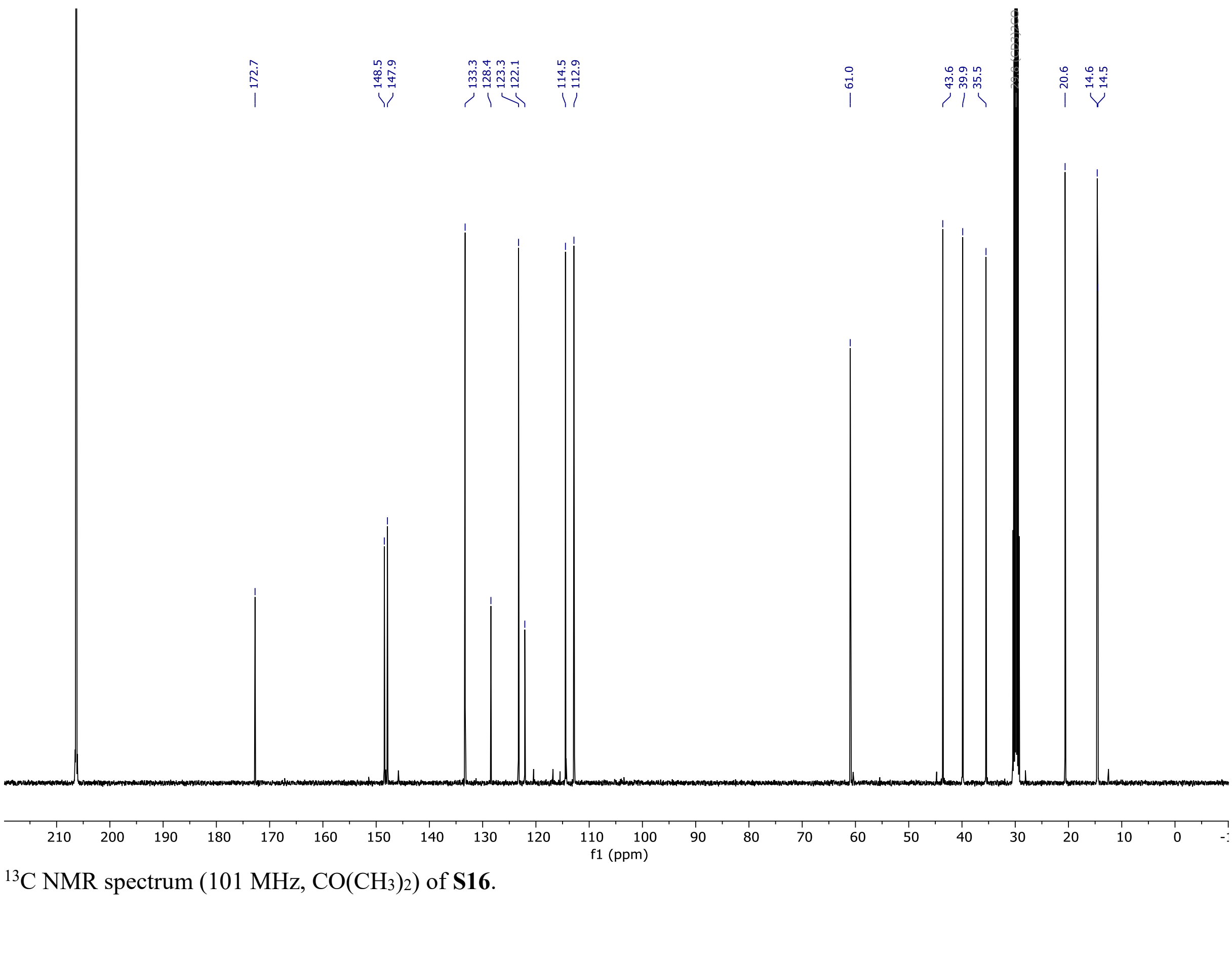

^{13}C NMR spectrum (101 MHz, $CO(CH_3)_2$) of **S16**.

9,10-Dihydro-9,10[1',2']-benzenoanthracene-1,4-diol (**S17**)

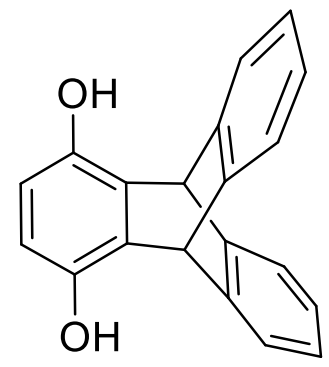

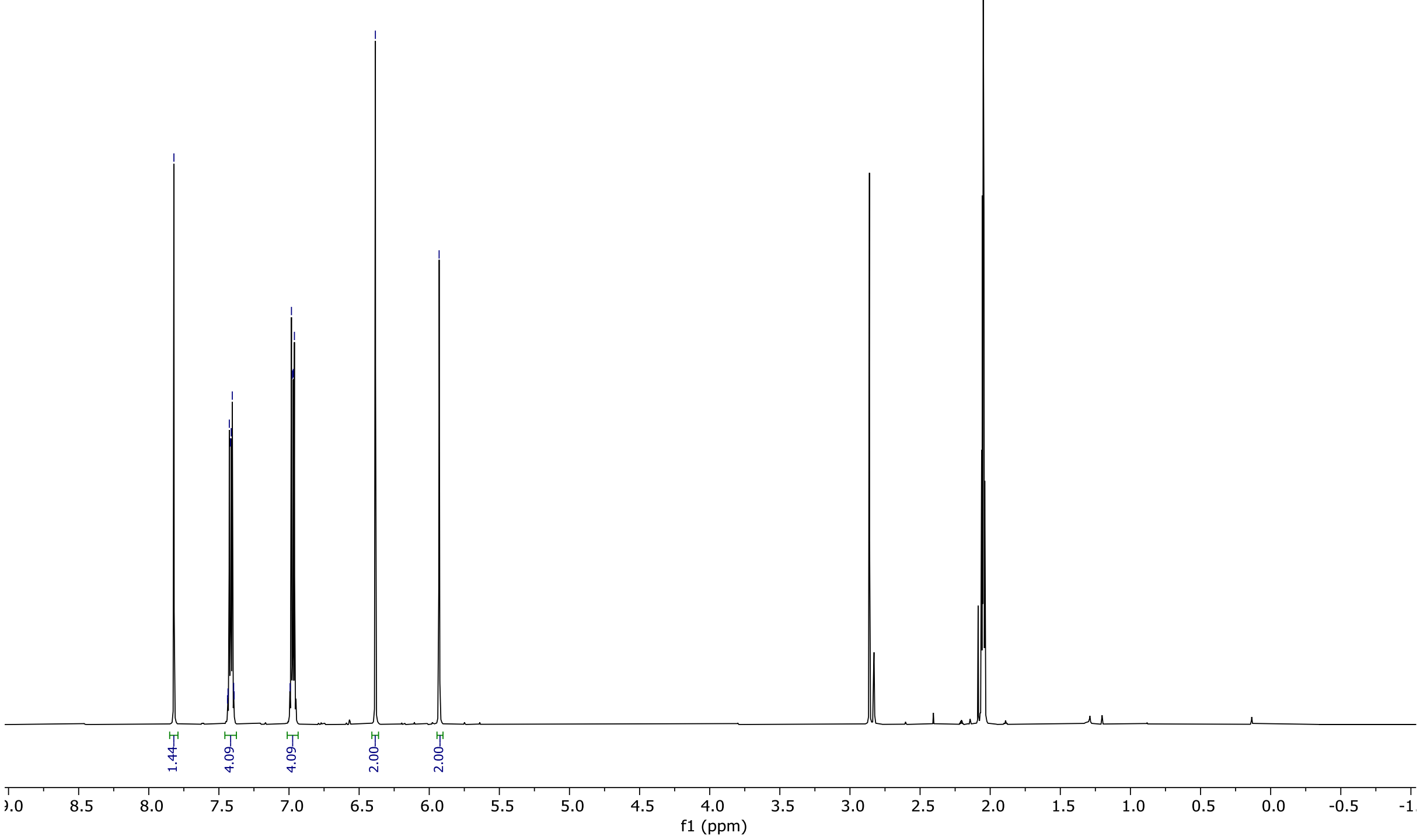

^{1}H NMR spectrum (400 MHz, $CO(CH_3)_2$) of **S17**.

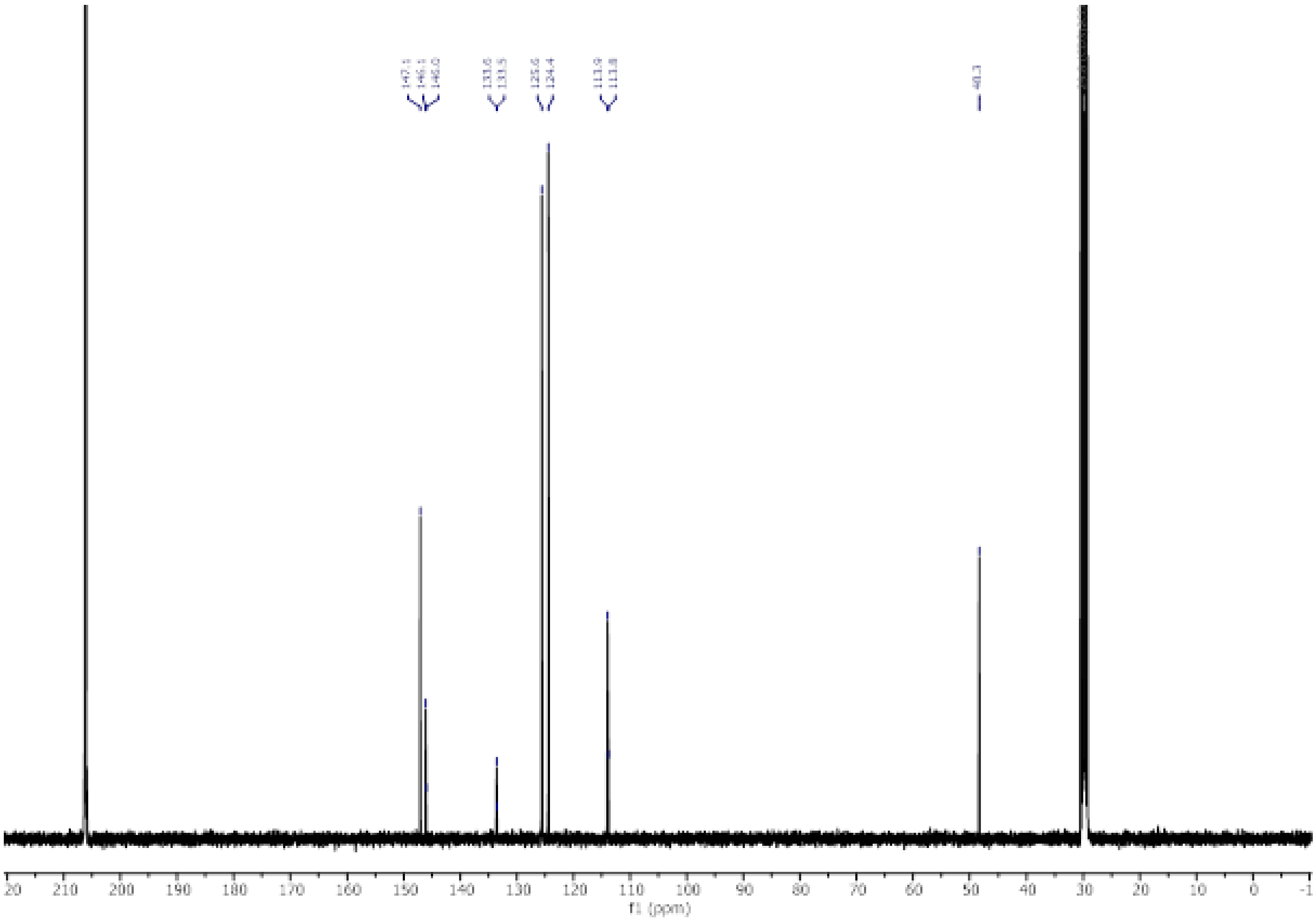

^{13}C NMR spectrum (101 MHz, $CO(CH_3)_2$) of **S17**.

9. References

(1) Vermeeren, P.; Tiezza, M. D.; van Dongen, M.; Fernández, I.; Bickelhaupt, F. M.; Hamlin, T. A. Lewis Acid-Catalyzed Diels-Alder Reactions: Reactivity Trends across the Periodic Table. *Chem. Eur. J.* **2021**, *27* (41), 10610–10620. https://doi.org/10.1002/chem.202100522.

(2) Huang, F.; Huang, Z.; Liu, G.; Huang, Z. Iridium-Catalyzed Selective Trans-Semihydrogenation of 1,3-Enynes with Ethanol: Access to (E,E)-1,4-Diarylbutadienes. *Org. Lett.* **2022**, *24* (29), 5486–5490. https://doi.org/10.1021/acs.orglett.2c02327.

(3) Li, H.; Gonçalves, T. P.; Hu, J.; Zhao, Q.; Gong, D.; Lai, Z.; Wang, Z.; Zheng, J.; Huang, K.-W. A Pseudodearomatized PN3P*Ni–H Complex as a Ligand and σ-Nucleophilic Catalyst. *J. Org. Chem.* **2018**, *83* (24), 14969–14977. https://doi.org/10.1021/acs.joc.8b02205.

(4) Wang, J.; Tong, X.; Xie, X.; Zhang, Z. Rhodium-Catalyzed Activation of C(Sp3)−X (X = Cl, Br) Bond: An Intermolecular Halogen Exchange Case. *Org. Lett.* **2010**, *12* (23), 5370–5373. https://doi.org/10.1021/ol101995v.

(5) Chakrabarty, S.; Palencia, H.; Morton, M. D.; Carr, R. O.; Takacs, J. M. Facile Access to Functionalized Chiral Secondary Benzylic Boronic Esters via Catalytic Asymmetric Hydroboration. *Chem. Sci.* **2019**, *10* (18), 4854–4861. https://doi.org/10.1039/C8SC05613G.

(6) Zhang, Z.; Zhang, D.; Zhu, L.; Zeng, D.; Kambe, N.; Qiu, R. Pd-Catalyzed Cross-Coupling of Organostibines with Styrenes to Give Unsymmetric (E)-Stilbenes and (1E,3E)-1,4-Diarylbuta-1,3-Dienes and Fluorescence Properties of the Products. *Org. Lett.* **2021**, *23* (14), 5317–5322. https://doi.org/10.1021/acs.orglett.1c01532.

(7) Watanabe, H.; Sato, T.; Sumita, M.; Shiroyama, M.; Sugawara, D.; Tokuyama, T.; Okuda, Y.; Wakamatsu, K.; Orita, A. Effective Synthesis of 1,4-Diarylbutadienes via Reductive Desulfonylation of 1,3-Butadienyl Sulfones by Proper Choice of Regioisomeric π-Expanded Pyrene Photocatalysts. *Bull. Chem. Soc. Jpn.* **2024**, *97* (2), uoad013. https://doi.org/10.1093/bulcsj/uoad013.

(8) Sahoo, S.; Mandal, A.; Patra, A.; Adak, L. Palladium-Catalyzed Decarboxylative Cross-Coupling of 2,4-Dienoic Acids and Aryl Halides: Access to Fluorescence Active (E,E)-1,4-Diarylbutadienes. *J. Org. Chem.* **2025**, *90* (40), 14027–14038. https://doi.org/10.1021/acs.joc.5c00782.

(9) Yamamoto, Y.; Kawaguchi, S.; Nishimura, M.; Sato, Y.; Shimada, Y.; Tabuchi, A.; Nomoto, A.; Ogawa, A. Phosphorus-Recycling Wittig Reaction: Design and Facile Synthesis of a Fluorous Phosphine and Its Reusable Process in the Wittig Reaction. *J. Org. Chem.* **2020**, *85* (22), 14684–14696. https://doi.org/10.1021/acs.joc.0c01926.

(10) Wang, Y.; McGonigal, P. R.; Herlé, B.; Besora, M.; Echavarren, A. M. Gold(I) Carbenes by Retro-Buchner Reaction: Generation and Fate. *J. Am. Chem. Soc.* **2014**, *136* (2), 801–809. https://doi.org/10.1021/ja411626v.

(11) Zhang, T.; Wang, R.; Chen, J.; Liu, L.; Huang, T.; Li, C.; Tang, Z.; Chen, T. Base-Promoted Direct E-Selective Olefination of Organoammonium Salts with Sulfones toward Stilbenes and Conjugated 1,3-Dienes. *Org. Biomol. Chem.* **2022**, *20* (21), 4369–4375. https://doi.org/10.1039/D2OB00716A.

(12) Shintani, R.; Duan, W.-L.; Park, S.; Hayashi, T. Rhodium-Catalyzed Isomerization of Unactivated Alkynes to 1,3-Dienes. *Chem. Commun.* **2006**, No. 34, 3646–3647. https://doi.org/10.1039/B605368H.

(13) Dong, D.-J.; Li, H.-H.; Tian, S.-K. A Highly Tunable Stereoselective Olefination of Semistabilized Triphenylphosphonium Ylides with N-Sulfonyl Imines. *J. Am. Chem. Soc.* **2010**, *132* (14), 5018–5020. https://doi.org/10.1021/ja910238f.

(14) Song, C.; Yang, C.; Zeng, H.; Zhang, W.; Guo, S.; Zhu, J. Rh(III)-Catalyzed Enaminone-Directed C–H Coupling with α-Diazo-α-Phosphonoacetate for Reactivity Discovery: Fluoride-Mediated Dephosphonation for C–C Coupling Reactions. *Org. Lett.* **2018**, *20* (13), 3819–3823. https://doi.org/10.1021/acs.orglett.8b01406.

(15) Kim, E.; Koh, M.; Lim, B. J.; Park, S. B. Emission Wavelength Prediction of a Full-Color-Tunable Fluorescent Core Skeleton, 9-Aryl-1,2-Dihydropyrrolo[3,4-b]Indolizin-3-One. *J. Am. Chem. Soc.* **2011**, *133* (17), 6642–6649. https://doi.org/10.1021/ja110766a.

(16) Lin, Q.; Hu, B.; Xu, X.; Dong, S.; Liu, X.; Feng, X. Chiral N,N′-Dioxide/Mg(OTf)2 Complex-Catalyzed Asymmetric [2,3]-Rearrangement of in Situ Generated Ammonium Salts. *Chem. Sci.* **2020**, *11* (11), 3068–3073. https://doi.org/10.1039/c9sc06342k.
(17) Grantham, H. F.; Lee, R. J.; Wardas, G. M.; Mistry, J.-R.; Elsegood, M. R. J.; Wright, I. A.; Pritchard, G. J.; Kimber, M. C. Transition-Metal-Free Continuous-Flow Synthesis of 2,5-Diaryl Furans: Access to Medicinal Building Blocks and Optoelectronic Materials. *J. Org. Chem.* **2024**, *89* (1), 484–497. https://doi.org/10.1021/acs.joc.3c02237.
(18) Davis, M. C.; Groshens, T. J. Synthesis of P-Quinquephenyl from E,E-1,4-Bis(4-Bromophenyl)-1,3-Butadiene. *Synth. Commun.* **2010**, *41* (2), 206–218. https://doi.org/10.1080/00397910903533991.
(19) Yu, J.; Zhou, Y.; Lin, Z.; Tong, R. Regioselective and Stereospecific Copper-Catalyzed Deoxygenation of Epoxides to Alkenes. *Org. Lett.* **2016**, *18* (18), 4734–4737. https://doi.org/10.1021/acs.orglett.6b02405.
(20) Shang, W.; Duan, D.; Liu, Y.; Lv, J. Carbocation Lewis Acid TrBF4-Catalyzed 1,2-Hydride Migration: Approaches to (Z)-α,β-Unsaturated Esters and α-Branched β-Ketocarbonyls. *Org. Lett.* **2019**, *21* (19), 8013–8017. https://doi.org/10.1021/acs.orglett.9b03005.
(21) Wolf, T.; Rheinberger, T.; Simon, J.; Wurm, F. R. Reversible Self-Assembly of Degradable Polymersomes with Upper Critical Solution Temperature in Water. *J. Am. Chem. Soc.* **2017**, *139* (32), 11064–11072. https://doi.org/10.1021/jacs.7b02723.
(22) Hemric, B. N.; Chen, A. W.; Wang, Q. Copper-Catalyzed 1,2-Amino Oxygenation of 1,3-Dienes: A Chemo-, Regio-, and Site-Selective Three-Component Reaction with O-Acylhydroxylamines and Carboxylic Acids. *ACS Catal.* **2019**, *9* (11), 10070–10076. https://doi.org/10.1021/acscatal.9b03076.
(23) Fujiwara, Y.; Domingo, V.; Seiple, I. B.; Gianatassio, R.; Del Bel, M.; Baran, P. S. Practical C−H Functionalization of Quinones with Boronic Acids. *J. Am. Chem. Soc.* **2011**, *133* (10), 3292–3295. https://doi.org/10.1021/ja111152z.
(24) Miyamura, H.; Shiramizu, M.; Matsubara, R.; Kobayashi, S. Aerobic Oxidation of Hydroquinone Derivatives Catalyzed by Polymer-Incarcerated Platinum Catalyst. *Angew. Chem. Int. Ed.* **2008**, *47* (42), 8093–8095. https://doi.org/10.1002/anie.200802192.
(25) Nawrat, C. C.; Lewis, W.; Moody, C. J. Synthesis of Amino-1,4-Benzoquinones and Their Use in Diels–Alder Approaches to the Aminonaphthoquinone Antibiotics. *J. Org. Chem.* **2011**, *76* (19), 7872–7881. https://doi.org/10.1021/jo201320g.
(26) Huang, C.; Li, S.; Thakellapalli, H.; Farajidizaji, B.; Huang, Y.; Akhmedov, N. G.; Popp, B. V.; Petersen, J. L.; Wang, K. K. Synthesis of Partially Hydrogenated Cycloparaphenylenes with Bent and Fused Structures Bearing Armchair Carbon Nanotube-like Connections. *J. Org. Chem.* **2017**, *82* (2), 1166–1174. https://doi.org/10.1021/acs.joc.6b02789.
(27) Savka, R.; Bergmann, M.; Kanai, Y.; Foro, S.; Plenio, H. Triptycene-Based Chiral and meso-N-Heterocyclic Carbene Ligands and Metal Complexes. *Chem. Eur. J.* **2016**, *22* (28), 9667–9675. https://doi.org/10.1002/chem.201601474.
(28) Sarkar, R.; Mukherjee, S. Catalytic Enantioselective Desymmetrization of Norbornenoquinones via C(Sp2)–H Alkylation. *Org. Lett.* **2016**, *18* (23), 6160–6163. https://doi.org/10.1021/acs.orglett.6b03168.
(29) Liu, D.; Canales, E.; Corey, E. J. Chiral Oxazaborolidine−Aluminum Bromide Complexes Are Unusually Powerful and Effective Catalysts for Enantioselective Diels−Alder Reactions. *J. Am. Chem. Soc.* **2007**, *129* (6), 1498–1499. https://doi.org/10.1021/ja068637r.
(30) Thompson, L. C.; Scheerer, J. R. Synthesis of 2-Azaanthraquinones from 1,4-Oxazinone Precursors. *J. Org. Chem.* **2025**, *90* (28), 9885–9891. https://doi.org/10.1021/acs.joc.5c00881.
(31) Lin, C.-H.; Lin, K.-H.; Pal, B.; Tsou, L.-D. Iterative Synthesis of Acenesvia Homo-Elongation. *Chem. Commun.* **2009**, No. 7, 803–805. https://doi.org/10.1039/B814840F.
(32) Kitazawa, K.; Kochi, T.; Sato, M.; Kakiuchi, F. Convenient Synthesis of Tetra- and Hexaarylanthracenes by Means of RuH2(CO)(PPh3)3-Catalyzed C−H Arylation of Anthraquinone with Arylboronates. *Org. Lett.* **2009**, *11* (9), 1951–1954. https://doi.org/10.1021/ol900393x.
